# Validation of the ITS2 Region as a Novel DNA Barcode for Identifying Medicinal Plant Species

**DOI:** 10.1371/journal.pone.0008613

**Published:** 2010-01-07

**Authors:** Shilin Chen, Hui Yao, Jianping Han, Chang Liu, Jingyuan Song, Linchun Shi, Yingjie Zhu, Xinye Ma, Ting Gao, Xiaohui Pang, Kun Luo, Ying Li, Xiwen Li, Xiaocheng Jia, Yulin Lin, Christine Leon

**Affiliations:** 1 Institute of Medicinal Plant Development, Chinese Academy of Medical Sciences, Peking Union Medical College, Beijing, People's Republic of China; 2 Li Ka Shing Faculty of Medicine, The University of Hong Kong, Hong Kong, People's Republic of China; 3 Department of Pharmacy, Hubei University of Chinese Medicine, Wuhan, Hubei, People's Republic of China; 4 Royal Botanic Gardens, Kew, Richmond, United Kingdom; Niels Bohr Institute and Biological Institutes, Denmark

## Abstract

**Background:**

The plant working group of the Consortium for the Barcode of Life recommended the two-locus combination of *rbcL* + *matK* as the plant barcode, yet the combination was shown to successfully discriminate among 907 samples from 550 species at the species level with a probability of 72%. The group admits that the two-locus barcode is far from perfect due to the low identification rate, and the search is not over.

**Methodology/Principal Findings:**

Here, we compared seven candidate DNA barcodes (*psbA-trnH*, *matK*, *rbcL*, *rpoC1*, *ycf5*, ITS2, and ITS) from medicinal plant species. Our ranking criteria included PCR amplification efficiency, differential intra- and inter-specific divergences, and the DNA barcoding gap. Our data suggest that the second internal transcribed spacer (ITS2) of nuclear ribosomal DNA represents the most suitable region for DNA barcoding applications. Furthermore, we tested the discrimination ability of ITS2 in more than 6600 plant samples belonging to 4800 species from 753 distinct genera and found that the rate of successful identification with the ITS2 was 92.7% at the species level.

**Conclusions:**

The ITS2 region can be potentially used as a standard DNA barcode to identify medicinal plants and their closely related species. We also propose that ITS2 can serve as a novel universal barcode for the identification of a broader range of plant taxa.

## Introduction

The World Health Organization estimates that 80 percent of the world's population utilizes traditional medicines for healing and curing diseases (http://www.worldwildlife.org/what/globalmarkets/wildlifetrade/faqs-medicinalplant.html). There is an increasing international market for medicinal plants, which are used both for herbal medicine and for pharmaceutical products. Medicinal plants cover a wide range of plant taxa and closely related species. According to surveys in China, medicinal plants belong to 11,146 species from 2,309 genera of 383 families, representing a rich biodiversity. Accurate and rapid authentication of these plants and their adulterants is difficult to achieve at the scale of international trade in medicinal plants. We aim to provide a practical and powerful tool for identifying medicinal plants and their adulterants in trade and for ensuring safety in their use.

**Figure 1:**
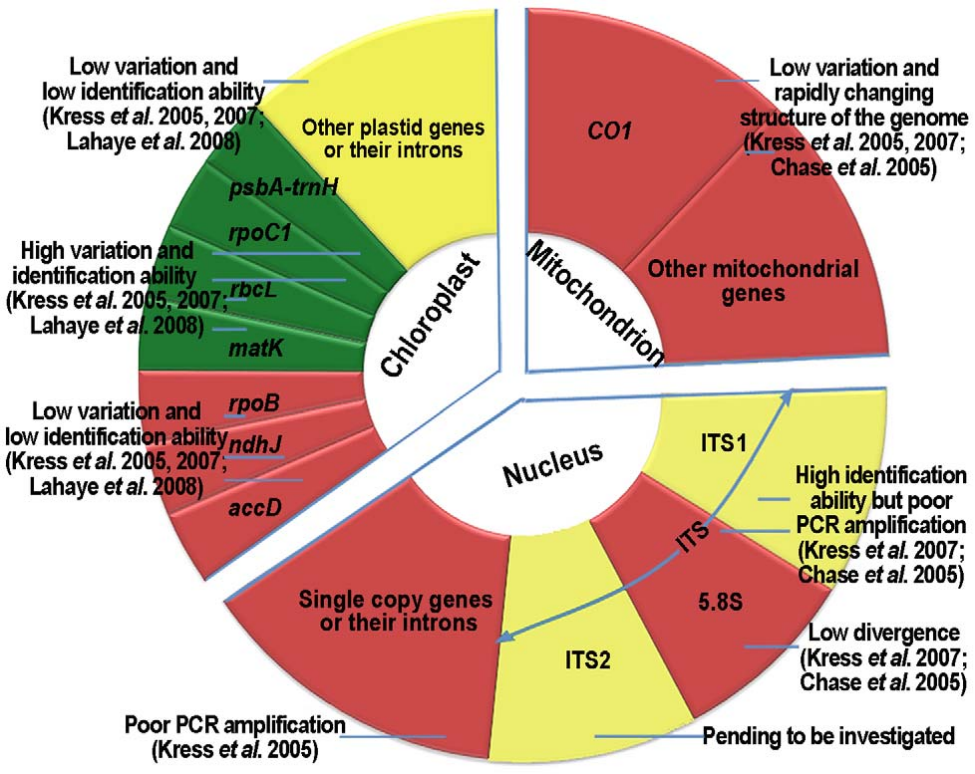
Genes from three genomes in plants that are candidate barcodes. Green markers are potential barcodes, red markers are poor candidates and yellow markers are pending to be investigated.

The term “DNA barcode” for global species identification was first coined by Hebert in 2003 [1], [2] and has gained worldwide attention in the scientific community [3]–[7]. Recognition of animals, plants and fungi has been performed using this technique [8]–[12]. Most researchers agree that the mitochondrial gene encoding cytochrome c oxidase subunit 1 is a favorable region for use as a DNA barcode in most animal species and even in some fungal species, including those of the groups *Ascomycota*, *Basidiomycota* and *Chytridiomycota*. However, the *CO1* gene and other mitochondrial genes from plants have limited usefulness for identifying plant species across a wide range of taxa due to the low amounts of variation in the genes, as well as the variable structure of the mitochondrial genome [9], [13]–[16]. Thus, screening for single or multiple regions appropriate for DNA barcoding studies in nuclear and plastid genomes in plants has been an important research focus (Fig. 1).

First, most single-copy genes in the nuclear genome, as well as their introns, have been excluded as barcode candidates because of the lack of universal primers for their amplification [9]. However, with the exception of 5.8S, the internal transcribed spacer (ITS) of nuclear ribosomal DNA and regions of the ITS could be potential barcodes [9], [14] (Fig. 1). Second, extensive studies have focused on genes and introns of the chloroplast genome. For example, Kress *et al.* [9] compared 10 loci for authenticating closely related species in 7 plant families and 99 species belonging to 88 genera in 53 families, and they reported that the *psbA-trnH* spacer and the internal transcribed spacer could be used as a pair of potential barcodes for identifying widely divergent angiosperm taxa. However, this pair failed to distinguish members of the order *Cycadales* [17]. Lahaye *et al.* [18] analyzed 1084 plant species (nearly 96% orchid species) and demonstrated that a portion of the plastid *matK* gene could be a universal DNA barcode for flowering plants. In another study, two separate regions (*matK* and *psbA-trnH*) had significant sequence variations and correctly discriminated 95% of 40 nutmeg samples representing 10 species in the Myristicaceae family [19]. Newmaster *et al.* [20] analyzed over 10,000 *rbcL* sequences from GenBank and found that *rbcL* could discriminate samples in approximately 85% of pairwise comparisons of congeneric species, whereas the discrimination efficiency was about 88% when a combination of *psbA-trnH* and *rbcL* was used across 96 diverse species of 48 genera from 43 families [21]. Altogether, although no single plant marker has been found that works as well as the COI in animals, several markers of the plastid genome, such as *psbA-trnH*, *matK*, *rbcL* and *rpoC1*, have shown superior qualities as DNA barcodes relative to *accD*, *ndhJ* and *rpoB* [9], [13], [18], [22], [23] (Fig. 1).

There are two groups of potential users of DNA barcodes: plant taxonomists/systematists and scientists in other fields [14], [24]. In addition, DNA barcodes will be a useful and powerful tool for non-professional users such as customs officers, traditional drug producers and managers and forensic specialists. Therefore, a rapid and simple DNA barcoding identification system, even an imperfect one, is likely to be welcomed.

In this study, we tested seven potential DNA regions (*psbA-trnH*, *matK*, *rbcL*, *rpoC1*, *ycf5*, ITS2 and ITS) for their suitability as DNA barcodes across 8557 medicinal plants and closely related samples belonging to 5905 species from 1010 diverse genera of 219 families in 7 phyla (Angiosperms, Gymnosperms, Ferns, Mosses, Liverworts, Algae and Fungi). These plants have a long history of use in traditional herbal medicines; included here are species from the Chinese and the Japanese Pharmacopoeias, as well as a few from the European Pharmacopoeia. The seven candidate DNA barcodes were compared using several criteria. Four of the loci (*rbcL*, *rpoC1*, *matK* and *ycf5*) were proposed by the Plant Working Group (www.kew.org/barcoding). As for the other three, *psbA-trnH* and nrITS were recommended by Kress *et al.* [9] and ITS2 was first described by Chiou *et al.* [25]. The ITS2 region was selected as a barcode candidate because ITS2 sequences are potential general phylogenetic markers and are widely used for phylogenetic reconstructions at both the genus and species levels [26]–[30]. The search for and development of herbal medicines is rapidly increasing worldwide, so practical and accurate authentication resources are urgently needed [31]–[34]. Our study shows the potential for a DNA barcoding technique to become a standard for the authentication of medicinal plants and their adulterants.

## Results

### Efficiency of PCR Amplification

The success rate of PCR amplification with four pairs of primers for ITS1 was poor in our pilot study, so ITS1 was not included in subsequent experiments. Two pairs of ITS2 primers designed by Chiou *et al.* [25] and one designed for this study failed to amplify the sequences in gymnosperms and ferns. The primer pairs with the highest success rate for each barcode are listed in Table S1. These rates were obtained in our pilot study. For example, we compared the PCR amplification efficiency of *psbA-trnH*, ITS2 and ITS sequences across 400 samples belonging to 326 species in 98 families including dicots, monocots, gymnosperms and ferns. The success rates for *psbA-trnH* and ITS2 sequences were 92.8% and 93.8%, respectively, while ITS fragments were only successfully amplified in 42.3% of the experiments (Table 1). Furthermore, we also calculated the efficiency of PCR amplification in total number of samples (Table S2). Results showed that *rpoC1* provided the highest rate, followed by *psbA-trnH* and ITS2.

**Table 1:** Efficiency of PCR amplification of three potential barcodes in a wide range of plant taxa.

Category	No. of families	No. of genera	No. of species	No. of samples	PCR efficiency of ITS2 (%)	PCR efficiency of *psbA-trnH* (%)	PCR efficiency of ITS (%)
**Angiosperms**	78	218	281	347	96.0	93.7	48.1
**Dicotyledons**	70	204	266	326	96.9	93.9	49.4
**Monocotyledons**	8	14	15	21	81.0	90.5	28.6
**Gymnosperms**	10	15	32	37	91.9	89.2	5.4
**Ferns**	10	12	13	16	50.0	81.3	0.0
**Total**	98	245	326	400	93.8	92.8	42.3

### Determination of Genetic Divergence Using Six Parameters

**Figure 2:**
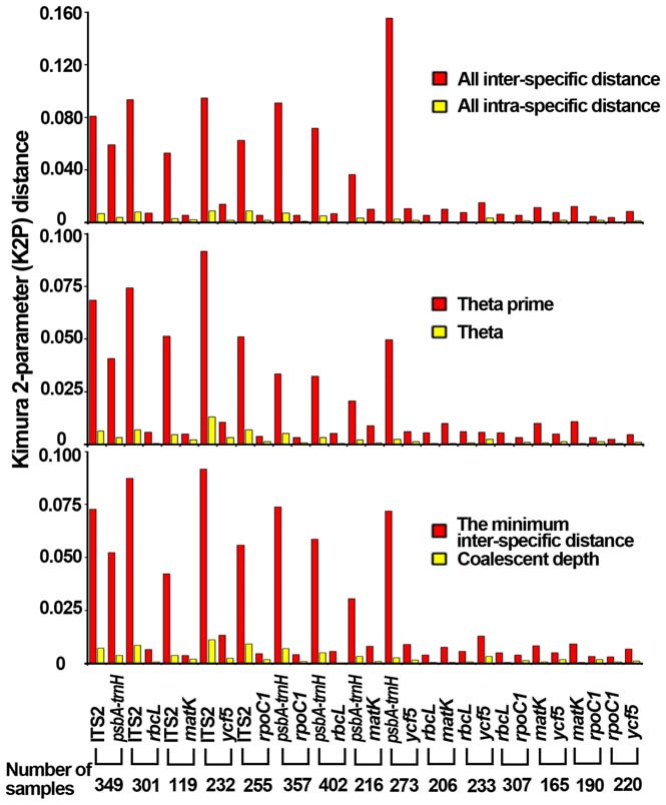
Inter-specific divergence between congeneric species and intra-specific variation of paired loci.

First, three parameters were used to characterize inter-specific divergence [35], [36]: (i) average inter-specific distance (K2P distance) between all species in each genus with at least two species; (ii) average theta prime (θ′), where theta prime is the mean pairwise distance within each genus with more than one species, thus eliminating biases associated with different numbers of species among genera; and (iii) smallest inter-specific distance, i.e., the minimum inter-specific distance within each genus with at least two species. A favorable barcode should possess high inter-specific divergence in order to distinguish different species. In comparisons of inter-specific genetic distances among congeneric species using six candidate barcodes, the ITS2 region exhibited the highest inter-specific divergence according to all three parameters, followed by *psbA-trnH* (Fig. 2 and Table 2), while *rpoC1* provided the lowest. Moreover, Wilcoxon signed rank tests confirmed that ITS2 and *psbA-trnH* provided the highest inter-specific divergence between congeneric species, whereas the lowest belonged to *rpoC1* (Table S3).

**Table 2:** Analysis of inter-specific divergence between congeneric species and intra-specific variation of candidate barcodes.

Marker	*psbA-trnH*	ITS2	*matK*	*rbcL*	*rpoC1*	*ycf5*
**All inter-specific distance**	0.0340±0.0809	0.0705±0.0821	0.0098±0.0134	0.0061±0.0064	0.0035±0.0065	0.0062±0.0134
**Theta prime**	0.0567±0.1106	0.0855±0.0934	0.0103±0.0169	0.0076±0.0089	0.0057±0.0101	0.0140±0.0279
**Minimum inter-specific distance**	0.0459±0.1001	0.0732±0.0916	0.0079±0.0158	0.0062±0.0089	0.0043±0.0094	0.0126±0.0281
**All intra-specific distance**	0.0065±0.0290	0.0090±0.0207	0.0012±0.0027	0.0005±0.0012	0.0014±0.0049	0.0016±0.0046
**Theta**	0.0041±0.0202	0.0087±0.0193	0.0009±0.0023	0.0004±0.0009	0.0018±0.0060	0.0013±0.0034
**Coalescent depth**	0.0068±0.0290	0.0102±0.0227	0.0011±0.0026	0.0005±0.0012	0.0019±0.0060	0.0016±0.0043

Second, three additional parameters were used to determine intra-specific variation [18], [35]: (i) average intra-specific difference (K2P distance), that between all samples collected within each species with more than one individual; (ii) theta (θ), where theta is the mean pairwise distance within each species with at least two representatives; θ eliminates biases associated with unequal sampling among a species; and (iii) average coalescent depth, which is the maximum intra-specific distance within each species with at least two individuals. Here, *rbcL* showed the lowest level of intra-specific variation with all three parameters, while ITS2 still exhibited the highest level of variation with all three parameters, followed by *psbA-trnH* (Fig. 2 and Table 2). For intra-specific divergence, Wilcoxon signed rank tests indicated that *rbcL*, *rpoC1* and *matK* showed the lowest variation between conspecific individuals, whereas ITS2 showed the highest (Table S4).

Similarly, the candidate DNA barcodes ITS2 and *psbA-trnH* were found to have high inter-specific divergence and high intra-specific variation using the six parameters and statistical tests described above. This analysis demonstrated that ITS2 and *psbA-trnH* sequences represent the most suitable DNA barcodes to meet our goal. Their further evaluation was then assessed using two other criteria: DNA barcoding gap and authentication ability.

### Assessment of Barcoding Gap

**Figure 3:**
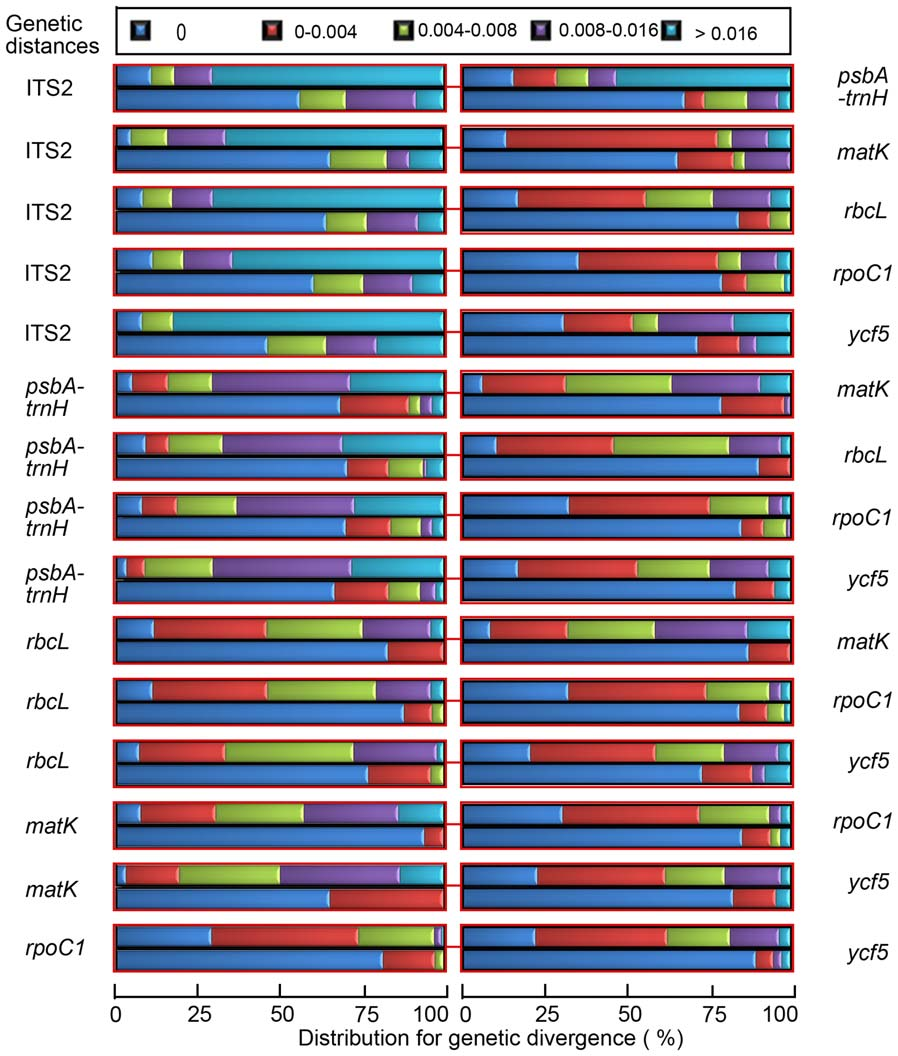
Distribution of inter-specific divergence between congeneric species and intra-specific variation for paired loci. Two color bars in each box represent inter-specific (above) and intra-specific (below) genetic distances.

In an ideal situation, genetic variation of a DNA barcode should demonstrate separate, non-overlapping distributions between intra- and inter-specific samples. Meyer *et al.* and Moritz *et al.* [35], [37] demonstrated that when the number of closely related species is increased, the overlap of genetic variation without barcoding gaps significantly increases. Our results demonstrated that the distributions of intra- and inter-specific variation of *psbA-trnH* and ITS2 exhibited distinct gaps, but when intra-specific variation between conspecific individuals and inter-specific divergence between all hetero-specifics were calculated using *matK*, *rbcL*, *ycf5* and *rpoC1*, there was significant overlap without gaps (Fig. S1). However, when intra-specific variation between conspecific individuals and inter-specific divergence between congeneric species were computed, i.e., the proportion of closely related species was enhanced, none of the barcodes revealed large gaps (Fig. 3 and Fig. S2). Furthermore, Wilcoxon's two-sample tests showed that, for six barcodes (*psbA-trnH*, ITS2, *matK*, *ycf5*, *rbcL* and *rpoC1*), the mean of the inter-specific divergences was significantly higher than that of the corresponding intra-specific variations (Fig. S3). Therefore, *psbA-trnH* and ITS2 pass this test, as they possess intra- and inter-specific variation gaps.

### Evaluation of Species Authentication Capability of Barcodes

To estimate the reliability of species identification using a DNA barcoding technique, two methods (BLAST1 and the nearest genetic distance) were used [12]. The BLAST1 method determines the identity of a sample based on the best hit of the query sequence and the E-value for the match must be less than a cutoff value. In comparison, the nearest genetic distance method determines the identity of a sample based on which sequence in the database has the smallest genetic distance from the query sequence; this distance must be less than a distance threshold. We first retrieved all ITS2 and *psbA-trnH* sequences and constructed a reference sequence library. We then searched the database with the sequences generated in this study from samples with proven taxonomic identity. The species identities of these query sequences were then determined using the BLAST1 or nearest genetic distance method.

The results indicated that, using the BLAST1 method, ITS2 correctly identified 92.7% and 99.8% of the samples at the species and genus level, respectively. When the nearest genetic distance method was used, ITS2 correctly identified 90.3% and 99.7% of the samples at the species and genus level, respectively (Table 3). In contrast to ITS2, the success rates of *psbA-trnH* were much lower (67.6% and 72.8%) at the species level using the BLAST1 and nearest genetic distance methods, respectively. However, *psbA-trnH* had a >95% success rate of identification at the genus level (Table 3). Our data demonstrate that, when samples from a wide range of plant taxa are tested, ITS2 possesses a higher success rate in species and genus identification compared to *psbA-trnH*. Therefore, the ITS2 region is a powerful universal barcode, and as such, it is a promising candidate for authenticating all major plant taxa used in traditional herbal medicine. The *psbA-trnH* spacer may be used as a complementary barcode.

**Table 3:** Identification efficiency for ITS2 and *psbA-trnH* loci using different methods of species identification.

Marker	Method of species identification	Plant taxa level	Correct identification (%)	Incorrect identification (%)	Ambiguous identification (%)
**ITS2**	BLAST	Species	92.7	0.0	7.3
		Genus	99.8	0.0	0.2
	Distance	Species	90.3	0.0	9.7
		Genus	99.7	0.0	0.3
***psbA-trnH***	BLAST	Species	67.6	0.0	32.4
		Genus	95.4	0.0	4.6
	Distance	Species	72.8	0.0	27.2
		Genus	96.5	0.0	3.5

## Discussion

In contrast to other studies, our study presents a strong case for the ITS2 region being the most promising universal DNA barcode for authenticating medicinal plants, as assessed against several criteria. First, at 160–320 base pairs, the ITS2 region is short. As a result, ITS2 sequences are relatively easy to amplify using one pair of universal primers selected by our group (Table S1). Second, determination of genetic divergence using six parameters and statistical tests confirmed that the ITS2 region possesses high inter-specific divergence (Fig. 2, Table 2, S3) and is well separated. Analyses of the DNA barcoding gap and Wilcoxon two-sample tests support the notion that the mean inter-specific divergence of the ITS2 region is significantly higher than its mean intra-specific variation (Fig. 3, S1-S3). Third, according to the BLAST1 method, for 6685 samples from 4800 species in 753 genera of 193 families, identification accuracies using the ITS2 region were 92.7% and 99.8% at the species and genus level, respectively. The plant samples represented lower and higher plants (Angiosperms, Gymnosperms, Ferns, Mosses, Liverworts, Algae and Fungi), along with a series of closely related species. To our knowledge, this is by far one of most comprehensive samples of plants reported in the literature. The inclusion of many closely related species supports the notion that the ITS2 region is not only capable of discriminating plant taxa from different plant families but is also able to distinguish closely related taxa at the genus and species levels. This finding suggests that, similar to *CO1* in animals, the ITS2 region in plants is a suitable DNA barcode for authenticating taxa at different taxonomic levels.

Although the ITS2 region possesses many advantages compared to plastid genomic fragments and other nuclear genomic regions, including ITS, other researchers have not given sufficient attention to this region. Previous studies have suggested that ITS1 and ITS exhibit higher inter-specific divergence relative to ITS2 and *psbA-trnH*, which were used for further testing [9], [21]. However, universal primers for ITS1 and ITS have not been identified for broad taxonomic use, leading to low amplification efficiency and the need for specific PCR conditions and additives [16], [21]. Our results confirmed these previous observations. Nevertheless, the potential of ITS2 as a suitable marker applicable for taxonomic classification and phylogenetic reconstructions has already been demonstrated using Eukaryota [26]–[29], [38], [39]. This finding contributed to the discovery of a conserved core of the secondary structure of ITS2 in green and brown algae, land plants and most animals [27]. Coleman argued that ITS2 has many advantages: a size of a few hundred nucleotides, comparison of relationships from the subspecies to the order levels, double-checking possible sequence errors in alignments directed by secondary structure, etc. Based on this evidence and our own findings, we propose that ITS2 should be a gold standard barcode for identifying plants and fungi [39]. At the recent Barcode Conference in Mexico City, it was reported that a significant portion of the ITS2 GenBank records from plants are likely to represent fungal sequences from endophytes. We checked the plant ITS2 sequences in our experiments using BLAST analysis (e-value<0.001) and Hidden-Markov-Model (HMM)-based ITS2 annotation methods (fungal model, e-value<0.001) and did not find any fungal sequences. Further, we also checked 6022 plant ITS2 sequences from GenBank used for our analysis. Indeed, 5 plant ITS2 sequences (Accession Numbers: AM920396, AM920397, AM920401, AM920402, and AM920403) may represent fungal sequences. Although the overall ratio is less than one in one thousand, it is very important for researchers to verify the sequences from GenBank.

The present study also evaluated a chloroplast non-coding region, *psbA-trnH*, and compared to the ITS2 region, it also demonstrated excellent reliability for species authentication. For 2108 plant samples representing 1433 species of 551 genera in 135 families from 4 phyla (Angiosperms, Gymnosperms, Ferns and Mosses), the identification rate of the *psbA-trnH* region was 96.5% at the genus level using the nearest distance method; however, this rate was lower, 72.8%, at the species level. In previous studies, most researchers accepted *psbA-trnH* as a potential plant barcode [9], [15], [16], [18], [19], [21], [33]. Similar to our findings (Table 2, S3), the inter-specific divergence of the *psbA-trnH* locus is higher than that of other plastid loci investigated [9], [21], even though the *matK* locus only demonstrated half of the inter-specific divergence of the *psbA-trnH* locus [18]. Therefore, we strongly recommend *psbA-trnH* as a complementary barcode to ITS2 for a broad series of plant taxa.

Comparing to ITS2 and *psbA-trnH*, ITS was rejected as a universal barcode due to the low PCR efficiency (Table 1), while *rpoC1* showed the lowest inter-specific divergence (Table 2, S3, Fig. 2), thus not all samples was amplified for *rpoC1* despite the highest PCR efficiency (Table S2). In our experiments, ITS2, *psbA-trnH*, *rpoC1*, and *rbcL* provided not bad PCR efficiency (80%–96%) and not satisfactory sequencing efficiency (63%–73%), because AT-rich or homologous sequences existed, or concentration of PCR products was not high enough. Thus sequencing technology should be improved to obtain more sequences with high quality. Anyway, the fact that ITS2 region is not a coding region but possesses a conserved core of the secondary structure promotes establishment of data handling systems [30]. Recently, the CBOL (Consortium for the Barcode of Life) plant working group recommended using the 2-locus combination of *rbcL* + *matK* as a plant barcode, yet the barcode was shown to successfully discriminate among 907 samples from 550 species at the species level with a probability of 72% [40]. The group admits that the two-locus barcode is far from perfect, and the search is not over [41]. In our study, for ITS2 data, it is convenient (90%–93%) to identify more than 6600 samples from 4800 species using BLAST1 and the nearest genetic distance methods. We believe that the ITS2 region should be a standard barcode applied to international trade and safe use of medicinal plants.

## Materials and Methods

### Taxon Sampling

To select the most suitable DNA barcoding fragments, a total of 8557 medicinal plants and closely related samples belonging to 5905 species from 1010 diverse genera of 219 families in 7 phyla (Angiosperms, Gymnosperms, Ferns, Mosses, Liverworts, Algae and Fungi) were used (Table S5, S6, S7). A first set of plant samples collected in nine provinces of China (Beijing, Guangxi, Yunnan, Hainan, Sichuan, Fujian, Chongqing, Jilin and Hubei) was used to test seven potential DNA barcode regions (Table S5). All corresponding voucher samples are curated in the Herbarium of the Institute of Medicinal Plant Development, Chinese Academy of Medicinal Sciences. A second set of plant samples was used for testing the selected potential barcodes (Table S5-S7), including a broader range of plant taxa with emphasis on closely related species. The selection of the first dataset was made mainly according to the Chinese Pharmacopoeia and Flora of China. This set of samples is of great medical and economic importance. The second sample set was selected to represent lower and higher plants (Angiosperms, Gymnosperms, Ferns, Mosses, Liverworts, Algae and Fungi).

### PCR Amplification and Sequencing of Candidate DNA Barcodes

Leaf tissues were first dried in silica gel. Ten milligrams of each of the dried tissues was rubbed for one minute at a frequency of 30 times/second in a FastPrep bead mill (Retsch MM400, Germany). DNA extractions were performed using the Plant Genomic DNA Kit (Tiangen Biotech Co., China) according to the manufacturer's instructions. The sequences of the universal primers for the DNA barcode to be tested, including those for *psbA-trnH*, *matK*, *rbcL*, *rpoC1*, *ycf5* and ITS, and general PCR reaction conditions were obtained from previous studies [9], [17], [18], [21]. Based on the conserved regions of 18S and 5.8S, we designed four pairs of primers for ITS1. Similarly, according to a previous study [25] and the conserved regions of 5.8S and 26S, we also designed four pairs of primers for ITS2. PCR amplification was performed in 25-µl reaction mixtures containing approximately 30 ng of genomic DNA template, 1 X PCR buffer without MgCl_2_, 2.0 mM MgCl_2_, 0.2 mM of each dNTP, 0.1 µM of each primer (synthesized by Sangon Co., China) and 1.0 U *Taq* DNA Polymerase (Biocolor BioScience & Technology Co., China), with a Peltier Thermal Cycler PTC0200 (BioRad Lab, Inc., USA). Purified PCR products were sequenced in both directions with the primers used for PCR amplification on a 3730XL sequencer (Applied Biosystems, USA). To estimate the quality of the generated sequence traces, the original forward and reverse sequences were assembled using CodonCode Aligner 3.0 (CodonCode Co., USA). Base calling was carried out using the Phred program (version no. 0.020425.c). The quality values were defined for three levels: low quality (0 to 19 QV), medium quality (20 to 30 QV) and high quality (higher than 30 QV). The sequences showing >2 bases with a quality value below 20 QV in a 20-base window were trimmed. The forward and reverse reads have a minimum length of 100 bp, a minimum average QV of 30, and the post-trim lengths should be >50% of the original read length. In addition, the assembled contig should have a minimum average QV score of 40 and >50% overlap in the alignment of the forward and reverse reads. All sequences of the second set of plant samples containing the “internal transcribed spacer 2”or “*psbA-trnH*” were retrieved according to Keller *et al*. [42] and GenBank annotations. Subsequences marked as ITS2 or *psbA-trnH* intergenic spacer were recovered after deleting sequences with ambiguous nucleotides and those shorter than 100 bp.

### Sequence Alignment, Genetic Analysis and Species Identification

Candidate DNA barcodes were aligned by Clustal W and Kimura 2-Parameter (K2P) distances were computed with PAUP4b10 (Florida State University, USA). Average intra-specific distances, theta and coalescent depth were calculated to determine intra-specific variation using a K2P distance matrix [35]. Average inter-specific distance, theta prime and smallest inter-specific distance were used to characterize inter-specific divergence [35], [36]. Wilcoxon signed rank tests were performed as described previously [18], [21]. The distribution of intra- versus inter-specific variability was compared using DNA barcoding gaps [18], [35]. Two methods of species identification, including BLAST1 and the nearest distance method, were performed as described previously [12].

## Supporting Information

Figure S1 The barcoding gap between inter-specific and intra-specific divergences for six candidate barcodes. (A) ITS2. (B) psbA-trnH. (C) matK. (D) rbcL. (E) ycf5. (F) rpoC1.
(0.64 MB TIF)

Figure S2 The presence/absence of barcode gaps. The percentage of species pairs with dintra/dinter ratios <1 were determined for six candidate regions including ITS2, psbA-trnH, matK, rbcL, ycf5, and rpoC1 to be 73.3%, 73.7%, 47.4%, 69.0%, 60.0%, and 35.7%, respectively. Therefore, ITS2 and psbA-trnH have significant barcode gaps.
(0.58 MB TIF)

Figure S3 Wilcoxon two-sample tests for the divergences of paired loci with the same set of samples. Inter and Intra mean number of inter-specific distances and number of intra-specific distances, respectively.
(0.55 MB TIF)

Table S1 List of universal primers and reaction conditions for candidate barcodes.
(0.06 MB DOC)

Table S2 Efficiency of PCR amplification of potential barcodes in total number of samples.
(0.03 MB DOC)

Table S3 Wilcoxon signed rank tests for inter-specific divergence.
(0.05 MB DOC)

Table S4 Wilcoxon signed rank tests for intra-specific variation.
(0.04 MB DOC)

Table S5 Samples for testing potential barcodes and accession numbers in GenBank.
(1.23 MB DOC)

Table S6 Samples for determining the ability of the ITS2 barcode to identify species and accession numbers in GenBank.
(5.25 MB DOC)

Table S7 Samples for determining the ability of the psbA-trnH barcode to identify species and accession numbers in GenBank.
(1.28 MB DOC)
