# Supplementary figures and images for: Validation of the ITS2 Region as a Novel DNA Barcode for Identifying Medicinal Plant Species
Source: PLoS One. 2010 Jan 7;5(1):e8613. doi: 10.1371/journal.pone.0008613 (PMC2799520; doi:10.1371/journal.pone.0008613)

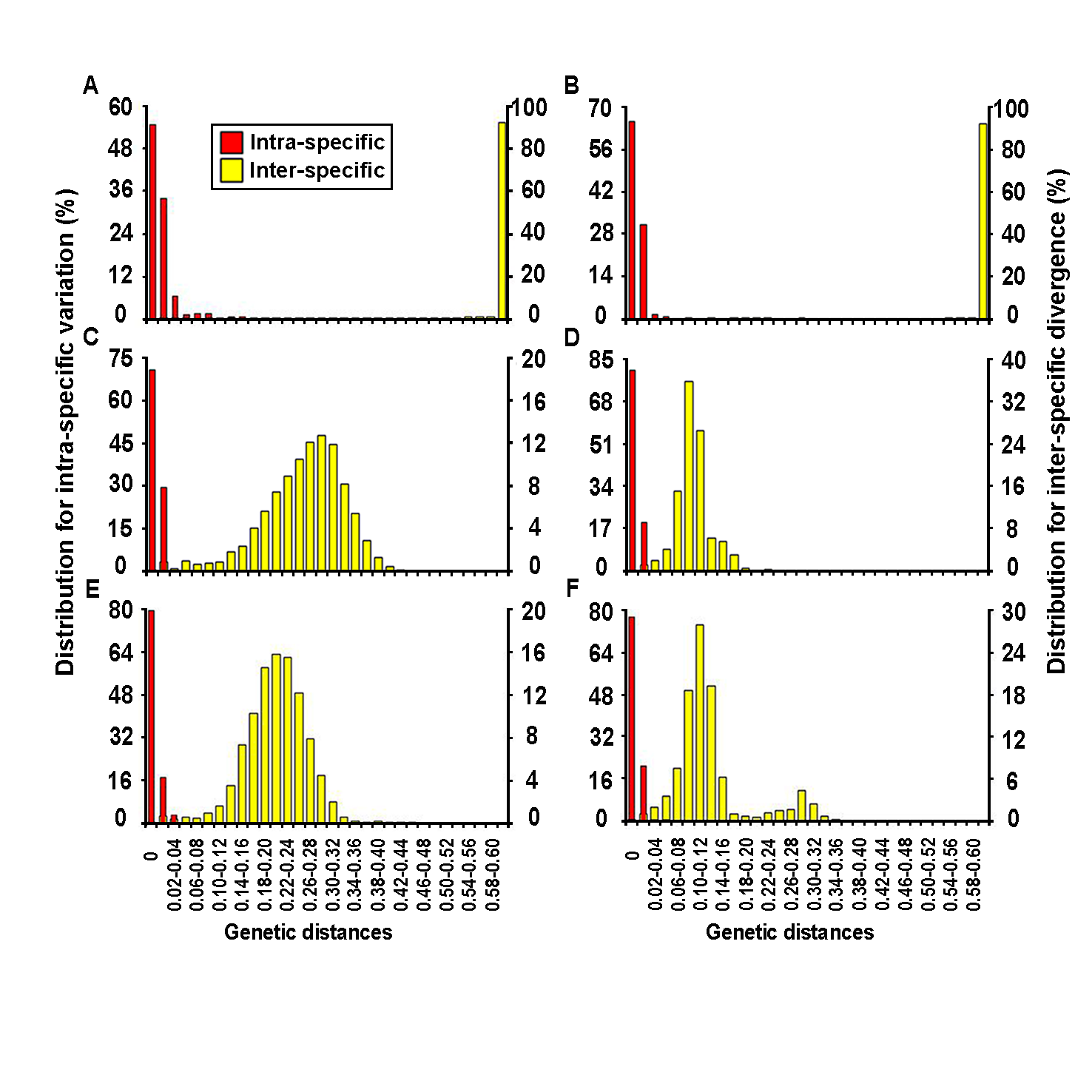

Supplement: Figure S1 — The barcoding gap between inter-specific and intra-specific divergences for six candidate barcodes. (A) ITS2. (B) psbA-trnH. (C) matK. (D) rbcL. (E) ycf5. (F) rpoC1. (0.64 MB TIF) [file pone.0008613.s001.tif]

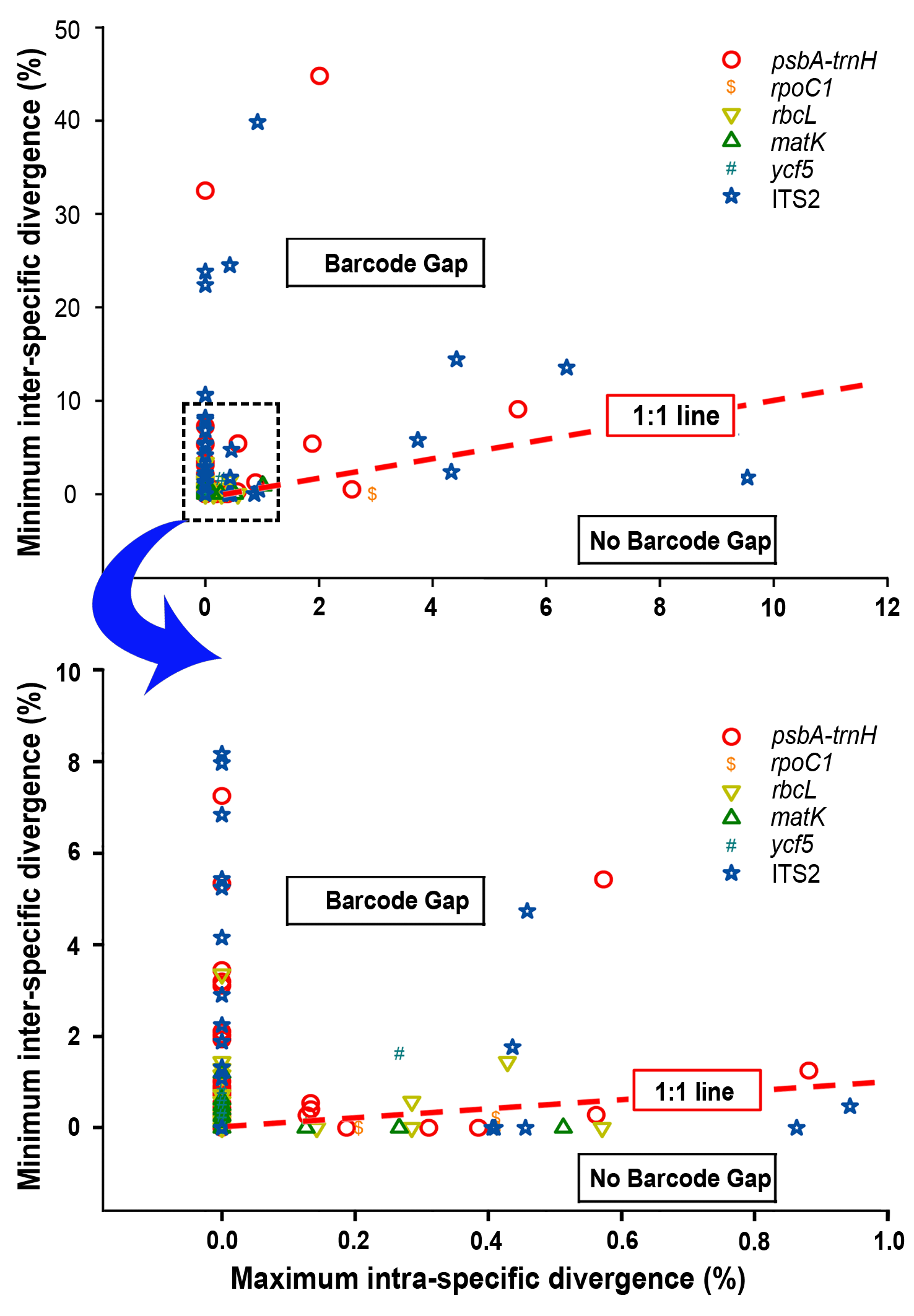

Supplement: Figure S2 — The presence/absence of barcode gaps. The percentage of species pairs with dintra/dinter ratios <1 were determined for six candidate regions including ITS2, psbA-trnH, matK, rbcL, ycf5, and rpoC1 to be 73.3%, 73.7%, 47.4%, 69.0%, 60.0%, and 35.7%, respectively. Therefore, ITS2 and psbA-trnH have significant barcode gaps. (0.58 MB TIF) [file pone.0008613.s002.tif]

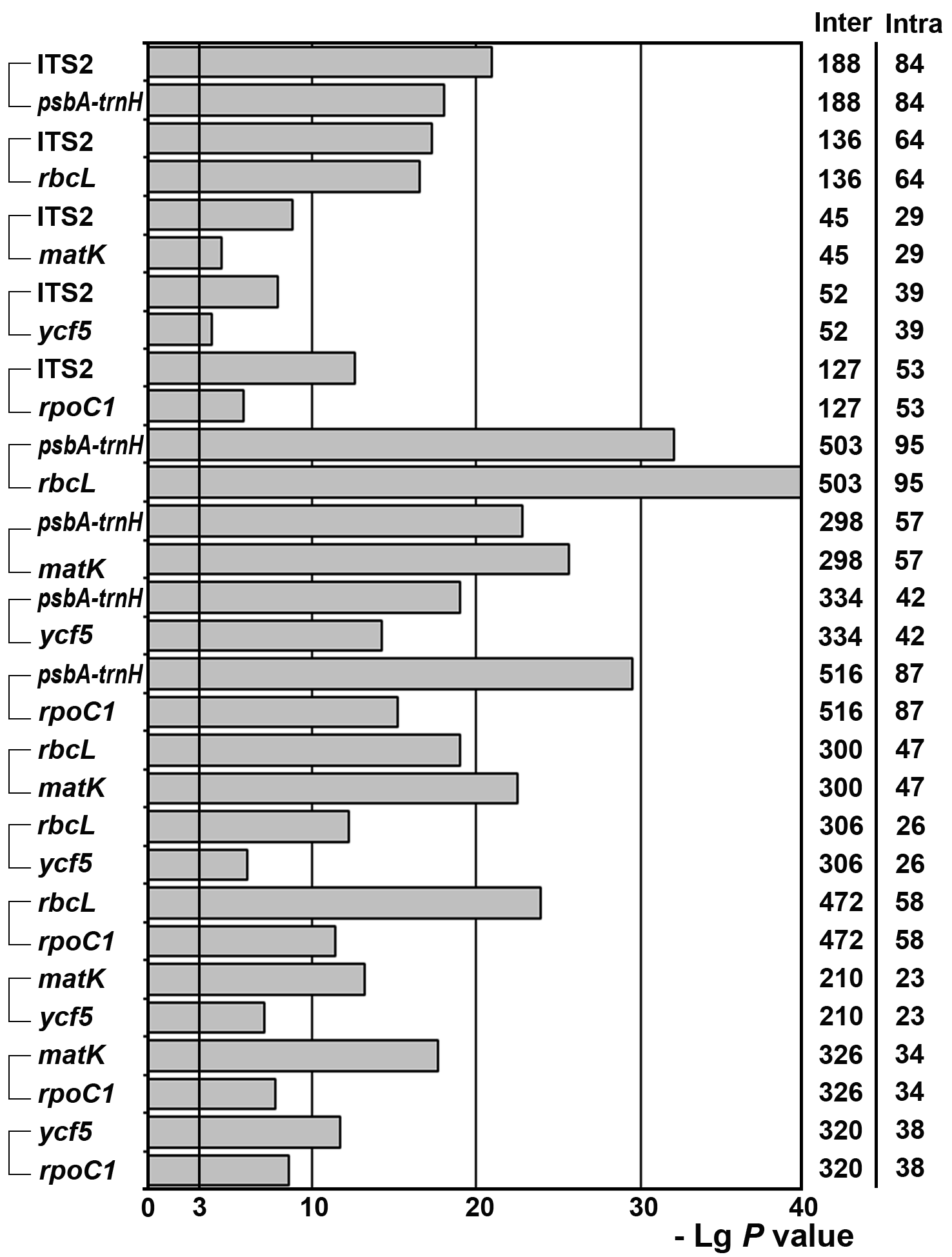

Supplement: Figure S3 — Wilcoxon two-sample tests for the divergences of paired loci with the same set of samples. Inter and Intra mean number of inter-specific distances and number of intra-specific distances, respectively. (0.55 MB TIF) [file pone.0008613.s003.tif]
