# Supplementary material for: Validation of the ITS2 Region as a Novel DNA Barcode for Identifying Medicinal Plant Species
Source: PLoS One. 2010 Jan 7;5(1):e8613. doi: 10.1371/journal.pone.0008613 (PMC2799520; doi:10.1371/journal.pone.0008613)
Supplement: Table S1 — List of universal primers and reaction conditions for candidate barcodes. (0.06 MB DOC) [file pone.0008613.s004.doc]

**Table S1. List of universal primers and reaction conditions for candidate barcodes.**

| **Marker** | **Name of primers** | **Primer sequences 5′-3′** | **PCR reaction conditions** | **Mean size of the marker (size range) in bp** |
| --- | --- | --- | --- | --- |
| **ITS** | 5a fwd | CCTTATCATTTAGAGGAAGGAG | 94℃ 5 min | 707 |
|  | 4 rev | TCCTCCGCTTATTGATATGC | 94℃ 1 min, 50℃ 1 min, 72℃ 1.5 min + 3 sec/cycle, 30 cycles | (571-1153） |
|  |  |  | 72℃ 7 min |  |
| **ITS2** | S2F | ATGCGATACTTGGTGTGAAT | 94℃ 5 min | 226 |
|  | S3R | GACGCTTCTCCAGACTACAAT | 94℃ 30 sec, 56℃ 30 sec, 72℃ 45 sec, 40 cycles | (163-311) |
|  |  |  | 72℃ 10 min |  |
| ***rbcL*** | 1f | ATGTCACCACAAACAGAAAC | 95℃ 2 min | 704 |
|  | 724r | TCGCATGTACCTGCAGTAGC | 94℃ 1 min, 55℃ 30 sec, 72℃ 1 min, 34 cycles | (702-883) |
|  |  |  | 72℃ 7 min |  |
| ***psbA-trnH*** | fwd PA | GTTATGCATGAACGTAATGCTC | 94℃ 5 min | 401 |
|  | rev TH | CGCGCATGGTGGATTCACAATCC | 94℃ 1 min, 55℃ 1 min, 72℃ 1.5 min, 30 cycles | (103-1025) |
|  |  |  | 72℃ 7 min |  |
| ***matK*** | 390F | CGATCTATTCATTCAATATTTC | 94℃ 1 min, 48℃ 30 sec, 72℃ 1 min, 26 cycles | 794 |
|  | 1326R | TCTAGCACACGAAAGTCGAAGT | 72℃ 7 min | (656-861) |
| ***rpoC1*** | 2 forward | GGCAAAGAGGGAAGATTTCG | 94℃ 4 min | 494 |
|  | 4 reverse | CCATAAGCATATCTTGAGTTGG | 94℃ 30 sec, 53℃ 40 sec, 72℃ 40 sec, 40 cycles | (462-556) |
|  |  |  | 72℃ 7 min |  |
| ***ycf5*** | 2 forward | ACTTTAGAGCATATATTAACTC | 94℃ 4 min | 376 |
|  | 3 reverse | ACTTACGTGCATCATTAACCA | 94℃ 30 sec, 53℃ 40 sec, 72℃ 40 sec, 40 cycles | (360-394) |
|  |  |  | 72℃ 7 min |  |
