# Supplementary material for: Validation of the ITS2 Region as a Novel DNA Barcode for Identifying Medicinal Plant Species
Source: PLoS One. 2010 Jan 7;5(1):e8613. doi: 10.1371/journal.pone.0008613 (PMC2799520; doi:10.1371/journal.pone.0008613)
Supplement: Table S2 — Efficiency of PCR amplification of potential barcodes in total number of samples. (0.03 MB DOC) [file pone.0008613.s005.doc]

**Table S2. Efficiency of PCR amplification of potential barcodes in total number of samples.**

| **Marker** | **ITS2** | ***psbA-trnH*** | ***rbcL*** | ***matK*** | ***rpoC1*** | ***ycf5*** |
| --- | --- | --- | --- | --- | --- | --- |
| **Number of samples** | 992 | 992 | 992 | 992 | 720 | 992 |
| **PCR efficiency (%)** | 89.6 | 92.0 | 81.0 | 54.0 | 95.6 | 67.6 |
