# Supplementary material for: Validation of the ITS2 Region as a Novel DNA Barcode for Identifying Medicinal Plant Species
Source: PLoS One. 2010 Jan 7;5(1):e8613. doi: 10.1371/journal.pone.0008613 (PMC2799520; doi:10.1371/journal.pone.0008613)
Supplement: Table S3 — Wilcoxon signed rank tests for inter-specific divergence. (0.05 MB DOC) [file pone.0008613.s006.doc]

**Table S3. Wilcoxon signed rank tests for inter-specific divergence.**

| **W+** | **W-** | **Relative Ranks, *n*, *P* value** | **Result** |
| --- | --- | --- | --- |
| *psbA-trnH* | ITS2 | W+ = 3940, W- = 11285, *n* = 174, *P* ≤ 3.4037 × 10^-8^ | *psbA-trnH* < ITS2 |
| *psbA-trnH* | *matK* | W+ = 34280, W- = 7048, *n* = 287, *P* ≤ 3.8210 × 10^-22^ | *psbA-trnH* >> *matK* |
| *psbA-trnH* | *ycf5* | W+ = 49252, W- = 4049, *n* = 326, *P* ≤ 3.4092 × 10^-40^ | *psbA-trnH* >> *ycf5* |
| *psbA-trnH* | *rbcL* | W+ = 113220, W- = 4635, *n* = 485, *P* ≤ 3.4332 × 10^-69^ | *psbA-trnH* >> *rbcL* |
| *psbA-trnH* | *rpoC1* | W+ = 119037, W- = 768, *n* = 489, *P* ≤ 8.5965 × 10^-80^ | *psbA-trnH* >> *rpoC1* |
| ITS2 | *rpoC1* | W+ = 6364, W- = 77, *n* = 113, *P* ≤ 2.1161 × 10^-19^ | ITS2 >> *rpoC1* |
| ITS2 | *matK* | W+ = 940, W- = 6, *n* = 43, *P* ≤ 1.6158 × 10^-8^ | ITS2 > *matK* |
| ITS2 | *rbcL* | W+ = 7740, W- =10, *n* = 124, *P* ≤ 5.4591 × 10^-22^ | ITS2 >> *rbcL* |
| ITS2 | *ycf5* | W+ = 1225, W- = 50, *n* = 50, *P* ≤ 1.4131 × 10^-8^ | ITS2 > *ycf5* |
| *matK* | *ycf5* | W+ = 18797, W- = 2524, *n* = 206, *P* ≤ 2.1317 × 10^-21^ | *matK* >> *ycf5* |
| *matK* | *rbcL* | W+ = 38505, W- = 3690, *n* = 290, *P* ≤ 4.0211 × 10^-34^ | *matK* >> *rbcL* |
| *matK* | *rpoC1* | W+ = 46572, W- = 1323, *n* = 309, *P* ≤ 5.6408 × 10^-47^ | *matK* >> *rpoC1* |
| *rbcL* | *rpoC1* | W+ = 84546, W- = 11157, *n* = 437, *P* ≤ 7.0458 × 10^-44^ | *rbcL* >> *rpoC1* |
| *rbcL* | *ycf5* | W+ = 28874, W- = 15677, *n* = 298, *P* ≤ 9.3151 × 10^-6^ | *rbcL* > *ycf5* |
| *rpoC1* | *ycf5* | W+ = 6702, W- = 34914, *n* = 288, *P* ≤ 1.8914 × 10^-23^ | *rpoC1* << *ycf5* |
