# Supplementary material for: Validation of the ITS2 Region as a Novel DNA Barcode for Identifying Medicinal Plant Species
Source: PLoS One. 2010 Jan 7;5(1):e8613. doi: 10.1371/journal.pone.0008613 (PMC2799520; doi:10.1371/journal.pone.0008613)
Supplement: Table S4 — Wilcoxon signed rank tests for intra-specific variation. (0.04 MB DOC) [file pone.0008613.s007.doc]

**Table S4. Wilcoxon signed rank tests for intra-specific variation.**

| **W+** | **W-** | **Relative Ranks, *n*, *P* value** | **Result** |
| --- | --- | --- | --- |
| *psbA-trnH* | ITS2 | W+ = 199, W- = 836, *n* = 45, *P* ≤ 3.2392 × 10^-4^ | *psbA-trnH* < ITS2 |
| *psbA-trnH* | *matK* | W+ = 247, W- = 53, *n* = 24, *P* ≤ 0.0055 | *psbA-trnH* > *matK* |
| *psbA-trnH* | *ycf5* | W+ = 108, W- = 63, *n* = 18, *P* ≤ 0.3270 | *psbA-trnH* = *ycf5* |
| *psbA-trnH* | *rbcL* | W+ = 476, W- = 119, *n* = 34, *P* ≤ 0.0023 | *psbA-trnH* > *rbcL* |
| *psbA-trnH* | *rpoC1* | W+ = 417, W- = 79, *n* = 31, *P* ≤ 9.2613 × 10^-4^ | *psbA-trnH* > *rpoC1* |
| ITS2 | *rpoC1* | W+ = 292, W- = 59, *n* = 26, *P* ≤ 0.0031 | ITS2 > *rpoC1* |
| ITS2 | *matK* | W+ = 93, W- = 27, *n* = 15, *P* ≤ 0.0608 | ITS2 > *matK* |
| ITS2 | *rbcL* | W+= 396,W- = 10, *n* = 28, P ≤ 1.0996 × 10^-5^ | ITS2 > *rbcL* |
| ITS2 | *ycf5* | W+ = 275, W- = 76, *n* = 26, *P* ≤ 0.0115 | ITS2 > *ycf5* |
| *matK* | *ycf5* | W+ = 18, W- = 27, *n* = 9, *P* ≤ 0.5940 | *matK* = *ycf5* |
| *matK* | *rbcL* | W+ = 15, W- = 51, *n* = 11, *P* ≤ 0.1086 | *matK* = *rbcL* |
| *matK* | *rpoC1* | W+ = 2, W- = 13, *n* = 5, *P* ≤ 0.1362 | *matK* = *rpoC1* |
| *rbcL* | *rpoC1* | W+ = 30, W- = 61, *n* = 13, *P* ≤ 0.2783 | *rbcL* = *rpoC1* |
| *rbcL* | *ycf5* | W+ = 16, W- = 39, *n* = 10, *P* ≤ 0.2408 | *rbcL* = *ycf5* |
| *rpoC1* | *ycf5* | W+ = 21, W- = 34, *n* = 10, *P* ≤ 0.5046 | *rpoC1* = *ycf5* |
