# Supplementary material for: Validation of the ITS2 Region as a Novel DNA Barcode for Identifying Medicinal Plant Species
Source: PLoS One. 2010 Jan 7;5(1):e8613. doi: 10.1371/journal.pone.0008613 (PMC2799520; doi:10.1371/journal.pone.0008613)
Supplement: Table S5 — Samples for testing potential barcodes and accession numbers in GenBank. (1.23 MB DOC) [file pone.0008613.s008.doc]

**Table S5.** Samples for testing potential barcodes and accession numbers in GenBank.

| **Phylum** | **Family** | **Species** | **Voucher number** | **ITS2 accession number** | ***psbA-trnH* accession number** | ***rbcL* accession number** | ***matK* accession number** | ***rpoC1* accession number** | ***ycf5* accession number** |
| --- | --- | --- | --- | --- | --- | --- | --- | --- | --- |
| Angiosperm | Acanthaceae | *Andrographis paniculata* | PS0732MT01 |  |  | GQ436494 |  |  | GQ435632 |
| Angiosperm | Acanthaceae | *Andrographis paniculata* | PS0732MT02 |  |  |  |  |  | GQ435633 |
| Angiosperm | Acanthaceae | *Andrographis paniculata* | PS0732MT03 |  |  | GQ436495 |  |  |  |
| Angiosperm | Acanthaceae | *Andrographis paniculata* | PS0732MT04 |  | GQ435145 |  |  |  |  |
| Angiosperm | Acanthaceae | *Andrographis paniculata* | PS0732MT05 |  | GQ435146 | GQ436496 |  |  |  |
| Angiosperm | Acanthaceae | *Baphicacanthus cusia* | PS0742MT01 |  |  |  | GQ434122 |  | GQ435635 |
| Angiosperm | Acanthaceae | *Baphicacanthus cusia* | PS0742MT02 |  | GQ435147 |  | GQ434123 |  |  |
| Angiosperm | Acanthaceae | *Baphicacanthus cusia* | PS0742MT03 | GQ434546 | GQ435148 |  |  |  |  |
| Angiosperm | Acanthaceae | *Baphicacanthus cusia* | PS0742MT07 |  | GQ435149 | GQ436498 | GQ434124 |  | GQ435636 |
| Angiosperm | Acanthaceae | *Baphicacanthus cusia* | PS0742MT09 |  |  | GQ436499 |  | GQ436044 |  |
| Angiosperm | Acanthaceae | *Clinacanthus nutans* | PS0749MT01 | GQ434547 | GQ435151 | GQ436501 |  |  |  |
| Angiosperm | Acanthaceae | *Gendarussa ventricosa* | PS0733MT03 |  |  | GQ436497 |  |  | GQ435634 |
| Angiosperm | Acanthaceae | *Gendarussa vulgaris* | PS0748MT01 |  |  | GQ436500 |  |  | GQ435637 |
| Angiosperm | Acanthaceae | *Rhinacanthus nasutus* | PS0730MT01 |  | GQ435144 | GQ436493 |  |  |  |
| Angiosperm | Alismataceae | *Alisma plantago-aquatica* | PS1621MT01 |  | GQ435456 |  |  |  |  |
| Angiosperm | Amaranthaceae | *Achyranthes aspera* | PS1496MT01 |  | GQ435413 |  | GQ434275 |  | GQ435799 |
| Angiosperm | Amaranthaceae | *Achyranthes bidentata* | PS1493MT05 | GQ434786 | GQ435410 | GQ436715 |  | GQ436229 |  |
| Angiosperm | Amaranthaceae | *Celosia argentea* | PS1495MT01 | GQ434787 | GQ435411 | GQ436716 | GQ434273 |  | GQ435798 |
| Angiosperm | Amaranthaceae | *Celosia argentea* | PS1495MT02 | GQ434788 | GQ435412 |  | GQ434274 | GQ436230 |  |
| Angiosperm | Amaranthaceae | *Celosia cristata* | PS1491MT01 | GQ434784 | GQ435409 |  | GQ434271 | GQ436227 | GQ435797 |
| Angiosperm | Amaranthaceae | *Celosia cristata* | PS1491MT02 | GQ434785 |  |  | GQ434272 | GQ436228 |  |
| Angiosperm | Amaryllidaceae | *Curculigo orchioides* | PS1485MT01 |  | GQ435408 |  |  |  | GQ435796 |
| Angiosperm | Amaryllidaceae | *Narcissus tazetta* | PS1302MT01 |  | GQ435346 | GQ436660 | GQ434246 |  | GQ435760 |
| Angiosperm | Anacardiaceae | *Choerospondias axillaris* | PS1010MT01 | GQ434625 |  |  |  |  | GQ435690 |
| Angiosperm | Anacardiaceae | *Mangifera indica* | PS1008MT01 |  | GQ435225 | GQ436547 |  |  |  |
| Angiosperm | Anacardiaceae | *Rhus chinensis* | PS1014MT01 | GQ434626 |  | GQ436548 |  |  | GQ435691 |
| Angiosperm | Anacardiaceae | *Rhus chinensis* | PS1014MT03 | GQ434627 |  |  |  | GQ436090 |  |
| Angiosperm | Apiaceae | *Angelica dahurica* | PS1197MT01 | GQ434688 | GQ435303 | GQ436628 |  | GQ436165 | GQ435736 |
| Angiosperm | Apiaceae | *Angelica dahurica* | PS1197MT03 | GQ434689 | GQ435304 | GQ436629 |  | GQ436166 | GQ435737 |
| Angiosperm | Apiaceae | *Angelica dahurica cv. hangbaizhi* var. *formosan* | PS1210MT04 | GQ434696 | GQ435308 | GQ436634 |  |  |  |
| Angiosperm | Apiaceae | *Angelica decursiva* | PS1226MT04 | GQ434705 | GQ435318 | GQ436638 |  | GQ436176 | GQ435747 |
| Angiosperm | Apiaceae | *Angelica decursiva* | PS1226MT05 | GQ434706 | GQ435319 | GQ436639 |  |  |  |
| Angiosperm | Apiaceae | *Angelica sinensis* | PS1205MT01 | GQ434694 |  | GQ436632 | GQ434227 | GQ436167 |  |
| Angiosperm | Apiaceae | *Bupleurum chinense* | PS1198MT02 | GQ434690 |  |  |  |  |  |
| Angiosperm | Apiaceae | *Centella asiatica* | PS1212MT01 | GQ434697 | GQ435309 | GQ436635 | GQ434229 | GQ436168 |  |
| Angiosperm | Apiaceae | *Centella asiatica* | PS1212MT04 | GQ434698 | GQ435310 | GQ436636 | GQ434230 |  | GQ435740 |
| Angiosperm | Apiaceae | *Centella asiatica* | PS1212MT05 |  | GQ435311 |  | GQ434231 |  | GQ435741 |
| Angiosperm | Apiaceae | *Chuanminshen violaceum* | PS1202MT01 | GQ434691 |  |  |  |  | GQ435738 |
| Angiosperm | Apiaceae | *Cryptotaenia japonica* | PS1223MT01 | GQ434702 | GQ435315 |  |  | GQ436173 | GQ435745 |
| Angiosperm | Apiaceae | *Daucus carota* | PS1225MT01 | GQ434703 | GQ435316 |  |  | GQ436174 |  |
| Angiosperm | Apiaceae | *Daucus carota* | PS1225MT02 | GQ434704 | GQ435317 |  | GQ434234 | GQ436175 | GQ435746 |
| Angiosperm | Apiaceae | *Foeniculum vulgare* | PS1222MT01 |  |  |  |  | GQ436171 |  |
| Angiosperm | Apiaceae | *Foeniculum vulgare* | PS1222MT02 | GQ434700 | GQ435313 |  |  |  |  |
| Angiosperm | Apiaceae | *Foeniculum vulgare* | PS1222MT03 | GQ434701 | GQ435314 |  | GQ434233 | GQ436172 | GQ435744 |
| Angiosperm | Apiaceae | *Ligusticum chuanxiong* | PS1203MT01 | GQ434692 | GQ435305 | GQ436630 |  |  | GQ435739 |
| Angiosperm | Apiaceae | *Ligusticum chuanxiong* | PS1203MT02 | GQ434693 | GQ435306 | GQ436631 |  |  |  |
| Angiosperm | Apiaceae | *Ligusticum jeholense* | PS1213MT02 | GQ434699 |  |  |  | GQ436169 | GQ435742 |
| Angiosperm | Apiaceae | *Ligusticum sinense* | PS1208MT01 | GQ434695 |  |  |  |  |  |
| Angiosperm | Apiaceae | *Notopterygium franchetii* | PS1215MT01 |  | GQ435312 | GQ436637 | GQ434232 | GQ436170 | GQ435743 |
| Angiosperm | Apiaceae | *Saposhnikovia divaricata* | PS1207MT02 |  | GQ435307 | GQ436633 | GQ434228 |  |  |
| Angiosperm | Apocynaceae | *Alstonia scholaris* | PS0506MT02 | GQ434436 | GQ435037 | GQ436401 |  | GQ435993 | GQ435583 |
| Angiosperm | Apocynaceae | *Alstonia scholaris* | PS0506MT03 | GQ434437 | GQ435038 | GQ436402 | GQ434101 |  |  |
| Angiosperm | Apocynaceae | *Rauvolfia verticillata* | PS0500MT01 | GQ434433 | GQ435034 | GQ436398 | GQ434097 |  | GQ435580 |
| Angiosperm | Apocynaceae | *Trachelospermum jasminoides* | PS0501MT01 | GQ434434 |  |  | GQ434098 | GQ435992 | GQ435581 |
| Angiosperm | Apocynaceae | *Trachelospermum jasminoides* | PS0501MT02 |  | GQ435035 | GQ436399 | GQ434099 |  |  |
| Angiosperm | Apocynaceae | *Trachelospermum jasminoides* | PS0501MT06 | GQ434435 | GQ435036 | GQ436400 | GQ434100 |  | GQ435582 |
| Angiosperm | Aquifoliaceae | *Ilex asprella* | PS0325MT01 | GQ434387 |  | GQ436367 |  |  | GQ435549 |
| Angiosperm | Aquifoliaceae | *Ilex cornuta* | PS0320MT01 | GQ434382 | GQ434981 | GQ436364 |  |  |  |
| Angiosperm | Aquifoliaceae | *Ilex cornuta* | PS0320MT03 | GQ434383 |  |  |  |  |  |
| Angiosperm | Aquifoliaceae | *Ilex cornuta* | PS0320MT05 | GQ434384 | GQ434982 | GQ436365 |  |  |  |
| Angiosperm | Aquifoliaceae | *Ilex pubilimba* | PS0324MT01 | GQ434386 | GQ434983 | GQ436366 |  |  |  |
| Angiosperm | Aquifoliaceae | *Ilex rotunda* | PS0321MT01 | GQ434385 |  |  |  |  | GQ435548 |
| Angiosperm | Araceae | *Acorus calamus* | PS1418MT03 |  | GQ435387 | GQ436696 | GQ434259 | GQ436214 | GQ435779 |
| Angiosperm | Araceae | *Acorus calamus* | PS1418MT04 | GQ434763 |  |  |  |  | GQ435780 |
| Angiosperm | Araceae | *Acorus tatarinowii* | PS1301MT04 |  |  |  | GQ434241 |  | GQ435759 |
| Angiosperm | Araceae | *Acorus tatarinowii* | PS1301MT05 | GQ434723 | GQ435343 |  | GQ434242 |  |  |
| Angiosperm | Araceae | *Acorus tatarinowii* | PS1301MT06 |  | GQ435344 |  |  |  |  |
| Angiosperm | Araceae | *Acorus tatarinowii* | PS1301MT07 | GQ434724 |  |  |  |  |  |
| Angiosperm | Araceae | *Acorus tatarinowii* | PS1301MT12 | GQ434725 |  | GQ436657 | GQ434243 |  |  |
| Angiosperm | Araceae | *Acorus tatarinowii* | PS1301MT13 | GQ434726 |  | GQ436658 | GQ434244 |  |  |
| Angiosperm | Araceae | *Acorus tatarinowii* | PS1301MT14 | GQ434727 | GQ435345 |  | GQ434245 |  |  |
| Angiosperm | Araceae | *Acorus tatarinowii* | PS1301MT15 | GQ434728 |  | GQ436659 |  |  |  |
| Angiosperm | Araceae | *Alocasia macrorrhiza* | PS1408MT01 |  |  |  |  | GQ436211 |  |
| Angiosperm | Araceae | *Arisaema amurense* var. *serratum* | PS3009MT01 | GQ434867 |  | GQ436774 | GQ434305 | GQ436277 |  |
| Angiosperm | Araceae | *Arisaema erubescens* | PS3013MT04 |  |  | GQ436779 | GQ434310 |  |  |
| Angiosperm | Araceae | *Arisaema erubescens* | PS3013MT05 |  |  |  |  | GQ436281 |  |
| Angiosperm | Araceae | *Arisaema heterophyllum* | PS3010MT01 | GQ434868 |  | GQ436775 | GQ434306 | GQ436278 |  |
| Angiosperm | Araceae | *Arisaema sikokianum* var. *serratum* | PS3012MT01 | GQ434869 |  | GQ436776 | GQ434307 | GQ436279 |  |
| Angiosperm | Araceae | *Arisaema sikokianum* var. *serratum* | PS3012MT02 | GQ434870 |  | GQ436777 | GQ434308 |  |  |
| Angiosperm | Araceae | *Arisaema sikokianum* var. *serratum* | PS3012MT03 | GQ434871 |  | GQ436778 | GQ434309 | GQ436280 |  |
| Angiosperm | Araceae | *Epipremnum aureum* | PS1412MT03 |  |  | GQ436692 | GQ434255 | GQ436212 |  |
| Angiosperm | Araceae | *Homalomena occulta* | PS1416MT01 |  |  |  | GQ434256 |  |  |
| Angiosperm | Araceae | *Homalomena occulta* | PS1416MT05 |  | GQ435386 | GQ436694 | GQ434257 |  |  |
| Angiosperm | Araceae | *Homalomena occulta* | PS1416MT06 |  |  | GQ436695 | GQ434258 |  |  |
| Angiosperm | Araceae | *Lasia spinosa* | PS1402MT03 |  |  | GQ436687 | GQ434251 |  |  |
| Angiosperm | Araceae | *Lasia spinosa* | PS1402MT04 |  |  | GQ436688 | GQ434252 |  |  |
| Angiosperm | Araceae | *Monstera deliciosa* | PS3002MT01 |  |  | GQ436772 | GQ434304 | GQ436275 |  |
| Angiosperm | Araceae | *Pinellia pedatisecta* | PS1409MT01 |  |  | GQ436691 |  |  |  |
| Angiosperm | Araceae | *Pinellia pedatisecta* | PS1409MT06 |  | GQ435384 |  |  |  |  |
| Angiosperm | Araceae | *Pinellia pedatisecta* | PS1409MT07 |  |  |  | GQ434254 |  | GQ435777 |
| Angiosperm | Araceae | *Pinellia ternata* | PS1399MT04 |  |  | GQ436686 |  |  |  |
| Angiosperm | Araceae | *Pistia stratiotes* | PS1403MT01 |  |  | GQ436689 |  |  |  |
| Angiosperm | Araceae | *Syngonium podophyllum* | PS3007MT01 | GQ434866 |  | GQ436773 |  | GQ436276 |  |
| Angiosperm | Araceae | *Typhonium giganteum* | PS1405MT01 |  | GQ435382 | GQ436690 | GQ434253 | GQ436209 | GQ435776 |
| Angiosperm | Araceae | *Typhonium giganteum* | PS1405MT02 |  | GQ435383 |  |  | GQ436210 |  |
| Angiosperm | Araceae | *Zantedeschia aethiopica* | PS1413MT01 |  | GQ435385 | GQ436693 |  | GQ436213 | GQ435778 |
| Angiosperm | Araliaceae | *Acanthopanax giraldii* | PS1461MT01 | GQ434776 | GQ435397 |  | GQ434263 |  | GQ435788 |
| Angiosperm | Araliaceae | *Acanthopanax gracilistylus* | PS1473MT01 | GQ434779 | GQ435404 | GQ436710 |  | GQ436223 | GQ435791 |
| Angiosperm | Araliaceae | *Acanthopanax senticosus* | PS1456MT03 | GQ434775 | GQ435396 | GQ436704 | GQ434262 |  |  |
| Angiosperm | Araliaceae | *Eleutherococcus sessiliflorus* | PS1471MT01 | GQ434778 |  |  | GQ434268 | GQ436221 | GQ435790 |
| Angiosperm | Araliaceae | *Hedera nepalensis* var. *sinensis* | PS1454MT02 | GQ434773 | GQ435394 |  | GQ434260 | GQ436216 | GQ435786 |
| Angiosperm | Araliaceae | *Panax ginseng* | PS1467MT01 |  | GQ435398 | GQ436705 | GQ434264 | GQ436217 |  |
| Angiosperm | Araliaceae | *Panax japonicus* | PS1477MT01 | GQ434780 |  |  |  | GQ436224 | GQ435792 |
| Angiosperm | Araliaceae | *Panax japonicus* | PS1477MT02 | GQ434781 | GQ435405 | GQ436711 |  | GQ436225 | GQ435793 |
| Angiosperm | Araliaceae | *Panax japonicus* | PS1477MT03 | GQ434782 | GQ435406 | GQ436712 |  | GQ436226 | GQ435794 |
| Angiosperm | Araliaceae | *Panax japonicus* | PS1477MT04 | GQ434783 | GQ435407 | GQ436713 | GQ434270 |  | GQ435795 |
| Angiosperm | Araliaceae | *Panax notoginseng* | PS1469MT01 |  | GQ435399 |  | GQ434265 | GQ436218 |  |
| Angiosperm | Araliaceae | *Panax notoginseng* | PS1469MT02 |  | GQ435400 | GQ436706 | GQ434266 | GQ436219 |  |
| Angiosperm | Araliaceae | *Panax notoginseng* | PS1469MT03 |  | GQ435401 | GQ436707 |  |  |  |
| Angiosperm | Araliaceae | *Panax quinquefolius* | PS1472MT01 |  | GQ435403 | GQ436709 | GQ434269 | GQ436222 |  |
| Angiosperm | Araliaceae | *Tetrapanax papyrifer* | PS1470MT02 | GQ434777 | GQ435402 | GQ436708 | GQ434267 | GQ436220 | GQ435789 |
| Angiosperm | Araliaceae | *Trevesia palmata* | PS1455MT01 | GQ434774 | GQ435395 | GQ436703 | GQ434261 |  | GQ435787 |
| Angiosperm | Aristolochiaceae | *Aristolochia austroszechuanica* | PS0878MT01 | GQ434591 | GQ435193 |  | GQ434151 | GQ436062 | GQ435659 |
| Angiosperm | Aristolochiaceae | *Aristolochia contorta* | PS0877MT02 |  | GQ435192 |  |  |  | GQ435658 |
| Angiosperm | Aristolochiaceae | *Aristolochia debilis* | PS0888MT01 |  | GQ435194 |  | GQ434153 | GQ436065 | GQ435661 |
| Angiosperm | Aristolochiaceae | *Asarum forbesii* | PS0880MT02 | GQ434592 |  |  | GQ434152 | GQ436063 | GQ435660 |
| Angiosperm | Aristolochiaceae | *Asarum maximum* | PS0885MT01 | GQ434593 |  |  |  | GQ436064 |  |
| Angiosperm | Asclepiadaceae | *Cynanchum atratum* | PS0833MT01 | GQ434569 | GQ435173 | GQ436511 |  | GQ436051 |  |
| Angiosperm | Asclepiadaceae | *Cynanchum mongolicum* | PS0834MT01 | GQ434570 | GQ435174 | GQ436512 |  | GQ436052 |  |
| Angiosperm | Asclepiadaceae | *Cynanchum paniculatum* | PS0836MT01 | GQ434572 | GQ435175 |  |  | GQ436053 |  |
| Angiosperm | Asclepiadaceae | *Cynanchum stauntonii* | PS0835MT04 | GQ434571 |  |  |  |  |  |
| Angiosperm | Asclepiadaceae | *Dregea volubilis* | PS0842MT01 | GQ434575 | GQ435176 | GQ436514 |  |  |  |
| Angiosperm | Asclepiadaceae | *Marsdenia tenacissima* | PS0844MT01 | GQ434576 | GQ435177 | GQ436515 |  |  |  |
| Angiosperm | Asclepiadaceae | *Periploca sepium* | PS0840MT01 | GQ434573 |  |  |  | GQ436054 |  |
| Angiosperm | Asclepiadaceae | *Periploca sepium* | PS0840MT03 | GQ434574 |  | GQ436513 |  | GQ436055 |  |
| Angiosperm | Asteraceae | *Achillea wilsoniana* | PS0715MT01 | GQ434539 | GQ435138 | GQ436490 |  | GQ436041 |  |
| Angiosperm | Asteraceae | *Achillea wilsoniana* | PS0715MT02 | GQ434540 | GQ435139 | GQ436491 |  | GQ436042 |  |
| Angiosperm | Asteraceae | *Ageratum conyzoides* | PS0636MT01 | GQ434496 | GQ435093 | GQ436453 |  | GQ436014 |  |
| Angiosperm | Asteraceae | *Arctium lappa* | PS0668MT01 |  | GQ435104 |  |  |  |  |
| Angiosperm | Asteraceae | *Arctium lappa* | PS0668MT02 |  | GQ435105 | GQ436459 | GQ434116 | GQ436017 |  |
| Angiosperm | Asteraceae | *Arctium lappa* | PS0668MT09 | GQ434509 |  | GQ436460 |  |  | GQ435623 |
| Angiosperm | Asteraceae | *Arctium lappa* | PS0668MT10 | GQ434510 |  |  |  |  |  |
| Angiosperm | Asteraceae | *Artemisia annua* | PS0633MT04 | GQ434492 | GQ435090 |  |  |  | GQ435615 |
| Angiosperm | Asteraceae | *Artemisia annua* | PS0633MT05 | GQ434493 |  |  |  | GQ436012 |  |
| Angiosperm | Asteraceae | *Artemisia annua* | PS0633MT08 | GQ434494 | GQ435091 |  |  |  |  |
| Angiosperm | Asteraceae | *Artemisia anomala* | PS0587MT01 | GQ434468 |  |  |  |  | GQ435599 |
| Angiosperm | Asteraceae | *Artemisia argyi* | PS0590MT04 | GQ434469 | GQ435067 | GQ436428 |  | GQ435994 |  |
| Angiosperm | Asteraceae | *Artemisia argyi* | PS0590MT05 | GQ434470 |  | GQ436429 |  |  | GQ435600 |
| Angiosperm | Asteraceae | *Artemisia capillaris* | PS0712MT02 | GQ434538 |  |  |  |  |  |
| Angiosperm | Asteraceae | *Artemisia gmelinii* | PS0594MT01 | GQ434472 | GQ435070 | GQ436432 | GQ434109 | GQ435997 | GQ435603 |
| Angiosperm | Asteraceae | *Artemisia lavandulaefolia* | PS0703MT01 | GQ434533 | GQ435131 | GQ436484 |  | GQ436036 |  |
| Angiosperm | Asteraceae | *Aster ageratoides* | PS0680MT01 | GQ434522 | GQ435121 | GQ436475 |  | GQ436030 |  |
| Angiosperm | Asteraceae | *Aster tataricus* | PS0721MT03 | GQ434542 |  |  |  |  | GQ435631 |
| Angiosperm | Asteraceae | *Aster tataricus* | PS0721MT04 | GQ434543 | GQ435142 | GQ436492 |  | GQ436043 |  |
| Angiosperm | Asteraceae | *Atractylodes lancea* | PS0596MT03 |  | GQ435071 | GQ436433 |  |  | GQ435604 |
| Angiosperm | Asteraceae | *Bidens pilosa* var. *radiata* | PS0593MT01 | GQ434471 | GQ435068 | GQ436430 | GQ434107 | GQ435995 | GQ435601 |
| Angiosperm | Asteraceae | *Bidens pilosa* var. *radiata* | PS0593MT02 |  | GQ435069 | GQ436431 | GQ434108 | GQ435996 | GQ435602 |
| Angiosperm | Asteraceae | *Carthamus tinctorius* | PS0629MT01 |  |  |  | GQ434115 |  | GQ435614 |
| Angiosperm | Asteraceae | *Carthamus tinctorius* | PS0629MT02 |  | GQ435089 | GQ436451 |  | GQ436011 |  |
| Angiosperm | Asteraceae | *Centipeda minima* | PS0620MT01 | GQ434488 | GQ435086 | GQ436448 |  | GQ436008 |  |
| Angiosperm | Asteraceae | *Centipeda minima* | PS0620MT03 | GQ434489 | GQ435087 | GQ436449 |  | GQ436009 |  |
| Angiosperm | Asteraceae | *Centipeda minima* | PS0620MT04 | GQ434490 |  |  | GQ434113 |  | GQ435613 |
| Angiosperm | Asteraceae | *Chrysanthemum indicum* | PS0705MT01 |  | GQ435133 |  |  |  |  |
| Angiosperm | Asteraceae | *Cichorium intybus* | PS0644MT02 | GQ434500 |  |  |  |  | GQ435617 |
| Angiosperm | Asteraceae | *Cirsium japonicum* | PS0612MT02 | GQ434478 | GQ435079 | GQ436441 |  |  |  |
| Angiosperm | Asteraceae | *Cirsium japonicum* | PS0612MT03 | GQ434479 |  | GQ436442 |  | GQ436004 |  |
| Angiosperm | Asteraceae | *Cirsium japonicum* | PS0612MT06 | GQ434480 | GQ435080 |  | GQ434111 |  | GQ435611 |
| Angiosperm | Asteraceae | *Cirsium japonicum* | PS0612MT07 |  | GQ435081 | GQ436443 |  |  |  |
| Angiosperm | Asteraceae | *Cirsium setosum* | PS0611MT01 |  | GQ435076 | GQ436438 |  |  |  |
| Angiosperm | Asteraceae | *Cirsium setosum* | PS0611MT02 |  | GQ435077 | GQ436439 |  | GQ436002 | GQ435609 |
| Angiosperm | Asteraceae | *Cirsium setosum* | PS0611MT04 | GQ434477 | GQ435078 | GQ436440 |  | GQ436003 | GQ435610 |
| Angiosperm | Asteraceae | *Cosmos bipinnata* | PS0678MT01 | GQ434521 | GQ435119 | GQ436473 |  | GQ436028 |  |
| Angiosperm | Asteraceae | *Cosmos bipinnata* | PS0678MT02 |  | GQ435120 | GQ436474 |  | GQ436029 |  |
| Angiosperm | Asteraceae | *Dendranthema lavandulifolium* | PS0704MT01 | GQ434534 | GQ435132 | GQ436485 | GQ434120 | GQ436037 |  |
| Angiosperm | Asteraceae | *Dendranthema morifolium* | PS0643MT01 | GQ434499 | GQ435094 |  |  |  |  |
| Angiosperm | Asteraceae | *Dichrocephala benthamii* | PS0697MT01 | GQ434529 | GQ435127 | GQ436480 |  | GQ436034 |  |
| Angiosperm | Asteraceae | *Eclipta prostrata* | PS0651MT04 | GQ434504 | GQ435098 | GQ436455 |  | GQ436015 |  |
| Angiosperm | Asteraceae | *Eclipta prostrata* | PS0651MT05 |  | GQ435099 | GQ436456 |  |  | GQ435619 |
| Angiosperm | Asteraceae | *Eclipta prostrata* | PS0651MT06 | GQ434505 | GQ435100 |  |  |  | GQ435620 |
| Angiosperm | Asteraceae | *Elephantopus scaber* | PS0617MT01 | GQ434482 | GQ435083 | GQ436445 |  |  |  |
| Angiosperm | Asteraceae | *Emilia sonchifolia* | PS0709MT01 | GQ434535 | GQ435134 | GQ436486 |  |  |  |
| Angiosperm | Asteraceae | *Emilia sonchifolia* | PS0709MT02 | GQ434536 | GQ435135 | GQ436487 |  | GQ436038 |  |
| Angiosperm | Asteraceae | *Emilia sonchifolia* | PS0709MT03 | GQ434537 | GQ435136 | GQ436488 |  | GQ436039 |  |
| Angiosperm | Asteraceae | *Eupatorium fortunei* | PS0672MT01 | GQ434513 | GQ435109 | GQ436464 |  |  | GQ435624 |
| Angiosperm | Asteraceae | *Eupatorium fortunei* | PS0672MT02 |  | GQ435110 |  |  | GQ436021 |  |
| Angiosperm | Asteraceae | *Eupatorium fortunei* | PS0672MT05 |  | GQ435111 | GQ436465 |  | GQ436022 |  |
| Angiosperm | Asteraceae | *Gaillardia pulchella* | PS0687MT01 | GQ434525 | GQ435124 | GQ436478 |  | GQ436032 |  |
| Angiosperm | Asteraceae | *Gaillardia pulchella* | PS0687MT02 | GQ434526 | GQ435125 | GQ436479 |  | GQ436033 |  |
| Angiosperm | Asteraceae | *Galinsoga parviflora* | PS0649MT01 | GQ434503 | GQ435097 |  |  |  |  |
| Angiosperm | Asteraceae | *Gerbera anandria* | PS0610MT01 |  | GQ435075 | GQ436437 | GQ434110 | GQ436001 | GQ435608 |
| Angiosperm | Asteraceae | *Gnaphalium affine* | PS0684MT01 | GQ434523 | GQ435122 | GQ436476 |  |  |  |
| Angiosperm | Asteraceae | *Gynura bicolor* | PS0628MT01 | GQ434491 | GQ435088 | GQ436450 | GQ434114 | GQ436010 |  |
| Angiosperm | Asteraceae | *Gynura procumbens* | PS0673MT01 | GQ434514 | GQ435112 | GQ436466 |  | GQ436023 |  |
| Angiosperm | Asteraceae | *Inula britanica* | PS0670MT01 | GQ434511 | GQ435106 | GQ436461 | GQ434117 | GQ436018 |  |
| Angiosperm | Asteraceae | *Inula britanica* | PS0670MT02 |  | GQ435107 | GQ436462 |  | GQ436019 |  |
| Angiosperm | Asteraceae | *Inula cappa* | PS0701MT01 | GQ434532 | GQ435129 |  |  |  |  |
| Angiosperm | Asteraceae | *Inula cappa* | PS0701MT02 |  | GQ435130 | GQ436483 |  |  |  |
| Angiosperm | Asteraceae | *Inula helenium* | PS0689MT01 | GQ434528 |  |  |  |  |  |
| Angiosperm | Asteraceae | *Laggera alata* | PS0653MT01 | GQ434506 | GQ435101 | GQ436457 |  | GQ436016 |  |
| Angiosperm | Asteraceae | *Leontopodium leontopodioides* | PS0634MT01 | GQ434495 | GQ435092 | GQ436452 |  | GQ436013 |  |
| Angiosperm | Asteraceae | *Ligularia hodgsonii* | PS0655MT01 | GQ434507 | GQ435102 | GQ436458 |  |  | GQ435621 |
| Angiosperm | Asteraceae | *Petasites japonicus* | PS0648MT01 | GQ434501 | GQ435095 |  |  |  | GQ435618 |
| Angiosperm | Asteraceae | *Petasites japonicus* | PS0648MT02 | GQ434502 | GQ435096 | GQ436454 |  |  |  |
| Angiosperm | Asteraceae | *Prenanthes macrophylla* | PS0615MT01 | GQ434481 | GQ435082 | GQ436444 | GQ434112 | GQ436005 | GQ435612 |
| Angiosperm | Asteraceae | *Prenanthes tatarinowii* | PS0671MT01 | GQ434512 | GQ435108 | GQ436463 |  | GQ436020 |  |
| Angiosperm | Asteraceae | *Rhaponticum uniflorum* | PS0674MT01 | GQ434515 | GQ435113 | GQ436467 |  | GQ436024 |  |
| Angiosperm | Asteraceae | *Saussurea involucrata* | PS0699MT01 | GQ434530 | GQ435128 | GQ436481 |  |  |  |
| Angiosperm | Asteraceae | *Saussurea nivea* | PS0713MT01 |  | GQ435137 | GQ436489 | GQ434121 | GQ436040 | GQ435630 |
| Angiosperm | Asteraceae | *Scorzonera austriaca* | PS0700MT01 | GQ434531 |  | GQ436482 | GQ434119 | GQ436035 |  |
| Angiosperm | Asteraceae | *Senecio scandens* | PS0676MT01 | GQ434516 |  |  |  |  | GQ435625 |
| Angiosperm | Asteraceae | *Senecio scandens* | PS0676MT02 | GQ434517 | GQ435114 | GQ436468 |  | GQ436025 |  |
| Angiosperm | Asteraceae | *Senecio scandens* | PS0676MT03 | GQ434518 | GQ435115 | GQ436469 |  | GQ436026 |  |
| Angiosperm | Asteraceae | *Senecio scandens* | PS0676MT05 | GQ434519 | GQ435116 | GQ436470 |  | GQ436027 |  |
| Angiosperm | Asteraceae | *Senecio scandens* | PS0676MT06 | GQ434520 | GQ435117 | GQ436471 |  |  | GQ435626 |
| Angiosperm | Asteraceae | *Senecio scandens* | PS0676MT07 |  | GQ435118 | GQ436472 |  |  | GQ435627 |
| Angiosperm | Asteraceae | *Siegesbeckia glabrescens* | PS0660MT01 |  | GQ435103 |  |  |  | GQ435622 |
| Angiosperm | Asteraceae | *Siegesbeckia orientalis* | PS0618MT01 | GQ434483 |  |  |  |  |  |
| Angiosperm | Asteraceae | *Siegesbeckia orientalis* | PS0618MT02 | GQ434484 |  |  |  |  |  |
| Angiosperm | Asteraceae | *Siegesbeckia orientalis* | PS0618MT03 | GQ434485 |  |  |  |  |  |
| Angiosperm | Asteraceae | *Siegesbeckia orientalis* | PS0618MT04 | GQ434486 | GQ435084 | GQ436446 |  | GQ436006 |  |
| Angiosperm | Asteraceae | *Siegesbeckia orientalis* | PS0618MT05 | GQ434487 | GQ435085 | GQ436447 |  | GQ436007 |  |
| Angiosperm | Asteraceae | *Siegesbeckia pubescens* | PS0661MT02 | GQ434508 |  |  |  |  |  |
| Angiosperm | Asteraceae | *Solidago decurrens* | PS0638MT01 | GQ434497 |  |  |  |  | GQ435616 |
| Angiosperm | Asteraceae | *Solidago decurrens* | PS0638MT02 | GQ434498 |  |  |  |  |  |
| Angiosperm | Asteraceae | *Stevia rebaudiana* | PS0688MT01 | GQ434527 | GQ435126 |  |  |  | GQ435629 |
| Angiosperm | Asteraceae | *Taraxacum platypecidum* var. *platypecidum* | PS0598MT01 | GQ434473 | GQ435072 | GQ436434 |  | GQ435998 | GQ435605 |
| Angiosperm | Asteraceae | *Tithonia rotundifolia* | PS0718MT01 | GQ434541 | GQ435140 |  |  |  |  |
| Angiosperm | Asteraceae | *Tragopogon porrifolius* | PS0686MT01 | GQ434524 | GQ435123 | GQ436477 | GQ434118 | GQ436031 | GQ435628 |
| Angiosperm | Asteraceae | *Xanthium sibiricum* | PS0604MT01 | GQ434474 | GQ435073 | GQ436435 |  | GQ435999 | GQ435606 |
| Angiosperm | Asteraceae | *Xanthium sibiricum* | PS0604MT02 | GQ434475 | GQ435074 |  |  | GQ436000 |  |
| Angiosperm | Asteraceae | *Xanthium sibiricum* | PS0604MT03 |  |  |  |  |  | GQ435607 |
| Angiosperm | Asteraceae | *Xanthium sibiricum* | PS0604MT04 | GQ434476 |  | GQ436436 |  |  |  |
| Angiosperm | Balsaminaceae | *Impatiens balsamina* | PS0365MT04 | GQ434400 | GQ434995 | GQ436373 |  | GQ435978 |  |
| Angiosperm | Basellaceae | *Anredera cordifolia* | PS0846MT01 | GQ434577 |  | GQ436516 | GQ434142 |  | GQ435649 |
| Angiosperm | Berberidaceae | *Dysosma veitchii* | PS0397MT01 | GQ434407 | GQ435004 |  |  | GQ435981 |  |
| Angiosperm | Berberidaceae | *Epimedium acuminatum* | PS1499MT01 | GQ434789 | GQ435414 |  |  | GQ436231 | GQ435800 |
| Angiosperm | Berberidaceae | *Epimedium acuminatum* | PS1499MT02 | GQ434790 | GQ435415 |  | GQ434276 |  | GQ435801 |
| Angiosperm | Berberidaceae | *Epimedium pubescens* | PS1505MT01 | GQ434793 |  | GQ436718 |  | GQ436234 | GQ435804 |
| Angiosperm | Berberidaceae | *Epimedium sagittatum* | PS1502MT02 | GQ434791 |  |  |  |  |  |
| Angiosperm | Berberidaceae | *Epimedium sagittatum* | PS1502MT03 | GQ434792 | GQ435416 |  |  | GQ436232 | GQ435802 |
| Angiosperm | Berberidaceae | *Mahonia fortunei* | PS1509MT01 | GQ434795 | GQ435419 |  |  | GQ436236 | GQ435806 |
| Angiosperm | Berberidaceae | *Mahonia fortunei* | PS1509MT03 | GQ434796 |  |  |  |  | GQ435807 |
| Angiosperm | Berberidaceae | *Nandina domestica* | PS1504MT01 |  | GQ435417 | GQ436717 |  | GQ436233 | GQ435803 |
| Angiosperm | Berberidaceae | *Podophyllum hexandrum* | PS1507MT01 | GQ434794 | GQ435418 |  |  | GQ436235 | GQ435805 |
| Angiosperm | Bignoniaceae | *Campsis grandiflora* | PS0814MT02 |  | GQ435166 |  | GQ434140 |  |  |
| Angiosperm | Bignoniaceae | *Campsis radicans* | PS0813MT01 |  | GQ435165 | GQ436508 | GQ434139 |  |  |
| Angiosperm | Bignoniaceae | *Oroxylum indicum* | PS1669MT01 | GQ434845 |  |  |  |  |  |
| Angiosperm | Bignoniaceae | *Oroxylum indicum* | PS1669MT02 | GQ434846 |  |  | GQ434292 |  |  |
| Angiosperm | Bignoniaceae | *Oroxylum indicum* | PS1669MT03 |  | GQ435463 |  |  |  |  |
| Angiosperm | Brassicaceae | *Brassica juncea* | PS1287MT01 | GQ434719 | GQ435335 | GQ436652 |  | GQ436184 | GQ435757 |
| Angiosperm | Brassicaceae | *Brassica juncea* | PS1287MT02 | GQ434720 | GQ435336 | GQ436653 |  |  |  |
| Angiosperm | Brassicaceae | *Isatis indigotica* | PS1284MT02 |  | GQ435333 |  |  |  | GQ435755 |
| Angiosperm | Brassicaceae | *Lepidium apetalum* | PS1286MT01 | GQ434718 | GQ435334 | GQ436651 |  |  | GQ435756 |
| Angiosperm | Brassicaceae | *Raphanus sativus* | PS1289MT01 | GQ434721 | GQ435337 | GQ436654 |  | GQ436185 | GQ435758 |
| Angiosperm | Brassicaceae | *Raphanus sativus* | PS1289MT02 | GQ434722 |  | GQ436655 |  |  |  |
| Angiosperm | Calycanthaceae | *Chimonanthus praecox* | PS0754MT01 | GQ434553 | GQ435155 |  | GQ434128 |  | GQ435640 |
| Angiosperm | Campanulaceae | *Codonopsis pilosula* | PS0581MT01 | GQ434466 | GQ435065 | GQ436426 |  |  | GQ435597 |
| Angiosperm | Campanulaceae | *Codonopsis tangshen* | PS0580MT01 | GQ434465 | GQ435064 |  |  |  | GQ435596 |
| Angiosperm | Campanulaceae | *Lobelia chinensis* | PS0579MT01 |  |  | GQ436424 |  |  |  |
| Angiosperm | Campanulaceae | *Lobelia chinensis* | PS0579MT02 |  | GQ435063 | GQ436425 |  |  |  |
| Angiosperm | Campanulaceae | *Platycodon grandiflorus* | PS0583MT02 | GQ434467 | GQ435066 | GQ436427 |  |  | GQ435598 |
| Angiosperm | Cannabaceae | *Cannabis sativa* | PS0216MT01 | GQ434337 |  | GQ436330 |  | GQ435928 |  |
| Angiosperm | Cannabaceae | *Cannabis sativa* | PS0216MT02 | GQ434338 | GQ434953 | GQ436331 |  | GQ435929 |  |
| Angiosperm | Cannabaceae | *Humulus scandens* | PS0217MT01 | GQ434339 | GQ434954 |  |  | GQ435930 |  |
| Angiosperm | Caprifoliaceae | *Lonicera confusa* | PS1161MT03 |  |  |  | GQ434222 |  | GQ435728 |
| Angiosperm | Caprifoliaceae | *Lonicera dasystyla* | PS1170MT01 |  | GQ435289 |  | GQ434223 |  | GQ435730 |
| Angiosperm | Caprifoliaceae | *Lonicera fulvotomentosa* | PS1743MT01 | GQ434851 | GQ435472 |  |  |  | GQ435852 |
| Angiosperm | Caprifoliaceae | *Lonicera japonica* | PS1165MT02 |  |  | GQ436618 |  | GQ436153 | GQ435729 |
| Angiosperm | Caprifoliaceae | *Lonicera macranthoides* | PS1162MT01 | GQ434673 | GQ435288 |  |  | GQ436152 |  |
| Angiosperm | Caricaceae | *Carica papaya* | PS1078MT01 |  |  | GQ436575 |  |  |  |
| Angiosperm | Caryophyllaceae | *Dianthus superbus* | PS1312MT01 | GQ434730 | GQ435348 | GQ436662 |  | GQ436189 |  |
| Angiosperm | Caryophyllaceae | *Drymaria diandra* | PS1311MT01 | GQ434729 | GQ435347 | GQ436661 |  | GQ436188 |  |
| Angiosperm | Caryophyllaceae | *Pseudostellaria heterophylla* | PS1320MT01 | GQ434732 |  |  |  |  |  |
| Angiosperm | Caryophyllaceae | *Stellaria saxatilis* | PS1317MT01 | GQ434731 | GQ435349 | GQ436663 |  | GQ436190 |  |
| Angiosperm | Caryophyllaceae | *Vaccaria segetalis* | PS1321MT01 |  | GQ435350 | GQ436664 |  | GQ436191 |  |
| Angiosperm | Celastraceae | *Maytenus austro-yunnanensis* | PS1426MT01 | GQ434764 | GQ435388 | GQ436697 |  |  | GQ435781 |
| Angiosperm | Celastraceae | *Maytenus hookeri* | PS1430MT01 | GQ434767 |  |  |  |  |  |
| Angiosperm | Celastraceae | *Tripterygium hypoglaucu* | PS1428MT01 | GQ434765 |  |  |  |  |  |
| Angiosperm | Celastraceae | *Tripterygium wilfordii* | PS1429MT01 | GQ434766 | GQ435389 | GQ436698 |  | GQ436215 | GQ435782 |
| Angiosperm | Chenopodiaceae | *Chenopodium album* | PS0771MT01 |  | GQ435157 | GQ436505 | GQ434131 |  | GQ435643 |
| Angiosperm | Chenopodiaceae | *Chenopodium ambrosioides* | PS0772MT01 | GQ434556 | GQ435158 | GQ436506 |  |  | GQ435644 |
| Angiosperm | Chenopodiaceae | *Kochia scoparia* | PS0770MT01 | GQ434555 | GQ435156 | GQ436504 |  |  |  |
| Angiosperm | Chloranthaceae | *Sarcandra glabra* | PS0548MT01 | GQ434454 |  |  |  |  |  |
| Angiosperm | Chloranthaceae | *Sarcandra glabra* | PS0548MT03 | GQ434455 |  | GQ436414 |  |  | GQ435586 |
| Angiosperm | Clusiaceae | *Hypericum japonicum* | PS1389MT01 | GQ434759 | GQ435379 | GQ436683 |  | GQ436207 |  |
| Angiosperm | Clusiaceae | *Hypericum japonicum* | PS1389MT02 | GQ434760 |  |  |  |  |  |
| Angiosperm | Clusiaceae | *Hypericum perforatum* | PS1391MT01 | GQ434761 | GQ435380 | GQ436684 |  | GQ436208 | GQ435774 |
| Angiosperm | Clusiaceae | *Mesua ferrea* | PS1395MT01 | GQ434762 | GQ435381 | GQ436685 |  |  | GQ435775 |
| Angiosperm | Combretaceae | *Quisqualis indica* | PS1325MT01 | GQ434733 | GQ435351 |  |  |  |  |
| Angiosperm | Combretaceae | *Quisqualis indica* | PS1325MT02 |  | GQ435352 |  |  |  |  |
| Angiosperm | Combretaceae | *Terminalia bellirica* | PS1326MT01 | GQ434734 |  |  |  |  |  |
| Angiosperm | Commelinaceae | *Commelina communis* | PS1546MT01 |  |  |  | GQ434279 | GQ436244 | GQ435816 |
| Angiosperm | Convolvulaceae | *Cuscuta australis* | PS1538MT01 | GQ434805 | GQ435423 | GQ436724 |  |  |  |
| Angiosperm | Convolvulaceae | *Cuscuta chinensis* | PS1540MT01 | GQ434806 |  |  |  |  |  |
| Angiosperm | Convolvulaceae | *Dichondra repens* | PS1537MT01 | GQ434804 | GQ435422 | GQ436723 |  |  |  |
| Angiosperm | Convolvulaceae | *Ipomoea nil* | PS1536MT01 | GQ434802 |  |  |  |  | GQ435812 |
| Angiosperm | Convolvulaceae | *Ipomoea nil* | PS1536MT05 | GQ434803 |  |  |  |  | GQ435813 |
| Angiosperm | Convolvulaceae | *Ipomoea nil* | PS1536MT09 |  |  | GQ436722 | GQ434278 | GQ436242 | GQ435814 |
| Angiosperm | Convolvulaceae | *Ipomoea purpurea* | PS1542MT01 | GQ434807 |  | GQ436725 |  | GQ436243 | GQ435815 |
| Angiosperm | Convolvulaceae | *Ipomoea purpurea* | PS1542MT05 | GQ434808 |  | GQ436726 |  |  |  |
| Angiosperm | Cornaceae | *Cornus officinalis* | PS1270MT02 |  | GQ435328 | GQ436648 |  |  | GQ435752 |
| Angiosperm | Cornaceae | *Helwingia japonica* | PS1755MT01 | GQ434854 | GQ435474 | GQ436764 |  |  |  |
| Angiosperm | Cornaceae | *Helwingia japonica* | PS1755MT02 | GQ434855 | GQ435475 |  |  |  |  |
| Angiosperm | Crassulaceae | *Orostachys fimbriatus* | PS1753MT01 | GQ434853 |  |  |  |  |  |
| Angiosperm | Crassulaceae | *Rhodiola crenulata* | PS0576MT01 | GQ434463 | GQ435061 |  |  |  |  |
| Angiosperm | Crassulaceae | *Sedum emarginatum* | PS0574MT03 |  | GQ435058 | GQ436420 |  |  | GQ435593 |
| Angiosperm | Crassulaceae | *Sedum lineare* | PS0577MT01 | GQ434464 | GQ435062 | GQ436423 |  |  | GQ435595 |
| Angiosperm | Crassulaceae | *Sedum sarmentosum* | PS0575MT01 | GQ434461 | GQ435059 | GQ436421 |  |  | GQ435594 |
| Angiosperm | Crassulaceae | *Sedum sarmentosum* | PS0575MT02 | GQ434462 | GQ435060 | GQ436422 |  |  |  |
| Angiosperm | Cucurbitaceae | *Benincasa hispida* | PS0460MT01 | GQ434424 | GQ435026 |  |  |  |  |
| Angiosperm | Cucurbitaceae | *Bolbostemma paniculatum* | PS0470MT01 | GQ434431 | GQ435033 | GQ436397 |  |  | GQ435578 |
| Angiosperm | Cucurbitaceae | *Gynostemma pentaphyllum* | PS0462MT01 | GQ434425 | GQ435027 | GQ436393 |  |  | GQ435572 |
| Angiosperm | Cucurbitaceae | *Gynostemma pentaphyllum* | PS0462MT02 | GQ434426 |  |  |  |  | GQ435573 |
| Angiosperm | Cucurbitaceae | *Gynostemma pentaphyllum* | PS0462MT03 | GQ434427 |  |  |  | GQ435990 | GQ435574 |
| Angiosperm | Cucurbitaceae | *Luffa cylindrica* | PS0469MT01 |  | GQ435032 |  |  |  | GQ435577 |
| Angiosperm | Cucurbitaceae | *Momordica cochinchinensis* | PS0467MT01 | GQ434430 | GQ435030 |  |  |  |  |
| Angiosperm | Cucurbitaceae | *Momordica cochinchinensis* | PS0467MT02 |  | GQ435031 | GQ436396 |  |  |  |
| Angiosperm | Cucurbitaceae | *Momordica grosvenori* | PS0464MT02 |  |  |  |  | GQ435991 | GQ435576 |
| Angiosperm | Cucurbitaceae | *Solena amplexicaulis* | PS0466MT01 | GQ434429 | GQ435029 | GQ436395 |  |  |  |
| Angiosperm | Cucurbitaceae | *Trichosanthes kirilowii* | PS0463MT01 | GQ434428 | GQ435028 | GQ436394 |  |  | GQ435575 |
| Angiosperm | Cynomoriaceae | *Cynomorium songaricum* | PS1688MT01 |  | GQ435470 |  |  |  |  |
| Angiosperm | Cyperaceae | *Cyperus rotundus* | PS1259MT01 |  |  | GQ436644 |  | GQ436181 |  |
| Angiosperm | Cyperaceae | *Cyperus rotundus* | PS1259MT04 |  |  | GQ436645 |  | GQ436182 |  |
| Angiosperm | Dioscoreaceae | *Dioscorea collettii* var. *hypoglauca* | PS1344MT01 |  | GQ435355 | GQ436670 |  | GQ436197 | GQ435765 |
| Angiosperm | Dioscoreaceae | *Dioscorea nipponica* | PS1343MT04 |  | GQ435354 | GQ436669 |  | GQ436196 |  |
| Angiosperm | Dioscoreaceae | *Dioscorea opposita* | PS1346MT01 |  |  | GQ436671 | GQ434249 |  | GQ435766 |
| Angiosperm | Dipsacaceae | *Dipsacus asperoides* | PS1514MT01 | GQ434797 |  |  |  | GQ436237 | GQ435808 |
| Angiosperm | Ebenaceae | *Diospyros kaki* | PS1329MT01 |  |  | GQ436665 | GQ434247 | GQ436192 | GQ435761 |
| Angiosperm | Elaeagnaceae | *Elaeagnus pungens* | PS0456MT01 |  | GQ435025 |  |  |  |  |
| Angiosperm | Eucommiaceae | *Eucommia ulmoides* | PS0331MT01 | GQ434388 | GQ434984 |  | GQ434085 | GQ435968 |  |
| Angiosperm | Eucommiaceae | *Eucommia ulmoides* | PS0331MT02 |  | GQ434985 |  |  | GQ435969 |  |
| Angiosperm | Euphorbiaceae | *Croton kongensis* | PS0168MT01 | GQ434326 | GQ434944 |  | GQ434077 |  | GQ435513 |
| Angiosperm | Euphorbiaceae | *Croton tiglium* | PS0167MT01 | GQ434325 |  | GQ436320 |  | GQ435919 |  |
| Angiosperm | Euphorbiaceae | *Baliospermum effusum* | PS0201MT01 | GQ434332 | GQ434951 | GQ436327 | GQ434082 |  | GQ435517 |
| Angiosperm | Euphorbiaceae | *Euphorbia hirta* | PS0175MT02 |  | GQ434945 | GQ436321 | GQ434078 | GQ435920 |  |
| Angiosperm | Euphorbiaceae | *Euphorbia hirta* | PS0175MT03 |  | GQ434946 | GQ436322 | GQ434079 | GQ435921 |  |
| Angiosperm | Euphorbiaceae | *Euphorbia lathyris* | PS0210MT01 |  | GQ434952 |  | GQ434084 | GQ435927 | GQ435518 |
| Angiosperm | Euphorbiaceae | *Euphorbia lunulata* | PS0193MT01 |  | GQ434950 | GQ436325 | GQ434081 | GQ435924 |  |
| Angiosperm | Euphorbiaceae | *Euphorbia pekinensis* | PS0187MT01 | GQ434330 | GQ434949 |  |  |  |  |
| Angiosperm | Euphorbiaceae | *Flueggea virosa* | PS0203MT01 |  |  | GQ436328 | GQ434083 | GQ435926 |  |
| Angiosperm | Euphorbiaceae | *Glochidion eriocarpum* | PS0194MT02 | GQ434331 |  | GQ436326 |  | GQ435925 | GQ435516 |
| Angiosperm | Euphorbiaceae | *Jatropha podagrica* | PS0176MT01 | GQ434328 | GQ434947 | GQ436323 | GQ434080 | GQ435922 | GQ435514 |
| Angiosperm | Euphorbiaceae | *Mallotus barbatus* | PS0205MT01 | GQ434333 |  |  |  |  |  |
| Angiosperm | Euphorbiaceae | *Phyllanthus acidus* | PS0211MT01 | GQ434334 |  | GQ436329 |  |  | GQ435519 |
| Angiosperm | Euphorbiaceae | *Phyllanthus emblica* | PS0212MT01 | GQ434335 |  |  |  |  |  |
| Angiosperm | Euphorbiaceae | *Phyllanthus emblica* | PS0212MT04 | GQ434336 |  |  |  |  |  |
| Angiosperm | Euphorbiaceae | *Ricinus communis* | PS0171MT01 | GQ434327 |  |  |  |  |  |
| Angiosperm | Fabaceae | *Abrus mollis* | PS0234MT01 | GQ434357 |  |  |  |  | GQ435526 |
| Angiosperm | Fabaceae | *Abrus mollis* | PS0234MT03 | GQ434358 |  | GQ436340 |  | GQ435943 |  |
| Angiosperm | Fabaceae | *Acacia catechu* | PS0288MT01 |  | GQ434968 | GQ436354 |  | GQ435957 | GQ435536 |
| Angiosperm | Fabaceae | *Acacia catechu* | PS0288MT02 |  | GQ434969 |  |  |  | GQ435537 |
| Angiosperm | Fabaceae | *Acacia catechu* | PS0288MT06 |  | GQ434970 | GQ436355 |  |  | GQ435538 |
| Angiosperm | Fabaceae | *Acacia confusa* | PS0402MT04 |  | GQ435006 | GQ436378 |  | GQ435983 |  |
| Angiosperm | Fabaceae | *Adenanthera pavonina* var. *microsperma* | PS0400MT01 | GQ434409 | GQ435005 | GQ436377 |  | GQ435982 | GQ435561 |
| Angiosperm | Fabaceae | *Astragalus chinensis* | PS0272MT01 | GQ434365 |  | GQ436349 |  |  |  |
| Angiosperm | Fabaceae | *Astragalus chinensis* | PS0272MT02 | GQ434366 | GQ434966 | GQ436350 |  | GQ435951 | GQ435532 |
| Angiosperm | Fabaceae | *Astragalus hancockii* | PS0271MT01 | GQ434364 |  | GQ436348 |  | GQ435950 |  |
| Angiosperm | Fabaceae | *Astragalus mongholicus* | PS0277MT01 | GQ434368 |  |  |  | GQ435953 | GQ435534 |
| Angiosperm | Fabaceae | *Caesalpinia sappan* | PS1370MT04 | GQ434751 | GQ435369 |  |  |  | GQ435769 |
| Angiosperm | Fabaceae | *Caesalpinia sappan* | PS1370MT06 | GQ434752 | GQ435370 |  |  |  | GQ435770 |
| Angiosperm | Fabaceae | *Caragana arborescens* | PS0293MT01 | GQ434373 |  | GQ436356 |  | GQ435958 |  |
| Angiosperm | Fabaceae | *Cassia alata* | PS1362MT02 | GQ434748 | GQ435366 | GQ436678 |  |  |  |
| Angiosperm | Fabaceae | *Cassia fistula* | PS1369MT02 | GQ434750 | GQ435368 |  |  |  | GQ435768 |
| Angiosperm | Fabaceae | *Cassia nodosa* | PS1365MT01 | GQ434749 | GQ435367 | GQ436679 |  |  | GQ435767 |
| Angiosperm | Fabaceae | *Cassia obtusifolia* | PS1588MT06 | GQ434815 | GQ435431 |  | GQ434282 |  | GQ435821 |
| Angiosperm | Fabaceae | *Cassia obtusifolia* | PS1588MT08 | GQ434816 | GQ435432 | GQ436733 |  |  |  |
| Angiosperm | Fabaceae | *Cercis chinensis* | PS0316MT01 | GQ434380 | GQ434979 |  |  |  |  |
| Angiosperm | Fabaceae | *Crotalaria usaramoensis* | PS0226MT01 | GQ434348 |  | GQ436334 |  | GQ435936 |  |
| Angiosperm | Fabaceae | *Dalbergia odorifera* | PS0262MT02 | GQ434362 |  |  |  | GQ435947 |  |
| Angiosperm | Fabaceae | *Dalbergia odorifera* | PS0262MT03 |  | GQ434964 |  |  |  |  |
| Angiosperm | Fabaceae | *Desmodium styracifolium* | PS0232MT02 | GQ434353 |  | GQ436338 |  | GQ435939 |  |
| Angiosperm | Fabaceae | *Desmodium styracifolium* | PS0232MT04 | GQ434354 |  | GQ436339 |  | GQ435940 |  |
| Angiosperm | Fabaceae | *Desmodium styracifolium* | PS0232MT05 | GQ434355 |  |  |  | GQ435941 | GQ435524 |
| Angiosperm | Fabaceae | *Desmodium styracifolium* | PS0232MT06 | GQ434356 | GQ434961 |  |  | GQ435942 | GQ435525 |
| Angiosperm | Fabaceae | *Dolichos lablab* | PS0268MT01 |  | GQ434965 | GQ436347 |  | GQ435949 | GQ435531 |
| Angiosperm | Fabaceae | *Entada phaseoloides* | PS0290MT02 | GQ434372 | GQ434971 |  |  |  | GQ435539 |
| Angiosperm | Fabaceae | *Flemingia glutinosa* | PS0223MT01 | GQ434347 | GQ434959 | GQ436333 |  |  | GQ435522 |
| Angiosperm | Fabaceae | *Flemingia macrophylla* | PS0222MT02 | GQ434345 |  | GQ436332 |  |  |  |
| Angiosperm | Fabaceae | *Flemingia macrophylla* | PS0222MT03 | GQ434346 | GQ434958 |  |  | GQ435935 | GQ435521 |
| Angiosperm | Fabaceae | *Fordia cauliflora* | PS0230MT01 | GQ434352 |  | GQ436337 |  | GQ435938 |  |
| Angiosperm | Fabaceae | *Gleditsia sinensis* | PS0315MT01 |  | GQ434978 | GQ436363 |  | GQ435966 | GQ435546 |
| Angiosperm | Fabaceae | *Glycine max* | PS1684MT01 | GQ434849 | GQ435466 | GQ436762 |  |  | GQ435850 |
| Angiosperm | Fabaceae | *Gueldenstaedtia multiflora* | PS0302MT01 | GQ434375 |  | GQ436359 |  | GQ435962 |  |
| Angiosperm | Fabaceae | *Hedysarum polybotrys* | PS0274MT01 | GQ434367 |  | GQ436351 |  | GQ435952 | GQ435533 |
| Angiosperm | Fabaceae | *Indigofera tinctoria* | PS0251MT04 | GQ434361 |  |  |  | GQ435946 | GQ435530 |
| Angiosperm | Fabaceae | *Lathyrus davidii* | PS0282MT01 | GQ434370 |  | GQ436352 |  | GQ435955 |  |
| Angiosperm | Fabaceae | *Lespedeza floribunda* | PS0286MT01 | GQ434371 |  | GQ436353 |  | GQ435956 |  |
| Angiosperm | Fabaceae | *Lotus corniculatus* | PS0220MT01 | GQ434343 |  |  |  | GQ435933 |  |
| Angiosperm | Fabaceae | *Millettia dielsiana* | PS0309MT01 | GQ434376 | GQ434974 | GQ436360 |  |  |  |
| Angiosperm | Fabaceae | *Mucuna hainanensis* | PS0235MT01 |  |  | GQ436341 |  |  | GQ435527 |
| Angiosperm | Fabaceae | *Pachyrhizus erosus* | PS0227MT01 | GQ434349 |  | GQ436335 |  |  |  |
| Angiosperm | Fabaceae | *Pachyrhizus erosus* | PS0227MT02 | GQ434350 |  | GQ436336 |  |  |  |
| Angiosperm | Fabaceae | *Phaseolus angularis* | PS0279MT01 | GQ434369 | GQ434967 |  |  | GQ435954 | GQ435535 |
| Angiosperm | Fabaceae | *Pithecellobium clypearia* | PS0296MT01 |  | GQ434972 | GQ436357 |  | GQ435959 | GQ435540 |
| Angiosperm | Fabaceae | *Pithecellobium clypearia* | PS0296MT02 |  |  |  |  | GQ435960 | GQ435541 |
| Angiosperm | Fabaceae | *Psoralea corylifolia* | PS0221MT04 | GQ434344 | GQ434957 |  |  | GQ435934 |  |
| Angiosperm | Fabaceae | *Pueraria lobata* | PS0313MT03 | GQ434377 | GQ434975 |  |  | GQ435963 | GQ435543 |
| Angiosperm | Fabaceae | *Pueraria lobata* | PS0313MT04 | GQ434378 | GQ434976 | GQ436361 |  | GQ435964 | GQ435544 |
| Angiosperm | Fabaceae | *Pueraria peduncularis* | PS0314MT01 | GQ434379 | GQ434977 | GQ436362 |  | GQ435965 | GQ435545 |
| Angiosperm | Fabaceae | *Shuteria pampaniniana* | PS0301MT01 | GQ434374 | GQ434973 | GQ436358 |  | GQ435961 | GQ435542 |
| Angiosperm | Fabaceae | *Sophora flavescens* | PS0247MT04 | GQ434360 | GQ434963 | GQ436344 |  | GQ435945 | GQ435529 |
| Angiosperm | Fabaceae | *Sophora japonica* | PS0241MT03 |  |  | GQ436343 |  |  |  |
| Angiosperm | Fabaceae | *Sophora tonkinensis* | PS0228MT01 | GQ434351 | GQ434960 |  |  | GQ435937 | GQ435523 |
| Angiosperm | Fabaceae | *Tadehagi triquetrum* | PS0237MT02 | GQ434359 | GQ434962 | GQ436342 |  | GQ435944 | GQ435528 |
| Angiosperm | Fabaceae | *Trifolium repens* | PS0265MT01 | GQ434363 |  | GQ436346 |  |  |  |
| Angiosperm | Fabaceae | *Trigonella foenum-graecum* | PS0319MT01 | GQ434381 | GQ434980 |  |  | GQ435967 | GQ435547 |
| Angiosperm | Fabaceae | *Whitfordiodendron filipes* | PS0264MT01 |  |  | GQ436345 |  | GQ435948 |  |
| Angiosperm | Flacourtiaceae | *Hydnocarpus anthelminticus* | PS0165MT02 |  |  |  |  |  | GQ435512 |
| Angiosperm | Gentianaceae | *Gentiana macrophylla* | PS0821MT02 | GQ434565 | GQ435169 | GQ436510 | GQ434141 | GQ436048 |  |
| Angiosperm | Gentianaceae | *Gentiana manshurica* | PS0823MT01 | GQ434566 |  |  |  |  |  |
| Angiosperm | Gentianaceae | *Gentiana rigescens* | PS0822MT01 |  | GQ435170 |  |  | GQ436049 |  |
| Angiosperm | Gentianaceae | *Gentiana straminea* | PS0820MT01 | GQ434564 | GQ435167 | GQ436509 |  |  |  |
| Angiosperm | Gentianaceae | *Gentiana straminea* | PS0820MT02 |  | GQ435168 |  |  | GQ436047 |  |
| Angiosperm | Geraniaceae | *Erodium stephanianum* | PS0896MT02 | GQ434598 | GQ435200 |  |  | GQ436067 |  |
| Angiosperm | Hamamelidaceae | *Liquidambar formosana* | PS0541MT02 |  | GQ435052 |  |  |  |  |
| Angiosperm | Hippocastanaceae | *Aesculus chinensis* | PS0999MT01 | GQ434624 |  | GQ436546 |  | GQ436089 | GQ435689 |
| Angiosperm | Iridaceae | *Belamcanda chinensis* | PS0056MT01 |  | GQ434907 |  |  | GQ435894 |  |
| Angiosperm | Iridaceae | *Belamcanda chinensis* | PS0056MT02 |  | GQ434908 | GQ436298 |  | GQ435895 | GQ435499 |
| Angiosperm | Iridaceae | *Iris tectorum* | PS1583MT01 |  | GQ435429 |  |  | GQ436248 |  |
| Angiosperm | Iridaceae | *Iris tectorum* | PS1583MT03 | GQ434814 | GQ435430 | GQ436732 |  | GQ436249 | GQ435820 |
| Angiosperm | Juglandaceae | *Juglans regia* | PS0454MT01 | GQ434423 | GQ435024 | GQ436392 |  |  | GQ435571 |
| Angiosperm | Juncaceae | *Juncus effusus* | PS0218MT01 | GQ434340 | GQ434955 |  |  | GQ435931 | GQ435520 |
| Angiosperm | Juncaceae | *Juncus effusus* | PS0218MT02 | GQ434341 | GQ434956 |  |  |  |  |
| Angiosperm | Juncaceae | *Juncus effusus* | PS0218MT03 | GQ434342 |  |  |  | GQ435932 |  |
| Angiosperm | Lamiaceae | *Ajuga ciliata* | PS0125MT01 | GQ434321 |  |  |  |  |  |
| Angiosperm | Lamiaceae | *Ajuga decumbens* | PS1738MT01 | FJ883496 |  |  |  |  |  |
| Angiosperm | Lamiaceae | *Ajuga lupulina* | PS0104MT01 | GQ434317 |  |  |  |  |  |
| Angiosperm | Lamiaceae | *Clerodendranthus spicatus* | PS0141MT01 |  | GQ464982 |  |  |  |  |
| Angiosperm | Lamiaceae | *Clerodendranthus spicatus* | PS0141MT02 |  | FJ513103 | GQ464985 |  | EU590889 | FJ513182 |
| Angiosperm | Lamiaceae | *Glechoma longituba* | PS0123MT02 |  | EU590860 | FJ513153 | FJ513163 | EU590884 |  |
| Angiosperm | Lamiaceae | *Leonurus artemisia* | PS0154MT01 |  | FJ513112 | FJ513159 | FJ513164 | EU590885 |  |
| Angiosperm | Lamiaceae | *Mentha haplocalyx* | PS0108MT01 |  | GQ434942 |  |  |  |  |
| Angiosperm | Lamiaceae | *Phlomis melanantha* | PS1733MT01 | FJ883498 |  |  |  |  |  |
| Angiosperm | Lamiaceae | *Pogostemon cablin* | PS0119MT01 |  | EU590858 | FJ513150 | FJ513165 | EU590883 |  |
| Angiosperm | Lamiaceae | *Pogostemon cablin* | PS0119MT02 | GQ434319 | GQ464980 | GQ464984 | GQ464979 | GQ464977 |  |
| Angiosperm | Lamiaceae | *Pogostemon cablin* | PS0119MT06 |  | GQ464981 |  |  | GQ464978 |  |
| Angiosperm | Lamiaceae | *Prunella vulgaris* | PS0144MT01 |  | EU590863 | FJ513157 | FJ513166 | EU590886 |  |
| Angiosperm | Lamiaceae | *Salvia miltiorrhiza* | PS0110MT01 | GQ434318 | EU590856 | FJ513145 | FJ513167 | EU590881 | FJ513174 |
| Angiosperm | Lamiaceae | *Salvia przewalskii* | PS1708MT03 |  | GQ435471 |  |  |  |  |
| Angiosperm | Lamiaceae | *Salvia splendens* | PS0153MT01 | GQ434322 |  |  |  |  |  |
| Angiosperm | Lamiaceae | *Salvia splendens* | PS0153MT02 | FJ883530 |  |  |  |  |  |
| Angiosperm | Lamiaceae | *Scutellaria indica* | PS0120MT01 | GQ434320 |  |  |  |  |  |
| Angiosperm | Lardizabalaceae | *Akebia quinata* | PS0958MT01 | GQ434614 | GQ435220 | GQ436540 | GQ434169 | GQ436083 | GQ435682 |
| Angiosperm | Lardizabalaceae | *Akebia trifoliata* subsp. *trifoliata* | PS0959MT01 | GQ434615 |  |  | GQ434170 | GQ436084 | GQ435683 |
| Angiosperm | Lardizabalaceae | *Akebia trifoliata* subsp. *trifoliata* | PS0959MT02 | GQ434616 |  |  | GQ434171 |  | GQ435684 |
| Angiosperm | Lardizabalaceae | *Akebia trifoliata* var. *australis* | PS0954MT01 |  |  | GQ436538 | GQ434168 |  | GQ435680 |
| Angiosperm | Lardizabalaceae | *Akebia trifoliata* var. *australis* | PS0954MT02 |  | GQ435219 |  |  |  | GQ435681 |
| Angiosperm | Lauraceae | *Cassytha filiformis* | PS1631MT01 |  |  |  |  |  | GQ435842 |
| Angiosperm | Lauraceae | *Cinnamomum burmannii* | PS1633MT04 |  |  |  | GQ434290 | GQ436267 | GQ435843 |
| Angiosperm | Lauraceae | *Cinnamomum camphora* | PS1634MT02 |  | GQ435461 | GQ436752 |  |  |  |
| Angiosperm | Lauraceae | *Cinnamomum cassia* | PS1628MT01 |  |  |  |  | GQ436265 | GQ435839 |
| Angiosperm | Lauraceae | *Cinnamomum cassia* | PS1628MT02 |  |  |  | GQ434289 |  | GQ435840 |
| Angiosperm | Lauraceae | *Cinnamomum porrectum* | PS1626MT01 |  | GQ435459 |  | GQ434288 |  | GQ435838 |
| Angiosperm | Lauraceae | *Lindera aggregata* | PS1630MT01 |  | GQ435460 |  |  | GQ436266 | GQ435841 |
| Angiosperm | Lauraceae | *Litsea cubeba* | PS1623MT01 |  | GQ435457 |  |  | GQ436264 | GQ435837 |
| Angiosperm | Lauraceae | *Litsea glutinosa* | PS1625MT01 |  | GQ435458 |  | GQ434287 |  |  |
| Angiosperm | Lemnaceae | *Lemna minor* | PS0367MT01 |  | GQ434996 | GQ436374 |  | GQ435979 | GQ435559 |
| Angiosperm | Liliaceae | *Allium macrostemon* | PS0065MT01 |  | GQ434917 |  |  | GQ435905 | GQ435503 |
| Angiosperm | Liliaceae | *Allium macrostemon* | PS0065MT02 |  | GQ434918 | GQ436306 |  |  | GQ435504 |
| Angiosperm | Liliaceae | *Allium macrostemon* | PS0065MT03 |  | GQ434919 | GQ436307 | GQ434063 | GQ435906 |  |
| Angiosperm | Liliaceae | *Allium senescens* | PS0054MT01 |  | GQ434905 |  | GQ434052 | GQ435892 |  |
| Angiosperm | Liliaceae | *Allium tuberosum* | PS0038MT01 |  | GQ434884 |  |  | GQ435876 | GQ435491 |
| Angiosperm | Liliaceae | *Allium tuberosum* | PS0038MT02 |  | GQ434885 |  |  | GQ435877 | GQ435492 |
| Angiosperm | Liliaceae | *Allium tuberosum* | PS0038MT03 | GQ434312 | GQ434886 |  |  |  | GQ435493 |
| Angiosperm | Liliaceae | *Allium tuberosum* | PS0038MT04 |  | GQ434887 |  |  | GQ435878 |  |
| Angiosperm | Liliaceae | *Allium tuberosum* | PS0038MT05 |  | GQ434888 | GQ436291 |  | GQ435879 |  |
| Angiosperm | Liliaceae | *Aloe arborescens* | PS0045MT01 |  | GQ434898 |  |  | GQ435885 |  |
| Angiosperm | Liliaceae | *Aloe barbadensi* | PS0047MT01 |  |  |  | GQ434051 | GQ435887 | GQ435495 |
| Angiosperm | Liliaceae | *Aloe vera* | PS0044MT01 |  | GQ434894 |  | GQ434047 | GQ435883 |  |
| Angiosperm | Liliaceae | *Aloe vera* | PS0044MT02 |  | GQ434895 |  | GQ434048 |  |  |
| Angiosperm | Liliaceae | *Aloe vera* | PS0044MT03 |  | GQ434896 |  | GQ434049 | GQ435884 |  |
| Angiosperm | Liliaceae | *Aloe vera* | PS0044MT04 |  | GQ434897 |  | GQ434050 |  |  |
| Angiosperm | Liliaceae | *Aloe vera* var. *chinensis* | PS0046MT01 |  | GQ434899 |  |  | GQ435886 |  |
| Angiosperm | Liliaceae | *Anemarrhena asphodeloides* | PS0072MT01 |  | GQ434926 |  | GQ434069 | GQ435910 | GQ435506 |
| Angiosperm | Liliaceae | *Anemarrhena asphodeloides* | PS0072MT03 |  | GQ434927 | GQ436312 | GQ434070 |  |  |
| Angiosperm | Liliaceae | *Anemarrhena asphodeloides* | PS0072MT04 |  | GQ434928 | GQ436313 | GQ434071 | GQ435911 |  |
| Angiosperm | Liliaceae | *Anemarrhena asphodeloides* | PS0072MT05 |  | GQ434929 | GQ436314 | GQ434072 | GQ435912 |  |
| Angiosperm | Liliaceae | *Asparagus cochinchinensis* | PS0057MT01 | GQ434315 | GQ434909 |  |  | GQ435896 | GQ435500 |
| Angiosperm | Liliaceae | *Asparagus cochinchinensis* | PS0057MT04 |  | GQ434910 | GQ436299 | GQ434053 |  |  |
| Angiosperm | Liliaceae | *Asparagus cochinchinensis* | PS0057MT05 |  |  |  | GQ434054 | GQ435897 | GQ435501 |
| Angiosperm | Liliaceae | *Asparagus cochinchinensis* | PS0057MT06 |  | GQ434911 | GQ436300 | GQ434055 |  | GQ435502 |
| Angiosperm | Liliaceae | *Asparagus schoberioides* | PS0058MT01 |  | GQ434912 | GQ436301 | GQ434056 | GQ435898 |  |
| Angiosperm | Liliaceae | *Asparagus trichophyllus* | PS0059MT01 |  | GQ434913 |  | GQ434057 | GQ435899 |  |
| Angiosperm | Liliaceae | *Asparagus trichophyllus* | PS0059MT02 |  | GQ434914 | GQ436302 | GQ434058 | GQ435900 |  |
| Angiosperm | Liliaceae | *Aspidistra elatior* | PS0762MT01 |  |  |  | GQ434130 |  | GQ435642 |
| Angiosperm | Liliaceae | *Chlorophytum elatum* var. *variegatum* | PS0037MT01 |  | GQ434883 | GQ436290 | GQ434043 | GQ435875 |  |
| Angiosperm | Liliaceae | *Convallaria majalis* | PS0043MT01 |  | GQ434893 | GQ436294 | GQ434046 | GQ435882 |  |
| Angiosperm | Liliaceae | *Cordyline fruticosa* | PS0073MT01 |  | GQ434930 |  | GQ434073 | GQ435913 |  |
| Angiosperm | Liliaceae | *Dianella ensifolia* | PS0053MT03 | GQ434314 |  |  |  |  |  |
| Angiosperm | Liliaceae | *Dianella ensifolia* | PS0053MT04 |  | GQ434904 | GQ436296 |  |  |  |
| Angiosperm | Liliaceae | *Disporum cantoniense* | PS0062MT01 |  | GQ434915 |  | GQ434060 | GQ435902 |  |
| Angiosperm | Liliaceae | *Dracaena cambodiana* | PS0825MT01 |  | GQ435171 |  |  |  | GQ435648 |
| Angiosperm | Liliaceae | *Dracaena cochinchinensis* | PS0036MT01 |  | GQ434882 |  |  | GQ435874 |  |
| Angiosperm | Liliaceae | *Fritillaria unibracteata* | PS0021MT01 |  | GQ434876 |  |  | GQ435868 |  |
| Angiosperm | Liliaceae | *Hemerocallis fulva* | PS0066MT01 |  | GQ434920 | GQ436308 | GQ434064 |  |  |
| Angiosperm | Liliaceae | *Hemerocallis fulva* | PS0066MT02 |  | GQ434921 | GQ436309 | GQ434065 |  |  |
| Angiosperm | Liliaceae | *Hemerocallis fulva* | PS0066MT03 |  | GQ434922 | GQ436310 | GQ434066 |  |  |
| Angiosperm | Liliaceae | *Lilium concolor* var. *pulchellum* | PS0069MT01 |  | GQ434923 | GQ436311 | GQ434067 | GQ435907 |  |
| Angiosperm | Liliaceae | *Lilium davidii* | PS0024MT01 |  | GQ434877 | GQ436288 |  | GQ435869 |  |
| Angiosperm | Liliaceae | *Lilium lancifolium* | PS0039MT01 |  | GQ434889 | GQ436292 | GQ434044 | GQ435880 |  |
| Angiosperm | Liliaceae | *Lilium pumilum* | PS0064MT01 |  | GQ434916 | GQ436305 | GQ434062 | GQ435904 |  |
| Angiosperm | Liliaceae | *Liriope platyphylla* | PS0041MT01 |  | GQ434890 |  |  | GQ435881 | GQ435494 |
| Angiosperm | Liliaceae | *Liriope platyphylla* | PS0041MT02 |  | GQ434891 |  |  |  |  |
| Angiosperm | Liliaceae | *Liriope spicata* | PS0055MT01 |  | GQ434906 | GQ436297 |  | GQ435893 | GQ435498 |
| Angiosperm | Liliaceae | *Maianthemum bifolium* | PS0063MT01 |  |  | GQ436304 | GQ434061 | GQ435903 |  |
| Angiosperm | Liliaceae | *Ophiopogon japonicus* | PS0049MT02 | GQ434313 | GQ434901 |  |  | GQ435889 | GQ435496 |
| Angiosperm | Liliaceae | *Ophiopogon japonicus* | PS0049MT03 |  | GQ434902 |  |  | GQ435890 | GQ435497 |
| Angiosperm | Liliaceae | *Polygonatum involucratum* | PS0028MT01 |  | GQ434878 |  |  | GQ435870 |  |
| Angiosperm | Liliaceae | *Polygonatum macropodium* | PS0052MT02 |  | GQ434903 |  |  | GQ435891 |  |
| Angiosperm | Liliaceae | *Polygonatum odoratum* | PS0070MT01 |  | GQ434924 |  | GQ434068 | GQ435908 |  |
| Angiosperm | Liliaceae | *Polygonatum odoratum* | PS0070MT03 |  | GQ434925 |  |  | GQ435909 | GQ435505 |
| Angiosperm | Liliaceae | *Polygonatum sibiricum* | PS0033MT01 |  | GQ434879 |  | GQ434040 | GQ435871 |  |
| Angiosperm | Liliaceae | *Polygonatum sibiricum* | PS0033MT03 |  | GQ434880 |  | GQ434041 | GQ435872 | GQ435490 |
| Angiosperm | Liliaceae | *Reineckia carnea* | PS0035MT01 |  | GQ434881 | GQ436289 | GQ434042 | GQ435873 |  |
| Angiosperm | Liliaceae | *Rohdea japonica* | PS0061MT01 |  |  | GQ436303 | GQ434059 | GQ435901 |  |
| Angiosperm | Liliaceae | *Smilacina japonica* | PS0048MT01 |  | GQ434900 | GQ436295 |  | GQ435888 |  |
| Angiosperm | Liliaceae | *Smilax chinensis* | PS0007MT01 |  |  |  |  | GQ435860 |  |
| Angiosperm | Liliaceae | *Smilax chinensis* | PS0007MT02 |  |  | GQ436282 |  |  |  |
| Angiosperm | Liliaceae | *Smilax ocreata* | PS0008MT01 |  | GQ434872 | GQ436283 | GQ434034 |  |  |
| Angiosperm | Liliaceae | *Veratrum nigrum* | PS0042MT01 |  | GQ434892 | GQ436293 | GQ434045 |  |  |
| Angiosperm | Loganiaceae | *Buddleja officinalis* | PS0892MT01 |  |  |  | GQ434158 |  | GQ435664 |
| Angiosperm | Loganiaceae | *Buddleja officinalis* | PS0892MT02 | GQ434596 |  |  | GQ434159 |  | GQ435665 |
| Angiosperm | Loganiaceae | *Strychnos nux-vomica* | PS0891MT02 | GQ434594 | GQ435195 | GQ436530 | GQ434154 |  | GQ435662 |
| Angiosperm | Loganiaceae | *Strychnos nux-vomica* | PS0891MT03 |  | GQ435196 | GQ436531 | GQ434155 |  |  |
| Angiosperm | Loganiaceae | *Strychnos nux-vomica* | PS0891MT04 |  | GQ435197 | GQ436532 | GQ434156 |  |  |
| Angiosperm | Loganiaceae | *Strychnos nux-vomica* | PS0891MT05 | GQ434595 | GQ435198 |  | GQ434157 | GQ436066 | GQ435663 |
| Angiosperm | Loranthaceae | *Taxillus chinensis* | PS1685MT01 |  | GQ435467 |  |  |  | GQ435851 |
| Angiosperm | Loranthaceae | *Viscum liquidambaricolum* | PS1227MT01 | GQ434707 |  | GQ436640 |  | GQ436177 |  |
| Angiosperm | Loranthaceae | *Viscum liquidambaricolum* | PS1227MT02 |  | GQ435320 |  |  | GQ436178 |  |
| Angiosperm | Lythraceae | *Lawsonia inermis* | PS1016MT01 | GQ434628 | GQ435226 | GQ436549 |  |  | GQ435692 |
| Angiosperm | Lythraceae | *Woodfordia fruticosa* | PS1017MT02 |  | GQ435227 | GQ436550 |  |  | GQ435693 |
| Angiosperm | Magnoliaceae | *Illicium verum* | PS0005MT02 |  |  |  | GQ434032 |  | GQ435483 |
| Angiosperm | Magnoliaceae | *Illicium verum* | PS0005MT03 | GQ434311 |  |  | GQ434033 | GQ435859 | GQ435484 |
| Angiosperm | Magnoliaceae | *Magnolia biondii* | PS0945MT02 |  | GQ435217 |  | GQ434166 | GQ436081 | GQ435678 |
| Angiosperm | Magnoliaceae | *Magnolia champaca* | PS0943MT01 |  | GQ435216 | GQ436537 | GQ434165 |  | GQ435677 |
| Angiosperm | Magnoliaceae | *Magnolia denudata* | PS0949MT01 |  | GQ435218 |  | GQ434167 |  | GQ435679 |
| Angiosperm | Magnoliaceae | *Magnolia grandiflora* | PS0941MT01 |  | GQ435214 |  | GQ434163 | GQ436079 | GQ435675 |
| Angiosperm | Magnoliaceae | *Magnolia officinalis* | PS0942MT02 |  | GQ435215 |  | GQ434164 | GQ436080 | GQ435676 |
| Angiosperm | Malpighiaceae | *Aspidopterys obcordata* | PS0538MT01 | GQ434453 |  |  |  |  |  |
| Angiosperm | Malvaceae | *Abutilon theophrasti* | PS0569MT04 | GQ434460 | GQ435057 | GQ436419 |  |  | GQ435592 |
| Angiosperm | Malvaceae | *Hibiscus mutabilis* | PS0567MT04 | GQ434458 |  |  |  |  | GQ435590 |
| Angiosperm | Malvaceae | *Hibiscus syriacus* | PS0568MT02 | GQ434459 | GQ435056 | GQ436418 |  |  | GQ435591 |
| Angiosperm | Malvaceae | *Urena lobata* | PS0563MT02 |  | GQ435055 |  |  |  |  |
| Angiosperm | Melastomataceae | *Melastoma candidum* | PS1560MT01 | GQ434810 | GQ435425 | GQ436728 |  |  |  |
| Angiosperm | Melastomataceae | *Melastoma dodecandrum* | PS1559MT01 | GQ434809 | GQ435424 | GQ436727 |  |  |  |
| Angiosperm | Meliaceae | *Melia azedarach* | PS0780MT03 |  | GQ435160 |  |  |  | GQ435646 |
| Angiosperm | Meliaceae | *Melia toosendan* | PS0777MT01 | GQ434557 | GQ435159 |  |  |  | GQ435645 |
| Angiosperm | Menispermaceae | *Cissampelos pareira* var. *hirsuta* | PS0358MT01 | GQ434399 |  | GQ436372 |  |  | GQ435558 |
| Angiosperm | Menispermaceae | *Cocculus orbiculatus* | PS0353MT02 | GQ434395 | GQ434990 |  |  | GQ435975 | GQ435556 |
| Angiosperm | Menispermaceae | *Cocculus orbiculatus* | PS0353MT03 | GQ434396 | GQ434991 |  |  | GQ435976 | GQ435557 |
| Angiosperm | Menispermaceae | *Cocculus orbiculatus* | PS0353MT04 | GQ434397 | GQ434992 |  |  |  |  |
| Angiosperm | Menispermaceae | *Cyclea racemosa* | PS0352MT01 | GQ434394 |  |  |  |  |  |
| Angiosperm | Menispermaceae | *Fibraurea recisa* | PS0350MT05 | GQ434393 |  |  |  | GQ435974 | GQ435555 |
| Angiosperm | Menispermaceae | *Menispermum dauricum* | PS0345MT01 | GQ434390 |  | GQ436368 |  |  | GQ435550 |
| Angiosperm | Menispermaceae | *Menispermum dauricum* | PS0345MT04 | GQ434391 | GQ434987 | GQ436369 |  | GQ435970 |  |
| Angiosperm | Menispermaceae | *Sinomenium acutum* | PS0347MT01 | GQ434392 |  |  |  |  | GQ435551 |
| Angiosperm | Menispermaceae | *Sinomenium acutum* | PS0347MT03 |  |  |  |  | GQ435971 | GQ435552 |
| Angiosperm | Menispermaceae | *Stephania tetrandra* | PS0348MT01 |  | GQ434988 |  |  | GQ435972 | GQ435553 |
| Angiosperm | Menispermaceae | *Stephania tetrandra* | PS0348MT02 |  | GQ434989 | GQ436370 |  | GQ435973 | GQ435554 |
| Angiosperm | Menispermaceae | *Tinospora sagittata* | PS0356MT01 | GQ434398 | GQ434993 | GQ436371 | GQ434086 | GQ435977 |  |
| Angiosperm | Molluginaceae | *Mollugo stricta* | PS1375MT01 | GQ434755 | GQ435373 |  |  |  | GQ435771 |
| Angiosperm | Moraceae | *Antiaris toxicaria* | PS1239MT02 | GQ434711 | GQ435322 | GQ436642 | GQ434236 |  | GQ435750 |
| Angiosperm | Moraceae | *Broussonetia papyrifera* | PS0178MT04 | GQ434329 | GQ434948 | GQ436324 |  | GQ435923 | GQ435515 |
| Angiosperm | Moraceae | *Ficus pumila* | PS1229MT01 | GQ434708 |  |  |  |  | GQ435748 |
| Angiosperm | Moraceae | *Ficus pumila* | PS1229MT05 | GQ434709 | GQ435321 |  |  | GQ436179 |  |
| Angiosperm | Moraceae | *Morus alba* | PS1246MT01 | GQ434713 |  |  | GQ434237 | GQ436180 | GQ435751 |
| Angiosperm | Moraceae | *Morus alba* | PS1246MT02 | GQ434714 | GQ435324 |  |  |  |  |
| Angiosperm | Moraceae | *Streblus asper* | PS1244MT01 | GQ434712 | GQ435323 | GQ436643 |  |  |  |
| Angiosperm | Moraceae | *Streblus indicus* | PS1238MT01 | GQ434710 |  | GQ436641 | GQ434235 |  | GQ435749 |
| Angiosperm | Myrsinaceae | *Ardisia crenata* | PS1658MT01 | GQ434841 |  | GQ436754 |  |  |  |
| Angiosperm | Myrsinaceae | *Ardisia crenata* | PS1658MT02 | GQ434842 |  |  |  |  |  |
| Angiosperm | Myrsinaceae | *Ardisia crenata* | PS1658MT03 |  |  | GQ436755 |  |  |  |
| Angiosperm | Myrsinaceae | *Ardisia elliptisepala* | PS1657MT01 | GQ434840 |  | GQ436753 |  |  | GQ435844 |
| Angiosperm | Myrsinaceae | *Ardisia japonica* | PS1659MT04 | GQ434843 |  | GQ436756 |  |  |  |
| Angiosperm | Myrsinaceae | *Ardisia japonica* | PS1659MT05 | GQ434844 |  |  |  |  | GQ435845 |
| Angiosperm | Myrtaceae | *Psidium guajava* | PS0344MT01 |  | GQ434986 |  |  |  |  |
| Angiosperm | Myrtaceae | *Syzygium buxifolium* | PS1379MT01 |  | GQ435378 |  |  |  |  |
| Angiosperm | Oleaceae | *Forsythia suspensa* | PS0968MT01 | GQ434618 | GQ435222 | GQ436541 | GQ434172 | GQ436086 |  |
| Angiosperm | Oleaceae | *Fraxinus chinensis* | PS0962MT01 | GQ434617 | GQ435221 |  |  | GQ436085 | GQ435685 |
| Angiosperm | Oleaceae | *Jasminum nervosum* | PS0975MT01 | GQ434620 | GQ435223 | GQ436543 | GQ434173 |  | GQ435686 |
| Angiosperm | Oleaceae | *Ligustrum lucidum* | PS0973MT02 | GQ434619 |  | GQ436542 |  |  |  |
| Angiosperm | Oleaceae | *Ligustrum quihoui* | PS0977MT01 | GQ434621 | GQ435224 | GQ436544 | GQ434174 |  | GQ435687 |
| Angiosperm | Orchidaceae | *Bletilla striata* | PS0756MT02 | GQ434554 |  |  | GQ434129 |  |  |
| Angiosperm | Orchidaceae | *Bulbophyllum odoratissimum* | PS2508MT01 |  | EU887945 | FJ216552 | FJ216638 | EU553947 | EU553964 |
| Angiosperm | Orchidaceae | *Dendrobium acinaciforme* | PS2527MT01 |  | EU887944 | FJ216578 |  |  | FJ216694 |
| Angiosperm | Orchidaceae | *Dendrobium aphyllum* | PS2523MT01 |  | EU887939 | FJ216571 | FJ216658 | FJ216504 | FJ216688 |
| Angiosperm | Orchidaceae | *Dendrobium aphyllum* | PS2523MT02 |  | FJ216486 | FJ216575 |  | FJ216507 | FJ216691 |
| Angiosperm | Orchidaceae | *Dendrobium capillipes* | PS2502MT01 |  | EU887926 | FJ216545 | FJ216630 | EU553943 | EU553960 |
| Angiosperm | Orchidaceae | *Dendrobium chrysanthum* | PS2515MT01 |  | EU887934 | FJ216560 | FJ216646 | FJ216499 | FJ216684 |
| Angiosperm | Orchidaceae | *Dendrobium chrysotoxum* | PS2501MT01 |  | EU887923 | FJ216544 | FJ216629 | FJ216489 | FJ216674 |
| Angiosperm | Orchidaceae | *Dendrobium chrysotoxum* | PS2501MT02 |  | EU887924 | FJ216576 | FJ216664 | FJ216508 |  |
| Angiosperm | Orchidaceae | *Dendrobium chrysotoxum* | PS2501MT03 |  | EU887925 | FJ216582 | FJ216671 | FJ216514 | FJ216699 |
| Angiosperm | Orchidaceae | *Dendrobium crepidatum* | PS2517MT01 |  | EU887935 | FJ216562 | FJ216648 | FJ216500 | FJ216685 |
| Angiosperm | Orchidaceae | *Dendrobium crepidatum* | PS2517MT02 |  | FJ216488 | FJ216584 | FJ216673 | FJ216516 |  |
| Angiosperm | Orchidaceae | *Dendrobium crystallinum* | PS2519MT01 |  | FJ216476 | FJ216564 | FJ216650 | EU553953 | EU553970 |
| Angiosperm | Orchidaceae | *Dendrobium denneanum* | PS0758MT01 |  |  |  |  |  | GQ435641 |
| Angiosperm | Orchidaceae | *Dendrobium devonianum* | PS2520MT01 |  | FJ216478 | FJ216566 | FJ216652 | EU553954 | EU553971 |
| Angiosperm | Orchidaceae | *Dendrobium fimbriatum* | PS2507MT01 |  | FJ216468 | FJ216550 |  | FJ216492 | FJ216677 |
| Angiosperm | Orchidaceae | *Dendrobium gratiosissimum* | PS2513MT01 |  | FJ216472 | FJ216557 | FJ216643 | FJ216497 | FJ216682 |
| Angiosperm | Orchidaceae | *Dendrobium hainanense* | PS2526MT01 |  | EU887943 | FJ216579 | FJ216667 | FJ216510 | FJ216695 |
| Angiosperm | Orchidaceae | *Dendrobium loddigesii* | PS1748MT02 |  | EU887940 | FJ216573 | FJ216661 | EU553959 | EU553976 |
| Angiosperm | Orchidaceae | *Dendrobium nobile* | PS0766MT04 |  | FJ216482 | FJ216570 |  | EU553957 | EU553974 |
| Angiosperm | Orchidaceae | *Dendrobium nobile* | PS0766MT05 |  | EU887941 | FJ216577 | FJ216665 | FJ216509 | FJ216693 |
| Angiosperm | Orchidaceae | *Dendrobium nobile* | PS0766MT06 |  | EU887942 | FJ216583 | FJ216672 | FJ216515 | FJ216700 |
| Angiosperm | Orchidaceae | *Dendrobium officinale* | PS2521MT01 |  | FJ216479 | FJ216567 | FJ216653 | EU553955 | EU553972 |
| Angiosperm | Orchidaceae | *Dendrobium pendulum* | PS2511MT01 |  | EU887933 | FJ216555 | FJ216641 | EU553948 | EU553965 |
| Angiosperm | Orchidaceae | *Dendrobium primulinum* | PS2518MT01 |  | EU887936 | FJ216563 |  | EU553952 | EU553969 |
| Angiosperm | Orchidaceae | *Dendrobium primulinum* | PS2518MT02 |  | EU887937 |  | FJ216669 | FJ216512 | FJ216697 |
| Angiosperm | Orchidaceae | *Dendrobium trigonopus* | PS2506MT01 |  |  | FJ216549 | FJ216634 | EU553946 | EU553963 |
| Angiosperm | Orchidaceae | *Dendrobium wardianum* | PS2509MT01 |  | EU887930 | FJ216553 |  | FJ216495 | FJ216680 |
| Angiosperm | Orchidaceae | *Dendrobium wardianum* | PS2509MT02 |  | EU887931 | FJ216581 | FJ216670 | FJ216513 | FJ216698 |
| Angiosperm | Orchidaceae | *Dendrobium williamsonii* | PS2503MT01 |  | EU887927 | FJ216547 | FJ216632 | EU553944 | EU553961 |
| Angiosperm | Orobanchaceae | *Cistanche deserticola* | PS1687MT01 | GQ434850 | GQ435468 |  |  |  |  |
| Angiosperm | Orobanchaceae | *Cistanche deserticola* | PS1687MT04 |  | GQ435469 |  |  |  |  |
| Angiosperm | Orobanchaceae | *Cistanche tubulosa* | PS0811MT01 | GQ434562 |  |  |  |  |  |
| Angiosperm | Orobanchaceae | *Cistanche tubulosa* | PS0811MT02 | GQ434563 | GQ435163 |  |  | GQ436046 |  |
| Angiosperm | Orobanchaceae | *Cistanche tubulosa* | PS0811MT03 |  | GQ435164 |  |  |  |  |
| Angiosperm | Oxalidaceae | *Averrhoa carambola* | PS0162MT03 | GQ434324 | GQ434943 | GQ436319 |  |  |  |
| Angiosperm | Oxalidaceae | *Oxalis griffithii* | PS0160MT01 | GQ434323 |  |  |  | GQ435918 |  |
| Angiosperm | Palmae | *Areca catechu* | PS1671MT02 |  |  | GQ436757 |  |  | GQ435846 |
| Angiosperm | Palmae | *Areca triandra* | PS1674MT02 |  |  | GQ436758 |  |  | GQ435847 |
| Angiosperm | Palmae | *Cocos nucifera* | PS1676MT01 |  | GQ435464 | GQ436759 | GQ434293 |  | GQ435848 |
| Angiosperm | Palmae | *Elaeis gunieensis* | PS1677MT01 |  |  | GQ436760 |  |  | GQ435849 |
| Angiosperm | Palmae | *Trachycarpus fortunei* | PS1679MT02 |  | GQ435465 | GQ436761 |  |  |  |
| Angiosperm | Papaveraceae | *Argemone mexicana* | PS1566MT02 | GQ434813 | GQ435427 | GQ436730 | GQ434281 | GQ436247 | GQ435818 |
| Angiosperm | Papaveraceae | *Chelidonium majus* | PS1562MT01 | GQ434812 | GQ435426 |  | GQ434280 | GQ436246 | GQ435817 |
| Angiosperm | Papaveraceae | *Corydalis edulis* | PS1571MT01 |  |  | GQ436731 |  |  | GQ435819 |
| Angiosperm | Papaveraceae | *Papaver somniferum* | PS1682MT01 | GQ434847 |  |  |  |  |  |
| Angiosperm | Passifloraceae | *Passiflora edulis* | PS1482MT01 |  |  | GQ436714 |  |  |  |
| Angiosperm | Phytolaccaceae | *Phytolacca americana* | PS1278MT01 | GQ434716 | GQ435330 |  | GQ434239 |  | GQ435753 |
| Angiosperm | Phytolaccaceae | *Phytolacca americana* | PS1278MT02 |  | GQ435331 |  | GQ434240 |  |  |
| Angiosperm | Phytolaccaceae | *Phytolacca americana* | PS1278MT05 | GQ434717 | GQ435332 | GQ436650 |  |  | GQ435754 |
| Angiosperm | Piperaceae | *Piper longum* | PS0445MT01 | GQ434421 | GQ435021 |  |  |  | GQ435570 |
| Angiosperm | Piperaceae | *Piper longum* | PS0445MT02 | GQ434422 | GQ435022 |  |  |  |  |
| Angiosperm | Piperaceae | *Piper nigrum* | PS0449MT02 |  | GQ435023 | GQ436391 |  |  |  |
| Angiosperm | Plantaginaceae | *Plantago asiatica* | PS0099MT02 |  | GQ434939 | GQ436317 | GQ434075 |  |  |
| Angiosperm | Plantaginaceae | *Plantago depressa* | PS0101MT02 |  | GQ434940 | GQ436318 | GQ434076 |  |  |
| Angiosperm | Poaceae | *Coix lacryma* var. *mayuen* | PS0423MT01 |  | GQ435012 | GQ436384 |  |  | GQ435568 |
| Angiosperm | Poaceae | *Coix lacryma* var. *mayuen* | PS0423MT02 | GQ434411 | GQ435013 |  | GQ434094 |  |  |
| Angiosperm | Poaceae | *Coix lacryma* var. *mayuen* | PS0423MT03 | GQ434412 | GQ435014 |  | GQ434095 |  |  |
| Angiosperm | Poaceae | *Cymbopogon citratus* | PS0422MT01 |  | GQ435011 | GQ436383 | GQ434093 |  | GQ435567 |
| Angiosperm | Poaceae | *Cymbopogon distans* | PS0425MT01 | GQ434413 |  |  |  |  |  |
| Angiosperm | Poaceae | *Eleusine indica* | PS0419MT01 |  | GQ435009 | GQ436380 | GQ434091 |  | GQ435564 |
| Angiosperm | Poaceae | *Imperata cylindrica* | PS0406MT01 |  | GQ435007 |  |  |  |  |
| Angiosperm | Poaceae | *Imperata cylindrica* | PS0406MT02 | GQ434410 |  |  |  |  |  |
| Angiosperm | Poaceae | *Lophatherum gracile* | PS0410MT02 |  |  |  | GQ434088 |  | GQ435562 |
| Angiosperm | Poaceae | *Oryza sativa* | PS0411MT02 |  | GQ435008 | GQ436379 | GQ434089 |  | GQ435563 |
| Angiosperm | Poaceae | *Phragmites australis* | PS0418MT02 |  |  |  | GQ434090 |  |  |
| Angiosperm | Polygonaceae | *Fagopyrum dibotrys* | PS0797MT01 |  | EU554044 | EU554009 | GQ434135 | FJ503019 | FJ503039 |
| Angiosperm | Polygonaceae | *Fallopia aubertii* | PS0799MT02 |  | EU554045 | EU554010 |  | FJ503020 |  |
| Angiosperm | Polygonaceae | *Fallopia multiflora* | PS0789MT01 |  | EU554047 | EU554014 |  | FJ503024 | FJ503042 |
| Angiosperm | Polygonaceae | *Fallopia multiflora* | PS0789MT03 |  |  | GQ436507 | GQ434132 |  |  |
| Angiosperm | Polygonaceae | *Oxyria digyna* | PS0803MT01 | GQ434561 | GQ435162 |  |  | GQ436045 |  |
| Angiosperm | Polygonaceae | *Oxyria digyna* | PS0803MT02 |  | EU554051 | EU554018 | GQ434136 | FJ503028 | GQ435647 |
| Angiosperm | Polygonaceae | *Persicaria lapathifolia* | PS1683MT01 | GQ434848 | FJ503036 | EU554022 | GQ434294 | FJ503032 | FJ503046 |
| Angiosperm | Polygonaceae | *Persicaria orientalis* | PS0790MT01 | GQ434558 | FJ503035 | EU554013 | GQ434133 | FJ503023 | FJ503041 |
| Angiosperm | Polygonaceae | *Persicaria tinctoria* | PS2901MT01 | GQ434856 | EU554055 | EU554023 |  | FJ503033 |  |
| Angiosperm | Polygonaceae | *Polygonum aviculare* | PS0785MT02 |  | FJ503034 | EU554012 |  | FJ503022 |  |
| Angiosperm | Polygonaceae | *Polygonum bistorta* | PS0801MT01 | GQ434560 | EU554046 | EU554011 |  | FJ503021 | FJ503040 |
| Angiosperm | Polygonaceae | *Polygonum chinense* | PS0794MT01 |  | GQ435161 |  |  |  |  |
| Angiosperm | Polygonaceae | *Polygonum chinense* | PS0794MT02 | GQ434559 | EU554053 | EU554020 | GQ434134 | FJ503030 |  |
| Angiosperm | Polygonaceae | *Polygonum plebeium* | PS0806MT01 |  | EU554052 | EU554019 |  | FJ503029 |  |
| Angiosperm | Polygonaceae | *Reynoutria japonica* | PS0792MT02 |  | EU554048 | EU554015 |  | FJ503025 |  |
| Angiosperm | Polygonaceae | *Rheum officinale* | PS2902MT02 |  |  |  |  |  | FJ503045 |
| Angiosperm | Polygonaceae | *Rheum officinale* | PS2902MT03 | GQ434857 | GQ435476 | GQ436765 | GQ434295 | GQ436268 |  |
| Angiosperm | Polygonaceae | *Rheum officinale* | PS2902MT04 |  |  |  |  |  | GQ435854 |
| Angiosperm | Polygonaceae | *Rheum officinale* | PS2902MT05 |  |  |  |  |  | GQ435855 |
| Angiosperm | Polygonaceae | *Rheum officinale* | PS2902MT08 | GQ434858 | GQ435477 | GQ436766 | GQ434296 | GQ436269 |  |
| Angiosperm | Polygonaceae | *Rheum officinale* | PS2902MT09 | GQ434859 | GQ435478 | GQ436767 | GQ434297 | GQ436270 |  |
| Angiosperm | Polygonaceae | *Rheum palmatum* | PS2903MT01 | GQ434860 | EU554042 | EU554007 | GQ434298 | FJ503017 | FJ503037 |
| Angiosperm | Polygonaceae | *Rheum palmatum* | PS2903MT02 | GQ434861 | GQ435479 | GQ436768 | GQ434299 | GQ436271 | GQ435856 |
| Angiosperm | Polygonaceae | *Rheum rhaponticum* | PS0804MT02 |  | EU554049 | EU554016 | GQ434137 | FJ503026 | FJ503043 |
| Angiosperm | Polygonaceae | *Rheum tanguticum* | PS2904MT01 |  |  |  |  |  | FJ503038 |
| Angiosperm | Polygonaceae | *Rheum tanguticum* | PS2904MT03 | GQ434862 | GQ435480 | GQ436769 | GQ434300 | GQ436272 | GQ435857 |
| Angiosperm | Polygonaceae | *Rheum tanguticum* | PS2904MT07 | GQ434863 | GQ435481 | GQ436770 | GQ434301 | GQ436273 | GQ435858 |
| Angiosperm | Polygonaceae | *Rheum tanguticum* | PS2904MT09 | GQ434864 | GQ435482 | GQ436771 | GQ434302 | GQ436274 |  |
| Angiosperm | Polygonaceae | *Rumex acetosa* | PS2905MT01 | GQ434865 | EU554050 | EU554017 | GQ434303 | FJ503027 | FJ503044 |
| Angiosperm | Polygonaceae | *Rumex japonicum* | PS0807MT01 |  | EU554054 | EU554021 | GQ434138 | FJ503031 |  |
| Angiosperm | Pontederiaceae | *Eichhornia crassipes* | PS1578MT01 |  | GQ435428 |  |  |  |  |
| Angiosperm | Portulacaceae | *Portulaca oleracea* | PS0875MT01 | GQ434589 |  | GQ436528 | GQ434149 | GQ436060 |  |
| Angiosperm | Portulacaceae | *Talinum paniculatum* | PS0876MT02 | GQ434590 |  | GQ436529 | GQ434150 | GQ436061 | GQ435657 |
| Angiosperm | Primulaceae | *Lysimachia christinae* | PS0089MT01 |  | GQ434938 |  |  |  | GQ435510 |
| Angiosperm | Primulaceae | *Lysimachia christinae* | PS0089MT03 |  |  |  |  |  | GQ435511 |
| Angiosperm | Punicaceae | *Punica granatum* | PS1295MT01 |  | GQ435338 | GQ436656 |  | GQ436186 |  |
| Angiosperm | Ranunculaceae | *Cimicifuga dahurica* | PS0929MT02 | GQ434611 |  |  |  |  | GQ435674 |
| Angiosperm | Ranunculaceae | *Cimicifuga foetida* | PS0925MT01 | GQ434609 |  | GQ436536 |  | GQ436076 | GQ435672 |
| Angiosperm | Ranunculaceae | *Cimicifuga foetida* | PS0925MT02 | GQ434610 | GQ435213 |  |  | GQ436077 | GQ435673 |
| Angiosperm | Ranunculaceae | *Clematis hexapetala* | PS0921MT02 | GQ434606 | GQ435208 |  |  | GQ436073 | GQ435669 |
| Angiosperm | Ranunculaceae | *Clematis montana* | PS0955MT01 | GQ434613 |  | GQ436539 |  | GQ436082 |  |
| Angiosperm | Ranunculaceae | *Coptis chinensis* | PS0915MT01 |  | GQ435206 |  |  | GQ436071 | GQ435667 |
| Angiosperm | Ranunculaceae | *Coptis chinensis* | PS0915MT02 | GQ434605 | GQ435207 |  |  | GQ436072 | GQ435668 |
| Angiosperm | Ranunculaceae | *Coptis deltoidea* | PS0924MT01 | GQ434608 | GQ435210 |  |  | GQ436074 | GQ435670 |
| Angiosperm | Ranunculaceae | *Coptis deltoidea* | PS0924MT02 |  | GQ435211 |  |  | GQ436075 |  |
| Angiosperm | Ranunculaceae | *Coptis deltoidea* | PS0924MT03 |  | GQ435212 |  |  |  | GQ435671 |
| Angiosperm | Ranunculaceae | *Paeonia anomala* | PS0906MT01 | GQ434602 | GQ435203 |  | GQ434162 | GQ436069 |  |
| Angiosperm | Ranunculaceae | *Paeonia anomala* | PS0906MT02 | GQ434603 | GQ435204 |  |  | GQ436070 |  |
| Angiosperm | Ranunculaceae | *Paeonia lactiflora* | PS0905MT01 | GQ434600 | GQ435202 |  | GQ434160 |  |  |
| Angiosperm | Ranunculaceae | *Paeonia lactiflora* | PS0905MT03 | GQ434601 |  | GQ436534 | GQ434161 |  |  |
| Angiosperm | Ranunculaceae | *Paeonia ostii* | PS0913MT02 | GQ434604 | GQ435205 |  |  |  |  |
| Angiosperm | Ranunculaceae | *Paeonia suffruticosa* | PS0922MT01 | GQ434607 | GQ435209 | GQ436535 |  |  |  |
| Angiosperm | Ranunculaceae | *Pulsatilla chinensis* | PS0899MT02 | GQ434599 | GQ435201 |  |  | GQ436068 | GQ435666 |
| Angiosperm | Ranunculaceae | *Ranunculus ternatus* | PS0934MT01 | GQ434612 |  |  |  | GQ436078 |  |
| Angiosperm | Rhamnaceae | *Ziziphus jujuba* | PS1331MT01 | GQ434735 |  | GQ436666 | GQ434248 | GQ436193 | GQ435762 |
| Angiosperm | Rhamnaceae | *Ziziphus jujuba* | PS1331MT02 | GQ434736 | GQ435353 | GQ436667 |  | GQ436194 | GQ435763 |
| Angiosperm | Rhamnaceae | *Ziziphus jujuba* var. *spinosa* | PS1339MT02 | GQ434737 |  | GQ436668 |  | GQ436195 | GQ435764 |
| Angiosperm | Rosaceae | *Agrimonia pilosa* var. *pilosa* | PS1087MT01 | GQ434652 | GQ435254 |  |  |  |  |
| Angiosperm | Rosaceae | *Agrimonia pilosa* var. *pilosa* | PS1087MT03 |  | GQ435255 | GQ436581 | GQ434190 |  | GQ435704 |
| Angiosperm | Rosaceae | *Amygdalus davidiana* var. *davidiana* | PS1109MT01 |  | GQ435261 | GQ436590 | GQ434198 |  |  |
| Angiosperm | Rosaceae | *Amygdalus davidiana* var. *davidiana* | PS1109MT02 |  | GQ435262 | GQ436591 | GQ434199 | GQ436118 | GQ435710 |
| Angiosperm | Rosaceae | *Amygdalus davidiana* var. *davidiana* | PS1109MT03 | GQ434656 | GQ435263 |  |  | GQ436119 |  |
| Angiosperm | Rosaceae | *Amygdalus persica* | PS1117MT01 |  | GQ435268 | GQ436597 | GQ434205 | GQ436127 |  |
| Angiosperm | Rosaceae | *Amygdalus persica* | PS1117MT02 |  | GQ435269 | GQ436598 |  | GQ436128 |  |
| Angiosperm | Rosaceae | *Amygdalus persica* var. *persica f. duplex* | PS1065MT01 |  | GQ435243 | GQ436565 | GQ434177 | GQ436096 | GQ435698 |
| Angiosperm | Rosaceae | *Amygdalus persica* var. *scleropersica* | PS1089MT01 | GQ434653 | GQ435256 |  |  |  |  |
| Angiosperm | Rosaceae | *Amygdalus triloba* | PS1127MT02 |  | GQ435277 | GQ436609 | GQ434213 | GQ436138 |  |
| Angiosperm | Rosaceae | *Armeniaca mume* var. *mume* | PS1097MT01 |  |  | GQ436584 |  | GQ436111 | GQ435707 |
| Angiosperm | Rosaceae | *Armeniaca sibirica* | PS1121MT03 | GQ434659 | GQ435274 | GQ436603 |  | GQ436133 | GQ435714 |
| Angiosperm | Rosaceae | *Armeniaca sibirica* | PS1121MT04 |  |  | GQ436604 | GQ434210 | GQ436134 |  |
| Angiosperm | Rosaceae | *Armeniaca vulgaris* | PS1123MT01 |  |  | GQ436605 | GQ434211 | GQ436135 |  |
| Angiosperm | Rosaceae | *Armeniaca vulgaris* | PS1123MT02 | GQ434660 |  | GQ436606 |  |  |  |
| Angiosperm | Rosaceae | *Cerasus glandulosa* | PS1088MT01 |  |  | GQ436582 | GQ434191 | GQ436109 | GQ435705 |
| Angiosperm | Rosaceae | *Cerasus japonica* | PS1128MT01 | GQ434661 |  |  |  | GQ436139 | GQ435716 |
| Angiosperm | Rosaceae | *Cerasus pseudocerasus* | PS1126MT01 |  | GQ435276 | GQ436608 |  | GQ436137 |  |
| Angiosperm | Rosaceae | *Cerasus tomentosa* | PS1092MT01 |  |  | GQ436583 | GQ434192 | GQ436110 |  |
| Angiosperm | Rosaceae | *Chaenomeles speciosa* | PS1101MT01 | GQ434655 |  | GQ436587 | GQ434195 | GQ436114 | GQ435708 |
| Angiosperm | Rosaceae | *Crataegus pinnatifida* var. *major* | PS1108MT01 |  |  | GQ436589 |  | GQ436117 | GQ435709 |
| Angiosperm | Rosaceae | *Crataegus pinnatifida* var. *pinnatifida* | PS1111MT01 |  |  | GQ436592 | GQ434200 | GQ436120 |  |
| Angiosperm | Rosaceae | *Crataegus pinnatifida* var. *pinnatifida* | PS1111MT02 |  |  |  |  | GQ436121 | GQ435711 |
| Angiosperm | Rosaceae | *Docynia delavayi* | PS1131MT01 |  |  | GQ436610 | GQ434214 |  | GQ435717 |
| Angiosperm | Rosaceae | *Duchesnea indica* var. *indica* | PS1113MT01 | GQ434657 |  |  |  | GQ436123 |  |
| Angiosperm | Rosaceae | *Duchesnea indica* var. *indica* | PS1113MT03 |  | GQ435265 |  | GQ434202 | GQ436124 |  |
| Angiosperm | Rosaceae | *Eriobotrya japonica* | PS1102MT02 |  |  |  | GQ434196 |  |  |
| Angiosperm | Rosaceae | *Geum aleppicum* | PS1116MT01 |  | GQ435266 | GQ436595 | GQ434203 | GQ436125 | GQ435712 |
| Angiosperm | Rosaceae | *Geum aleppicum* | PS1116MT02 |  | GQ435267 | GQ436596 | GQ434204 | GQ436126 |  |
| Angiosperm | Rosaceae | *Malus baccata* var. *baccata* | PS1107MT02 |  |  |  |  | GQ436116 |  |
| Angiosperm | Rosaceae | *Photinia serratifolia* var. *serratifolia* | PS1115MT01 |  |  | GQ436594 |  |  |  |
| Angiosperm | Rosaceae | *Potentilla ancistrifolia* var. *ancistrifolia* | PS1080MT01 |  | GQ435252 | GQ436577 | GQ434187 | GQ436106 |  |
| Angiosperm | Rosaceae | *Potentilla ancistrifolia* var. *ancistrifolia* | PS1080MT02 |  | GQ435253 | GQ436578 | GQ434188 | GQ436107 |  |
| Angiosperm | Rosaceae | *Potentilla chinensis* | PS1120MT01 |  | GQ435272 | GQ436601 | GQ434208 | GQ436131 |  |
| Angiosperm | Rosaceae | *Potentilla chinensis* | PS1120MT02 |  | GQ435273 | GQ436602 | GQ434209 | GQ436132 |  |
| Angiosperm | Rosaceae | *Potentilla discolor* | PS1079MT01 | GQ434650 | GQ435251 | GQ436576 |  | GQ436105 |  |
| Angiosperm | Rosaceae | *Potentilla fruticosa* | PS1084MT01 |  |  | GQ436580 | GQ434189 | GQ436108 | GQ435703 |
| Angiosperm | Rosaceae | *Potentilla multicaulis* | PS1077MT01 |  | GQ435250 | GQ436574 | GQ434186 | GQ436104 |  |
| Angiosperm | Rosaceae | *Potentilla nivea* | PS1124MT01 |  | GQ435275 | GQ436607 | GQ434212 | GQ436136 | GQ435715 |
| Angiosperm | Rosaceae | *Potentilla supina* | PS1067MT01 |  |  | GQ436567 | GQ434179 | GQ436098 | GQ435700 |
| Angiosperm | Rosaceae | *Potentilla supina* | PS1067MT02 |  | GQ435244 | GQ436568 | GQ434180 | GQ436099 |  |
| Angiosperm | Rosaceae | *Prinsepia sinensis* | PS1075MT01 |  | GQ435248 | GQ436572 | GQ434184 | GQ436102 |  |
| Angiosperm | Rosaceae | *Prinsepia uniflora* var. *uniflora* | PS1118MT01 | GQ434658 | GQ435270 | GQ436599 | GQ434206 | GQ436129 | GQ435713 |
| Angiosperm | Rosaceae | *Prunus cerasifera f. atropurpurea* | PS1134MT01 |  |  | GQ436612 | GQ434216 | GQ436141 |  |
| Angiosperm | Rosaceae | *Rosa bella* | PS1098MT01 |  | GQ435258 | GQ436585 | GQ434193 | GQ436112 |  |
| Angiosperm | Rosaceae | *Rosa laevigata* | PS1085MT01 | GQ434651 |  |  |  |  |  |
| Angiosperm | Rosaceae | *Rosa multifiora* var. *cathayensis* | PS1076MT02 |  | GQ435249 | GQ436573 | GQ434185 | GQ436103 |  |
| Angiosperm | Rosaceae | *Rosa rugosa* | PS1094MT02 | GQ434654 |  |  |  |  | GQ435706 |
| Angiosperm | Rosaceae | *Rubus chingii* | PS1135MT01 | GQ434662 |  |  |  |  |  |
| Angiosperm | Rosaceae | *Rubus crataegifolius* | PS1112MT01 |  | GQ435264 | GQ436593 | GQ434201 | GQ436122 |  |
| Angiosperm | Rosaceae | *Rubus parvifolius* | PS1093MT01 |  | GQ435257 |  |  |  |  |
| Angiosperm | Rosaceae | *Sanguisorba officinalis* | PS1073MT01 | GQ434648 | GQ435245 |  |  |  |  |
| Angiosperm | Rosaceae | *Sanguisorba officinalis* | PS1073MT04 | GQ434649 |  | GQ436569 | GQ434181 |  |  |
| Angiosperm | Rosaceae | *Sanguisorba officinalis* | PS1073MT06 |  | GQ435246 | GQ436570 | GQ434182 | GQ436100 | GQ435701 |
| Angiosperm | Rosaceae | *Sanguisorba officinalis* | PS1073MT07 |  | GQ435247 | GQ436571 | GQ434183 | GQ436101 | GQ435702 |
| Angiosperm | Rosaceae | *Sanguisorba officinalis* var. *longifolia* | PS1066MT01 |  |  | GQ436566 | GQ434178 | GQ436097 | GQ435699 |
| Angiosperm | Rosaceae | *Sorbaria kirilowii* | PS1082MT01 |  |  | GQ436579 |  |  |  |
| Angiosperm | Rosaceae | *Sorbaria sorbifolia* | PS1132MT01 |  | GQ435278 | GQ436611 | GQ434215 | GQ436140 | GQ435718 |
| Angiosperm | Rosaceae | *Spiraea mongolica* var. *mongolica* | PS1099MT01 |  | GQ435259 | GQ436586 | GQ434194 | GQ436113 |  |
| Angiosperm | Rosaceae | *Spiraea pubescens* var. *pubescens* | PS1119MT01 |  | GQ435271 | GQ436600 | GQ434207 | GQ436130 |  |
| Angiosperm | Rosaceae | *Spiraea trilobata* var. *trilobata* | PS1104MT01 |  | GQ435260 | GQ436588 | GQ434197 | GQ436115 |  |
| Angiosperm | Rubiaceae | *Adina pilulifera* | PS1059MT01 |  | GQ435240 |  | GQ434176 | GQ436095 | GQ435697 |
| Angiosperm | Rubiaceae | *Coffea arabica* | PS1061MT01 |  | GQ435241 |  |  |  |  |
| Angiosperm | Rubiaceae | *Gardenia jasminoides* | PS1062MT01 |  |  | GQ436563 |  |  |  |
| Angiosperm | Rubiaceae | *Gardenia jasminoides* | PS1062MT02 | GQ434646 | GQ435242 |  |  |  |  |
| Angiosperm | Rubiaceae | *Gardenia jasminoides* | PS1062MT07 | GQ434647 |  | GQ436564 |  |  |  |
| Angiosperm | Rubiaceae | *Gardenia jasminoides* var. *grandiflora* | PS1058MT01 |  | GQ435239 |  |  | GQ436094 |  |
| Angiosperm | Rubiaceae | *Hedyotis diffusa* | PS1034MT01 |  | GQ435231 |  |  | GQ436091 |  |
| Angiosperm | Rubiaceae | *Hedyotis hedyotidea* | PS1053MT01 | GQ434645 |  |  |  |  | GQ435696 |
| Angiosperm | Rubiaceae | *Morinda officinalis* | PS1033MT01 | GQ434633 |  |  |  |  |  |
| Angiosperm | Rubiaceae | *Morinda officinalis* | PS1033MT02 | GQ434634 |  | GQ436556 | GQ434175 |  |  |
| Angiosperm | Rubiaceae | *Paederia scandens* | PS1048MT01 | GQ434643 |  |  |  |  |  |
| Angiosperm | Rubiaceae | *Psychotria rubra* | PS1049MT01 | GQ434644 |  | GQ436561 |  |  |  |
| Angiosperm | Rubiaceae | *Rubia cordifolia* | PS1055MT03 |  | GQ435238 | GQ436562 |  |  |  |
| Angiosperm | Rubiaceae | *Serissa serissoides* | PS1035MT03 | GQ434635 | GQ435232 |  |  | GQ436092 |  |
| Angiosperm | Rubiaceae | *Uncaria hirsuta* | PS1043MT01 | GQ434641 | GQ435237 |  |  |  |  |
| Angiosperm | Rubiaceae | *Uncaria macrophylla* | PS1038MT01 | GQ434636 |  |  |  |  |  |
| Angiosperm | Rubiaceae | *Uncaria macrophylla* | PS1038MT03 | GQ434637 | GQ435234 | GQ436558 |  |  |  |
| Angiosperm | Rubiaceae | *Uncaria macrophylla* | PS1038MT04 | GQ434638 | GQ435235 | GQ436559 |  |  |  |
| Angiosperm | Rubiaceae | *Uncaria sessilifructus* | PS1041MT02 | GQ434640 |  |  |  |  |  |
| Angiosperm | Rubiaceae | *Uncaria sinensis* | PS1039MT01 | GQ434639 | GQ435236 | GQ436560 |  | GQ436093 |  |
| Angiosperm | Rubiaceae | *Wendlandia pendula* | PS1037MT01 |  | GQ435233 | GQ436557 |  |  |  |
| Angiosperm | Rubiaceae | *Wendlandia tinctoria* | PS1044MT01 | GQ434642 |  |  |  |  |  |
| Angiosperm | Rutaceae | *Citrus grandis* | PS1600MT04 | GQ434825 | GQ435442 |  |  | GQ436255 | GQ435827 |
| Angiosperm | Rutaceae | *Citrus grandis* | PS1600MT05 | GQ434826 | GQ435443 |  |  | GQ436256 |  |
| Angiosperm | Rutaceae | *Citrus grandis* | PS1600MT06 | GQ434827 | GQ435444 |  |  | GQ436257 |  |
| Angiosperm | Rutaceae | *Citrus grandis* | PS1600MT08 |  | GQ435445 |  |  |  |  |
| Angiosperm | Rutaceae | *Citrus grandis* | PS1600MT09 |  | GQ435446 |  |  |  |  |
| Angiosperm | Rutaceae | *Citrus grandis* var. *tomentosa* | PS1590MT01 | GQ434817 | GQ435433 |  | GQ434283 |  | GQ435822 |
| Angiosperm | Rutaceae | *Citrus grandis* var. *tomentosa* | PS1590MT02 | GQ434818 | GQ435434 | GQ436734 |  |  |  |
| Angiosperm | Rutaceae | *Citrus limonum* | PS1609MT01 | GQ434832 | GQ435450 | GQ436744 |  |  |  |
| Angiosperm | Rutaceae | *Citrus medica* | PS1616MT01 | GQ434836 |  | GQ436749 |  |  | GQ435836 |
| Angiosperm | Rutaceae | *Citrus medica* | PS1616MT02 | GQ434837 | GQ435453 |  |  | GQ436263 |  |
| Angiosperm | Rutaceae | *Citrus medica* | PS1616MT03 |  | GQ435454 | GQ436750 |  |  |  |
| Angiosperm | Rutaceae | *Citrus medica* var. *sarcodactylis* | PS1595MT01 | GQ434821 | GQ435437 |  |  | GQ436251 |  |
| Angiosperm | Rutaceae | *Citrus reticulata* | PS1596MT01 | GQ434822 | GQ435438 | GQ436736 |  | GQ436252 |  |
| Angiosperm | Rutaceae | *Citrus sinensis* | PS1613MT01 | GQ434833 |  | GQ436746 |  | GQ436261 |  |
| Angiosperm | Rutaceae | *Clausena excavata* | PS1603MT04 |  |  | GQ436740 |  |  | GQ435828 |
| Angiosperm | Rutaceae | *Dictamnus dasycarpus* | PS1591MT02 | GQ434819 | GQ435435 |  |  |  |  |
| Angiosperm | Rutaceae | *Evodia lepta* | PS1610MT01 |  |  | GQ436745 |  |  |  |
| Angiosperm | Rutaceae | *Evodia rutaecarpa* | PS1614MT01 | GQ434834 |  | GQ436747 | GQ434286 | GQ436262 | GQ435834 |
| Angiosperm | Rutaceae | *Evodia rutaecarpa* | PS1614MT02 |  | GQ435451 |  |  |  |  |
| Angiosperm | Rutaceae | *Glycosmis pentaphylla* | PS1615MT01 | GQ434835 | GQ435452 | GQ436748 |  |  | GQ435835 |
| Angiosperm | Rutaceae | *Murraya exotica* | PS1605MT01 | GQ434828 | GQ435447 |  |  | GQ436258 | GQ435829 |
| Angiosperm | Rutaceae | *Murraya exotica* | PS1605MT02 | GQ434829 |  | GQ436741 | GQ434284 | GQ436259 | GQ435830 |
| Angiosperm | Rutaceae | *Murraya exotica* | PS1605MT03 |  | GQ435448 | GQ436742 |  |  |  |
| Angiosperm | Rutaceae | *Murraya exotica* | PS1605MT04 | GQ434830 |  |  |  |  | GQ435831 |
| Angiosperm | Rutaceae | *Murraya exotica* | PS1605MT05 | GQ434831 | GQ435449 | GQ436743 | GQ434285 |  | GQ435832 |
| Angiosperm | Rutaceae | *Phellodendron amurense* | PS1598MT01 |  | GQ435440 | GQ436738 |  | GQ436253 | GQ435825 |
| Angiosperm | Rutaceae | *Phellodendron chinense* | PS1593MT01 | GQ434820 | GQ435436 | GQ436735 |  | GQ436250 | GQ435823 |
| Angiosperm | Rutaceae | *Poncirus trifoliata* | PS1597MT01 | GQ434823 | GQ435439 | GQ436737 |  |  | GQ435824 |
| Angiosperm | Rutaceae | *Zanthoxylum armatum* | PS1619MT01 | GQ434838 | GQ435455 | GQ436751 |  |  |  |
| Angiosperm | Rutaceae | *Zanthoxylum bungeanum* | PS1599MT01 | GQ434824 | GQ435441 | GQ436739 |  | GQ436254 | GQ435826 |
| Angiosperm | Rutaceae | *Zanthoxylum nitidum* | PS1608MT01 |  |  |  |  |  | GQ435833 |
| Angiosperm | Rutaceae | *Zanthoxylum nitidum* | PS1608MT02 |  |  |  |  | GQ436260 |  |
| Angiosperm | Santalaceae | *Santalum album* | PS1377MT01 | GQ434756 | GQ435374 | GQ436681 |  |  |  |
| Angiosperm | Santalaceae | *Santalum album* | PS1377MT03 | GQ434757 | GQ435375 |  | GQ434250 |  | GQ435772 |
| Angiosperm | Santalaceae | *Santalum album* | PS1377MT04 | GQ434758 | GQ435376 | GQ436682 |  |  | GQ435773 |
| Angiosperm | Santalaceae | *Santalum album* | PS1377MT05 |  | GQ435377 |  |  |  |  |
| Angiosperm | Sapindaceae | *Dimocarpus longan* | PS1439MT01 | GQ434768 | GQ435391 |  |  |  | GQ435783 |
| Angiosperm | Sapindaceae | *Dimocarpus longan* | PS1439MT03 | GQ434769 |  |  |  |  | GQ435784 |
| Angiosperm | Sapindaceae | *Dimocarpus longan* | PS1439MT04 | GQ434770 |  | GQ436699 |  |  |  |
| Angiosperm | Sapindaceae | *Litchi chinensis* | PS1438MT03 |  | GQ435390 |  |  |  |  |
| Angiosperm | Saururaceae | *Houttuynia cordata* | PS1180MT01 | GQ434677 | GQ435292 |  |  |  |  |
| Angiosperm | Saururaceae | *Houttuynia cordata* | PS1180MT02 | GQ434678 |  |  | GQ434224 | GQ436154 | GQ435733 |
| Angiosperm | Saururaceae | *Saururus chinensis* | PS1182MT01 |  | GQ435293 |  | GQ434225 | GQ436155 | GQ435734 |
| Angiosperm | Saururaceae | *Saururus chinensis* | PS1182MT02 |  |  |  | GQ434226 |  | GQ435735 |
| Angiosperm | Saxifragaceae | *Dichroa febrifuga* | PS0850MT01 | GQ434580 |  | GQ436520 | GQ434143 | GQ436056 | GQ435650 |
| Angiosperm | Scrophulariaceae | *Rehmannia glutinosa* | PS1518MT01 | GQ434798 |  | GQ436719 | GQ434277 | GQ436238 | GQ435809 |
| Angiosperm | Scrophulariaceae | *Scrophularia ningpoensis* | PS1528MT01 | GQ434801 |  | GQ436721 |  | GQ436241 | GQ435811 |
| Angiosperm | Scrophulariaceae | *Veronicastrum axillare* | PS1525MT01 | GQ434800 | GQ435421 | GQ436720 |  | GQ436240 | GQ435810 |
| Angiosperm | Scrophulariaceae | *Veronicastrum stenostachyum* | PS1519MT01 | GQ434799 | GQ435420 |  |  | GQ436239 |  |
| Angiosperm | Simaroubaceae | *Brucea javanica* | PS0753MT01 | GQ434550 |  |  | GQ434126 |  | GQ435639 |
| Angiosperm | Simaroubaceae | *Brucea javanica* | PS0753MT02 | GQ434551 | GQ435154 | GQ436503 | GQ434127 |  |  |
| Angiosperm | Simaroubaceae | *Brucea javanica* | PS0753MT05 | GQ434552 |  |  |  |  |  |
| Angiosperm | Simaroubaceae | *Brucea mollis* | PS0752MT01 | GQ434549 | GQ435153 | GQ436502 | GQ434125 |  | GQ435638 |
| Angiosperm | Simaroubaceae | *Picrasma quassioides* | PS0751MT01 | GQ434548 | GQ435152 |  |  |  |  |
| Angiosperm | Solanaceae | *Datura arborea* | PS1147MT01 | GQ434670 |  |  | GQ434219 | GQ436149 | GQ435725 |
| Angiosperm | Solanaceae | *Datura innoxia* | PS1146MT04 | GQ434669 | GQ435286 |  | GQ434218 | GQ436148 | GQ435724 |
| Angiosperm | Solanaceae | *Datura metel* | PS1152MT01 | GQ434671 | GQ435287 |  | GQ434220 | GQ436150 | GQ435726 |
| Angiosperm | Solanaceae | *Datura metel* | PS1152MT02 | GQ434672 |  |  | GQ434221 | GQ436151 | GQ435727 |
| Angiosperm | Solanaceae | *Lycium chinense* | PS1139MT01 |  | GQ435281 | GQ436614 |  | GQ436143 | GQ435720 |
| Angiosperm | Solanaceae | *Physalis alkekengi* var. *franchetii* | PS1141MT01 | GQ434666 | GQ435282 | GQ436615 |  | GQ436144 | GQ435721 |
| Angiosperm | Solanaceae | *Physalis pubescens* | PS1142MT01 |  | GQ435283 | GQ436616 |  | GQ436145 | GQ435722 |
| Angiosperm | Solanaceae | *Solanum laciniatum* | PS1136MT01 | GQ434663 | GQ435279 | GQ436613 | GQ434217 | GQ436142 | GQ435719 |
| Angiosperm | Solanaceae | *Solanum lyratum* | PS1137MT01 | GQ434664 | GQ435280 |  |  |  |  |
| Angiosperm | Solanaceae | *Solanum lyratum* | PS1137MT02 | GQ434665 |  |  |  |  |  |
| Angiosperm | Solanaceae | *Solanum nigrum* var. *nigrum* | PS1144MT01 | GQ434667 | GQ435284 | GQ436617 |  | GQ436146 | GQ435723 |
| Angiosperm | Solanaceae | *Solanum nigrum* var. *nigrum* | PS1144MT02 | GQ434668 | GQ435285 |  |  | GQ436147 |  |
| Angiosperm | Sonneratiaceae | *Duabanga grandiflora* | PS0398MT01 | GQ434408 |  | GQ436376 | GQ434087 |  |  |
| Angiosperm | Sparganiaceae | *Sparganium stoloniferum* | PS0427MT01 | GQ434414 |  | GQ436385 | GQ434096 |  | GQ435569 |
| Angiosperm | Stemonaceae | *Stemona japonica* | PS0019MT01 |  | GQ434875 |  | GQ434037 | GQ435865 | GQ435488 |
| Angiosperm | Stemonaceae | *Stemona japonica* | PS0019MT02 |  |  | GQ436286 | GQ434038 | GQ435866 | GQ435489 |
| Angiosperm | Stemonaceae | *Stemona sessilifolia* | PS0020MT01 |  |  | GQ436287 | GQ434039 | GQ435867 |  |
| Angiosperm | Stemonaceae | *Stemona tuberosa* | PS0018MT02 |  |  |  |  | GQ435861 | GQ435485 |
| Angiosperm | Stemonaceae | *Stemona tuberosa* | PS0018MT03 |  |  | GQ436284 |  | GQ435862 | GQ435486 |
| Angiosperm | Stemonaceae | *Stemona tuberosa* | PS0018MT07 |  |  |  | GQ434035 |  |  |
| Angiosperm | Stemonaceae | *Stemona tuberosa* | PS0018MT08 |  | GQ434873 | GQ436285 | GQ434036 | GQ435863 | GQ435487 |
| Angiosperm | Stemonaceae | *Stemona tuberosa* | PS0018MT09 |  | GQ434874 |  |  | GQ435864 |  |
| Angiosperm | Sterculiaceae | *Scaphium lychnophorum* | PS1443MT01 | GQ434771 | GQ435392 | GQ436700 |  |  |  |
| Angiosperm | Sterculiaceae | *Scaphium wallichii* | PS1452MT01 | GQ434772 | GQ435393 | GQ436702 |  |  | GQ435785 |
| Angiosperm | Sterculiaceae | *Sterculia lychnophora* | PS1449MT01 |  |  | GQ436701 |  |  |  |
| Angiosperm | Tamaricaceae | *Tamarix chinensis* | PS0102MT01 |  | GQ434941 |  |  | GQ435917 |  |
| Angiosperm | Theaceae | *Camellia oleifera* | PS1261MT01 |  | GQ435325 | GQ436646 |  |  |  |
| Angiosperm | Theaceae | *Camellia oleifera* | PS1261MT02 |  | GQ435326 | GQ436647 |  |  |  |
| Angiosperm | Theaceae | *Schima superba* | PS1264MT01 |  | GQ435327 |  | GQ434238 |  |  |
| Angiosperm | Thymelaeaceae | *Aquilaria sinensis* | PS1178MT01 | GQ434674 | GQ435290 | GQ436619 |  |  |  |
| Angiosperm | Thymelaeaceae | *Aquilaria sinensis* | PS1178MT02 | GQ434675 |  | GQ436620 |  |  | GQ435731 |
| Angiosperm | Thymelaeaceae | *Aquilaria sinensis* | PS1178MT06 |  | GQ435291 |  |  |  |  |
| Angiosperm | Thymelaeaceae | *Wikstroemia indica* | PS1179MT01 | GQ434676 |  |  |  |  | GQ435732 |
| Angiosperm | Trilliaceae | *Paris polyphylla* var. *yunnanensis* | PS1637MT02 | GQ434839 | GQ435462 |  | GQ434291 |  |  |
| Angiosperm | Typhaceae | *Typha angustifolia* | PS0420MT02 |  | GQ435010 | GQ436381 |  |  | GQ435565 |
| Angiosperm | Typhaceae | *Typha angustifolia* | PS0420MT03 |  |  | GQ436382 | GQ434092 |  | GQ435566 |
| Angiosperm | Typhaceae | *Typha minima* | PS0743MT01 |  | GQ435150 |  |  |  |  |
| Angiosperm | Urticaceae | *Boehmeria longispica* | PS1025MT01 |  |  | GQ436552 |  |  | GQ435694 |
| Angiosperm | Urticaceae | *Boehmeria nivea* | PS1031MT01 | GQ434631 | GQ435228 | GQ436553 |  |  |  |
| Angiosperm | Urticaceae | *Boehmeria nivea* | PS1031MT05 |  | GQ435229 | GQ436554 |  |  | GQ435695 |
| Angiosperm | Urticaceae | *Boehmeria siamensis* | PS1032MT01 | GQ434632 | GQ435230 | GQ436555 |  |  |  |
| Angiosperm | Urticaceae | *Gonostegia hirta* | PS1024MT02 | GQ434629 |  |  |  |  |  |
| Angiosperm | Urticaceae | *Gonostegia hirta* | PS1024MT03 | GQ434630 |  | GQ436551 |  |  |  |
| Angiosperm | Valerianaceae | *Nardostachys chinensis* | PS0080MT01 |  | GQ434935 | GQ436316 |  | GQ435916 | GQ435509 |
| Angiosperm | Valerianaceae | *Patrinia villosa* | PS0078MT01 |  | GQ434933 |  | GQ434074 |  | GQ435507 |
| Angiosperm | Valerianaceae | *Patrinia villosa* | PS0078MT03 |  | GQ434934 | GQ436315 |  | GQ435915 | GQ435508 |
| Angiosperm | Valerianaceae | *Valeriana jatamansi* | PS0083MT01 |  | GQ434936 |  |  |  |  |
| Angiosperm | Verbenaceae | *Clerodendrum bungei* | PS0853MT01 | GQ434582 |  |  |  |  | GQ435651 |
| Angiosperm | Verbenaceae | *Clerodendrum bungei* | PS0853MT02 | GQ434583 | GQ435182 |  |  |  |  |
| Angiosperm | Verbenaceae | *Clerodendrum crytophyllum* | PS0856MT02 |  | GQ435183 |  |  |  |  |
| Angiosperm | Verbenaceae | *Clerodendrum japonicum* | PS0851MT01 | GQ434581 | GQ435181 | GQ436521 |  |  |  |
| Angiosperm | Verbenaceae | *Duranta erecta* | PS0862MT01 |  | GQ435186 |  | GQ434145 | GQ436057 | GQ435653 |
| Angiosperm | Verbenaceae | *Nyctanthes arbor-tristis* | PS0871MT01 | GQ434588 |  | GQ436527 |  |  | GQ435656 |
| Angiosperm | Verbenaceae | *Verbena officinalis* | PS0865MT01 | GQ434585 | GQ435187 |  | GQ434146 | GQ436058 |  |
| Angiosperm | Verbenaceae | *Verbena officinalis* | PS0865MT03 | GQ434586 | GQ435188 | GQ436523 | GQ434147 |  | GQ435654 |
| Angiosperm | Verbenaceae | *Vitex negundo* var. *cannabifolia* | PS0867MT01 | GQ434587 | GQ435189 | GQ436524 | GQ434148 | GQ436059 | GQ435655 |
| Angiosperm | Verbenaceae | *Vitex negundo* var. *cannabifolia* | PS0867MT02 |  | GQ435190 | GQ436525 |  |  |  |
| Angiosperm | Verbenaceae | *Vitex negundo* var. *negundo* | PS0861MT03 | GQ434584 | GQ435185 | GQ436522 | GQ434144 |  | GQ435652 |
| Angiosperm | Verbenaceae | *Vitex trifolia* | PS0868MT02 |  | GQ435191 | GQ436526 |  |  |  |
| Angiosperm | Verbenaceae | *Vitex trifolia* var. *simplicifolia* | PS0858MT01 |  | GQ435184 |  |  |  |  |
| Angiosperm | Violaceae | *Viola diffusa* | PS0555MT01 | GQ434456 |  | GQ436415 |  |  | GQ435587 |
| Angiosperm | Violaceae | *Viola philippica* | PS0561MT02 | GQ434457 | GQ435054 | GQ436417 |  |  | GQ435589 |
| Angiosperm | Violaceae | *Viola prioantha* | PS0560MT01 |  | GQ435053 | GQ436416 |  |  | GQ435588 |
| Angiosperm | Vitaceae | *Ampelopsis japonica* | PS0989MT01 | GQ434623 |  |  |  | GQ436088 | GQ435688 |
| Angiosperm | Zingiberaceae | *Alpinia galanga* | PS0515MT01 | GQ434440 |  |  |  |  | GQ435584 |
| Angiosperm | Zingiberaceae | *Alpinia galanga* | PS0515MT02 |  | GQ435039 |  |  |  |  |
| Angiosperm | Zingiberaceae | *Alpinia galanga* | PS0515MT03 |  | GQ435040 |  |  |  |  |
| Angiosperm | Zingiberaceae | *Alpinia galanga* | PS0515MT05 | GQ434441 | GQ435041 | GQ436404 |  |  |  |
| Angiosperm | Zingiberaceae | *Alpinia hainanensis* | PS0511MT01 |  |  | GQ436403 | GQ434102 |  |  |
| Angiosperm | Zingiberaceae | *Alpinia hainanensis* | PS0511MT02 | GQ434438 |  |  | GQ434103 |  |  |
| Angiosperm | Zingiberaceae | *Alpinia officinarum* | PS0519MT01 | GQ434443 |  |  |  |  |  |
| Angiosperm | Zingiberaceae | *Alpinia oxyphylla* | PS0533MT03 | GQ434450 |  | GQ436412 |  |  |  |
| Angiosperm | Zingiberaceae | *Alpinia zerumbet* | PS0532MT01 | GQ434449 |  |  |  |  |  |
| Angiosperm | Zingiberaceae | *Alpinia zerumbet* | PS0532MT02 |  | GQ435051 | GQ436411 |  |  |  |
| Angiosperm | Zingiberaceae | *Amomum compactum* | PS0535MT01 |  |  | GQ118665 |  |  |  |
| Angiosperm | Zingiberaceae | *Amomum kravanh* | PS0516MT01 | GQ434442 |  |  |  |  |  |
| Angiosperm | Zingiberaceae | *Amomum longiligulare* | PS0522MT01 | GQ434444 | GQ464983 | GQ118663 |  |  |  |
| Angiosperm | Zingiberaceae | *Amomum villosum* | PS0514MT01 | GQ434439 |  | GQ118662 |  |  |  |
| Angiosperm | Zingiberaceae | *Amomum villosum* var. *xanthioides* | PS0526MT01 | GQ434448 | GQ118659 | GQ118664 |  |  |  |
| Angiosperm | Zingiberaceae | *Curcuma aromatica* | PS0534MT01 |  |  | GQ436413 |  |  |  |
| Angiosperm | Zingiberaceae | *Curcuma aromatica* | PS0534MT03 | GQ434451 |  |  |  |  |  |
| Angiosperm | Zingiberaceae | *Curcuma aromatica* | PS0534MT04 | GQ434452 |  |  |  |  |  |
| Angiosperm | Zingiberaceae | *Curcuma aromatica cv. wenyujin* | PS0531MT05 |  | GQ435050 |  |  |  |  |
| Angiosperm | Zingiberaceae | *Curcuma aromatica cv. wenyujin* | PS0531MT06 |  |  |  | GQ434106 |  |  |
| Angiosperm | Zingiberaceae | *Curcuma kwangsiensis* | PS0520MT01 |  | GQ435042 | GQ436405 | GQ434104 |  |  |
| Angiosperm | Zingiberaceae | *Curcuma kwangsiensis* | PS0520MT02 |  | GQ435043 | GQ436406 |  |  |  |
| Angiosperm | Zingiberaceae | *Curcuma zedoaria* | PS0518MT04 |  |  |  |  |  | GQ435585 |
| Angiosperm | Zingiberaceae | *Hedychium coronarium* | PS0524MT01 | GQ434447 | GQ435046 | GQ436407 |  |  |  |
| Angiosperm | Zingiberaceae | *Kaempferia galanga* | PS0529MT01 |  | GQ435048 | GQ436409 |  |  |  |
| Angiosperm | Zingiberaceae | *Kaempferia galanga* | PS0529MT02 |  | GQ435049 | GQ436410 |  |  |  |
| Angiosperm | Zingiberaceae | *Zingiber mioga* | PS0528MT01 |  | GQ435047 | GQ436408 |  |  |  |
| Angiosperm | Zingiberaceae | *Zingiber officinale* | PS0523MT01 | GQ434445 |  |  |  |  |  |
| Angiosperm | Zingiberaceae | *Zingiber officinale* | PS0523MT02 |  |  |  | GQ434105 |  |  |
| Angiosperm | Zingiberaceae | *Zingiber officinale* | PS0523MT03 | GQ434446 | GQ435044 |  |  |  |  |
| Angiosperm | Zingiberaceae | *Zingiber officinale* | PS0523MT04 |  | GQ435045 |  |  |  |  |
| Angiosperm | Zygophyllaceae | *Tribulus terrester* | PS0490MT04 | GQ434432 |  |  |  |  | GQ435579 |
| Gymnosperm | Araucariaceae | *Araucaria cunninghamii* | PS0988MT01 | GQ434622 |  |  |  |  |  |
| Gymnosperm | Araucariaceae | *Araucaria cunninghamii* | PS0988MT02 |  |  | GQ436545 |  | GQ436087 |  |
| Gymnosperm | Cephalotaxaceae | *Cephalotaxus hainanensis* | PS1185MT02 | GQ434682 | GQ435297 | GQ436624 |  | GQ436159 |  |
| Gymnosperm | Cephalotaxaceae | *Cephalotaxus hainanensis* | PS1185MT06 | GQ434683 | GQ435298 | GQ436625 |  | GQ436160 |  |
| Gymnosperm | Cephalotaxaceae | *Cephalotaxus harringtonia* var. *drupacea* | PS1192MT01 | GQ434686 | GQ435302 | GQ436627 |  | GQ436163 |  |
| Gymnosperm | Cephalotaxaceae | *Cephalotaxus sinensis* var. *sinensis* | PS1183MT05 | GQ434679 | GQ435294 | GQ436621 |  | GQ436156 |  |
| Gymnosperm | Cephalotaxaceae | *Cephalotaxus sinensis* var. *sinensis* | PS1183MT06 | GQ434680 | GQ435295 | GQ436622 |  | GQ436157 |  |
| Gymnosperm | Cephalotaxaceae | *Cephalotaxus sinensis* var. *sinensis* | PS1183MT07 | GQ434681 | GQ435296 | GQ436623 |  | GQ436158 |  |
| Gymnosperm | Cephalotaxaceae | *Cephalotaxus wilsoniana* | PS1188MT01 | GQ434684 | GQ435300 | GQ436626 |  |  |  |
| Gymnosperm | Cupressaceae | *Juniperus rigida* | PS1351MT01 | GQ434741 | GQ435359 |  |  | GQ436200 |  |
| Gymnosperm | Cupressaceae | *Platycladus orientalis* | PS0074MT04 |  | GQ434931 |  |  | GQ435914 |  |
| Gymnosperm | Cupressaceae | *Sabina chinensis* var. *chinensis cv. kaizuca* | PS0075MT01 | GQ434316 | GQ434932 |  |  |  |  |
| Gymnosperm | Cycadaceae | *Cycas micholitzii* | PS1374MT01 | GQ434754 | GQ435372 | GQ436680 |  | GQ436206 |  |
| Gymnosperm | Cycadaceae | *Cycas pectinata* | PS1373MT01 | GQ434753 | GQ435371 |  |  |  |  |
| Gymnosperm | Ginkgoaceae | *Ginkgo biloba* | PS1561MT03 | GQ434811 |  | GQ436729 |  | GQ436245 |  |
| Gymnosperm | Gnetaceae | *Gnetum montanum* | PS0894MT01 | GQ434597 | GQ435199 | GQ436533 |  |  |  |
| Gymnosperm | Pinaceae | *Cedrus deodara* | PS1360MT01 |  | GQ435365 | GQ436676 |  | GQ436205 |  |
| Gymnosperm | Pinaceae | *Pinus armandii* var. *armandii* | PS1355MT01 | GQ434742 | GQ435360 |  |  | GQ436201 |  |
| Gymnosperm | Pinaceae | *Pinus armandii* var. *armandii* | PS1355MT02 | GQ434743 | GQ435361 | GQ436674 |  | GQ436202 |  |
| Gymnosperm | Pinaceae | *Pinus bungeana* | PS1348MT01 | GQ434738 | GQ435356 | GQ436672 |  | GQ436198 |  |
| Gymnosperm | Pinaceae | *Pinus bungeana* | PS1348MT02 | GQ434739 | GQ435357 |  |  |  |  |
| Gymnosperm | Pinaceae | *Pinus griffithii* | PS1357MT01 | GQ434745 | GQ435363 | GQ436675 |  | GQ436203 |  |
| Gymnosperm | Pinaceae | *Pinus massoniana* var. *massoniana* | PS1356MT01 | GQ434744 | GQ435362 |  |  |  |  |
| Gymnosperm | Pinaceae | *Pinus ponderosa* | PS1359MT01 | GQ434746 | GQ435364 |  |  | GQ436204 |  |
| Gymnosperm | Pinaceae | *Pinus strobus* | PS1350MT01 | GQ434740 | GQ435358 | GQ436673 |  | GQ436199 |  |
| Gymnosperm | Pinaceae | *Pinus tabuliformis* var. *tabuliformis* | PS1361MT06 | GQ434747 |  | GQ436677 |  |  |  |
| Gymnosperm | Podocarpaceae | *Podocarpus imbricatus* | PS0830MT01 | GQ434567 | GQ435172 |  |  |  |  |
| Gymnosperm | Podocarpaceae | *Podocarpus macrophyllus* | PS0831MT02 | GQ434568 |  |  |  | GQ436050 |  |
| Gymnosperm | Taxaceae | *Amentotaxus yunnanensis* | PS0442MT01 | GQ434420 | GQ435020 |  |  | GQ435989 |  |
| Gymnosperm | Taxaceae | *Taxus canadensis* | PS0435MT01 | GQ434417 | GQ435017 | GQ436388 |  | GQ435986 |  |
| Gymnosperm | Taxaceae | *Taxus chinensis* | PS1196MT01 | GQ434687 |  |  |  | GQ436164 |  |
| Gymnosperm | Taxaceae | *Taxus chinensis* var. *mairei* | PS0438MT01 | GQ434419 | GQ435019 | GQ436390 |  | GQ435988 |  |
| Gymnosperm | Taxaceae | *Taxus cuspidata* | PS0431MT01 | GQ434416 | GQ435016 | GQ436387 |  | GQ435985 |  |
| Gymnosperm | Taxaceae | *Taxus cuspidata* var. *nana* | PS0429MT01 | GQ434415 | GQ435015 | GQ436386 |  | GQ435984 |  |
| Gymnosperm | Taxaceae | *Taxus fuana* | PS0436MT01 | GQ434418 | GQ435018 | GQ436389 |  | GQ435987 |  |
| Gymnosperm | Taxaceae | *Torreya californica* | PS1186MT01 |  | GQ435299 |  |  | GQ436161 |  |
| Gymnosperm | Taxaceae | *Torreya grandis* | PS1744MT01 | GQ434852 | GQ435473 | GQ436763 |  |  | GQ435853 |
| Gymnosperm | Taxaceae | *Torreya yunnanensis* | PS1190MT01 | GQ434685 | GQ435301 |  |  | GQ436162 |  |
| Gymnosperm | Taxodiaceae | *Taxodium distichum* | PS1272MT01 | GQ434715 | GQ435329 | GQ436649 |  | GQ436183 |  |
| Fern | Athyriaceae | *Athyrium multidentatum* | PS0375MT01 | GQ434403 |  |  |  |  |  |
| Fern | Blechnaceae | *Blechnum orientale* | PS0374MT01 |  | GQ434997 |  |  |  |  |
| Fern | Blechnaceae | *Blechnum orientale* | PS0374MT02 | GQ434402 | GQ434998 |  |  |  |  |
| Fern | Davalliaceae | *Humata repens* | PS0370MT01 | GQ434401 |  |  |  |  |  |
| Fern | Dicksoniaceae | *Cibotium barometz* | PS0085MT01 |  | GQ434937 |  |  |  |  |
| Fern | Drynariaceae | *Drynaria bonii* | PS0391MT01 |  | GQ435000 |  |  |  |  |
| Fern | Drynariaceae | *Drynaria sinica* | PS0393MT01 |  | GQ435001 |  |  |  |  |
| Fern | Drynariaceae | *Pseudodrynaria coronans* | PS0392MT01 | GQ434404 |  |  |  |  |  |
| Fern | Ephedraceae | *Ephedra equisetina* | PS0848MT01 | GQ434578 | GQ435179 | GQ436518 |  |  |  |
| Fern | Ephedraceae | *Ephedra equisetina* | PS0848MT02 | GQ434579 | GQ435180 | GQ436519 |  |  |  |
| Fern | Ephedraceae | *Ephedra sinica* | PS0847MT02 |  | GQ435178 | GQ436517 |  |  |  |
| Fern | Lycopodiaceae | *Lycopodium japonicum* | PS1298MT01 |  | GQ435342 |  |  |  |  |
| Fern | Lycopodiaceae | *Lycopodium serratum* | PS1297MT01 |  | GQ435341 |  |  |  |  |
| Fern | Lycopodiaceae | *Palhinhaea cernua* | PS1296MT01 |  | GQ435339 |  |  |  |  |
| Fern | Lycopodiaceae | *Palhinhaea cernua* | PS1296MT02 |  | GQ435340 |  |  | GQ436187 |  |
| Fern | Lygodiaceae | *Lygodium japonicum* | PS0395MT01 |  |  |  |  |  | GQ435560 |
| Fern | Lygodiaceae | *Lygodium japonicum* | PS0395MT04 | GQ434405 | GQ435002 |  |  |  |  |
| Fern | Lygodiaceae | *Lygodium japonicum* | PS0395MT05 | GQ434406 | GQ435003 |  |  |  |  |
| Fern | Polypodiaceae | *Phymatodes cuspidata* | PS0378MT01 |  | GQ434999 |  |  | GQ435980 |  |
| Fern | Pteridaceae | *Pteris multifida* | PS0361MT03 |  | GQ434994 |  |  |  |  |
| Fern | Pteridaceae | *Pteris semipinnata* | PS0719MT01 |  | GQ435141 |  |  |  |  |
| Fern | Selaginellaceae | *Selaginella moellendorffii* | PS0727MT01 | GQ434545 | GQ435143 |  |  |  |  |
| Fern | Selaginellaceae | *Selaginella uncinata* | PS0726MT01 | GQ434544 |  |  |  |  |  |
| Fern | Thelypteridaceae | *Cyclosorus parasiticus* | PS0387MT01 |  |  | GQ436375 |  |  |  |
| Fungi | Polyporaceae | *Polyporus umbellatus* | PS0338MT01 | GQ434389 |  |  |  |  |  |
