# Supplementary material for: Validation of the ITS2 Region as a Novel DNA Barcode for Identifying Medicinal Plant Species
Source: PLoS One. 2010 Jan 7;5(1):e8613. doi: 10.1371/journal.pone.0008613 (PMC2799520; doi:10.1371/journal.pone.0008613)
Supplement: Table S6 — Samples for determining the ability of the ITS2 barcode to identify species and accession numbers in GenBank. (5.25 MB DOC) [file pone.0008613.s009.doc]

**Table S6.** Samples for determining the ability of the ITS2 barcode to identify species and accession numbers in GenBank.

| **Phylum** | **Family** | **Species** | **GenBank accession** |
| --- | --- | --- | --- |
| Angiosperm | Acanthaceae | *Baphicacanthus cusia* | AY863064 |
| Angiosperm | Acanthaceae | *Dicliptera suberecta* | AF289763 |
| Angiosperm | Acanthaceae | *Hygrophila brasiliensis* | EF214418 |
| Angiosperm | Acanthaceae | *Hygrophila costata* | EF214419 |
| Angiosperm | Acanthaceae | *Hygrophila schulli* | EU489060 |
| Angiosperm | Acanthaceae | *Rhinacanthus gracilis* | AF289766 |
| Angiosperm | Acanthaceae | *Ruellia acutangula* | EF214436 |
| Angiosperm | Acanthaceae | *Ruellia acutangula* | EF214437 |
| Angiosperm | Acanthaceae | *Ruellia adenocalyx* | EF214438 |
| Angiosperm | Acanthaceae | *Ruellia amoena* | EF214441 |
| Angiosperm | Acanthaceae | *Ruellia amplexicaulis* | EF214442 |
| Angiosperm | Acanthaceae | *Ruellia ansericollis* | EF214447 |
| Angiosperm | Acanthaceae | *Ruellia asperula* | EF214448 |
| Angiosperm | Acanthaceae | *Ruellia beyrichiana* | EF214449 |
| Angiosperm | Acanthaceae | *Ruellia biolleyi* | EF214450 |
| Angiosperm | Acanthaceae | *Ruellia biolleyi* | EF214451 |
| Angiosperm | Acanthaceae | *Ruellia blechioides* | EU812547 |
| Angiosperm | Acanthaceae | *Ruellia blechum* | EU812549 |
| Angiosperm | Acanthaceae | *Ruellia blechum* | EU812550 |
| Angiosperm | Acanthaceae | *Ruellia bourgaei* | EF214452 |
| Angiosperm | Acanthaceae | *Ruellia bourgaei* | EF214453 |
| Angiosperm | Acanthaceae | *Ruellia breedlovei* | EF214454 |
| Angiosperm | Acanthaceae | *Ruellia brevifolia* | EF214455 |
| Angiosperm | Acanthaceae | *Ruellia brevifolia* | EF214457 |
| Angiosperm | Acanthaceae | *Ruellia brittoniana* | EF214458 |
| Angiosperm | Acanthaceae | *Ruellia bulbifera* | EF214459 |
| Angiosperm | Acanthaceae | *Ruellia caroliniensis* | EF214460 |
| Angiosperm | Acanthaceae | *Ruellia chartacea* | EF214461 |
| Angiosperm | Acanthaceae | *Ruellia chartacea* | EF214462 |
| Angiosperm | Acanthaceae | *Ruellia ciliatiflora* | EF214463 |
| Angiosperm | Acanthaceae | *Ruellia conzattii* | EF214467 |
| Angiosperm | Acanthaceae | *Ruellia cordata* | EF214468 |
| Angiosperm | Acanthaceae | *Ruellia costaricensis* | EU812551 |
| Angiosperm | Acanthaceae | *Ruellia costata* | EF214469 |
| Angiosperm | Acanthaceae | *Ruellia cyanea* | EF214470 |
| Angiosperm | Acanthaceae | *Ruellia densa* | EF214471 |
| Angiosperm | Acanthaceae | *Ruellia detonsa* | EF214472 |
| Angiosperm | Acanthaceae | *Ruellia devosiana* | EF214473 |
| Angiosperm | Acanthaceae | *Ruellia devosiana* | EF214474 |
| Angiosperm | Acanthaceae | *Ruellia discifolia* | EF214475 |
| Angiosperm | Acanthaceae | *Ruellia dissidens* | EF214476 |
| Angiosperm | Acanthaceae | *Ruellia donnell-smithii* | EF214477 |
| Angiosperm | Acanthaceae | *Ruellia donnell-smithii* | EF214478 |
| Angiosperm | Acanthaceae | *Ruellia drummondiana* | EF214479 |
| Angiosperm | Acanthaceae | *Ruellia edwardsae* | EF214481 |
| Angiosperm | Acanthaceae | *Ruellia elegans* | EF214482 |
| Angiosperm | Acanthaceae | *Ruellia elegans* | EF214484 |
| Angiosperm | Acanthaceae | *Ruellia eriocalyx* | EF214485 |
| Angiosperm | Acanthaceae | *Ruellia erythropus* | EF214486 |
| Angiosperm | Acanthaceae | *Ruellia exserta* | EF214490 |
| Angiosperm | Acanthaceae | *Ruellia floribunda* | EF214492 |
| Angiosperm | Acanthaceae | *Ruellia foetida* | EF214493 |
| Angiosperm | Acanthaceae | *Ruellia fulgida* | EF214495 |
| Angiosperm | Acanthaceae | *Ruellia fulgida* | EU431001 |
| Angiosperm | Acanthaceae | *Ruellia galeottii* | EF214497 |
| Angiosperm | Acanthaceae | *Ruellia geminiflora* | EF214498 |
| Angiosperm | Acanthaceae | *Ruellia gracilis* | EF214499 |
| Angiosperm | Acanthaceae | *Ruellia grantii* | EF214500 |
| Angiosperm | Acanthaceae | *Ruellia hirsutoglandulosa* | EF214505 |
| Angiosperm | Acanthaceae | *Ruellia hookeriana* | EF214506 |
| Angiosperm | Acanthaceae | *Ruellia humboldtiana* | EF214507 |
| Angiosperm | Acanthaceae | *Ruellia humilis* | EF214508 |
| Angiosperm | Acanthaceae | *Ruellia incompta* | EU431002 |
| Angiosperm | Acanthaceae | *Ruellia inflata* | EF214512 |
| Angiosperm | Acanthaceae | *Ruellia jaliscana* | EF214517 |
| Angiosperm | Acanthaceae | *Ruellia jussieuoides* | EF214519 |
| Angiosperm | Acanthaceae | *Ruellia lactea* | EF214520 |
| Angiosperm | Acanthaceae | *Ruellia latibracteata* | EU812552 |
| Angiosperm | Acanthaceae | *Ruellia latibracteata* | EU812553 |
| Angiosperm | Acanthaceae | *Ruellia latisepala* | EF214521 |
| Angiosperm | Acanthaceae | *Ruellia leucantha* | EF214522 |
| Angiosperm | Acanthaceae | *Ruellia linearibracteolata* | EF214523 |
| Angiosperm | Acanthaceae | *Ruellia longifilamentosa* | EF214525 |
| Angiosperm | Acanthaceae | *Ruellia longipedunculata* | EF214526 |
| Angiosperm | Acanthaceae | *Ruellia macrantha* | EF214530 |
| Angiosperm | Acanthaceae | *Ruellia macrophylla* | EF214531 |
| Angiosperm | Acanthaceae | *Ruellia macrosolen* | EF214527 |
| Angiosperm | Acanthaceae | *Ruellia malacophylla* | EF214532 |
| Angiosperm | Acanthaceae | *Ruellia matagalpae* | EF214533 |
| Angiosperm | Acanthaceae | *Ruellia maya* | EF214536 |
| Angiosperm | Acanthaceae | *Ruellia mcvaughii* | EF214537 |
| Angiosperm | Acanthaceae | *Ruellia megachlamys* | EF214539 |
| Angiosperm | Acanthaceae | *Ruellia menthifolia* | EF214540 |
| Angiosperm | Acanthaceae | *Ruellia menthoides* | EF214541 |
| Angiosperm | Acanthaceae | *Ruellia morongii* | EF214543 |
| Angiosperm | Acanthaceae | *Ruellia multifolia* | EF214544 |
| Angiosperm | Acanthaceae | *Ruellia nitida* | EF214545 |
| Angiosperm | Acanthaceae | *Ruellia nobilis* | EF214546 |
| Angiosperm | Acanthaceae | *Ruellia novogaliciana* | EF214547 |
| Angiosperm | Acanthaceae | *Ruellia paniculata* | EF214552 |
| Angiosperm | Acanthaceae | *Ruellia patula* | EF214554 |
| Angiosperm | Acanthaceae | *Ruellia patula* | EF214555 |
| Angiosperm | Acanthaceae | *Ruellia pearcei* | EF214557 |
| Angiosperm | Acanthaceae | *Ruellia petiolaris* | EF214559 |
| Angiosperm | Acanthaceae | *Ruellia petiolaris* | EF214560 |
| Angiosperm | Acanthaceae | *Ruellia pringlei* | EF214563 |
| Angiosperm | Acanthaceae | *Ruellia puri* | EF214565 |
| Angiosperm | Acanthaceae | *Ruellia rubra* | EF214569 |
| Angiosperm | Acanthaceae | *Ruellia sanguinea* | EF214573 |
| Angiosperm | Acanthaceae | *Ruellia saulensis* | EF214575 |
| Angiosperm | Acanthaceae | *Ruellia standleyi* | EF214579 |
| Angiosperm | Acanthaceae | *Ruellia standleyi* | EF214580 |
| Angiosperm | Acanthaceae | *Ruellia stemonacanthoides* | EF214584 |
| Angiosperm | Acanthaceae | *Ruellia steyermarkii* | EF214581 |
| Angiosperm | Acanthaceae | *Ruellia steyermarkii* | EF214582 |
| Angiosperm | Acanthaceae | *Ruellia strepens* | EF214585 |
| Angiosperm | Acanthaceae | *Ruellia subsessilis* | EF214586 |
| Angiosperm | Acanthaceae | *Ruellia tarapotana* | EF214587 |
| Angiosperm | Acanthaceae | *Ruellia tomentosa* | EF214589 |
| Angiosperm | Acanthaceae | *Ruellia tuberosa* | EF214592 |
| Angiosperm | Acanthaceae | *Ruellia tuberosa* | EF214593 |
| Angiosperm | Acanthaceae | *Ruellia tubiflora* | EF214590 |
| Angiosperm | Acanthaceae | *Ruellia tuxtlensis* | EF214596 |
| Angiosperm | Acanthaceae | *Ruellia villosa* | EF214599 |
| Angiosperm | Aceraceae | *Acer albopurpurascens* | DQ238471 |
| Angiosperm | Aceraceae | *Acer albopurpurascens* | DQ238472 |
| Angiosperm | Aceraceae | *Acer barbinerve* | AJ634569 |
| Angiosperm | Aceraceae | *Acer barbinerve* | AJ634573 |
| Angiosperm | Aceraceae | *Acer buergerianum* | AF020381 |
| Angiosperm | Aceraceae | *Acer buergerianum* | AY605465 |
| Angiosperm | Aceraceae | *Acer buergerianum* | AY605466 |
| Angiosperm | Aceraceae | *Acer buergerianum* subsp. *buergerianum* | AJ634588 |
| Angiosperm | Aceraceae | *Acer buergerianum* subsp. *buergerianum* | AM265514 |
| Angiosperm | Aceraceae | *Acer buergerianum* subsp. *buergerianum* | DQ238462 |
| Angiosperm | Aceraceae | *Acer buergerianum* subsp. *buergerianum* | DQ238464 |
| Angiosperm | Aceraceae | *Acer buergerianum* subsp. *buergerianum* | DQ238465 |
| Angiosperm | Aceraceae | *Acer buergerianum* subsp. *buergerianum* | DQ238466 |
| Angiosperm | Aceraceae | *Acer caesium* subsp. *caesium* | AY605293 |
| Angiosperm | Aceraceae | *Acer caesium* subsp. *caesium* | AY605294 |
| Angiosperm | Aceraceae | *Acer caesium* subsp. *caesium* | DQ366115 |
| Angiosperm | Aceraceae | *Acer caesium* subsp. *caesium* | DQ366117 |
| Angiosperm | Aceraceae | *Acer caesium* subsp. *giraldii* | DQ366119 |
| Angiosperm | Aceraceae | *Acer caesium* subsp. *giraldii* | DQ366121 |
| Angiosperm | Aceraceae | *Acer campbellii* subsp. *campbellii* | AY605411 |
| Angiosperm | Aceraceae | *Acer campbellii* subsp. *flabellatum* | AY605417 |
| Angiosperm | Aceraceae | *Acer campbellii* subsp. *flabellatum* | AY605419 |
| Angiosperm | Aceraceae | *Acer campbellii* subsp. *flabellatum* | DQ238394 |
| Angiosperm | Aceraceae | *Acer campbellii* subsp. *sinense* | AY605429 |
| Angiosperm | Aceraceae | *Acer campbellii* subsp. *sinense* | AY605430 |
| Angiosperm | Aceraceae | *Acer campestre* | DQ238425 |
| Angiosperm | Aceraceae | *Acer campestre* | DQ238426 |
| Angiosperm | Aceraceae | *Acer campestre* | DQ238427 |
| Angiosperm | Aceraceae | *Acer campestre* | DQ238428 |
| Angiosperm | Aceraceae | *Acer campestre* | DQ238429 |
| Angiosperm | Aceraceae | *Acer campestre* | DQ238430 |
| Angiosperm | Aceraceae | *Acer campestre* | DQ238431 |
| Angiosperm | Aceraceae | *Acer campestre* | DQ238432 |
| Angiosperm | Aceraceae | *Acer campestre* | DQ238434 |
| Angiosperm | Aceraceae | *Acer campestre* | DQ238435 |
| Angiosperm | Aceraceae | *Acer cappadocicum* | AY605445 |
| Angiosperm | Aceraceae | *Acer cappadocicum* subsp. *cappadocicum* | AJ634579 |
| Angiosperm | Aceraceae | *Acer cappadocicum* subsp. *cappadocicum* | DQ238437 |
| Angiosperm | Aceraceae | *Acer cappadocicum* subsp. *cappadocicum* | DQ238440 |
| Angiosperm | Aceraceae | *Acer cappadocicum* subsp. *cappadocicum* | DQ238441 |
| Angiosperm | Aceraceae | *Acer cappadocicum* subsp. *cappadocicum* | DQ238443 |
| Angiosperm | Aceraceae | *Acer carpinifolium* | AF020365 |
| Angiosperm | Aceraceae | *Acer carpinifolium* | AY605377 |
| Angiosperm | Aceraceae | *Acer carpinifolium* | AY605378 |
| Angiosperm | Aceraceae | *Acer carpinifolium* | AY605379 |
| Angiosperm | Aceraceae | *Acer carpinifolium* | AY605380 |
| Angiosperm | Aceraceae | *Acer carpinifolium* | AY605381 |
| Angiosperm | Aceraceae | *Acer caudatifolium* | DQ238380 |
| Angiosperm | Aceraceae | *Acer caudatum* subsp. *multiserratum* | AY605433 |
| Angiosperm | Aceraceae | *Acer cinnamomifolium* | DQ238467 |
| Angiosperm | Aceraceae | *Acer cinnamomifolium* | DQ238469 |
| Angiosperm | Aceraceae | *Acer cinnamomifolium* | DQ238470 |
| Angiosperm | Aceraceae | *Acer circinatum* | AY605412 |
| Angiosperm | Aceraceae | *Acer circinatum* | AY605413 |
| Angiosperm | Aceraceae | *Acer cissifolium* | AY605401 |
| Angiosperm | Aceraceae | *Acer cissifolium* | AY605402 |
| Angiosperm | Aceraceae | *Acer crataegifolium* | AF020368 |
| Angiosperm | Aceraceae | *Acer crataegifolium* | AY605391 |
| Angiosperm | Aceraceae | *Acer crataegifolium* | DQ238375 |
| Angiosperm | Aceraceae | *Acer crataegifolium* | DQ238377 |
| Angiosperm | Aceraceae | *Acer crataegifolium* | DQ238378 |
| Angiosperm | Aceraceae | *Acer crataegifolium* | DQ238379 |
| Angiosperm | Aceraceae | *Acer davidii* subsp. *davidii* | DQ238364 |
| Angiosperm | Aceraceae | *Acer davidii* subsp. *davidii* | DQ238367 |
| Angiosperm | Aceraceae | *Acer davidii* subsp. *grosseri* | AY605396 |
| Angiosperm | Aceraceae | *Acer davidii* subsp. *grosseri* | AY605397 |
| Angiosperm | Aceraceae | *Acer diabolicum* | AY605383 |
| Angiosperm | Aceraceae | *Acer diabolicum* | AY605384 |
| Angiosperm | Aceraceae | *Acer erianthum* | AY605414 |
| Angiosperm | Aceraceae | *Acer erianthum* | AY605415 |
| Angiosperm | Aceraceae | *Acer erianthum* | DQ238393 |
| Angiosperm | Aceraceae | *Acer glabrum* | DQ238336 |
| Angiosperm | Aceraceae | *Acer glabrum* | DQ238337 |
| Angiosperm | Aceraceae | *Acer glabrum* | DQ238339 |
| Angiosperm | Aceraceae | *Acer glabrum* | DQ238340 |
| Angiosperm | Aceraceae | *Acer glabrum* | DQ238342 |
| Angiosperm | Aceraceae | *Acer glabrum* | DQ238343 |
| Angiosperm | Aceraceae | *Acer griseum* | AY605467 |
| Angiosperm | Aceraceae | *Acer griseum* | AY605468 |
| Angiosperm | Aceraceae | *Acer griseum* | AY605469 |
| Angiosperm | Aceraceae | *Acer griseum* | AY605470 |
| Angiosperm | Aceraceae | *Acer griseum* | AY605471 |
| Angiosperm | Aceraceae | *Acer griseum* | DQ238479 |
| Angiosperm | Aceraceae | *Acer griseum* | DQ238481 |
| Angiosperm | Aceraceae | *Acer heldreichii* | AY605301 |
| Angiosperm | Aceraceae | *Acer heldreichii* | AY605304 |
| Angiosperm | Aceraceae | *Acer hyrcanum* subsp. *hyrcanum* | AY605305 |
| Angiosperm | Aceraceae | *Acer hyrcanum* subsp. *hyrcanum* | AY605306 |
| Angiosperm | Aceraceae | *Acer hyrcanum* subsp. *hyrcanum* | DQ366129 |
| Angiosperm | Aceraceae | *Acer hyrcanum* subsp. *hyrcanum* | DQ366130 |
| Angiosperm | Aceraceae | *Acer ibericum* | AY605312 |
| Angiosperm | Aceraceae | *Acer ibericum* | AY605313 |
| Angiosperm | Aceraceae | *Acer ibericum* | AY605314 |
| Angiosperm | Aceraceae | *Acer japonicum* | AY605420 |
| Angiosperm | Aceraceae | *Acer japonicum* | AY605421 |
| Angiosperm | Aceraceae | *Acer japonicum* | DQ238395 |
| Angiosperm | Aceraceae | *Acer japonicum* | DQ238397 |
| Angiosperm | Aceraceae | *Acer laevigatum* | DQ238398 |
| Angiosperm | Aceraceae | *Acer laurinum* | AM113541 |
| Angiosperm | Aceraceae | *Acer laurinum* | AM113543 |
| Angiosperm | Aceraceae | *Acer laurinum* | DQ366114 |
| Angiosperm | Aceraceae | *Acer macrophyllum* | AF020367 |
| Angiosperm | Aceraceae | *Acer macrophyllum* | DQ238351 |
| Angiosperm | Aceraceae | *Acer macrophyllum* | DQ238352 |
| Angiosperm | Aceraceae | *Acer macrophyllum* | DQ238353 |
| Angiosperm | Aceraceae | *Acer mandshuricum* | DQ238474 |
| Angiosperm | Aceraceae | *Acer mandshuricum* | DQ238475 |
| Angiosperm | Aceraceae | *Acer mandshuricum* | DQ238476 |
| Angiosperm | Aceraceae | *Acer maximowiczianum* | DQ238483 |
| Angiosperm | Aceraceae | *Acer maximowiczianum* | DQ238484 |
| Angiosperm | Aceraceae | *Acer micranthum* | AF020369 |
| Angiosperm | Aceraceae | *Acer miyabei* | AY605451 |
| Angiosperm | Aceraceae | *Acer miyabei* | AY605452 |
| Angiosperm | Aceraceae | *Acer mono* subsp. *mono* | DQ238445 |
| Angiosperm | Aceraceae | *Acer mono* subsp. *mono* | DQ238446 |
| Angiosperm | Aceraceae | *Acer mono* subsp. *mono* | DQ238447 |
| Angiosperm | Aceraceae | *Acer mono* subsp. *mono* | DQ238448 |
| Angiosperm | Aceraceae | *Acer mono* subsp. *mono* | DQ238449 |
| Angiosperm | Aceraceae | *Acer mono* subsp. *mono* | DQ238450 |
| Angiosperm | Aceraceae | *Acer mono* subsp. *mono* | DQ238452 |
| Angiosperm | Aceraceae | *Acer mono* subsp. *mono* | DQ238453 |
| Angiosperm | Aceraceae | *Acer mono* subsp. *okamotoanum* | DQ238454 |
| Angiosperm | Aceraceae | *Acer mono* subsp. *okamotoanum* | DQ238455 |
| Angiosperm | Aceraceae | *Acer monspessulanum* subsp. *monspessulanum* | AM238373 |
| Angiosperm | Aceraceae | *Acer monspessulanum* subsp. *monspessulanum* | AM238375 |
| Angiosperm | Aceraceae | *Acer monspessulanum* subsp. *monspessulanum* | AM238376 |
| Angiosperm | Aceraceae | *Acer monspessulanum* subsp. *monspessulanum* | AM238378 |
| Angiosperm | Aceraceae | *Acer monspessulanum* subsp. *monspessulanum* | AM238381 |
| Angiosperm | Aceraceae | *Acer monspessulanum* subsp. *monspessulanum* | AM238384 |
| Angiosperm | Aceraceae | *Acer monspessulanum* subsp. *monspessulanum* | AM238388 |
| Angiosperm | Aceraceae | *Acer monspessulanum* subsp. *monspessulanum* | AM238396 |
| Angiosperm | Aceraceae | *Acer monspessulanum* subsp. *monspessulanum* | AM238397 |
| Angiosperm | Aceraceae | *Acer monspessulanum* subsp. *monspessulanum* | DQ366128 |
| Angiosperm | Aceraceae | *Acer negundo* | AY605406 |
| Angiosperm | Aceraceae | *Acer negundo* | AY605407 |
| Angiosperm | Aceraceae | *Acer negundo* | AY605408 |
| Angiosperm | Aceraceae | *Acer negundo* | AY605409 |
| Angiosperm | Aceraceae | *Acer negundo* | DQ238358 |
| Angiosperm | Aceraceae | *Acer negundo* | DQ238359 |
| Angiosperm | Aceraceae | *Acer negundo* | DQ238360 |
| Angiosperm | Aceraceae | *Acer negundo* | DQ238361 |
| Angiosperm | Aceraceae | *Acer negundo* | DQ238362 |
| Angiosperm | Aceraceae | *Acer nipponicum* | AF020380 |
| Angiosperm | Aceraceae | *Acer nipponicum* | DQ366140 |
| Angiosperm | Aceraceae | *Acer nipponicum* | DQ366142 |
| Angiosperm | Aceraceae | *Acer obtusifolium* | AM238328 |
| Angiosperm | Aceraceae | *Acer obtusifolium* | AM238329 |
| Angiosperm | Aceraceae | *Acer obtusifolium* | AM238331 |
| Angiosperm | Aceraceae | *Acer obtusifolium* | AM238332 |
| Angiosperm | Aceraceae | *Acer oliverianum* subsp. *oliverianum* | AY605422 |
| Angiosperm | Aceraceae | *Acer oliverianum* subsp. *oliverianum* | AY605423 |
| Angiosperm | Aceraceae | *Acer oliverianum* subsp. *oliverianum* | AY605424 |
| Angiosperm | Aceraceae | *Acer opalus* subsp. *obtusatum* | AY605336 |
| Angiosperm | Aceraceae | *Acer opalus* subsp. *opalus* | AM238317 |
| Angiosperm | Aceraceae | *Acer opalus* subsp. *opalus* | AM238318 |
| Angiosperm | Aceraceae | *Acer opalus* subsp. *opalus* | AM238320 |
| Angiosperm | Aceraceae | *Acer opalus* subsp. *opalus* | AM238321 |
| Angiosperm | Aceraceae | *Acer opalus* subsp. *opalus* | AY605331 |
| Angiosperm | Aceraceae | *Acer opalus* subsp. *opalus* | AY605332 |
| Angiosperm | Aceraceae | *Acer palmatum* subsp. *amoenum* | DQ238399 |
| Angiosperm | Aceraceae | *Acer palmatum* subsp. *amoenum* | DQ238400 |
| Angiosperm | Aceraceae | *Acer palmatum* subsp. *amoenum* | DQ238401 |
| Angiosperm | Aceraceae | *Acer palmatum* subsp. *palmatum* | AY605425 |
| Angiosperm | Aceraceae | *Acer palmatum* subsp. *palmatum* | AY605426 |
| Angiosperm | Aceraceae | *Acer pentaphyllum* | DQ238477 |
| Angiosperm | Aceraceae | *Acer pentaphyllum* | DQ238478 |
| Angiosperm | Aceraceae | *Acer platanoides* | AY605456 |
| Angiosperm | Aceraceae | *Acer platanoides* | DQ238460 |
| Angiosperm | Aceraceae | *Acer platanoides* | DQ238461 |
| Angiosperm | Aceraceae | *Acer platanoides* | EF494236 |
| Angiosperm | Aceraceae | *Acer pseudoplatanus* | AM238273 |
| Angiosperm | Aceraceae | *Acer pseudoplatanus* | AM238276 |
| Angiosperm | Aceraceae | *Acer pseudoplatanus* | AY605338 |
| Angiosperm | Aceraceae | *Acer pseudoplatanus* | AY605339 |
| Angiosperm | Aceraceae | *Acer pseudoplatanus* | AY605340 |
| Angiosperm | Aceraceae | *Acer pseudoplatanus* | AY605341 |
| Angiosperm | Aceraceae | *Acer pseudoplatanus* | AY605343 |
| Angiosperm | Aceraceae | *Acer pseudoplatanus* | DQ366131 |
| Angiosperm | Aceraceae | *Acer pseudoplatanus* | DQ366132 |
| Angiosperm | Aceraceae | *Acer pseudoplatanus* | DQ366133 |
| Angiosperm | Aceraceae | *Acer pseudosieboldianum* | DQ238402 |
| Angiosperm | Aceraceae | *Acer pseudosieboldianum* subsp. *pseudosieboldianum* | DQ238407 |
| Angiosperm | Aceraceae | *Acer pseudosieboldianum* subsp. *pseudosieboldianum* | DQ238408 |
| Angiosperm | Aceraceae | *Acer pycnanthum* | AM113528 |
| Angiosperm | Aceraceae | *Acer pycnanthum* | AM113529 |
| Angiosperm | Aceraceae | *Acer rubrum* | AJ634580 |
| Angiosperm | Aceraceae | *Acer rubrum* | AJ634585 |
| Angiosperm | Aceraceae | *Acer rubrum* | AJ634586 |
| Angiosperm | Aceraceae | *Acer rubrum* | AY605460 |
| Angiosperm | Aceraceae | *Acer rubrum* | AY605461 |
| Angiosperm | Aceraceae | *Acer rufinerve* | AF020371 |
| Angiosperm | Aceraceae | *Acer saccharinum* | AM113531 |
| Angiosperm | Aceraceae | *Acer saccharinum* | AM113532 |
| Angiosperm | Aceraceae | *Acer saccharinum* | AM113535 |
| Angiosperm | Aceraceae | *Acer saccharinum* | AM113537 |
| Angiosperm | Aceraceae | *Acer saccharinum* | AM113539 |
| Angiosperm | Aceraceae | *Acer saccharum* subsp. *floridanum* | DQ366138 |
| Angiosperm | Aceraceae | *Acer saccharum* subsp. *nigrum* | AM238326 |
| Angiosperm | Aceraceae | *Acer saccharum* subsp. *nigrum* | AY605322 |
| Angiosperm | Aceraceae | *Acer saccharum* subsp. *nigrum* | AY605323 |
| Angiosperm | Aceraceae | *Acer saccharum* subsp. *nigrum* | AY605324 |
| Angiosperm | Aceraceae | *Acer saccharum* subsp. *saccharum* | AY605347 |
| Angiosperm | Aceraceae | *Acer saccharum* subsp. *saccharum* | AY605348 |
| Angiosperm | Aceraceae | *Acer sempervirens* | AY605349 |
| Angiosperm | Aceraceae | *Acer sempervirens* | AY605350 |
| Angiosperm | Aceraceae | *Acer sempervirens* | AY605351 |
| Angiosperm | Aceraceae | *Acer sempervirens* | AY605352 |
| Angiosperm | Aceraceae | *Acer sempervirens* | AY605353 |
| Angiosperm | Aceraceae | *Acer sempervirens* | DQ366122 |
| Angiosperm | Aceraceae | *Acer sempervirens* | DQ366123 |
| Angiosperm | Aceraceae | *Acer shirasawanum* | AY605427 |
| Angiosperm | Aceraceae | *Acer shirasawanum* | DQ238411 |
| Angiosperm | Aceraceae | *Acer spicatum* | AF020378 |
| Angiosperm | Aceraceae | *Acer spicatum* | AJ634575 |
| Angiosperm | Aceraceae | *Acer spicatum* | AJ634576 |
| Angiosperm | Aceraceae | *Acer spicatum* | AJ634577 |
| Angiosperm | Aceraceae | *Acer spicatum* | AJ634578 |
| Angiosperm | Aceraceae | *Acer spicatum* | AY605431 |
| Angiosperm | Aceraceae | *Acer stachyophyllum* subsp. *betulifolium* | AY605373 |
| Angiosperm | Aceraceae | *Acer stachyophyllum* subsp. *betulifolium* | AY605374 |
| Angiosperm | Aceraceae | *Acer stachyophyllum* subsp. *stachyophyllum* | AY605375 |
| Angiosperm | Aceraceae | *Acer stachyophyllum* subsp. *stachyophyllum* | AY605376 |
| Angiosperm | Aceraceae | *Acer sterculiaceum* subsp. *franchetii* | DQ366145 |
| Angiosperm | Aceraceae | *Acer sterculiaceum* subsp. *sterculiaceum* | DQ366144 |
| Angiosperm | Aceraceae | *Acer tataricum* | AM265513 |
| Angiosperm | Aceraceae | *Acer tataricum* subsp. *ginnala* | AY605363 |
| Angiosperm | Aceraceae | *Acer tataricum* subsp. *ginnala* | AY605364 |
| Angiosperm | Aceraceae | *Acer tataricum* subsp. *semenovii* | AY605365 |
| Angiosperm | Aceraceae | *Acer tataricum* subsp. *semenovii* | AY605366 |
| Angiosperm | Aceraceae | *Acer tataricum* subsp. *tataricum* | AY605367 |
| Angiosperm | Aceraceae | *Acer tataricum* subsp. *tataricum* | AY605368 |
| Angiosperm | Aceraceae | *Acer tataricum* subsp. *tataricum* | AY605369 |
| Angiosperm | Aceraceae | *Acer tegmentosum* | DQ366113 |
| Angiosperm | Aceraceae | *Acer trautvetteri* | AM238282 |
| Angiosperm | Aceraceae | *Acer trautvetteri* | AM238283 |
| Angiosperm | Aceraceae | *Acer trautvetteri* | AM238285 |
| Angiosperm | Aceraceae | *Acer trautvetteri* | AY605354 |
| Angiosperm | Aceraceae | *Acer trautvetteri* | AY605355 |
| Angiosperm | Aceraceae | *Acer triflorum* | AY605472 |
| Angiosperm | Aceraceae | *Acer truncatum* | AY605458 |
| Angiosperm | Aceraceae | *Acer truncatum* | AY605459 |
| Angiosperm | Aceraceae | *Acer tschonoskii* | AF020372 |
| Angiosperm | Aceraceae | *Acer tschonoskii* | DQ238389 |
| Angiosperm | Aceraceae | *Acer tschonoskii* subsp. *koreanum* | DQ238381 |
| Angiosperm | Aceraceae | *Acer tschonoskii* subsp. *tschonoskii* | DQ238386 |
| Angiosperm | Aceraceae | *Acer tschonoskii* subsp. *tschonoskii* | DQ238387 |
| Angiosperm | Aceraceae | *Acer tschonoskii* subsp. *tschonoskii* | DQ238388 |
| Angiosperm | Aceraceae | *Acer ukurunduense* | AY605435 |
| Angiosperm | Aceraceae | *Acer velutinum* | AM238286 |
| Angiosperm | Aceraceae | *Acer velutinum* | AM238288 |
| Angiosperm | Aceraceae | *Acer velutinum* | AM238289 |
| Angiosperm | Aceraceae | *Acer velutinum* | AM238290 |
| Angiosperm | Aceraceae | *Acer velutinum* | AM238291 |
| Angiosperm | Aceraceae | *Acer velutinum* | AM238292 |
| Angiosperm | Aceraceae | *Acer velutinum* | AM238293 |
| Angiosperm | Aceraceae | *Acer velutinum* | AM238294 |
| Angiosperm | Aceraceae | *Acer velutinum* | AY605356 |
| Angiosperm | Aceraceae | *Acer velutinum* | AY605357 |
| Angiosperm | Aceraceae | *Acer velutinum* | AY605358 |
| Angiosperm | Aceraceae | *Acer velutinum* | AY605359 |
| Angiosperm | Aceraceae | *Acer velutinum* | AY605360 |
| Angiosperm | Aceraceae | *Acer velutinum* | AY605361 |
| Angiosperm | Aceraceae | *Acer velutinum* | AY605362 |
| Angiosperm | Aceraceae | *Acer velutinum* | DQ366135 |
| Angiosperm | Aceraceae | *Acer velutinum* | DQ366136 |
| Angiosperm | Alismataceae | *Alisma canaliculatum* | DQ468388 |
| Angiosperm | Alismataceae | *Alisma gramineum* | DQ468391 |
| Angiosperm | Alismataceae | *Alisma lanceolatum* | DQ468390 |
| Angiosperm | Alismataceae | *Alisma nanum* | DQ468389 |
| Angiosperm | Alismataceae | *Alisma orientale* | AY519469 |
| Angiosperm | Alismataceae | *Alisma plantago-aquatica* | AY588940 |
| Angiosperm | Amaranthaceae | *Achyranthes bidentata* | DQ813300 |
| Angiosperm | Amaranthaceae | *Alternanthera albotomentosa* var*. ecuadoriensis* | AY255504 |
| Angiosperm | Amaranthaceae | *Alternanthera caracasana* | AY174409 |
| Angiosperm | Amaranthaceae | *Alternanthera costaricensis* | AY174417 |
| Angiosperm | Amaranthaceae | *Alternanthera flavescens* | AY173399 |
| Angiosperm | Amaranthaceae | *Alternanthera laguroides* | AY174419 |
| Angiosperm | Amaranthaceae | *Alternanthera pubiflora* | AY255509 |
| Angiosperm | Amaranthaceae | *Alternanthera sessilis* | AY174415 |
| Angiosperm | Amaranthaceae | *Celosia argentea* | AY174418 |
| Angiosperm | Amaranthaceae | *Cyathula prostrata* | AY174421 |
| Angiosperm | Amaranthaceae | *Gomphrena boliviana* | AY171563 |
| Angiosperm | Amaranthaceae | *Gomphrena celosioides f. aureiflora* | AY171564 |
| Angiosperm | Amaranthaceae | *Gomphrena celosioides f. roseiflora* | AY174404 |
| Angiosperm | Amaranthaceae | *Gomphrena gnaphiotricha* | AY174408 |
| Angiosperm | Amaranthaceae | *Gomphrena mandonii* | AY174416 |
| Angiosperm | Amaranthaceae | *Gomphrena nealleyi* | AY171565 |
| Angiosperm | Amaranthaceae | *Gomphrena sonorae* | AY173405 |
| Angiosperm | Amaryllidaceae | *Crinum asiaticum* | AY139120 |
| Angiosperm | Amaryllidaceae | *Crinum baumii* | AY139121 |
| Angiosperm | Amaryllidaceae | *Crinum bulbispermum* | AY139123 |
| Angiosperm | Amaryllidaceae | *Crinum buphanoides* | AY139124 |
| Angiosperm | Amaryllidaceae | *Crinum carolo-schmidtii* | AY139125 |
| Angiosperm | Amaryllidaceae | *Crinum crassicaule* | AY139126 |
| Angiosperm | Amaryllidaceae | *Crinum cruentum* | AY139127 |
| Angiosperm | Amaryllidaceae | *Crinum defixum* | AY139128 |
| Angiosperm | Amaryllidaceae | *Crinum distichum* | AY139129 |
| Angiosperm | Amaryllidaceae | *Crinum erubescens* | AY139130 |
| Angiosperm | Amaryllidaceae | *Crinum fimbriatulum* | AY139131 |
| Angiosperm | Amaryllidaceae | *Crinum flaccidum* | AY139132 |
| Angiosperm | Amaryllidaceae | *Crinum forbesii* | AY139133 |
| Angiosperm | Amaryllidaceae | *Crinum humile* | AY139134 |
| Angiosperm | Amaryllidaceae | *Crinum jagus* | AY139135 |
| Angiosperm | Amaryllidaceae | *Crinum latifolium* | AY139137 |
| Angiosperm | Amaryllidaceae | *Crinum mauritianum* | AY139139 |
| Angiosperm | Amaryllidaceae | *Crinum moorei* | AY139141 |
| Angiosperm | Amaryllidaceae | *Crinum oliganthum* | AY139142 |
| Angiosperm | Amaryllidaceae | *Crinum pedunculatum* | AY139143 |
| Angiosperm | Amaryllidaceae | *Crinum politifolium* | AY139144 |
| Angiosperm | Amaryllidaceae | *Crinum razafindratsiraea* | AY139145 |
| Angiosperm | Amaryllidaceae | *Crinum subcernuum* | AY139150 |
| Angiosperm | Amaryllidaceae | *Crinum venosum* | AY139146 |
| Angiosperm | Amaryllidaceae | *Crinum yemense* | AY139151 |
| Angiosperm | Anacardiaceae | *Mangifera camptosperma* | AJ890468 |
| Angiosperm | Anacardiaceae | *Mangifera indica* | AJ890466 |
| Angiosperm | Anacardiaceae | *Mangifera indica* | AJ890467 |
| Angiosperm | Anacardiaceae | *Mangifera indica* | AJ890471 |
| Angiosperm | Anacardiaceae | *Mangifera indica* | AJ890472 |
| Angiosperm | Anacardiaceae | *Pistacia chinensis* | EF193079 |
| Angiosperm | Anacardiaceae | *Pistacia integerrima* | EF193081 |
| Angiosperm | Anacardiaceae | *Pistacia khinjuk* | EF193104 |
| Angiosperm | Anacardiaceae | *Pistacia khinjuk* | EF193105 |
| Angiosperm | Anacardiaceae | *Pistacia terebinthus* | EF193086 |
| Angiosperm | Anacardiaceae | *Pistacia vera* | AY677201 |
| Angiosperm | Anacardiaceae | *Rhus aromatica* | AY641493 |
| Angiosperm | Anacardiaceae | *Rhus aromatica* | AY641494 |
| Angiosperm | Anacardiaceae | *Rhus chinensis* var. *roxburghii* | AY641482 |
| Angiosperm | Anacardiaceae | *Rhus choriophylla* | AY641498 |
| Angiosperm | Anacardiaceae | *Rhus coriaria* | AY641485 |
| Angiosperm | Anacardiaceae | *Rhus glabra* | AY641486 |
| Angiosperm | Anacardiaceae | *Rhus integrifolia* | AY641499 |
| Angiosperm | Anacardiaceae | *Rhus kearneyi* | AY641500 |
| Angiosperm | Anacardiaceae | *Rhus lanceolata* | AY641487 |
| Angiosperm | Anacardiaceae | *Rhus michauxii* | AY641488 |
| Angiosperm | Anacardiaceae | *Rhus ovata* | AY641501 |
| Angiosperm | Anacardiaceae | *Rhus ovata* | AY641502 |
| Angiosperm | Anacardiaceae | *Rhus pachyrrhachis* | AY641503 |
| Angiosperm | Anacardiaceae | *Rhus potaninii* | AY641489 |
| Angiosperm | Anacardiaceae | *Rhus punjabensis* | AY641490 |
| Angiosperm | Anacardiaceae | *Rhus rubifolia* | AY641508 |
| Angiosperm | Anacardiaceae | *Rhus sandwicensis* | AY641491 |
| Angiosperm | Anacardiaceae | *Rhus schiedeana* | AY641504 |
| Angiosperm | Anacardiaceae | *Rhus trilobata* | AY641497 |
| Angiosperm | Anacardiaceae | *Rhus typhina* | AY641492 |
| Angiosperm | Anacardiaceae | *Rhus virens* | AY641505 |
| Angiosperm | Anacardiaceae | *Rhus virens* | AY641506 |
| Angiosperm | Anacardiaceae | *Spondias mombin* | AF080064 |
| Angiosperm | Anacardiaceae | *Toxicodendron vernix* | AY641520 |
| Angiosperm | Apiaceae | *Aciphylla aurea* | U72377 |
| Angiosperm | Apiaceae | *Aciphylla dieffenbachii* | U72381 |
| Angiosperm | Apiaceae | *Aciphylla monroi* | U72378 |
| Angiosperm | Apiaceae | *Aciphylla subflabellata* | U72380 |
| Angiosperm | Apiaceae | *Anthriscus sylvestris* | AY548228 |
| Angiosperm | Apiaceae | *Chaerophyllum colensoi* var. *delicatulum* | EU331133 |
| Angiosperm | Apiaceae | *Ferula akitschkensis* | DQ379385 |
| Angiosperm | Apiaceae | *Ferula arida* | DQ379386 |
| Angiosperm | Apiaceae | *Ferula clematidifolia* | DQ379391 |
| Angiosperm | Apiaceae | *Ferula communis* | EU169269 |
| Angiosperm | Apiaceae | *Ferula decurrens* | DQ379393 |
| Angiosperm | Apiaceae | *Ferula diversivittata* | DQ379395 |
| Angiosperm | Apiaceae | *Ferula dshaudshamyr* | DQ379396 |
| Angiosperm | Apiaceae | *Ferula equisetacea* | DQ379398 |
| Angiosperm | Apiaceae | *Ferula eugenii* | DQ379399 |
| Angiosperm | Apiaceae | *Ferula fedoroviorum* | DQ379400 |
| Angiosperm | Apiaceae | *Ferula foliosa* | DQ379405 |
| Angiosperm | Apiaceae | *Ferula gummosa* | DQ379407 |
| Angiosperm | Apiaceae | *Ferula karelinii* | EF560692 |
| Angiosperm | Apiaceae | *Ferula kingdon-wardii* | EU236166 |
| Angiosperm | Apiaceae | *Ferula kokanica* | DQ379412 |
| Angiosperm | Apiaceae | *Ferula koso-poljanskyi* | DQ379414 |
| Angiosperm | Apiaceae | *Ferula leucographa* | DQ379419 |
| Angiosperm | Apiaceae | *Ferula linkii* | DQ379421 |
| Angiosperm | Apiaceae | *Ferula litwinowiana* | DQ379423 |
| Angiosperm | Apiaceae | *Ferula loscosii* | DQ379424 |
| Angiosperm | Apiaceae | *Ferula loscosii* | DQ379425 |
| Angiosperm | Apiaceae | *Ferula microloba* | DQ379427 |
| Angiosperm | Apiaceae | *Ferula mogoltavica* | DQ379429 |
| Angiosperm | Apiaceae | *Ferula neapolitana* | DQ379431 |
| Angiosperm | Apiaceae | *Ferula nuratavica* | DQ379433 |
| Angiosperm | Apiaceae | *Ferula olgae* | DQ379434 |
| Angiosperm | Apiaceae | *Ferula olivacea* | EF560691 |
| Angiosperm | Apiaceae | *Ferula olivacea* | EU169270 |
| Angiosperm | Apiaceae | *Ferula oopoda* | DQ379435 |
| Angiosperm | Apiaceae | *Ferula penninervis* | DQ379439 |
| Angiosperm | Apiaceae | *Ferula pseudooreoselinum* | DQ379443 |
| Angiosperm | Apiaceae | *Ferula schtschurowskiana* | DQ379446 |
| Angiosperm | Apiaceae | *Ferula sibirica* | DQ379447 |
| Angiosperm | Apiaceae | *Ferula tenuisecta* | AF077890 |
| Angiosperm | Apiaceae | *Ferula teterrima* | DQ379452 |
| Angiosperm | Apiaceae | *Ferula tingitana* | DQ379453 |
| Angiosperm | Apiaceae | *Ferula tuberifera* | DQ379454 |
| Angiosperm | Apiaceae | *Ferula undulata* | DQ379456 |
| Angiosperm | Apiaceae | *Ferula urceolata* | DQ379457 |
| Angiosperm | Apiaceae | *Ferula violacea* | AF077891 |
| Angiosperm | Apiaceae | *Ferula xeromorpha* | DQ379458 |
| Angiosperm | Apiaceae | *Ferulago galbanifera* | AF077889 |
| Angiosperm | Apiaceae | *Gingidia algens* | U72366 |
| Angiosperm | Apiaceae | *Gingidia baxterae* | U72365 |
| Angiosperm | Apiaceae | *Gingidia decipiens* | U72368 |
| Angiosperm | Apiaceae | *Gingidia enysii* | U72364 |
| Angiosperm | Apiaceae | *Gingidia flabellata* | U72369 |
| Angiosperm | Apiaceae | *Gingidia harveyana* | U72370 |
| Angiosperm | Apiaceae | *Gingidia montana* | U72363 |
| Angiosperm | Apiaceae | *Gingidia trifoliolata* | U72367 |
| Angiosperm | Apiaceae | *Osmorhiza aristata* | AF453954 |
| Angiosperm | Apiaceae | *Osmorhiza aristata* | AY548217 |
| Angiosperm | Apiaceae | *Osmorhiza berteroi* | AF453962 |
| Angiosperm | Apiaceae | *Osmorhiza berteroi* | AF453964 |
| Angiosperm | Apiaceae | *Osmorhiza claytonii* | AF453967 |
| Angiosperm | Apiaceae | *Osmorhiza claytonii* | AF453972 |
| Angiosperm | Apiaceae | *Osmorhiza depauperata* | AF453975 |
| Angiosperm | Apiaceae | *Osmorhiza glabrata* | AF453976 |
| Angiosperm | Apiaceae | *Osmorhiza glabrata* | AF453977 |
| Angiosperm | Apiaceae | *Osmorhiza longistylis* | AF453981 |
| Angiosperm | Apiaceae | *Osmorhiza longistylis* | DQ005965 |
| Angiosperm | Apiaceae | *Osmorhiza mexicana* subsp. *bipatriata* | AF453984 |
| Angiosperm | Apiaceae | *Osmorhiza mexicana* subsp. *mexicana* | AF453983 |
| Angiosperm | Apiaceae | *Osmorhiza occidentalis* | AF453985 |
| Angiosperm | Apiaceae | *Osmorhiza occidentalis* | AF453990 |
| Angiosperm | Apiaceae | *Osmorhiza occidentalis* | AF453991 |
| Angiosperm | Apiaceae | *Osmorhiza occidentalis* | AF453993 |
| Angiosperm | Apiaceae | *Osmorhiza purpurea* | AF453994 |
| Angiosperm | Apiaceae | *Osmorhiza purpurea* | AF453995 |
| Angiosperm | Apiaceae | *Pimpinella anisum* | EU785940 |
| Angiosperm | Apiaceae | *Pimpinella brachycarpa* | AY548230 |
| Angiosperm | Apiaceae | *Seseli diffusum* | AB243688 |
| Angiosperm | Apiaceae | *Sium suave* | AY548213 |
| Angiosperm | Apiaceae | *Thapsia garganica* | AJ007930 |
| Angiosperm | Apiaceae | *Angelica acutiloba* | AY548227 |
| Angiosperm | Apiaceae | *Angelica archangelica* | EF590754 |
| Angiosperm | Apiaceae | *Angelica cartilaginomarginata* var*. distans* | AY548222 |
| Angiosperm | Apiaceae | *Angelica decursiva* | AY548220 |
| Angiosperm | Apiaceae | *Angelica decursiva* | DQ132872 |
| Angiosperm | Apiaceae | *Angelica japonica* | AY548214 |
| Angiosperm | Apiaceae | *Angelica purpureifolia* | AY548229 |
| Angiosperm | Apiaceae | *Angelica sinensis* | AF393784 |
| Angiosperm | Apiaceae | *Bupleurum chinense* | AY551293 |
| Angiosperm | Apiaceae | *Bupleurum falcatum* | AJ131344 |
| Angiosperm | Apiaceae | *Bupleurum latissimum* | AY551292 |
| Angiosperm | Apiaceae | *Bupleurum longiradiatum* | AY551291 |
| Angiosperm | Apiaceae | *Bupleurum scorzonerifolium* | AY551294 |
| Angiosperm | Apiaceae | *Centella asiatica* | AF272352 |
| Angiosperm | Apiaceae | *Centella capensis* | AF272351 |
| Angiosperm | Apiaceae | *Cnidium monnieri* | AY863065 |
| Angiosperm | Apiaceae | *Cnidium monnieri* | EU236164 |
| Angiosperm | Apiaceae | *Cryptotaenia canadensis* | DQ005964 |
| Angiosperm | Apiaceae | *Cryptotaenia japonica* | AY548233 |
| Angiosperm | Apiaceae | *Daucus carota* | AY552527 |
| Angiosperm | Apiaceae | *Daucus glochidiatus* | EU331132 |
| Angiosperm | Apiaceae | *Eryngium vesiculosum* | U72388 |
| Angiosperm | Apiaceae | *Foeniculum vulgare* | AY551289 |
| Angiosperm | Apiaceae | *Foeniculum vulgare* var. *dulce* | EF421428 |
| Angiosperm | Apiaceae | *Foeniculum vulgare* var. *vulgare* | EU796894 |
| Angiosperm | Apiaceae | *Heracleum moellendorffii* | AY548226 |
| Angiosperm | Apiaceae | *Ligusticum jeholense* | AY548218 |
| Angiosperm | Apiaceae | *Ligusticum sinense* | AY548231 |
| Angiosperm | Apiaceae | *Notopterygium incisum* | DQ278168 |
| Angiosperm | Apiaceae | *Notopterygium incisum* | EU236180 |
| Angiosperm | Apiaceae | *Ostericum grosseserratum* | AF455749 |
| Angiosperm | Apiaceae | *Ostericum sieboldii* | AY548219 |
| Angiosperm | Apiaceae | *Peucedanum japonicum* | AJ131343 |
| Angiosperm | Apiaceae | *Peucedanum praeruptorum* | DQ132871 |
| Angiosperm | Apiaceae | *Saposhnikovia divaricata* | AY548221 |
| Angiosperm | Apiaceae | *Saposhnikovia divaricata* | DQ132873 |
| Angiosperm | Apiaceae | *Saposhnikovia divaricata* | EU164927 |
| Angiosperm | Apocynaceae | *Apocynum cannabinum* | DQ005966 |
| Angiosperm | Apocynaceae | *Apocynum venetum* | DQ449485 |
| Angiosperm | Apocynaceae | *Kopsia flavida* | AB331856 |
| Angiosperm | Apocynaceae | *Rauvolfia sumatrana* | AB365186 |
| Angiosperm | Apocynaceae | *Rauvolfia verticillata* | AB365187 |
| Angiosperm | Apocynaceae | *Trachelospermum jasminoides* | FJ980308 |
| Angiosperm | Aquifoliaceae | *Ilex amelanchier* | AJ275340 |
| Angiosperm | Aquifoliaceae | *Ilex anomala* | AJ492652 |
| Angiosperm | Aquifoliaceae | *Ilex anomala* | AJ786505 |
| Angiosperm | Aquifoliaceae | *Ilex argentina* | AJ492655 |
| Angiosperm | Aquifoliaceae | *Ilex brasiliensis* | AJ492661 |
| Angiosperm | Aquifoliaceae | *Ilex brasiliensis* | AJ786506 |
| Angiosperm | Aquifoliaceae | *Ilex brevicuspis* | AJ786507 |
| Angiosperm | Aquifoliaceae | *Ilex buergeri* | U92597 |
| Angiosperm | Aquifoliaceae | *Ilex canariensis* | AJ786490 |
| Angiosperm | Aquifoliaceae | *Ilex canariensis* | AJ786491 |
| Angiosperm | Aquifoliaceae | *Ilex canariensis* | AJ786495 |
| Angiosperm | Aquifoliaceae | *Ilex canariensis* | AJ786498 |
| Angiosperm | Aquifoliaceae | *Ilex canariensis* | AJ786499 |
| Angiosperm | Aquifoliaceae | *Ilex canariensis* | AJ786500 |
| Angiosperm | Aquifoliaceae | *Ilex canariensis* | AJ786501 |
| Angiosperm | Aquifoliaceae | *Ilex canariensis* | AJ786502 |
| Angiosperm | Aquifoliaceae | *Ilex canariensis* | AJ786503 |
| Angiosperm | Aquifoliaceae | *Ilex canariensis* | AJ786504 |
| Angiosperm | Aquifoliaceae | *Ilex cassine* | AF200588 |
| Angiosperm | Aquifoliaceae | *Ilex cassine* | AJ492667 |
| Angiosperm | Aquifoliaceae | *Ilex collina* | AF200586 |
| Angiosperm | Aquifoliaceae | *Ilex collina* | AJ492679 |
| Angiosperm | Aquifoliaceae | *Ilex cornuta* | AF200591 |
| Angiosperm | Aquifoliaceae | *Ilex crenata* | U92579 |
| Angiosperm | Aquifoliaceae | *Ilex dimorphophylla* | U92593 |
| Angiosperm | Aquifoliaceae | *Ilex glabra* | AJ275342 |
| Angiosperm | Aquifoliaceae | *Ilex goshiensis* | AJ492687 |
| Angiosperm | Aquifoliaceae | *Ilex guianensis* | AJ492668 |
| Angiosperm | Aquifoliaceae | *Ilex guianensis* | AJ492669 |
| Angiosperm | Aquifoliaceae | *Ilex hippocrateoides* | AJ492658 |
| Angiosperm | Aquifoliaceae | *Ilex integerrima* | AJ492664 |
| Angiosperm | Aquifoliaceae | *Ilex integerrima* | AJ786508 |
| Angiosperm | Aquifoliaceae | *Ilex integra* | U92591 |
| Angiosperm | Aquifoliaceae | *Ilex latifolia* | AJ492691 |
| Angiosperm | Aquifoliaceae | *Ilex latifolia* | DQ200798 |
| Angiosperm | Aquifoliaceae | *Ilex laurina* | AJ492651 |
| Angiosperm | Aquifoliaceae | *Ilex leucoclada* | AJ492690 |
| Angiosperm | Aquifoliaceae | *Ilex liukiuensis* | U92609 |
| Angiosperm | Aquifoliaceae | *Ilex macrocarpa* | AJ492689 |
| Angiosperm | Aquifoliaceae | *Ilex macropoda* | U92617 |
| Angiosperm | Aquifoliaceae | *Ilex maximowicziana* | AJ492678 |
| Angiosperm | Aquifoliaceae | *Ilex maximowicziana* | U92581 |
| Angiosperm | Aquifoliaceae | *Ilex mertensii* | U92605 |
| Angiosperm | Aquifoliaceae | *Ilex micrococca* | AJ492684 |
| Angiosperm | Aquifoliaceae | *Ilex micrococca* | U92615 |
| Angiosperm | Aquifoliaceae | *Ilex microdonta* | AJ492665 |
| Angiosperm | Aquifoliaceae | *Ilex mitis* | AJ275338 |
| Angiosperm | Aquifoliaceae | *Ilex mucronulata* | AF200587 |
| Angiosperm | Aquifoliaceae | *Ilex mutchagara* | U92583 |
| Angiosperm | Aquifoliaceae | *Ilex opaca* | AF200590 |
| Angiosperm | Aquifoliaceae | *Ilex oppositifolia* | AJ492685 |
| Angiosperm | Aquifoliaceae | *Ilex pedunculosa* | AJ275341 |
| Angiosperm | Aquifoliaceae | *Ilex pedunculosa* | U92587 |
| Angiosperm | Aquifoliaceae | *Ilex perado* | AJ492693 |
| Angiosperm | Aquifoliaceae | *Ilex perado* | AJ786413 |
| Angiosperm | Aquifoliaceae | *Ilex percoriacea* | U92607 |
| Angiosperm | Aquifoliaceae | *Ilex pseudobuxus* | AJ492660 |
| Angiosperm | Aquifoliaceae | *Ilex pseudobuxus* | AY183486 |
| Angiosperm | Aquifoliaceae | *Ilex purpurea* | AJ492673 |
| Angiosperm | Aquifoliaceae | *Ilex purpurea* | AJ786509 |
| Angiosperm | Aquifoliaceae | *Ilex repanda* | AJ492654 |
| Angiosperm | Aquifoliaceae | *Ilex revoluta* | AJ492676 |
| Angiosperm | Aquifoliaceae | *Ilex rotunda* | AJ492683 |
| Angiosperm | Aquifoliaceae | *Ilex rotunda* | AJ786510 |
| Angiosperm | Aquifoliaceae | *Ilex rotunda* | U92589 |
| Angiosperm | Aquifoliaceae | *Ilex rugosa* | AJ492692 |
| Angiosperm | Aquifoliaceae | *Ilex rugosa* | AJ786511 |
| Angiosperm | Aquifoliaceae | *Ilex rugosa* | U92611 |
| Angiosperm | Aquifoliaceae | *Ilex serrata* | AJ492682 |
| Angiosperm | Aquifoliaceae | *Ilex serrata* | U92613 |
| Angiosperm | Aquifoliaceae | *Ilex shennongjiaensis* | AJ492670 |
| Angiosperm | Aquifoliaceae | *Ilex sugerokii* | AJ492671 |
| Angiosperm | Aquifoliaceae | *Ilex teratopis* | AJ492649 |
| Angiosperm | Aquifoliaceae | *Ilex theezans* | AJ492666 |
| Angiosperm | Aquifoliaceae | *Ilex triflora* | AJ492675 |
| Angiosperm | Aquifoliaceae | *Ilex verticillata* | AJ492681 |
| Angiosperm | Aquifoliaceae | *Ilex warburgii* | U92601 |
| Angiosperm | Aquifoliaceae | *Ilex x kiusiana* | U92599 |
| Angiosperm | Aquifoliaceae | *Ilex yunnanensis* | AJ275337 |
| Angiosperm | Araceae | *Acorus americanus* | DQ008850 |
| Angiosperm | Araceae | *Acorus calamus* | DQ008848 |
| Angiosperm | Araceae | *Acorus calamus* | DQ008852 |
| Angiosperm | Araceae | *Acorus calamus* var*. angustatus* | DQ008853 |
| Angiosperm | Araceae | *Acorus gramineus* | DQ008846 |
| Angiosperm | Araceae | *Acorus gramineus* | DQ008849 |
| Angiosperm | Araceae | *Acorus latifolius* | DQ008843 |
| Angiosperm | Araceae | *Acorus macrospadiceus* | DQ008842 |
| Angiosperm | Araceae | *Acorus tatarinowii* | DQ008844 |
| Angiosperm | Araceae | *Acorus tatarinowii* | DQ008845 |
| Angiosperm | Araceae | *Acorus tatarinowii* | DQ008851 |
| Angiosperm | Araceae | *Acorus xiangyeus* | DQ008841 |
| Angiosperm | Araceae | *Arisaema angustatum* | AF291914 |
| Angiosperm | Araceae | *Calamus castaneus* | AJ242047 |
| Angiosperm | Araceae | *Calamus deeratus* | AJ242051 |
| Angiosperm | Araceae | *Calamus hollrungii* | AJ242056 |
| Angiosperm | Araceae | *Calamus hollrungii* | AJ242058 |
| Angiosperm | Araceae | *Calamus hollrungii* | AJ242061 |
| Angiosperm | Araceae | *Homalomena erythropus* | DQ866879 |
| Angiosperm | Araceae | *Homalomena panamensis* | DQ866880 |
| Angiosperm | Araceae | *Homalomena philippinensis* | DQ866881 |
| Angiosperm | Araceae | *Homalomena picturata* | DQ866882 |
| Angiosperm | Araceae | *Homalomena wendlandii* | DQ866883 |
| Angiosperm | Araceae | *Philodendron brevispathum* | DQ866887 |
| Angiosperm | Araceae | *Philodendron ecordatum* | DQ866890 |
| Angiosperm | Araceae | *Philodendron goeldii* | DQ866895 |
| Angiosperm | Araceae | *Philodendron heleniae* | DQ866897 |
| Angiosperm | Araceae | *Philodendron longistilum* | DQ866902 |
| Angiosperm | Araceae | *Philodendron lundii* | DQ866903 |
| Angiosperm | Araceae | *Philodendron ornatum* | DQ866891 |
| Angiosperm | Araceae | *Philodendron pedatum* | DQ866906 |
| Angiosperm | Araceae | *Philodendron serpens* | DQ866911 |
| Angiosperm | Araceae | *Philodendron smithii* | DQ866912 |
| Angiosperm | Araceae | *Philodendron xanadu* | DQ866920 |
| Angiosperm | Araceae | *Pinellia cordata* | AF469039 |
| Angiosperm | Araceae | *Pinellia integrifolia* | AF469033 |
| Angiosperm | Araceae | *Pinellia pedatisecta* | AF469040 |
| Angiosperm | Araceae | *Pinellia peltata* | AF469034 |
| Angiosperm | Araceae | *Pinellia polyphylla* | AF469035 |
| Angiosperm | Araceae | *Pinellia ternata* | AF469036 |
| Angiosperm | Araceae | *Pinellia tripartita* | AF469037 |
| Angiosperm | Araceae | *Pinellia yaoluopingensis* | AF469038 |
| Angiosperm | Araceae | *Zantedeschia elliottiana* | AF144037 |
| Angiosperm | Araliaceae | *Aralia armata* | AY233310 |
| Angiosperm | Araliaceae | *Aralia bipinnata* | DQ007354 |
| Angiosperm | Araliaceae | *Aralia chinensis* | U63181 |
| Angiosperm | Araliaceae | *Aralia cordata* | AJ786236 |
| Angiosperm | Araliaceae | *Aralia dasyphylla* | DQ007355 |
| Angiosperm | Araliaceae | *Aralia elata* | AJ786234 |
| Angiosperm | Araliaceae | *Aralia elata* var. *mandshurica* | AJ786233 |
| Angiosperm | Araliaceae | *Aralia foliolosa* | AY233312 |
| Angiosperm | Araliaceae | *Aralia franchetii* | AY271909 |
| Angiosperm | Araliaceae | *Aralia gigantea* | AY233313 |
| Angiosperm | Araliaceae | *Aralia gigantea* | AY233314 |
| Angiosperm | Araliaceae | *Aralia hiepiana* | AY233316 |
| Angiosperm | Araliaceae | *Aralia leschenaultii* | AY233317 |
| Angiosperm | Araliaceae | *Aralia leschenaultii* | AY233318 |
| Angiosperm | Araliaceae | *Aralia lihengiana* | AY233315 |
| Angiosperm | Araliaceae | *Aralia parasitica* | AY233319 |
| Angiosperm | Araliaceae | *Aralia subcordata* | DQ007357 |
| Angiosperm | Araliaceae | *Aralia thomsonii* | DQ007358 |
| Angiosperm | Araliaceae | *Brassaiopsis ciliata* | AF551723 |
| Angiosperm | Araliaceae | *Brassaiopsis elegans* | DQ007363 |
| Angiosperm | Araliaceae | *Brassaiopsis fatsioides* | AF551725 |
| Angiosperm | Araliaceae | *Brassaiopsis ficifolia* | AF551722 |
| Angiosperm | Araliaceae | *Brassaiopsis ficifolia* | DQ007403 |
| Angiosperm | Araliaceae | *Brassaiopsis gracilis* | AF551721 |
| Angiosperm | Araliaceae | *Brassaiopsis malayana* | DQ007367 |
| Angiosperm | Araliaceae | *Brassaiopsis mitis* | AF551726 |
| Angiosperm | Araliaceae | *Brassaiopsis phanrangensis* | AF551724 |
| Angiosperm | Araliaceae | *Brassaiopsis polyacantha* | DQ007364 |
| Angiosperm | Araliaceae | *Brassaiopsis sumatrana* | DQ007365 |
| Angiosperm | Araliaceae | *Brassaiopsis tripteris* | AY256898 |
| Angiosperm | Araliaceae | *Cheirodendron trigynum* | U63183 |
| Angiosperm | Araliaceae | *Chengiopanax sciadophylloides* | AM400882 |
| Angiosperm | Araliaceae | *Cussonia paniculata* | DQ007368 |
| Angiosperm | Araliaceae | *Dendropanax arboreus* | AF551737 |
| Angiosperm | Araliaceae | *Dendropanax dentigerus* | DQ007369 |
| Angiosperm | Araliaceae | *Dendropanax hainanensis* | DQ007402 |
| Angiosperm | Araliaceae | *Dendropanax lancifolius* | DQ007370 |
| Angiosperm | Araliaceae | *Dendropanax maingayi* | DQ007371 |
| Angiosperm | Araliaceae | *Eleutherococcus divaricatus f. distigmatis* | AY548177 |
| Angiosperm | Araliaceae | *Eleutherococcus divaricatus f. sachunense* | AY548179 |
| Angiosperm | Araliaceae | *Eleutherococcus giraldii* | AY548181 |
| Angiosperm | Araliaceae | *Eleutherococcus gracilistylus* | AY548182 |
| Angiosperm | Araliaceae | *Eleutherococcus gracilistylus* | FJ980422 |
| Angiosperm | Araliaceae | *Eleutherococcus japonicus f. kiusianus* | AY548183 |
| Angiosperm | Araliaceae | *Eleutherococcus koreanus* | AY548184 |
| Angiosperm | Araliaceae | *Eleutherococcus nodiflorus* | U63184 |
| Angiosperm | Araliaceae | *Eleutherococcus senticosus* | AY548185 |
| Angiosperm | Araliaceae | *Eleutherococcus sessiliflorus* | AF242252 |
| Angiosperm | Araliaceae | *Eleutherococcus sessiliflorus* | AJ786229 |
| Angiosperm | Araliaceae | *Eleutherococcus trifoliatus* | AF551739 |
| Angiosperm | Araliaceae | *Eleutherococcus trifoliatus* | AY548189 |
| Angiosperm | Araliaceae | *Eleutherococcus trifoliatus* | DQ007373 |
| Angiosperm | Araliaceae | *Eleutherococcus trifoliatus f. spinifolia* | AY548191 |
| Angiosperm | Araliaceae | *Evodiopanax evodiifolius* | AF242228 |
| Angiosperm | Araliaceae | *Gamblea malayana* | DQ007374 |
| Angiosperm | Araliaceae | *Hedera helix* | AM503887 |
| Angiosperm | Araliaceae | *Hedera helix* | U63186 |
| Angiosperm | Araliaceae | *Heteropanax fragrans* | DQ007377 |
| Angiosperm | Araliaceae | *Kalopanax pictus* | U63187 |
| Angiosperm | Araliaceae | *Kalopanax septemlobus* | AJ786228 |
| Angiosperm | Araliaceae | *Kalopanax septemlobus* | AY256899 |
| Angiosperm | Araliaceae | *Macropanax dispermus* | DQ007378 |
| Angiosperm | Araliaceae | *Macropanax maingayi* | DQ007379 |
| Angiosperm | Araliaceae | *Macropanax rosthornii* | AF551738 |
| Angiosperm | Araliaceae | *Merrilliopanax alpinus* | AY233309 |
| Angiosperm | Araliaceae | *Meryta balansae* | AY746556 |
| Angiosperm | Araliaceae | *Meryta coriacea* | AY746559 |
| Angiosperm | Araliaceae | *Meryta denhamii* | AY746560 |
| Angiosperm | Araliaceae | *Meryta denhamii* | DQ007380 |
| Angiosperm | Araliaceae | *Meryta heleneae* | AY746561 |
| Angiosperm | Araliaceae | *Meryta lanceolata* | AY746562 |
| Angiosperm | Araliaceae | *Meryta oxylaena* | AY746563 |
| Angiosperm | Araliaceae | *Meryta pauciflora* | U63195 |
| Angiosperm | Araliaceae | *Meryta senfftiana* | AY746564 |
| Angiosperm | Araliaceae | *Oreopanax argentatus* | DQ007400 |
| Angiosperm | Araliaceae | *Osmoxylon lineare* | DQ007382 |
| Angiosperm | Araliaceae | *Panax assamicus* | AY233320 |
| Angiosperm | Araliaceae | *Panax assamicus* | AY233322 |
| Angiosperm | Araliaceae | *Panax japonicus* | AY271918 |
| Angiosperm | Araliaceae | *Panax japonicus* var. *angustifolius* | AY271915 |
| Angiosperm | Araliaceae | *Panax japonicus* var. *angustifolius* | AY271916 |
| Angiosperm | Araliaceae | *Panax japonicus* var. *bipinnatifidus* | AY233323 |
| Angiosperm | Araliaceae | *Panax japonicus* var. *bipinnatifidus* | AY233324 |
| Angiosperm | Araliaceae | *Panax japonicus* var. *bipinnatifidus* | AY233325 |
| Angiosperm | Araliaceae | *Panax japonicus* var. *bipinnatifidus* | AY271911 |
| Angiosperm | Araliaceae | *Panax japonicus* var. *bipinnatifidus* | AY271912 |
| Angiosperm | Araliaceae | *Panax japonicus* var. *bipinnatifidus* | AY271913 |
| Angiosperm | Araliaceae | *Panax japonicus* var. *bipinnatifidus* | AY271914 |
| Angiosperm | Araliaceae | *Panax notoginseng* | AY271919 |
| Angiosperm | Araliaceae | *Panax pseudoginseng* var. *elegantior* | AY271917 |
| Angiosperm | Araliaceae | *Panax shangianus* | AY233328 |
| Angiosperm | Araliaceae | *Panax sinensis* | AY271920 |
| Angiosperm | Araliaceae | *Panax stipuleanatus* | AY271921 |
| Angiosperm | Araliaceae | *Panax stipuleanatus* | AY271922 |
| Angiosperm | Araliaceae | *Panax variabilis* | AY233329 |
| Angiosperm | Araliaceae | *Panax variabilis* | AY233330 |
| Angiosperm | Araliaceae | *Panax vietnamensis* | AY271924 |
| Angiosperm | Araliaceae | *Polyscias javanica* | DQ007383 |
| Angiosperm | Araliaceae | *Polyscias samoensis* | DQ007384 |
| Angiosperm | Araliaceae | *Pseudopanax arboreus* | U63167 |
| Angiosperm | Araliaceae | *Pseudopanax chathamicus* | U63166 |
| Angiosperm | Araliaceae | *Pseudopanax edgerleyi* | U63171 |
| Angiosperm | Araliaceae | *Pseudopanax gilliesii* | U63173 |
| Angiosperm | Araliaceae | *Pseudopanax kermadecensis* | U63175 |
| Angiosperm | Araliaceae | *Pseudopanax laetus* | U63176 |
| Angiosperm | Araliaceae | *Pseudopanax lessonii* | U63177 |
| Angiosperm | Araliaceae | *Pseudopanax linearis* | U63178 |
| Angiosperm | Araliaceae | *Pseudopanax macintyrei* | U63179 |
| Angiosperm | Araliaceae | *Raukaua anomalus* | U63164 |
| Angiosperm | Araliaceae | *Raukaua simplex* | U63180 |
| Angiosperm | Araliaceae | *Schefflera crassipes* | AY746567 |
| Angiosperm | Araliaceae | *Schefflera delavayi* | DQ007391 |
| Angiosperm | Araliaceae | *Schefflera digitata* | U63188 |
| Angiosperm | Araliaceae | *Schefflera elegantissima* | AY746568 |
| Angiosperm | Araliaceae | *Schefflera gabriellae* | AY746569 |
| Angiosperm | Araliaceae | *Schefflera heterophylla* | DQ007388 |
| Angiosperm | Araliaceae | *Schefflera hullettii* | DQ007392 |
| Angiosperm | Araliaceae | *Schefflera oblongifolia* | DQ007390 |
| Angiosperm | Araliaceae | *Schefflera oxyphylla* | DQ007393 |
| Angiosperm | Araliaceae | *Tetrapanax papyriferus* | U63192 |
| Angiosperm | Araliaceae | *Tetraplasandra hawaiensis* | DQ007395 |
| Angiosperm | Araliaceae | *Trevesia baviensis* | AF551731 |
| Angiosperm | Araliaceae | *Trevesia beccarii* | DQ007396 |
| Angiosperm | Araliaceae | *Trevesia burckii* | DQ007398 |
| Angiosperm | Araliaceae | *Trevesia lateospina* | DQ007399 |
| Angiosperm | Araliaceae | *Trevesia longipedicellata* | AF551733 |
| Angiosperm | Araliaceae | *Trevesia sundaica* | AF551732 |
| Angiosperm | Aristolochiaceae | *Aristolochia clematitis* | EF427951 |
| Angiosperm | Aristolochiaceae | *Asarum asaroides* | AF061539 |
| Angiosperm | Aristolochiaceae | *Asarum blumei* | AF061484 |
| Angiosperm | Aristolochiaceae | *Asarum canadense* | AF061450 |
| Angiosperm | Aristolochiaceae | *Asarum caudigerellum* | AF061466 |
| Angiosperm | Aristolochiaceae | *Asarum caulescens* | AF061460 |
| Angiosperm | Aristolochiaceae | *Asarum cordifolium* | AB219828 |
| Angiosperm | Aristolochiaceae | *Asarum debile* | AF061468 |
| Angiosperm | Aristolochiaceae | *Asarum dimidiatum* | AB247115 |
| Angiosperm | Aristolochiaceae | *Asarum epigynum* | AF061476 |
| Angiosperm | Aristolochiaceae | *Asarum europaeum* | AF061456 |
| Angiosperm | Aristolochiaceae | *Asarum fudsinoi* | AF061478 |
| Angiosperm | Aristolochiaceae | *Asarum heterotropoides* var. *heterotropoides* | AB248268 |
| Angiosperm | Aristolochiaceae | *Asarum himalaicum* | AF061462 |
| Angiosperm | Aristolochiaceae | *Asarum kumageanum* | AF061545 |
| Angiosperm | Aristolochiaceae | *Asarum minamitanianum* | AF061535 |
| Angiosperm | Aristolochiaceae | *Asarum muramatsui* | AB247046 |
| Angiosperm | Aristolochiaceae | *Asarum patens* | AB247101 |
| Angiosperm | Aristolochiaceae | *Asarum satsumense* | AF061549 |
| Angiosperm | Aristolochiaceae | *Asarum tohokuense* | AB247119 |
| Angiosperm | Aristolochiaceae | *Asarum versicolor* | AB247123 |
| Angiosperm | Asclepiadaceae | *Cynanchum wilfordii* | AY548207 |
| Angiosperm | Asclepiadaceae | *Hoya affinis* | DQ334481 |
| Angiosperm | Asclepiadaceae | *Hoya albiflora* | DQ334495 |
| Angiosperm | Asclepiadaceae | *Hoya anulata* | DQ334485 |
| Angiosperm | Asclepiadaceae | *Hoya ariadna* | DQ334502 |
| Angiosperm | Asclepiadaceae | *Hoya ariadna* | DQ334503 |
| Angiosperm | Asclepiadaceae | *Hoya ariadna* | DQ334504 |
| Angiosperm | Asclepiadaceae | *Hoya ariadna* | DQ334505 |
| Angiosperm | Asclepiadaceae | *Hoya australis* | DQ334428 |
| Angiosperm | Asclepiadaceae | *Hoya australis* | DQ334445 |
| Angiosperm | Asclepiadaceae | *Hoya bilobata* | DQ334490 |
| Angiosperm | Asclepiadaceae | *Hoya bilobata* | DQ334492 |
| Angiosperm | Asclepiadaceae | *Hoya camphorifolia* | DQ334471 |
| Angiosperm | Asclepiadaceae | *Hoya camphorifolia* | DQ334473 |
| Angiosperm | Asclepiadaceae | *Hoya carnosa* | DQ334463 |
| Angiosperm | Asclepiadaceae | *Hoya carnosa* | DQ334464 |
| Angiosperm | Asclepiadaceae | *Hoya caudata* | DQ334483 |
| Angiosperm | Asclepiadaceae | *Hoya ciliata* | DQ334512 |
| Angiosperm | Asclepiadaceae | *Hoya ciliata* | DQ334515 |
| Angiosperm | Asclepiadaceae | *Hoya curtisii* | DQ334479 |
| Angiosperm | Asclepiadaceae | *Hoya edeni* | DQ334476 |
| Angiosperm | Asclepiadaceae | *Hoya gracilis* | DQ334441 |
| Angiosperm | Asclepiadaceae | *Hoya gracilis* | DQ334443 |
| Angiosperm | Asclepiadaceae | *Hoya heuschkeliana* | DQ334416 |
| Angiosperm | Asclepiadaceae | *Hoya heuschkeliana* | DQ334417 |
| Angiosperm | Asclepiadaceae | *Hoya heuschkeliana* | DQ334418 |
| Angiosperm | Asclepiadaceae | *Hoya hypolasia* | DQ334470 |
| Angiosperm | Asclepiadaceae | *Hoya imbricata* | DQ334480 |
| Angiosperm | Asclepiadaceae | *Hoya kerrii* | DQ334458 |
| Angiosperm | Asclepiadaceae | *Hoya lacunosa* | DQ334499 |
| Angiosperm | Asclepiadaceae | *Hoya macgillivrayi* | DQ334488 |
| Angiosperm | Asclepiadaceae | *Hoya meliflua* | DQ334431 |
| Angiosperm | Asclepiadaceae | *Hoya meliflua* | DQ334432 |
| Angiosperm | Asclepiadaceae | *Hoya meliflua* | DQ334433 |
| Angiosperm | Asclepiadaceae | *Hoya meliflua* | DQ334434 |
| Angiosperm | Asclepiadaceae | *Hoya mitrata* | DQ334500 |
| Angiosperm | Asclepiadaceae | *Hoya multiflora* | DQ334487 |
| Angiosperm | Asclepiadaceae | *Hoya patella* | DQ334498 |
| Angiosperm | Asclepiadaceae | *Hoya pauciflora* | DQ334465 |
| Angiosperm | Asclepiadaceae | *Hoya pauciflora* | DQ334468 |
| Angiosperm | Asclepiadaceae | *Hoya pseudolittoralis* | DQ334478 |
| Angiosperm | Asclepiadaceae | *Hoya pubicalyx* | DQ334447 |
| Angiosperm | Asclepiadaceae | *Hoya pubicalyx* | DQ334448 |
| Angiosperm | Asclepiadaceae | *Hoya pubicalyx* | DQ334449 |
| Angiosperm | Asclepiadaceae | *Hoya pubicalyx* | DQ334450 |
| Angiosperm | Asclepiadaceae | *Hoya pubicalyx* | DQ334451 |
| Angiosperm | Asclepiadaceae | *Hoya retusa* | DQ334457 |
| Angiosperm | Asclepiadaceae | *Hoya spartioides* | DQ334484 |
| Angiosperm | Asclepiadaceae | *Hoya telosmoides* | DQ334486 |
| Angiosperm | Asclepiadaceae | *Hoya tsangii* | DQ334425 |
| Angiosperm | Asclepiadaceae | *Hoya tsangii* | DQ334426 |
| Angiosperm | Asclepiadaceae | *Hoya tsangii* | DQ334427 |
| Angiosperm | Asclepiadaceae | *Hoya venusta* | DQ334507 |
| Angiosperm | Asclepiadaceae | *Marsdenia carvalhoi* | DQ334419 |
| Angiosperm | Asclepiadaceae | *Marsdenia carvalhoi* | DQ334420 |
| Angiosperm | Asclepiadaceae | *Marsdenia carvalhoi* | DQ334421 |
| Angiosperm | Asclepiadaceae | *Marsdenia carvalhoi* | DQ334422 |
| Angiosperm | Asclepiadaceae | *Marsdenia carvalhoi* | DQ334423 |
| Angiosperm | Asclepiadaceae | *Periploca calophylla* | DQ916856 |
| Angiosperm | Asclepiadaceae | *Periploca graeca* | DQ916857 |
| Angiosperm | Asclepiadaceae | *Periploca laevigata* | DQ916858 |
| Angiosperm | Asclepiadaceae | *Periploca nigrescens* | DQ916859 |
| Angiosperm | Asclepiadaceae | *Periploca visciformis* | AJ581683 |
| Angiosperm | Asteraceae | *Arctium minus* | AY826230 |
| Angiosperm | Asteraceae | *Artemisia annua* | AY548199 |
| Angiosperm | Asteraceae | *Artemisia annua* | DQ005971 |
| Angiosperm | Asteraceae | *Artemisia caruifolia* var. *apiacea* | AY548198 |
| Angiosperm | Asteraceae | *Artemisia indica* | EF107651 |
| Angiosperm | Asteraceae | *Artemisia japonica* | AY548200 |
| Angiosperm | Asteraceae | *Artemisia rupestris* | AJ297261 |
| Angiosperm | Asteraceae | *Aster glehnii* | AY722010 |
| Angiosperm | Asteraceae | *Aster indicus* | EF108396 |
| Angiosperm | Asteraceae | *Atractylodes chinensis* | AY548205 |
| Angiosperm | Asteraceae | *Atractylodes japonica* | AY548203 |
| Angiosperm | Asteraceae | *Atractylodes lancea* | DQ159944 |
| Angiosperm | Asteraceae | *Atractylodes macrocephala* | AY548206 |
| Angiosperm | Asteraceae | *Bidens alba* | EF107649 |
| Angiosperm | Asteraceae | *Bidens polylepis* | DQ005975 |
| Angiosperm | Asteraceae | *Carpesium abrotanoides* | AF545824 |
| Angiosperm | Asteraceae | *Carpesium cernuum* | AF545825 |
| Angiosperm | Asteraceae | *Carpesium divaricatum* | EF210965 |
| Angiosperm | Asteraceae | *Carthamus tinctorius* | EF483943 |
| Angiosperm | Asteraceae | *Carthamus tinctorius* | EF483944 |
| Angiosperm | Asteraceae | *Carthamus tinctorius* | EF483945 |
| Angiosperm | Asteraceae | *Carthamus tinctorius* | EF483946 |
| Angiosperm | Asteraceae | *Carthamus tinctorius* | EF483947 |
| Angiosperm | Asteraceae | *Carthamus tinctorius* | EF483949 |
| Angiosperm | Asteraceae | *Carthamus tinctorius* | EF483950 |
| Angiosperm | Asteraceae | *Centipeda aotearoana* | EU352239 |
| Angiosperm | Asteraceae | *Centipeda cunninghamii* | AF422116 |
| Angiosperm | Asteraceae | *Centipeda minima* | DQ826452 |
| Angiosperm | Asteraceae | *Centipeda minima* subsp. *minima* | EU352240 |
| Angiosperm | Asteraceae | *Chrysanthemum coronarium* | EF091578 |
| Angiosperm | Asteraceae | *Chrysanthemum crassum* | EF091582 |
| Angiosperm | Asteraceae | *Chrysanthemum japonicum* | EF091587 |
| Angiosperm | Asteraceae | *Chrysanthemum okiense* | EF091589 |
| Angiosperm | Asteraceae | *Chrysanthemum weyrichii* | EF091592 |
| Angiosperm | Asteraceae | *Chrysanthemum yoshinaganthum* | EF091598 |
| Angiosperm | Asteraceae | *Cichorium bottae* | AF118919 |
| Angiosperm | Asteraceae | *Cichorium intybus* | AY504694 |
| Angiosperm | Asteraceae | *Cichorium spinosum* | AJ746394 |
| Angiosperm | Asteraceae | *Cirsium andrewsii* | AF443684 |
| Angiosperm | Asteraceae | *Cirsium aomorense* | AB035996 |
| Angiosperm | Asteraceae | *Cirsium calcareum* | AF443687 |
| Angiosperm | Asteraceae | *Cirsium canovirens* | AF443688 |
| Angiosperm | Asteraceae | *Cirsium cymosum* | AF443691 |
| Angiosperm | Asteraceae | *Cirsium discolor* | AF443692 |
| Angiosperm | Asteraceae | *Cirsium drummondii* | AY504690 |
| Angiosperm | Asteraceae | *Cirsium hydrophilum* var. *hydrophilum* | AF443698 |
| Angiosperm | Asteraceae | *Cirsium muticum* | AF443722 |
| Angiosperm | Asteraceae | *Cirsium rhaphilepis* | AF443708 |
| Angiosperm | Asteraceae | *Cirsium rhothophilum* | AF443709 |
| Angiosperm | Asteraceae | *Cirsium scariosum* | AF443693 |
| Angiosperm | Asteraceae | *Cirsium spinosissimum* | AF443720 |
| Angiosperm | Asteraceae | *Cirsium subniveum* | AF443712 |
| Angiosperm | Asteraceae | *Cirsium velatum* | AF443714 |
| Angiosperm | Asteraceae | *Cirsium vulgare* | AF443716 |
| Angiosperm | Asteraceae | *Crepis alpestris* | DQ451817 |
| Angiosperm | Asteraceae | *Crepis biennis* | DQ451818 |
| Angiosperm | Asteraceae | *Dahlia apiculata* | AY117446 |
| Angiosperm | Asteraceae | *Dahlia atropurpurea* | AY117450 |
| Angiosperm | Asteraceae | *Dahlia barkeriae* | AY117451 |
| Angiosperm | Asteraceae | *Dahlia brevis* | AY117459 |
| Angiosperm | Asteraceae | *Dahlia campanulata* | AY117440 |
| Angiosperm | Asteraceae | *Dahlia coccinea* | AY117453 |
| Angiosperm | Asteraceae | *Dahlia dissecta* | AY117465 |
| Angiosperm | Asteraceae | *Dahlia excelsa* | AY117444 |
| Angiosperm | Asteraceae | *Dahlia foeniculifolia* | AY117466 |
| Angiosperm | Asteraceae | *Dahlia hjertingii* | AY117464 |
| Angiosperm | Asteraceae | *Dahlia imperialis* | AY117441 |
| Angiosperm | Asteraceae | *Dahlia linearis* | AY117467 |
| Angiosperm | Asteraceae | *Dahlia macdougallii* | AY117445 |
| Angiosperm | Asteraceae | *Dahlia merckii* | AY117471 |
| Angiosperm | Asteraceae | *Dahlia neglecta* | AY117448 |
| Angiosperm | Asteraceae | *Dahlia rudis* | AY117443 |
| Angiosperm | Asteraceae | *Dahlia rupicola* | AY117468 |
| Angiosperm | Asteraceae | *Dahlia scapigera* | AY117460 |
| Angiosperm | Asteraceae | *Dahlia scapigeroides* | AY117469 |
| Angiosperm | Asteraceae | *Dahlia sherffii* | AY117449 |
| Angiosperm | Asteraceae | *Dahlia spectabilis* | AY117462 |
| Angiosperm | Asteraceae | *Dahlia sublignosa* | DQ198259 |
| Angiosperm | Asteraceae | *Dahlia tenuicaulis* | AY117447 |
| Angiosperm | Asteraceae | *Echinacea purpurea* | EU785937 |
| Angiosperm | Asteraceae | *Echinops albicaulis* | AY538638 |
| Angiosperm | Asteraceae | *Echinops bannaticus* | AY538636 |
| Angiosperm | Asteraceae | *Echinops cornigerus* | AY538645 |
| Angiosperm | Asteraceae | *Echinops emiliae* | AY538641 |
| Angiosperm | Asteraceae | *Echinops fontqueri* | AY538632 |
| Angiosperm | Asteraceae | *Echinops galaticus* | AY538644 |
| Angiosperm | Asteraceae | *Echinops hedgei* | AY538648 |
| Angiosperm | Asteraceae | *Echinops heteromorphus* | AY538630 |
| Angiosperm | Asteraceae | *Echinops humilis* | AY538635 |
| Angiosperm | Asteraceae | *Echinops microcephalus* | AY538643 |
| Angiosperm | Asteraceae | *Echinops nanus* | AY538654 |
| Angiosperm | Asteraceae | *Echinops niveus* | AY538634 |
| Angiosperm | Asteraceae | *Echinops onopordum* | AY538642 |
| Angiosperm | Asteraceae | *Echinops persicus* | AY538639 |
| Angiosperm | Asteraceae | *Echinops przewalskii* | AY914820 |
| Angiosperm | Asteraceae | *Echinops saissanicus* | AY538640 |
| Angiosperm | Asteraceae | *Echinops siculus* | AY538652 |
| Angiosperm | Asteraceae | *Echinops sphaerocephalus* | AY538637 |
| Angiosperm | Asteraceae | *Echinops strigosus* | AY538653 |
| Angiosperm | Asteraceae | *Echinops tournefortii* | AY538646 |
| Angiosperm | Asteraceae | *Echinops tschimganicus* | AY538633 |
| Angiosperm | Asteraceae | *Echinops viscosus* | AY826283 |
| Angiosperm | Asteraceae | *Eclipta prostrata* | DQ005977 |
| Angiosperm | Asteraceae | *Eclipta prostrata* | EF190031 |
| Angiosperm | Asteraceae | *Elephantopus mollis* | EF108394 |
| Angiosperm | Asteraceae | *Elephantopus scaber* | DQ813304 |
| Angiosperm | Asteraceae | *Emilia coccinea* | EF538194 |
| Angiosperm | Asteraceae | *Emilia discifolia* | AY953930 |
| Angiosperm | Asteraceae | *Emilia prenanthoidea* | EF538196 |
| Angiosperm | Asteraceae | *Emilia sonchifolia* var. *javanica* | EF108405 |
| Angiosperm | Asteraceae | *Erigeron annuus* | EF107653 |
| Angiosperm | Asteraceae | *Erigeron morrisonensis* var. *fukuyamae* | EF107654 |
| Angiosperm | Asteraceae | *Eupatorium altissimum* | DQ236178 |
| Angiosperm | Asteraceae | *Eupatorium cannabinum* | AB032039 |
| Angiosperm | Asteraceae | *Eupatorium glaucescens* | DQ236199 |
| Angiosperm | Asteraceae | *Eupatorium glehnii* | AB032033 |
| Angiosperm | Asteraceae | *Eupatorium godfreyanum* | DQ236190 |
| Angiosperm | Asteraceae | *Eupatorium hyssopifolium* | DQ236177 |
| Angiosperm | Asteraceae | *Eupatorium japonicum* | AB032034 |
| Angiosperm | Asteraceae | *Eupatorium lancifolium* | DQ236175 |
| Angiosperm | Asteraceae | *Eupatorium leucolepis* | DQ415738 |
| Angiosperm | Asteraceae | *Eupatorium mikanioides* | DQ415739 |
| Angiosperm | Asteraceae | *Eupatorium mohrii* | DQ236204 |
| Angiosperm | Asteraceae | *Eupatorium novae-angliae* | DQ415744 |
| Angiosperm | Asteraceae | *Eupatorium paludicola* | DQ415735 |
| Angiosperm | Asteraceae | *Eupatorium petaloideum* | EU646467 |
| Angiosperm | Asteraceae | *Eupatorium pilosum* | DQ236198 |
| Angiosperm | Asteraceae | *Eupatorium resinosum* | DQ415742 |
| Angiosperm | Asteraceae | *Eupatorium rotundifolium* | DQ236197 |
| Angiosperm | Asteraceae | *Eupatorium saltuense* | EU646496 |
| Angiosperm | Asteraceae | *Eupatorium semiserratum* | DQ236173 |
| Angiosperm | Asteraceae | *Eupatorium serotinum* | DQ236176 |
| Angiosperm | Asteraceae | *Eupatorium sessilifolium* | DQ236184 |
| Angiosperm | Asteraceae | *Eutrochium dubium* | AB032040 |
| Angiosperm | Asteraceae | *Eutrochium fistulosum* | AB032041 |
| Angiosperm | Asteraceae | *Gaillardia pulchella* | EF108395 |
| Angiosperm | Asteraceae | *Gnaphalium affine* | EF108400 |
| Angiosperm | Asteraceae | *Gnaphalium hypoleucum* | EF108392 |
| Angiosperm | Asteraceae | *Gnaphalium luteoalbum* var. *compactum* | EU352242 |
| Angiosperm | Asteraceae | *Graphistylis dichroa* | EF538212 |
| Angiosperm | Asteraceae | *Hemistepta lyrata* | AB118139 |
| Angiosperm | Asteraceae | *Inula racemosa* | EU257425 |
| Angiosperm | Asteraceae | *Inula racemosa* | EU257426 |
| Angiosperm | Asteraceae | *Ixeridium chinense* | AY862578 |
| Angiosperm | Asteraceae | *Ixeridium laevigatum* | AY862582 |
| Angiosperm | Asteraceae | *Ixeris stolonifera* | AJ633284 |
| Angiosperm | Asteraceae | *Jacobaea vulgaris* | AY554112 |
| Angiosperm | Asteraceae | *Ligularia pleurocaulis* | AB212735 |
| Angiosperm | Asteraceae | *Ligularia tsangchanensis* | AB284129 |
| Angiosperm | Asteraceae | *Ligularia virgaurea* | AB245093 |
| Angiosperm | Asteraceae | *Matricaria chamomilla* | AM086626 |
| Angiosperm | Asteraceae | *Matricaria matricarioides* | EU179213 |
| Angiosperm | Asteraceae | *Matricaria recutita* | EU179212 |
| Angiosperm | Asteraceae | *Monticalia abietina* | EF538255 |
| Angiosperm | Asteraceae | *Monticalia arbutifolia* | EF538257 |
| Angiosperm | Asteraceae | *Monticalia flocculidens* | EF538259 |
| Angiosperm | Asteraceae | *Monticalia greenmaniana* | EF538260 |
| Angiosperm | Asteraceae | *Monticalia myrsinites* | EF538261 |
| Angiosperm | Asteraceae | *Monticalia vaccinioides* | EF538263 |
| Angiosperm | Asteraceae | *Parasenecio delphinifolius* | EF538281 |
| Angiosperm | Asteraceae | *Parthenium hysterophorus* | EF108403 |
| Angiosperm | Asteraceae | *Petasites fragrans* | AY554108 |
| Angiosperm | Asteraceae | *Picris burbidgeae* | EU352247 |
| Angiosperm | Asteraceae | *Picris echioides* | DQ451796 |
| Angiosperm | Asteraceae | *Picris hieracioides* | AF528490 |
| Angiosperm | Asteraceae | *Picris hieracioides* subsp. *morrisonensis* | EF107658 |
| Angiosperm | Asteraceae | *Picris hispanica* | DQ451809 |
| Angiosperm | Asteraceae | *Picris rhagadioloides* | DQ451815 |
| Angiosperm | Asteraceae | *Picris squarrosa* | DQ451813 |
| Angiosperm | Asteraceae | *Rhaponticum canariense* | DQ310954 |
| Angiosperm | Asteraceae | *Rhaponticum carthamoides* | DQ310933 |
| Angiosperm | Asteraceae | *Rhaponticum cossonianum* | DQ310949 |
| Angiosperm | Asteraceae | *Rhaponticum cynaroides* | DQ310946 |
| Angiosperm | Asteraceae | *Rhaponticum exaltatum* | DQ310953 |
| Angiosperm | Asteraceae | *Rhaponticum heleniifolium* | DQ310945 |
| Angiosperm | Asteraceae | *Rhaponticum insigne* | DQ310944 |
| Angiosperm | Asteraceae | *Rhaponticum integrifolium* | DQ310934 |
| Angiosperm | Asteraceae | *Rhaponticum longifolium* | DQ310950 |
| Angiosperm | Asteraceae | *Rhaponticum lyratum* | DQ310935 |
| Angiosperm | Asteraceae | *Rhaponticum nitidum* | DQ310937 |
| Angiosperm | Asteraceae | *Rhaponticum pulchrum* | DQ310943 |
| Angiosperm | Asteraceae | *Rhaponticum serratuloides* | DQ310941 |
| Angiosperm | Asteraceae | *Rhaponticum uniflorum* | DQ310932 |
| Angiosperm | Asteraceae | *Saussurea alpina* | AB118124 |
| Angiosperm | Asteraceae | *Saussurea costus* | EU239685 |
| Angiosperm | Asteraceae | *Saussurea costus* | EU257418 |
| Angiosperm | Asteraceae | *Saussurea hookeri* | AB118118 |
| Angiosperm | Asteraceae | *Saussurea katochaete* | AB118120 |
| Angiosperm | Asteraceae | *Saussurea przewalskii* | AB118127 |
| Angiosperm | Asteraceae | *Saussurea rockii* | AB118128 |
| Angiosperm | Asteraceae | *Saussurea stracheyana* | AB118131 |
| Angiosperm | Asteraceae | *Saussurea subulata* | AB118132 |
| Angiosperm | Asteraceae | *Senecio achilleifolius* | AY953929 |
| Angiosperm | Asteraceae | *Senecio adamantinus* | EF538294 |
| Angiosperm | Asteraceae | *Senecio algens* | EF538296 |
| Angiosperm | Asteraceae | *Senecio arnaldii* | EF538297 |
| Angiosperm | Asteraceae | *Senecio arnicoides* | EF538298 |
| Angiosperm | Asteraceae | *Senecio aspericaulis* | EF538299 |
| Angiosperm | Asteraceae | *Senecio ayopayensis* | EF538303 |
| Angiosperm | Asteraceae | *Senecio banksii* | EF538305 |
| Angiosperm | Asteraceae | *Senecio bonariensis* | EF538306 |
| Angiosperm | Asteraceae | *Senecio brassii* | EF538307 |
| Angiosperm | Asteraceae | *Senecio callosus* | EF538310 |
| Angiosperm | Asteraceae | *Senecio candollei* | EF538311 |
| Angiosperm | Asteraceae | *Senecio carnosulus* | EU331121 |
| Angiosperm | Asteraceae | *Senecio colensoi* | EF538315 |
| Angiosperm | Asteraceae | *Senecio cordifolius* | AY953926 |
| Angiosperm | Asteraceae | *Senecio coriaceus* | EF538317 |
| Angiosperm | Asteraceae | *Senecio crassissimus* | EF538318 |
| Angiosperm | Asteraceae | *Senecio cremeiflorus* | EF538320 |
| Angiosperm | Asteraceae | *Senecio crithmoides* | EF538321 |
| Angiosperm | Asteraceae | *Senecio ctenophyllus* | EF538322 |
| Angiosperm | Asteraceae | *Senecio cunninghamii* | EF538323 |
| Angiosperm | Asteraceae | *Senecio decurrens* | EF538324 |
| Angiosperm | Asteraceae | *Senecio deferens* | EF538325 |
| Angiosperm | Asteraceae | *Senecio doronicum* | EF538330 |
| Angiosperm | Asteraceae | *Senecio dregeanus* | EF538331 |
| Angiosperm | Asteraceae | *Senecio dunedinensis* | AY554109 |
| Angiosperm | Asteraceae | *Senecio elegans* | EU812812 |
| Angiosperm | Asteraceae | *Senecio espinosae* | EF538332 |
| Angiosperm | Asteraceae | *Senecio farinifer* | EF538333 |
| Angiosperm | Asteraceae | *Senecio filaginoides* | EF538334 |
| Angiosperm | Asteraceae | *Senecio fistulosus* | EF538335 |
| Angiosperm | Asteraceae | *Senecio franchetii* | EF538337 |
| Angiosperm | Asteraceae | *Senecio glaucophyllus* | AY554110 |
| Angiosperm | Asteraceae | *Senecio glaucophyllus* subsp. *glaucophyllus* | EU812813 |
| Angiosperm | Asteraceae | *Senecio glomeratus* | AY554111 |
| Angiosperm | Asteraceae | *Senecio grossidens* | EF538342 |
| Angiosperm | Asteraceae | *Senecio gunnii* | EF538343 |
| Angiosperm | Asteraceae | *Senecio hansweberi* | EF538344 |
| Angiosperm | Asteraceae | *Senecio hastatus* | EF538345 |
| Angiosperm | Asteraceae | *Senecio hederiformis* | AY953928 |
| Angiosperm | Asteraceae | *Senecio hemmendorffii* | EF538346 |
| Angiosperm | Asteraceae | *Senecio hispidulus* | EU331118 |
| Angiosperm | Asteraceae | *Senecio humillimus* | EF538347 |
| Angiosperm | Asteraceae | *Senecio junceus* | EF538351 |
| Angiosperm | Asteraceae | *Senecio laetevirens* | EF538352 |
| Angiosperm | Asteraceae | *Senecio lautus* var. *esperensis* | AY554113 |
| Angiosperm | Asteraceae | *Senecio lautus* var. *lautus* | EU812814 |
| Angiosperm | Asteraceae | *Senecio leptolobus* | EF538355 |
| Angiosperm | Asteraceae | *Senecio linifolius* | EF538356 |
| Angiosperm | Asteraceae | *Senecio macranthus* | EF538358 |
| Angiosperm | Asteraceae | *Senecio madagascariensis* | DQ322599 |
| Angiosperm | Asteraceae | *Senecio madagascariensis* | DQ322600 |
| Angiosperm | Asteraceae | *Senecio madagascariensis* | DQ322602 |
| Angiosperm | Asteraceae | *Senecio madagascariensis* | DQ322606 |
| Angiosperm | Asteraceae | *Senecio madagascariensis* | DQ322609 |
| Angiosperm | Asteraceae | *Senecio madagascariensis* | DQ322610 |
| Angiosperm | Asteraceae | *Senecio madagascariensis* | DQ322612 |
| Angiosperm | Asteraceae | *Senecio meyeri-johannis* | EF538360 |
| Angiosperm | Asteraceae | *Senecio niveoaureus* | EF538178 |
| Angiosperm | Asteraceae | *Senecio otites* | EF538363 |
| Angiosperm | Asteraceae | *Senecio panduriformis* | EF538364 |
| Angiosperm | Asteraceae | *Senecio pinifolius* | EF538366 |
| Angiosperm | Asteraceae | *Senecio pubiger* | EF538368 |
| Angiosperm | Asteraceae | *Senecio purpureus* | EF538370 |
| Angiosperm | Asteraceae | *Senecio quadridentatus* | AF422134 |
| Angiosperm | Asteraceae | *Senecio retrorsus* | EF538372 |
| Angiosperm | Asteraceae | *Senecio roseus* | EF538373 |
| Angiosperm | Asteraceae | *Senecio rufescens* | EF538374 |
| Angiosperm | Asteraceae | *Senecio rufiglandulosus* | AF422135 |
| Angiosperm | Asteraceae | *Senecio saxatilis* | EF538376 |
| Angiosperm | Asteraceae | *Senecio selloi* | EF538379 |
| Angiosperm | Asteraceae | *Senecio sinuatilobus* | EF538380 |
| Angiosperm | Asteraceae | *Senecio sisymbriifolius* | EF538381 |
| Angiosperm | Asteraceae | *Senecio smithii* | EF538382 |
| Angiosperm | Asteraceae | *Senecio spartioides* | EF538383 |
| Angiosperm | Asteraceae | *Senecio sterquilinus* | EU331122 |
| Angiosperm | Asteraceae | *Senecio sylvaticus* | AF459928 |
| Angiosperm | Asteraceae | *Senecio sylvaticus* | EU812815 |
| Angiosperm | Asteraceae | *Senecio telekii* | EF538387 |
| Angiosperm | Asteraceae | *Senecio triangularis* | EF538389 |
| Angiosperm | Asteraceae | *Senecio trifidus* | EF538391 |
| Angiosperm | Asteraceae | *Senecio triqueter* | EF538392 |
| Angiosperm | Asteraceae | *Senecio vulgaris* | AF422136 |
| Angiosperm | Asteraceae | *Senecio vulgaris* | EF538396 |
| Angiosperm | Asteraceae | *Senecio wairauensis* | EF538397 |
| Angiosperm | Asteraceae | *Senecio wairauensis* | EU812816 |
| Angiosperm | Asteraceae | *Sigesbeckia flosculosa* | AF465888 |
| Angiosperm | Asteraceae | *Sigesbeckia jorullensis* | AF465889 |
| Angiosperm | Asteraceae | *Sigesbeckia orientalis* | AF465890 |
| Angiosperm | Asteraceae | *Silybum marianum* | AJ831537 |
| Angiosperm | Asteraceae | *Solidago flexicaulis* | DQ005979 |
| Angiosperm | Asteraceae | *Solidago gigantea* | DQ005980 |
| Angiosperm | Asteraceae | *Solidago juncea* | DQ005981 |
| Angiosperm | Asteraceae | *Solidago simplex* | DQ005982 |
| Angiosperm | Asteraceae | *Sonchus kirkii* | AF422137 |
| Angiosperm | Asteraceae | *Synedrella nodiflora* | EF108404 |
| Angiosperm | Asteraceae | *Tagetes filifolia* | DQ862118 |
| Angiosperm | Asteraceae | *Tagetes foetidissima* | DQ862119 |
| Angiosperm | Asteraceae | *Tagetes lucida* | DQ862120 |
| Angiosperm | Asteraceae | *Tagetes patula* | DQ862121 |
| Angiosperm | Asteraceae | *Taraxacum album* | EU637105 |
| Angiosperm | Asteraceae | *Taraxacum album* | EU637106 |
| Angiosperm | Asteraceae | *Taraxacum album* | EU637107 |
| Angiosperm | Asteraceae | *Taraxacum album* | EU637108 |
| Angiosperm | Asteraceae | *Taraxacum album* | EU637109 |
| Angiosperm | Asteraceae | *Taraxacum album* | EU637110 |
| Angiosperm | Asteraceae | *Taraxacum album* | EU637111 |
| Angiosperm | Asteraceae | *Taraxacum album* | EU637113 |
| Angiosperm | Asteraceae | *Taraxacum album* | EU637114 |
| Angiosperm | Asteraceae | *Taraxacum armeriifolium* | EU637115 |
| Angiosperm | Asteraceae | *Taraxacum armeriifolium* | EU637117 |
| Angiosperm | Asteraceae | *Taraxacum armeriifolium* | EU637119 |
| Angiosperm | Asteraceae | *Taraxacum bessarabicum* | EU637121 |
| Angiosperm | Asteraceae | *Taraxacum bessarabicum* | EU637122 |
| Angiosperm | Asteraceae | *Taraxacum bessarabicum* | EU637124 |
| Angiosperm | Asteraceae | *Taraxacum bessarabicum* | EU637126 |
| Angiosperm | Asteraceae | *Taraxacum bessarabicum* | EU637128 |
| Angiosperm | Asteraceae | *Taraxacum cylleneum* | EU637135 |
| Angiosperm | Asteraceae | *Taraxacum cylleneum* | EU637136 |
| Angiosperm | Asteraceae | *Taraxacum cylleneum* | EU637140 |
| Angiosperm | Asteraceae | *Taraxacum cylleneum* | EU637141 |
| Angiosperm | Asteraceae | *Taraxacum cylleneum* | EU637142 |
| Angiosperm | Asteraceae | *Taraxacum cylleneum* | EU637143 |
| Angiosperm | Asteraceae | *Taraxacum formosissimum* | EU637144 |
| Angiosperm | Asteraceae | *Taraxacum formosissimum* | EU637145 |
| Angiosperm | Asteraceae | *Taraxacum formosissimum* | EU637146 |
| Angiosperm | Asteraceae | *Taraxacum formosissimum* | EU637147 |
| Angiosperm | Asteraceae | *Taraxacum formosissimum* | EU637148 |
| Angiosperm | Asteraceae | *Taraxacum formosissimum* | EU637149 |
| Angiosperm | Asteraceae | *Taraxacum formosissimum* | EU637150 |
| Angiosperm | Asteraceae | *Taraxacum formosissimum* | EU637151 |
| Angiosperm | Asteraceae | *Taraxacum formosissimum* | EU637152 |
| Angiosperm | Asteraceae | *Taraxacum formosissimum* | EU637153 |
| Angiosperm | Asteraceae | *Taraxacum glaucophyllum* | EU637155 |
| Angiosperm | Asteraceae | *Taraxacum glaucophyllum* | EU637156 |
| Angiosperm | Asteraceae | *Taraxacum glaucophyllum* | EU637158 |
| Angiosperm | Asteraceae | *Taraxacum glaucophyllum* | EU637163 |
| Angiosperm | Asteraceae | *Taraxacum glaucophyllum* | EU637164 |
| Angiosperm | Asteraceae | *Taraxacum inimitabile* | EU637166 |
| Angiosperm | Asteraceae | *Taraxacum inimitabile* | EU637167 |
| Angiosperm | Asteraceae | *Taraxacum inimitabile* | EU637168 |
| Angiosperm | Asteraceae | *Taraxacum inimitabile* | EU637169 |
| Angiosperm | Asteraceae | *Taraxacum inimitabile* | EU637170 |
| Angiosperm | Asteraceae | *Taraxacum inimitabile* | EU637171 |
| Angiosperm | Asteraceae | *Taraxacum inimitabile* | EU637172 |
| Angiosperm | Asteraceae | *Taraxacum inimitabile* | EU637173 |
| Angiosperm | Asteraceae | *Taraxacum inimitabile* | EU637174 |
| Angiosperm | Asteraceae | *Taraxacum leucanthum* | EU637185 |
| Angiosperm | Asteraceae | *Taraxacum leucanthum* | EU637186 |
| Angiosperm | Asteraceae | *Taraxacum leucanthum* | EU637187 |
| Angiosperm | Asteraceae | *Taraxacum leucanthum* | EU637188 |
| Angiosperm | Asteraceae | *Taraxacum leucanthum* | EU637189 |
| Angiosperm | Asteraceae | *Taraxacum leucanthum* | EU637190 |
| Angiosperm | Asteraceae | *Taraxacum leucanthum* | EU637192 |
| Angiosperm | Asteraceae | *Taraxacum leucanthum* | EU637194 |
| Angiosperm | Asteraceae | *Taraxacum luridum* | EU637195 |
| Angiosperm | Asteraceae | *Taraxacum luridum* | EU637196 |
| Angiosperm | Asteraceae | *Taraxacum luridum* | EU637197 |
| Angiosperm | Asteraceae | *Taraxacum luridum* | EU637198 |
| Angiosperm | Asteraceae | *Taraxacum luridum* | EU637199 |
| Angiosperm | Asteraceae | *Taraxacum luridum* | EU637200 |
| Angiosperm | Asteraceae | *Taraxacum margaritarium* | EU637201 |
| Angiosperm | Asteraceae | *Taraxacum mongolicum* | AY548210 |
| Angiosperm | Asteraceae | *Taraxacum mongolicum* | EF114672 |
| Angiosperm | Asteraceae | *Taraxacum mongoliforme* | EU637203 |
| Angiosperm | Asteraceae | *Taraxacum mongoliforme* | EU637204 |
| Angiosperm | Asteraceae | *Taraxacum mongoliforme* | EU637208 |
| Angiosperm | Asteraceae | *Taraxacum mongoliforme* | EU637209 |
| Angiosperm | Asteraceae | *Taraxacum mongoliforme* | EU637211 |
| Angiosperm | Asteraceae | *Taraxacum niveum* | EU637212 |
| Angiosperm | Asteraceae | *Taraxacum niveum* | EU637213 |
| Angiosperm | Asteraceae | *Taraxacum niveum* | EU637214 |
| Angiosperm | Asteraceae | *Taraxacum niveum* | EU637215 |
| Angiosperm | Asteraceae | *Taraxacum niveum* | EU637216 |
| Angiosperm | Asteraceae | *Taraxacum niveum* | EU637217 |
| Angiosperm | Asteraceae | *Taraxacum niveum* | EU637218 |
| Angiosperm | Asteraceae | *Taraxacum niveum* | EU637219 |
| Angiosperm | Asteraceae | *Taraxacum niveum* | EU637220 |
| Angiosperm | Asteraceae | *Taraxacum niveum* | EU637221 |
| Angiosperm | Asteraceae | *Taraxacum niveum* | EU637222 |
| Angiosperm | Asteraceae | *Taraxacum nobile* | EU637223 |
| Angiosperm | Asteraceae | *Taraxacum nobile* | EU637224 |
| Angiosperm | Asteraceae | *Taraxacum nobile* | EU637225 |
| Angiosperm | Asteraceae | *Taraxacum nobile* | EU637226 |
| Angiosperm | Asteraceae | *Taraxacum nobile* | EU637229 |
| Angiosperm | Asteraceae | *Taraxacum nobile* | EU637230 |
| Angiosperm | Asteraceae | *Taraxacum nobile* | EU637231 |
| Angiosperm | Asteraceae | *Taraxacum nobile* | EU637232 |
| Angiosperm | Asteraceae | *Taraxacum occultum* | EU637233 |
| Angiosperm | Asteraceae | *Taraxacum occultum* | EU637237 |
| Angiosperm | Asteraceae | *Taraxacum occultum* | EU637238 |
| Angiosperm | Asteraceae | *Taraxacum occultum* | EU637239 |
| Angiosperm | Asteraceae | *Taraxacum occultum* | EU637240 |
| Angiosperm | Asteraceae | *Taraxacum occultum* | EU637241 |
| Angiosperm | Asteraceae | *Taraxacum officinale* | AY548211 |
| Angiosperm | Asteraceae | *Taraxacum serotinum* | EU637252 |
| Angiosperm | Asteraceae | *Taraxacum serotinum* | EU637253 |
| Angiosperm | Asteraceae | *Taraxacum serotinum* | EU637254 |
| Angiosperm | Asteraceae | *Taraxacum serotinum* | EU637255 |
| Angiosperm | Asteraceae | *Taraxacum serotinum* | EU637256 |
| Angiosperm | Asteraceae | *Taraxacum sherriffii* | EU637257 |
| Angiosperm | Asteraceae | *Taraxacum sherriffii* | EU637258 |
| Angiosperm | Asteraceae | *Taraxacum sherriffii* | EU637259 |
| Angiosperm | Asteraceae | *Taraxacum sherriffii* | EU637260 |
| Angiosperm | Asteraceae | *Taraxacum sherriffii* | EU637261 |
| Angiosperm | Asteraceae | *Taraxacum sherriffii* | EU637262 |
| Angiosperm | Asteraceae | *Taraxacum sherriffii* | EU637263 |
| Angiosperm | Asteraceae | *Taraxacum sherriffii* | EU637265 |
| Angiosperm | Asteraceae | *Taraxacum sherriffii* | EU637266 |
| Angiosperm | Asteraceae | *Taraxacum sinicum* | EU637270 |
| Angiosperm | Asteraceae | *Taraxacum sinicum* | EU637272 |
| Angiosperm | Asteraceae | *Taraxacum sinicum* | EU637274 |
| Angiosperm | Asteraceae | *Taraxacum sinicum* | EU637275 |
| Angiosperm | Asteraceae | *Taraxacum sinicum* | EU637276 |
| Angiosperm | Asteraceae | *Taraxacum sinicum* | EU637277 |
| Angiosperm | Asteraceae | *Taraxacum stenocephalum* | EU637278 |
| Angiosperm | Asteraceae | *Taraxacum stenocephalum* | EU637279 |
| Angiosperm | Asteraceae | *Taraxacum stenocephalum* | EU637283 |
| Angiosperm | Asteraceae | *Taraxacum stenocephalum* | EU637286 |
| Angiosperm | Asteraceae | *Taraxacum stupendum* | EU637287 |
| Angiosperm | Asteraceae | *Taraxacum stupendum* | EU637288 |
| Angiosperm | Asteraceae | *Taraxacum stupendum* | EU637289 |
| Angiosperm | Asteraceae | *Taraxacum stupendum* | EU637290 |
| Angiosperm | Asteraceae | *Taraxacum stupendum* | EU637291 |
| Angiosperm | Asteraceae | *Taraxacum stupendum* | EU637292 |
| Angiosperm | Asteraceae | *Taraxacum suasorium* | EU637297 |
| Angiosperm | Asteraceae | *Taraxacum suasorium* | EU637299 |
| Angiosperm | Asteraceae | *Taraxacum suasorium* | EU637303 |
| Angiosperm | Asteraceae | *Taraxacum suasorium* | EU637304 |
| Angiosperm | Asteraceae | *Taraxacum suasorium* | EU637306 |
| Angiosperm | Asteraceae | *Taraxacum suave* | EU637309 |
| Angiosperm | Asteraceae | *Taraxacum suave* | EU637311 |
| Angiosperm | Asteraceae | *Taraxacum suave* | EU637312 |
| Angiosperm | Asteraceae | *Taraxacum suave* | EU637315 |
| Angiosperm | Asteraceae | *Taraxacum suave* | EU637316 |
| Angiosperm | Asteraceae | *Taraxacum suave* | EU637317 |
| Angiosperm | Asteraceae | *Taraxacum tenuifolium* | EU637319 |
| Angiosperm | Asteraceae | *Taraxacum tenuifolium* | EU637322 |
| Angiosperm | Asteraceae | *Taraxacum tenuifolium* | EU637323 |
| Angiosperm | Asteraceae | *Taraxacum tenuifolium* | EU637324 |
| Angiosperm | Asteraceae | *Taraxacum tenuifolium* | EU637328 |
| Angiosperm | Asteraceae | *Taraxacum venustius* | EU637329 |
| Angiosperm | Asteraceae | *Taraxacum venustius* | EU637330 |
| Angiosperm | Asteraceae | *Taraxacum venustius* | EU637331 |
| Angiosperm | Asteraceae | *Taraxacum venustius* | EU637335 |
| Angiosperm | Asteraceae | *Taraxacum virgineum* | EU637336 |
| Angiosperm | Asteraceae | *Taraxacum virgineum* | EU637340 |
| Angiosperm | Asteraceae | *Tragopogon dubius* | AM493992 |
| Angiosperm | Asteraceae | *Tragopogon dubius* | AM493993 |
| Angiosperm | Asteraceae | *Tragopogon miscellus* | AY458588 |
| Angiosperm | Asteraceae | *Tragopogon porrifolius* | AM493994 |
| Angiosperm | Asteraceae | *Tragopogon porrifolius* | AM493995 |
| Angiosperm | Asteraceae | *Tragopogon pratensis* | AM493998 |
| Angiosperm | Asteraceae | *Tragopogon pratensis* | AM493999 |
| Angiosperm | Asteraceae | *Tussilago farfara* | AY176167 |
| Angiosperm | Asteraceae | *Tussilago farfara* | EU785941 |
| Angiosperm | Asteraceae | *Vernonia cinerea* | EF107650 |
| Angiosperm | Asteraceae | *Xanthium strumarium* | DQ005983 |
| Angiosperm | Balsaminaceae | *Impatiens buccinalis* | EF649983 |
| Angiosperm | Balsaminaceae | *Impatiens hians* | EF649985 |
| Angiosperm | Balsaminaceae | *Impatiens javensis* | AB119663 |
| Angiosperm | Balsaminaceae | *Impatiens javensis* | AB119666 |
| Angiosperm | Balsaminaceae | *Impatiens javensis* | AB119667 |
| Angiosperm | Balsaminaceae | *Impatiens javensis* | AB119668 |
| Angiosperm | Balsaminaceae | *Impatiens javensis* | AB119669 |
| Angiosperm | Balsaminaceae | *Impatiens javensis* | AB119670 |
| Angiosperm | Balsaminaceae | *Impatiens javensis* | AB175947 |
| Angiosperm | Balsaminaceae | *Impatiens mackeyana* subsp. *zenkeri* | EF649984 |
| Angiosperm | Balsaminaceae | *Impatiens manteroana* | EF649986 |
| Angiosperm | Balsaminaceae | *Impatiens radicans* | AB119682 |
| Angiosperm | Balsaminaceae | *Impatiens radicans* | AB175965 |
| Angiosperm | Balsaminaceae | *Impatiens thomensis* | EF649987 |
| Angiosperm | Begoniaceae | *Begonia aequata* | AF485147 |
| Angiosperm | Begoniaceae | *Begonia ampla* | AY429306 |
| Angiosperm | Begoniaceae | *Begonia angularis* | AF485183 |
| Angiosperm | Begoniaceae | *Begonia ankaranensis* | AF485229 |
| Angiosperm | Begoniaceae | *Begonia annulata* | AF485095 |
| Angiosperm | Begoniaceae | *Begonia aptera* | AJ287258 |
| Angiosperm | Begoniaceae | *Begonia aptera* | AJ287259 |
| Angiosperm | Begoniaceae | *Begonia aptera* | AJ287260 |
| Angiosperm | Begoniaceae | *Begonia aptera* | AJ287261 |
| Angiosperm | Begoniaceae | *Begonia aptera* | AJ287262 |
| Angiosperm | Begoniaceae | *Begonia aptera* | AJ491196 |
| Angiosperm | Begoniaceae | *Begonia austrotaiwanensis* | AJ491198 |
| Angiosperm | Begoniaceae | *Begonia baccata* | AY429305 |
| Angiosperm | Begoniaceae | *Begonia bogneri* | AF485235 |
| Angiosperm | Begoniaceae | *Begonia boliviensis* | AF485198 |
| Angiosperm | Begoniaceae | *Begonia brevirimosa* | AF485145 |
| Angiosperm | Begoniaceae | *Begonia bui-montana* | AJ491200 |
| Angiosperm | Begoniaceae | *Begonia chitoensis* | AJ491201 |
| Angiosperm | Begoniaceae | *Begonia chitoensis* | AJ491202 |
| Angiosperm | Begoniaceae | *Begonia chitoensis* | AJ491204 |
| Angiosperm | Begoniaceae | *Begonia chitoensis* | AJ491206 |
| Angiosperm | Begoniaceae | *Begonia chloroneura* | AF485134 |
| Angiosperm | Begoniaceae | *Begonia comorensis* | AY429326 |
| Angiosperm | Begoniaceae | *Begonia convolvulacea* | AF485178 |
| Angiosperm | Begoniaceae | *Begonia crassirostris* | AF485103 |
| Angiosperm | Begoniaceae | *Begonia crassirostris* | AJ491207 |
| Angiosperm | Begoniaceae | *Begonia cubensis* | AF485169 |
| Angiosperm | Begoniaceae | *Begonia dipetala* | AF469124 |
| Angiosperm | Begoniaceae | *Begonia dregei* | AF469125 |
| Angiosperm | Begoniaceae | *Begonia dregei* | AY429336 |
| Angiosperm | Begoniaceae | *Begonia duncan-thomasii* | AF485224 |
| Angiosperm | Begoniaceae | *Begonia ebolowensis* | AY429309 |
| Angiosperm | Begoniaceae | *Begonia echinosepala* | AF485187 |
| Angiosperm | Begoniaceae | *Begonia egregia* | AF485184 |
| Angiosperm | Begoniaceae | *Begonia eminii* | AY429316 |
| Angiosperm | Begoniaceae | *Begonia fallax* | AF485140 |
| Angiosperm | Begoniaceae | *Begonia foliosa* | AF485166 |
| Angiosperm | Begoniaceae | *Begonia formosana* | AJ491232 |
| Angiosperm | Begoniaceae | *Begonia formosana* | AJ491233 |
| Angiosperm | Begoniaceae | *Begonia formosana* | AJ491235 |
| Angiosperm | Begoniaceae | *Begonia formosana* | AJ491236 |
| Angiosperm | Begoniaceae | *Begonia formosana* | AJ491237 |
| Angiosperm | Begoniaceae | *Begonia formosana* | AJ491238 |
| Angiosperm | Begoniaceae | *Begonia formosana* | AJ491239 |
| Angiosperm | Begoniaceae | *Begonia formosana* | AJ491240 |
| Angiosperm | Begoniaceae | *Begonia formosana* | AJ491241 |
| Angiosperm | Begoniaceae | *Begonia francoisii* | AF485234 |
| Angiosperm | Begoniaceae | *Begonia fuchsioides* | AF485163 |
| Angiosperm | Begoniaceae | *Begonia furfuracea* | AY429325 |
| Angiosperm | Begoniaceae | *Begonia fusialata* | AY429320 |
| Angiosperm | Begoniaceae | *Begonia gabonensis* | AF485242 |
| Angiosperm | Begoniaceae | *Begonia gabonensis* | AY429313 |
| Angiosperm | Begoniaceae | *Begonia geranioides* | AF469120 |
| Angiosperm | Begoniaceae | *Begonia glabra* | AF485176 |
| Angiosperm | Begoniaceae | *Begonia gracilis* | AF485202 |
| Angiosperm | Begoniaceae | *Begonia grandis* subsp. *grandis* | AF485089 |
| Angiosperm | Begoniaceae | *Begonia grandis* subsp. *holostyla* | AF485088 |
| Angiosperm | Begoniaceae | *Begonia halconensis* | AF485106 |
| Angiosperm | Begoniaceae | *Begonia hatacoa* | AF485111 |
| Angiosperm | Begoniaceae | *Begonia holtonis* | AF485162 |
| Angiosperm | Begoniaceae | *Begonia homonyma* | AF469119 |
| Angiosperm | Begoniaceae | *Begonia homonyma* | AY429334 |
| Angiosperm | Begoniaceae | *Begonia horticola* | AF485241 |
| Angiosperm | Begoniaceae | *Begonia imperialis* | AF485204 |
| Angiosperm | Begoniaceae | *Begonia incarnata* | AF485173 |
| Angiosperm | Begoniaceae | *Begonia involucrata* | AF485209 |
| Angiosperm | Begoniaceae | *Begonia iucunda* | AF485249 |
| Angiosperm | Begoniaceae | *Begonia kingiana* | AF485139 |
| Angiosperm | Begoniaceae | *Begonia labordei* | AF485122 |
| Angiosperm | Begoniaceae | *Begonia lobata* | AF485185 |
| Angiosperm | Begoniaceae | *Begonia longicarpa* | AF485109 |
| Angiosperm | Begoniaceae | *Begonia longifolia* | AF485105 |
| Angiosperm | Begoniaceae | *Begonia longipetiolata* | AF485245 |
| Angiosperm | Begoniaceae | *Begonia lukuana* | AJ491243 |
| Angiosperm | Begoniaceae | *Begonia lukuana* | AJ491244 |
| Angiosperm | Begoniaceae | *Begonia luxurians* | AF485192 |
| Angiosperm | Begoniaceae | *Begonia luxurians* | AF485194 |
| Angiosperm | Begoniaceae | *Begonia luxurians* | AF485196 |
| Angiosperm | Begoniaceae | *Begonia madecassa* | AF485230 |
| Angiosperm | Begoniaceae | *Begonia malachosticta* | AF485156 |
| Angiosperm | Begoniaceae | *Begonia mananjebensis* | AF485231 |
| Angiosperm | Begoniaceae | *Begonia manicata* | AF485211 |
| Angiosperm | Begoniaceae | *Begonia masoniana* var. *maculata* | AF485125 |
| Angiosperm | Begoniaceae | *Begonia masoniana* var. *maculata* | AF485127 |
| Angiosperm | Begoniaceae | *Begonia masoniana* var. *maculata* | AF485128 |
| Angiosperm | Begoniaceae | *Begonia meridensis* | AF485161 |
| Angiosperm | Begoniaceae | *Begonia molleri* | AF485240 |
| Angiosperm | Begoniaceae | *Begonia molleri* | AY429323 |
| Angiosperm | Begoniaceae | *Begonia nantoensis* | AJ491245 |
| Angiosperm | Begoniaceae | *Begonia nantoensis* | AJ491246 |
| Angiosperm | Begoniaceae | *Begonia nossibea* | AF485233 |
| Angiosperm | Begoniaceae | *Begonia obliqua* | AF485170 |
| Angiosperm | Begoniaceae | *Begonia odorata* | AF485168 |
| Angiosperm | Begoniaceae | *Begonia olbia* | AF485201 |
| Angiosperm | Begoniaceae | *Begonia oxysperma* | AF485131 |
| Angiosperm | Begoniaceae | *Begonia palmata* | AF485114 |
| Angiosperm | Begoniaceae | *Begonia palmata* | AJ491247 |
| Angiosperm | Begoniaceae | *Begonia palmata* | AJ491248 |
| Angiosperm | Begoniaceae | *Begonia peltata* | AF485210 |
| Angiosperm | Begoniaceae | *Begonia polygonoides* | AY429322 |
| Angiosperm | Begoniaceae | *Begonia potamophila* | AF485219 |
| Angiosperm | Begoniaceae | *Begonia preussii* | AY429321 |
| Angiosperm | Begoniaceae | *Begonia rajah* | AF485136 |
| Angiosperm | Begoniaceae | *Begonia reniformis* | AF485180 |
| Angiosperm | Begoniaceae | *Begonia reniformis* | AF485181 |
| Angiosperm | Begoniaceae | *Begonia roxburghii* | AF485092 |
| Angiosperm | Begoniaceae | *Begonia salaziensis* | AF485232 |
| Angiosperm | Begoniaceae | *Begonia samhaensis* | AF469122 |
| Angiosperm | Begoniaceae | *Begonia scapigera* | AF485223 |
| Angiosperm | Begoniaceae | *Begonia sonderiana* | AF485216 |
| Angiosperm | Begoniaceae | *Begonia taipeiensis* | AJ287286 |
| Angiosperm | Begoniaceae | *Begonia taipeiensis* | AJ287288 |
| Angiosperm | Begoniaceae | *Begonia taiwaniana* | AJ491251 |
| Angiosperm | Begoniaceae | *Begonia taiwaniana* | AJ491252 |
| Angiosperm | Begoniaceae | *Begonia tayabensis* | AF485135 |
| Angiosperm | Begoniaceae | *Begonia ulmifolia* | AF485179 |
| Angiosperm | Begoniaceae | *Begonia valida* | AF485182 |
| Angiosperm | Begoniaceae | *Begonia violifolia* | AF485197 |
| Angiosperm | Berberidaceae | *Berberis aquifolium* | AY383434 |
| Angiosperm | Berberidaceae | *Berberis bidentata* | AF403368 |
| Angiosperm | Berberidaceae | *Berberis bidentata* | AF403383 |
| Angiosperm | Berberidaceae | *Berberis buxifolia* | AF403376 |
| Angiosperm | Berberidaceae | *Berberis comberi* | AF403380 |
| Angiosperm | Berberidaceae | *Berberis croatica* | EF488083 |
| Angiosperm | Berberidaceae | *Berberis croatica* | EF488084 |
| Angiosperm | Berberidaceae | *Berberis croatica* | EF488085 |
| Angiosperm | Berberidaceae | *Berberis grevilleana* | AF403379 |
| Angiosperm | Berberidaceae | *Berberis heterophylla* | AF403374 |
| Angiosperm | Berberidaceae | *Berberis linearifolia* | AF403382 |
| Angiosperm | Berberidaceae | *Berberis microphylla* | AF403377 |
| Angiosperm | Berberidaceae | *Berberis serrato-dentata* | AF403370 |
| Angiosperm | Berberidaceae | *Berberis silva-taroucana* | AF328969 |
| Angiosperm | Berberidaceae | *Epimedium acuminatum* | AY362423 |
| Angiosperm | Berberidaceae | *Epimedium alpinum* | AY362422 |
| Angiosperm | Berberidaceae | *Epimedium brevicornu* | AY362429 |
| Angiosperm | Berberidaceae | *Epimedium chlorandrum* | AY362418 |
| Angiosperm | Berberidaceae | *Epimedium davidii* | AY362414 |
| Angiosperm | Berberidaceae | *Epimedium diphyllum* | AY362409 |
| Angiosperm | Berberidaceae | *Epimedium dolichostemon* | AY362424 |
| Angiosperm | Berberidaceae | *Epimedium epsteinii* | AY362417 |
| Angiosperm | Berberidaceae | *Epimedium franchetii* | AY362412 |
| Angiosperm | Berberidaceae | *Epimedium grandiflorum* | AY362410 |
| Angiosperm | Berberidaceae | *Epimedium koreanum* | AY362413 |
| Angiosperm | Berberidaceae | *Epimedium leptorrhizum* | AY362419 |
| Angiosperm | Berberidaceae | *Epimedium ogisui* | AY362425 |
| Angiosperm | Berberidaceae | *Epimedium pauciflorum* | AY362428 |
| Angiosperm | Berberidaceae | *Epimedium pubescens* | AY362416 |
| Angiosperm | Berberidaceae | *Epimedium pubigerum* | AY362408 |
| Angiosperm | Berberidaceae | *Epimedium rhizomatosum* | AY362415 |
| Angiosperm | Berberidaceae | *Epimedium sagittatum* | AY362427 |
| Angiosperm | Berberidaceae | *Epimedium simplicifolium* | AY362420 |
| Angiosperm | Berberidaceae | *Epimedium wushanense* | AY362421 |
| Angiosperm | Berberidaceae | *Nandina domestica* | AY362430 |
| Angiosperm | Betulaceae | *Betula humilis* | AJ783643 |
| Angiosperm | Betulaceae | *Betula insignis* | AJ783645 |
| Angiosperm | Betulaceae | *Betula nigra* | AJ783646 |
| Angiosperm | Betulaceae | *Betula pendula* | AJ006445 |
| Angiosperm | Betulaceae | *Betula pendula* | AM503889 |
| Angiosperm | Betulaceae | *Betula populifolia* | AJ783644 |
| Angiosperm | Betulaceae | *Corylus chinensis* | AF297359 |
| Angiosperm | Betulaceae | *Corylus cornuta* var. *californica* | AF297342 |
| Angiosperm | Betulaceae | *Corylus ferox* var. *tibetica* | AF297361 |
| Angiosperm | Betulaceae | *Corylus jacquemontii* | AF297360 |
| Angiosperm | Bignoniaceae | *Campsis radicans* | DQ515201 |
| Angiosperm | Bignoniaceae | *Oroxylum indicum* | FJ606747 |
| Angiosperm | Bombacaceae | *Bombax malabaricum* | AF460192 |
| Angiosperm | Bombacaceae | *Bombax malabaricum* | DQ826447 |
| Angiosperm | Bombacaceae | *Ceiba pentandra* | AY635501 |
| Angiosperm | Bombacaceae | *Ceiba pentandra* | DQ284854 |
| Angiosperm | Bombacaceae | *Ceiba pentandra* | EF432374 |
| Angiosperm | Bombacaceae | *Ceiba pentandra* | EF432375 |
| Angiosperm | Bombacaceae | *Pachira aquatica* | AF028522 |
| Angiosperm | Boraginaceae | *Arnebia euchroma* | EF199848 |
| Angiosperm | Boraginaceae | *Arnebia euchroma* | EF199860 |
| Angiosperm | Boraginaceae | *Arnebia guttata* | EF199862 |
| Angiosperm | Boraginaceae | *Arnebia szechenyi* | EF199863 |
| Angiosperm | Boraginaceae | *Cynoglossum amabile* | DQ248971 |
| Angiosperm | Boraginaceae | *Ehretia ovalifolia* | AF091156 |
| Angiosperm | Boraginaceae | *Lithospermum erythrorhizon* | EF199861 |
| Angiosperm | Brassicaceae | *Brassica balearica* | AF263402 |
| Angiosperm | Brassicaceae | *Brassica juncea* | AF128093 |
| Angiosperm | Brassicaceae | *Brassica juncea* | AF128094 |
| Angiosperm | Brassicaceae | *Brassica napus* | D10840 |
| Angiosperm | Brassicaceae | *Brassica nigra* | AF128102 |
| Angiosperm | Brassicaceae | *Brassica nigra* | AF128103 |
| Angiosperm | Brassicaceae | *Brassica oleracea* | AY833603 |
| Angiosperm | Brassicaceae | *Brassica oleracea* var. *alboglabra* | AF128101 |
| Angiosperm | Brassicaceae | *Brassica oleracea* var. *botrytis* | AF128099 |
| Angiosperm | Brassicaceae | *Brassica oleracea* var. *botrytis* | AF128100 |
| Angiosperm | Brassicaceae | *Brassica rapa* subsp. *chinensis* | AF128098 |
| Angiosperm | Brassicaceae | *Cardamine alpina* | AM905716 |
| Angiosperm | Brassicaceae | *Cardamine concatenata* | DQ005988 |
| Angiosperm | Brassicaceae | *Cardamine flexuosa* | AM905717 |
| Angiosperm | Brassicaceae | *Cardamine flexuosa* | EF523395 |
| Angiosperm | Brassicaceae | *Cardamine flexuosa* | EF523401 |
| Angiosperm | Brassicaceae | *Cardamine flexuosa* | EF523402 |
| Angiosperm | Brassicaceae | *Cardamine impatiens* | AM905720 |
| Angiosperm | Brassicaceae | *Cardamine scutata* var. *formosana* | AF128107 |
| Angiosperm | Brassicaceae | *Draba nemorosa* | DQ467361 |
| Angiosperm | Brassicaceae | *Draba nivalis* | DQ467477 |
| Angiosperm | Brassicaceae | *Draba nivalis* | DQ467598 |
| Angiosperm | Brassicaceae | *Draba norvegica* | DQ467605 |
| Angiosperm | Brassicaceae | *Draba oblongata* | DQ467466 |
| Angiosperm | Brassicaceae | *Draba ogilviensis* | DQ467600 |
| Angiosperm | Brassicaceae | *Erysimum capitatum* | AY254534 |
| Angiosperm | Brassicaceae | *Erysimum cheiranthoides* | DQ005989 |
| Angiosperm | Brassicaceae | *Erysimum cheiri* | AM905722 |
| Angiosperm | Brassicaceae | *Erysimum cheiri* | AY254538 |
| Angiosperm | Brassicaceae | *Isatis indigotica* | AF384104 |
| Angiosperm | Brassicaceae | *Isatis tinctoria* | AF384105 |
| Angiosperm | Brassicaceae | *Lepidium africanum* | AY254529 |
| Angiosperm | Brassicaceae | *Lepidium apetalum* | DQ310525 |
| Angiosperm | Brassicaceae | *Lepidium draba* | EF367913 |
| Angiosperm | Brassicaceae | *Lepidium montanum* | EF367952 |
| Angiosperm | Brassicaceae | *Lepidium montanum* | EF367953 |
| Angiosperm | Brassicaceae | *Lepidium montanum* | EF367956 |
| Angiosperm | Brassicaceae | *Lepidium montanum* | EF367957 |
| Angiosperm | Brassicaceae | *Lepidium montanum* | EF367961 |
| Angiosperm | Brassicaceae | *Lepidium montanum* | EF367963 |
| Angiosperm | Brassicaceae | *Lepidium montanum* | EF367966 |
| Angiosperm | Brassicaceae | *Lepidium montanum* | EF367967 |
| Angiosperm | Brassicaceae | *Lepidium montanum* | EF367968 |
| Angiosperm | Brassicaceae | *Lepidium perfoliatum* | EF368007 |
| Angiosperm | Brassicaceae | *Lepidium phlebopetalum* | AY254528 |
| Angiosperm | Brassicaceae | *Lepidium sisymbrioides* | DQ997562 |
| Angiosperm | Brassicaceae | *Lepidium sisymbrioides* | DQ997565 |
| Angiosperm | Brassicaceae | *Lepidium solandri* | DQ997566 |
| Angiosperm | Brassicaceae | *Lepidium virginicum* | AF128109 |
| Angiosperm | Brassicaceae | *Raphanus sativus* | AF128104 |
| Angiosperm | Brassicaceae | *Raphanus sativus* | AF128105 |
| Angiosperm | Brassicaceae | *Raphanus sativus* | AY746461 |
| Angiosperm | Brassicaceae | *Rorippa austriaca* | AM905725 |
| Angiosperm | Brassicaceae | *Rorippa indica* | AF128108 |
| Angiosperm | Bretschneideraceae | *Bretschneidera sinensis* | AF254758 |
| Angiosperm | Burseraceae | *Boswellia hildebrandtii* | AF080063 |
| Angiosperm | Burseraceae | *Boswellia hildebrandtii* | AF445881 |
| Angiosperm | Burseraceae | *Boswellia sacra* | AF445880 |
| Angiosperm | Burseraceae | *Canarium album* | DQ517524 |
| Angiosperm | Burseraceae | *Commiphora mollis* | AF445876 |
| Angiosperm | Burseraceae | *Commiphora monstruosa* | AF080004 |
| Angiosperm | Burseraceae | *Commiphora wightii* | EU419976 |
| Angiosperm | Burseraceae | *Commiphora wightii* | EU419977 |
| Angiosperm | Burseraceae | *Commiphora wightii* | EU419978 |
| Angiosperm | Burseraceae | *Commiphora wightii* | EU419979 |
| Angiosperm | Burseraceae | *Commiphora wightii* | EU419980 |
| Angiosperm | Burseraceae | *Commiphora wightii* | EU419981 |
| Angiosperm | Burseraceae | *Commiphora wightii* | EU419982 |
| Angiosperm | Burseraceae | *Commiphora wightii* | EU419983 |
| Angiosperm | Burseraceae | *Commiphora wightii* | EU419984 |
| Angiosperm | Burseraceae | *Commiphora wightii* | EU419985 |
| Angiosperm | Buxaceae | *Buxus balearica* | AF245423 |
| Angiosperm | Buxaceae | *Buxus glomerata* | AF245426 |
| Angiosperm | Buxaceae | *Buxus gonoclada* | AF245427 |
| Angiosperm | Buxaceae | *Buxus harlandii* | AF245410 |
| Angiosperm | Buxaceae | *Buxus henryi* | AF245409 |
| Angiosperm | Buxaceae | *Buxus hildebrandtii* | AF245415 |
| Angiosperm | Buxaceae | *Buxus liukiuensis* | AF245428 |
| Angiosperm | Buxaceae | *Buxus macowanii* | AF245411 |
| Angiosperm | Buxaceae | *Buxus microphylla* var. *japonica* | AF245412 |
| Angiosperm | Buxaceae | *Buxus riparia* | AF245413 |
| Angiosperm | Buxaceae | *Buxus sinica* | AF245414 |
| Angiosperm | Cactaceae | *Echinopsis chiloensis* | AY064346 |
| Angiosperm | Campanulaceae | *Adenophora remotiflora* | AY548197 |
| Angiosperm | Campanulaceae | *Adenophora trachelioides* | AY548196 |
| Angiosperm | Campanulaceae | *Adenophora triphylla* | AY548194 |
| Angiosperm | Campanulaceae | *Adenophora triphylla* var. *japonica* | AY548193 |
| Angiosperm | Campanulaceae | *Codonopsis javanica* subsp. *japonica* | DQ889459 |
| Angiosperm | Campanulaceae | *Codonopsis kawakamii* | DQ810274 |
| Angiosperm | Campanulaceae | *Codonopsis lanceolata* | AY548195 |
| Angiosperm | Campanulaceae | *Codonopsis nervosa* | AF136237 |
| Angiosperm | Campanulaceae | *Codonopsis pilosula* | EF190460 |
| Angiosperm | Campanulaceae | *Codonopsis pilosula* var. *modesta* | AF134859 |
| Angiosperm | Campanulaceae | *Codonopsis tangshen* | AF134861 |
| Angiosperm | Campanulaceae | *Codonopsis tangshen* | EF190462 |
| Angiosperm | Campanulaceae | *Heterotoma cordifolia* | AY350624 |
| Angiosperm | Campanulaceae | *Lobelia anatina* | AY350628 |
| Angiosperm | Campanulaceae | *Lobelia calcarata* | AY350623 |
| Angiosperm | Campanulaceae | *Lobelia cardinalis* | AY350630 |
| Angiosperm | Campanulaceae | *Lobelia fenestralis* | AY350634 |
| Angiosperm | Campanulaceae | *Lobelia laxiflora* | AY350631 |
| Angiosperm | Campanulaceae | *Lobelia macrodon* | AY568734 |
| Angiosperm | Campanulaceae | *Lobelia mcvaughii* | AY350627 |
| Angiosperm | Campanulaceae | *Lobelia nana* | AY350629 |
| Angiosperm | Campanulaceae | *Lobelia polyphylla* | AY350633 |
| Angiosperm | Campanulaceae | *Lobelia siphilitica* | DQ006015 |
| Angiosperm | Campanulaceae | *Lobelia tenera* | AF054938 |
| Angiosperm | Campanulaceae | *Lobelia tupa* | AY350632 |
| Angiosperm | Campanulaceae | *Lobelia volcanica* | AY350625 |
| Angiosperm | Campanulaceae | *Platycodon grandiflorum* | AF134863 |
| Angiosperm | Campanulaceae | *Pratia angulata* | AY568727 |
| Angiosperm | Campanulaceae | *Pratia angulata* | AY568731 |
| Angiosperm | Campanulaceae | *Pratia arenaria* | AY568726 |
| Angiosperm | Campanulaceae | *Pratia perpusilla* | AY568729 |
| Angiosperm | Cannabaceae | *Cannabis sativa* | FJ572045 |
| Angiosperm | Cannabaceae | *Humulus lupulus* | DQ005990 |
| Angiosperm | Cannabaceae | *Humulus lupulus* | EF136401 |
| Angiosperm | Cannabaceae | *Humulus lupulus* | Y12588 |
| Angiosperm | Cannaceae | *Canna paniculata* | AY673069 |
| Angiosperm | Caprifoliaceae | *Lonicera maackii* | DQ005991 |
| Angiosperm | Caprifoliaceae | *Lonicera xylosteum* | AM503886 |
| Angiosperm | Caprifoliaceae | *Sambucus adnata* | DQ009613 |
| Angiosperm | Caprifoliaceae | *Sambucus adnata* | U88210 |
| Angiosperm | Caprifoliaceae | *Sambucus africana* | U88195 |
| Angiosperm | Caprifoliaceae | *Sambucus australasica* | U41381 |
| Angiosperm | Caprifoliaceae | *Sambucus australis* | U88196 |
| Angiosperm | Caprifoliaceae | *Sambucus caerulea* | DQ521255 |
| Angiosperm | Caprifoliaceae | *Sambucus caerulea* | U88197 |
| Angiosperm | Caprifoliaceae | *Sambucus canadensis* | AY265157 |
| Angiosperm | Caprifoliaceae | *Sambucus canadensis* | U88199 |
| Angiosperm | Caprifoliaceae | *Sambucus ebulus* | DQ521256 |
| Angiosperm | Caprifoliaceae | *Sambucus ebulus* | U88200 |
| Angiosperm | Caprifoliaceae | *Sambucus gaudichaudiana* | U41382 |
| Angiosperm | Caprifoliaceae | *Sambucus javanica* | DQ521257 |
| Angiosperm | Caprifoliaceae | *Sambucus javanica* | U88201 |
| Angiosperm | Caprifoliaceae | *Sambucus maderensis* | U88202 |
| Angiosperm | Caprifoliaceae | *Sambucus nigra* | DQ521258 |
| Angiosperm | Caprifoliaceae | *Sambucus nigra* | U88204 |
| Angiosperm | Caprifoliaceae | *Sambucus peruviana* | U88205 |
| Angiosperm | Caprifoliaceae | *Sambucus racemosa* | AY236171 |
| Angiosperm | Caprifoliaceae | *Sambucus racemosa* | AY265158 |
| Angiosperm | Caprifoliaceae | *Sambucus racemosa* | DQ521252 |
| Angiosperm | Caprifoliaceae | *Sambucus racemosa* | U88207 |
| Angiosperm | Caprifoliaceae | *Sambucus sieboldiana* | U88559 |
| Angiosperm | Caprifoliaceae | *Sambucus wightiana* | U88208 |
| Angiosperm | Caprifoliaceae | *Viburnum acerifolium* | DQ005992 |
| Angiosperm | Caprifoliaceae | *Viburnum prunifolium* | DQ005993 |
| Angiosperm | Caprifoliaceae | *Weigela maximowiczii* | AB055087 |
| Angiosperm | Caryophyllaceae | *Dianthus amurensis* | AY594318 |
| Angiosperm | Caryophyllaceae | *Dianthus superbus* | AY594315 |
| Angiosperm | Caryophyllaceae | *Dianthus sylvestris* | AY594317 |
| Angiosperm | Caryophyllaceae | *Myosoton aquaticum* | AY594303 |
| Angiosperm | Caryophyllaceae | *Pseudostellaria heterophylla* | EF121855 |
| Angiosperm | Caryophyllaceae | *Pseudostellaria heterophylla* | EF197885 |
| Angiosperm | Caryophyllaceae | *Pseudostellaria heterophylla* | EF197886 |
| Angiosperm | Caryophyllaceae | *Silene acaulis* | AY116473 |
| Angiosperm | Caryophyllaceae | *Silene ajanensis* | AJ831780 |
| Angiosperm | Caryophyllaceae | *Silene alexandri* | EF060222 |
| Angiosperm | Caryophyllaceae | *Silene brevistaminea* | EF060235 |
| Angiosperm | Caryophyllaceae | *Silene caroliniana* subsp. *caroliniana* | AY116474 |
| Angiosperm | Caryophyllaceae | *Silene caroliniana* subsp. *wherryi* | AY116481 |
| Angiosperm | Caryophyllaceae | *Silene falcata* | EF060225 |
| Angiosperm | Caryophyllaceae | *Silene hawaiiensis* | EF060218 |
| Angiosperm | Caryophyllaceae | *Silene hayekiana* | AY594307 |
| Angiosperm | Caryophyllaceae | *Silene involucrata* | AJ831787 |
| Angiosperm | Caryophyllaceae | *Silene involucrata* | AJ831788 |
| Angiosperm | Caryophyllaceae | *Silene latifolia* subsp. *alba* | AY594308 |
| Angiosperm | Caryophyllaceae | *Silene latifolia* subsp. *alba* | DQ005994 |
| Angiosperm | Caryophyllaceae | *Silene macrostyla* | EF060227 |
| Angiosperm | Caryophyllaceae | *Silene nemoralis* | EF060230 |
| Angiosperm | Caryophyllaceae | *Silene ostenfeldii* | AJ831791 |
| Angiosperm | Caryophyllaceae | *Silene otites* | EF060233 |
| Angiosperm | Caryophyllaceae | *Silene ovata* | AY116475 |
| Angiosperm | Caryophyllaceae | *Silene perlmanii* | EF060220 |
| Angiosperm | Caryophyllaceae | *Silene polypetala* | AY116480 |
| Angiosperm | Caryophyllaceae | *Silene regia* | AY116476 |
| Angiosperm | Caryophyllaceae | *Silene rotundifolia* | AY116477 |
| Angiosperm | Caryophyllaceae | *Silene saxifraga* | EF060234 |
| Angiosperm | Caryophyllaceae | *Silene stellata* | AY116472 |
| Angiosperm | Caryophyllaceae | *Silene stenophylla* | X86872 |
| Angiosperm | Caryophyllaceae | *Silene subciliata* | AY116471 |
| Angiosperm | Caryophyllaceae | *Silene subciliata* | AY116478 |
| Angiosperm | Caryophyllaceae | *Silene uralensis* | AJ831781 |
| Angiosperm | Caryophyllaceae | *Silene uralensis* | AJ831782 |
| Angiosperm | Caryophyllaceae | *Silene uralensis* | AJ831783 |
| Angiosperm | Caryophyllaceae | *Silene uralensis* | AJ831784 |
| Angiosperm | Caryophyllaceae | *Silene uralensis* | AJ831785 |
| Angiosperm | Caryophyllaceae | *Silene virginica* | AY116479 |
| Angiosperm | Caryophyllaceae | *Silene viscosa* | AY594306 |
| Angiosperm | Caryophyllaceae | *Stellaria alsine* | AY438312 |
| Angiosperm | Caryophyllaceae | *Stellaria graminea* | AY594304 |
| Angiosperm | Caryophyllaceae | *Stellaria vestita* | EF160065 |
| Angiosperm | Casuarinaceae | *Casuarina equisetifolia* subsp. *equisetifolia* | AY864057 |
| Angiosperm | Celastraceae | *Celastrus angulatus* | EU328759 |
| Angiosperm | Celastraceae | *Celastrus orbiculatus* | EU328766 |
| Angiosperm | Celastraceae | *Celastrus rosthornianus* | EU328762 |
| Angiosperm | Celastraceae | *Celastrus scandens* | DQ005996 |
| Angiosperm | Celastraceae | *Celastrus scandens* | EU328761 |
| Angiosperm | Celastraceae | *Euonymus alatus* | EU328755 |
| Angiosperm | Celastraceae | *Maytenus boaria* | EU328753 |
| Angiosperm | Celastraceae | *Maytenus cordata* | EU328742 |
| Angiosperm | Celastraceae | *Maytenus cunninghamii* | EU328770 |
| Angiosperm | Celastraceae | *Maytenus disticha* | EU328754 |
| Angiosperm | Celastraceae | *Maytenus fournieri* | EU328720 |
| Angiosperm | Celastraceae | *Maytenus silvestris* | EU328721 |
| Angiosperm | Celastraceae | *Maytenus undata* | EU328743 |
| Angiosperm | Celastraceae | *Maytenus undata* | EU328744 |
| Angiosperm | Cercidiphyllaceae | *Cercidiphyllum japonicum* | AF147756 |
| Angiosperm | Chenopodiaceae | *Axyris hybrida* | AM849226 |
| Angiosperm | Chenopodiaceae | *Axyris prostrata* | AM849228 |
| Angiosperm | Chenopodiaceae | *Chenopodium ambiguum* | EU812823 |
| Angiosperm | Chenopodiaceae | *Chenopodium ambrosioides* | DQ005963 |
| Angiosperm | Chenopodiaceae | *Chenopodium detestans* | EU812824 |
| Angiosperm | Chenopodiaceae | *Kochia americana* | AY489210 |
| Angiosperm | Chenopodiaceae | *Kochia densiflora* | AY489212 |
| Angiosperm | Chenopodiaceae | *Kochia saxicola* | AY489217 |
| Angiosperm | Chenopodiaceae | *Kochia stellaris* | AY489219 |
| Angiosperm | Chloranthaceae | *Chloranthus holostegius* | AF280749 |
| Angiosperm | Chloranthaceae | *Sarcandra glabra* | AF203635 |
| Angiosperm | Clusiaceae | *Garcinia afzelii* | EU128424 |
| Angiosperm | Clusiaceae | *Garcinia afzelii* | EU128425 |
| Angiosperm | Clusiaceae | *Garcinia afzelii* | EU128426 |
| Angiosperm | Clusiaceae | *Garcinia afzelii* | EU128430 |
| Angiosperm | Clusiaceae | *Garcinia amplexicaulis* | EU128480 |
| Angiosperm | Clusiaceae | *Garcinia asterandra* | EU128478 |
| Angiosperm | Clusiaceae | *Garcinia atroviridis* | EU128374 |
| Angiosperm | Clusiaceae | *Garcinia cantleyana* var. *grandifolia* | EU128477 |
| Angiosperm | Clusiaceae | *Garcinia cataractalis* | EU128476 |
| Angiosperm | Clusiaceae | *Garcinia chapieleri* | EU128474 |
| Angiosperm | Clusiaceae | *Garcinia conrauana* | EU128470 |
| Angiosperm | Clusiaceae | *Garcinia conrauana* | EU128471 |
| Angiosperm | Clusiaceae | *Garcinia conrauana* | EU128472 |
| Angiosperm | Clusiaceae | *Garcinia conrauana* | EU128473 |
| Angiosperm | Clusiaceae | *Garcinia dumosa* | EU128464 |
| Angiosperm | Clusiaceae | *Garcinia dumosa* | EU128467 |
| Angiosperm | Clusiaceae | *Garcinia eugenifolia* | EU128462 |
| Angiosperm | Clusiaceae | *Garcinia fruticosa* | EU128461 |
| Angiosperm | Clusiaceae | *Garcinia gnetoides* | EU128460 |
| Angiosperm | Clusiaceae | *Garcinia hombroniana* | EU128454 |
| Angiosperm | Clusiaceae | *Garcinia hombroniana* | EU128455 |
| Angiosperm | Clusiaceae | *Garcinia hombroniana* | EU128456 |
| Angiosperm | Clusiaceae | *Garcinia hombroniana* | EU128457 |
| Angiosperm | Clusiaceae | *Garcinia intermedia* | AJ312605 |
| Angiosperm | Clusiaceae | *Garcinia intermedia* | EU128444 |
| Angiosperm | Clusiaceae | *Garcinia intermedia* | EU128445 |
| Angiosperm | Clusiaceae | *Garcinia kola* | EU128453 |
| Angiosperm | Clusiaceae | *Garcinia latissima* | EU128451 |
| Angiosperm | Clusiaceae | *Garcinia livingstonei* | EU128450 |
| Angiosperm | Clusiaceae | *Garcinia lucida* | EU128449 |
| Angiosperm | Clusiaceae | *Garcinia macrophylla* | EU128439 |
| Angiosperm | Clusiaceae | *Garcinia macrophylla* | EU128442 |
| Angiosperm | Clusiaceae | *Garcinia macrophylla* | EU128446 |
| Angiosperm | Clusiaceae | *Garcinia macrophylla* | EU128447 |
| Angiosperm | Clusiaceae | *Garcinia macrophylla* | EU128448 |
| Angiosperm | Clusiaceae | *Garcinia madruno* | AJ509215 |
| Angiosperm | Clusiaceae | *Garcinia madruno* | AY635533 |
| Angiosperm | Clusiaceae | *Garcinia malaccensis* | EU128437 |
| Angiosperm | Clusiaceae | *Garcinia mangostana* | AJ509214 |
| Angiosperm | Clusiaceae | *Garcinia mangostana* | EU128432 |
| Angiosperm | Clusiaceae | *Garcinia mangostana* | EU128433 |
| Angiosperm | Clusiaceae | *Garcinia mangostana* | EU128434 |
| Angiosperm | Clusiaceae | *Garcinia mannii* | EU128428 |
| Angiosperm | Clusiaceae | *Garcinia opaca* var. *minor* | EU128421 |
| Angiosperm | Clusiaceae | *Garcinia ovalifolia* | EU128413 |
| Angiosperm | Clusiaceae | *Garcinia ovalifolia* | EU128414 |
| Angiosperm | Clusiaceae | *Garcinia ovalifolia* | EU128415 |
| Angiosperm | Clusiaceae | *Garcinia ovalifolia* | EU128416 |
| Angiosperm | Clusiaceae | *Garcinia ovalifolia* | EU128417 |
| Angiosperm | Clusiaceae | *Garcinia ovalifolia* | EU128418 |
| Angiosperm | Clusiaceae | *Garcinia ovalifolia* | EU128419 |
| Angiosperm | Clusiaceae | *Garcinia ovalifolia* | EU128420 |
| Angiosperm | Clusiaceae | *Garcinia pauciflora* | EU128411 |
| Angiosperm | Clusiaceae | *Garcinia porrecta* | EU128410 |
| Angiosperm | Clusiaceae | *Garcinia prainiana* | EU128409 |
| Angiosperm | Clusiaceae | *Garcinia punctata* | EU128391 |
| Angiosperm | Clusiaceae | *Garcinia rigida* | EU128408 |
| Angiosperm | Clusiaceae | *Garcinia rostrata* | EU128407 |
| Angiosperm | Clusiaceae | *Garcinia rubriflora* | EU128406 |
| Angiosperm | Clusiaceae | *Garcinia scortechinii* | EU128401 |
| Angiosperm | Clusiaceae | *Garcinia scortechinii* | EU128402 |
| Angiosperm | Clusiaceae | *Garcinia scortechinii* | EU128403 |
| Angiosperm | Clusiaceae | *Garcinia spicata* | EU128389 |
| Angiosperm | Clusiaceae | *Garcinia spicata* | EU128390 |
| Angiosperm | Clusiaceae | *Garcinia staudtii* | EU128386 |
| Angiosperm | Clusiaceae | *Garcinia staudtii* | EU128387 |
| Angiosperm | Clusiaceae | *Garcinia subelliptica* | EU128381 |
| Angiosperm | Clusiaceae | *Garcinia subelliptica* | EU128382 |
| Angiosperm | Clusiaceae | *Garcinia subelliptica* | EU128383 |
| Angiosperm | Clusiaceae | *Garcinia syzygiifolia* | EU128400 |
| Angiosperm | Clusiaceae | *Garcinia uniflora* | EU128379 |
| Angiosperm | Clusiaceae | *Garcinia urophylla* | EU128378 |
| Angiosperm | Clusiaceae | *Garcinia verrucosa* | EU128376 |
| Angiosperm | Clusiaceae | *Garcinia warrenii* | EU128375 |
| Angiosperm | Clusiaceae | *Hypericum calycinum* | AJ414728 |
| Angiosperm | Clusiaceae | *Hypericum canariense* var. *canariense* | EF015303 |
| Angiosperm | Clusiaceae | *Hypericum canariense* var. *canariense* | EF034041 |
| Angiosperm | Clusiaceae | *Hypericum canariense* var. *floribundum* | EF015304 |
| Angiosperm | Clusiaceae | *Hypericum mutilum* | DQ006013 |
| Angiosperm | Clusiaceae | *Hypericum revolutum* | EF638821 |
| Angiosperm | Combretaceae | *Quisqualis indica* | AF425687 |
| Angiosperm | Combretaceae | *Terminalia arjuna* | AF338255 |
| Angiosperm | Combretaceae | *Terminalia bellirica* | AF334768 |
| Angiosperm | Combretaceae | *Terminalia chebula* | AF334769 |
| Angiosperm | Combretaceae | *Terminalia muelleri* | AF334767 |
| Angiosperm | Convolvulaceae | *Cuscuta abyssinica* | DQ924631 |
| Angiosperm | Convolvulaceae | *Cuscuta africana* | DQ924574 |
| Angiosperm | Convolvulaceae | *Cuscuta americana* | EU330310 |
| Angiosperm | Convolvulaceae | *Cuscuta angulata* | DQ924575 |
| Angiosperm | Convolvulaceae | *Cuscuta applanata* | EF194605 |
| Angiosperm | Convolvulaceae | *Cuscuta applanata* | EU330307 |
| Angiosperm | Convolvulaceae | *Cuscuta approximata* | DQ924595 |
| Angiosperm | Convolvulaceae | *Cuscuta approximata* | DQ924596 |
| Angiosperm | Convolvulaceae | *Cuscuta approximata* | DQ924597 |
| Angiosperm | Convolvulaceae | *Cuscuta approximata* | DQ924598 |
| Angiosperm | Convolvulaceae | *Cuscuta approximata* | DQ924599 |
| Angiosperm | Convolvulaceae | *Cuscuta approximata* | EF202561 |
| Angiosperm | Convolvulaceae | *Cuscuta approximata* subsp. *macranthera* | DQ924600 |
| Angiosperm | Convolvulaceae | *Cuscuta attenuata* | AF348405 |
| Angiosperm | Convolvulaceae | *Cuscuta australis* | DQ211590 |
| Angiosperm | Convolvulaceae | *Cuscuta australis* | EF194667 |
| Angiosperm | Convolvulaceae | *Cuscuta australis* | EF194668 |
| Angiosperm | Convolvulaceae | *Cuscuta australis* | EF194669 |
| Angiosperm | Convolvulaceae | *Cuscuta australis* | EF194670 |
| Angiosperm | Convolvulaceae | *Cuscuta babylonica* | DQ924578 |
| Angiosperm | Convolvulaceae | *Cuscuta babylonica* | DQ924579 |
| Angiosperm | Convolvulaceae | *Cuscuta campestris* | EF194665 |
| Angiosperm | Convolvulaceae | *Cuscuta campestris* | EF194677 |
| Angiosperm | Convolvulaceae | *Cuscuta campestris* | EF194680 |
| Angiosperm | Convolvulaceae | *Cuscuta capitata* | DQ924583 |
| Angiosperm | Convolvulaceae | *Cuscuta capitata* | DQ924584 |
| Angiosperm | Convolvulaceae | *Cuscuta chinensis* | AY558824 |
| Angiosperm | Convolvulaceae | *Cuscuta chinensis* | DQ211587 |
| Angiosperm | Convolvulaceae | *Cuscuta cockerellii* | EF194518 |
| Angiosperm | Convolvulaceae | *Cuscuta compacta* | AY558825 |
| Angiosperm | Convolvulaceae | *Cuscuta compacta* | EF194640 |
| Angiosperm | Convolvulaceae | *Cuscuta compacta* | EU330294 |
| Angiosperm | Convolvulaceae | *Cuscuta coryli* | EU330312 |
| Angiosperm | Convolvulaceae | *Cuscuta decipiens* | EF194718 |
| Angiosperm | Convolvulaceae | *Cuscuta denticulata* | EF194626 |
| Angiosperm | Convolvulaceae | *Cuscuta denticulata* | EF194627 |
| Angiosperm | Convolvulaceae | *Cuscuta denticulata* | EF194628 |
| Angiosperm | Convolvulaceae | *Cuscuta denticulata* | EU330295 |
| Angiosperm | Convolvulaceae | *Cuscuta epithymum* | DQ924606 |
| Angiosperm | Convolvulaceae | *Cuscuta epithymum* | DQ924608 |
| Angiosperm | Convolvulaceae | *Cuscuta epithymum* | DQ924609 |
| Angiosperm | Convolvulaceae | *Cuscuta exaltata* | EU330323 |
| Angiosperm | Convolvulaceae | *Cuscuta foetida* | EF194512 |
| Angiosperm | Convolvulaceae | *Cuscuta foetida* var. *pycnantha* | EF194527 |
| Angiosperm | Convolvulaceae | *Cuscuta friesii* | EF194536 |
| Angiosperm | Convolvulaceae | *Cuscuta glomerata* | EF194644 |
| Angiosperm | Convolvulaceae | *Cuscuta glomerata* | EU330293 |
| Angiosperm | Convolvulaceae | *Cuscuta gracillima* | EF194550 |
| Angiosperm | Convolvulaceae | *Cuscuta grandiflora* | EF194535 |
| Angiosperm | Convolvulaceae | *Cuscuta gronovii* | AY554402 |
| Angiosperm | Convolvulaceae | *Cuscuta gronovii* | EF194637 |
| Angiosperm | Convolvulaceae | *Cuscuta gronovii* | EF194638 |
| Angiosperm | Convolvulaceae | *Cuscuta harperi* | EF194681 |
| Angiosperm | Convolvulaceae | *Cuscuta haussknechtii* | DQ924580 |
| Angiosperm | Convolvulaceae | *Cuscuta indecora* var. *longisepala* | EF194549 |
| Angiosperm | Convolvulaceae | *Cuscuta japonica* | DQ211588 |
| Angiosperm | Convolvulaceae | *Cuscuta japonica* | EU330320 |
| Angiosperm | Convolvulaceae | *Cuscuta kotschyana* | DQ924586 |
| Angiosperm | Convolvulaceae | *Cuscuta kotschyana* | DQ924587 |
| Angiosperm | Convolvulaceae | *Cuscuta kurdica* | DQ924613 |
| Angiosperm | Convolvulaceae | *Cuscuta leptantha* | EU330313 |
| Angiosperm | Convolvulaceae | *Cuscuta lupuliformis* | DQ924570 |
| Angiosperm | Convolvulaceae | *Cuscuta lupuliformis* | EU330321 |
| Angiosperm | Convolvulaceae | *Cuscuta monogyna* | AY554405 |
| Angiosperm | Convolvulaceae | *Cuscuta monogyna* | DQ924569 |
| Angiosperm | Convolvulaceae | *Cuscuta nevadensis* | EF194629 |
| Angiosperm | Convolvulaceae | *Cuscuta nevadensis* | EF194630 |
| Angiosperm | Convolvulaceae | *Cuscuta nitida* | DQ924573 |
| Angiosperm | Convolvulaceae | *Cuscuta nitida* | EF202562 |
| Angiosperm | Convolvulaceae | *Cuscuta nitida* | EU330317 |
| Angiosperm | Convolvulaceae | *Cuscuta nivea* | DQ924614 |
| Angiosperm | Convolvulaceae | *Cuscuta odorata* | EF194514 |
| Angiosperm | Convolvulaceae | *Cuscuta odorata* | EF194515 |
| Angiosperm | Convolvulaceae | *Cuscuta odorata* | EF194519 |
| Angiosperm | Convolvulaceae | *Cuscuta parviflora* var. *elongata* | EF194657 |
| Angiosperm | Convolvulaceae | *Cuscuta pentagona* | AY558821 |
| Angiosperm | Convolvulaceae | *Cuscuta pentagona* | EF194664 |
| Angiosperm | Convolvulaceae | *Cuscuta pentagona* | EF194678 |
| Angiosperm | Convolvulaceae | *Cuscuta pentagona* | EF194679 |
| Angiosperm | Convolvulaceae | *Cuscuta planiflora* | DQ924621 |
| Angiosperm | Convolvulaceae | *Cuscuta planiflora* | DQ924622 |
| Angiosperm | Convolvulaceae | *Cuscuta planiflora* | DQ924623 |
| Angiosperm | Convolvulaceae | *Cuscuta planiflora* | DQ924624 |
| Angiosperm | Convolvulaceae | *Cuscuta planiflora* | DQ924632 |
| Angiosperm | Convolvulaceae | *Cuscuta planiflora* | DQ924633 |
| Angiosperm | Convolvulaceae | *Cuscuta planiflora* | DQ924636 |
| Angiosperm | Convolvulaceae | *Cuscuta planiflora* | DQ924637 |
| Angiosperm | Convolvulaceae | *Cuscuta plattensis* | EF194682 |
| Angiosperm | Convolvulaceae | *Cuscuta polyanthemos* | EF194572 |
| Angiosperm | Convolvulaceae | *Cuscuta potosina* | EU330308 |
| Angiosperm | Convolvulaceae | *Cuscuta potosina* var. *globifera* | EF194600 |
| Angiosperm | Convolvulaceae | *Cuscuta pretoriana* | DQ924626 |
| Angiosperm | Convolvulaceae | *Cuscuta pretoriana* | DQ924627 |
| Angiosperm | Convolvulaceae | *Cuscuta pretoriana* | DQ924628 |
| Angiosperm | Convolvulaceae | *Cuscuta pulchella* | DQ924589 |
| Angiosperm | Convolvulaceae | *Cuscuta pulchella* | DQ924590 |
| Angiosperm | Convolvulaceae | *Cuscuta purpusii* | EF194623 |
| Angiosperm | Convolvulaceae | *Cuscuta rausii* | DQ924615 |
| Angiosperm | Convolvulaceae | *Cuscuta rhodesiana* | DQ924640 |
| Angiosperm | Convolvulaceae | *Cuscuta rugosiceps* | EF194606 |
| Angiosperm | Convolvulaceae | *Cuscuta rugosiceps* | EF194607 |
| Angiosperm | Convolvulaceae | *Cuscuta runyonii* | EF194683 |
| Angiosperm | Convolvulaceae | *Cuscuta salina* | EU330300 |
| Angiosperm | Convolvulaceae | *Cuscuta salina* var. *apoda* | EU330301 |
| Angiosperm | Convolvulaceae | *Cuscuta salina* var. *major* | EF194711 |
| Angiosperm | Convolvulaceae | *Cuscuta sandwichiana* | EU330309 |
| Angiosperm | Convolvulaceae | *Cuscuta sidarum* | EF194552 |
| Angiosperm | Convolvulaceae | *Cuscuta somaliensis* | DQ924625 |
| Angiosperm | Convolvulaceae | *Cuscuta somaliensis* | DQ924629 |
| Angiosperm | Convolvulaceae | *Cuscuta somaliensis* | DQ924634 |
| Angiosperm | Convolvulaceae | *Cuscuta somaliensis* | DQ924635 |
| Angiosperm | Convolvulaceae | *Cuscuta subinclusa* | EF194702 |
| Angiosperm | Convolvulaceae | *Cuscuta subinclusa* | EF194703 |
| Angiosperm | Convolvulaceae | *Cuscuta suksdorfii* | EU330302 |
| Angiosperm | Convolvulaceae | *Cuscuta tinctoria* | EF194617 |
| Angiosperm | Convolvulaceae | *Cuscuta tinctoria* | EU330306 |
| Angiosperm | Convolvulaceae | *Cuscuta triumvirati* | DQ924611 |
| Angiosperm | Convolvulaceae | *Cuscuta triumvirati* | DQ924612 |
| Angiosperm | Convolvulaceae | *Cuscuta umbellata* | EF192271 |
| Angiosperm | Convolvulaceae | *Cuscuta umbellata* | EU330314 |
| Angiosperm | Convolvulaceae | *Cuscuta umbellata* var. *reflexa* | EF194566 |
| Angiosperm | Convolvulaceae | *Cuscuta veatchii* | EU330296 |
| Angiosperm | Convolvulaceae | *Cuscuta victoriana* | EF194615 |
| Angiosperm | Convolvulaceae | *Cuscuta victoriana* | EF194616 |
| Angiosperm | Convolvulaceae | *Cuscuta werdermannii* | EF194655 |
| Angiosperm | Convolvulaceae | *Cuscuta yucatana* | EF194598 |
| Angiosperm | Convolvulaceae | *Dichondra carolinensis* | EU330328 |
| Angiosperm | Convolvulaceae | *Ipomoea coccinea x Ipomoea quamoclit* | EU330325 |
| Angiosperm | Convolvulaceae | *Ipomoea dichroa* | AF309158 |
| Angiosperm | Convolvulaceae | *Ipomoea lacunosa* | DQ005997 |
| Angiosperm | Convolvulaceae | *Ipomoea purpurea* | EU330324 |
| Angiosperm | Convolvulaceae | *Ipomoea rubrocaerulea* | DQ845161 |
| Angiosperm | Convolvulaceae | *Ipomoea violacea* | DQ845160 |
| Angiosperm | Cornaceae | *Cornus officinalis* | DQ683358 |
| Angiosperm | Cornaceae | *Helwingia chinensis* | AF200594 |
| Angiosperm | Crassulaceae | *Kalanchoe beauverdii* | AJ231305 |
| Angiosperm | Crassulaceae | *Kalanchoe beharensis* | AJ231307 |
| Angiosperm | Crassulaceae | *Kalanchoe beharensis* | AJ231308 |
| Angiosperm | Crassulaceae | *Kalanchoe beharensis* | AJ231314 |
| Angiosperm | Crassulaceae | *Kalanchoe beharensis* | AJ231315 |
| Angiosperm | Crassulaceae | *Kalanchoe beharensis* | AJ231316 |
| Angiosperm | Crassulaceae | *Kalanchoe beharensis* | AJ231317 |
| Angiosperm | Crassulaceae | *Kalanchoe beharensis* | AJ231318 |
| Angiosperm | Crassulaceae | *Kalanchoe beharensis* | AJ231319 |
| Angiosperm | Crassulaceae | *Kalanchoe beharensis* | AJ252913 |
| Angiosperm | Crassulaceae | *Kalanchoe bitteri* | AJ252898 |
| Angiosperm | Crassulaceae | *Kalanchoe blossfeldiana* | AJ231320 |
| Angiosperm | Crassulaceae | *Kalanchoe blossfeldiana* var. *coccinea* | AJ231326 |
| Angiosperm | Crassulaceae | *Kalanchoe bryophyllum* | AJ231328 |
| Angiosperm | Crassulaceae | *Kalanchoe campanulata* | AJ231309 |
| Angiosperm | Crassulaceae | *Kalanchoe daigremontiana* | AJ231327 |
| Angiosperm | Crassulaceae | *Kalanchoe delagoensis* | AJ231306 |
| Angiosperm | Crassulaceae | *Kalanchoe densiflora* | AJ231334 |
| Angiosperm | Crassulaceae | *Kalanchoe farinacea* | AJ231342 |
| Angiosperm | Crassulaceae | *Kalanchoe fedtschenkoi* | AJ231329 |
| Angiosperm | Crassulaceae | *Kalanchoe gastonis-bonnieri* | AJ231310 |
| Angiosperm | Crassulaceae | *Kalanchoe germanae* | AJ252889 |
| Angiosperm | Crassulaceae | *Kalanchoe geroldii* var. *geroldii* | AJ252890 |
| Angiosperm | Crassulaceae | *Kalanchoe geroldii* var. *viridifolia* | AJ252891 |
| Angiosperm | Crassulaceae | *Kalanchoe gracilipes* | AJ231312 |
| Angiosperm | Crassulaceae | *Kalanchoe grandiflora* | AJ252893 |
| Angiosperm | Crassulaceae | *Kalanchoe integrifolia* | AJ252902 |
| Angiosperm | Crassulaceae | *Kalanchoe integrifolia* | AJ252903 |
| Angiosperm | Crassulaceae | *Kalanchoe jongmansii* | AJ231311 |
| Angiosperm | Crassulaceae | *Kalanchoe kewensis* | AJ231333 |
| Angiosperm | Crassulaceae | *Kalanchoe linearifolia* | AJ231340 |
| Angiosperm | Crassulaceae | *Kalanchoe longiflora* | AJ231339 |
| Angiosperm | Crassulaceae | *Kalanchoe longifolia* | AJ231335 |
| Angiosperm | Crassulaceae | *Kalanchoe manginii* | AJ231344 |
| Angiosperm | Crassulaceae | *Kalanchoe marmorata* | AJ252911 |
| Angiosperm | Crassulaceae | *Kalanchoe marnieriana* | AJ231346 |
| Angiosperm | Crassulaceae | *Kalanchoe marnieriana* | AJ252888 |
| Angiosperm | Crassulaceae | *Kalanchoe millottii* | AJ231337 |
| Angiosperm | Crassulaceae | *Kalanchoe miniata* | AJ231324 |
| Angiosperm | Crassulaceae | *Kalanchoe mitejea* | AJ252895 |
| Angiosperm | Crassulaceae | *Kalanchoe mocambicana* | AJ252901 |
| Angiosperm | Crassulaceae | *Kalanchoe nyikae* | AJ252894 |
| Angiosperm | Crassulaceae | *Kalanchoe orygalis* | AJ231338 |
| Angiosperm | Crassulaceae | *Kalanchoe peltata* | AJ231330 |
| Angiosperm | Crassulaceae | *Kalanchoe petitiana* | AJ231325 |
| Angiosperm | Crassulaceae | *Kalanchoe pinnata* | AJ231323 |
| Angiosperm | Crassulaceae | *Kalanchoe porphyrocalyx* | AJ231331 |
| Angiosperm | Crassulaceae | *Kalanchoe pubescens* | AJ231347 |
| Angiosperm | Crassulaceae | *Kalanchoe pumila* | AJ231332 |
| Angiosperm | Crassulaceae | *Kalanchoe rechingeri* | AJ252892 |
| Angiosperm | Crassulaceae | *Kalanchoe rhombopilosa* | AJ231348 |
| Angiosperm | Crassulaceae | *Kalanchoe rhombopilosa* | AJ252896 |
| Angiosperm | Crassulaceae | *Kalanchoe rhombopilosa* var. *viridifolia* | AJ252897 |
| Angiosperm | Crassulaceae | *Kalanchoe rosei* | AJ252886 |
| Angiosperm | Crassulaceae | *Kalanchoe rosei* | AJ252887 |
| Angiosperm | Crassulaceae | *Kalanchoe scapigera* | AJ252912 |
| Angiosperm | Crassulaceae | *Kalanchoe streptantha* | AJ231322 |
| Angiosperm | Crassulaceae | *Kalanchoe thyrsiflora* | AJ231341 |
| Angiosperm | Crassulaceae | *Kalanchoe tomentosa* | AJ231349 |
| Angiosperm | Crassulaceae | *Kalanchoe tomentosa* | AJ252904 |
| Angiosperm | Crassulaceae | *Kalanchoe tomentosa* | AJ252905 |
| Angiosperm | Crassulaceae | *Kalanchoe uniflora* | AJ231343 |
| Angiosperm | Crassulaceae | *Kalanchoe waldheimii* | AJ252885 |
| Angiosperm | Crassulaceae | *Orostachys fimbriata* | AB088578 |
| Angiosperm | Crassulaceae | *Orostachys furusei* | AM039921 |
| Angiosperm | Crassulaceae | *Orostachys iwarenge* | AB088573 |
| Angiosperm | Crassulaceae | *Orostachys japonica* | AB088576 |
| Angiosperm | Crassulaceae | *Orostachys malacophylla* | AB088572 |
| Angiosperm | Crassulaceae | *Orostachys maximowiczii* | AM039923 |
| Angiosperm | Crassulaceae | *Orostachys spinosa* | AB088577 |
| Angiosperm | Crassulaceae | *Rhodiola algida* | AB088608 |
| Angiosperm | Crassulaceae | *Rhodiola algida* | EU239670 |
| Angiosperm | Crassulaceae | *Rhodiola angusta* | AM039927 |
| Angiosperm | Crassulaceae | *Rhodiola bupleuroides* | AB088592 |
| Angiosperm | Crassulaceae | *Rhodiola bupleuroides* | EU239671 |
| Angiosperm | Crassulaceae | *Rhodiola chrysanthemifolia* subsp. *chrysanthemifolia* | AB088604 |
| Angiosperm | Crassulaceae | *Rhodiola chrysanthemifolia* subsp. *sacra* | AB088603 |
| Angiosperm | Crassulaceae | *Rhodiola chrysanthemifolia* subsp. *sacra* | AB088606 |
| Angiosperm | Crassulaceae | *Rhodiola crenulata* | AY352898 |
| Angiosperm | Crassulaceae | *Rhodiola crenulata* | AY359888 |
| Angiosperm | Crassulaceae | *Rhodiola crenulata* | AY359890 |
| Angiosperm | Crassulaceae | *Rhodiola cretinii* | AB088588 |
| Angiosperm | Crassulaceae | *Rhodiola dumulosa* | AB088595 |
| Angiosperm | Crassulaceae | *Rhodiola fastigiata* | AY359898 |
| Angiosperm | Crassulaceae | *Rhodiola fastigiata* | AY359899 |
| Angiosperm | Crassulaceae | *Rhodiola fastigiata* | AY359906 |
| Angiosperm | Crassulaceae | *Rhodiola fastigiata* | EU239672 |
| Angiosperm | Crassulaceae | *Rhodiola heterodonta* | AB088596 |
| Angiosperm | Crassulaceae | *Rhodiola himalensis* | AB088593 |
| Angiosperm | Crassulaceae | *Rhodiola humilis* | AB088611 |
| Angiosperm | Crassulaceae | *Rhodiola ishidae* | AB088600 |
| Angiosperm | Crassulaceae | *Rhodiola kirilowii* | AB088601 |
| Angiosperm | Crassulaceae | *Rhodiola kirilowii* | EU239669 |
| Angiosperm | Crassulaceae | *Rhodiola macrocarpa* | AB088590 |
| Angiosperm | Crassulaceae | *Rhodiola nepalica* | AB088598 |
| Angiosperm | Crassulaceae | *Rhodiola nobilis* subsp. *atuntsuensis* | AB088589 |
| Angiosperm | Crassulaceae | *Rhodiola purpureoviridis* subsp. *phariensis* | AB088591 |
| Angiosperm | Crassulaceae | *Rhodiola rosea* | AB088599 |
| Angiosperm | Crassulaceae | *Rhodiola serrata* | AB088597 |
| Angiosperm | Crassulaceae | *Rhodiola sinuata* | AB088605 |
| Angiosperm | Crassulaceae | *Rhodiola wallichiana* | AB088607 |
| Angiosperm | Crassulaceae | *Rhodiola yunnanensis* | AB088602 |
| Angiosperm | Crassulaceae | *Rhodiola yunnanensis* | AY352896 |
| Angiosperm | Crassulaceae | *Rhodiola yunnanensis* | EU239673 |
| Angiosperm | Crassulaceae | *Sedum alexanderi* | EF632174 |
| Angiosperm | Crassulaceae | *Sedum allantoides* | AY545712 |
| Angiosperm | Crassulaceae | *Sedum bergeri* | AY352897 |
| Angiosperm | Crassulaceae | *Sedum bulbiferum* | AB088628 |
| Angiosperm | Crassulaceae | *Sedum clavatum* | AY545713 |
| Angiosperm | Crassulaceae | *Sedum corynephyllum* | AY545715 |
| Angiosperm | Crassulaceae | *Sedum hakonense* | AB088625 |
| Angiosperm | Crassulaceae | *Sedum jaccardianum* | AY082100 |
| Angiosperm | Crassulaceae | *Sedum japonicum* | AB088617 |
| Angiosperm | Crassulaceae | *Sedum lineare* | AB088623 |
| Angiosperm | Crassulaceae | *Sedum makinoi* | AB088627 |
| Angiosperm | Crassulaceae | *Sedum mexicanum* | AB088621 |
| Angiosperm | Crassulaceae | *Sedum modestum* | AY082101 |
| Angiosperm | Crassulaceae | *Sedum multicaule* | AB088631 |
| Angiosperm | Crassulaceae | *Sedum oaxacanum* | AY545716 |
| Angiosperm | Crassulaceae | *Sedum oaxacanum* | EF632176 |
| Angiosperm | Crassulaceae | *Sedum oreades* | AB088632 |
| Angiosperm | Crassulaceae | *Sedum oryzifolium* | AB088618 |
| Angiosperm | Crassulaceae | *Sedum palmeri* | AY545717 |
| Angiosperm | Crassulaceae | *Sedum sarmentosum* | AB088624 |
| Angiosperm | Crassulaceae | *Sedum subtile* | AB088622 |
| Angiosperm | Crassulaceae | *Sedum tosaense* | AB088620 |
| Angiosperm | Crassulaceae | *Sedum triactina* | AB088629 |
| Angiosperm | Crassulaceae | *Sedum trullipetalum* | AB088630 |
| Angiosperm | Crassulaceae | *Sedum yabeanum* | AB088626 |
| Angiosperm | Crassulaceae | *Sedum zentaro-tashiroi* | AB088619 |
| Angiosperm | Cruciferae | *Descurainia artemisioides* | DQ418708 |
| Angiosperm | Cruciferae | *Descurainia bourgeauana* | DQ418709 |
| Angiosperm | Cruciferae | *Descurainia californica* | AY230616 |
| Angiosperm | Cruciferae | *Descurainia californica* | AY230617 |
| Angiosperm | Cruciferae | *Descurainia depressa* | DQ418712 |
| Angiosperm | Cruciferae | *Descurainia gilva* | DQ249860 |
| Angiosperm | Cruciferae | *Descurainia gilva* | DQ418714 |
| Angiosperm | Cruciferae | *Descurainia incana* subsp. *incana* | AF205582 |
| Angiosperm | Cruciferae | *Descurainia incana* subsp. *incana* | AF205583 |
| Angiosperm | Cruciferae | *Descurainia incisa* | DQ418717 |
| Angiosperm | Cruciferae | *Descurainia incisa* subsp. *viscosa* | AF118859 |
| Angiosperm | Cruciferae | *Descurainia kochii* | DQ418718 |
| Angiosperm | Cruciferae | *Descurainia lemsii* | DQ418719 |
| Angiosperm | Cruciferae | *Descurainia millefolia* | DQ418721 |
| Angiosperm | Cruciferae | *Descurainia pinnata* | AY230620 |
| Angiosperm | Cruciferae | *Descurainia pinnata* | AY230621 |
| Angiosperm | Cruciferae | *Descurainia pinnata* | AY230622 |
| Angiosperm | Cruciferae | *Descurainia pinnata* subsp. *brachycarpa* | AF205584 |
| Angiosperm | Cruciferae | *Descurainia pinnata* subsp. *filipes* | AF205585 |
| Angiosperm | Cruciferae | *Descurainia pinnata* subsp. *nelsonii* | AF205586 |
| Angiosperm | Cruciferae | *Descurainia sophia* | AF118860 |
| Angiosperm | Cruciferae | *Descurainia sophia* | AF205587 |
| Angiosperm | Cruciferae | *Descurainia sophia* | AY230618 |
| Angiosperm | Cruciferae | *Descurainia sophia* | AY230619 |
| Angiosperm | Cruciferae | *Descurainia sophia* | DQ418727 |
| Angiosperm | Cruciferae | *Descurainia tanacetifolia* | DQ418728 |
| Angiosperm | Cruciferae | *Descurainia torulosa* | AF118861 |
| Angiosperm | Cruciferae | *Descurainia torulosa* | AF118862 |
| Angiosperm | Cruciferae | *Descurainia torulosa* | AF118863 |
| Angiosperm | Cruciferae | *Descurainia torulosa* | AF118864 |
| Angiosperm | Cruciferae | *Descurainia torulosa* | AF118865 |
| Angiosperm | Cruciferae | *Pegaeophyton scapiflorum* | DQ518398 |
| Angiosperm | Cruciferae | *Sinapis alba* | AF128106 |
| Angiosperm | Cucurbitaceae | *Benincasa hispida* | FJ980302 |
| Angiosperm | Cucurbitaceae | *Bolbostemma paniculatum* | FJ980305 |
| Angiosperm | Cucurbitaceae | *Citrullus lanatus* | AJ488232 |
| Angiosperm | Cucurbitaceae | *Citrullus lanatus* | EF595859 |
| Angiosperm | Cucurbitaceae | *Cucumis melo* | EU312157 |
| Angiosperm | Cucurbitaceae | *Cucumis melo* | EU312158 |
| Angiosperm | Cucurbitaceae | *Cucumis melo* | EU312159 |
| Angiosperm | Cucurbitaceae | *Momordica charantia* var. *abbreviata* | AY606265 |
| Angiosperm | Cucurbitaceae | *Momordica cochinchinensis* | AY606266 |
| Angiosperm | Cucurbitaceae | *Trichosanthes cucumerina* | AF013353 |
| Angiosperm | Cyperaceae | *Carex acutiformis* | AY278300 |
| Angiosperm | Cyperaceae | *Carex aff. raoulii* | AY699634 |
| Angiosperm | Cyperaceae | *Carex albula* | AY699614 |
| Angiosperm | Cyperaceae | *Carex appressa* | AY699637 |
| Angiosperm | Cyperaceae | *Carex austroalpina* | AY278276 |
| Angiosperm | Cyperaceae | *Carex berggrenii* | EU352219 |
| Angiosperm | Cyperaceae | *Carex bicolor* | AY278283 |
| Angiosperm | Cyperaceae | *Carex bigelowii* | AY278303 |
| Angiosperm | Cyperaceae | *Carex brachystachys* | AY278277 |
| Angiosperm | Cyperaceae | *Carex breviculmis* | AY699627 |
| Angiosperm | Cyperaceae | *Carex capillaris* | AY278256 |
| Angiosperm | Cyperaceae | *Carex carsei* | EU352220 |
| Angiosperm | Cyperaceae | *Carex colensoi* | EU352222 |
| Angiosperm | Cyperaceae | *Carex comans* | AY699629 |
| Angiosperm | Cyperaceae | *Carex diandra* | AY699626 |
| Angiosperm | Cyperaceae | *Carex dipsacea* | AY699622 |
| Angiosperm | Cyperaceae | *Carex echinata* | EU352224 |
| Angiosperm | Cyperaceae | *Carex enysii* | EU352225 |
| Angiosperm | Cyperaceae | *Carex extensa* | AY278311 |
| Angiosperm | Cyperaceae | *Carex fretalis* | EU352226 |
| Angiosperm | Cyperaceae | *Carex frigida* | AY278291 |
| Angiosperm | Cyperaceae | *Carex fuliginosa* | AY278254 |
| Angiosperm | Cyperaceae | *Carex geminata* | EU352227 |
| Angiosperm | Cyperaceae | *Carex hectorii* | AY699618 |
| Angiosperm | Cyperaceae | *Carex hispida* | AY278272 |
| Angiosperm | Cyperaceae | *Carex hostiana* | AY278309 |
| Angiosperm | Cyperaceae | *Carex humilis* | AY278260 |
| Angiosperm | Cyperaceae | *Carex inopinata* | EU352235 |
| Angiosperm | Cyperaceae | *Carex inversa* | AY699625 |
| Angiosperm | Cyperaceae | *Carex kaloides* | AY699638 |
| Angiosperm | Cyperaceae | *Carex kitaibeliana* | AY278258 |
| Angiosperm | Cyperaceae | *Carex lambertiana* | EU352236 |
| Angiosperm | Cyperaceae | *Carex lasiocarpa* | AY278297 |
| Angiosperm | Cyperaceae | *Carex limosa* | AY278298 |
| Angiosperm | Cyperaceae | *Carex litorosa* | AY699616 |
| Angiosperm | Cyperaceae | *Carex luzulina* | AY278252 |
| Angiosperm | Cyperaceae | *Carex magellanica* | AY278292 |
| Angiosperm | Cyperaceae | *Carex montana* | AY278271 |
| Angiosperm | Cyperaceae | *Carex muelleri* | AY699643 |
| Angiosperm | Cyperaceae | *Carex nigra* | AY278304 |
| Angiosperm | Cyperaceae | *Carex norvegica* | AY278264 |
| Angiosperm | Cyperaceae | *Carex ochrosaccus* | EU331114 |
| Angiosperm | Cyperaceae | *Carex ophiolithica* | EU352229 |
| Angiosperm | Cyperaceae | *Carex ornithopodioides* | AY278268 |
| Angiosperm | Cyperaceae | *Carex panicea* | AY278284 |
| Angiosperm | Cyperaceae | *Carex parviflora* | AY278265 |
| Angiosperm | Cyperaceae | *Carex pseudocyperus* | AY278295 |
| Angiosperm | Cyperaceae | *Carex pumila* | EU352230 |
| Angiosperm | Cyperaceae | *Carex rostrata* | AY278294 |
| Angiosperm | Cyperaceae | *Carex secta* | AY699611 |
| Angiosperm | Cyperaceae | *Carex secta* | AY699640 |
| Angiosperm | Cyperaceae | *Carex sectoides* | AY699639 |
| Angiosperm | Cyperaceae | *Carex serrulata* | AY278273 |
| Angiosperm | Cyperaceae | *Carex tenuiculmis* | AY699624 |
| Angiosperm | Cyperaceae | *Carex ternaria* | EU352233 |
| Angiosperm | Cyperaceae | *Carex testacea* | AY699621 |
| Angiosperm | Cyperaceae | *Carex trifida* | AY699620 |
| Angiosperm | Cyperaceae | *Carex uncifolia* | AY699612 |
| Angiosperm | Cyperaceae | *Carex ventosa* | AY699628 |
| Angiosperm | Cyperaceae | *Carex vesicaria* | AY278289 |
| Angiosperm | Cyperaceae | *Carex wakatipu* | EU352238 |
| Angiosperm | Cyperaceae | *Cyperus insularis* | DQ385559 |
| Angiosperm | Cyperaceae | *Cyperus ustulatus* | DQ385558 |
| Angiosperm | Dioscoreaceae | *Dioscorea collettii* var. *hypoglauca* | DQ267931 |
| Angiosperm | Dioscoreaceae | *Dioscorea nipponica* | DQ267930 |
| Angiosperm | Dipsacaceae | *Dipsacus japonicus* | AM296456 |
| Angiosperm | Dipsacaceae | *Dipsacus mitis* | AY236187 |
| Angiosperm | Dipsacaceae | *Dipsacus pilosus* | AY290016 |
| Angiosperm | Dipsacaceae | *Dipsacus strigosus* | AM296462 |
| Angiosperm | Dipsacaceae | *Pterocephalus hookeri* | AY236186 |
| Angiosperm | Ebenaceae | *Diospyros discolor* | AB175004 |
| Angiosperm | Ebenaceae | *Diospyros ehretioides* | AB175005 |
| Angiosperm | Ebenaceae | *Diospyros glandulosa* | AB175006 |
| Angiosperm | Ebenaceae | *Diospyros kuroiwai* | AB175010 |
| Angiosperm | Ebenaceae | *Diospyros lotus* | AB175012 |
| Angiosperm | Ebenaceae | *Diospyros lotus* | AB175013 |
| Angiosperm | Ebenaceae | *Diospyros mollis* | AB175015 |
| Angiosperm | Ebenaceae | *Diospyros oleifera* | AB175016 |
| Angiosperm | Ebenaceae | *Diospyros rhodocalyx* | AB175017 |
| Angiosperm | Ebenaceae | *Diospyros rhombifolia* | AB175018 |
| Angiosperm | Ebenaceae | *Diospyros rubra* | AB175019 |
| Angiosperm | Ebenaceae | *Diospyros sumatrana* | AB175020 |
| Angiosperm | Ebenaceae | *Diospyros virginiana* | AB175021 |
| Angiosperm | Elaeagnaceae | *Elaeagnus bockii* | AF440258 |
| Angiosperm | Elaeagnaceae | *Elaeagnus umbellata* | AF440257 |
| Angiosperm | Elaeagnaceae | *Hippophae gyantsensis* | AF440247 |
| Angiosperm | Elaeagnaceae | *Hippophae litangensis* | AF440251 |
| Angiosperm | Elaeagnaceae | *Hippophae neurocarpa* subsp. *stellatopilosa* | AF440254 |
| Angiosperm | Elaeagnaceae | *Hippophae rhamnoides* subsp. *carpatica* | AF440245 |
| Angiosperm | Elaeagnaceae | *Hippophae rhamnoides* subsp. *fluviatilis* | AF440248 |
| Angiosperm | Elaeagnaceae | *Hippophae rhamnoides* subsp. *mongolica* | AF440244 |
| Angiosperm | Elaeagnaceae | *Hippophae rhamnoides* subsp. *rhamnoides* | AF440242 |
| Angiosperm | Elaeagnaceae | *Hippophae rhamnoides* subsp. *sinensis* | AF440241 |
| Angiosperm | Elaeagnaceae | *Hippophae rhamnoides* subsp. *turkestanica* | AF440243 |
| Angiosperm | Elaeagnaceae | *Hippophae rhamnoides* subsp. *wolongensis* | AF440252 |
| Angiosperm | Elaeagnaceae | *Hippophae salicifolia* | AF440246 |
| Angiosperm | Elaeagnaceae | *Hippophae thibetana* | AF440249 |
| Angiosperm | Ericaceae | *Rhododendron arborescens* | AB300711 |
| Angiosperm | Ericaceae | *Rhododendron arborescens* | AF072477 |
| Angiosperm | Ericaceae | *Rhododendron atlanticum* | AF072479 |
| Angiosperm | Ericaceae | *Rhododendron cumberlandense* | AF072484 |
| Angiosperm | Ericaceae | *Rhododendron decorum* | DQ295782 |
| Angiosperm | Ericaceae | *Rhododendron delavayi* | DQ295783 |
| Angiosperm | Ericaceae | *Rhododendron formosanum* | AF297190 |
| Angiosperm | Ericaceae | *Rhododendron formosanum* | AF297191 |
| Angiosperm | Ericaceae | *Rhododendron hybrid* | AJ626914 |
| Angiosperm | Ericaceae | *Rhododendron hybrid* | AJ626915 |
| Angiosperm | Ericaceae | *Rhododendron hyperythrum* | AF297193 |
| Angiosperm | Ericaceae | *Rhododendron luteum* | AF072485 |
| Angiosperm | Ericaceae | *Rhododendron mariesii* | AF297202 |
| Angiosperm | Ericaceae | *Rhododendron maximum* | AJ626908 |
| Angiosperm | Ericaceae | *Rhododendron molle* | AF072486 |
| Angiosperm | Ericaceae | *Rhododendron nakaharae* | AB300709 |
| Angiosperm | Ericaceae | *Rhododendron nakaharae x Rhododendron indicum* | AB300708 |
| Angiosperm | Ericaceae | *Rhododendron occidentale* | AF072487 |
| Angiosperm | Ericaceae | *Rhododendron oldhamii* | AF297201 |
| Angiosperm | Ericaceae | *Rhododendron prunifolium* | AF072490 |
| Angiosperm | Ericaceae | *Rhododendron pseudochrysanthum* | AF297196 |
| Angiosperm | Ericaceae | *Rhododendron pseudochrysanthum* | AF297197 |
| Angiosperm | Ericaceae | *Rhododendron vaseyi* | AF072491 |
| Angiosperm | Ericaceae | *Rhododendron viscosum* | AB300710 |
| Angiosperm | Ericaceae | *Rhododendron viscosum* | AF072492 |
| Angiosperm | Ericaceae | *Rhododendron williamsianum* | AJ626909 |
| Angiosperm | Ericaceae | *Rhododendron williamsianum* | AJ626910 |
| Angiosperm | Eucommiaceae | *Eucommia ulmoides* | AY649996 |
| Angiosperm | Eucommiaceae | *Eucommia ulmoides* | AY649997 |
| Angiosperm | Eucommiaceae | *Eucommia ulmoides* | AY649998 |
| Angiosperm | Eucommiaceae | *Eucommia ulmoides* | AY649999 |
| Angiosperm | Eucommiaceae | *Eucommia ulmoides* | AY650000 |
| Angiosperm | Eucommiaceae | *Eucommia ulmoides* | AY650001 |
| Angiosperm | Eucommiaceae | *Eucommia ulmoides* | AY650002 |
| Angiosperm | Eucommiaceae | *Eucommia ulmoides* | AY650003 |
| Angiosperm | Eucommiaceae | *Eucommia ulmoides* | AY650004 |
| Angiosperm | Eucommiaceae | *Eucommia ulmoides* | AY650005 |
| Angiosperm | Eucommiaceae | *Eucommia ulmoides* | AY650006 |
| Angiosperm | Euphorbiaceae | *Breynia cernua* | AY936650 |
| Angiosperm | Euphorbiaceae | *Breynia disticha* | AY936651 |
| Angiosperm | Euphorbiaceae | *Breynia retusa* | AY936652 |
| Angiosperm | Euphorbiaceae | *Breynia vitis-idaea* | AY936653 |
| Angiosperm | Euphorbiaceae | *Croton abutiloides* | EU586903 |
| Angiosperm | Euphorbiaceae | *Croton aequatoris* | EU586904 |
| Angiosperm | Euphorbiaceae | *Croton astroites* | EU586901 |
| Angiosperm | Euphorbiaceae | *Croton ater* | EU586952 |
| Angiosperm | Euphorbiaceae | *Croton beetlei* | EU586916 |
| Angiosperm | Euphorbiaceae | *Croton cajucara* | EU586913 |
| Angiosperm | Euphorbiaceae | *Croton chilensis* | EU586905 |
| Angiosperm | Euphorbiaceae | *Croton chocoanus* | EU586941 |
| Angiosperm | Euphorbiaceae | *Croton cupreatus* | EU586919 |
| Angiosperm | Euphorbiaceae | *Croton curiosus* | EU586906 |
| Angiosperm | Euphorbiaceae | *Croton echinocarpus* | EU586922 |
| Angiosperm | Euphorbiaceae | *Croton echioides* | EU586907 |
| Angiosperm | Euphorbiaceae | *Croton eichleri* | EU586949 |
| Angiosperm | Euphorbiaceae | *Croton emporiorum* | EU586908 |
| Angiosperm | Euphorbiaceae | *Croton erythroxyloides* | EU586938 |
| Angiosperm | Euphorbiaceae | *Croton floccosus* | EU586923 |
| Angiosperm | Euphorbiaceae | *Croton gossypiifolius* | EU586924 |
| Angiosperm | Euphorbiaceae | *Croton gracilipes* | EU586909 |
| Angiosperm | Euphorbiaceae | *Croton helicoideus* | EU586902 |
| Angiosperm | Euphorbiaceae | *Croton hibiscifolius* | EU586925 |
| Angiosperm | Euphorbiaceae | *Croton laceratoglandulosus* | DQ836744 |
| Angiosperm | Euphorbiaceae | *Croton lagoensis* | EU586926 |
| Angiosperm | Euphorbiaceae | *Croton lechleri* | EU586927 |
| Angiosperm | Euphorbiaceae | *Croton macrobothrys* | EU586928 |
| Angiosperm | Euphorbiaceae | *Croton magdalenensis* | EU586929 |
| Angiosperm | Euphorbiaceae | *Croton medusae* | EU586933 |
| Angiosperm | Euphorbiaceae | *Croton megalodendron* | EU586942 |
| Angiosperm | Euphorbiaceae | *Croton megistocarpus* | EU586953 |
| Angiosperm | Euphorbiaceae | *Croton mongue* | EU586947 |
| Angiosperm | Euphorbiaceae | *Croton organensis* | EU586914 |
| Angiosperm | Euphorbiaceae | *Croton palanostigma* | EU586943 |
| Angiosperm | Euphorbiaceae | *Croton pallidulus* | EU586939 |
| Angiosperm | Euphorbiaceae | *Croton paludosus* | DQ787388 |
| Angiosperm | Euphorbiaceae | *Croton perspeciosus* | EU586931 |
| Angiosperm | Euphorbiaceae | *Croton piluliferus* | EU586932 |
| Angiosperm | Euphorbiaceae | *Croton priscus* | EU586950 |
| Angiosperm | Euphorbiaceae | *Croton purdiei* | EU586934 |
| Angiosperm | Euphorbiaceae | *Croton rimbachii* | EU586936 |
| Angiosperm | Euphorbiaceae | *Croton ruizianus* | EU586910 |
| Angiosperm | Euphorbiaceae | *Croton saltensis* | EU586911 |
| Angiosperm | Euphorbiaceae | *Croton sarcopetalus* | EU586912 |
| Angiosperm | Euphorbiaceae | *Croton troncosoi* | EU586940 |
| Angiosperm | Euphorbiaceae | *Croton warmingii* | EU586915 |
| Angiosperm | Euphorbiaceae | *Croton yavitensis* | EU586918 |
| Angiosperm | Euphorbiaceae | *Acalypha rhomboidea* | DQ006003 |
| Angiosperm | Euphorbiaceae | *Euphorbia spinosa* | EU650608 |
| Angiosperm | Euphorbiaceae | *Euphorbia spinosa* | EU650613 |
| Angiosperm | Euphorbiaceae | *Euphorbia spinosa* | EU650615 |
| Angiosperm | Euphorbiaceae | *Euphorbia spinosa* | EU650618 |
| Angiosperm | Euphorbiaceae | *Euphorbia spinosa* | EU650619 |
| Angiosperm | Euphorbiaceae | *Euphorbia spinosa* | EU650620 |
| Angiosperm | Euphorbiaceae | *Euphorbia spinosa* | EU650621 |
| Angiosperm | Euphorbiaceae | *Euphorbia spinosa* | EU650622 |
| Angiosperm | Euphorbiaceae | *Euphorbia spinosa* | EU650623 |
| Angiosperm | Euphorbiaceae | *Euphorbia spinosa* | EU650624 |
| Angiosperm | Euphorbiaceae | *Euphorbia spinosa* | EU650625 |
| Angiosperm | Euphorbiaceae | *Flueggea leucopyrus* | AY936654 |
| Angiosperm | Euphorbiaceae | *Flueggea tinctoria* | AY936655 |
| Angiosperm | Euphorbiaceae | *Flueggea virosa* | AY936656 |
| Angiosperm | Euphorbiaceae | *Glochidion eucleoides* | AY936657 |
| Angiosperm | Euphorbiaceae | *Glochidion puberum* | AY936659 |
| Angiosperm | Euphorbiaceae | *Jatropha curcas* | AM774639 |
| Angiosperm | Euphorbiaceae | *Jatropha curcas* | EU340800 |
| Angiosperm | Euphorbiaceae | *Leptopus australis* | AM745811 |
| Angiosperm | Euphorbiaceae | *Leptopus australis* | AM745813 |
| Angiosperm | Euphorbiaceae | *Leptopus calcareus* | AM745815 |
| Angiosperm | Euphorbiaceae | *Leptopus calcareus* | AM745817 |
| Angiosperm | Euphorbiaceae | *Leptopus chinensis* | AM745819 |
| Angiosperm | Euphorbiaceae | *Leptopus chinensis* | AM745821 |
| Angiosperm | Euphorbiaceae | *Leptopus esquirolii* | AM745834 |
| Angiosperm | Euphorbiaceae | *Leptopus phyllanthoides* | AM745836 |
| Angiosperm | Euphorbiaceae | *Mallotus barbatus* | DQ866591 |
| Angiosperm | Euphorbiaceae | *Mallotus brachythyrsus* | DQ866592 |
| Angiosperm | Euphorbiaceae | *Mallotus caudatus* | DQ866593 |
| Angiosperm | Euphorbiaceae | *Mallotus claoxyloides* | DQ866594 |
| Angiosperm | Euphorbiaceae | *Mallotus discolor* | DQ866597 |
| Angiosperm | Euphorbiaceae | *Mallotus ficifolius* | DQ866599 |
| Angiosperm | Euphorbiaceae | *Mallotus griffithianus* | DQ866600 |
| Angiosperm | Euphorbiaceae | *Mallotus khasianus* | DQ866601 |
| Angiosperm | Euphorbiaceae | *Mallotus lackeyi* | DQ866602 |
| Angiosperm | Euphorbiaceae | *Mallotus leucocalyx* | DQ866603 |
| Angiosperm | Euphorbiaceae | *Mallotus macrostachyus* | DQ866604 |
| Angiosperm | Euphorbiaceae | *Mallotus miquelianus* | DQ866605 |
| Angiosperm | Euphorbiaceae | *Mallotus oppositifolius* | DQ866606 |
| Angiosperm | Euphorbiaceae | *Mallotus pallidus* | DQ866607 |
| Angiosperm | Euphorbiaceae | *Mallotus paniculatus* | DQ866608 |
| Angiosperm | Euphorbiaceae | *Mallotus paniculatus* | DQ866609 |
| Angiosperm | Euphorbiaceae | *Mallotus penangensis* | DQ866611 |
| Angiosperm | Euphorbiaceae | *Mallotus pierrei* | DQ866615 |
| Angiosperm | Euphorbiaceae | *Mallotus polyadenos* | DQ866616 |
| Angiosperm | Euphorbiaceae | *Mallotus repandus* | DQ813305 |
| Angiosperm | Euphorbiaceae | *Mallotus repandus* | DQ866617 |
| Angiosperm | Euphorbiaceae | *Mallotus resinosus* | DQ866618 |
| Angiosperm | Euphorbiaceae | *Mallotus rhamnifolius* | DQ866619 |
| Angiosperm | Euphorbiaceae | *Mallotus rufidulus* | DQ866620 |
| Angiosperm | Euphorbiaceae | *Mallotus spinulosus* | DQ866532 |
| Angiosperm | Euphorbiaceae | *Mallotus subpeltatus* | DQ866621 |
| Angiosperm | Euphorbiaceae | *Mallotus subulatus* | DQ866622 |
| Angiosperm | Euphorbiaceae | *Mallotus tetracoccus* | DQ866623 |
| Angiosperm | Euphorbiaceae | *Mallotus thorelii* | DQ866624 |
| Angiosperm | Euphorbiaceae | *Phyllanthus acuminatus* | AY936667 |
| Angiosperm | Euphorbiaceae | *Phyllanthus amarus* | AY725467 |
| Angiosperm | Euphorbiaceae | *Phyllanthus amarus* | AY936669 |
| Angiosperm | Euphorbiaceae | *Phyllanthus amarus* | EU429328 |
| Angiosperm | Euphorbiaceae | *Phyllanthus amarus* | EU876849 |
| Angiosperm | Euphorbiaceae | *Phyllanthus andalangiensis* | AY936670 |
| Angiosperm | Euphorbiaceae | *Phyllanthus angustifolius* | AY936671 |
| Angiosperm | Euphorbiaceae | *Phyllanthus betsileanus* | AY936672 |
| Angiosperm | Euphorbiaceae | *Phyllanthus betsileanus* | AY936673 |
| Angiosperm | Euphorbiaceae | *Phyllanthus calycinus* | AY936674 |
| Angiosperm | Euphorbiaceae | *Phyllanthus caroliniensis* | AY936675 |
| Angiosperm | Euphorbiaceae | *Phyllanthus casticum* | AY936676 |
| Angiosperm | Euphorbiaceae | *Phyllanthus chacoensis* | AY936677 |
| Angiosperm | Euphorbiaceae | *Phyllanthus chamaecerasus* | AY936678 |
| Angiosperm | Euphorbiaceae | *Phyllanthus chrysanthus* | AY936680 |
| Angiosperm | Euphorbiaceae | *Phyllanthus chryseus* | AY936681 |
| Angiosperm | Euphorbiaceae | *Phyllanthus cinereus* | AY936682 |
| Angiosperm | Euphorbiaceae | *Phyllanthus claussenii* | AY936683 |
| Angiosperm | Euphorbiaceae | *Phyllanthus cochinchinensis* | AY936684 |
| Angiosperm | Euphorbiaceae | *Phyllanthus comosus* | AY936685 |
| Angiosperm | Euphorbiaceae | *Phyllanthus debilis* | AY725465 |
| Angiosperm | Euphorbiaceae | *Phyllanthus debilis* | AY936686 |
| Angiosperm | Euphorbiaceae | *Phyllanthus discolor* | AY936688 |
| Angiosperm | Euphorbiaceae | *Phyllanthus embergeri* | AY725463 |
| Angiosperm | Euphorbiaceae | *Phyllanthus emblica* | AY830087 |
| Angiosperm | Euphorbiaceae | *Phyllanthus emblica* | AY936689 |
| Angiosperm | Euphorbiaceae | *Phyllanthus emblica* | EU626400 |
| Angiosperm | Euphorbiaceae | *Phyllanthus emblica* | EU876843 |
| Angiosperm | Euphorbiaceae | *Phyllanthus fraternus* | EU429330 |
| Angiosperm | Euphorbiaceae | *Phyllanthus fraternus* | EU876847 |
| Angiosperm | Euphorbiaceae | *Phyllanthus gardnerianus* | AY936694 |
| Angiosperm | Euphorbiaceae | *Phyllanthus graveolens* | AY936696 |
| Angiosperm | Euphorbiaceae | *Phyllanthus hookeri* | AY831634 |
| Angiosperm | Euphorbiaceae | *Phyllanthus hutchinsonianus* | AY936697 |
| Angiosperm | Euphorbiaceae | *Phyllanthus juglandifolius* | AY936698 |
| Angiosperm | Euphorbiaceae | *Phyllanthus juglandifolius* | AY936699 |
| Angiosperm | Euphorbiaceae | *Phyllanthus kaessneri* | AY936700 |
| Angiosperm | Euphorbiaceae | *Phyllanthus kanalensis* | AY936701 |
| Angiosperm | Euphorbiaceae | *Phyllanthus klotzschianus* | AY936702 |
| Angiosperm | Euphorbiaceae | *Phyllanthus lokohensis* | AY936703 |
| Angiosperm | Euphorbiaceae | *Phyllanthus lokohensis* | AY936704 |
| Angiosperm | Euphorbiaceae | *Phyllanthus loranthoides* | AY936705 |
| Angiosperm | Euphorbiaceae | *Phyllanthus madagascariensis* | AY936706 |
| Angiosperm | Euphorbiaceae | *Phyllanthus maderaspatensis* | AY936707 |
| Angiosperm | Euphorbiaceae | *Phyllanthus mannianus* | AY936708 |
| Angiosperm | Euphorbiaceae | *Phyllanthus microdictyus* | AY936709 |
| Angiosperm | Euphorbiaceae | *Phyllanthus muellerianus* | AY936711 |
| Angiosperm | Euphorbiaceae | *Phyllanthus myrtifolius* | AY725466 |
| Angiosperm | Euphorbiaceae | *Phyllanthus myrtifolius* | AY936712 |
| Angiosperm | Euphorbiaceae | *Phyllanthus niruri* | AY936713 |
| Angiosperm | Euphorbiaceae | *Phyllanthus orbicularis* | AY936718 |
| Angiosperm | Euphorbiaceae | *Phyllanthus oxyphyllus* | AY936719 |
| Angiosperm | Euphorbiaceae | *Phyllanthus pachystylus* | AY936720 |
| Angiosperm | Euphorbiaceae | *Phyllanthus pancherianus* | AY936721 |
| Angiosperm | Euphorbiaceae | *Phyllanthus pentandrus* | AY936722 |
| Angiosperm | Euphorbiaceae | *Phyllanthus pervilleanus* | AY936723 |
| Angiosperm | Euphorbiaceae | *Phyllanthus pinnatus* | AY936724 |
| Angiosperm | Euphorbiaceae | *Phyllanthus polyphyllus* | AY936725 |
| Angiosperm | Euphorbiaceae | *Phyllanthus pulcher* | AY936726 |
| Angiosperm | Euphorbiaceae | *Phyllanthus purpusii* | AY936727 |
| Angiosperm | Euphorbiaceae | *Phyllanthus reticulatus* | AY835843 |
| Angiosperm | Euphorbiaceae | *Phyllanthus reticulatus* | AY936728 |
| Angiosperm | Euphorbiaceae | *Phyllanthus reticulatus* | EU309043 |
| Angiosperm | Euphorbiaceae | *Phyllanthus rheedii* | AY936729 |
| Angiosperm | Euphorbiaceae | *Phyllanthus salviifolius* | AY936730 |
| Angiosperm | Euphorbiaceae | *Phyllanthus sellowianus* | AY936731 |
| Angiosperm | Euphorbiaceae | *Phyllanthus sepialis* | AY936732 |
| Angiosperm | Euphorbiaceae | *Phyllanthus unifoliatus* | AY936734 |
| Angiosperm | Euphorbiaceae | *Phyllanthus urinaria* | AY936735 |
| Angiosperm | Euphorbiaceae | *Phyllanthus urinaria* | AY936736 |
| Angiosperm | Euphorbiaceae | *Phyllanthus urinaria* | EU876851 |
| Angiosperm | Euphorbiaceae | *Phyllanthus urinaria* subsp. *nudicarpus* | AY830088 |
| Angiosperm | Euphorbiaceae | *Phyllanthus urinaria* subsp. *urinaria* | AY830089 |
| Angiosperm | Euphorbiaceae | *Phyllanthus ussuriensis* | AY842254 |
| Angiosperm | Euphorbiaceae | *Phyllanthus vakinankaratrae* | AY936737 |
| Angiosperm | Euphorbiaceae | *Phyllanthus virgatus* | AY831635 |
| Angiosperm | Euphorbiaceae | *Phyllanthus virgatus* | AY936738 |
| Angiosperm | Euphorbiaceae | *Phyllanthus welwitschianus* | AY936739 |
| Angiosperm | Euphorbiaceae | *Phyllanthus wheeleri* | AY936740 |
| Angiosperm | Euphorbiaceae | *Ricinus communis* | AY918198 |
| Angiosperm | Euphorbiaceae | *Sauropus androgynus* | AY936744 |
| Angiosperm | Euphorbiaceae | *Sauropus elachophyllus* | AY936745 |
| Angiosperm | Euphorbiaceae | *Sauropus orbicularis* | AY936746 |
| Angiosperm | Euphorbiaceae | *Sauropus retroversus* | AY936747 |
| Angiosperm | Euphorbiaceae | *Sauropus spatulifolius* | AY936748 |
| Angiosperm | Eupteleaceae | *Euptelea pleiosperma* | AF162214 |
| Angiosperm | Fabaceae | *Abrus precatorius* | AF467015 |
| Angiosperm | Fabaceae | *Acacia alexandri* | DQ029264 |
| Angiosperm | Fabaceae | *Acacia anthochaera* | DQ029243 |
| Angiosperm | Fabaceae | *Acacia aphanoclada* | DQ029256 |
| Angiosperm | Fabaceae | *Acacia chartacea* | DQ029241 |
| Angiosperm | Fabaceae | *Acacia chartacea* | DQ029263 |
| Angiosperm | Fabaceae | *Acacia dempsteri* | DQ029259 |
| Angiosperm | Fabaceae | *Acacia glaucocaesia* | DQ029265 |
| Angiosperm | Fabaceae | *Acacia inaequilatera* | DQ029242 |
| Angiosperm | Fabaceae | *Acacia longispinea* | DQ029246 |
| Angiosperm | Fabaceae | *Acacia marramamba* | DQ029257 |
| Angiosperm | Fabaceae | *Acacia mearnsii* | AY864897 |
| Angiosperm | Fabaceae | *Acacia murrayana* | DQ029248 |
| Angiosperm | Fabaceae | *Acacia pachyacra* | DQ029249 |
| Angiosperm | Fabaceae | *Acacia platycarpa* | DQ029244 |
| Angiosperm | Fabaceae | *Acacia platycarpa* | DQ029245 |
| Angiosperm | Fabaceae | *Acacia praelongata* | DQ029250 |
| Angiosperm | Fabaceae | *Acacia ryaniana* | DQ029262 |
| Angiosperm | Fabaceae | *Acacia strongylophylla* | DQ029258 |
| Angiosperm | Fabaceae | *Acacia synchronicia* | DQ029266 |
| Angiosperm | Fabaceae | *Acacia synchronicia* | DQ029267 |
| Angiosperm | Fabaceae | *Acacia synchronicia* | DQ029268 |
| Angiosperm | Fabaceae | *Acacia victoriae* | DQ029276 |
| Angiosperm | Fabaceae | *Acacia victoriae* | DQ029277 |
| Angiosperm | Fabaceae | *Acacia victoriae* | DQ029278 |
| Angiosperm | Fabaceae | *Acacia victoriae* | DQ029279 |
| Angiosperm | Fabaceae | *Acacia victoriae* | DQ029280 |
| Angiosperm | Fabaceae | *Acacia victoriae* | DQ029281 |
| Angiosperm | Fabaceae | *Albizia adinocephala* | EF638170 |
| Angiosperm | Fabaceae | *Albizia julibrissin* | DQ499102 |
| Angiosperm | Fabaceae | *Albizia procera* | DQ499103 |
| Angiosperm | Fabaceae | *Albizia saponaria* | EF638171 |
| Angiosperm | Fabaceae | *Almaleea cambagei* | AF113758 |
| Angiosperm | Fabaceae | *Amorpha apiculata* | AY426771 |
| Angiosperm | Fabaceae | *Amorpha fruticosa* | AY426774 |
| Angiosperm | Fabaceae | *Amphicarpaea bracteata* | AF417008 |
| Angiosperm | Fabaceae | *Amphicarpaea bracteata* | AF417010 |
| Angiosperm | Fabaceae | *Amphicarpaea bracteata* | AF417011 |
| Angiosperm | Fabaceae | *Amphicarpaea bracteata* | AF417015 |
| Angiosperm | Fabaceae | *Amphicarpaea bracteata* | AF417016 |
| Angiosperm | Fabaceae | *Amphicarpaea bracteata* | AF417019 |
| Angiosperm | Fabaceae | *Amphicarpaea edgeworthii* | AF417012 |
| Angiosperm | Fabaceae | *Amphicarpaea edgeworthii* | AF417013 |
| Angiosperm | Fabaceae | *Anthonotha gabunensis* | AF513652 |
| Angiosperm | Fabaceae | *Anthonotha macrophylla* | AF513653 |
| Angiosperm | Fabaceae | *Arachis correntina* | AY862309 |
| Angiosperm | Fabaceae | *Arachis ipaensis* | AY862311 |
| Angiosperm | Fabaceae | *Arachis monticola* | AY862312 |
| Angiosperm | Fabaceae | *Astragalus balfourianus* | AF521951 |
| Angiosperm | Fabaceae | *Astragalus complanatus* | AF521950 |
| Angiosperm | Fabaceae | *Astragalus yatungensis* | AF521955 |
| Angiosperm | Fabaceae | *Baikiaea insignis* | AY955811 |
| Angiosperm | Fabaceae | *Bauhinia cheilantha* | DQ787410 |
| Angiosperm | Fabaceae | *Berlinia confusa* | AF513669 |
| Angiosperm | Fabaceae | *Bikinia breynei* | AF513672 |
| Angiosperm | Fabaceae | *Bikinia congensis* | AF513673 |
| Angiosperm | Fabaceae | *Bikinia congensis* | AF513674 |
| Angiosperm | Fabaceae | *Bikinia coriacea* | AF513675 |
| Angiosperm | Fabaceae | *Bikinia durandii* | AF513676 |
| Angiosperm | Fabaceae | *Bikinia evrardii* | AF513677 |
| Angiosperm | Fabaceae | *Bikinia grisea* | AF513678 |
| Angiosperm | Fabaceae | *Bikinia letestui* subsp. *letestui* | AF513679 |
| Angiosperm | Fabaceae | *Bikinia letestui* subsp. *mayumbensis* | AF513680 |
| Angiosperm | Fabaceae | *Bikinia media* | AF513681 |
| Angiosperm | Fabaceae | *Bikinia media* | AF513682 |
| Angiosperm | Fabaceae | *Bikinia pellegrinii* | AF513684 |
| Angiosperm | Fabaceae | *Bikinia pellegrinii* | AF513685 |
| Angiosperm | Fabaceae | *Bossiaea buxifolia* | AY883354 |
| Angiosperm | Fabaceae | *Bossiaea lenticularis* | AF518104 |
| Angiosperm | Fabaceae | *Bossiaea linophylla* | AF287657 |
| Angiosperm | Fabaceae | *Brachystegia leonensis* | AF513686 |
| Angiosperm | Fabaceae | *Brachystegia longifolia* | AF513687 |
| Angiosperm | Fabaceae | *Caesalpinia sappan* | EU243573 |
| Angiosperm | Fabaceae | *Canavalia boliviana* | AY293838 |
| Angiosperm | Fabaceae | *Canavalia bonariensis* | AY293839 |
| Angiosperm | Fabaceae | *Canavalia ensiformis* | EU288912 |
| Angiosperm | Fabaceae | *Canavalia ensiformis* | EU288913 |
| Angiosperm | Fabaceae | *Canavalia gladiata* | AY293837 |
| Angiosperm | Fabaceae | *Canavalia gladiata* | EU288929 |
| Angiosperm | Fabaceae | *Canavalia grandiflora* | AY293840 |
| Angiosperm | Fabaceae | *Caragana acanthophylla* | AB262523 |
| Angiosperm | Fabaceae | *Caragana acanthophylla* | DQ914778 |
| Angiosperm | Fabaceae | *Caragana arborescens* | DQ311963 |
| Angiosperm | Fabaceae | *Caragana bicolor* | DQ914790 |
| Angiosperm | Fabaceae | *Caragana bongardiana* | DQ914777 |
| Angiosperm | Fabaceae | *Caragana dasyphylla* | DQ914786 |
| Angiosperm | Fabaceae | *Caragana erinacea* | AB262532 |
| Angiosperm | Fabaceae | *Caragana franchetiana* | EU243572 |
| Angiosperm | Fabaceae | *Caragana hololeuca* | AB262524 |
| Angiosperm | Fabaceae | *Caragana hololeuca* | DQ914779 |
| Angiosperm | Fabaceae | *Caragana jubata* | EU243571 |
| Angiosperm | Fabaceae | *Caragana korshinskii* | AY626914 |
| Angiosperm | Fabaceae | *Caragana korshinskii* var. *intermedia* | AB262534 |
| Angiosperm | Fabaceae | *Caragana leucophloea* | AB262535 |
| Angiosperm | Fabaceae | *Caragana microphylla* | DQ311964 |
| Angiosperm | Fabaceae | *Caragana opulens* | AB262531 |
| Angiosperm | Fabaceae | *Caragana ordosica* | DQ914780 |
| Angiosperm | Fabaceae | *Caragana pruinosa* | AB262533 |
| Angiosperm | Fabaceae | *Caragana purdomii* | DQ914784 |
| Angiosperm | Fabaceae | *Caragana roborovskyi* | AF521958 |
| Angiosperm | Fabaceae | *Caragana roborovskyi* | DQ914781 |
| Angiosperm | Fabaceae | *Caragana rosea* | AB262526 |
| Angiosperm | Fabaceae | *Caragana rosea* | DQ914775 |
| Angiosperm | Fabaceae | *Caragana sibirica* | AY626912 |
| Angiosperm | Fabaceae | *Caragana sinica* | DQ914785 |
| Angiosperm | Fabaceae | *Caragana tibetica* | AB262527 |
| Angiosperm | Fabaceae | *Caragana tibetica* | EU243570 |
| Angiosperm | Fabaceae | *Caragana tragacanthoides* | AB262536 |
| Angiosperm | Fabaceae | *Cassia javanica* subsp. *nodosa* | FJ980413 |
| Angiosperm | Fabaceae | *Chamaecrista fasciculata* | EF590760 |
| Angiosperm | Fabaceae | *Collaea cipoensis* | EU499369 |
| Angiosperm | Fabaceae | *Cologania broussonetii* | AY583501 |
| Angiosperm | Fabaceae | *Copaifera officinalis* | AY955816 |
| Angiosperm | Fabaceae | *Copaifera salikounda* | AY955815 |
| Angiosperm | Fabaceae | *Crotalaria arenaria* | AJ313485 |
| Angiosperm | Fabaceae | *Crotalaria atrorubens* | AJ313486 |
| Angiosperm | Fabaceae | *Crotalaria barkae* | AJ313487 |
| Angiosperm | Fabaceae | *Crotalaria calycina* | AJ313488 |
| Angiosperm | Fabaceae | *Crotalaria capensis* | AF287674 |
| Angiosperm | Fabaceae | *Crotalaria cylindrocarpa* | AJ313490 |
| Angiosperm | Fabaceae | *Crotalaria goreensis* | AJ313492 |
| Angiosperm | Fabaceae | *Crotalaria lanceolata* | AJ313495 |
| Angiosperm | Fabaceae | *Crotalaria lathyroides* | AJ313496 |
| Angiosperm | Fabaceae | *Crotalaria ochroleuca* | AJ313497 |
| Angiosperm | Fabaceae | *Crotalaria pallida* | AJ313499 |
| Angiosperm | Fabaceae | *Crotalaria perrottetii* | AJ313498 |
| Angiosperm | Fabaceae | *Crotalaria retusa* | AJ313501 |
| Angiosperm | Fabaceae | *Crotalaria senegalensis* | AJ313502 |
| Angiosperm | Fabaceae | *Cryptosepalum tetraphyllum* | AF513689 |
| Angiosperm | Fabaceae | *Cullen corylifolium* | FJ606754 |
| Angiosperm | Fabaceae | *Dalbergia miscolobium* | DQ787405 |
| Angiosperm | Fabaceae | *Desmodium floridanum* | EF517898 |
| Angiosperm | Fabaceae | *Dolichos trilobus* | AY583525 |
| Angiosperm | Fabaceae | *Euchilopsis linearis* | AF113761 |
| Angiosperm | Fabaceae | *Eurypetalum tessmannii* | AY955804 |
| Angiosperm | Fabaceae | *Eurypetalum unijugum* | AY955803 |
| Angiosperm | Fabaceae | *Eutaxia microphylla* | AF113762 |
| Angiosperm | Fabaceae | *Flemingia glutinosa* | FJ980289 |
| Angiosperm | Fabaceae | *Flemingia macrophylla* | FJ980288 |
| Angiosperm | Fabaceae | *Galactia lindenii* | EU499371 |
| Angiosperm | Fabaceae | *Galega officinalis* | DQ311965 |
| Angiosperm | Fabaceae | *Galega orientalis* | DQ311966 |
| Angiosperm | Fabaceae | *Gleditsia amorphoides* | AF510001 |
| Angiosperm | Fabaceae | *Gleditsia amorphoides* | AF510002 |
| Angiosperm | Fabaceae | *Gleditsia aquatica* | AF509993 |
| Angiosperm | Fabaceae | *Gleditsia aquatica* | AF509994 |
| Angiosperm | Fabaceae | *Gleditsia aquatica* | AF509995 |
| Angiosperm | Fabaceae | *Gleditsia aquatica* | AF509996 |
| Angiosperm | Fabaceae | *Gleditsia aquatica* | AF509997 |
| Angiosperm | Fabaceae | *Gleditsia aquatica* | AF509998 |
| Angiosperm | Fabaceae | *Gleditsia aquatica* | AF509999 |
| Angiosperm | Fabaceae | *Gleditsia aquatica* | AF510000 |
| Angiosperm | Fabaceae | *Gleditsia australis* | AF510023 |
| Angiosperm | Fabaceae | *Gleditsia australis* | AF510024 |
| Angiosperm | Fabaceae | *Gleditsia caspica* | AF510004 |
| Angiosperm | Fabaceae | *Gleditsia caspica* | AF510007 |
| Angiosperm | Fabaceae | *Gleditsia delavayi* | AF510009 |
| Angiosperm | Fabaceae | *Gleditsia fera* | AF510025 |
| Angiosperm | Fabaceae | *Gleditsia fera* | AF510026 |
| Angiosperm | Fabaceae | *Gleditsia japonica* | AF510015 |
| Angiosperm | Fabaceae | *Gleditsia japonica* | AF510016 |
| Angiosperm | Fabaceae | *Gleditsia microphylla* | AF510027 |
| Angiosperm | Fabaceae | *Gleditsia microphylla* | AF510028 |
| Angiosperm | Fabaceae | *Gleditsia microphylla* | AF510029 |
| Angiosperm | Fabaceae | *Gleditsia rolfei* | AF510017 |
| Angiosperm | Fabaceae | *Gleditsia rolfei* | AF510018 |
| Angiosperm | Fabaceae | *Gleditsia triacanthos* | AF509969 |
| Angiosperm | Fabaceae | *Gleditsia triacanthos* | AF509970 |
| Angiosperm | Fabaceae | *Gleditsia triacanthos* | AF509971 |
| Angiosperm | Fabaceae | *Gleditsia triacanthos* | AF509973 |
| Angiosperm | Fabaceae | *Gleditsia triacanthos* | AF509975 |
| Angiosperm | Fabaceae | *Gleditsia triacanthos* | AF509976 |
| Angiosperm | Fabaceae | *Gleditsia triacanthos* | AF509977 |
| Angiosperm | Fabaceae | *Gleditsia triacanthos* | AF509978 |
| Angiosperm | Fabaceae | *Gleditsia triacanthos* | AF509979 |
| Angiosperm | Fabaceae | *Gleditsia triacanthos* | AF509980 |
| Angiosperm | Fabaceae | *Gleditsia triacanthos* | AF509981 |
| Angiosperm | Fabaceae | *Glycine albicans* | U60541 |
| Angiosperm | Fabaceae | *Glycine arenaria* | U60543 |
| Angiosperm | Fabaceae | *Glycine argyrea* | U60535 |
| Angiosperm | Fabaceae | *Glycine canescens* | AF023444 |
| Angiosperm | Fabaceae | *Glycine canescens* | U60533 |
| Angiosperm | Fabaceae | *Glycine clandestina* | AY168326 |
| Angiosperm | Fabaceae | *Glycine clandestina* | U60534 |
| Angiosperm | Fabaceae | *Glycine curvata* | U60547 |
| Angiosperm | Fabaceae | *Glycine cyrtoloba* | U60548 |
| Angiosperm | Fabaceae | *Glycine dolichocarpa* | AJ011339 |
| Angiosperm | Fabaceae | *Glycine dolichocarpa* | AJ011340 |
| Angiosperm | Fabaceae | *Glycine dolichocarpa* | AJ011341 |
| Angiosperm | Fabaceae | *Glycine falcata* | U60549 |
| Angiosperm | Fabaceae | *Glycine hirticaulis* | AY433931 |
| Angiosperm | Fabaceae | *Glycine hirticaulis* | AY433932 |
| Angiosperm | Fabaceae | *Glycine hirticaulis* | U60545 |
| Angiosperm | Fabaceae | *Glycine lactovirens* | U60540 |
| Angiosperm | Fabaceae | *Glycine latifolia* | U60538 |
| Angiosperm | Fabaceae | *Glycine latrobeana* | U60536 |
| Angiosperm | Fabaceae | *Glycine max* | AJ011337 |
| Angiosperm | Fabaceae | *Glycine max* | EU288921 |
| Angiosperm | Fabaceae | *Glycine microphylla* | U60537 |
| Angiosperm | Fabaceae | *Glycine soja* | U60550 |
| Angiosperm | Fabaceae | *Glycine tabacina* | AJ011346 |
| Angiosperm | Fabaceae | *Glycine tabacina* | AJ011347 |
| Angiosperm | Fabaceae | *Glycine tabacina* | U60539 |
| Angiosperm | Fabaceae | *Glycine tomentella* | AY433906 |
| Angiosperm | Fabaceae | *Glycine tomentella* | AY433908 |
| Angiosperm | Fabaceae | *Glycine tomentella* | AY433909 |
| Angiosperm | Fabaceae | *Glycine tomentella* | AY433910 |
| Angiosperm | Fabaceae | *Glycine tomentella* | AY433911 |
| Angiosperm | Fabaceae | *Glycine tomentella* | AY433912 |
| Angiosperm | Fabaceae | *Glycine tomentella* | AY433913 |
| Angiosperm | Fabaceae | *Glycine tomentella* | AY433914 |
| Angiosperm | Fabaceae | *Glycine tomentella* | AY433920 |
| Angiosperm | Fabaceae | *Glycine tomentella* | AY433925 |
| Angiosperm | Fabaceae | *Glycine tomentella* | U60542 |
| Angiosperm | Fabaceae | *Glycine tomentella* | U60544 |
| Angiosperm | Fabaceae | *Glycyrrhiza glabra* | AY065623 |
| Angiosperm | Fabaceae | *Glycyrrhiza uralensis* | AY065622 |
| Angiosperm | Fabaceae | *Goodia lotifolia* | AF287655 |
| Angiosperm | Fabaceae | *Goodia medicaginea* | AF518103 |
| Angiosperm | Fabaceae | *Guibourtia ehie* | AY955801 |
| Angiosperm | Fabaceae | *Guibourtia hymenaeifolia* | AY955802 |
| Angiosperm | Fabaceae | *Guibourtia hymenaeifolia* | DQ787421 |
| Angiosperm | Fabaceae | *Hedysarum carnosum* | AY772224 |
| Angiosperm | Fabaceae | *Hedysarum membranaceum* | AY772228 |
| Angiosperm | Fabaceae | *Hedysarum pallidum* | AY772229 |
| Angiosperm | Fabaceae | *Hedysarum spinosissimum* subsp. *capitatum* | AY772223 |
| Angiosperm | Fabaceae | *Hedysarum wrightianum* | AB329695 |
| Angiosperm | Fabaceae | *Hovea elliptica* | AF287640 |
| Angiosperm | Fabaceae | *Hovea trisperma* | AF287639 |
| Angiosperm | Fabaceae | *Hymenaea courbaril* | AY955800 |
| Angiosperm | Fabaceae | *Indigofera ammoxylum* | AF274699 |
| Angiosperm | Fabaceae | *Indigofera amoena* | AF521789 |
| Angiosperm | Fabaceae | *Indigofera angustata* | AF521756 |
| Angiosperm | Fabaceae | *Indigofera angustifolia* | AF521752 |
| Angiosperm | Fabaceae | *Indigofera argentea* | AF521785 |
| Angiosperm | Fabaceae | *Indigofera bemarahaensis* | AF521744 |
| Angiosperm | Fabaceae | *Indigofera braamtonyi* | AF521729 |
| Angiosperm | Fabaceae | *Indigofera candolleana* | AF287641 |
| Angiosperm | Fabaceae | *Indigofera colutea* | AF521776 |
| Angiosperm | Fabaceae | *Indigofera conzattii* | AF521585 |
| Angiosperm | Fabaceae | *Indigofera cordifolia* | AF521741 |
| Angiosperm | Fabaceae | *Indigofera cuneifolia* | AF521749 |
| Angiosperm | Fabaceae | *Indigofera cytisoides* | AF521754 |
| Angiosperm | Fabaceae | *Indigofera dalzellii* | AF521793 |
| Angiosperm | Fabaceae | *Indigofera decora* | AF521732 |
| Angiosperm | Fabaceae | *Indigofera decora* | AF534797 |
| Angiosperm | Fabaceae | *Indigofera denudata* | AF521753 |
| Angiosperm | Fabaceae | *Indigofera dimidiata* | AF521792 |
| Angiosperm | Fabaceae | *Indigofera disticha* | AF274693 |
| Angiosperm | Fabaceae | *Indigofera filicaulis* | AF521759 |
| Angiosperm | Fabaceae | *Indigofera filifolia* | AF521760 |
| Angiosperm | Fabaceae | *Indigofera frondosa* | AF521722 |
| Angiosperm | Fabaceae | *Indigofera gleichenioides* | AF521723 |
| Angiosperm | Fabaceae | *Indigofera grandiflora* | AF521734 |
| Angiosperm | Fabaceae | *Indigofera grandiflora* | AF534795 |
| Angiosperm | Fabaceae | *Indigofera grata* | AF521790 |
| Angiosperm | Fabaceae | *Indigofera heterophylla* | AF521751 |
| Angiosperm | Fabaceae | *Indigofera hilaris* | AF274694 |
| Angiosperm | Fabaceae | *Indigofera hispida* | AF521755 |
| Angiosperm | Fabaceae | *Indigofera jucunda* | AF521728 |
| Angiosperm | Fabaceae | *Indigofera kirilowii* | AF534796 |
| Angiosperm | Fabaceae | *Indigofera kirkii* | AF521718 |
| Angiosperm | Fabaceae | *Indigofera koreana* | AF534794 |
| Angiosperm | Fabaceae | *Indigofera leptocarpa* | AF521742 |
| Angiosperm | Fabaceae | *Indigofera lespedezioides* | AF521780 |
| Angiosperm | Fabaceae | *Indigofera leucoclada* | AF521743 |
| Angiosperm | Fabaceae | *Indigofera longimucronata* | AF274695 |
| Angiosperm | Fabaceae | *Indigofera marmorata* | AF521779 |
| Angiosperm | Fabaceae | *Indigofera mauritanica* | AF521794 |
| Angiosperm | Fabaceae | *Indigofera mildbraediana* | AF521762 |
| Angiosperm | Fabaceae | *Indigofera nebrowniana* | AF521791 |
| Angiosperm | Fabaceae | *Indigofera paniculata* | AF521720 |
| Angiosperm | Fabaceae | *Indigofera porrecta* | AF521750 |
| Angiosperm | Fabaceae | *Indigofera pseudotinctoria* | AF521747 |
| Angiosperm | Fabaceae | *Indigofera pseudotinctoria* | AF534784 |
| Angiosperm | Fabaceae | *Indigofera rhynchocarpa* | AF521787 |
| Angiosperm | Fabaceae | *Indigofera rubroglandulosa* | AF521761 |
| Angiosperm | Fabaceae | *Indigofera sanguinea* | AF521721 |
| Angiosperm | Fabaceae | *Indigofera schimperi* | AF274696 |
| Angiosperm | Fabaceae | *Indigofera spinosa* | AF521784 |
| Angiosperm | Fabaceae | *Indigofera strobilifera* | AF521719 |
| Angiosperm | Fabaceae | *Indigofera swaziensis* | AF521788 |
| Angiosperm | Fabaceae | *Indigofera tephrosioides* | AF521781 |
| Angiosperm | Fabaceae | *Indigofera thibaudiana* | AF521586 |
| Angiosperm | Fabaceae | *Indigofera torulosa* | AF521774 |
| Angiosperm | Fabaceae | *Indigofera trifoliata* | AF521746 |
| Angiosperm | Fabaceae | *Indigofera tristis* | AF521724 |
| Angiosperm | Fabaceae | *Indigofera trita* | AF521745 |
| Angiosperm | Fabaceae | *Indigofera truxillensis* | AF521777 |
| Angiosperm | Fabaceae | *Indigofera venulosa* | AF521733 |
| Angiosperm | Fabaceae | *Indigofera venulosa* | AF534798 |
| Angiosperm | Fabaceae | *Indigofera vohemarensis* | AF274697 |
| Angiosperm | Fabaceae | *Indigofera zeyheri* | AF274698 |
| Angiosperm | Fabaceae | *Isoberlinia doka* | AF513691 |
| Angiosperm | Fabaceae | *Lathyrus annuus* | AY839344 |
| Angiosperm | Fabaceae | *Lathyrus davidii* | AY839350 |
| Angiosperm | Fabaceae | *Lathyrus gmelinii* | AY839357 |
| Angiosperm | Fabaceae | *Lathyrus holochlorus* | AY839359 |
| Angiosperm | Fabaceae | *Lathyrus jepsonii* | AY839362 |
| Angiosperm | Fabaceae | *Lathyrus lanszwertii* | AY839366 |
| Angiosperm | Fabaceae | *Lathyrus linifolius* | AY839368 |
| Angiosperm | Fabaceae | *Lathyrus littoralis* | AY839369 |
| Angiosperm | Fabaceae | *Lathyrus multiceps* var. *setiger* | AY839370 |
| Angiosperm | Fabaceae | *Lathyrus neurolobus* | AY839373 |
| Angiosperm | Fabaceae | *Lathyrus polyphyllus* | AY839383 |
| Angiosperm | Fabaceae | *Lathyrus rigidus* | AY839387 |
| Angiosperm | Fabaceae | *Lathyrus sativus* | DQ311968 |
| Angiosperm | Fabaceae | *Lathyrus setifolius* | AY839391 |
| Angiosperm | Fabaceae | *Lathyrus sphaericus* | AY839393 |
| Angiosperm | Fabaceae | *Lathyrus subandinus* | AY839396 |
| Angiosperm | Fabaceae | *Lathyrus transsylvanicus* | AY839400 |
| Angiosperm | Fabaceae | *Lathyrus vernus* | AY839404 |
| Angiosperm | Fabaceae | *Lathyrus vestitus* | AY839405 |
| Angiosperm | Fabaceae | *Lotus angustissimus* | DQ311970 |
| Angiosperm | Fabaceae | *Lotus corniculatus* | DQ312207 |
| Angiosperm | Fabaceae | *Lotus edulis* | DQ311972 |
| Angiosperm | Fabaceae | *Lotus glaucus* | DQ311973 |
| Angiosperm | Fabaceae | *Lotus japonicus* | DQ311975 |
| Angiosperm | Fabaceae | *Lotus parviflorus* | DQ311976 |
| Angiosperm | Fabaceae | *Lotus pedunculatus* | DQ311977 |
| Angiosperm | Fabaceae | *Lotus suaveolens* | DQ311978 |
| Angiosperm | Fabaceae | *Lotus tenuis* | DQ311979 |
| Angiosperm | Fabaceae | *Lotus unifoliolatus* | DQ311969 |
| Angiosperm | Fabaceae | *Medicago edgeworthii* | AY256393 |
| Angiosperm | Fabaceae | *Medicago lanigera* | AY256394 |
| Angiosperm | Fabaceae | *Medicago lupulina* | DQ311980 |
| Angiosperm | Fabaceae | *Medicago medicaginoides* | AY256395 |
| Angiosperm | Fabaceae | *Medicago monantha* | AY256396 |
| Angiosperm | Fabaceae | *Medicago monspeliaca* | AY256397 |
| Angiosperm | Fabaceae | *Medicago platycarpa* | AY256398 |
| Angiosperm | Fabaceae | *Medicago polyceratia* | AY256399 |
| Angiosperm | Fabaceae | *Medicago polymorpha* | DQ311981 |
| Angiosperm | Fabaceae | *Medicago popovii* | AY256400 |
| Angiosperm | Fabaceae | *Medicago radiata* | AY256401 |
| Angiosperm | Fabaceae | *Medicago sativa* | AY256392 |
| Angiosperm | Fabaceae | *Medicago truncatula* | AF074399 |
| Angiosperm | Fabaceae | *Medicago truncatula* | AF233339 |
| Angiosperm | Fabaceae | *Melilotus albus* | DQ311984 |
| Angiosperm | Fabaceae | *Melilotus officinalis* | DQ311985 |
| Angiosperm | Fabaceae | *Millettia grandis* | AY009139 |
| Angiosperm | Fabaceae | *Oddoniodendron normandii* | AF513698 |
| Angiosperm | Fabaceae | *Ononis mitissima* | DQ311986 |
| Angiosperm | Fabaceae | *Oxytropis aucherii* | AB051908 |
| Angiosperm | Fabaceae | *Oxytropis besseyi* var. *ventosa* | AF121756 |
| Angiosperm | Fabaceae | *Oxytropis borealis* var. *viscida* | AF121758 |
| Angiosperm | Fabaceae | *Oxytropis multiceps* | AF121760 |
| Angiosperm | Fabaceae | *Oxytropis pilosa* | AF121759 |
| Angiosperm | Fabaceae | *Oxytropis sericea* | AF121757 |
| Angiosperm | Fabaceae | *Oxytropis splendens* | AF121761 |
| Angiosperm | Fabaceae | *Oxytropis szovitsii* | AB051909 |
| Angiosperm | Fabaceae | *Oxytropis szovitsii* | AF121754 |
| Angiosperm | Fabaceae | *Parochetus communis* | DQ311987 |
| Angiosperm | Fabaceae | *Phaseolus acutifolius* | AF069126 |
| Angiosperm | Fabaceae | *Phaseolus acutifolius* | AF115140 |
| Angiosperm | Fabaceae | *Phaseolus acutifolius* | AF115143 |
| Angiosperm | Fabaceae | *Phaseolus acutifolius* | AF115145 |
| Angiosperm | Fabaceae | *Phaseolus acutifolius* | AF115146 |
| Angiosperm | Fabaceae | *Phaseolus albinervus* | AF115183 |
| Angiosperm | Fabaceae | *Phaseolus altimontanus* | DQ445743 |
| Angiosperm | Fabaceae | *Phaseolus amblysepalus* | AF115217 |
| Angiosperm | Fabaceae | *Phaseolus amblysepalus* | AF115218 |
| Angiosperm | Fabaceae | *Phaseolus angustissimus* | AF115208 |
| Angiosperm | Fabaceae | *Phaseolus augusti* | AF115179 |
| Angiosperm | Fabaceae | *Phaseolus augusti* | AF115180 |
| Angiosperm | Fabaceae | *Phaseolus campanulatus* | AF115232 |
| Angiosperm | Fabaceae | *Phaseolus carterae* | DQ445744 |
| Angiosperm | Fabaceae | *Phaseolus chiapasanus* | AF115222 |
| Angiosperm | Fabaceae | *Phaseolus coccineus* | AF115154 |
| Angiosperm | Fabaceae | *Phaseolus coccineus* | AF115156 |
| Angiosperm | Fabaceae | *Phaseolus coccineus* | EU288920 |
| Angiosperm | Fabaceae | *Phaseolus costaricensis* | AF115147 |
| Angiosperm | Fabaceae | *Phaseolus dasycarpus* | DQ445746 |
| Angiosperm | Fabaceae | *Phaseolus dumosus* | AF115149 |
| Angiosperm | Fabaceae | *Phaseolus esquincensis* | AF115225 |
| Angiosperm | Fabaceae | *Phaseolus filiformis* | AF115206 |
| Angiosperm | Fabaceae | *Phaseolus filiformis* | AF115207 |
| Angiosperm | Fabaceae | *Phaseolus glabellus* | AF115244 |
| Angiosperm | Fabaceae | *Phaseolus gladiolatus* | AF115230 |
| Angiosperm | Fabaceae | *Phaseolus grayanus* | AF115240 |
| Angiosperm | Fabaceae | *Phaseolus grayanus* | AF115241 |
| Angiosperm | Fabaceae | *Phaseolus grayanus* | DQ445749 |
| Angiosperm | Fabaceae | *Phaseolus grayanus* | DQ445750 |
| Angiosperm | Fabaceae | *Phaseolus hintonii* | AF115226 |
| Angiosperm | Fabaceae | *Phaseolus jaliscanus* | AF115190 |
| Angiosperm | Fabaceae | *Phaseolus juquilensis* | AF115192 |
| Angiosperm | Fabaceae | *Phaseolus leptostachyus* | AF115201 |
| Angiosperm | Fabaceae | *Phaseolus leptostachyus* | AF115202 |
| Angiosperm | Fabaceae | *Phaseolus leptostachyus* | AF115203 |
| Angiosperm | Fabaceae | *Phaseolus leptostachyus* | AF115204 |
| Angiosperm | Fabaceae | *Phaseolus lignosus* | AF115177 |
| Angiosperm | Fabaceae | *Phaseolus lunatus* | AF069129 |
| Angiosperm | Fabaceae | *Phaseolus lunatus* | AF115171 |
| Angiosperm | Fabaceae | *Phaseolus lunatus* | AF115172 |
| Angiosperm | Fabaceae | *Phaseolus lunatus* | AF115175 |
| Angiosperm | Fabaceae | *Phaseolus lunatus* | AF115176 |
| Angiosperm | Fabaceae | *Phaseolus macrolepis* | DQ445752 |
| Angiosperm | Fabaceae | *Phaseolus maculatifolius* | AF115195 |
| Angiosperm | Fabaceae | *Phaseolus maculatifolius* | DQ445754 |
| Angiosperm | Fabaceae | *Phaseolus maculatifolius* | DQ445755 |
| Angiosperm | Fabaceae | *Phaseolus maculatus* | AF115189 |
| Angiosperm | Fabaceae | *Phaseolus maculatus* subsp. *ritensis* | AF115184 |
| Angiosperm | Fabaceae | *Phaseolus maculatus* subsp. *ritensis* | AF115185 |
| Angiosperm | Fabaceae | *Phaseolus maculatus* subsp. *ritensis* | AF115186 |
| Angiosperm | Fabaceae | *Phaseolus marechalii* | AF115197 |
| Angiosperm | Fabaceae | *Phaseolus mollis* | AF115170 |
| Angiosperm | Fabaceae | *Phaseolus nodosus* | DQ445757 |
| Angiosperm | Fabaceae | *Phaseolus oaxacanus* | DQ445760 |
| Angiosperm | Fabaceae | *Phaseolus oligospermus* | AF115233 |
| Angiosperm | Fabaceae | *Phaseolus oligospermus* | AF115234 |
| Angiosperm | Fabaceae | *Phaseolus pachyrrhizoides* | AF115178 |
| Angiosperm | Fabaceae | *Phaseolus parvifolius* | AF115141 |
| Angiosperm | Fabaceae | *Phaseolus parvifolius* | DQ445761 |
| Angiosperm | Fabaceae | *Phaseolus parvulus* | AF115211 |
| Angiosperm | Fabaceae | *Phaseolus pauciflorus* | AF115216 |
| Angiosperm | Fabaceae | *Phaseolus pauciflorus* | DQ445762 |
| Angiosperm | Fabaceae | *Phaseolus pedicellatus* | AF115237 |
| Angiosperm | Fabaceae | *Phaseolus pedicellatus* | DQ445763 |
| Angiosperm | Fabaceae | *Phaseolus pedicellatus* | DQ445764 |
| Angiosperm | Fabaceae | *Phaseolus pedicellatus* | DQ445765 |
| Angiosperm | Fabaceae | *Phaseolus perplexus* | AF115220 |
| Angiosperm | Fabaceae | *Phaseolus perplexus* | AF115221 |
| Angiosperm | Fabaceae | *Phaseolus plagiocylix* | AF115215 |
| Angiosperm | Fabaceae | *Phaseolus pluriflorus* | AF115213 |
| Angiosperm | Fabaceae | *Phaseolus pluriflorus* | AF115214 |
| Angiosperm | Fabaceae | *Phaseolus polymorphus* | DQ445769 |
| Angiosperm | Fabaceae | *Phaseolus polystachios* subsp. *sinuatus* | AF115194 |
| Angiosperm | Fabaceae | *Phaseolus polystachios* subsp. *smilacifolius* | AF115193 |
| Angiosperm | Fabaceae | *Phaseolus reticulatus* | DQ445770 |
| Angiosperm | Fabaceae | *Phaseolus rotundatus* | DQ445771 |
| Angiosperm | Fabaceae | *Phaseolus sonorensis* | DQ445773 |
| Angiosperm | Fabaceae | *Phaseolus talamancensis* | AF115246 |
| Angiosperm | Fabaceae | *Phaseolus tenellus* | AF115219 |
| Angiosperm | Fabaceae | *Phaseolus tuerckheimii* | DQ445774 |
| Angiosperm | Fabaceae | *Phaseolus vulgaris* | AF074398 |
| Angiosperm | Fabaceae | *Phaseolus vulgaris* | AF115161 |
| Angiosperm | Fabaceae | *Phaseolus vulgaris* | AF115162 |
| Angiosperm | Fabaceae | *Phaseolus vulgaris* | AF115167 |
| Angiosperm | Fabaceae | *Phaseolus vulgaris* | AF115168 |
| Angiosperm | Fabaceae | *Phaseolus vulgaris* | AF115169 |
| Angiosperm | Fabaceae | *Phaseolus vulgaris* | EU288905 |
| Angiosperm | Fabaceae | *Phaseolus vulgaris* | EU288906 |
| Angiosperm | Fabaceae | *Phaseolus xanthotrichus* | AF115224 |
| Angiosperm | Fabaceae | *Phaseolus xolocotzii* | AF115199 |
| Angiosperm | Fabaceae | *Phaseolus xolocotzii* | AF115247 |
| Angiosperm | Fabaceae | *Phaseolus zimapanensis* | AF115229 |
| Angiosperm | Fabaceae | *Platylobium formosum* | AF287651 |
| Angiosperm | Fabaceae | *Pueraria montana* | DQ472517 |
| Angiosperm | Fabaceae | *Pueraria montana* | DQ472518 |
| Angiosperm | Fabaceae | *Pueraria montana* var. *lobata* | DQ302145 |
| Angiosperm | Fabaceae | *Pueraria montana* var. *lobata* | DQ472511 |
| Angiosperm | Fabaceae | *Pueraria montana* var. *lobata* | DQ472512 |
| Angiosperm | Fabaceae | *Pueraria montana* var. *lobata* | DQ472513 |
| Angiosperm | Fabaceae | *Pueraria montana* var. *thomsonii* | DQ302146 |
| Angiosperm | Fabaceae | *Pueraria montana* var. *thomsonii* | DQ302147 |
| Angiosperm | Fabaceae | *Pueraria montana* var. *thomsonii* | DQ302148 |
| Angiosperm | Fabaceae | *Pueraria montana* var. *thomsonii* | DQ302149 |
| Angiosperm | Fabaceae | *Pultenaea adunca* | AY883362 |
| Angiosperm | Fabaceae | *Pultenaea arida* | AF518120 |
| Angiosperm | Fabaceae | *Pultenaea barbata* | AY883363 |
| Angiosperm | Fabaceae | *Pultenaea costata* | AY883365 |
| Angiosperm | Fabaceae | *Pultenaea daphnoides* | AF113768 |
| Angiosperm | Fabaceae | *Pultenaea dentata* | AF113769 |
| Angiosperm | Fabaceae | *Pultenaea largiflorens* | AY883370 |
| Angiosperm | Fabaceae | *Pultenaea myrtoides* | AY883372 |
| Angiosperm | Fabaceae | *Pultenaea neurocalyx* | AF113771 |
| Angiosperm | Fabaceae | *Pultenaea pedunculata* | AY883374 |
| Angiosperm | Fabaceae | *Pultenaea purpurea* | AY883376 |
| Angiosperm | Fabaceae | *Pultenaea reticulata* | AF113772 |
| Angiosperm | Fabaceae | *Pultenaea stipularis* | AY883378 |
| Angiosperm | Fabaceae | *Pultenaea strobilifera* | AY883409 |
| Angiosperm | Fabaceae | *Pultenaea strobilifera* | AY883410 |
| Angiosperm | Fabaceae | *Pultenaea subalpina* | AY883379 |
| Angiosperm | Fabaceae | *Pultenaea vestita* | AY883381 |
| Angiosperm | Fabaceae | *Pultenaea viscidula* | AY883382 |
| Angiosperm | Fabaceae | *Pultenaea williamsoniana* | AY883383 |
| Angiosperm | Fabaceae | *Rafnia alata* | AJ744938 |
| Angiosperm | Fabaceae | *Rafnia amplexicaulis* | AJ744943 |
| Angiosperm | Fabaceae | *Rafnia angulata* subsp. *thunbergii* | AJ744935 |
| Angiosperm | Fabaceae | *Rafnia capensis* subsp. *dichotoma* | AJ744949 |
| Angiosperm | Fabaceae | *Rafnia capensis* subsp. *ovata* | AJ744948 |
| Angiosperm | Fabaceae | *Rafnia crassifolia* | AJ744939 |
| Angiosperm | Fabaceae | *Rafnia diffusa* | AJ744944 |
| Angiosperm | Fabaceae | *Rafnia ovata* | AF287680 |
| Angiosperm | Fabaceae | *Rafnia ovata* | AJ744941 |
| Angiosperm | Fabaceae | *Rafnia racemosa* subsp. *racemosa* | AJ744933 |
| Angiosperm | Fabaceae | *Rafnia rostrata* subsp. *rostrata* | AJ744936 |
| Angiosperm | Fabaceae | *Rafnia schlechteriana* | AJ744950 |
| Angiosperm | Fabaceae | *Rafnia spicata* | AJ744945 |
| Angiosperm | Fabaceae | *Rafnia vlokii* | AJ744937 |
| Angiosperm | Fabaceae | *Robinia pseudoacacia* | EF494737 |
| Angiosperm | Fabaceae | *Senna alata* | FJ980412 |
| Angiosperm | Fabaceae | *Senna tora* | FJ572046 |
| Angiosperm | Fabaceae | *Sindoropsis le-testui* | AY955818 |
| Angiosperm | Fabaceae | *Sophora tomentosa* | AY725482 |
| Angiosperm | Fabaceae | *Sphaerolobium medium* | AF287660 |
| Angiosperm | Fabaceae | *Sphaerolobium minus* | AF518101 |
| Angiosperm | Fabaceae | *Sphaerolobium nudiflorum* | AF518102 |
| Angiosperm | Fabaceae | *Templetonia retusa* | AF287636 |
| Angiosperm | Fabaceae | *Templetonia sulcata* | AF287635 |
| Angiosperm | Fabaceae | *Tessmannia anomala* | AY955813 |
| Angiosperm | Fabaceae | *Tessmannia lescrauwaetii* | AY955812 |
| Angiosperm | Fabaceae | *Trigonella foenum-graecum* | DQ312196 |
| Angiosperm | Fabaceae | *Vaughania cerighellii* | AF521740 |
| Angiosperm | Fabaceae | *Vaughania depauperata* | AF521737 |
| Angiosperm | Fabaceae | *Vaughania humbertiana* | AF274700 |
| Angiosperm | Fabaceae | *Vaughania mahafalensis* | AF521736 |
| Angiosperm | Fabaceae | *Vaughania perrieri* | AF534800 |
| Angiosperm | Fabaceae | *Vaughania pseudocompressa* | AF274701 |
| Angiosperm | Fabaceae | *Vicia cracca* | DQ312197 |
| Angiosperm | Fabaceae | *Vicia montbretii* | EU202644 |
| Angiosperm | Fabaceae | *Vicia sativa* | DQ312198 |
| Angiosperm | Fabaceae | *Vicia villosa* | DQ312199 |
| Angiosperm | Fabaceae | *Vigna aconitifolia* | AF069118 |
| Angiosperm | Fabaceae | *Vigna adenantha* | AF069119 |
| Angiosperm | Fabaceae | *Vigna adenantha* | AY583526 |
| Angiosperm | Fabaceae | *Vigna angularis* | EU288895 |
| Angiosperm | Fabaceae | *Vigna angularis* | EU288896 |
| Angiosperm | Fabaceae | *Vigna angularis* | EU288897 |
| Angiosperm | Fabaceae | *Vigna angularis* | EU288898 |
| Angiosperm | Fabaceae | *Vigna angularis* | EU288899 |
| Angiosperm | Fabaceae | *Vigna angularis* | EU288900 |
| Angiosperm | Fabaceae | *Vigna angularis* | EU288901 |
| Angiosperm | Fabaceae | *Vigna angularis* | EU727147 |
| Angiosperm | Fabaceae | *Vigna caracalla* | AF069124 |
| Angiosperm | Fabaceae | *Vigna lasiocarpa* | AY583522 |
| Angiosperm | Fabaceae | *Vigna linearis* | AF069123 |
| Angiosperm | Fabaceae | *Vigna longifolia* | AY583520 |
| Angiosperm | Fabaceae | *Vigna longifolia* | DQ445739 |
| Angiosperm | Fabaceae | *Vigna luteola* | AY583519 |
| Angiosperm | Fabaceae | *Vigna peduncularis* | AF069122 |
| Angiosperm | Fabaceae | *Vigna peduncularis* | AY583523 |
| Angiosperm | Fabaceae | *Vigna populnea* | AF115136 |
| Angiosperm | Fabaceae | *Vigna radiata* | EU288914 |
| Angiosperm | Fabaceae | *Vigna radiata* | EU727150 |
| Angiosperm | Fabaceae | *Vigna speciosa* | AF069121 |
| Angiosperm | Fabaceae | *Vigna spectabilis* | AY583524 |
| Angiosperm | Fabaceae | *Vigna trichocarpa* | AY583521 |
| Angiosperm | Fabaceae | *Vigna trichocarpa* | DQ445737 |
| Angiosperm | Fabaceae | *Vigna umbellata* | EU727151 |
| Angiosperm | Fabaceae | *Vigna unguiculata* | DQ445736 |
| Angiosperm | Fabaceae | *Vigna unguiculata* | EU288907 |
| Angiosperm | Fabaceae | *Vigna unguiculata* | EU288908 |
| Angiosperm | Fabaceae | *Vigna unguiculata* | EU288909 |
| Angiosperm | Fabaceae | *Vigna unguiculata* | EU288910 |
| Angiosperm | Fabaceae | *Vigna unguiculata* | EU288911 |
| Angiosperm | Fabaceae | *Vigna unguiculata* | EU693524 |
| Angiosperm | Fabaceae | *Vigna unguiculata* subsp. *cylindrica* | EU727149 |
| Angiosperm | Fabaceae | *Vigna unguiculata* subsp. *sesquipedalis* | EU727148 |
| Angiosperm | Flacourtiaceae | *Xylosma congesta* | DQ521290 |
| Angiosperm | Gentianaceae | *Gentiana asclepiadea* | AJ580549 |
| Angiosperm | Gentianaceae | *Gentiana lilliputiana* | AY160218 |
| Angiosperm | Gentianaceae | *Gentiana pleurogynoides* | AY136506 |
| Angiosperm | Gentianaceae | *Swertia perennis* | AJ580550 |
| Angiosperm | Geraniaceae | *Erodium stephanianum* | DQ345327 |
| Angiosperm | Geraniaceae | *Geranium brevicaule* | EU044753 |
| Angiosperm | Geraniaceae | *Geranium crenophilum* | AY944412 |
| Angiosperm | Geraniaceae | *Geranium dissectum* | AY944413 |
| Angiosperm | Geraniaceae | *Geranium gymnocaulon* | AJ884926 |
| Angiosperm | Geraniaceae | *Geranium homeanum* | AY752471 |
| Angiosperm | Geraniaceae | *Geranium homeanum* | EU044757 |
| Angiosperm | Geraniaceae | *Geranium ibericum* | AJ884928 |
| Angiosperm | Geraniaceae | *Geranium ibericum* | AJ884936 |
| Angiosperm | Geraniaceae | *Geranium lanuginosum* | AJ884942 |
| Angiosperm | Geraniaceae | *Geranium lanuginosum* | AJ884943 |
| Angiosperm | Geraniaceae | *Geranium libani* | AJ884932 |
| Angiosperm | Geraniaceae | *Geranium microphyllum* | EU044751 |
| Angiosperm | Geraniaceae | *Geranium microphyllum* | EU044759 |
| Angiosperm | Geraniaceae | *Geranium palmatum* | AY944411 |
| Angiosperm | Geraniaceae | *Geranium potentilloides* | EU044758 |
| Angiosperm | Geraniaceae | *Geranium retrorsum* | AY752473 |
| Angiosperm | Geraniaceae | *Geranium retrorsum* | EU044749 |
| Angiosperm | Geraniaceae | *Geranium sessiliflorum* var. *arenarium* | AY752474 |
| Angiosperm | Geraniaceae | *Geranium sessiliflorum* var. *arenarum* | EU044756 |
| Angiosperm | Geraniaceae | *Geranium sibiricum* | DQ309044 |
| Angiosperm | Geraniaceae | *Geranium solanderi* | AY752467 |
| Angiosperm | Geraniaceae | *Geranium traversii* | EU044755 |
| Angiosperm | Geraniaceae | *Geranium tuberosum* | AJ884946 |
| Angiosperm | Geraniaceae | *Pelargonium aestivale* | AY036218 |
| Angiosperm | Geraniaceae | *Pelargonium appendiculatum* | AY036227 |
| Angiosperm | Geraniaceae | *Pelargonium aristatum* | AY036206 |
| Angiosperm | Geraniaceae | *Pelargonium asarifolium* | AY036208 |
| Angiosperm | Geraniaceae | *Pelargonium auritum* subsp. *auritum* | AY036184 |
| Angiosperm | Geraniaceae | *Pelargonium auritum* subsp. *carneum* | AY036183 |
| Angiosperm | Geraniaceae | *Pelargonium carneum* | AY036180 |
| Angiosperm | Geraniaceae | *Pelargonium carneum* | AY036181 |
| Angiosperm | Geraniaceae | *Pelargonium caroli-henrici* | AY036220 |
| Angiosperm | Geraniaceae | *Pelargonium confertum* | AY036216 |
| Angiosperm | Geraniaceae | *Pelargonium connivens* | AY036198 |
| Angiosperm | Geraniaceae | *Pelargonium denticulatum* | AY352883 |
| Angiosperm | Geraniaceae | *Pelargonium ellaphieae* | AY036203 |
| Angiosperm | Geraniaceae | *Pelargonium fasciculaceum* | AY036188 |
| Angiosperm | Geraniaceae | *Pelargonium fissifolium* | AY036205 |
| Angiosperm | Geraniaceae | *Pelargonium fumariifolium* | AY036217 |
| Angiosperm | Geraniaceae | *Pelargonium glabriphyllum* | AY036200 |
| Angiosperm | Geraniaceae | *Pelargonium grenvilleae* | AY036210 |
| Angiosperm | Geraniaceae | *Pelargonium incrassatum* | AY036190 |
| Angiosperm | Geraniaceae | *Pelargonium leptum* | AY036192 |
| Angiosperm | Geraniaceae | *Pelargonium luridum* | AF256560 |
| Angiosperm | Geraniaceae | *Pelargonium minimum* | AF256539 |
| Angiosperm | Geraniaceae | *Pelargonium moniliforme* | AY036222 |
| Angiosperm | Geraniaceae | *Pelargonium myrrhifolium* var. *myrrhifolium* | AY352889 |
| Angiosperm | Geraniaceae | *Pelargonium parvipetalum* | AY036197 |
| Angiosperm | Geraniaceae | *Pelargonium petroselinifolium* | AY036207 |
| Angiosperm | Geraniaceae | *Pelargonium pinnatum* | AY036225 |
| Angiosperm | Geraniaceae | *Pelargonium quarciticola* | AY036211 |
| Angiosperm | Geraniaceae | *Pelargonium rapaceum* | AY036185 |
| Angiosperm | Geraniaceae | *Pelargonium reflexipetalum* | AY036199 |
| Angiosperm | Geraniaceae | *Pelargonium stipulaceum* | AF256550 |
| Angiosperm | Geraniaceae | *Pelargonium triandrum* | AY036212 |
| Angiosperm | Geraniaceae | *Pelargonium triphyllum* | AY036204 |
| Angiosperm | Geraniaceae | *Pelargonium undulatum* | AY036186 |
| Angiosperm | Geraniaceae | *Pelargonium vinaceum* | AY036223 |
| Angiosperm | Geraniaceae | *Pelargonium vitifolium* | AY352894 |
| Angiosperm | Geraniaceae | *Pelargonium zonale* | DQ345326 |
| Angiosperm | Grossulariaceae | *Ribes alpinum* | AJ293765 |
| Angiosperm | Grossulariaceae | *Ribes aureum* | AJ293766 |
| Angiosperm | Grossulariaceae | *Ribes nigrum* | AJ297579 |
| Angiosperm | Grossulariaceae | *Ribes spicatum* | AJ297580 |
| Angiosperm | Grossulariaceae | *Ribes uva-crispa* | AJ297578 |
| Angiosperm | Guttiferae | *Calophyllum inophyllum* | AJ312608 |
| Angiosperm | Guttiferae | *Calophyllum longifolium* | AJ312609 |
| Angiosperm | Hamamelidaceae | *Liquidambar acalycina* | AF133231 |
| Angiosperm | Hamamelidaceae | *Liquidambar formosana* | AF015436 |
| Angiosperm | Hamamelidaceae | *Liquidambar formosana* | AF133230 |
| Angiosperm | Hamamelidaceae | *Liquidambar orientalis* | AF133229 |
| Angiosperm | Hamamelidaceae | *Liquidambar styraciflua* | AF055886 |
| Angiosperm | Hamamelidaceae | *Liquidambar styraciflua* | AF133227 |
| Angiosperm | Hamamelidaceae | *Liquidambar styraciflua* | AF133228 |
| Angiosperm | Hippocastanaceae | *Aesculus wangii* | AF406968 |
| Angiosperm | Iridaceae | *Belamcanda chinensis* | DQ277644 |
| Angiosperm | Iridaceae | *Crocus sativus* | DQ094185 |
| Angiosperm | Iridaceae | *Crocus vernus* | AM503892 |
| Angiosperm | Iridaceae | *Crocus vernus* | DQ224363 |
| Angiosperm | Iridaceae | *Crocus vernus* | DQ224364 |
| Angiosperm | Iridaceae | *Iris lactea* | DQ277639 |
| Angiosperm | Iridaceae | *Iris mandshurica* | DQ277642 |
| Angiosperm | Iridaceae | *Iris pseudacorus* | DQ277645 |
| Angiosperm | Iridaceae | *Iris ruthenica* | DQ277640 |
| Angiosperm | Iridaceae | *Iris uniflora* | DQ277641 |
| Angiosperm | Juglandaceae | *Juglans californica* | AF338474 |
| Angiosperm | Juglandaceae | *Juglans nigra* | AF338489 |
| Angiosperm | Juglandaceae | *Juglans nigra* | AF338490 |
| Angiosperm | Juglandaceae | *Juglans nigra* | AF338491 |
| Angiosperm | Juglandaceae | *Juglans nigra* | AF338492 |
| Angiosperm | Juncaceae | *Juncus lomatophyllus* | AY973503 |
| Angiosperm | Juncaceae | *Juncus oxymeris* | AY973505 |
| Angiosperm | Juncaceae | *Juncus trifidus* | AY973508 |
| Angiosperm | Labiatae | *Hyptis alata* | DQ667235 |
| Angiosperm | Lamiaceae | *Acanthomintha lanceolata* | DQ667333 |
| Angiosperm | Lamiaceae | *Agastache cana* | AJ421000 |
| Angiosperm | Lamiaceae | *Agastache pallida* | AJ421001 |
| Angiosperm | Lamiaceae | *Agastache urticifolia* | DQ667247 |
| Angiosperm | Lamiaceae | *Ballota hispanica* | AF335641 |
| Angiosperm | Lamiaceae | *Cleonia lusitanica* | DQ667309 |
| Angiosperm | Lamiaceae | *Clinopodium ashei* | DQ667237 |
| Angiosperm | Lamiaceae | *Clinopodium axillare* | DQ017565 |
| Angiosperm | Lamiaceae | *Clinopodium bolivianum* | DQ017564 |
| Angiosperm | Lamiaceae | *Clinopodium cylindristachys* | DQ017562 |
| Angiosperm | Lamiaceae | *Clinopodium fasciculatum* | DQ017558 |
| Angiosperm | Lamiaceae | *Clinopodium gilliesii* | DQ017566 |
| Angiosperm | Lamiaceae | *Clinopodium jacquelinae* | DQ017557 |
| Angiosperm | Lamiaceae | *Clinopodium nubigenum* | DQ017563 |
| Angiosperm | Lamiaceae | *Clinopodium sericeum* | DQ017561 |
| Angiosperm | Lamiaceae | *Clinopodium speciosum* | DQ017560 |
| Angiosperm | Lamiaceae | *Clinopodium tomentosum* | DQ017559 |
| Angiosperm | Lamiaceae | *Clinopodium vanum* | DQ017568 |
| Angiosperm | Lamiaceae | *Conradina canescens* | DQ667238 |
| Angiosperm | Lamiaceae | *Cunila galioides* | DQ667305 |
| Angiosperm | Lamiaceae | *Dicerandra odoratissima* | DQ667234 |
| Angiosperm | Lamiaceae | *Dorystaechas hastata* | DQ667252 |
| Angiosperm | Lamiaceae | *Dracocephalum grandiflorum* | AJ420999 |
| Angiosperm | Lamiaceae | *Dracocephalum kotschyi* | AJ420998 |
| Angiosperm | Lamiaceae | *Faradaya splendida* | U77773 |
| Angiosperm | Lamiaceae | *Glechoma hederacea* | DQ006014 |
| Angiosperm | Lamiaceae | *Glechon marifolia* | DQ667303 |
| Angiosperm | Lamiaceae | *Glechon thymoides* | DQ667310 |
| Angiosperm | Lamiaceae | *Lallemantia peltata* | AJ420997 |
| Angiosperm | Lamiaceae | *Lamium purpureum* | AM503882 |
| Angiosperm | Lamiaceae | *Leonurus chaituroides* | DQ903316 |
| Angiosperm | Lamiaceae | *Leonurus japonicus* | DQ903314 |
| Angiosperm | Lamiaceae | *Leonurus pseudomacranthus* | DQ903315 |
| Angiosperm | Lamiaceae | *Leonurus sibiricus* | DQ903317 |
| Angiosperm | Lamiaceae | *Leonurus sibiricus* | EF395805 |
| Angiosperm | Lamiaceae | *Leonurus turkestanicus* | EF395809 |
| Angiosperm | Lamiaceae | *Lepechinia conferta* | DQ667307 |
| Angiosperm | Lamiaceae | *Lepechinia lancifolia* | DQ667306 |
| Angiosperm | Lamiaceae | *Lycopus uniflorus* | DQ667302 |
| Angiosperm | Lamiaceae | *Mentha arvensis* | DQ667325 |
| Angiosperm | Lamiaceae | *Mentha spicata* | DQ667244 |
| Angiosperm | Lamiaceae | *Monarda fistulosa* | DQ667318 |
| Angiosperm | Lamiaceae | *Nepeta assurgens* | AJ515316 |
| Angiosperm | Lamiaceae | *Nepeta balouchestanica* | AJ515606 |
| Angiosperm | Lamiaceae | *Nepeta binaloudensis* | AJ515311 |
| Angiosperm | Lamiaceae | *Nepeta cataria* | AJ515313 |
| Angiosperm | Lamiaceae | *Nepeta congesta* var. *cryptantha* | AJ515161 |
| Angiosperm | Lamiaceae | *Nepeta crassifolia* | AJ515307 |
| Angiosperm | Lamiaceae | *Nepeta crispa* | AJ515159 |
| Angiosperm | Lamiaceae | *Nepeta denudata* | AJ515304 |
| Angiosperm | Lamiaceae | *Nepeta gloeocephala* | AJ515308 |
| Angiosperm | Lamiaceae | *Nepeta glomerulosa* | AJ515317 |
| Angiosperm | Lamiaceae | *Nepeta heliotropifolia* | AJ515312 |
| Angiosperm | Lamiaceae | *Nepeta hormozganica* | AJ515160 |
| Angiosperm | Lamiaceae | *Nepeta isaurica* | AJ515306 |
| Angiosperm | Lamiaceae | *Nepeta ispahanica* | AJ515318 |
| Angiosperm | Lamiaceae | *Nepeta kurdica* | AJ515320 |
| Angiosperm | Lamiaceae | *Nepeta laxiflora* | AJ420995 |
| Angiosperm | Lamiaceae | *Nepeta menthoides* | AJ421002 |
| Angiosperm | Lamiaceae | *Nepeta mirzayanii* | AJ515309 |
| Angiosperm | Lamiaceae | *Nepeta mussinii* | AJ515305 |
| Angiosperm | Lamiaceae | *Nepeta oxyodonta* | AJ420996 |
| Angiosperm | Lamiaceae | *Nepeta saccharata* | AJ515314 |
| Angiosperm | Lamiaceae | *Nepeta scrophularioides* | AJ515319 |
| Angiosperm | Lamiaceae | *Ocimum basilicum* | DQ667240 |
| Angiosperm | Lamiaceae | *Origanum vulgare* | DQ667243 |
| Angiosperm | Lamiaceae | *Perilla frutescens* | DQ667246 |
| Angiosperm | Lamiaceae | *Perovskia scrophulariifolia* | DQ667330 |
| Angiosperm | Lamiaceae | *Phlomis crinita* subsp. *crinita* | AY792815 |
| Angiosperm | Lamiaceae | *Phlomis crinita* subsp. *malacitana* | AY792794 |
| Angiosperm | Lamiaceae | *Phlomis crinita* subsp. *malacitana* | AY792806 |
| Angiosperm | Lamiaceae | *Phlomis crinita* subsp. *malacitana* | AY792807 |
| Angiosperm | Lamiaceae | *Phlomis crinita* subsp. *mauritanica* | AY792818 |
| Angiosperm | Lamiaceae | *Phlomis purpurea* | AY792819 |
| Angiosperm | Lamiaceae | *Pogostemon cablin* | EF529538 |
| Angiosperm | Lamiaceae | *Pogostemon cablin* | EF529542 |
| Angiosperm | Lamiaceae | *Poliomintha palmeri* | DQ667311 |
| Angiosperm | Lamiaceae | *Prunella asiatica* | AY947422 |
| Angiosperm | Lamiaceae | *Prunella vulgaris* | AY506653 |
| Angiosperm | Lamiaceae | *Pycnanthemum virginianum* | DQ667319 |
| Angiosperm | Lamiaceae | *Salvia aegyptiaca* | DQ667285 |
| Angiosperm | Lamiaceae | *Salvia aethiopis* | DQ667272 |
| Angiosperm | Lamiaceae | *Salvia apiana* | DQ667214 |
| Angiosperm | Lamiaceae | *Salvia aristata* | DQ667280 |
| Angiosperm | Lamiaceae | *Salvia axillaris* var. *axillaris* | DQ667294 |
| Angiosperm | Lamiaceae | *Salvia azurea* | DQ667317 |
| Angiosperm | Lamiaceae | *Salvia brevilabra* | EF373636 |
| Angiosperm | Lamiaceae | *Salvia brevilabra* | EF373637 |
| Angiosperm | Lamiaceae | *Salvia brevilabra* | EF373638 |
| Angiosperm | Lamiaceae | *Salvia cacaliifolia* | DQ667259 |
| Angiosperm | Lamiaceae | *Salvia californica* | DQ667213 |
| Angiosperm | Lamiaceae | *Salvia canariensis* | DQ667256 |
| Angiosperm | Lamiaceae | *Salvia candicans* | DQ667299 |
| Angiosperm | Lamiaceae | *Salvia candidissima* | DQ667261 |
| Angiosperm | Lamiaceae | *Salvia cavaleriei* var. *simplicifolia* | EF373618 |
| Angiosperm | Lamiaceae | *Salvia cavaleriei* var. *simplicifolia* | EF373619 |
| Angiosperm | Lamiaceae | *Salvia cavaleriei* var. *simplicifolia* | EF373620 |
| Angiosperm | Lamiaceae | *Salvia cedrosensis* | DQ667228 |
| Angiosperm | Lamiaceae | *Salvia chienii* | DQ132868 |
| Angiosperm | Lamiaceae | *Salvia clevelandii* | DQ667219 |
| Angiosperm | Lamiaceae | *Salvia cynica* | DQ667332 |
| Angiosperm | Lamiaceae | *Salvia cynica* | EF373639 |
| Angiosperm | Lamiaceae | *Salvia cynica* | EF373640 |
| Angiosperm | Lamiaceae | *Salvia cynica* | EF373641 |
| Angiosperm | Lamiaceae | *Salvia daghestanica* | DQ667258 |
| Angiosperm | Lamiaceae | *Salvia davidsonii* | AF538919 |
| Angiosperm | Lamiaceae | *Salvia deserta* | DQ132865 |
| Angiosperm | Lamiaceae | *Salvia divinorum* | DQ667249 |
| Angiosperm | Lamiaceae | *Salvia dolomitica* | DQ667322 |
| Angiosperm | Lamiaceae | *Salvia dorrii* subsp. *mearnsii* | AF538901 |
| Angiosperm | Lamiaceae | *Salvia dorrii* var. *dorrii* | AF543682 |
| Angiosperm | Lamiaceae | *Salvia evansiana* | EF373622 |
| Angiosperm | Lamiaceae | *Salvia evansiana* | EF373623 |
| Angiosperm | Lamiaceae | *Salvia fulgens* | DQ667251 |
| Angiosperm | Lamiaceae | *Salvia glutinosa* | DQ667250 |
| Angiosperm | Lamiaceae | *Salvia greatae* | DQ667215 |
| Angiosperm | Lamiaceae | *Salvia haenkei* | DQ667271 |
| Angiosperm | Lamiaceae | *Salvia henryi* | DQ667216 |
| Angiosperm | Lamiaceae | *Salvia inconspicua* | DQ667298 |
| Angiosperm | Lamiaceae | *Salvia lasiantha* | DQ667300 |
| Angiosperm | Lamiaceae | *Salvia lavanduloides* | DQ667297 |
| Angiosperm | Lamiaceae | *Salvia miltiorrhiza* | EF373601 |
| Angiosperm | Lamiaceae | *Salvia miltiorrhiza* | EF373603 |
| Angiosperm | Lamiaceae | *Salvia miltiorrhiza* | EF373604 |
| Angiosperm | Lamiaceae | *Salvia miltiorrhiza* | EF373606 |
| Angiosperm | Lamiaceae | *Salvia miltiorrhiza* | EF373608 |
| Angiosperm | Lamiaceae | *Salvia miltiorrhiza* | EF373609 |
| Angiosperm | Lamiaceae | *Salvia miltiorrhiza* | EF373610 |
| Angiosperm | Lamiaceae | *Salvia miltiorrhiza f. alba* | EF373611 |
| Angiosperm | Lamiaceae | *Salvia miltiorrhiza f. alba* | EF373612 |
| Angiosperm | Lamiaceae | *Salvia miltiorrhiza f. alba* | EF373613 |
| Angiosperm | Lamiaceae | *Salvia mocinoi* | DQ667274 |
| Angiosperm | Lamiaceae | *Salvia mohavensis* | AF538920 |
| Angiosperm | Lamiaceae | *Salvia mohavensis* | AF538921 |
| Angiosperm | Lamiaceae | *Salvia ovalifolia* | DQ667315 |
| Angiosperm | Lamiaceae | *Salvia oxyphora* | DQ667262 |
| Angiosperm | Lamiaceae | *Salvia pachyphylla* | AF538906 |
| Angiosperm | Lamiaceae | *Salvia pachyphylla* | AF538907 |
| Angiosperm | Lamiaceae | *Salvia pachyphylla* | AF538908 |
| Angiosperm | Lamiaceae | *Salvia pachyphylla* | AF538909 |
| Angiosperm | Lamiaceae | *Salvia pachyphylla* | AF538910 |
| Angiosperm | Lamiaceae | *Salvia pachyphylla* | AF538911 |
| Angiosperm | Lamiaceae | *Salvia pachyphylla* | AF538912 |
| Angiosperm | Lamiaceae | *Salvia pachyphylla* | AF538913 |
| Angiosperm | Lamiaceae | *Salvia pachyphylla* | AF538915 |
| Angiosperm | Lamiaceae | *Salvia pachyphylla* | AF538916 |
| Angiosperm | Lamiaceae | *Salvia pachyphylla* | AF538917 |
| Angiosperm | Lamiaceae | *Salvia pachyphylla* | AF538918 |
| Angiosperm | Lamiaceae | *Salvia pentstemonoides* | DQ667221 |
| Angiosperm | Lamiaceae | *Salvia personata* | DQ667269 |
| Angiosperm | Lamiaceae | *Salvia platystoma* | DQ667277 |
| Angiosperm | Lamiaceae | *Salvia polystachya* | DQ667292 |
| Angiosperm | Lamiaceae | *Salvia prunelloides* | DQ667275 |
| Angiosperm | Lamiaceae | *Salvia przewalskii* | EF373628 |
| Angiosperm | Lamiaceae | *Salvia przewalskii* | EF373629 |
| Angiosperm | Lamiaceae | *Salvia pubescens* | DQ667296 |
| Angiosperm | Lamiaceae | *Salvia roemeriana* | DQ667211 |
| Angiosperm | Lamiaceae | *Salvia rugosa* | DQ667290 |
| Angiosperm | Lamiaceae | *Salvia rusbyi* | DQ667278 |
| Angiosperm | Lamiaceae | *Salvia rypara* | DQ667266 |
| Angiosperm | Lamiaceae | *Salvia sagittata* | DQ667260 |
| Angiosperm | Lamiaceae | *Salvia sclarea* | DQ667222 |
| Angiosperm | Lamiaceae | *Salvia scutellarioides* | DQ667327 |
| Angiosperm | Lamiaceae | *Salvia semiatrata* | DQ667295 |
| Angiosperm | Lamiaceae | *Salvia sessilifolia* | DQ667282 |
| Angiosperm | Lamiaceae | *Salvia stachydifolia* | DQ667267 |
| Angiosperm | Lamiaceae | *Salvia summa* | DQ667217 |
| Angiosperm | Lamiaceae | *Salvia taraxacifolia* | DQ667209 |
| Angiosperm | Lamiaceae | *Salvia texana* | DQ667321 |
| Angiosperm | Lamiaceae | *Salvia thymoides* | DQ667273 |
| Angiosperm | Lamiaceae | *Salvia trichocalycina* | DQ667283 |
| Angiosperm | Lamiaceae | *Salvia trijuga* | DQ132870 |
| Angiosperm | Lamiaceae | *Salvia verbascifolia* | DQ667264 |
| Angiosperm | Lamiaceae | *Salvia whitehousei* | DQ667320 |
| Angiosperm | Lamiaceae | *Salvia yunnanensis* | DQ132866 |
| Angiosperm | Lamiaceae | *Salvia yunnanensis* | EF373615 |
| Angiosperm | Lamiaceae | *Salvia yunnanensis* | EF373616 |
| Angiosperm | Lamiaceae | *Schizonepeta multifida* | DQ667313 |
| Angiosperm | Lamiaceae | *Schizonepeta tenuifolia* | EU383034 |
| Angiosperm | Lamiaceae | *Scutellaria baicalensis* | AY394851 |
| Angiosperm | Lamiaceae | *Scutellaria barbata* | DQ813302 |
| Angiosperm | Lamiaceae | *Sideritis algarviensis* | AF335623 |
| Angiosperm | Lamiaceae | *Sideritis athoa* | AF335615 |
| Angiosperm | Lamiaceae | *Sideritis barbellata* | DQ900750 |
| Angiosperm | Lamiaceae | *Sideritis brevicaulis* | DQ900751 |
| Angiosperm | Lamiaceae | *Sideritis canariensis* | AF335605 |
| Angiosperm | Lamiaceae | *Sideritis canariensis* | DQ900753 |
| Angiosperm | Lamiaceae | *Sideritis candicans* | AF335606 |
| Angiosperm | Lamiaceae | *Sideritis clandestina* | AF335616 |
| Angiosperm | Lamiaceae | *Sideritis cossoniana* | AF335613 |
| Angiosperm | Lamiaceae | *Sideritis cretica* subsp. *cretica* | DQ900754 |
| Angiosperm | Lamiaceae | *Sideritis cretica* subsp. *spicata* | DQ900755 |
| Angiosperm | Lamiaceae | *Sideritis cystosiphon* | DQ900756 |
| Angiosperm | Lamiaceae | *Sideritis dendro-chahorra* | DQ900758 |
| Angiosperm | Lamiaceae | *Sideritis discolor* | DQ900759 |
| Angiosperm | Lamiaceae | *Sideritis endressii* subsp. *emporitana* | AF335628 |
| Angiosperm | Lamiaceae | *Sideritis eriocephala* | AF335607 |
| Angiosperm | Lamiaceae | *Sideritis euboae* | AF335617 |
| Angiosperm | Lamiaceae | *Sideritis ferrensis* | DQ900760 |
| Angiosperm | Lamiaceae | *Sideritis hyssopifolia* | AF335633 |
| Angiosperm | Lamiaceae | *Sideritis infernalis* | DQ900762 |
| Angiosperm | Lamiaceae | *Sideritis kuegleriana* | DQ900763 |
| Angiosperm | Lamiaceae | *Sideritis lotsyi* | DQ900764 |
| Angiosperm | Lamiaceae | *Sideritis macrostachys* | AF335609 |
| Angiosperm | Lamiaceae | *Sideritis marmorea* | DQ900765 |
| Angiosperm | Lamiaceae | *Sideritis nervosa* | DQ900766 |
| Angiosperm | Lamiaceae | *Sideritis nutans* | DQ900767 |
| Angiosperm | Lamiaceae | *Sideritis scardica* | AF335619 |
| Angiosperm | Lamiaceae | *Sideritis sventenii* | DQ900772 |
| Angiosperm | Lamiaceae | *Sideritis syriaca* | AF335620 |
| Angiosperm | Lamiaceae | *Sideritis syriaca* | AF335621 |
| Angiosperm | Lamiaceae | *Sideritis taurica* | AF335622 |
| Angiosperm | Lamiaceae | *Stachys byzantina* | AJ420994 |
| Angiosperm | Lamiaceae | *Stachys hirta* | AF335643 |
| Angiosperm | Lamiaceae | *Thymus persicus* | EU735058 |
| Angiosperm | Lamiaceae | *Thymus serpyllum* | DQ667242 |
| Angiosperm | Lamiaceae | *Thymus trautvetteri* | EU735059 |
| Angiosperm | Lardizabalaceae | *Akebia quinata* | AY029791 |
| Angiosperm | Lardizabalaceae | *Akebia trifoliata* subsp. *australis* | AY029788 |
| Angiosperm | Lardizabalaceae | *Sargentodoxa cuneata* | EF076045 |
| Angiosperm | Lardizabalaceae | *Sargentodoxa cuneata* | EF076047 |
| Angiosperm | Lardizabalaceae | *Sargentodoxa cuneata* | EF076049 |
| Angiosperm | Lardizabalaceae | *Sargentodoxa cuneata* | EF076050 |
| Angiosperm | Lardizabalaceae | *Sargentodoxa cuneata* | EF076051 |
| Angiosperm | Lardizabalaceae | *Sargentodoxa cuneata* | EF076053 |
| Angiosperm | Lardizabalaceae | *Sargentodoxa cuneata* | EF076054 |
| Angiosperm | Lardizabalaceae | *Sargentodoxa simplicifolia* | EF076052 |
| Angiosperm | Lauraceae | *Cinnamomum cinnamomifolium* | AF272262 |
| Angiosperm | Lauraceae | *Cinnamomum pittosporoides* | DQ124269 |
| Angiosperm | Lauraceae | *Lindera chunii* | DQ124266 |
| Angiosperm | Lauraceae | *Lindera erythrocarpa* | AB260855 |
| Angiosperm | Lauraceae | *Lindera kariensis* | DQ124263 |
| Angiosperm | Lauraceae | *Lindera latifolia* | DQ124264 |
| Angiosperm | Lauraceae | *Lindera longipedunculata* | DQ124265 |
| Angiosperm | Lauraceae | *Lindera lucida* | AB260856 |
| Angiosperm | Lauraceae | *Lindera metcalfiana* | AY265408 |
| Angiosperm | Lauraceae | *Lindera polyantha* | AB260858 |
| Angiosperm | Lauraceae | *Lindera pulcherrima* | DQ124267 |
| Angiosperm | Lauraceae | *Lindera reflexa* | AY265407 |
| Angiosperm | Lauraceae | *Lindera umbellata* | AB260859 |
| Angiosperm | Lauraceae | *Lindera villipes* | DQ124268 |
| Angiosperm | Lauraceae | *Litsea accedens* | AB260860 |
| Angiosperm | Lauraceae | *Litsea acutivena* | DQ120605 |
| Angiosperm | Lauraceae | *Litsea caulocarpa* | AB260861 |
| Angiosperm | Lauraceae | *Litsea costalis* | AB260862 |
| Angiosperm | Lauraceae | *Litsea cubeba* | AB260863 |
| Angiosperm | Lauraceae | *Litsea cubeba* | AY265402 |
| Angiosperm | Lauraceae | *Litsea dilleniifolia* | AY265405 |
| Angiosperm | Lauraceae | *Litsea diversifolia* | AB260864 |
| Angiosperm | Lauraceae | *Litsea elongata* | DQ120606 |
| Angiosperm | Lauraceae | *Litsea erectinervia* | AB260865 |
| Angiosperm | Lauraceae | *Litsea fenestrata* | AB260866 |
| Angiosperm | Lauraceae | *Litsea ferruginea* | AB260867 |
| Angiosperm | Lauraceae | *Litsea firma* | AB260868 |
| Angiosperm | Lauraceae | *Litsea garciae* | AB260869 |
| Angiosperm | Lauraceae | *Litsea garrettii* | DQ120607 |
| Angiosperm | Lauraceae | *Litsea globularia* | AB260870 |
| Angiosperm | Lauraceae | *Litsea glutinosa* | AB260871 |
| Angiosperm | Lauraceae | *Litsea glutinosa* | AY265403 |
| Angiosperm | Lauraceae | *Litsea grandis* | AB260872 |
| Angiosperm | Lauraceae | *Litsea kingii* | DQ120599 |
| Angiosperm | Lauraceae | *Litsea lancifolia* var. *grandifolia* | AB260873 |
| Angiosperm | Lauraceae | *Litsea liyuyingi* | DQ120608 |
| Angiosperm | Lauraceae | *Litsea longistaminata* | DQ120609 |
| Angiosperm | Lauraceae | *Litsea machilifolia* | AB260874 |
| Angiosperm | Lauraceae | *Litsea maingayi* | AB260875 |
| Angiosperm | Lauraceae | *Litsea mappacea* | AB260876 |
| Angiosperm | Lauraceae | *Litsea monopetala* | DQ120602 |
| Angiosperm | Lauraceae | *Litsea noronhae* | AB260877 |
| Angiosperm | Lauraceae | *Litsea ochracea* | AB260878 |
| Angiosperm | Lauraceae | *Litsea panamanja* | DQ120610 |
| Angiosperm | Lauraceae | *Litsea resinosa* | AB260879 |
| Angiosperm | Lauraceae | *Litsea rubicunda* | AB260880 |
| Angiosperm | Lauraceae | *Litsea sarawacensis* | AB260881 |
| Angiosperm | Lauraceae | *Litsea sericea* | DQ120601 |
| Angiosperm | Lauraceae | *Litsea tomentosa* | AB260882 |
| Angiosperm | Lauraceae | *Litsea umbellata* | AB260883 |
| Angiosperm | Lauraceae | *Litsea umbellata* | AY265404 |
| Angiosperm | Lauraceae | *Litsea variabilis* | DQ120603 |
| Angiosperm | Lauraceae | *Litsea variabilis* var. *oblonga* | DQ120604 |
| Angiosperm | Lauraceae | *Litsea yaoshanensis* | DQ120611 |
| Angiosperm | Lauraceae | *Machilus rimosa* | AB260888 |
| Angiosperm | Leguminosae | *Pterocarpus acapulcensis* | AF269175 |
| Angiosperm | Leguminosae | *Pterocarpus indicus* | AF269177 |
| Angiosperm | Leguminosae | *Pterocarpus macrocarpus* | AF269176 |
| Angiosperm | Leguminosae | *Pterocarpus rohrii* | EF451061 |
| Angiosperm | Liliaceae | *Allium abramsii* | EU096131 |
| Angiosperm | Liliaceae | *Allium amplectens* | EU096133 |
| Angiosperm | Liliaceae | *Allium anceps* | EU096135 |
| Angiosperm | Liliaceae | *Allium asarense* | AM418365 |
| Angiosperm | Liliaceae | *Allium atrorubens* var. *cristatum* | EU096138 |
| Angiosperm | Liliaceae | *Allium beesianum* | AJ411860 |
| Angiosperm | Liliaceae | *Allium bolanderi* var. *bolanderi* | EU096141 |
| Angiosperm | Liliaceae | *Allium brevistylum* | AJ412763 |
| Angiosperm | Liliaceae | *Allium caeruleum* | AJ412729 |
| Angiosperm | Liliaceae | *Allium caesium* | AJ412731 |
| Angiosperm | Liliaceae | *Allium campanulatum* | EU096144 |
| Angiosperm | Liliaceae | *Allium canadense* var. *canadense* | EU096145 |
| Angiosperm | Liliaceae | *Allium cepa* | AM492188 |
| Angiosperm | Liliaceae | *Allium cernuum* | AY427533 |
| Angiosperm | Liliaceae | *Allium chamaemoly* | AF055109 |
| Angiosperm | Liliaceae | *Allium cratericola* | EU096146 |
| Angiosperm | Liliaceae | *Allium crispum* | EU096147 |
| Angiosperm | Liliaceae | *Allium cupanii* | AJ412737 |
| Angiosperm | Liliaceae | *Allium cyathophorum* | EU096148 |
| Angiosperm | Liliaceae | *Allium daghestanicum* | AJ411850 |
| Angiosperm | Liliaceae | *Allium dentigerum* | AJ411958 |
| Angiosperm | Liliaceae | *Allium dichlamydeum* | EU096152 |
| Angiosperm | Liliaceae | *Allium drepanophyllum* | AJ411854 |
| Angiosperm | Liliaceae | *Allium eduardii* | AJ412756 |
| Angiosperm | Liliaceae | *Aloe acutissima* | AF234348 |
| Angiosperm | Liliaceae | *Aloe ammophila* | AF234347 |
| Angiosperm | Liliaceae | *Aloe arborescens* | AF234333 |
| Angiosperm | Liliaceae | *Aloe bakeri* | AF234346 |
| Angiosperm | Liliaceae | *Aloe cameronii* | AF234343 |
| Angiosperm | Liliaceae | *Aloe ciliaris* | AF234324 |
| Angiosperm | Liliaceae | *Aloe distans* | AF234327 |
| Angiosperm | Liliaceae | *Aloe ferox* | AF234338 |
| Angiosperm | Liliaceae | *Aloe forbesii* | AF234342 |
| Angiosperm | Liliaceae | *Aloe glauca* | AF234344 |
| Angiosperm | Liliaceae | *Aloe inermis* | AF234328 |
| Angiosperm | Liliaceae | *Aloe jucunda* | AF234337 |
| Angiosperm | Liliaceae | *Aloe juvenna* | AF234349 |
| Angiosperm | Liliaceae | *Aloe morijensis* | AF234325 |
| Angiosperm | Liliaceae | *Aloe ngobitensis* | AF234322 |
| Angiosperm | Liliaceae | *Aloe nyeriensis* | AF234339 |
| Angiosperm | Liliaceae | *Aloe peckii* | AF234323 |
| Angiosperm | Liliaceae | *Aloe pendens* | AF234340 |
| Angiosperm | Liliaceae | *Aloe penduliflora* | AF234330 |
| Angiosperm | Liliaceae | *Aloe perryi* | AF234341 |
| Angiosperm | Liliaceae | *Aloe scobinifolia* | AF234331 |
| Angiosperm | Liliaceae | *Aloe somaliensis* | AF234334 |
| Angiosperm | Liliaceae | *Aloe tenuior* | AF234326 |
| Angiosperm | Liliaceae | *Aloe vera* | AF234345 |
| Angiosperm | Liliaceae | *Aloe verdoorniae* | AF234332 |
| Angiosperm | Liliaceae | *Asparagus officinalis* | U24024 |
| Angiosperm | Liliaceae | *Fritillaria acmopetala* | AY616709 |
| Angiosperm | Liliaceae | *Fritillaria affinis* | AY616710 |
| Angiosperm | Liliaceae | *Fritillaria agrestis* | AY616711 |
| Angiosperm | Liliaceae | *Fritillaria alburyana* | AY616712 |
| Angiosperm | Liliaceae | *Fritillaria aurea* | AY616713 |
| Angiosperm | Liliaceae | *Fritillaria camschatcensis* | AY616714 |
| Angiosperm | Liliaceae | *Fritillaria caucasica* | AY616715 |
| Angiosperm | Liliaceae | *Fritillaria chitralensis* | AY616716 |
| Angiosperm | Liliaceae | *Fritillaria crassifolia* | AY616717 |
| Angiosperm | Liliaceae | *Fritillaria davidii* | AY616718 |
| Angiosperm | Liliaceae | *Fritillaria eastwoodiae* | AY616719 |
| Angiosperm | Liliaceae | *Fritillaria falcata* | AY616720 |
| Angiosperm | Liliaceae | *Fritillaria gentneri* | AY616721 |
| Angiosperm | Liliaceae | *Fritillaria glauca* | AY616723 |
| Angiosperm | Liliaceae | *Fritillaria hermontis* | AY616724 |
| Angiosperm | Liliaceae | *Fritillaria imperialis* | AY616725 |
| Angiosperm | Liliaceae | *Fritillaria karelini* | AY616727 |
| Angiosperm | Liliaceae | *Fritillaria lusitanica* | AY616728 |
| Angiosperm | Liliaceae | *Fritillaria maximoviczii* | AY616729 |
| Angiosperm | Liliaceae | *Fritillaria meleagris* | AY616730 |
| Angiosperm | Liliaceae | *Fritillaria michailovskyi* | AY616731 |
| Angiosperm | Liliaceae | *Fritillaria micrantha* | AY616732 |
| Angiosperm | Liliaceae | *Fritillaria minuta* | AY616733 |
| Angiosperm | Liliaceae | *Fritillaria olivieri* | AY616734 |
| Angiosperm | Liliaceae | *Fritillaria pallidiflora* | AY616735 |
| Angiosperm | Liliaceae | *Fritillaria persica* | AY616736 |
| Angiosperm | Liliaceae | *Fritillaria phaeanthera* | AY616737 |
| Angiosperm | Liliaceae | *Fritillaria raddeana* | AY616739 |
| Angiosperm | Liliaceae | *Fritillaria recurva* | AY616740 |
| Angiosperm | Liliaceae | *Fritillaria reuteri* | AY616741 |
| Angiosperm | Liliaceae | *Fritillaria sewerzowii* | AY616742 |
| Angiosperm | Liliaceae | *Fritillaria striata* | AY616743 |
| Angiosperm | Liliaceae | *Fritillaria tenella* | AY616744 |
| Angiosperm | Liliaceae | *Fritillaria tubiformis* | AY616745 |
| Angiosperm | Liliaceae | *Fritillaria ussuriensis* | DQ191622 |
| Angiosperm | Liliaceae | *Gagea afghanica* | AM087953 |
| Angiosperm | Liliaceae | *Gagea altaica* | AM162670 |
| Angiosperm | Liliaceae | *Gagea artemczukii* | AM409331 |
| Angiosperm | Liliaceae | *Gagea bohemica* | AM265527 |
| Angiosperm | Liliaceae | *Gagea bohemica* | AM287271 |
| Angiosperm | Liliaceae | *Gagea bulbifera* | AM162669 |
| Angiosperm | Liliaceae | *Gagea caelestis* | AM180456 |
| Angiosperm | Liliaceae | *Gagea capillifolia* | AM087951 |
| Angiosperm | Liliaceae | *Gagea capusii* | AM422455 |
| Angiosperm | Liliaceae | *Gagea circumplexa* | AM265529 |
| Angiosperm | Liliaceae | *Gagea confusa* | AM087949 |
| Angiosperm | Liliaceae | *Gagea dschungarica* | AM087952 |
| Angiosperm | Liliaceae | *Gagea dubia* | AM422462 |
| Angiosperm | Liliaceae | *Gagea dubia* | AM903048 |
| Angiosperm | Liliaceae | *Gagea filiformis* | AM180457 |
| Angiosperm | Liliaceae | *Gagea foliosa* | AM409346 |
| Angiosperm | Liliaceae | *Gagea gageoides* | AM162673 |
| Angiosperm | Liliaceae | *Gagea granatellii* | AM409342 |
| Angiosperm | Liliaceae | *Gagea granulosa* | AM265533 |
| Angiosperm | Liliaceae | *Gagea granulosa* | AM287278 |
| Angiosperm | Liliaceae | *Gagea helenae* | AM265531 |
| Angiosperm | Liliaceae | *Gagea hiensis* | AM287279 |
| Angiosperm | Liliaceae | *Gagea lactea* | AM180452 |
| Angiosperm | Liliaceae | *Gagea minima* | AM087948 |
| Angiosperm | Liliaceae | *Gagea minima* | AM180459 |
| Angiosperm | Liliaceae | *Gagea minima* | AM287273 |
| Angiosperm | Liliaceae | *Gagea nakaiana* | AM180454 |
| Angiosperm | Liliaceae | *Gagea pauciflora* | AM493952 |
| Angiosperm | Liliaceae | *Gagea peduncularis* | AM903050 |
| Angiosperm | Liliaceae | *Gagea pratensis* | AM903047 |
| Angiosperm | Liliaceae | *Gagea pusilla* | AM422453 |
| Angiosperm | Liliaceae | *Gagea rubicunda* | AM493954 |
| Angiosperm | Liliaceae | *Gagea sarmentosa* | AM422458 |
| Angiosperm | Liliaceae | *Gagea shmakoviana* | AM422454 |
| Angiosperm | Liliaceae | *Gagea stipitata* | AM409336 |
| Angiosperm | Liliaceae | *Gagea tenera* | AM422460 |
| Angiosperm | Liliaceae | *Gagea terraccianoana* | AM493955 |
| Angiosperm | Liliaceae | *Gagea tisoniana* | AM422466 |
| Angiosperm | Liliaceae | *Gagea trinervia* | AM779093 |
| Angiosperm | Liliaceae | *Gagea ugamica* | AM422459 |
| Angiosperm | Liliaceae | *Gagea vegeta* | AM287275 |
| Angiosperm | Liliaceae | *Heterosmilax japonica* | AY775257 |
| Angiosperm | Liliaceae | *Lilium humboldtii* | AY616746 |
| Angiosperm | Liliaceae | *Lilium longiflorum* | AY684927 |
| Angiosperm | Liliaceae | *Lilium lophophorum* | AY616747 |
| Angiosperm | Liliaceae | *Lilium rubescens* | AY616749 |
| Angiosperm | Liliaceae | *Lilium sachalinense* | AY616750 |
| Angiosperm | Liliaceae | *Liriope muscari* | U24040 |
| Angiosperm | Liliaceae | *Smilax biltmoreana* | AY775252 |
| Angiosperm | Liliaceae | *Smilax davidiana* | AY748351 |
| Angiosperm | Liliaceae | *Smilax herbacea* | AY775247 |
| Angiosperm | Liliaceae | *Smilax herbacea* | AY775248 |
| Angiosperm | Liliaceae | *Smilax herbacea* | AY775249 |
| Angiosperm | Liliaceae | *Smilax herbacea* | AY775250 |
| Angiosperm | Liliaceae | *Smilax hugeri* | AY775254 |
| Angiosperm | Liliaceae | *Smilax lasioneura* | AY775251 |
| Angiosperm | Liliaceae | *Smilax nipponica* | AY775242 |
| Angiosperm | Liliaceae | *Smilax nipponica* | AY775246 |
| Angiosperm | Liliaceae | *Smilax pulverulenta* | AY775253 |
| Angiosperm | Liliaceae | *Smilax riparia* | AY775231 |
| Angiosperm | Liliaceae | *Smilax riparia* | AY775232 |
| Angiosperm | Liliaceae | *Smilax riparia* | AY775233 |
| Angiosperm | Liliaceae | *Smilax riparia* | AY775234 |
| Angiosperm | Liliaceae | *Smilax riparia* | AY775235 |
| Angiosperm | Liliaceae | *Smilax riparia* | AY775236 |
| Angiosperm | Liliaceae | *Smilax riparia* | AY775237 |
| Angiosperm | Liliaceae | *Smilax riparia* | AY775238 |
| Angiosperm | Liliaceae | *Smilax riparia* var. *acuminata* | AY775239 |
| Angiosperm | Liliaceae | *Smilax riparia* var. *acuminata* | AY775240 |
| Angiosperm | Liliaceae | *Smilax riparia* var. *acuminata* | AY775241 |
| Angiosperm | Liliaceae | *Smilax tsinchengshanensis* | AY775256 |
| Angiosperm | Liliaceae | *Tulipa clusiana* | AM180460 |
| Angiosperm | Liliaceae | *Tulipa cretica* | AM180461 |
| Angiosperm | Liliaceae | *Tulipa sprengeri* | AM162675 |
| Angiosperm | Liliaceae | *Veratrum album* | AF303707 |
| Angiosperm | Liliaceae | *Veratrum californicum* | AF303704 |
| Angiosperm | Liliaceae | *Veratrum fimbriatum* | AF303705 |
| Angiosperm | Liliaceae | *Veratrum maackii* | AF303708 |
| Angiosperm | Liliaceae | *Veratrum viride* | AF303706 |
| Angiosperm | Linaceae | *Linum monogynum* var. *chathamicum* | EU331100 |
| Angiosperm | Linaceae | *Linum monogynum* var. *chathamicum* | EU331101 |
| Angiosperm | Loganiaceae | *Buddleja marrubiifolia* | AF363671 |
| Angiosperm | Loganiaceae | *Buddleja polystachya* | AJ550577 |
| Angiosperm | Loganiaceae | *Buddleja saligna* | AJ550578 |
| Angiosperm | Loganiaceae | *Buddleja thyrsoides* | AJ550579 |
| Angiosperm | Loranthaceae | *Viscum album* | EU796892 |
| Angiosperm | Lowiaceae | *Orchidantha borneensis* | AF434877 |
| Angiosperm | Lowiaceae | *Orchidantha chinensis* | AF434878 |
| Angiosperm | Lowiaceae | *Orchidantha fimbriata* | AF434879 |
| Angiosperm | Lowiaceae | *Orchidantha grandiflora* | AF434880 |
| Angiosperm | Lowiaceae | *Orchidantha holttumii* | AF434881 |
| Angiosperm | Lowiaceae | *Orchidantha inouei* | AF434882 |
| Angiosperm | Lowiaceae | *Orchidantha longiflora* | AF434883 |
| Angiosperm | Lowiaceae | *Orchidantha maxillarioides* | AF434884 |
| Angiosperm | Lowiaceae | *Orchidantha sabahensis* | AF434886 |
| Angiosperm | Lowiaceae | *Orchidantha siamensis* | AF434887 |
| Angiosperm | Lythraceae | *Lythrum borysthenicum* | AY910747 |
| Angiosperm | Lythraceae | *Lythrum lineare* | AY910748 |
| Angiosperm | Magnoliaceae | *Illicium fargesii* | AF263449 |
| Angiosperm | Magnoliaceae | *Kadsura ananosma* | AF263447 |
| Angiosperm | Magnoliaceae | *Kadsura coccinea* | AF263445 |
| Angiosperm | Magnoliaceae | *Kadsura heteroclita* | AF263446 |
| Angiosperm | Magnoliaceae | *Kadsura japonica* | EF138798 |
| Angiosperm | Magnoliaceae | *Kadsura longipedunculata* | AF263448 |
| Angiosperm | Magnoliaceae | *Magnolia denudata* | AY858638 |
| Angiosperm | Magnoliaceae | *Michelia chapensis* | DQ234270 |
| Angiosperm | Magnoliaceae | *Michelia odora* | DQ674740 |
| Angiosperm | Magnoliaceae | *Schisandra bicolor* var. *tuberculata* | AF263442 |
| Angiosperm | Magnoliaceae | *Schisandra chinensis* | AF263441 |
| Angiosperm | Magnoliaceae | *Schisandra glabra* | DQ342254 |
| Angiosperm | Magnoliaceae | *Schisandra plena* | AF263443 |
| Angiosperm | Magnoliaceae | *Schisandra propinqua* var. *sinensis* | AF263444 |
| Angiosperm | Magnoliaceae | *Schisandra rubriflora* | AF263440 |
| Angiosperm | Magnoliaceae | *Schisandra sphenanthera* | AF263437 |
| Angiosperm | Magnoliaceae | *Schisandra viridis* | AF263438 |
| Angiosperm | Malpighiaceae | *Aspidopterys elliptica* | AF436780 |
| Angiosperm | Malvaceae | *Abutilon eremitopetalum* | EF219363 |
| Angiosperm | Malvaceae | *Abutilon eremitopetalum* | EF219364 |
| Angiosperm | Malvaceae | *Abutilon grandifolium* | EF219369 |
| Angiosperm | Malvaceae | *Abutilon incanum* | EF219368 |
| Angiosperm | Malvaceae | *Abutilon menziesii* | EF219365 |
| Angiosperm | Malvaceae | *Abutilon sandwicense* | EF219366 |
| Angiosperm | Malvaceae | *Abutilon sandwicense* | EF219367 |
| Angiosperm | Malvaceae | *Abutilon theophrasti* | DQ006017 |
| Angiosperm | Malvaceae | *Althaea cannabina* | EF419539 |
| Angiosperm | Malvaceae | *Althaea cannabina* | EF419540 |
| Angiosperm | Malvaceae | *Althaea cannabina* | EF419541 |
| Angiosperm | Malvaceae | *Althaea hirsuta* | EF419507 |
| Angiosperm | Malvaceae | *Althaea hirsuta* | EF419510 |
| Angiosperm | Malvaceae | *Althaea ludwigii* | EF419504 |
| Angiosperm | Malvaceae | *Althaea ludwigii* | EF419505 |
| Angiosperm | Malvaceae | *Hibiscus kokio* subsp. *kokio* | AY962407 |
| Angiosperm | Malvaceae | *Malva aegyptia* | EF419516 |
| Angiosperm | Malvaceae | *Malva aegyptia* | EF419517 |
| Angiosperm | Malvaceae | *Malva aegyptia* | EF419518 |
| Angiosperm | Malvaceae | *Malva assurgentiflora* | EF419460 |
| Angiosperm | Malvaceae | *Malva australiana* | EF419462 |
| Angiosperm | Malvaceae | *Malva canariensis* | EF419459 |
| Angiosperm | Malvaceae | *Malva cretica* subsp. *althaeoides* | EF419513 |
| Angiosperm | Malvaceae | *Malva cretica* subsp. *althaeoides* | EF419515 |
| Angiosperm | Malvaceae | *Malva dendromorpha* | EF419468 |
| Angiosperm | Malvaceae | *Malva dendromorpha* | EF419469 |
| Angiosperm | Malvaceae | *Malva linnaei* | EF419470 |
| Angiosperm | Malvaceae | *Malva linnaei* | EF419471 |
| Angiosperm | Malvaceae | *Malva neglecta* | EF419478 |
| Angiosperm | Malvaceae | *Malva neglecta* | EF419479 |
| Angiosperm | Malvaceae | *Malva parviflora* | EF419483 |
| Angiosperm | Malvaceae | *Malva parviflora* | EF419484 |
| Angiosperm | Malvaceae | *Malva tournefortiana* | EF419497 |
| Angiosperm | Malvaceae | *Malva tournefortiana* | EF419498 |
| Angiosperm | Malvaceae | *Malva tournefortiana* | EF419499 |
| Angiosperm | Malvaceae | *Malva trifida* | EF419521 |
| Angiosperm | Malvaceae | *Malva trifida* | EF419522 |
| Angiosperm | Malvaceae | *Malva verticillata* | EF419487 |
| Angiosperm | Malvaceae | *Malva wigandii* | EF419456 |
| Angiosperm | Malvaceae | *Malva wigandii* | EF419457 |
| Angiosperm | Meliaceae | *Melia azedarach* | AY695595 |
| Angiosperm | Menispermaceae | *Cissampelos pareira* | EF143843 |
| Angiosperm | Menispermaceae | *Cocculus orbiculatus* | AY864900 |
| Angiosperm | Menispermaceae | *Cocculus orbiculatus* | EU079374 |
| Angiosperm | Menispermaceae | *Menispermum canadense* | L77163 |
| Angiosperm | Menispermaceae | *Menispermum dauricum* | L77161 |
| Angiosperm | Menispermaceae | *Sinomenium acutum* | AY017394 |
| Angiosperm | Menispermaceae | *Stephania tetrandra* | EU808017 |
| Angiosperm | Menispermaceae | *Tinospora capillipes* | AY017385 |
| Angiosperm | Menispermaceae | *Tinospora sinensis* | AY017386 |
| Angiosperm | Molluginaceae | *Mollugo stricta* | EF523566 |
| Angiosperm | Molluginaceae | *Mollugo verticillata* | DQ006020 |
| Angiosperm | Moraceae | *Morus alba* | AM041998 |
| Angiosperm | Moraceae | *Morus alba* | AY345148 |
| Angiosperm | Moraceae | *Morus alba* | AY345149 |
| Angiosperm | Moraceae | *Morus alba* var. *multicaulis* | AM042003 |
| Angiosperm | Moraceae | *Morus lhou* | AM041999 |
| Angiosperm | Moraceae | *Morus macroura* | AY345147 |
| Angiosperm | Moraceae | *Morus nigra* | AM042002 |
| Angiosperm | Moraceae | *Morus rotundiloba* | AY345150 |
| Angiosperm | Moraceae | *Morus wittiorum* | AY345154 |
| Angiosperm | Moraceae | *Morus wittiorum* | AY345155 |
| Angiosperm | Moraceae | *Streblus banksii* | EF635452 |
| Angiosperm | Moraceae | *Streblus smithii* | EF635447 |
| Angiosperm | Musaceae | *Musa acuminata* subsp. *burmannicoides* | EU418630 |
| Angiosperm | Musaceae | *Musa beccarii* | AF434900 |
| Angiosperm | Myricaceae | *Myrica gale* | AJ626768 |
| Angiosperm | Myricaceae | *Myrica gale* | AJ626769 |
| Angiosperm | Myrsinaceae | *Ardisia crenata* | AF547730 |
| Angiosperm | Myrtaceae | *Eucalyptus camaldulensis* | AF190363 |
| Angiosperm | Myrtaceae | *Eucalyptus cloeziana* | AF190366 |
| Angiosperm | Myrtaceae | *Eucalyptus deglupta* | AF190362 |
| Angiosperm | Myrtaceae | *Eucalyptus erythrocorys* | AF190365 |
| Angiosperm | Myrtaceae | *Eucalyptus globulus* subsp. *bicostata* | U62665 |
| Angiosperm | Myrtaceae | *Eucalyptus pilularis* | AF190367 |
| Angiosperm | Myrtaceae | *Eucalyptus tereticornis* | AY864901 |
| Angiosperm | Myrtaceae | *Eucalyptus tetragona* | AF190364 |
| Angiosperm | Myrtaceae | *Eugenia albanensis* | AY487286 |
| Angiosperm | Myrtaceae | *Eugenia axillaris* | AY487300 |
| Angiosperm | Myrtaceae | *Eugenia capensis* | AY487292 |
| Angiosperm | Myrtaceae | *Eugenia cerasiflora* | AY487296 |
| Angiosperm | Myrtaceae | *Eugenia crassipetala* | AY487288 |
| Angiosperm | Myrtaceae | *Eugenia erythrophylla* | AY463139 |
| Angiosperm | Myrtaceae | *Eugenia florida* | AM234090 |
| Angiosperm | Myrtaceae | *Eugenia foetida* | AY487298 |
| Angiosperm | Myrtaceae | *Eugenia greggii* | AY487285 |
| Angiosperm | Myrtaceae | *Eugenia involucrata* | AY487294 |
| Angiosperm | Myrtaceae | *Eugenia langsdorffii* | AM234092 |
| Angiosperm | Myrtaceae | *Eugenia latifolia* | AM234091 |
| Angiosperm | Myrtaceae | *Eugenia lucida* | AY487289 |
| Angiosperm | Myrtaceae | *Eugenia microphylla* | AY487295 |
| Angiosperm | Myrtaceae | *Eugenia orbiculata* | AY487290 |
| Angiosperm | Myrtaceae | *Eugenia orbiculata* | AY487291 |
| Angiosperm | Myrtaceae | *Eugenia punicifolia* | AM234087 |
| Angiosperm | Myrtaceae | *Eugenia reinwardtiana* | AY487301 |
| Angiosperm | Myrtaceae | *Eugenia stictosepala* | AM234086 |
| Angiosperm | Myrtaceae | *Eugenia sulcata* | AM234089 |
| Angiosperm | Myrtaceae | *Eugenia tinifolia* | AY487287 |
| Angiosperm | Myrtaceae | *Eugenia tropophylla* | AY487303 |
| Angiosperm | Myrtaceae | *Eugenia umtamvunensis* | AY463142 |
| Angiosperm | Myrtaceae | *Eugenia uniflora* | AM234088 |
| Angiosperm | Myrtaceae | *Eugenia uniflora* | AY487284 |
| Angiosperm | Myrtaceae | *Eugenia verdoorniae* | AY463137 |
| Angiosperm | Myrtaceae | *Eugenia woodii* | AY463138 |
| Angiosperm | Myrtaceae | *Eugenia zeyheri* | AY463136 |
| Angiosperm | Myrtaceae | *Eugenia zuluensis* | AY487293 |
| Angiosperm | Nelumbonaceae | *Nelumbo lutea* | AY620419 |
| Angiosperm | Nelumbonaceae | *Nelumbo lutea* | AY858639 |
| Angiosperm | Nelumbonaceae | *Nelumbo nucifera* | AY858640 |
| Angiosperm | Nelumbonaceae | *Nelumbo nucifera* | DQ105981 |
| Angiosperm | Nelumbonaceae | *Nelumbo nucifera* | DQ105982 |
| Angiosperm | Nelumbonaceae | *Nelumbo nucifera* | DQ105984 |
| Angiosperm | Nelumbonaceae | *Nelumbo nucifera* | DQ901015 |
| Angiosperm | Nymphaeaceae | *Euryale ferox* | AY858637 |
| Angiosperm | Olacaceae | *Schoepfia schreberi* | DQ788706 |
| Angiosperm | Oleaceae | *Olea europaea* subsp. *europaea* | AJ585193 |
| Angiosperm | Oleaceae | *Forsythia giraldiana* | AF534809 |
| Angiosperm | Oleaceae | *Forsythia ovata* | AF534812 |
| Angiosperm | Oleaceae | *Forsythia suspensa* | AF534808 |
| Angiosperm | Oleaceae | *Forsythia viridissima* | AF534810 |
| Angiosperm | Oleaceae | *Forsythia viridissima* var. *koreana* | AF534806 |
| Angiosperm | Oleaceae | *Fraxinus americana* | U82909 |
| Angiosperm | Oleaceae | *Fraxinus angustifolia* | EU314820 |
| Angiosperm | Oleaceae | *Fraxinus angustifolia* subsp. *oxycarpa* | U82869 |
| Angiosperm | Oleaceae | *Fraxinus angustifolia* subsp. *syriaca* | U82873 |
| Angiosperm | Oleaceae | *Fraxinus anomala* | U82915 |
| Angiosperm | Oleaceae | *Fraxinus biltmoreana* | U82911 |
| Angiosperm | Oleaceae | *Fraxinus chiisanensis* | EU314833 |
| Angiosperm | Oleaceae | *Fraxinus chinensis* | U82885 |
| Angiosperm | Oleaceae | *Fraxinus chinensis* subsp. *rhynchophylla* | U82887 |
| Angiosperm | Oleaceae | *Fraxinus cuspidata* | U82917 |
| Angiosperm | Oleaceae | *Fraxinus dipetala* | EU314842 |
| Angiosperm | Oleaceae | *Fraxinus dubia* | EU314846 |
| Angiosperm | Oleaceae | *Fraxinus excelsior* | U82867 |
| Angiosperm | Oleaceae | *Fraxinus gooddingii* | EU314852 |
| Angiosperm | Oleaceae | *Fraxinus greggii* | EU314855 |
| Angiosperm | Oleaceae | *Fraxinus lanuginosa* | EU314857 |
| Angiosperm | Oleaceae | *Fraxinus latifolia* | U82913 |
| Angiosperm | Oleaceae | *Fraxinus longicuspis* | U82891 |
| Angiosperm | Oleaceae | *Fraxinus mandshurica* | U82875 |
| Angiosperm | Oleaceae | *Fraxinus micrantha* | EU314866 |
| Angiosperm | Oleaceae | *Fraxinus nigra* | U82879 |
| Angiosperm | Oleaceae | *Fraxinus ornus* | U82893 |
| Angiosperm | Oleaceae | *Fraxinus pallisae* | U82871 |
| Angiosperm | Oleaceae | *Fraxinus papillosa* | EU314872 |
| Angiosperm | Oleaceae | *Fraxinus paxiana* | EU314874 |
| Angiosperm | Oleaceae | *Fraxinus pennsylvanica* | U82903 |
| Angiosperm | Oleaceae | *Fraxinus platypoda* | U82877 |
| Angiosperm | Oleaceae | *Fraxinus purpusii* | EU314880 |
| Angiosperm | Oleaceae | *Fraxinus quadrangulata* | U82883 |
| Angiosperm | Oleaceae | *Fraxinus raibocarpa* | EU314884 |
| Angiosperm | Oleaceae | *Fraxinus rufescens* | EU314885 |
| Angiosperm | Oleaceae | *Fraxinus sieboldiana* | EU314886 |
| Angiosperm | Oleaceae | *Fraxinus spaethiana* | EU314890 |
| Angiosperm | Oleaceae | *Fraxinus texensis* | EU314892 |
| Angiosperm | Oleaceae | *Fraxinus tomentosa* | U82899 |
| Angiosperm | Oleaceae | *Fraxinus uhdei* | EU314896 |
| Angiosperm | Oleaceae | *Fraxinus xanthoxyloides* | EU314900 |
| Angiosperm | Oleaceae | *Ligustrum acutissimum* | AF361295 |
| Angiosperm | Oleaceae | *Ligustrum compactum* | AF361292 |
| Angiosperm | Oleaceae | *Ligustrum japonicum* | AF361299 |
| Angiosperm | Oleaceae | *Ligustrum obtusifolium* | AF361294 |
| Angiosperm | Oleaceae | *Ligustrum ovalifolium* | AF361296 |
| Angiosperm | Oleaceae | *Ligustrum sempervirens* | AF361300 |
| Angiosperm | Oleaceae | *Ligustrum vulgare* | EU314901 |
| Angiosperm | Oleaceae | *Syringa amurensis* | AF297072 |
| Angiosperm | Oleaceae | *Syringa amurensis* | AF297074 |
| Angiosperm | Oleaceae | *Syringa patula* | AF277755 |
| Angiosperm | Oleaceae | *Syringa patula* | AF277756 |
| Angiosperm | Oleaceae | *Syringa patula* | AF277757 |
| Angiosperm | Oleaceae | *Syringa patula* | AF277758 |
| Angiosperm | Oleaceae | *Syringa patula* | AF277759 |
| Angiosperm | Oleaceae | *Syringa pinnatifolia* | AF297081 |
| Angiosperm | Oleaceae | *Syringa pubescens* | AF277745 |
| Angiosperm | Oleaceae | *Syringa pubescens* | AF277746 |
| Angiosperm | Oleaceae | *Syringa pubescens* | AF277747 |
| Angiosperm | Oleaceae | *Syringa reticulata* | AF297078 |
| Angiosperm | Oleaceae | *Syringa reticulata* | AF297079 |
| Angiosperm | Oleaceae | *Syringa reticulata* subsp. *pekinensis* | AF297075 |
| Angiosperm | Oleaceae | *Syringa reticulata* subsp. *pekinensis* | AF297076 |
| Angiosperm | Oleaceae | *Syringa villosa* | AF277760 |
| Angiosperm | Oleaceae | *Syringa vulgaris* | DQ184479 |
| Angiosperm | Onagraceae | *Oenothera albicaulis* | DQ075612 |
| Angiosperm | Onagraceae | *Oenothera biennis* | DQ006021 |
| Angiosperm | Onagraceae | *Oenothera coronopifolia* | DQ075617 |
| Angiosperm | Onagraceae | *Oenothera deltoides* subsp. *cognata* | DQ075619 |
| Angiosperm | Onagraceae | *Oenothera deltoides* subsp. *deltoides* | DQ075620 |
| Angiosperm | Onagraceae | *Oenothera deltoides* subsp. *piperi* | DQ075622 |
| Angiosperm | Onagraceae | *Oenothera drummondii* | AJ620537 |
| Angiosperm | Onagraceae | *Oenothera engelmannii* | DQ075623 |
| Angiosperm | Onagraceae | *Oenothera flava* subsp. *taraxacoides* | DQ075624 |
| Angiosperm | Onagraceae | *Oenothera laciniata* | AJ620539 |
| Angiosperm | Onagraceae | *Oenothera macrocarpa* | AJ620540 |
| Angiosperm | Onagraceae | *Oenothera magellanica* | DQ075627 |
| Angiosperm | Onagraceae | *Oenothera nuttallii* | DQ075629 |
| Angiosperm | Onagraceae | *Oenothera organensis* | DQ075630 |
| Angiosperm | Onagraceae | *Oenothera pallida* subsp. *gypsophila* | DQ075631 |
| Angiosperm | Onagraceae | *Oenothera pallida* subsp. *pallida* | DQ075632 |
| Angiosperm | Onagraceae | *Oenothera primiveris* | DQ075635 |
| Angiosperm | Onagraceae | *Oenothera speciosa* | AJ620541 |
| Angiosperm | Onagraceae | *Oenothera speciosa* | DQ075636 |
| Angiosperm | Onagraceae | *Oenothera tetraptera* | DQ075638 |
| Angiosperm | Onagraceae | *Oenothera tubifera* | DQ075639 |
| Angiosperm | Onagraceae | *Oenothera wigginsii* | DQ075640 |
| Angiosperm | Onagraceae | *Oenothera xylocarpa* | DQ075641 |
| Angiosperm | Orchidaceae | *Bletilla formosana* | EU100761 |
| Angiosperm | Orchidaceae | *Bletilla striata* | EU100762 |
| Angiosperm | Orchidaceae | *Bulbophyllum burfordiense* | AY273716 |
| Angiosperm | Orchidaceae | *Cremastra appendiculata* | EF525673 |
| Angiosperm | Orchidaceae | *Cremastra appendiculata* | EU391320 |
| Angiosperm | Orchidaceae | *Cremastra appendiculata* var. *variabilis* | EU266414 |
| Angiosperm | Orchidaceae | *Cremastra unguiculata* | EF525674 |
| Angiosperm | Orchidaceae | *Cremastra unguiculata* | EU266415 |
| Angiosperm | orchidaceae | *Cymbidium ensifolium* | AF470512 |
| Angiosperm | orchidaceae | *Cymbidium erythraeum* | AF470502 |
| Angiosperm | orchidaceae | *Cymbidium floribundum* | AF470506 |
| Angiosperm | orchidaceae | *Cymbidium madidum* | AF470507 |
| Angiosperm | orchidaceae | *Cymbidium sinense* | AF470517 |
| Angiosperm | orchidaceae | *Cymbidium suavissimum* | AF470505 |
| Angiosperm | orchidaceae | *Cypripedium calceolus* | AY557232 |
| Angiosperm | Orchidaceae | *Dendrobium adae* | EU430371 |
| Angiosperm | Orchidaceae | *Dendrobium aduncum* | AF314125 |
| Angiosperm | Orchidaceae | *Dendrobium aemulum* | EU430372 |
| Angiosperm | Orchidaceae | *Dendrobium bifalce* | EU430373 |
| Angiosperm | Orchidaceae | *Dendrobium callitrophilum* | EU430374 |
| Angiosperm | Orchidaceae | *Dendrobium carrii* | EU430376 |
| Angiosperm | Orchidaceae | *Dendrobium chrysanthum* | AF314126 |
| Angiosperm | Orchidaceae | *Dendrobium chrysotoxum* | AF314127 |
| Angiosperm | Orchidaceae | *Dendrobium crepidatum* | AF314128 |
| Angiosperm | Orchidaceae | *Dendrobium crumenatum* | AY273708 |
| Angiosperm | Orchidaceae | *Dendrobium densiflorum* | AF314129 |
| Angiosperm | Orchidaceae | *Dendrobium fimbriatum* | AF314130 |
| Angiosperm | Orchidaceae | *Dendrobium gracilicaule* | EU430382 |
| Angiosperm | Orchidaceae | *Dendrobium hercoglossum* | AF314131 |
| Angiosperm | Orchidaceae | *Dendrobium jenkinsii* | AF314132 |
| Angiosperm | Orchidaceae | *Dendrobium jonesii* var. *magnificum* | EU430383 |
| Angiosperm | Orchidaceae | *Dendrobium kingianum* subsp. *carnarvonense* | EU430384 |
| Angiosperm | Orchidaceae | *Dendrobium kingianum* var. *pulcherrimum* | EU430385 |
| Angiosperm | Orchidaceae | *Dendrobium lindleyi* | AF314133 |
| Angiosperm | Orchidaceae | *Dendrobium loddigesii* | AF314134 |
| Angiosperm | Orchidaceae | *Dendrobium lohohense* | AF314135 |
| Angiosperm | Orchidaceae | *Dendrobium moniliforme* | AF314136 |
| Angiosperm | Orchidaceae | *Dendrobium monophyllum* | EU430387 |
| Angiosperm | Orchidaceae | *Dendrobium moschatum* | AF314137 |
| Angiosperm | Orchidaceae | *Dendrobium nobile* | AF314138 |
| Angiosperm | Orchidaceae | *Dendrobium officinale* | AF314139 |
| Angiosperm | Orchidaceae | *Dendrobium racemosum* | EU430389 |
| Angiosperm | Orchidaceae | *Dendrobium speciosum* var. *curvicaule* | EU430395 |
| Angiosperm | Orchidaceae | *Dendrobium speciosum* var. *pedunculatum* | EU430398 |
| Angiosperm | Orchidaceae | *Dendrobium speciosum* var. *speciosum* | EU430399 |
| Angiosperm | Orchidaceae | *Dendrobium tetragonum* var. *cacatua* | EU430400 |
| Angiosperm | Orchidaceae | *Dendrobium tetragonum* var. *giganteum* | EU430401 |
| Angiosperm | Orchidaceae | *Dendrobium tetragonum* var. *melaleucaphilum* | EU430402 |
| Angiosperm | Orchidaceae | *Dendrobium tetragonum* var. *tetragonum* | EU430403 |
| Angiosperm | Orchidaceae | *Dendrobium williamsonii* | AF314140 |
| Angiosperm | Orchidaceae | *Epipactis albensis* | AY154384 |
| Angiosperm | Orchidaceae | *Epipactis palustris* | AY146448 |
| Angiosperm | Orchidaceae | *Gastrodia elata* | EU135903 |
| Angiosperm | Orchidaceae | *Gastrodia elata* | EU135904 |
| Angiosperm | Orchidaceae | *Gastrodia elata* | EU135910 |
| Angiosperm | Orchidaceae | *Gastrodia elata* | EU135912 |
| Angiosperm | Orchidaceae | *Goodyera macrantha* | AF366894 |
| Angiosperm | Orchidaceae | *Goodyera maximowicziana* | AF366895 |
| Angiosperm | Orchidaceae | *Goodyera pubescens* | AJ539519 |
| Angiosperm | Orchidaceae | *Goodyera repens* | AF366896 |
| Angiosperm | Orchidaceae | *Goodyera schlechtendaliana* | AF366897 |
| Angiosperm | Orchidaceae | *Goodyera velutina* | AF366898 |
| Angiosperm | Orchidaceae | *Liparis auriculata* | AB289458 |
| Angiosperm | Orchidaceae | *Liparis cordifolia* | AB289459 |
| Angiosperm | Orchidaceae | *Liparis liliifolia* | AB289475 |
| Angiosperm | Orchidaceae | *Liparis maingayi* | AB289476 |
| Angiosperm | Orchidaceae | *Liparis makinoana* | AB289474 |
| Angiosperm | Orchidaceae | *Liparis montana* | AB289477 |
| Angiosperm | Orchidaceae | *Liparis nervosa* | AB289482 |
| Angiosperm | Orchidaceae | *Liparis purpureoviridis* | AB289478 |
| Angiosperm | Orchidaceae | *Liparis truncata* | AB289479 |
| Angiosperm | orchidaceae | *Paphiopedilum adductum* | AY643459 |
| Angiosperm | orchidaceae | *Paphiopedilum argus* | AJ564363 |
| Angiosperm | orchidaceae | *Paphiopedilum barbigerum* | AY643442 |
| Angiosperm | orchidaceae | *Paphiopedilum callosum* | AJ564365 |
| Angiosperm | orchidaceae | *Paphiopedilum chamberlainianum* | AJ564360 |
| Angiosperm | orchidaceae | *Paphiopedilum concolor* | AJ564366 |
| Angiosperm | orchidaceae | *Paphiopedilum 'Dark Roller' x Paphiopedilum rothschildianum* | AJ564354 |
| Angiosperm | orchidaceae | *Paphiopedilum delenatii x Paphiopedilum emersonii* | AJ564372 |
| Angiosperm | orchidaceae | *Paphiopedilum emersonii* | AJ564355 |
| Angiosperm | orchidaceae | *Paphiopedilum fairrieanum* | AJ564367 |
| Angiosperm | orchidaceae | *Paphiopedilum glaucophyllum* | AY643437 |
| Angiosperm | orchidaceae | *Paphiopedilum haynaldianum* | AF324175 |
| Angiosperm | orchidaceae | *Paphiopedilum hennisianum* | AY643460 |
| Angiosperm | orchidaceae | *Paphiopedilum henryanum* | AY643445 |
| Angiosperm | orchidaceae | *Paphiopedilum hirsutissimum* | AJ564368 |
| Angiosperm | orchidaceae | *Paphiopedilum hirsutissimum* | AY643447 |
| Angiosperm | orchidaceae | *Paphiopedilum hybrid* | AY643444 |
| Angiosperm | orchidaceae | *Paphiopedilum hybrid* | AY643471 |
| Angiosperm | orchidaceae | *Paphiopedilum javanicum* | AY643461 |
| Angiosperm | orchidaceae | *Paphiopedilum kolopakingii* | AJ564359 |
| Angiosperm | orchidaceae | *Paphiopedilum lowii* | AY643456 |
| Angiosperm | orchidaceae | *Paphiopedilum malipoense* | AJ564357 |
| Angiosperm | orchidaceae | *Paphiopedilum mastersianum* | AY643466 |
| Angiosperm | orchidaceae | *Paphiopedilum micranthum* | AY643432 |
| Angiosperm | orchidaceae | *Paphiopedilum niveum* | AY643436 |
| Angiosperm | orchidaceae | *Paphiopedilum philippinense x Paphiopedilum stonei* | AJ564356 |
| Angiosperm | orchidaceae | *Paphiopedilum primulinum* var. *purpurascens* | AY643439 |
| Angiosperm | orchidaceae | *Paphiopedilum purpuratum* | AJ564364 |
| Angiosperm | orchidaceae | *Paphiopedilum randsii* | AY643458 |
| Angiosperm | orchidaceae | *Paphiopedilum richardianum* | AY643457 |
| Angiosperm | orchidaceae | *Paphiopedilum richardianum* | AY643474 |
| Angiosperm | orchidaceae | *Paphiopedilum rothschildianum* | AJ564370 |
| Angiosperm | orchidaceae | *Paphiopedilum rothschildianum* | EF459731 |
| Angiosperm | orchidaceae | *Paphiopedilum sanderianum* | AJ564374 |
| Angiosperm | orchidaceae | *Paphiopedilum schoseri* | AY643462 |
| Angiosperm | orchidaceae | *Paphiopedilum stonei* | AJ564361 |
| Angiosperm | orchidaceae | *Paphiopedilum sukhakulii* | AJ564362 |
| Angiosperm | orchidaceae | *Paphiopedilum sukhakulii* | AY643468 |
| Angiosperm | orchidaceae | *Paphiopedilum supardii* | AY643454 |
| Angiosperm | orchidaceae | *Paphiopedilum victoria-mariae* | AY643440 |
| Angiosperm | orchidaceae | *Paphiopedilum victoria-regina* | AY643441 |
| Angiosperm | orchidaceae | *Paphiopedilum wilhelminiae* | AJ564371 |
| Angiosperm | orchidaceae | *Paphiopedilum wilhelminiae* | AJ564373 |
| Angiosperm | orchidaceae | *Paphiopedilum wilhelminiae* | AY643455 |
| Angiosperm | Orchidaceae | *Platanthera praeclara* | AF301445 |
| Angiosperm | Orchidaceae | *Pleione albiflora* | AY101967 |
| Angiosperm | Orchidaceae | *Pleione bulbocodioides* | AF302739 |
| Angiosperm | Orchidaceae | *Pleione bulbocodioides* | AF461482 |
| Angiosperm | Orchidaceae | *Pleione bulbocodioides* | AF461483 |
| Angiosperm | Orchidaceae | *Pleione formosana* | AF302740 |
| Angiosperm | Orchidaceae | *Pleione formosana* | AF461484 |
| Angiosperm | Orchidaceae | *Pleione formosana* | AF461485 |
| Angiosperm | Orchidaceae | *Pleione formosana* | EU100746 |
| Angiosperm | Orchidaceae | *Pleione formosana* | EU100747 |
| Angiosperm | Orchidaceae | *Pleione formosana* | EU100748 |
| Angiosperm | Orchidaceae | *Pleione formosana* | EU100749 |
| Angiosperm | Orchidaceae | *Pleione formosana* | EU100750 |
| Angiosperm | Orchidaceae | *Pleione formosana* | EU100751 |
| Angiosperm | Orchidaceae | *Pleione formosana* | EU100752 |
| Angiosperm | Orchidaceae | *Pleione formosana* | EU100753 |
| Angiosperm | Orchidaceae | *Pleione formosana* | EU100754 |
| Angiosperm | Orchidaceae | *Pleione formosana* | EU100755 |
| Angiosperm | Orchidaceae | *Pleione formosana* | EU100756 |
| Angiosperm | Orchidaceae | *Pleione formosana* | EU100757 |
| Angiosperm | Orchidaceae | *Pleione formosana* | EU100758 |
| Angiosperm | Orchidaceae | *Pleione formosana* | EU100759 |
| Angiosperm | Orchidaceae | *Pleione formosana* | EU100760 |
| Angiosperm | Orchidaceae | *Pleione forrestii* | AF461478 |
| Angiosperm | Orchidaceae | *Pleione grandiflora* | AF461473 |
| Angiosperm | Orchidaceae | *Pleione grandiflora* | AF461474 |
| Angiosperm | Orchidaceae | *Pleione grandiflora* | AF461475 |
| Angiosperm | Orchidaceae | *Pleione grandiflora* | AF461476 |
| Angiosperm | Orchidaceae | *Pleione grandiflora* | AF461477 |
| Angiosperm | Orchidaceae | *Pleione hookeriana* | AF461468 |
| Angiosperm | Orchidaceae | *Pleione hookeriana* | AF461469 |
| Angiosperm | Orchidaceae | *Pleione humilis* | AF461495 |
| Angiosperm | Orchidaceae | *Pleione limprichtii* | AF461489 |
| Angiosperm | Orchidaceae | *Pleione limprichtii* | AF461490 |
| Angiosperm | Orchidaceae | *Pleione maculata* | AF461493 |
| Angiosperm | Orchidaceae | *Pleione pleionoides* | AF461480 |
| Angiosperm | Orchidaceae | *Pleione praecox* | AF461491 |
| Angiosperm | Orchidaceae | *Pleione saxicola* | AF461492 |
| Angiosperm | Orchidaceae | *Pleione scopulorum* | AF461471 |
| Angiosperm | Orchidaceae | *Vanda lilacina* | EF079437 |
| Angiosperm | Orchidaceae | *Vanda luzonica* | AY278111 |
| Angiosperm | Orchidaceae | *Vanda pumila* | EF670372 |
| Angiosperm | Orchidaceae | *Vanda tricolor* | EF670373 |
| Angiosperm | Orchidaceae | *Vanda tricolor* | EF670374 |
| Angiosperm | Orchidaceae | *Vanda tricolor* | EF670375 |
| Angiosperm | Orobanchaceae | *Cistanche deserticola* | AB217873 |
| Angiosperm | Orobanchaceae | *Cistanche phelypaea* subsp. *phelypaea* | AY209303 |
| Angiosperm | Orobanchaceae | *Cistanche salsa* | AB217872 |
| Angiosperm | Orobanchaceae | *Cistanche sinensis* | AB217874 |
| Angiosperm | Orobanchaceae | *Cistanche tubulosa* | AY209299 |
| Angiosperm | Orobanchaceae | *Cistanche violacea* | AY209305 |
| Angiosperm | Orobanchaceae | *Orobanche coerulescens* | AY881142 |
| Angiosperm | Oxalidaceae | *Averrhoa bilimbi* | EU436862 |
| Angiosperm | Oxalidaceae | *Averrhoa carambola* | EU436863 |
| Angiosperm | Oxalidaceae | *Oxalis acetosella* | EU436870 |
| Angiosperm | Oxalidaceae | *Oxalis adenodes* | EU436903 |
| Angiosperm | Oxalidaceae | *Oxalis adspersa* | EU436936 |
| Angiosperm | Oxalidaceae | *Oxalis ambigua* | EU436905 |
| Angiosperm | Oxalidaceae | *Oxalis amblyodonta* | EU437007 |
| Angiosperm | Oxalidaceae | *Oxalis amblyosepala* | EU436982 |
| Angiosperm | Oxalidaceae | *Oxalis andina* | U74263 |
| Angiosperm | Oxalidaceae | *Oxalis annae* | EU436920 |
| Angiosperm | Oxalidaceae | *Oxalis argillacea* | EU436940 |
| Angiosperm | Oxalidaceae | *Oxalis argyrophylla* | EU437005 |
| Angiosperm | Oxalidaceae | *Oxalis aridicola* | EU436977 |
| Angiosperm | Oxalidaceae | *Oxalis attaquana* | EU436918 |
| Angiosperm | Oxalidaceae | *Oxalis aurea* | EU436935 |
| Angiosperm | Oxalidaceae | *Oxalis barrelieri* | EU436866 |
| Angiosperm | Oxalidaceae | *Oxalis bifida* | EU437017 |
| Angiosperm | Oxalidaceae | *Oxalis bifurca* | EU436965 |
| Angiosperm | Oxalidaceae | *Oxalis boliviana* | U74264 |
| Angiosperm | Oxalidaceae | *Oxalis bowiei* | EU436895 |
| Angiosperm | Oxalidaceae | *Oxalis brasiliensis* | EU436879 |
| Angiosperm | Oxalidaceae | *Oxalis bullulata* | EU436908 |
| Angiosperm | Oxalidaceae | *Oxalis burkei* | EU437029 |
| Angiosperm | Oxalidaceae | *Oxalis burtoniae* | EU437008 |
| Angiosperm | Oxalidaceae | *Oxalis callosa* | EU436973 |
| Angiosperm | Oxalidaceae | *Oxalis camelopardalis* | EU437012 |
| Angiosperm | Oxalidaceae | *Oxalis campicola* | EU436981 |
| Angiosperm | Oxalidaceae | *Oxalis campylorrhiza* | EU436947 |
| Angiosperm | Oxalidaceae | *Oxalis capillacea* | EU437013 |
| Angiosperm | Oxalidaceae | *Oxalis caprina* | EU436892 |
| Angiosperm | Oxalidaceae | *Oxalis cathara* | EU436931 |
| Angiosperm | Oxalidaceae | *Oxalis ciliaris* | EU436979 |
| Angiosperm | Oxalidaceae | *Oxalis clavifolia* | EU436951 |
| Angiosperm | Oxalidaceae | *Oxalis commutata* | EU436898 |
| Angiosperm | Oxalidaceae | *Oxalis comosa* | EU436954 |
| Angiosperm | Oxalidaceae | *Oxalis comptonii* | EU436985 |
| Angiosperm | Oxalidaceae | *Oxalis confertifolia* | EU437011 |
| Angiosperm | Oxalidaceae | *Oxalis convexula* | EU436913 |
| Angiosperm | Oxalidaceae | *Oxalis copiosa* | EU436887 |
| Angiosperm | Oxalidaceae | *Oxalis corniculata* | EU274617 |
| Angiosperm | Oxalidaceae | *Oxalis corniculata* | EU274619 |
| Angiosperm | Oxalidaceae | *Oxalis corniculata* | EU436867 |
| Angiosperm | Oxalidaceae | *Oxalis crocea* | EU436950 |
| Angiosperm | Oxalidaceae | *Oxalis densa* | EU436921 |
| Angiosperm | Oxalidaceae | *Oxalis dentata* | EU436888 |
| Angiosperm | Oxalidaceae | *Oxalis depressa* | EU436909 |
| Angiosperm | Oxalidaceae | *Oxalis dichotoma* | EU436893 |
| Angiosperm | Oxalidaceae | *Oxalis dilatata* | EU436910 |
| Angiosperm | Oxalidaceae | *Oxalis dillenii* | EU436868 |
| Angiosperm | Oxalidaceae | *Oxalis dines* | EU436943 |
| Angiosperm | Oxalidaceae | *Oxalis disticha* | EU436942 |
| Angiosperm | Oxalidaceae | *Oxalis dregei* | EU436930 |
| Angiosperm | Oxalidaceae | *Oxalis droseroides* | EU436994 |
| Angiosperm | Oxalidaceae | *Oxalis duriuscula* | EU437020 |
| Angiosperm | Oxalidaceae | *Oxalis ebracteata* | EU436990 |
| Angiosperm | Oxalidaceae | *Oxalis eckloniana* | EU437025 |
| Angiosperm | Oxalidaceae | *Oxalis engleriana* | EU436961 |
| Angiosperm | Oxalidaceae | *Oxalis exserta* | EU436967 |
| Angiosperm | Oxalidaceae | *Oxalis fabifolia* | EU436925 |
| Angiosperm | Oxalidaceae | *Oxalis falcatula* | EU436992 |
| Angiosperm | Oxalidaceae | *Oxalis fibrosa* | EU436958 |
| Angiosperm | Oxalidaceae | *Oxalis flava* | EU436924 |
| Angiosperm | Oxalidaceae | *Oxalis flaviuscula* | EU436929 |
| Angiosperm | Oxalidaceae | *Oxalis fontana* | EU274620 |
| Angiosperm | Oxalidaceae | *Oxalis fontana* | EU274622 |
| Angiosperm | Oxalidaceae | *Oxalis fourcadei* | EU436923 |
| Angiosperm | Oxalidaceae | *Oxalis furcillata* | EU436952 |
| Angiosperm | Oxalidaceae | *Oxalis gracilipes* | EU436991 |
| Angiosperm | Oxalidaceae | *Oxalis gracilis* | EU436976 |
| Angiosperm | Oxalidaceae | *Oxalis grammopetala* | EU436906 |
| Angiosperm | Oxalidaceae | *Oxalis grammophylla* | EU437016 |
| Angiosperm | Oxalidaceae | *Oxalis haedulipes* | EU436886 |
| Angiosperm | Oxalidaceae | *Oxalis herrerae* | U74269 |
| Angiosperm | Oxalidaceae | *Oxalis heterophylla* | EU437018 |
| Angiosperm | Oxalidaceae | *Oxalis hirta* | EU436971 |
| Angiosperm | Oxalidaceae | *Oxalis hygrophila* | EU437024 |
| Angiosperm | Oxalidaceae | *Oxalis hypsophila* | EU436874 |
| Angiosperm | Oxalidaceae | *Oxalis imbricata* | EU436896 |
| Angiosperm | Oxalidaceae | *Oxalis incarnata* | EU436957 |
| Angiosperm | Oxalidaceae | *Oxalis inconspicua* | EU436944 |
| Angiosperm | Oxalidaceae | *Oxalis kamiesbergensis* | EU436970 |
| Angiosperm | Oxalidaceae | *Oxalis knuthiana* | EU436884 |
| Angiosperm | Oxalidaceae | *Oxalis lanata* | EU437027 |
| Angiosperm | Oxalidaceae | *Oxalis lateriflora* | EU436890 |
| Angiosperm | Oxalidaceae | *Oxalis latifolia* | EU436875 |
| Angiosperm | Oxalidaceae | *Oxalis leptogramma* | EU437014 |
| Angiosperm | Oxalidaceae | *Oxalis lichenoides* | EU436919 |
| Angiosperm | Oxalidaceae | *Oxalis linearis* | EU436972 |
| Angiosperm | Oxalidaceae | *Oxalis livida* | EU436889 |
| Angiosperm | Oxalidaceae | *Oxalis louisae* | EU436928 |
| Angiosperm | Oxalidaceae | *Oxalis luteola* | EU436904 |
| Angiosperm | Oxalidaceae | *Oxalis massoniana* | EU437015 |
| Angiosperm | Oxalidaceae | *Oxalis meisneri* | EU436993 |
| Angiosperm | Oxalidaceae | *Oxalis melanosticta* | EU436901 |
| Angiosperm | Oxalidaceae | *Oxalis minuta* | EU437028 |
| Angiosperm | Oxalidaceae | *Oxalis monophylla* | EU436927 |
| Angiosperm | Oxalidaceae | *Oxalis multicaulis* | EU436998 |
| Angiosperm | Oxalidaceae | *Oxalis namaquana* | EU436941 |
| Angiosperm | Oxalidaceae | *Oxalis natans* | EU437009 |
| Angiosperm | Oxalidaceae | *Oxalis nidulans* | EU437026 |
| Angiosperm | Oxalidaceae | *Oxalis nortieri* | EU436914 |
| Angiosperm | Oxalidaceae | *Oxalis obliquifolia* | EU436915 |
| Angiosperm | Oxalidaceae | *Oxalis obtusa* | EU436922 |
| Angiosperm | Oxalidaceae | *Oxalis oculifera* | EU436953 |
| Angiosperm | Oxalidaceae | *Oxalis oligophylla* | EU437023 |
| Angiosperm | Oxalidaceae | *Oxalis orbicularis* | EU436899 |
| Angiosperm | Oxalidaceae | *Oxalis oreophila* | EU436975 |
| Angiosperm | Oxalidaceae | *Oxalis ortgiesii* | U74273 |
| Angiosperm | Oxalidaceae | *Oxalis orthopoda* | EU436956 |
| Angiosperm | Oxalidaceae | *Oxalis pachyrrhiza* | U74275 |
| Angiosperm | Oxalidaceae | *Oxalis palmifrons* | EU437021 |
| Angiosperm | Oxalidaceae | *Oxalis pendulifolia* | EU437019 |
| Angiosperm | Oxalidaceae | *Oxalis perdicaria* | EU436878 |
| Angiosperm | Oxalidaceae | *Oxalis phloxidiflora* | EU436984 |
| Angiosperm | Oxalidaceae | *Oxalis pocockiae* | EU436911 |
| Angiosperm | Oxalidaceae | *Oxalis pseudocernua* | EU436880 |
| Angiosperm | Oxalidaceae | *Oxalis psilopoda* | EU436891 |
| Angiosperm | Oxalidaceae | *Oxalis pulchella* | EU436907 |
| Angiosperm | Oxalidaceae | *Oxalis punctata* | EU436917 |
| Angiosperm | Oxalidaceae | *Oxalis purpurascens* | EU436881 |
| Angiosperm | Oxalidaceae | *Oxalis purpurea* | EU436902 |
| Angiosperm | Oxalidaceae | *Oxalis salteri* | EU436932 |
| Angiosperm | Oxalidaceae | *Oxalis setosa* | EU436916 |
| Angiosperm | Oxalidaceae | *Oxalis smithiana* | EU436964 |
| Angiosperm | Oxalidaceae | *Oxalis stellata* | EU436894 |
| Angiosperm | Oxalidaceae | *Oxalis stenopetala* | EU436983 |
| Angiosperm | Oxalidaceae | *Oxalis stenoptera* | EU436934 |
| Angiosperm | Oxalidaceae | *Oxalis stictocheila* | EU436996 |
| Angiosperm | Oxalidaceae | *Oxalis stricta* | EU436869 |
| Angiosperm | Oxalidaceae | *Oxalis strigosa* | EU436959 |
| Angiosperm | Oxalidaceae | *Oxalis suavis* | EU436937 |
| Angiosperm | Oxalidaceae | *Oxalis suteroides* | EU436963 |
| Angiosperm | Oxalidaceae | *Oxalis tenella* | EU436978 |
| Angiosperm | Oxalidaceae | *Oxalis tenuifolia* | EU436995 |
| Angiosperm | Oxalidaceae | *Oxalis tenuipes* | EU436986 |
| Angiosperm | Oxalidaceae | *Oxalis tenuis* | EU436989 |
| Angiosperm | Oxalidaceae | *Oxalis tetraphylla* | EU436877 |
| Angiosperm | Oxalidaceae | *Oxalis tomentosa* | EU437022 |
| Angiosperm | Oxalidaceae | *Oxalis truncatula* | EU436960 |
| Angiosperm | Oxalidaceae | *Oxalis uliginosa* | EU436933 |
| Angiosperm | Oxalidaceae | *Oxalis urbaniana* | EU436999 |
| Angiosperm | Oxalidaceae | *Oxalis valdiviensis* | EU436873 |
| Angiosperm | Oxalidaceae | *Oxalis versicolor* | EU437006 |
| Angiosperm | Oxalidaceae | *Oxalis violacea* | EF590789 |
| Angiosperm | Oxalidaceae | *Oxalis virginea* | EU436955 |
| Angiosperm | Oxalidaceae | *Oxalis viscosa* | EU436949 |
| Angiosperm | Oxalidaceae | *Oxalis xantha* | EU436980 |
| Angiosperm | Oxalidaceae | *Oxalis yungasensis* | U74286 |
| Angiosperm | Oxalidaceae | *Oxalis zeekoevleyensis* | EU436900 |
| Angiosperm | Oxalidaceae | *Oxalis zeyheri* | EU436962 |
| Angiosperm | Palmaceae | *Daemonorops didymophylla* | AJ242071 |
| Angiosperm | Palmaceae | *Daemonorops didymophylla* | AJ242072 |
| Angiosperm | Palmae | *Areca catechu* | AB271407 |
| Angiosperm | Palmae | *Areca catechu* | AB271408 |
| Angiosperm | Palmae | *Areca hutchinsoniana* | AB271409 |
| Angiosperm | Palmae | *Areca hutchinsoniana* | AB271410 |
| Angiosperm | Palmae | *Areca macrocalyx* | AB271411 |
| Angiosperm | Palmae | *Areca macrocalyx* | AB271412 |
| Angiosperm | Palmae | *Areca triandra* | AB271413 |
| Angiosperm | Palmae | *Areca triandra* | AB271414 |
| Angiosperm | Palmae | *Areca tunku* | AB271415 |
| Angiosperm | Palmae | *Areca tunku* | AB271417 |
| Angiosperm | Palmae | *Areca vestiaria* | AB271419 |
| Angiosperm | Palmae | *Areca vestiaria* | AB271420 |
| Angiosperm | Palmae | *Areca vestiaria* | AB271421 |
| Angiosperm | Palmae | *Trachycarpus martianus* | EF635378 |
| Angiosperm | Palmae | *Trachycarpus wagnerianus* | EF635379 |
| Angiosperm | Pandanaceae | *Pandanus tectorius* | EU816709 |
| Angiosperm | Papaveraceae | *Chelidonium majus* | DQ912878 |
| Angiosperm | Papaveraceae | *Corydalis ambigua* | DQ912888 |
| Angiosperm | Papaveraceae | *Corydalis flavula* | DQ006011 |
| Angiosperm | Papaveraceae | *Corydalis incisa* | DQ912889 |
| Angiosperm | Papaveraceae | *Hylomecon hylomeconoides* | DQ912876 |
| Angiosperm | Papaveraceae | *Hylomecon vernalis* | DQ912877 |
| Angiosperm | Papaveraceae | *Papaver bracteatum* | DQ912881 |
| Angiosperm | Papaveraceae | *Papaver nudicaule* | DQ912885 |
| Angiosperm | Papaveraceae | *Papaver orientale* | DQ912882 |
| Angiosperm | Papaveraceae | *Papaver radicatum* | DQ912879 |
| Angiosperm | Papaveraceae | *Papaver rhoeas* | DQ912886 |
| Angiosperm | Passifloraceae | *Passiflora alata* | AF454800 |
| Angiosperm | Passifloraceae | *Passiflora ambigua* | AF454801 |
| Angiosperm | Passifloraceae | *Passiflora arbelaezii* | DQ521278 |
| Angiosperm | Passifloraceae | *Passiflora auriculata* | AF454804 |
| Angiosperm | Passifloraceae | *Passiflora auriculata* | DQ284532 |
| Angiosperm | Passifloraceae | *Passiflora biflora* | AF454805 |
| Angiosperm | Passifloraceae | *Passiflora biflora* | AY632705 |
| Angiosperm | Passifloraceae | *Passiflora biflora* | DQ521281 |
| Angiosperm | Passifloraceae | *Passiflora caerulea* | AF454802 |
| Angiosperm | Passifloraceae | *Passiflora caerulea* | DQ521375 |
| Angiosperm | Passifloraceae | *Passiflora cinnabarina* | AY632706 |
| Angiosperm | Passifloraceae | *Passiflora citrifolia* | AY632707 |
| Angiosperm | Passifloraceae | *Passiflora coriacea* | AF454807 |
| Angiosperm | Passifloraceae | *Passiflora coriacea x Passiflora xiikzodz* | DQ087420 |
| Angiosperm | Passifloraceae | *Passiflora cupiformis* | AY632708 |
| Angiosperm | Passifloraceae | *Passiflora edulis* | AF454803 |
| Angiosperm | Passifloraceae | *Passiflora filipes* | AY632709 |
| Angiosperm | Passifloraceae | *Passiflora foetida* | DQ521376 |
| Angiosperm | Passifloraceae | *Passiflora henryi* | AY632710 |
| Angiosperm | Passifloraceae | *Passiflora herbertiana* | AY632711 |
| Angiosperm | Passifloraceae | *Passiflora jugorum* | AY632712 |
| Angiosperm | Passifloraceae | *Passiflora lobata* | AF454808 |
| Angiosperm | Passifloraceae | *Passiflora lutea* | DQ006022 |
| Angiosperm | Passifloraceae | *Passiflora membranacea* | AY632701 |
| Angiosperm | Passifloraceae | *Passiflora mexicana* | AY632713 |
| Angiosperm | Passifloraceae | *Passiflora moluccana* var. *glaberrima* | DQ284536 |
| Angiosperm | Passifloraceae | *Passiflora moluccana* var. *teysmanniana* | DQ087421 |
| Angiosperm | Passifloraceae | *Passiflora monadelpha* | DQ087418 |
| Angiosperm | Passifloraceae | *Passiflora multiflora* | AY632715 |
| Angiosperm | Passifloraceae | *Passiflora murucuja* | AY648559 |
| Angiosperm | Passifloraceae | *Passiflora oerstedii* | AF454797 |
| Angiosperm | Passifloraceae | *Passiflora perakensis* | DQ087422 |
| Angiosperm | Passifloraceae | *Passiflora platyloba* | AF454798 |
| Angiosperm | Passifloraceae | *Passiflora quadrangularis* | AF454799 |
| Angiosperm | Passifloraceae | *Passiflora quadrangularis* | AY636107 |
| Angiosperm | Passifloraceae | *Passiflora rubra* | AY632716 |
| Angiosperm | Passifloraceae | *Passiflora siamica* | AY632717 |
| Angiosperm | Passifloraceae | *Passiflora suberosa* | AF454806 |
| Angiosperm | Passifloraceae | *Passiflora suberosa* | AY632718 |
| Angiosperm | Passifloraceae | *Passiflora talamancensis* | AF454809 |
| Angiosperm | Passifloraceae | *Passiflora tenuiloba* | AY632719 |
| Angiosperm | Passifloraceae | *Passiflora tonkinensis* | DQ087424 |
| Angiosperm | Passifloraceae | *Passiflora vitifolia* | AF454796 |
| Angiosperm | Passifloraceae | *Passiflora wilsonii* | DQ087425 |
| Angiosperm | Pedaliaceae | *Sesamum indicum* | AF478946 |
| Angiosperm | Phrymaceae | *Phryma leptostachya* var. *asiatica* | AF478924 |
| Angiosperm | Phrymaceae | *Phryma leptostachya* var. *asiatica* | DQ533809 |
| Angiosperm | Phrymaceae | *Phryma leptostachya* var. *asiatica* | DQ533811 |
| Angiosperm | Phrymaceae | *Phryma leptostachya* var. *asiatica* | DQ533814 |
| Angiosperm | Phrymaceae | *Phryma leptostachya* var. *asiatica* | DQ533817 |
| Angiosperm | Phrymaceae | *Phryma leptostachya* var. *asiatica* | DQ533819 |
| Angiosperm | Phytolaccaceae | *Phytolacca acinosa* | EU239681 |
| Angiosperm | Piperaceae | *Peperomia pellucida* | EF450291 |
| Angiosperm | Piperaceae | *Peperomia tetraphylla* | AF203631 |
| Angiosperm | Piperaceae | *Piper austrosinense* | EF450277 |
| Angiosperm | Piperaceae | *Piper austrosinense* | EF450278 |
| Angiosperm | Piperaceae | *Piper betle* | EF450279 |
| Angiosperm | Piperaceae | *Piper betle* | EF450280 |
| Angiosperm | Piperaceae | *Piper chinense* | EF450287 |
| Angiosperm | Piperaceae | *Piper hancei* | EF450270 |
| Angiosperm | Piperaceae | *Piper hancei* | EF450271 |
| Angiosperm | Piperaceae | *Piper hancei* | EF450272 |
| Angiosperm | Piperaceae | *Piper kadsura* | EF450290 |
| Angiosperm | Piperaceae | *Piper laetispicum* | EF450259 |
| Angiosperm | Piperaceae | *Piper laetispicum* | EF450260 |
| Angiosperm | Piperaceae | *Piper laetispicum* | EF450263 |
| Angiosperm | Piperaceae | *Piper longum* | EF450288 |
| Angiosperm | Piperaceae | *Piper sarmentosum* | EF450285 |
| Angiosperm | Piperaceae | *Piper sarmentosum* | EF450286 |
| Angiosperm | Piperaceae | *Piper wallichii* | EF450289 |
| Angiosperm | Pittosporaceae | *Pittosporum angustifolium* | AY829029 |
| Angiosperm | Pittosporaceae | *Pittosporum bracteolatum* | EF635474 |
| Angiosperm | Pittosporaceae | *Pittosporum crassifolium* | EF635471 |
| Angiosperm | Pittosporaceae | *Pittosporum eugenioides* | AY829031 |
| Angiosperm | Pittosporaceae | *Pittosporum fairchildii* | EF635472 |
| Angiosperm | Pittosporaceae | *Pittosporum ferrugineum* | AY829032 |
| Angiosperm | Pittosporaceae | *Pittosporum lancifolium* | AY829033 |
| Angiosperm | Pittosporaceae | *Pittosporum ligustrifolium* | AY829034 |
| Angiosperm | Pittosporaceae | *Pittosporum moluccanum* | AY829035 |
| Angiosperm | Pittosporaceae | *Pittosporum multiflorum* | AY829036 |
| Angiosperm | Pittosporaceae | *Pittosporum oreillyanum* | AY829037 |
| Angiosperm | Pittosporaceae | *Pittosporum ralphii* | EF635473 |
| Angiosperm | Pittosporaceae | *Pittosporum revolutum* | AY829038 |
| Angiosperm | Pittosporaceae | *Pittosporum rubiginosum* | AY829039 |
| Angiosperm | Pittosporaceae | *Pittosporum spinescens* | AY829040 |
| Angiosperm | Pittosporaceae | *Pittosporum suberosum* | AY829041 |
| Angiosperm | Pittosporaceae | *Pittosporum trilobum* | AY829042 |
| Angiosperm | Pittosporaceae | *Pittosporum venulosum* | AY829043 |
| Angiosperm | Pittosporaceae | *Pittosporum wingii* | AY829044 |
| Angiosperm | Plantaginaceae | *Plantago afra* | AY101892 |
| Angiosperm | Plantaginaceae | *Plantago albicans* | AY101905 |
| Angiosperm | Plantaginaceae | *Plantago alpina* | AY101877 |
| Angiosperm | Plantaginaceae | *Plantago amplexicaulis* | AY101900 |
| Angiosperm | Plantaginaceae | *Plantago arborescens* | AJ548954 |
| Angiosperm | Plantaginaceae | *Plantago arborescens* | AY101886 |
| Angiosperm | Plantaginaceae | *Plantago arenaria* | AY101891 |
| Angiosperm | Plantaginaceae | *Plantago arenaria* | AY692082 |
| Angiosperm | Plantaginaceae | *Plantago aristata* | AJ548983 |
| Angiosperm | Plantaginaceae | *Plantago aristata* | AY101911 |
| Angiosperm | Plantaginaceae | *Plantago asiatica* | AJ548977 |
| Angiosperm | Plantaginaceae | *Plantago asiatica* | AY101862 |
| Angiosperm | Plantaginaceae | *Plantago asiatica* var. *densiuscula* | AB223154 |
| Angiosperm | Plantaginaceae | *Plantago asiatica* var. *densiuscula* | AB223156 |
| Angiosperm | Plantaginaceae | *Plantago asiatica* var. *densiuscula* | AB223162 |
| Angiosperm | Plantaginaceae | *Plantago atrata* | AY101895 |
| Angiosperm | Plantaginaceae | *Plantago australis* | AY101874 |
| Angiosperm | Plantaginaceae | *Plantago bellardii* | AY101902 |
| Angiosperm | Plantaginaceae | *Plantago camtschatica* | AB281169 |
| Angiosperm | Plantaginaceae | *Plantago camtschatica* | AJ548971 |
| Angiosperm | Plantaginaceae | *Plantago ciliata* | AY101906 |
| Angiosperm | Plantaginaceae | *Plantago cornuti* | AY101859 |
| Angiosperm | Plantaginaceae | *Plantago crassifolia* | AY101881 |
| Angiosperm | Plantaginaceae | *Plantago cretica* | AY101901 |
| Angiosperm | Plantaginaceae | *Plantago daltonii* | AJ548968 |
| Angiosperm | Plantaginaceae | *Plantago debilis* | AY101868 |
| Angiosperm | Plantaginaceae | *Plantago elongata* | AJ548974 |
| Angiosperm | Plantaginaceae | *Plantago erecta* | AJ548982 |
| Angiosperm | Plantaginaceae | *Plantago erecta* | AY101909 |
| Angiosperm | Plantaginaceae | *Plantago erosa* | AB281167 |
| Angiosperm | Plantaginaceae | *Plantago euryphylla* | AJ548966 |
| Angiosperm | Plantaginaceae | *Plantago famarae* | AY101888 |
| Angiosperm | Plantaginaceae | *Plantago heterophylla* | AJ548975 |
| Angiosperm | Plantaginaceae | *Plantago hispida* | AJ548967 |
| Angiosperm | Plantaginaceae | *Plantago hookeriana* | AY101913 |
| Angiosperm | Plantaginaceae | *Plantago hostifolia* | AB281166 |
| Angiosperm | Plantaginaceae | *Plantago lagopus* | AY101897 |
| Angiosperm | Plantaginaceae | *Plantago lagopus* | AY692078 |
| Angiosperm | Plantaginaceae | *Plantago lanceolata* | AB281171 |
| Angiosperm | Plantaginaceae | *Plantago lanceolata* | AY101898 |
| Angiosperm | Plantaginaceae | *Plantago lanceolata* | AY692077 |
| Angiosperm | Plantaginaceae | *Plantago lundborgii* | AY101907 |
| Angiosperm | Plantaginaceae | *Plantago macrorhiza* | AY101883 |
| Angiosperm | Plantaginaceae | *Plantago major* | AY101861 |
| Angiosperm | Plantaginaceae | *Plantago major* | AY692079 |
| Angiosperm | Plantaginaceae | *Plantago maritima* | AJ548986 |
| Angiosperm | Plantaginaceae | *Plantago maritima* | AY101879 |
| Angiosperm | Plantaginaceae | *Plantago mauritanica* | AY101890 |
| Angiosperm | Plantaginaceae | *Plantago maxima* | AY101864 |
| Angiosperm | Plantaginaceae | *Plantago media* | AJ548964 |
| Angiosperm | Plantaginaceae | *Plantago media* | AY101865 |
| Angiosperm | Plantaginaceae | *Plantago myosuros* | AY101873 |
| Angiosperm | Plantaginaceae | *Plantago nivalis* | AY101896 |
| Angiosperm | Plantaginaceae | *Plantago nubicola* | AJ548972 |
| Angiosperm | Plantaginaceae | *Plantago ovata* | AJ548973 |
| Angiosperm | Plantaginaceae | *Plantago ovata* | AY101903 |
| Angiosperm | Plantaginaceae | *Plantago ovata* | EU381196 |
| Angiosperm | Plantaginaceae | *Plantago palmata* | AY101860 |
| Angiosperm | Plantaginaceae | *Plantago paradoxa* | AJ548969 |
| Angiosperm | Plantaginaceae | *Plantago patagonica* | AY101912 |
| Angiosperm | Plantaginaceae | *Plantago raoulii* | AY101867 |
| Angiosperm | Plantaginaceae | *Plantago rhodosperma* | AJ548976 |
| Angiosperm | Plantaginaceae | *Plantago rhodosperma* | AY692081 |
| Angiosperm | Plantaginaceae | *Plantago rigida* | AY101876 |
| Angiosperm | Plantaginaceae | *Plantago sarcophylla* | AY101893 |
| Angiosperm | Plantaginaceae | *Plantago sempervirens* | AY101889 |
| Angiosperm | Plantaginaceae | *Plantago sericea* | AY101910 |
| Angiosperm | Plantaginaceae | *Plantago serraria* | AY101880 |
| Angiosperm | Plantaginaceae | *Plantago sparsiflora* | AJ548979 |
| Angiosperm | Plantaginaceae | *Plantago spathulata* | AY101869 |
| Angiosperm | Plantaginaceae | *Plantago squarrosa* | AY101894 |
| Angiosperm | Plantaginaceae | *Plantago stauntonii* | AY101870 |
| Angiosperm | Plantaginaceae | *Plantago stocksii* | AY101904 |
| Angiosperm | Plantaginaceae | *Plantago subspathulata* | AY101884 |
| Angiosperm | Plantaginaceae | *Plantago subulata* | AY101878 |
| Angiosperm | Plantaginaceae | *Plantago tandilensis* | AY101908 |
| Angiosperm | Plantaginaceae | *Plantago tasmanica* | AJ548970 |
| Angiosperm | Plantaginaceae | *Plantago tenuiflora* | AY101866 |
| Angiosperm | Plantaginaceae | *Plantago tomentosa* | AY101872 |
| Angiosperm | Plantaginaceae | *Plantago triandra* | AJ548965 |
| Angiosperm | Plantaginaceae | *Plantago trinitatis* | AY101871 |
| Angiosperm | Plantaginaceae | *Plantago virginica* | AB281170 |
| Angiosperm | Plantaginaceae | *Plantago webbii* | AY101887 |
| Angiosperm | Poaceae | *Aegilops tauschii* | AJ301802 |
| Angiosperm | Poaceae | *Arrhenatherum album* var. *album* | AJ632191 |
| Angiosperm | Poaceae | *Arrhenatherum album* var. *album* | AJ632192 |
| Angiosperm | Poaceae | *Arrhenatherum album* var. *erianthum* | AJ632180 |
| Angiosperm | Poaceae | *Arrhenatherum album* var. *erianthum* | AJ632182 |
| Angiosperm | Poaceae | *Arrhenatherum album* var. *erianthum* | AJ632185 |
| Angiosperm | Poaceae | *Arrhenatherum calderae* | AJ632229 |
| Angiosperm | Poaceae | *Arrhenatherum calderae* | AJ632230 |
| Angiosperm | Poaceae | *Arrhenatherum calderae* | AJ632231 |
| Angiosperm | Poaceae | *Arrhenatherum elatius* | AJ632163 |
| Angiosperm | Poaceae | *Arrhenatherum elatius* | AJ632167 |
| Angiosperm | Poaceae | *Arrhenatherum elatius* | AJ632169 |
| Angiosperm | Poaceae | *Arrhenatherum elatius* | AJ632173 |
| Angiosperm | Poaceae | *Arrhenatherum elatius* | AJ632174 |
| Angiosperm | Poaceae | *Arrhenatherum elatius* subsp. *elatius* | AJ632226 |
| Angiosperm | Poaceae | *Arrhenatherum elatius* subsp. *elatius* | AJ632228 |
| Angiosperm | Poaceae | *Arrhenatherum elatius* subsp. *sardoum* | AJ632247 |
| Angiosperm | Poaceae | *Arrhenatherum kotschyi* | AJ632233 |
| Angiosperm | Poaceae | *Arrhenatherum kotschyi* | AJ632234 |
| Angiosperm | Poaceae | *Arrhenatherum kotschyi* | AJ632235 |
| Angiosperm | Poaceae | *Arrhenatherum nebrodense* | AJ632236 |
| Angiosperm | Poaceae | *Arrhenatherum nebrodense* | AJ632237 |
| Angiosperm | Poaceae | *Arrhenatherum palaestinum* | AJ632239 |
| Angiosperm | Poaceae | *Arrhenatherum palaestinum* | AJ632241 |
| Angiosperm | Poaceae | *Avena longiglumis* | AY522436 |
| Angiosperm | Poaceae | *Avena macrostachya* | AY522433 |
| Angiosperm | Poaceae | *Avena sativa* | AY520821 |
| Angiosperm | Poaceae | *Avena ventricosa* | AY522437 |
| Angiosperm | Poaceae | *Avenula hookeri* | AY870327 |
| Angiosperm | Poaceae | *Bambusa balcooa* | EU244594 |
| Angiosperm | Poaceae | *Bambusa bambos* | DQ915808 |
| Angiosperm | Poaceae | *Bambusa beecheyana* | AY839720 |
| Angiosperm | Poaceae | *Bambusa chungii* | AY839709 |
| Angiosperm | Poaceae | *Bambusa contracta* | AY839703 |
| Angiosperm | Poaceae | *Bambusa emeiensis* | AY839711 |
| Angiosperm | Poaceae | *Bambusa flexuosa* | AY839701 |
| Angiosperm | Poaceae | *Bambusa hainanensis* | AY839702 |
| Angiosperm | Poaceae | *Bambusa membranacea* | AY839704 |
| Angiosperm | Poaceae | *Bambusa multiplex* | AY839710 |
| Angiosperm | Poaceae | *Bambusa nutans* | AY839706 |
| Angiosperm | Poaceae | *Bambusa oldhamii* | AY839707 |
| Angiosperm | Poaceae | *Bambusa sinospinosa* | AY839714 |
| Angiosperm | Poaceae | *Bambusa subaequalis* | AY839712 |
| Angiosperm | Poaceae | *Bambusa textilis* | AY839717 |
| Angiosperm | Poaceae | *Bambusa tuldoides* | AY839708 |
| Angiosperm | Poaceae | *Bromus ayacuchensis* | DQ676867 |
| Angiosperm | Poaceae | *Catabrosa aquatica* | EF577510 |
| Angiosperm | Poaceae | *Coix lacryma-jobi* | DQ005034 |
| Angiosperm | Poaceae | *Eleusine coracana* | AJ272185 |
| Angiosperm | Poaceae | *Hierochloe equiseta* | AY705901 |
| Angiosperm | Poaceae | *Hierochloe fusca* | AY705902 |
| Angiosperm | Poaceae | *Hierochloe novae-zelandiae* | AY705900 |
| Angiosperm | Poaceae | *Hordeum bogdanii* | AY740876 |
| Angiosperm | Poaceae | *Hordeum brevisubulatum* | AY740877 |
| Angiosperm | Poaceae | *Hymenachne grumosa* | AY129708 |
| Angiosperm | Poaceae | *Imperata cylindrica* | AF092512 |
| Angiosperm | Poaceae | *Lolium multiflorum* | EU050655 |
| Angiosperm | Poaceae | *Lolium multiflorum* | EU814857 |
| Angiosperm | Poaceae | *Lolium multiflorum* | EU814859 |
| Angiosperm | Poaceae | *Lolium multiflorum* | EU814864 |
| Angiosperm | Poaceae | *Lolium multiflorum* | EU814867 |
| Angiosperm | Poaceae | *Lolium persicum* | AJ240147 |
| Angiosperm | Poaceae | *Lolium rigidum* | AJ240142 |
| Angiosperm | Poaceae | *Lolium rigidum* | AJ240144 |
| Angiosperm | Poaceae | *Lolium subulatum* | AJ240148 |
| Angiosperm | Poaceae | *Lolium temulentum* | AJ240145 |
| Angiosperm | Poaceae | *Lolium temulentum* | EU050653 |
| Angiosperm | Poaceae | *Megathyrsus maximus* | AY129712 |
| Angiosperm | Poaceae | *Oryza australiensis* | DQ888638 |
| Angiosperm | Poaceae | *Oryza rufipogon* | DQ888639 |
| Angiosperm | Poaceae | *Panicum amarum* | AY129693 |
| Angiosperm | Poaceae | *Panicum anceps* | AY129694 |
| Angiosperm | Poaceae | *Panicum antidotale* | AY129695 |
| Angiosperm | Poaceae | *Panicum bergii* | AY129696 |
| Angiosperm | Poaceae | *Panicum bisulcatum* | AY129697 |
| Angiosperm | Poaceae | *Panicum boliviense* | AY129698 |
| Angiosperm | Poaceae | *Panicum capillare* | AY129700 |
| Angiosperm | Poaceae | *Panicum coloratum* var. *coloratum* | AY129701 |
| Angiosperm | Poaceae | *Panicum coloratum* var. *makarikariensis* | AY129702 |
| Angiosperm | Poaceae | *Panicum deustum* | AY129705 |
| Angiosperm | Poaceae | *Panicum dichotomiflorum* | AY129706 |
| Angiosperm | Poaceae | *Panicum dregeanum* | AY129707 |
| Angiosperm | Poaceae | *Panicum hallii* | AY129692 |
| Angiosperm | Poaceae | *Panicum infestum* | AY129709 |
| Angiosperm | Poaceae | *Panicum lanipes* | AY129710 |
| Angiosperm | Poaceae | *Panicum miliaceum* | AY129713 |
| Angiosperm | Poaceae | *Panicum miliaceum* | AY129714 |
| Angiosperm | Poaceae | *Panicum milioides* | AY129717 |
| Angiosperm | Poaceae | *Panicum natalense* | AY129718 |
| Angiosperm | Poaceae | *Panicum prionitis* | AY129720 |
| Angiosperm | Poaceae | *Panicum queenslandicum* | AY129721 |
| Angiosperm | Poaceae | *Panicum repens* | AY129722 |
| Angiosperm | Poaceae | *Panicum schinzii* | AY129723 |
| Angiosperm | Poaceae | *Panicum stapfianum* | AY129724 |
| Angiosperm | Poaceae | *Panicum subalbidum* | AY129725 |
| Angiosperm | Poaceae | *Panicum virgatum* | AM404348 |
| Angiosperm | Poaceae | *Panicum virgatum* | AY129727 |
| Angiosperm | Poaceae | *Panicum virgatum* | AY129728 |
| Angiosperm | Poaceae | *Panicum virgatum* | AY129729 |
| Angiosperm | Poaceae | *Panicum virgatum* | AY129730 |
| Angiosperm | Poaceae | *Panicum whitei* | AY129731 |
| Angiosperm | Poaceae | *Paracolpodium altaicum* | EF432735 |
| Angiosperm | Poaceae | *Phragmites australis* | AF019810 |
| Angiosperm | Poaceae | *Phyllostachys edulis* | AF019789 |
| Angiosperm | Poaceae | *Phyllostachys nidularia* | DQ131503 |
| Angiosperm | Poaceae | *Sarga leioclada* | DQ888629 |
| Angiosperm | Poaceae | *Sarga leioclada* | DQ888630 |
| Angiosperm | Poaceae | *Schizostachyum blumei* | DQ131536 |
| Angiosperm | Poaceae | *Schizostachyum dumetorum* | DQ131530 |
| Angiosperm | Poaceae | *Schizostachyum funghomii* | DQ131528 |
| Angiosperm | Poaceae | *Schizostachyum gracile* | DQ131538 |
| Angiosperm | Poaceae | *Schizostachyum jaculans* | DQ131531 |
| Angiosperm | Poaceae | *Schizostachyum pseudolima* | DQ131529 |
| Angiosperm | Poaceae | *Schizostachyum sanguineum* | DQ131533 |
| Angiosperm | Poaceae | *Schizostachyum xinwuense* | DQ131532 |
| Angiosperm | Poaceae | *Schizostachyum zollingeri* | DQ131534 |
| Angiosperm | Poaceae | *Secale strictum* subsp. *africanum* | AJ409209 |
| Angiosperm | Poaceae | *Secale strictum* subsp. *anatolicum* | AJ409208 |
| Angiosperm | Poaceae | *Secale strictum* subsp. *kuprijanovii* | AJ409205 |
| Angiosperm | Poaceae | *Secale sylvestre* | AJ409210 |
| Angiosperm | Poaceae | *Secale sylvestre* | AJ409211 |
| Angiosperm | Poaceae | *Secale sylvestre* | AJ409212 |
| Angiosperm | Poaceae | *Secale vavilovii* | AJ409204 |
| Angiosperm | Poaceae | *Sorghum nitidum* | DQ888636 |
| Angiosperm | Poaceae | *Steinchisma decipiens* | AY129703 |
| Angiosperm | Poaceae | *Steinchisma laxa* | AY129711 |
| Angiosperm | Poaceae | *Triticum aestivum* | AF438188 |
| Angiosperm | Poaceae | *Triticum aestivum* | AF438191 |
| Angiosperm | Poaceae | *Triticum aestivum* | AF440676 |
| Angiosperm | Poaceae | *Triticum aestivum* | AF521903 |
| Angiosperm | Poaceae | *Triticum aestivum* | AJ301799 |
| Angiosperm | Poaceae | *Triticum aestivum* | AM040486 |
| Angiosperm | Poaceae | *Triticum aestivum* | AY346110 |
| Angiosperm | Poaceae | *Triticum aestivum* | AY346111 |
| Angiosperm | Poaceae | *Triticum aestivum* | AY346112 |
| Angiosperm | Poaceae | *Triticum aestivum* | AY346113 |
| Angiosperm | Poaceae | *Triticum aestivum* | AY346114 |
| Angiosperm | Poaceae | *Triticum aestivum* | AY346117 |
| Angiosperm | Poaceae | *Triticum aestivum* | AY346118 |
| Angiosperm | Poaceae | *Triticum aestivum* | AY346120 |
| Angiosperm | Poaceae | *Triticum aestivum* | AY450258 |
| Angiosperm | Poaceae | *Triticum aestivum* | DQ981408 |
| Angiosperm | Poaceae | *Triticum aestivum* | DQ981409 |
| Angiosperm | Poaceae | *Triticum aestivum* | DQ981410 |
| Angiosperm | Poaceae | *Triticum monococcum* | AJ301800 |
| Angiosperm | Poaceae | *Triticum monococcum* subsp. *aegilopoides* | AY450259 |
| Angiosperm | Poaceae | *Triticum monococcum* subsp. *aegilopoides* | AY450260 |
| Angiosperm | Poaceae | *Triticum monococcum* subsp. *aegilopoides* | AY450261 |
| Angiosperm | Poaceae | *Triticum monococcum* subsp. *aegilopoides* | AY450262 |
| Angiosperm | Poaceae | *Triticum monococcum* subsp. *aegilopoides* | AY450263 |
| Angiosperm | Poaceae | *Triticum monococcum* subsp. *aegilopoides* | AY450264 |
| Angiosperm | Poaceae | *Triticum urartu* | AJ301803 |
| Angiosperm | Poaceae | *Triticum urartu* | AY450265 |
| Angiosperm | Poaceae | *Zizania aquatica* | AF057999 |
| Angiosperm | Polygalaceae | *Polygala bracteolata* | AJ812646 |
| Angiosperm | Polygalaceae | *Polygala chamaebuxus* | AJ812647 |
| Angiosperm | Polygalaceae | *Polygala myrtifolia* | AJ812650 |
| Angiosperm | Polygalaceae | *Polygala senega* | AJ812649 |
| Angiosperm | Polygalaceae | *Polygala tenuifolia* | DQ267099 |
| Angiosperm | Polygonaceae | *Chorizanthe angustifolia* | EU753735 |
| Angiosperm | Polygonaceae | *Chorizanthe angustifolia* | EU753739 |
| Angiosperm | Polygonaceae | *Chorizanthe cuspidata* var. *villosa* | EU753740 |
| Angiosperm | Polygonaceae | *Chorizanthe cuspidata* var. *villosa* | EU753744 |
| Angiosperm | Polygonaceae | *Chorizanthe diffusa* | EU753731 |
| Angiosperm | Polygonaceae | *Chorizanthe diffusa* | EU753732 |
| Angiosperm | Polygonaceae | *Chorizanthe douglasii* | EU753775 |
| Angiosperm | Polygonaceae | *Chorizanthe robusta* var. *robusta* | EU753765 |
| Angiosperm | Polygonaceae | *Chorizanthe robusta* var. *robusta* | EU753769 |
| Angiosperm | Polygonaceae | *Chorizanthe robusta* var. *robusta* | EU753773 |
| Angiosperm | Polygonaceae | *Chorizanthe valida* | EU753782 |
| Angiosperm | Polygonaceae | *Chorizanthe valida* | EU753784 |
| Angiosperm | Polygonaceae | *Chorizanthe valida* | EU753785 |
| Angiosperm | Polygonaceae | *Eriogonum nudum* var. *nudum* | EU753786 |
| Angiosperm | Polygonaceae | *Fagopyrum tataricum* | DQ780601 |
| Angiosperm | Polygonaceae | *Fallopia convolvulus* | AF040064 |
| Angiosperm | Polygonaceae | *Fallopia forbesii* | AF040072 |
| Angiosperm | Polygonaceae | *Fallopia japonica* | AF040070 |
| Angiosperm | Polygonaceae | *Fallopia japonica* | EU808015 |
| Angiosperm | Polygonaceae | *Fallopia multiflora* | EF016287 |
| Angiosperm | Polygonaceae | *Fallopia multiflora* | EF016288 |
| Angiosperm | Polygonaceae | *Fallopia multiflora* | EF016289 |
| Angiosperm | Polygonaceae | *Fallopia multiflora* | EF016290 |
| Angiosperm | Polygonaceae | *Fallopia multiflora* | EF016291 |
| Angiosperm | Polygonaceae | *Fallopia multiflora* | EF016292 |
| Angiosperm | Polygonaceae | *Fallopia multiflora* | EF532407 |
| Angiosperm | Polygonaceae | *Fallopia multiflora* | EU808016 |
| Angiosperm | Polygonaceae | *Fallopia multiflora* var. *angulata* | EF532408 |
| Angiosperm | Polygonaceae | *Fallopia sachalinensis* | AF040073 |
| Angiosperm | Polygonaceae | *Fallopia scandens* | AF040069 |
| Angiosperm | Polygonaceae | *Muehlenbeckia astonii* | AF040075 |
| Angiosperm | Polygonaceae | *Muehlenbeckia astonii* | EF635479 |
| Angiosperm | Polygonaceae | *Muehlenbeckia complexa* | AF040076 |
| Angiosperm | Polygonaceae | *Muehlenbeckia platyclada* | AF189738 |
| Angiosperm | Polygonaceae | *Muehlenbeckia rhyticarya* | AF189739 |
| Angiosperm | Polygonaceae | *Persicaria hydropiper* | U51275 |
| Angiosperm | Polygonaceae | *Persicaria posumbu* | DQ006030 |
| Angiosperm | Polygonaceae | *Polygonum sagittatum* | DQ006031 |
| Angiosperm | Polygonaceae | *Rheum tanguticum* | AM777851 |
| Angiosperm | Polygonaceae | *Rumex acetosella* | AF189730 |
| Angiosperm | Polygonaceae | *Rumex graminifolius* | AJ844277 |
| Angiosperm | Polygonaceae | *Rumex hastatus* | AF338218 |
| Angiosperm | Polygonaceae | *Rumex japonicus* | AF338220 |
| Angiosperm | Polygonaceae | *Rumex nepalensis* | AF338219 |
| Angiosperm | Polygonaceae | *Rumex roseus* | AJ844276 |
| Angiosperm | Polygonaceae | *Ruprechtia albida* | AY256537 |
| Angiosperm | Polygonaceae | *Ruprechtia albida* | AY256538 |
| Angiosperm | Polygonaceae | *Ruprechtia aperta* | AY256555 |
| Angiosperm | Polygonaceae | *Ruprechtia apetala* | AY256532 |
| Angiosperm | Polygonaceae | *Ruprechtia carina* | AY256553 |
| Angiosperm | Polygonaceae | *Ruprechtia cruegerii* | AY256549 |
| Angiosperm | Polygonaceae | *Ruprechtia fagifolia* | AY256536 |
| Angiosperm | Polygonaceae | *Ruprechtia fusca* | AY256530 |
| Angiosperm | Polygonaceae | *Ruprechtia fusca* | AY256531 |
| Angiosperm | Polygonaceae | *Ruprechtia laevigata* | AY256525 |
| Angiosperm | Polygonaceae | *Ruprechtia laevigata* | AY256544 |
| Angiosperm | Polygonaceae | *Ruprechtia pallida* | AY256528 |
| Angiosperm | Polygonaceae | *Ruprechtia ramiflora* | AY256545 |
| Angiosperm | Polygonaceae | *Ruprechtia ramiflora* | AY256550 |
| Angiosperm | Polygonaceae | *Ruprechtia ramiflora* | AY256551 |
| Angiosperm | Polygonaceae | *Ruprechtia ramiflora* | AY256552 |
| Angiosperm | Polygonaceae | *Ruprechtia ramiflora* | AY256554 |
| Angiosperm | Polygonaceae | *Ruprechtia tangarana* | AY256543 |
| Angiosperm | Polygonaceae | *Ruprechtia tenuiflora* | AY256548 |
| Angiosperm | Polygonaceae | *Triplaris gardneriana* | AY256520 |
| Angiosperm | Portulacaceae | *Portulaca jacobseniana* | L78045 |
| Angiosperm | Portulacaceae | *Portulaca molokiniensis* | L78046 |
| Angiosperm | Portulacaceae | *Portulaca oleracea* | L78047 |
| Angiosperm | Portulacaceae | *Portulaca quadrifida* | L78048 |
| Angiosperm | Portulacaceae | *Talinum paniculatum* | EU410357 |
| Angiosperm | Primulaceae | *Cortusa matthioli* subsp. *matthioli* | AY232294 |
| Angiosperm | Primulaceae | *Cortusa matthioli* subsp. *matthioli* | AY566866 |
| Angiosperm | Primulaceae | *Cortusa matthioli* subsp. *matthioli* | AY566867 |
| Angiosperm | Primulaceae | *Cortusa matthioli* subsp. *moravica* | AY232295 |
| Angiosperm | Primulaceae | *Lysimachia ciliata* | DQ006032 |
| Angiosperm | Primulaceae | *Primula davisii* | AY680710 |
| Angiosperm | Primulaceae | *Primula floribunda* | AY680707 |
| Angiosperm | Primulaceae | *Primula veris* | AY680704 |
| Angiosperm | Primulaceae | *Primula verticillata* | AY680732 |
| Angiosperm | Punicaceae | *Punica granatum* | AY035760 |
| Angiosperm | Punicaceae | *Punica granatum* | AY035761 |
| Angiosperm | Pyrolaceae | *Pyrola americana* | AF133737 |
| Angiosperm | Pyrolaceae | *Pyrola angustifolia* | AF133738 |
| Angiosperm | Pyrolaceae | *Pyrola aphylla* | AF133743 |
| Angiosperm | Pyrolaceae | *Pyrola asarifolia* | AF133736 |
| Angiosperm | Pyrolaceae | *Pyrola chlorantha* | AF133742 |
| Angiosperm | Pyrolaceae | *Pyrola minor* | AF133744 |
| Angiosperm | Pyrolaceae | *Pyrola minor* | AF133745 |
| Angiosperm | Pyrolaceae | *Pyrola picta* | AF352013 |
| Angiosperm | Pyrolaceae | *Pyrola rotundifolia* | AF091939 |
| Angiosperm | Ranunculaceae | *Aconitum alboviolaceum* | AY571363 |
| Angiosperm | Ranunculaceae | *Aconitum anthora* | AF216547 |
| Angiosperm | Ranunculaceae | *Aconitum brachypodum* | AY189789 |
| Angiosperm | Ranunculaceae | *Aconitum brunneum* | AY571344 |
| Angiosperm | Ranunculaceae | *Aconitum bulbilliferum* | AY571360 |
| Angiosperm | Ranunculaceae | *Aconitum bulleyanum* | AY189798 |
| Angiosperm | Ranunculaceae | *Aconitum campylorrhynchum* | AY571359 |
| Angiosperm | Ranunculaceae | *Aconitum cannabifolium* | AY189801 |
| Angiosperm | Ranunculaceae | *Aconitum carmichaelii* | AY571352 |
| Angiosperm | Ranunculaceae | *Aconitum chienningense* | AY164646 |
| Angiosperm | Ranunculaceae | *Aconitum chrysotrichum* | AY164642 |
| Angiosperm | Ranunculaceae | *Aconitum columbianum* | AF258683 |
| Angiosperm | Ranunculaceae | *Aconitum crassiflorum* | AY150230 |
| Angiosperm | Ranunculaceae | *Aconitum delphiniifolium* | AF258681 |
| Angiosperm | Ranunculaceae | *Aconitum episcopale* | AY189794 |
| Angiosperm | Ranunculaceae | *Aconitum episcopale* | AY571351 |
| Angiosperm | Ranunculaceae | *Aconitum finetianum* | AY164643 |
| Angiosperm | Ranunculaceae | *Aconitum flavum* | AY571357 |
| Angiosperm | Ranunculaceae | *Aconitum franchetii* | AY150236 |
| Angiosperm | Ranunculaceae | *Aconitum georgei* | AY189792 |
| Angiosperm | Ranunculaceae | *Aconitum gymnandrum* | AY150238 |
| Angiosperm | Ranunculaceae | *Aconitum hamatipetalum* | AY571355 |
| Angiosperm | Ranunculaceae | *Aconitum hemsleyanum* | AY189793 |
| Angiosperm | Ranunculaceae | *Aconitum huiliense* | AY571362 |
| Angiosperm | Ranunculaceae | *Aconitum karakolicum* | AY571358 |
| Angiosperm | Ranunculaceae | *Aconitum kusnezoffii* | AY571346 |
| Angiosperm | Ranunculaceae | *Aconitum legendrei* | AY571354 |
| Angiosperm | Ranunculaceae | *Aconitum liangshanicum* | AY571364 |
| Angiosperm | Ranunculaceae | *Aconitum liljestrandii* | AY164645 |
| Angiosperm | Ranunculaceae | *Aconitum lycoctonum* | AF216538 |
| Angiosperm | Ranunculaceae | *Aconitum lycoctonum* | AF216539 |
| Angiosperm | Ranunculaceae | *Aconitum lycoctonum* | AF216540 |
| Angiosperm | Ranunculaceae | *Aconitum lycoctonum* | AF216541 |
| Angiosperm | Ranunculaceae | *Aconitum lycoctonum* | AF216542 |
| Angiosperm | Ranunculaceae | *Aconitum lycoctonum* | AF216543 |
| Angiosperm | Ranunculaceae | *Aconitum lycoctonum* | AF216545 |
| Angiosperm | Ranunculaceae | *Aconitum lycoctonum* | AF216546 |
| Angiosperm | Ranunculaceae | *Aconitum lycoctonum* | AF216548 |
| Angiosperm | Ranunculaceae | *Aconitum lycoctonum* | AF216550 |
| Angiosperm | Ranunculaceae | *Aconitum lycoctonum* | AF216551 |
| Angiosperm | Ranunculaceae | *Aconitum monanthum* | AY189803 |
| Angiosperm | Ranunculaceae | *Aconitum nagarum* | AY189790 |
| Angiosperm | Ranunculaceae | *Aconitum nagarum* | AY571347 |
| Angiosperm | Ranunculaceae | *Aconitum napellus* | AF216544 |
| Angiosperm | Ranunculaceae | *Aconitum pendulum* | AY150235 |
| Angiosperm | Ranunculaceae | *Aconitum pilopetalum* | AY571343 |
| Angiosperm | Ranunculaceae | *Aconitum polycarpum* | AY571356 |
| Angiosperm | Ranunculaceae | *Aconitum pulchellum* | AY164647 |
| Angiosperm | Ranunculaceae | *Aconitum racemulosum* | AY150233 |
| Angiosperm | Ranunculaceae | *Aconitum refractum* | AY571349 |
| Angiosperm | Ranunculaceae | *Aconitum scaposum* | AY150231 |
| Angiosperm | Ranunculaceae | *Aconitum septentrionale* | AF216555 |
| Angiosperm | Ranunculaceae | *Aconitum sessiliflorum* | AY164648 |
| Angiosperm | Ranunculaceae | *Aconitum spiripetalum* | AY571345 |
| Angiosperm | Ranunculaceae | *Aconitum stapfianum* | AY189786 |
| Angiosperm | Ranunculaceae | *Aconitum sungpanense* var. *leucanthum* | AY189796 |
| Angiosperm | Ranunculaceae | *Aconitum tanguticum* | AY150234 |
| Angiosperm | Ranunculaceae | *Aconitum tatsienense* | AY571348 |
| Angiosperm | Ranunculaceae | *Aconitum tongolense* | AY571350 |
| Angiosperm | Ranunculaceae | *Aconitum tsaii* | AY189784 |
| Angiosperm | Ranunculaceae | *Aconitum tuguancunense* | AY189782 |
| Angiosperm | Ranunculaceae | *Aconitum variegatum* | AF216549 |
| Angiosperm | Ranunculaceae | *Aconitum vilmorinianum* | AY189787 |
| Angiosperm | Ranunculaceae | *Adonis vernalis* | AJ347910 |
| Angiosperm | Ranunculaceae | *Anemone antucensis* | AY056049 |
| Angiosperm | Ranunculaceae | *Anemone virginiana* | DQ006033 |
| Angiosperm | Ranunculaceae | *Cimicifuga acerina* | AF055041 |
| Angiosperm | Ranunculaceae | *Cimicifuga americana* | Z98280 |
| Angiosperm | Ranunculaceae | *Cimicifuga arizonica* | Z98281 |
| Angiosperm | Ranunculaceae | *Cimicifuga biternata* | Z98282 |
| Angiosperm | Ranunculaceae | *Cimicifuga brachycarpa* | Z98283 |
| Angiosperm | Ranunculaceae | *Cimicifuga dahurica* | Z98284 |
| Angiosperm | Ranunculaceae | *Cimicifuga elata* | Z98285 |
| Angiosperm | Ranunculaceae | *Cimicifuga europaea* | Z98286 |
| Angiosperm | Ranunculaceae | *Cimicifuga foetida* | Z98287 |
| Angiosperm | Ranunculaceae | *Cimicifuga frigida* | Z98288 |
| Angiosperm | Ranunculaceae | *Cimicifuga heracleifolia* | Z98290 |
| Angiosperm | Ranunculaceae | *Cimicifuga japonica* | Z98291 |
| Angiosperm | Ranunculaceae | *Cimicifuga kashmiriana* | Z98292 |
| Angiosperm | Ranunculaceae | *Cimicifuga laciniata* | Z98293 |
| Angiosperm | Ranunculaceae | *Cimicifuga mairei* | Z98294 |
| Angiosperm | Ranunculaceae | *Cimicifuga nanchuanensis* | AB044412 |
| Angiosperm | Ranunculaceae | *Cimicifuga purpurea* | Z98295 |
| Angiosperm | Ranunculaceae | *Cimicifuga racemosa* | Z98296 |
| Angiosperm | Ranunculaceae | *Cimicifuga rubifolia* | Z98297 |
| Angiosperm | Ranunculaceae | *Cimicifuga simplex* | AB194177 |
| Angiosperm | Ranunculaceae | *Cimicifuga yunnanensis* | Z98302 |
| Angiosperm | Ranunculaceae | *Clematis afoliata* | AJ347911 |
| Angiosperm | Ranunculaceae | *Coptis quinquefolia* | EF206702 |
| Angiosperm | Ranunculaceae | *Helleborus argutifolius* | AJ347920 |
| Angiosperm | Ranunculaceae | *Helleborus foetidus* | AJ347892 |
| Angiosperm | Ranunculaceae | *Helleborus lividus* | AJ347893 |
| Angiosperm | Ranunculaceae | *Helleborus multifidus* subsp. *bocconei* | AJ347894 |
| Angiosperm | Ranunculaceae | *Helleborus multifidus* subsp. *istriacus* | AJ347896 |
| Angiosperm | Ranunculaceae | *Helleborus multifidus* subsp. *multifidus* | AJ347897 |
| Angiosperm | Ranunculaceae | *Helleborus niger* | AJ347898 |
| Angiosperm | Ranunculaceae | *Helleborus orientalis* | AJ347901 |
| Angiosperm | Ranunculaceae | *Helleborus purpurascens* | AJ347902 |
| Angiosperm | Ranunculaceae | *Helleborus thibetanus* | AJ347904 |
| Angiosperm | Ranunculaceae | *Helleborus torquatus* | AJ347906 |
| Angiosperm | Ranunculaceae | *Helleborus vesicarius* | AJ347907 |
| Angiosperm | Ranunculaceae | *Helleborus viridis* subsp. *viridis* | AJ347908 |
| Angiosperm | Ranunculaceae | *Leptopyrum fumarioides* | AJ347912 |
| Angiosperm | Ranunculaceae | *Nigella damascena* | AB020376 |
| Angiosperm | Ranunculaceae | *Paeonia albiflora* | AY328313 |
| Angiosperm | Ranunculaceae | *Paeonia arietina* | U27671 |
| Angiosperm | Ranunculaceae | *Paeonia broteri* | U27673 |
| Angiosperm | Ranunculaceae | *Paeonia brownii* | U27674 |
| Angiosperm | Ranunculaceae | *Paeonia californica* | U27675 |
| Angiosperm | Ranunculaceae | *Paeonia intermedia* | DQ313698 |
| Angiosperm | Ranunculaceae | *Paeonia lactiflora* | U27682 |
| Angiosperm | Ranunculaceae | *Paeonia sinjiangensis* | U27697 |
| Angiosperm | Ranunculaceae | *Paeonia suffruticosa* subsp. *spontanea* | U27692 |
| Angiosperm | Ranunculaceae | *Paeonia veitchii* | U27695 |
| Angiosperm | Ranunculaceae | *Ranunculus bulbosus* | AM503891 |
| Angiosperm | Ranunculaceae | *Ranunculus sceleratus* | EF526405 |
| Angiosperm | Ranunculaceae | *Semiaquilegia adoxoides* | DQ410731 |
| Angiosperm | Ranunculaceae | *Thalictrum dioicum* | DQ006034 |
| Angiosperm | Ranunculaceae | *Thalictrum pubescens* | EU438836 |
| Angiosperm | Rhamnaceae | *Hovenia dulcis* | DQ146607 |
| Angiosperm | Rhamnaceae | *Hovenia trichocarpa* | DQ146608 |
| Angiosperm | Rhamnaceae | *Paliurus ramosissimus* | DQ146612 |
| Angiosperm | Rhamnaceae | *Ventilago madaraspatana* | DQ146616 |
| Angiosperm | Rhamnaceae | *Ziziphus acidojujuba* | DQ146572 |
| Angiosperm | Rhamnaceae | *Ziziphus amole* | DQ146579 |
| Angiosperm | Rhamnaceae | *Ziziphus celata* | DQ146581 |
| Angiosperm | Rhamnaceae | *Ziziphus celata* | DQ146582 |
| Angiosperm | Rhamnaceae | *Ziziphus guatemalensis* | DQ146585 |
| Angiosperm | Rhamnaceae | *Ziziphus jujuba* | DQ146576 |
| Angiosperm | Rhamnaceae | *Ziziphus lotus* | DQ146587 |
| Angiosperm | Rhamnaceae | *Ziziphus mauritiana* | DQ146588 |
| Angiosperm | Rhamnaceae | *Ziziphus mistol* | DQ146590 |
| Angiosperm | Rhamnaceae | *Ziziphus mucronata* | DQ146593 |
| Angiosperm | Rhamnaceae | *Ziziphus obtusifolia* var. *obtusifolia* | DQ146596 |
| Angiosperm | Rhamnaceae | *Ziziphus oenoplia* | DQ146598 |
| Angiosperm | Rhamnaceae | *Ziziphus pubescens* | DQ146600 |
| Angiosperm | Rhamnaceae | *Ziziphus rugosa* | DQ146601 |
| Angiosperm | Rhamnaceae | *Ziziphus taylori* | DQ146605 |
| Angiosperm | Rhamnaceae | *Ziziphus thyrsiflora* | DQ146606 |
| Angiosperm | Rhizophoraceae | *Carallia brachiata* | AF130320 |
| Angiosperm | Rhizophoraceae | *Carallia eugenioidea* | AF130321 |
| Angiosperm | Rhoipteleaceae | *Rhoiptelea chiliantha* | AF303800 |
| Angiosperm | Rosaceae | *Agrimonia parviflora* | AY634862 |
| Angiosperm | Rosaceae | *Agrimonia parviflora* | AY634863 |
| Angiosperm | Rosaceae | *Crataegus aestivalis* | EF127023 |
| Angiosperm | Rosaceae | *Crataegus brachyacantha* | EF127032 |
| Angiosperm | Rosaceae | *Crataegus calpodendron* | EF127018 |
| Angiosperm | Rosaceae | *Crataegus chlorosarca* | EF127009 |
| Angiosperm | Rosaceae | *Crataegus crus-galli* | EF127010 |
| Angiosperm | Rosaceae | *Crataegus dahurica* | EF127028 |
| Angiosperm | Rosaceae | *Crataegus heldreichii* | EF127016 |
| Angiosperm | Rosaceae | *Crataegus hupehensis* | EF127038 |
| Angiosperm | Rosaceae | *Crataegus kansuensis* | EF127029 |
| Angiosperm | Rosaceae | *Crataegus laevigata* | EF127015 |
| Angiosperm | Rosaceae | *Crataegus lassa* | EF127024 |
| Angiosperm | Rosaceae | *Crataegus marshallii* | EF127037 |
| Angiosperm | Rosaceae | *Crataegus maximowiczii* | EF127030 |
| Angiosperm | Rosaceae | *Crataegus mollis* | EF127012 |
| Angiosperm | Rosaceae | *Crataegus opaca* | EF127022 |
| Angiosperm | Rosaceae | *Crataegus pentagyna* | EF127035 |
| Angiosperm | Rosaceae | *Crataegus phaenopyrum* | EF127034 |
| Angiosperm | Rosaceae | *Crataegus pubescens* | EF127021 |
| Angiosperm | Rosaceae | *Crataegus punctata* | EF127011 |
| Angiosperm | Rosaceae | *Crataegus saligna* | EF127031 |
| Angiosperm | Rosaceae | *Crataegus sanguinea* | EF127027 |
| Angiosperm | Rosaceae | *Crataegus songarica* | EF127036 |
| Angiosperm | Rosaceae | *Crataegus spathulata* | EF127033 |
| Angiosperm | Rosaceae | *Crataegus suksdorfii* | EF127025 |
| Angiosperm | Rosaceae | *Crataegus suksdorfii* | EF127026 |
| Angiosperm | Rosaceae | *Crataegus triflora* | EF127019 |
| Angiosperm | Rosaceae | *Crataegus uniflora* | EF127020 |
| Angiosperm | Rosaceae | *Crataegus viridis* | EF127013 |
| Angiosperm | Rosaceae | *Duchesnea indica* | AY635025 |
| Angiosperm | Rosaceae | *Malus angustifolia* | EF127042 |
| Angiosperm | Rosaceae | *Polylepis australis* | AY634885 |
| Angiosperm | Rosaceae | *Polylepis australis* | AY634886 |
| Angiosperm | Rosaceae | *Polylepis besseri* subsp. *incarum* | AY634899 |
| Angiosperm | Rosaceae | *Polylepis besseri* subsp. *incarum* | AY634900 |
| Angiosperm | Rosaceae | *Polylepis besseri* subsp. *incarum* | AY634901 |
| Angiosperm | Rosaceae | *Polylepis besseri* subsp. *incarum* | AY634902 |
| Angiosperm | Rosaceae | *Polylepis besseri* subsp. *incarum* | AY634906 |
| Angiosperm | Rosaceae | *Polylepis besseri* subsp. *incarum* | AY634907 |
| Angiosperm | Rosaceae | *Polylepis besseri* subsp. *incarum* | AY634908 |
| Angiosperm | Rosaceae | *Polylepis besseri* subsp. *incarum* | AY634910 |
| Angiosperm | Rosaceae | *Polylepis besseri* subsp. *subtusalbida* | AY634911 |
| Angiosperm | Rosaceae | *Polylepis crista-galli* | AY634916 |
| Angiosperm | Rosaceae | *Polylepis crista-galli* | AY634919 |
| Angiosperm | Rosaceae | *Polylepis hieronymi* | AY634921 |
| Angiosperm | Rosaceae | *Polylepis incana* | AY634923 |
| Angiosperm | Rosaceae | *Polylepis incana* | AY634924 |
| Angiosperm | Rosaceae | *Polylepis incana* | AY634928 |
| Angiosperm | Rosaceae | *Polylepis lanuginosa* | AY634929 |
| Angiosperm | Rosaceae | *Polylepis lanuginosa* | AY634930 |
| Angiosperm | Rosaceae | *Polylepis lanuginosa* | AY634931 |
| Angiosperm | Rosaceae | *Polylepis lanuginosa* | AY634933 |
| Angiosperm | Rosaceae | *Polylepis lanuginosa* | AY634934 |
| Angiosperm | Rosaceae | *Polylepis lanuginosa* | AY634936 |
| Angiosperm | Rosaceae | *Polylepis multijuga* | AY634937 |
| Angiosperm | Rosaceae | *Polylepis multijuga* | AY634939 |
| Angiosperm | Rosaceae | *Polylepis multijuga* | AY634940 |
| Angiosperm | Rosaceae | *Polylepis neglecta* | AY634941 |
| Angiosperm | Rosaceae | *Polylepis neglecta* | AY634943 |
| Angiosperm | Rosaceae | *Polylepis neglecta* | AY634945 |
| Angiosperm | Rosaceae | *Polylepis pauta* | AY634950 |
| Angiosperm | Rosaceae | *Polylepis pauta* | AY634951 |
| Angiosperm | Rosaceae | *Polylepis pepei* | AY634953 |
| Angiosperm | Rosaceae | *Polylepis quadrijuga* | AY634954 |
| Angiosperm | Rosaceae | *Polylepis quadrijuga* | AY634955 |
| Angiosperm | Rosaceae | *Polylepis quadrijuga* | AY634960 |
| Angiosperm | Rosaceae | *Polylepis racemosa* subsp. *lanata* | AY634962 |
| Angiosperm | Rosaceae | *Polylepis racemosa* subsp. *triacontandra* | AY634965 |
| Angiosperm | Rosaceae | *Polylepis racemosa* subsp. *triacontandra* | AY634966 |
| Angiosperm | Rosaceae | *Polylepis racemosa* subsp. *triacontandra* | AY634967 |
| Angiosperm | Rosaceae | *Polylepis reticulata* | AY634975 |
| Angiosperm | Rosaceae | *Polylepis reticulata* | AY634976 |
| Angiosperm | Rosaceae | *Polylepis reticulata* | AY634979 |
| Angiosperm | Rosaceae | *Polylepis reticulata* | AY634980 |
| Angiosperm | Rosaceae | *Polylepis reticulata* | AY634981 |
| Angiosperm | Rosaceae | *Polylepis reticulata* | AY634983 |
| Angiosperm | Rosaceae | *Polylepis reticulata* | AY634984 |
| Angiosperm | Rosaceae | *Polylepis rugulosa* | AY634985 |
| Angiosperm | Rosaceae | *Polylepis rugulosa* | AY634986 |
| Angiosperm | Rosaceae | *Polylepis rugulosa* | AY634987 |
| Angiosperm | Rosaceae | *Polylepis rugulosa* | AY634988 |
| Angiosperm | Rosaceae | *Polylepis rugulosa* | AY634990 |
| Angiosperm | Rosaceae | *Polylepis rugulosa* | AY634991 |
| Angiosperm | Rosaceae | *Polylepis rugulosa* | AY634998 |
| Angiosperm | Rosaceae | *Polylepis sericea* | AY634999 |
| Angiosperm | Rosaceae | *Polylepis sericea* | AY635001 |
| Angiosperm | Rosaceae | *Polylepis sericea* | AY635003 |
| Angiosperm | Rosaceae | *Polylepis subsericans* | AY635008 |
| Angiosperm | Rosaceae | *Polylepis subsericans* | AY635009 |
| Angiosperm | Rosaceae | *Polylepis subsericans* | AY635011 |
| Angiosperm | Rosaceae | *Polylepis tarapacana* | AY635012 |
| Angiosperm | Rosaceae | *Polylepis tarapacana* | AY635013 |
| Angiosperm | Rosaceae | *Polylepis tarapacana* | AY635014 |
| Angiosperm | Rosaceae | *Polylepis tomentella* subsp. *tomentella* | AY635016 |
| Angiosperm | Rosaceae | *Polylepis tomentella* subsp. *tomentella* | AY635019 |
| Angiosperm | Rosaceae | *Polylepis tomentella* subsp. *tomentella* | AY635020 |
| Angiosperm | Rosaceae | *Polylepis tomentella* subsp. *tomentella* | AY635022 |
| Angiosperm | Rosaceae | *Polylepis tomentella* subsp. *tomentella* | AY635023 |
| Angiosperm | Rosaceae | *Polylepis weberbaueri* | AY635024 |
| Angiosperm | Rosaceae | *Prinsepia sinensis* | AF318751 |
| Angiosperm | Rosaceae | *Prunus andersonii* | AF318735 |
| Angiosperm | Rosaceae | *Prunus angustifolia* | AY177138 |
| Angiosperm | Rosaceae | *Prunus apetela* | AF411509 |
| Angiosperm | Rosaceae | *Prunus argentea* | AF318749 |
| Angiosperm | Rosaceae | *Prunus armeniaca* | AF318756 |
| Angiosperm | Rosaceae | *Prunus bifrons* | AF318757 |
| Angiosperm | Rosaceae | *Prunus bucharica* | AF318719 |
| Angiosperm | Rosaceae | *Prunus buergeriana* | AF143531 |
| Angiosperm | Rosaceae | *Prunus buergeriana* | AY052502 |
| Angiosperm | Rosaceae | *Prunus caroliniana* | AY055466 |
| Angiosperm | Rosaceae | *Prunus cerasifera* | AF318755 |
| Angiosperm | Rosaceae | *Prunus cerasoides* var. *campanulata* | AF411511 |
| Angiosperm | Rosaceae | *Prunus cerasus* | AF318729 |
| Angiosperm | Rosaceae | *Prunus davidiana* | DQ003550 |
| Angiosperm | Rosaceae | *Prunus davidiana* | DQ006281 |
| Angiosperm | Rosaceae | *Prunus dulcis* | AF318754 |
| Angiosperm | Rosaceae | *Prunus emarginata* | AF318717 |
| Angiosperm | Rosaceae | *Prunus fasciculata* | AF318752 |
| Angiosperm | Rosaceae | *Prunus ferganensis* | DQ006277 |
| Angiosperm | Rosaceae | *Prunus ferganensis* | DQ006280 |
| Angiosperm | Rosaceae | *Prunus fremontii* | AF318753 |
| Angiosperm | Rosaceae | *Prunus fruticosa* | AF318738 |
| Angiosperm | Rosaceae | *Prunus grayana* | AF411514 |
| Angiosperm | Rosaceae | *Prunus ilicifolia* subsp. *lyonii* | AF318743 |
| Angiosperm | Rosaceae | *Prunus incisa* | AF411508 |
| Angiosperm | Rosaceae | *Prunus incisa* var. *kinkiensis* | AF411512 |
| Angiosperm | Rosaceae | *Prunus kansuensis* | DQ003552 |
| Angiosperm | Rosaceae | *Prunus laurocerasus* | AF318724 |
| Angiosperm | Rosaceae | *Prunus leveilleana* | AY052503 |
| Angiosperm | Rosaceae | *Prunus leveilleana* | AY052511 |
| Angiosperm | Rosaceae | *Prunus lusitanica* | AY100698 |
| Angiosperm | Rosaceae | *Prunus maackii* | AY864831 |
| Angiosperm | Rosaceae | *Prunus mahaleb* | AF318747 |
| Angiosperm | Rosaceae | *Prunus mandshurica* | AF318714 |
| Angiosperm | Rosaceae | *Prunus maximowiczii* | AF143532 |
| Angiosperm | Rosaceae | *Prunus microcarpa* | AF492416 |
| Angiosperm | Rosaceae | *Prunus mira* | DQ003551 |
| Angiosperm | Rosaceae | *Prunus mume* | AF318728 |
| Angiosperm | Rosaceae | *Prunus mume* | EF523487 |
| Angiosperm | Rosaceae | *Prunus mume* | EF523488 |
| Angiosperm | Rosaceae | *Prunus mume* | EF523489 |
| Angiosperm | Rosaceae | *Prunus mume* | EF523490 |
| Angiosperm | Rosaceae | *Prunus mume* | EF523491 |
| Angiosperm | Rosaceae | *Prunus mume* | EF523492 |
| Angiosperm | Rosaceae | *Prunus mume* | EF523493 |
| Angiosperm | Rosaceae | *Prunus mume* | EF529435 |
| Angiosperm | Rosaceae | *Prunus mume* | EF529436 |
| Angiosperm | Rosaceae | *Prunus mume* | EU334746 |
| Angiosperm | Rosaceae | *Prunus munsoniana* | AF318736 |
| Angiosperm | Rosaceae | *Prunus padus* | AF143533 |
| Angiosperm | Rosaceae | *Prunus padus* | AF318726 |
| Angiosperm | Rosaceae | *Prunus persica* | AF143535 |
| Angiosperm | Rosaceae | *Prunus persica* | DQ003548 |
| Angiosperm | Rosaceae | *Prunus persica* | DQ003549 |
| Angiosperm | Rosaceae | *Prunus persica f. compressa* | DQ006278 |
| Angiosperm | Rosaceae | *Prunus petunnikowii* | AY177135 |
| Angiosperm | Rosaceae | *Prunus prostrata* | AF492415 |
| Angiosperm | Rosaceae | *Prunus pseudocerasus* | AF318731 |
| Angiosperm | Rosaceae | *Prunus salicina* | AF318725 |
| Angiosperm | Rosaceae | *Prunus serotina* | AY864830 |
| Angiosperm | Rosaceae | *Prunus serrulata* | AF318721 |
| Angiosperm | Rosaceae | *Prunus serrulata* var. *lannesiana* | AF411507 |
| Angiosperm | Rosaceae | *Prunus serrulata* var. *lannesiana* | AY052509 |
| Angiosperm | Rosaceae | *Prunus serrulata* var. *spontanea* | AY052508 |
| Angiosperm | Rosaceae | *Prunus sibirica* | AF318739 |
| Angiosperm | Rosaceae | *Prunus simonii* | AF318720 |
| Angiosperm | Rosaceae | *Prunus spinosa* | AF318730 |
| Angiosperm | Rosaceae | *Prunus spinulosa* | AF411513 |
| Angiosperm | Rosaceae | *Prunus subhirtella* var. *ascendens* | AY052505 |
| Angiosperm | Rosaceae | *Prunus takesimensis* | AF143534 |
| Angiosperm | Rosaceae | *Prunus takesimensis* | AF318718 |
| Angiosperm | Rosaceae | *Prunus tenella* | DQ006279 |
| Angiosperm | Rosaceae | *Prunus texana* | AY177139 |
| Angiosperm | Rosaceae | *Prunus tomentosa* | AF318746 |
| Angiosperm | Rosaceae | *Prunus virginiana* | AF318742 |
| Angiosperm | Rosaceae | *Prunus virginiana* | AY177136 |
| Angiosperm | Rosaceae | *Prunus x yedoensis* | AF117895 |
| Angiosperm | Rosaceae | *Rosa canina* | FM164423 |
| Angiosperm | Rosaceae | *Rosa canina* | FM164424 |
| Angiosperm | Rosaceae | *Rosa dumalis* subsp. *dumalis* | FM164949 |
| Angiosperm | Rosaceae | *Rosa dumalis* subsp. *dumalis* | FM164950 |
| Angiosperm | Rosaceae | *Rosa eglanteria* | FM164947 |
| Angiosperm | Rosaceae | *Rosa hybrid* | AB043833 |
| Angiosperm | Rosaceae | *Rosa hybrid* | AB043836 |
| Angiosperm | Rosaceae | *Rosa moschata* | AB043001 |
| Angiosperm | Rosaceae | *Rosa moschata* | AB043002 |
| Angiosperm | Rosaceae | *Rosa moschata* | AB043003 |
| Angiosperm | Rosaceae | *Rosa moschata* | AB043004 |
| Angiosperm | Rosaceae | *Rosa multiflora* | AY635026 |
| Angiosperm | Rosaceae | *Rosa phoenicia* | AB043826 |
| Angiosperm | Rosaceae | *Rosa phoenicia* | AB043827 |
| Angiosperm | Rosaceae | *Rosa phoenicia* | AB043828 |
| Angiosperm | Rosaceae | *Rosa virginiana* | DQ242528 |
| Angiosperm | Rosaceae | *Rosa woodsii* | DQ242529 |
| Angiosperm | Rosaceae | *Rubus caesius x Rubus idaeus* | AF362718 |
| Angiosperm | Rosaceae | *Rubus caesius x Rubus idaeus* | AF362719 |
| Angiosperm | Rosaceae | *Rubus corchorifolius* | DQ217770 |
| Angiosperm | Rosaceae | *Rubus idaeus* | AF055757 |
| Angiosperm | Rosaceae | *Rubus picticaulis* | AF362723 |
| Angiosperm | Rosaceae | *Rubus picticaulis* | AF362724 |
| Angiosperm | Rosaceae | *Rubus picticaulis* | AF362725 |
| Angiosperm | Rosaceae | *Rubus picticaulis* | AF362726 |
| Angiosperm | Rosaceae | *Sanguisorba annua* | AY635030 |
| Angiosperm | Rosaceae | *Sanguisorba annua* | AY635031 |
| Angiosperm | Rosaceae | *Sanguisorba canadensis* | AY635033 |
| Angiosperm | Rosaceae | *Sanguisorba canadensis* | AY635034 |
| Angiosperm | Rosaceae | *Sanguisorba hakusanensis* | AY635035 |
| Angiosperm | Rosaceae | *Sanguisorba minor* | AY635036 |
| Angiosperm | Rosaceae | *Sanguisorba minor* | AY635037 |
| Angiosperm | Rosaceae | *Sanguisorba minor* | AY635038 |
| Angiosperm | Rosaceae | *Sanguisorba minor* | AY635039 |
| Angiosperm | Rosaceae | *Sanguisorba officinalis* | AY635040 |
| Angiosperm | Rosaceae | *Sanguisorba officinalis* | AY635041 |
| Angiosperm | Rosaceae | *Sanguisorba stipulata* | AY635042 |
| Angiosperm | Rosaceae | *Sorbaria sorbifolia* | AF318758 |
| Angiosperm | Rosaceae | *Spiraea cantoniensis* | AF318722 |
| Angiosperm | Rubiaceae | *Coffea ambongensis* | DQ153539 |
| Angiosperm | Rubiaceae | *Coffea ambongensis* | DQ153540 |
| Angiosperm | Rubiaceae | *Coffea ankaranensis* | DQ153527 |
| Angiosperm | Rubiaceae | *Coffea arenesiana* | DQ153568 |
| Angiosperm | Rubiaceae | *Coffea augagneuri* | DQ153561 |
| Angiosperm | Rubiaceae | *Coffea bakossi* | DQ153599 |
| Angiosperm | Rubiaceae | *Coffea betamponensis* | DQ153543 |
| Angiosperm | Rubiaceae | *Coffea boinensis* | DQ153528 |
| Angiosperm | Rubiaceae | *Coffea boiviniana* | DQ153551 |
| Angiosperm | Rubiaceae | *Coffea boiviniana* | DQ153552 |
| Angiosperm | Rubiaceae | *Coffea boiviniana* | DQ153553 |
| Angiosperm | Rubiaceae | *Coffea brevipes* | DQ153591 |
| Angiosperm | Rubiaceae | *Coffea bridsoniae* | DQ153584 |
| Angiosperm | Rubiaceae | *Coffea bridsoniae* | DQ153586 |
| Angiosperm | Rubiaceae | *Coffea buxifolia* | DQ153570 |
| Angiosperm | Rubiaceae | *Coffea campaniensis* | DQ153601 |
| Angiosperm | Rubiaceae | *Coffea commersoniana* | DQ153560 |
| Angiosperm | Rubiaceae | *Coffea coursiana* | DQ153537 |
| Angiosperm | Rubiaceae | *Coffea decaryana* | DQ153556 |
| Angiosperm | Rubiaceae | *Coffea dubardii* | DQ153563 |
| Angiosperm | Rubiaceae | *Coffea grevei* | DQ153534 |
| Angiosperm | Rubiaceae | *Coffea homollei* | DQ153521 |
| Angiosperm | Rubiaceae | *Coffea humbertii* | DQ153565 |
| Angiosperm | Rubiaceae | *Coffea humblotiana* | DQ153531 |
| Angiosperm | Rubiaceae | *Coffea humilis* | DQ153611 |
| Angiosperm | Rubiaceae | *Coffea kapakata* | DQ153596 |
| Angiosperm | Rubiaceae | *Coffea kianjavatensis* | DQ153613 |
| Angiosperm | Rubiaceae | *Coffea kihansiensis* | DQ153583 |
| Angiosperm | Rubiaceae | *Coffea kimbozensis* | DQ153575 |
| Angiosperm | Rubiaceae | *Coffea kivuensis* | DQ153612 |
| Angiosperm | Rubiaceae | *Coffea lancifolia* | DQ153522 |
| Angiosperm | Rubiaceae | *Coffea littoralis* | DQ153569 |
| Angiosperm | Rubiaceae | *Coffea magnistipula* | DQ153640 |
| Angiosperm | Rubiaceae | *Coffea mangoroensis* | DQ153634 |
| Angiosperm | Rubiaceae | *Coffea manombensis* | DQ153573 |
| Angiosperm | Rubiaceae | *Coffea mauritiana* | DQ153600 |
| Angiosperm | Rubiaceae | *Coffea mayombensis* | DQ153592 |
| Angiosperm | Rubiaceae | *Coffea mcphersonii* | DQ153546 |
| Angiosperm | Rubiaceae | *Coffea mcphersonii* | DQ153547 |
| Angiosperm | Rubiaceae | *Coffea mcphersonii* | DQ153548 |
| Angiosperm | Rubiaceae | *Coffea millotii* | DQ153529 |
| Angiosperm | Rubiaceae | *Coffea montekupensis* | DQ153590 |
| Angiosperm | Rubiaceae | *Coffea montis-sacri* | DQ153557 |
| Angiosperm | Rubiaceae | *Coffea myrtifolia* | DQ153608 |
| Angiosperm | Rubiaceae | *Coffea pervilleana* | DQ153532 |
| Angiosperm | Rubiaceae | *Coffea pocsii* | DQ153581 |
| Angiosperm | Rubiaceae | *Coffea pocsii* | DQ153582 |
| Angiosperm | Rubiaceae | *Coffea pseudozanguebariae* | DQ153578 |
| Angiosperm | Rubiaceae | *Coffea racemosa* | DQ153595 |
| Angiosperm | Rubiaceae | *Coffea rakotonasoloi* | DQ153536 |
| Angiosperm | Rubiaceae | *Coffea ratsimamangae* | DQ153572 |
| Angiosperm | Rubiaceae | *Coffea resinosa* | DQ153555 |
| Angiosperm | Rubiaceae | *Coffea rhamnifolia* | DQ153589 |
| Angiosperm | Rubiaceae | *Coffea salvatrix* | DQ153622 |
| Angiosperm | Rubiaceae | *Coffea sambavensis* | DQ153538 |
| Angiosperm | Rubiaceae | *Coffea stenophylla* | DQ153597 |
| Angiosperm | Rubiaceae | *Coffea tetragona* | DQ153526 |
| Angiosperm | Rubiaceae | *Coffea togoensis* | DQ153607 |
| Angiosperm | Rubiaceae | *Coffea tsirananae* | DQ153571 |
| Angiosperm | Rubiaceae | *Coffea vatovavyensis* | DQ153530 |
| Angiosperm | Rubiaceae | *Galium aparine* | DQ006036 |
| Angiosperm | Rubiaceae | *Galium mollugo* | AM503885 |
| Angiosperm | Rubiaceae | *Gardenia thunbergia* | AJ224833 |
| Angiosperm | Rubiaceae | *Hedyotis pinifolia* | AY438316 |
| Angiosperm | Rubiaceae | *Knoxia manika* | AM267001 |
| Angiosperm | Rubiaceae | *Knoxia platycarpa* | AM267002 |
| Angiosperm | Rubiaceae | *Knoxia sumatrensis* | AM267003 |
| Angiosperm | Rubiaceae | *Morinda citrifolia* | AY762833 |
| Angiosperm | Rubiaceae | *Morinda citrifolia* | AY762840 |
| Angiosperm | Rubiaceae | *Morinda citrifolia* | AY762842 |
| Angiosperm | Rubiaceae | *Morinda parvifolia* | AY551327 |
| Angiosperm | Rubiaceae | *Morinda royoc* | AF333845 |
| Angiosperm | Rubiaceae | *Morinda umbellata* | AY514063 |
| Angiosperm | Rubiaceae | *Notopleura dukei* | AF071989 |
| Angiosperm | Rubiaceae | *Notopleura guadalupensis* | AF071992 |
| Angiosperm | Rubiaceae | *Notopleura uliginosa* | AF071991 |
| Angiosperm | Rubiaceae | *Oldenlandia corymbosa* | AY438323 |
| Angiosperm | Rubiaceae | *Oldenlandia diffusa* | AY438319 |
| Angiosperm | Rubiaceae | *Psychotria aubletiana* | AF072002 |
| Angiosperm | Rubiaceae | *Psychotria berteriana* | AF072003 |
| Angiosperm | Rubiaceae | *Psychotria borjensis* | AF072041 |
| Angiosperm | Rubiaceae | *Psychotria borucana* | AF072021 |
| Angiosperm | Rubiaceae | *Psychotria brachiata* | AF072001 |
| Angiosperm | Rubiaceae | *Psychotria brachybotrya* | AF072004 |
| Angiosperm | Rubiaceae | *Psychotria brasiliensis* | AF072053 |
| Angiosperm | Rubiaceae | *Psychotria bremekampiana* | AF072000 |
| Angiosperm | Rubiaceae | *Psychotria capitata* | AF072005 |
| Angiosperm | Rubiaceae | *Psychotria chagrensis* | AF072051 |
| Angiosperm | Rubiaceae | *Psychotria chiriquiensis* | AF071999 |
| Angiosperm | Rubiaceae | *Psychotria deflexa* | AF072006 |
| Angiosperm | Rubiaceae | *Psychotria furcata* | AF071996 |
| Angiosperm | Rubiaceae | *Psychotria hexandra* | AF034907 |
| Angiosperm | Rubiaceae | *Psychotria hobdyi* | AF034906 |
| Angiosperm | Rubiaceae | *Psychotria hombroniana* | AF072032 |
| Angiosperm | Rubiaceae | *Psychotria horizontalis* | AF072047 |
| Angiosperm | Rubiaceae | *Psychotria ipecacuanha* | AF072020 |
| Angiosperm | Rubiaceae | *Psychotria kirkii* | AF072038 |
| Angiosperm | Rubiaceae | *Psychotria limonensis* | AF072052 |
| Angiosperm | Rubiaceae | *Psychotria loniceroides* | AF072034 |
| Angiosperm | Rubiaceae | *Psychotria luzoniensis* | AF072037 |
| Angiosperm | Rubiaceae | *Psychotria macrophylla* | AF071990 |
| Angiosperm | Rubiaceae | *Psychotria mahonii* | AF072042 |
| Angiosperm | Rubiaceae | *Psychotria mapourioides* | AF072040 |
| Angiosperm | Rubiaceae | *Psychotria marginata* | AF072049 |
| Angiosperm | Rubiaceae | *Psychotria mariniana* | AF034904 |
| Angiosperm | Rubiaceae | *Psychotria mauiensis* | AF034905 |
| Angiosperm | Rubiaceae | *Psychotria micrantha* | AF072048 |
| Angiosperm | Rubiaceae | *Psychotria microdon* | AF072013 |
| Angiosperm | Rubiaceae | *Psychotria mucronata* | AF072045 |
| Angiosperm | Rubiaceae | *Psychotria muscosa* | AF071994 |
| Angiosperm | Rubiaceae | *Psychotria nervosa* | AF072046 |
| Angiosperm | Rubiaceae | *Psychotria pittieri* | AF071998 |
| Angiosperm | Rubiaceae | *Psychotria poeppigiana* | AF071993 |
| Angiosperm | Rubiaceae | *Psychotria pubescens* | AF071997 |
| Angiosperm | Rubiaceae | *Psychotria racemosa* | AF071995 |
| Angiosperm | Rubiaceae | *Psychotria rhombocarpa* | AF072031 |
| Angiosperm | Rubiaceae | *Psychotria rubra* | AF072035 |
| Angiosperm | Rubiaceae | *Psychotria simmondsiana* | AF072022 |
| Angiosperm | Rubiaceae | *Psychotria submontana* | AF072023 |
| Angiosperm | Rubiaceae | *Psychotria tahitiensis* | AF072029 |
| Angiosperm | Rubiaceae | *Psychotria tenuifolia* | AF072050 |
| Angiosperm | Rubiaceae | *Psychotria trichocalyx* | AF072030 |
| Angiosperm | Rubiaceae | *Psychotria urceolata* | AF072007 |
| Angiosperm | Rubiaceae | *Rubia tinctorum* | DQ358885 |
| Angiosperm | Rubiaceae | *Uncaria africana* | AJ414545 |
| Angiosperm | Rubiaceae | *Uncaria guianensis* | AJ414546 |
| Angiosperm | Rubiaceae | *Uncaria rhynchophylla* | AJ346900 |
| Angiosperm | Rutaceae | *Acronychia baeuerlenii* | AY588596 |
| Angiosperm | Rutaceae | *Acronychia imperforata* | AY588597 |
| Angiosperm | Rutaceae | *Acronychia oblongifolia* | AY588598 |
| Angiosperm | Rutaceae | *Acronychia pauciflora* | AY588593 |
| Angiosperm | Rutaceae | *Acronychia pubescens* | AY588594 |
| Angiosperm | Rutaceae | *Acronychia suberosa* | AY588595 |
| Angiosperm | Rutaceae | *Acronychia wilcoxiana* | AY588592 |
| Angiosperm | Rutaceae | *Citrus medica* | AM260544 |
| Angiosperm | Rutaceae | *Citrus medica* var. *sarcodactylis* | AM260543 |
| Angiosperm | Rutaceae | *Citrus reticulata* | AM398230 |
| Angiosperm | Rutaceae | *Glycosmis pentaphylla* | FJ434151 |
| Angiosperm | Rutaceae | *Murraya koenigii* | AJ879084 |
| Angiosperm | Rutaceae | *Murraya paniculata* | AJ879085 |
| Angiosperm | Rutaceae | *Zanthoxylum schinifolium* | DQ225861 |
| Angiosperm | Salicaceae | *Populus alba* | AJ006437 |
| Angiosperm | Salicaceae | *Populus deltoides* | AJ006438 |
| Angiosperm | Salicaceae | *Populus deltoides* | EF060365 |
| Angiosperm | Salicaceae | *Populus lasiocarpa* | AJ006439 |
| Angiosperm | Salicaceae | *Populus trichocarpa* | AJ006440 |
| Angiosperm | Santalaceae | *Santalum album* | EF569317 |
| Angiosperm | Santalaceae | *Santalum album* | EF569318 |
| Angiosperm | Santalaceae | *Santalum album* | EF569319 |
| Angiosperm | Santalaceae | *Santalum album* | EF569320 |
| Angiosperm | Santalaceae | *Santalum austrocaledonicum* var. *austrocaledonicum* | EF569331 |
| Angiosperm | Santalaceae | *Santalum boninense* | EF569351 |
| Angiosperm | Santalaceae | *Santalum ellipticum* var. *ellipticum* | EF569362 |
| Angiosperm | Santalaceae | *Santalum insulare* var. *raivavense* | EF569348 |
| Angiosperm | Santalaceae | *Santalum leptocladum* | EU095520 |
| Angiosperm | Santalaceae | *Santalum macgregorii* | EF569322 |
| Angiosperm | Santalaceae | *Santalum murrayanum* | EF569310 |
| Angiosperm | Santalaceae | *Santalum obtusifolium* | EF569313 |
| Angiosperm | Santalaceae | *Santalum paniculatum* var. *pilgeri* | EF569372 |
| Angiosperm | Santalaceae | *Santalum yasi* | EF569336 |
| Angiosperm | Santalaceae | *Thesium impeditum* | AF291908 |
| Angiosperm | Sapindaceae | *Dimocarpus confinis* | EF532336 |
| Angiosperm | Sapindaceae | *Dimocarpus longan* | EF532329 |
| Angiosperm | Sapindaceae | *Dimocarpus longan* | EF532330 |
| Angiosperm | Sapindaceae | *Dimocarpus longan* | EF532331 |
| Angiosperm | Sapindaceae | *Dimocarpus longan* | EF532332 |
| Angiosperm | Sapindaceae | *Dimocarpus longan* | EF532333 |
| Angiosperm | Sapindaceae | *Dimocarpus longan* | EF532334 |
| Angiosperm | Sapindaceae | *Dimocarpus longan* | EF532335 |
| Angiosperm | Sapindaceae | *Dimocarpus longan* | EF532337 |
| Angiosperm | Sapindaceae | *Dimocarpus longan* | EF532340 |
| Angiosperm | Sapindaceae | *Dimocarpus longan* | EU243565 |
| Angiosperm | Sapindaceae | *Dodonaea viscosa* | AY864896 |
| Angiosperm | Sapindaceae | *Eurycorymbus cavaleriei* | EU243563 |
| Angiosperm | Sapindaceae | *Litchi chinensis* | EU243564 |
| Angiosperm | Sapindaceae | *Sapindus delavayi* | AY207570 |
| Angiosperm | Saururaceae | *Houttuynia cordata* | EF108407 |
| Angiosperm | Saururaceae | *Saururus chinensis* | AF215921 |
| Angiosperm | Saxifragaceae | *Dichroa febrifuga* | FJ980367 |
| Angiosperm | Saxifragaceae | *Hydrangea arborescens* | DQ006012 |
| Angiosperm | Saxifragaceae | *Hydrangea chinensis* | AB377209 |
| Angiosperm | Saxifragaceae | *Hydrangea chinensis* | AB377210 |
| Angiosperm | Saxifragaceae | *Hydrangea chinensis* | AB377211 |
| Angiosperm | Saxifragaceae | *Hydrangea grosseserrata* | AB377202 |
| Angiosperm | Saxifragaceae | *Hydrangea grosseserrata* | AB377203 |
| Angiosperm | Saxifragaceae | *Hydrangea liukiuensis* | AB377194 |
| Angiosperm | Saxifragaceae | *Hydrangea liukiuensis* | AB377195 |
| Angiosperm | Saxifragaceae | *Hydrangea liukiuensis* | AB377196 |
| Angiosperm | Saxifragaceae | *Hydrangea liukiuensis* | AB377197 |
| Angiosperm | Saxifragaceae | *Philadelphus incanus* | DQ248970 |
| Angiosperm | Saxifragaceae | *Rodgersia podophylla* | AB248848 |
| Angiosperm | Saxifragaceae | *Saxifraga adscendens* | EF028688 |
| Angiosperm | Saxifragaceae | *Saxifraga afghanica* | EU158853 |
| Angiosperm | Saxifragaceae | *Saxifraga aizoides* | AF504547 |
| Angiosperm | Saxifragaceae | *Saxifraga androsacea* | AF261159 |
| Angiosperm | Saxifragaceae | *Saxifraga aphylla* | AF261160 |
| Angiosperm | Saxifragaceae | *Saxifraga aquatica* | AF261161 |
| Angiosperm | Saxifragaceae | *Saxifraga arachnoidea* | AF261162 |
| Angiosperm | Saxifragaceae | *Saxifraga atrata* | EU158852 |
| Angiosperm | Saxifragaceae | *Saxifraga balfourii* | EU158838 |
| Angiosperm | Saxifragaceae | *Saxifraga biternata* | AF261163 |
| Angiosperm | Saxifragaceae | *Saxifraga blepharophylla* | AF504545 |
| Angiosperm | Saxifragaceae | *Saxifraga brachypoda* | EU158855 |
| Angiosperm | Saxifragaceae | *Saxifraga bulbifera* | AF261166 |
| Angiosperm | Saxifragaceae | *Saxifraga canaliculata* | AF261167 |
| Angiosperm | Saxifragaceae | *Saxifraga carpetana* | AF261168 |
| Angiosperm | Saxifragaceae | *Saxifraga cebennensis* | AF261169 |
| Angiosperm | Saxifragaceae | *Saxifraga cernua* | EU158861 |
| Angiosperm | Saxifragaceae | *Saxifraga cespitosa* | AF261170 |
| Angiosperm | Saxifragaceae | *Saxifraga cintrana* | AF261171 |
| Angiosperm | Saxifragaceae | *Saxifraga consanguinea* | EU158837 |
| Angiosperm | Saxifragaceae | *Saxifraga cossoniana* | AF261172 |
| Angiosperm | Saxifragaceae | *Saxifraga depressa* | AF261173 |
| Angiosperm | Saxifragaceae | *Saxifraga dichotoma* | AF261174 |
| Angiosperm | Saxifragaceae | *Saxifraga dingqingensis* | EU158857 |
| Angiosperm | Saxifragaceae | *Saxifraga egregia* var. *eciliata* | EU158842 |
| Angiosperm | Saxifragaceae | *Saxifraga facchini* | AF261175 |
| Angiosperm | Saxifragaceae | *Saxifraga fragilis* | AF261176 |
| Angiosperm | Saxifragaceae | *Saxifraga gemmigera* var. *gemmuligera* | EU158856 |
| Angiosperm | Saxifragaceae | *Saxifraga gemmipara* | EU158859 |
| Angiosperm | Saxifragaceae | *Saxifraga gemmulosa* | AF261177 |
| Angiosperm | Saxifragaceae | *Saxifraga genesiana* | AF261178 |
| Angiosperm | Saxifragaceae | *Saxifraga geranioides* | AF261192 |
| Angiosperm | Saxifragaceae | *Saxifraga graeca* | AF261179 |
| Angiosperm | Saxifragaceae | *Saxifraga granulata* | AF482692 |
| Angiosperm | Saxifragaceae | *Saxifraga haenseleri* | AF261180 |
| Angiosperm | Saxifragaceae | *Saxifraga hariotii* | AF261181 |
| Angiosperm | Saxifragaceae | *Saxifraga hederacea* | AF261182 |
| Angiosperm | Saxifragaceae | *Saxifraga hookeri* | EU158840 |
| Angiosperm | Saxifragaceae | *Saxifraga insolens* | EU158841 |
| Angiosperm | Saxifragaceae | *Saxifraga intricata* | AJ133030 |
| Angiosperm | Saxifragaceae | *Saxifraga isophylla* | EU158848 |
| Angiosperm | Saxifragaceae | *Saxifraga kingiana* | EU158851 |
| Angiosperm | Saxifragaceae | *Saxifraga latepetiolata* | AF261183 |
| Angiosperm | Saxifragaceae | *Saxifraga melanocentra* | EU158846 |
| Angiosperm | Saxifragaceae | *Saxifraga mertensiana* | AY231367 |
| Angiosperm | Saxifragaceae | *Saxifraga moncayensis* | AJ133028 |
| Angiosperm | Saxifragaceae | *Saxifraga moschata* | AF261184 |
| Angiosperm | Saxifragaceae | *Saxifraga nangxianensis* | EU158850 |
| Angiosperm | Saxifragaceae | *Saxifraga nevadensis* | AF261185 |
| Angiosperm | Saxifragaceae | *Saxifraga oppositifolia* | EF687810 |
| Angiosperm | Saxifragaceae | *Saxifraga pallida* | EU158863 |
| Angiosperm | Saxifragaceae | *Saxifraga pentadactylis* | AJ133031 |
| Angiosperm | Saxifragaceae | *Saxifraga pentadactylis* subsp. *willkommiana* | AY354307 |
| Angiosperm | Saxifragaceae | *Saxifraga pentadactylis* subsp. *willkommiana* | AY354308 |
| Angiosperm | Saxifragaceae | *Saxifraga peplidifolia* | EU158843 |
| Angiosperm | Saxifragaceae | *Saxifraga praetermissa* | AF261186 |
| Angiosperm | Saxifragaceae | *Saxifraga pseudohirculus* | EU158844 |
| Angiosperm | Saxifragaceae | *Saxifraga pulvinaria* | EU158860 |
| Angiosperm | Saxifragaceae | *Saxifraga punctulata* | EU158854 |
| Angiosperm | Saxifragaceae | *Saxifraga rosacea* | AF261190 |
| Angiosperm | Saxifragaceae | *Saxifraga sanguinea* | EU158849 |
| Angiosperm | Saxifragaceae | *Saxifraga sedoides* | AF261191 |
| Angiosperm | Saxifragaceae | *Saxifraga sinomontana* | EU158834 |
| Angiosperm | Saxifragaceae | *Saxifraga strigosa* | EF369514 |
| Angiosperm | Saxifragaceae | *Saxifraga tangutica* | EU158858 |
| Angiosperm | Saxifragaceae | *Saxifraga tatsienluensis* | EU158845 |
| Angiosperm | Saxifragaceae | *Saxifraga trabutiana* | AF482693 |
| Angiosperm | Saxifragaceae | *Saxifraga tridactylites* | EF028686 |
| Angiosperm | Saxifragaceae | *Saxifraga tridactylites* | EF028687 |
| Angiosperm | Saxifragaceae | *Saxifraga unguiculata* | EU158839 |
| Angiosperm | Saxifragaceae | *Saxifraga vayredana* | AJ133029 |
| Angiosperm | Saxifragaceae | *Saxifraga wallichiana* | EU158847 |
| Angiosperm | Saxifragaceae | *Saxifraga xiaozhongdianensis* | EU158835 |
| Angiosperm | Saxifragaceae | *Saxifraga zhidoensis* | EU158862 |
| Angiosperm | Scrophulariaceae | *Antirrhinum breweri* | AF513901 |
| Angiosperm | Scrophulariaceae | *Antirrhinum cornutum* | AF513905 |
| Angiosperm | Scrophulariaceae | *Antirrhinum costatum* | AF513893 |
| Angiosperm | Scrophulariaceae | *Antirrhinum coulterianum* | AF513890 |
| Angiosperm | Scrophulariaceae | *Antirrhinum cyathiferum* | AF513884 |
| Angiosperm | Scrophulariaceae | *Antirrhinum filipes* | AF513896 |
| Angiosperm | Scrophulariaceae | *Antirrhinum hispanicum* subsp. *mollissimum* | AF513886 |
| Angiosperm | Scrophulariaceae | *Antirrhinum kelloggii* | AF513904 |
| Angiosperm | Scrophulariaceae | *Antirrhinum leptaleum* | AF513906 |
| Angiosperm | Scrophulariaceae | *Antirrhinum meonanthum* | AF513887 |
| Angiosperm | Scrophulariaceae | *Antirrhinum multiflorum* | AF513897 |
| Angiosperm | Scrophulariaceae | *Antirrhinum nuttallianum* | AF513895 |
| Angiosperm | Scrophulariaceae | *Antirrhinum ovatum* | AF513899 |
| Angiosperm | Scrophulariaceae | *Antirrhinum subcordatum* | AF513902 |
| Angiosperm | Scrophulariaceae | *Antirrhinum vexillocalyculatum* subsp. *intermedium* | AF513907 |
| Angiosperm | Scrophulariaceae | *Antirrhinum vexillocalyculatum* subsp. *vexillocalyculatum* | AF513900 |
| Angiosperm | Scrophulariaceae | *Antirrhinum virga* | AF513898 |
| Angiosperm | Scrophulariaceae | *Antirrhinum watsonii* | AF513894 |
| Angiosperm | Scrophulariaceae | *Lagotis angustibracteata* | AF313028 |
| Angiosperm | Scrophulariaceae | *Misopates orontium* | AF513889 |
| Angiosperm | Scrophulariaceae | *Pedicularis attollens* | EF103743 |
| Angiosperm | Scrophulariaceae | *Pedicularis verticillata* | AY596818 |
| Angiosperm | Scrophulariaceae | *Picrorhiza kurrooa* | AF509813 |
| Angiosperm | Scrophulariaceae | *Rehmannia glutinosa* | EU266022 |
| Angiosperm | Scrophulariaceae | *Rehmannia glutinosa* | EU266023 |
| Angiosperm | Scrophulariaceae | *Rehmannia glutinosa* | EU266024 |
| Angiosperm | Scrophulariaceae | *Rehmannia glutinosa* | EU266025 |
| Angiosperm | Scrophulariaceae | *Rehmannia glutinosa* | EU787018 |
| Angiosperm | Scrophulariaceae | *Rehmannia glutinosa* | EU810383 |
| Angiosperm | Scrophulariaceae | *Rehmannia glutinosa* | EU810385 |
| Angiosperm | Scrophulariaceae | *Rehmannia glutinosa* | EU810386 |
| Angiosperm | Scrophulariaceae | *Rehmannia henryi* | DQ272447 |
| Angiosperm | Scrophulariaceae | *Rehmannia piasezkii* | DQ069316 |
| Angiosperm | Scrophulariaceae | *Rehmannia solanifolia* | DQ069314 |
| Angiosperm | Scrophulariaceae | *Scoparia dulcis* | AY963776 |
| Angiosperm | Scrophulariaceae | *Scrophularia canina* | AM503881 |
| Angiosperm | Scrophulariaceae | *Veronicastrum liukiuense* | AF509815 |
| Angiosperm | Scrophulariaceae | *Veronicastrum stenostachyum* | AF313031 |
| Angiosperm | Simaroubaceae | *Brucea javanica* | AY510155 |
| Angiosperm | Solanaceae | *Capsicum eximium* | AY665841 |
| Angiosperm | Solanaceae | *Capsicum lycianthoides* | DQ314158 |
| Angiosperm | Solanaceae | *Cestrum acutifolium* | DQ508655 |
| Angiosperm | Solanaceae | *Cestrum aurantiacum* | DQ508656 |
| Angiosperm | Solanaceae | *Cestrum aurantiacum* var. *macrocalyx* | DQ508657 |
| Angiosperm | Solanaceae | *Cestrum chiriquianum* | DQ508658 |
| Angiosperm | Solanaceae | *Cestrum elegans* | AJ492459 |
| Angiosperm | Solanaceae | *Cestrum endlicheri* | DQ508660 |
| Angiosperm | Solanaceae | *Cestrum fasciculatum* | DQ508661 |
| Angiosperm | Solanaceae | *Cestrum fragile* | DQ508662 |
| Angiosperm | Solanaceae | *Cestrum fulvescens* | DQ508663 |
| Angiosperm | Solanaceae | *Cestrum glanduliferum* | DQ508664 |
| Angiosperm | Solanaceae | *Cestrum guatemalense* | DQ508665 |
| Angiosperm | Solanaceae | *Cestrum inclusum* | DQ508666 |
| Angiosperm | Solanaceae | *Cestrum irazuense* | DQ508667 |
| Angiosperm | Solanaceae | *Cestrum laxum* | DQ508668 |
| Angiosperm | Solanaceae | *Cestrum miradorense* | DQ508671 |
| Angiosperm | Solanaceae | *Cestrum oblongifolium* | DQ508674 |
| Angiosperm | Solanaceae | *Cestrum pacayense* | DQ508675 |
| Angiosperm | Solanaceae | *Cestrum poasanum* | DQ508676 |
| Angiosperm | Solanaceae | *Cestrum roseum* | DQ508678 |
| Angiosperm | Solanaceae | *Cestrum sphaerocarpum* | DQ508679 |
| Angiosperm | Solanaceae | *Cestrum thyrsoideum* | DQ508681 |
| Angiosperm | Solanaceae | *Cestrum virgaurea* | DQ508685 |
| Angiosperm | Solanaceae | *Datura metel* | EF590767 |
| Angiosperm | Solanaceae | *Datura stramonium* | DQ006041 |
| Angiosperm | Solanaceae | *Lycianthes amatitlanensis* | AY665842 |
| Angiosperm | Solanaceae | *Lycianthes inaequilatera* | DQ314159 |
| Angiosperm | Solanaceae | *Lycium ameghinoi* | DQ124618 |
| Angiosperm | Solanaceae | *Lycium americanum* | DQ124619 |
| Angiosperm | Solanaceae | *Lycium berlandieri* | AF238989 |
| Angiosperm | Solanaceae | *Lycium californicum* | AF238993 |
| Angiosperm | Solanaceae | *Lycium californicum* | DQ124636 |
| Angiosperm | Solanaceae | *Lycium californicum* | DQ124639 |
| Angiosperm | Solanaceae | *Lycium californicum* | DQ124644 |
| Angiosperm | Solanaceae | *Lycium californicum* | DQ124647 |
| Angiosperm | Solanaceae | *Lycium californicum* | DQ124650 |
| Angiosperm | Solanaceae | *Lycium californicum* | DQ124651 |
| Angiosperm | Solanaceae | *Lycium californicum* | DQ124653 |
| Angiosperm | Solanaceae | *Lycium carolinianum* var. *quadrifidum* | DQ124622 |
| Angiosperm | Solanaceae | *Lycium cestroides* | DQ124623 |
| Angiosperm | Solanaceae | *Lycium cooperi* | AF238984 |
| Angiosperm | Solanaceae | *Lycium infaustum* | DQ124627 |
| Angiosperm | Solanaceae | *Lycium macrodon* | AF238983 |
| Angiosperm | Solanaceae | *Lycium macrodon* | DQ124628 |
| Angiosperm | Solanaceae | *Lycium morongii* | DQ124629 |
| Angiosperm | Solanaceae | *Lycium nodosum* | DQ124630 |
| Angiosperm | Solanaceae | *Lycium pallidum* | AF238986 |
| Angiosperm | Solanaceae | *Lycium pallidum* | DQ124631 |
| Angiosperm | Solanaceae | *Lycium parishii* | AF238990 |
| Angiosperm | Solanaceae | *Lycium parishii* | DQ124632 |
| Angiosperm | Solanaceae | *Lycium puberulum* | AF238985 |
| Angiosperm | Solanaceae | *Lycium shockleyi* | AF238987 |
| Angiosperm | Solanaceae | *Lycium tenuispinosum* | DQ124633 |
| Angiosperm | Solanaceae | *Lycium torreyi* | DQ124634 |
| Angiosperm | Solanaceae | *Lycium vimineum* | DQ124635 |
| Angiosperm | Solanaceae | *Nicandra physalodes* | DQ314155 |
| Angiosperm | Solanaceae | *Physalis acutifolia* | AY665876 |
| Angiosperm | Solanaceae | *Physalis alkekengi* | AM503883 |
| Angiosperm | Solanaceae | *Physalis alkekengi* | AM503884 |
| Angiosperm | Solanaceae | *Physalis alkekengi* | AY665849 |
| Angiosperm | Solanaceae | *Physalis alkekengi* | AY665850 |
| Angiosperm | Solanaceae | *Physalis angulata* | AY665875 |
| Angiosperm | Solanaceae | *Physalis angustifolia* | AY665878 |
| Angiosperm | Solanaceae | *Physalis angustiphysa* | AY665879 |
| Angiosperm | Solanaceae | *Physalis arborescens* | AY665866 |
| Angiosperm | Solanaceae | *Physalis arborescens* | AY665867 |
| Angiosperm | Solanaceae | *Physalis campanulata* | AY665882 |
| Angiosperm | Solanaceae | *Physalis caudella* | AY665891 |
| Angiosperm | Solanaceae | *Physalis chenopodiifolia* | AY665883 |
| Angiosperm | Solanaceae | *Physalis cordata* | AY665886 |
| Angiosperm | Solanaceae | *Physalis crassifolia* | AY665889 |
| Angiosperm | Solanaceae | *Physalis crassifolia* | AY665890 |
| Angiosperm | Solanaceae | *Physalis glutinosa* | AY665892 |
| Angiosperm | Solanaceae | *Physalis greenmanii* | AY665893 |
| Angiosperm | Solanaceae | *Physalis hederifolia* | AY665894 |
| Angiosperm | Solanaceae | *Physalis hederifolia* var. *puberula* | AY665874 |
| Angiosperm | Solanaceae | *Physalis heterophylla* | AY665907 |
| Angiosperm | Solanaceae | *Physalis hintonii* | AY665895 |
| Angiosperm | Solanaceae | *Physalis hintonii* | AY665896 |
| Angiosperm | Solanaceae | *Physalis ignota* | AY665897 |
| Angiosperm | Solanaceae | *Physalis lagascae* | AY665898 |
| Angiosperm | Solanaceae | *Physalis lassa* | AY665900 |
| Angiosperm | Solanaceae | *Physalis melanocystis* | AY665865 |
| Angiosperm | Solanaceae | *Physalis microcarpa* | AY665903 |
| Angiosperm | Solanaceae | *Physalis microphysa* | AY665859 |
| Angiosperm | Solanaceae | *Physalis minima* | AY665904 |
| Angiosperm | Solanaceae | *Physalis minimaculata* | AY665905 |
| Angiosperm | Solanaceae | *Physalis minimaculata* | AY665906 |
| Angiosperm | Solanaceae | *Physalis mollis* | AY665908 |
| Angiosperm | Solanaceae | *Physalis nicandroides* | AY665912 |
| Angiosperm | Solanaceae | *Physalis patula* | AY665913 |
| Angiosperm | Solanaceae | *Physalis philadelphica* | AY665871 |
| Angiosperm | Solanaceae | *Physalis pruinosa* | AY665915 |
| Angiosperm | Solanaceae | *Physalis pubescens* | AY665917 |
| Angiosperm | Solanaceae | *Physalis pumila* | AY665909 |
| Angiosperm | Solanaceae | *Physalis sordida* | AY665869 |
| Angiosperm | Solanaceae | *Physalis viscosa* | AY665870 |
| Angiosperm | Solanaceae | *Physalis walteri* | AY665918 |
| Angiosperm | Solanaceae | *Solanum aculeastrum* | DQ364755 |
| Angiosperm | Solanaceae | *Solanum americanum* | EF108406 |
| Angiosperm | Solanaceae | *Solanum cataphractum* | DQ364717 |
| Angiosperm | Solanaceae | *Solanum cinereum* | DQ364756 |
| Angiosperm | Solanaceae | *Solanum lycopersicoides* | AJ300212 |
| Angiosperm | Solanaceae | *Solanum lycopersicum* | EU760390 |
| Angiosperm | Solanaceae | *Solanum neorickii* | AJ300207 |
| Angiosperm | Solanaceae | *Solanum peruvianum* | AJ300210 |
| Angiosperm | Solanaceae | *Solanum pimpinellifolium* | AJ300196 |
| Angiosperm | Solanaceae | *Solanum stupefactum* | DQ364757 |
| Angiosperm | Stachyuraceae | *Stachyurus chinensis* | DQ307102 |
| Angiosperm | Stachyuraceae | *Stachyurus cordatulus* | DQ307114 |
| Angiosperm | Stachyuraceae | *Stachyurus himalaicus* | DQ307104 |
| Angiosperm | Stachyuraceae | *Stachyurus salicifolius* var. *lancicifolius* | DQ307106 |
| Angiosperm | Stachyuraceae | *Stachyurus salicifolius* var. *lancicifolius* | DQ307107 |
| Angiosperm | Stachyuraceae | *Stachyurus salicifolius* var. *salicifolius* | DQ307103 |
| Angiosperm | Sterculiaceae | *Sterculia lanceolata* | AF460184 |
| Angiosperm | Sterculiaceae | *Sterculia nobilis* | AF460183 |
| Angiosperm | Styracaceae | *Styrax japonicus* | AB114900 |
| Angiosperm | Tamaricaceae | *Tamarix arceuthoides* | AY452028 |
| Angiosperm | Tamaricaceae | *Tamarix chinensis* | AY207484 |
| Angiosperm | Tamaricaceae | *Tamarix elongata* | AY207483 |
| Angiosperm | Tamaricaceae | *Tamarix gansuensis* | AY452022 |
| Angiosperm | Tamaricaceae | *Tamarix gracilis* | AY452030 |
| Angiosperm | Tamaricaceae | *Tamarix hispida* | AY207482 |
| Angiosperm | Tamaricaceae | *Tamarix hispida* | AY428797 |
| Angiosperm | Tamaricaceae | *Tamarix hohenackeri* | AY452023 |
| Angiosperm | Tamaricaceae | *Tamarix karelinii* | AY452029 |
| Angiosperm | Tamaricaceae | *Tamarix laxa* | AY453692 |
| Angiosperm | Tamaricaceae | *Tamarix leptostachys* | AY207489 |
| Angiosperm | Tamaricaceae | *Tamarix leptostachys* | AY442318 |
| Angiosperm | Tamaricaceae | *Tamarix parviflora* | AY452026 |
| Angiosperm | Tamaricaceae | *Tamarix ramosissima* | AY207481 |
| Angiosperm | Tamaricaceae | *Tamarix sachensis* | AY452027 |
| Angiosperm | Tamaricaceae | *Tamarix taklamakanensis* | AY452021 |
| Angiosperm | Theaceae | *Camellia brachygyna* | EF646287 |
| Angiosperm | Theaceae | *Camellia compressa* | EF646284 |
| Angiosperm | Theaceae | *Camellia fascicularis* | AF315485 |
| Angiosperm | Theaceae | *Camellia flavida* | AF315480 |
| Angiosperm | Theaceae | *Camellia glabriperulata* | EF649683 |
| Angiosperm | Theaceae | *Camellia grandis* | AF315488 |
| Angiosperm | Theaceae | *Camellia japonica* | AY697417 |
| Angiosperm | Theaceae | *Camellia japonica* | AY697418 |
| Angiosperm | Theaceae | *Camellia japonica* | AY701854 |
| Angiosperm | Theaceae | *Camellia japonica* | AY701855 |
| Angiosperm | Theaceae | *Camellia japonica* | AY701856 |
| Angiosperm | Theaceae | *Camellia japonica* | AY701857 |
| Angiosperm | Theaceae | *Camellia japonica* | EF649690 |
| Angiosperm | Theaceae | *Camellia liberistamina* | EF649692 |
| Angiosperm | Theaceae | *Camellia liberofilamenta* | AF315490 |
| Angiosperm | Theaceae | *Camellia limonia* | AF315479 |
| Angiosperm | Theaceae | *Camellia longruiensis* | AF315478 |
| Angiosperm | Theaceae | *Camellia magniflora* | EF649684 |
| Angiosperm | Theaceae | *Camellia mongshanica* | EF649687 |
| Angiosperm | Theaceae | *Camellia multipetala* | AF315471 |
| Angiosperm | Theaceae | *Camellia nitidissima* | AY096013 |
| Angiosperm | Theaceae | *Camellia oviformis* | EF646292 |
| Angiosperm | Theaceae | *Camellia parvipetala* | AF315482 |
| Angiosperm | Theaceae | *Camellia phellocapsa* | EF649689 |
| Angiosperm | Theaceae | *Camellia pingguoensis* | AF315475 |
| Angiosperm | Theaceae | *Camellia pitardii* var. *pitardii* | EF646288 |
| Angiosperm | Theaceae | *Camellia pitardii* var. *yunnanica* | EF646289 |
| Angiosperm | Theaceae | *Camellia ptilosperma* | AF315491 |
| Angiosperm | Theaceae | *Camellia reticulata* | EF639856 |
| Angiosperm | Theaceae | *Camellia semiserrata* | EF649688 |
| Angiosperm | Theaceae | *Camellia semiserrata f. albiflora* | EF649691 |
| Angiosperm | Theaceae | *Camellia sinensis* | AF315492 |
| Angiosperm | Theaceae | *Camellia sinensis* | AY096014 |
| Angiosperm | Theaceae | *Camellia tenuivalvis* | EF646296 |
| Angiosperm | Theaceae | *Camellia terminalis* | AF315476 |
| Angiosperm | Theaceae | *Camellia villosa* | EF646283 |
| Angiosperm | Theaceae | *Camellia xiashiensis* | AF315493 |
| Angiosperm | Theaceae | *Camellia yunnanensis* | AF456256 |
| Angiosperm | Theaceae | *Eurya nitida* | AY096026 |
| Angiosperm | Thymelaeaceae | *Aquilaria sinensis* | EF645833 |
| Angiosperm | Thymelaeaceae | *Aquilaria sinensis* | EF645834 |
| Angiosperm | Thymelaeaceae | *Aquilaria yunnanensis* | EF645835 |
| Angiosperm | Thymelaeaceae | *Daphne mezereum* | AJ744931 |
| Angiosperm | Tiliaceae | *Tilia miqueliana* | DQ120724 |
| Angiosperm | Tiliaceae | *Tilia platyphyllos* | AF250292 |
| Angiosperm | Tiliaceae | *Tilia tomentosa* | AF250023 |
| Angiosperm | Trapaceae | *Trapa acornis* | AY315461 |
| Angiosperm | Trapaceae | *Trapa bicornis* | AY315469 |
| Angiosperm | Trapaceae | *Trapa bicornis* | AY315470 |
| Angiosperm | Trapaceae | *Trapa bispinosa* | AY315465 |
| Angiosperm | Trapaceae | *Trapa bispinosa* | AY315468 |
| Angiosperm | Trapaceae | *Trapa incisa* | AY315462 |
| Angiosperm | Trapaceae | *Trapa maximowiczii* | AY035757 |
| Angiosperm | Trilliaceae | *Paris axialis* | AY192537 |
| Angiosperm | Trilliaceae | *Paris bashanensis* | DQ486015 |
| Angiosperm | Trilliaceae | *Paris cronquistii* | AY192025 |
| Angiosperm | Trilliaceae | *Paris fargesii* | AY192536 |
| Angiosperm | Trilliaceae | *Paris incompleta* | AY974172 |
| Angiosperm | Trilliaceae | *Paris quadrifolia* | AY974174 |
| Angiosperm | Trilliaceae | *Paris thibetica* var. *apetala* | DQ486016 |
| Angiosperm | Tropaeolaceae | *Tropaeolum majus* | AF254020 |
| Angiosperm | Ulmaceae | *Celtis africana* | AY702559 |
| Angiosperm | Ulmaceae | *Trema micrantha* | AY635566 |
| Angiosperm | Ulmaceae | *Trema micrantha* | AY635568 |
| Angiosperm | Ulmaceae | *Trema micrantha* | AY635569 |
| Angiosperm | Ulmaceae | *Trema micrantha* | AY635570 |
| Angiosperm | Ulmaceae | *Ulmus minor* subsp. *canescens* | AJ622835 |
| Angiosperm | Ulmaceae | *Ulmus minor* subsp. *canescens* | AJ622836 |
| Angiosperm | Umbelliferae | *Changium smyrnioides* | DQ517340 |
| Angiosperm | Umbelliferae | *Glehnia littoralis* | EU164928 |
| Angiosperm | Urticaceae | *Boehmeria clidemioides* | EU003920 |
| Angiosperm | Urticaceae | *Boehmeria clidemioides* | EU003922 |
| Angiosperm | Urticaceae | *Boehmeria clidemioides* | EU003923 |
| Angiosperm | Urticaceae | *Boehmeria clidemioides* | EU003924 |
| Angiosperm | Urticaceae | *Boehmeria clidemioides* | EU003925 |
| Angiosperm | Urticaceae | *Boehmeria clidemioides* var. *diffusa* | EU747109 |
| Angiosperm | Urticaceae | *Boehmeria clidemioides* var. *diffusa* | EU747110 |
| Angiosperm | Urticaceae | *Boehmeria clidemioides* var. *diffusa* | EU747123 |
| Angiosperm | Urticaceae | *Boehmeria clidemioides* var. *diffusa* | EU747124 |
| Angiosperm | Urticaceae | *Boehmeria nivea* | EU003918 |
| Angiosperm | Urticaceae | *Boehmeria nivea* | EU003919 |
| Angiosperm | Urticaceae | *Boehmeria nivea* | EU747095 |
| Angiosperm | Urticaceae | *Boehmeria nivea* | EU747099 |
| Angiosperm | Urticaceae | *Debregeasia edulis* | EU747102 |
| Angiosperm | Urticaceae | *Debregeasia edulis* | EU747107 |
| Angiosperm | Urticaceae | *Debregeasia edulis* | EU747114 |
| Angiosperm | Urticaceae | *Debregeasia edulis* | EU747119 |
| Angiosperm | Urticaceae | *Debregeasia edulis* | EU747121 |
| Angiosperm | Urticaceae | *Debregeasia edulis* | EU747122 |
| Angiosperm | Urticaceae | *Debregeasia elliptica* | EU747100 |
| Angiosperm | Urticaceae | *Debregeasia elliptica* | EU747108 |
| Angiosperm | Urticaceae | *Debregeasia longifolia* | EU747093 |
| Angiosperm | Urticaceae | *Debregeasia longifolia* | EU747120 |
| Angiosperm | Urticaceae | *Urtica mairei* | EU747118 |
| Angiosperm | Valerianaceae | *Valeriana jatamansi* | AY236190 |
| Angiosperm | Valerianaceae | *Valeriana officinalis* | DQ180745 |
| Angiosperm | Verbenaceae | *Aloysia triphylla* | EU761080 |
| Angiosperm | Verbenaceae | *Callicarpa angusta* | FM163240 |
| Angiosperm | Verbenaceae | *Callicarpa angustifolia* | FM163251 |
| Angiosperm | Verbenaceae | *Callicarpa arborea* | FM163233 |
| Angiosperm | Verbenaceae | *Callicarpa arborea* | FM163241 |
| Angiosperm | Verbenaceae | *Callicarpa candicans* | FM163242 |
| Angiosperm | Verbenaceae | *Callicarpa erioclona* | FM163234 |
| Angiosperm | Verbenaceae | *Callicarpa havilandii* var. *hispida* | FM163235 |
| Angiosperm | Verbenaceae | *Callicarpa havilandii* var. *hispida* | FM163236 |
| Angiosperm | Verbenaceae | *Callicarpa japonica* | FM163230 |
| Angiosperm | Verbenaceae | *Callicarpa macrophylla* | FM163246 |
| Angiosperm | Verbenaceae | *Callicarpa maingayi* | FM163247 |
| Angiosperm | Verbenaceae | *Callicarpa pentandra* | FM163237 |
| Angiosperm | Verbenaceae | *Callicarpa pentandra* | FM163248 |
| Angiosperm | Verbenaceae | *Callicarpa poilanei* | FM163254 |
| Angiosperm | Verbenaceae | *Callicarpa scandens* | FM163239 |
| Angiosperm | Verbenaceae | *Callicarpa stapfii* | FM163238 |
| Angiosperm | Verbenaceae | *Clerodendrum buchananii* | U77742 |
| Angiosperm | Verbenaceae | *Clerodendrum buchneri* | U77743 |
| Angiosperm | Verbenaceae | *Clerodendrum bungei* | U77744 |
| Angiosperm | Verbenaceae | *Clerodendrum cephalanthum* | U77745 |
| Angiosperm | Verbenaceae | *Clerodendrum eriophyllum* | U77747 |
| Angiosperm | Verbenaceae | *Clerodendrum glabrum* | U77748 |
| Angiosperm | Verbenaceae | *Clerodendrum mandarinorum* | U77758 |
| Angiosperm | Verbenaceae | *Clerodendrum minahassae* | U77759 |
| Angiosperm | Verbenaceae | *Clerodendrum paniculatum* | U77765 |
| Angiosperm | Verbenaceae | *Clerodendrum rotundifolium* | U77766 |
| Angiosperm | Verbenaceae | *Clerodendrum speciosissimum* | U77769 |
| Angiosperm | Verbenaceae | *Clerodendrum splendens* | U77770 |
| Angiosperm | Verbenaceae | *Clerodendrum trichotomum* | U77771 |
| Angiosperm | Verbenaceae | *Lippia alba* | EU761076 |
| Angiosperm | Verbenaceae | *Lippia alba* | EU761077 |
| Angiosperm | Verbenaceae | *Lippia alba* | EU761078 |
| Angiosperm | Verbenaceae | *Tetraclea coulteri* | AY307081 |
| Angiosperm | Verbenaceae | *Verbena urticifolia* | DQ006043 |
| Angiosperm | Verbenaceae | *Vitex agnus-castus* | EU785943 |
| Angiosperm | Verbenaceae | *Vitex cofassus* | DQ499149 |
| Angiosperm | Verbenaceae | *Vitex lucens* | DQ499150 |
| Angiosperm | Verbenaceae | *Vitex quinata* | AF477794 |
| Angiosperm | Violaceae | *Viola acuminata* | AY928273 |
| Angiosperm | Violaceae | *Viola albida* | AY928292 |
| Angiosperm | Violaceae | *Viola albida* | DQ112179 |
| Angiosperm | Violaceae | *Viola albida* | DQ787754 |
| Angiosperm | Violaceae | *Viola albida* | DQ787755 |
| Angiosperm | Violaceae | *Viola albida* var. *takahashii* | AY928293 |
| Angiosperm | Violaceae | *Viola albida x Viola x takahashii* | DQ112180 |
| Angiosperm | Violaceae | *Viola arvensis* | DQ055342 |
| Angiosperm | Violaceae | *Viola arvensis* | DQ055347 |
| Angiosperm | Violaceae | *Viola biflora* | DQ055348 |
| Angiosperm | Violaceae | *Viola biflora* | DQ055349 |
| Angiosperm | Violaceae | *Viola biflora* | DQ055350 |
| Angiosperm | Violaceae | *Viola brevistipulata* var. *minor* | AY928275 |
| Angiosperm | Violaceae | *Viola chaerophylloides* | DQ112183 |
| Angiosperm | Violaceae | *Viola chaerophylloides* | DQ787763 |
| Angiosperm | Violaceae | *Viola chaerophylloides* | DQ787764 |
| Angiosperm | Violaceae | *Viola chaerophylloides* | DQ787767 |
| Angiosperm | Violaceae | *Viola chaerophylloides* | DQ787770 |
| Angiosperm | Violaceae | *Viola chaerophylloides* | DQ787771 |
| Angiosperm | Violaceae | *Viola chaerophylloides x Viola x takahashii* | DQ112182 |
| Angiosperm | Violaceae | *Viola diamantiaca* | AY928288 |
| Angiosperm | Violaceae | *Viola eizanensis* | DQ787773 |
| Angiosperm | Violaceae | *Viola grypoceras* | AY928280 |
| Angiosperm | Violaceae | *Viola grypoceras f. albiflora* | AY928281 |
| Angiosperm | Violaceae | *Viola grypoceras* var. *exilis* | AY928282 |
| Angiosperm | Violaceae | *Viola guestphalica* | DQ055378 |
| Angiosperm | Violaceae | *Viola guestphalica* | DQ055380 |
| Angiosperm | Violaceae | *Viola guestphalica* | DQ055381 |
| Angiosperm | Violaceae | *Viola hirtipes* | AY928297 |
| Angiosperm | Violaceae | *Viola hondoensis* | AY928272 |
| Angiosperm | Violaceae | *Viola hondoensis* | AY928296 |
| Angiosperm | Violaceae | *Viola japonica* | AY928295 |
| Angiosperm | Violaceae | *Viola japonica* | AY928304 |
| Angiosperm | Violaceae | *Viola keiskei* | AY928303 |
| Angiosperm | Violaceae | *Viola kusanoana* | AY928278 |
| Angiosperm | Violaceae | *Viola lactiflora* | AY928299 |
| Angiosperm | Violaceae | *Viola lutea* | DQ055365 |
| Angiosperm | Violaceae | *Viola lutea* | DQ055373 |
| Angiosperm | Violaceae | *Viola lutea* subsp. *calaminaria* | DQ055355 |
| Angiosperm | Violaceae | *Viola lutea* subsp. *calaminaria* | DQ055357 |
| Angiosperm | Violaceae | *Viola lutea* subsp. *calaminaria* | DQ055362 |
| Angiosperm | Violaceae | *Viola mandshurica* | AY928300 |
| Angiosperm | Violaceae | *Viola orientalis* | AY928271 |
| Angiosperm | Violaceae | *Viola ovato-oblonga* | AY928277 |
| Angiosperm | Violaceae | *Viola patrinii* | AY928298 |
| Angiosperm | Violaceae | *Viola phalacrocarpa* | AY928294 |
| Angiosperm | Violaceae | *Viola philippica* | AY928302 |
| Angiosperm | Violaceae | *Viola raddeana* | AY928279 |
| Angiosperm | Violaceae | *Viola reichenbachiana* | DQ055388 |
| Angiosperm | Violaceae | *Viola reichenbachiana* | DQ055389 |
| Angiosperm | Violaceae | *Viola sacchalinensis* | AY928276 |
| Angiosperm | Violaceae | *Viola selkirkii* | AY928307 |
| Angiosperm | Violaceae | *Viola seoulensis* | AY928301 |
| Angiosperm | Violaceae | *Viola sieboldiana* | DQ787772 |
| Angiosperm | Violaceae | *Viola tenuicornis* | AY928306 |
| Angiosperm | Violaceae | *Viola tricolor* subsp. *curtisii* | DQ055399 |
| Angiosperm | Violaceae | *Viola tricolor* subsp. *curtisii* | DQ055403 |
| Angiosperm | Violaceae | *Viola tricolor* subsp. *curtisii* | DQ055405 |
| Angiosperm | Violaceae | *Viola tricolor* subsp. *tricolor* | DQ055392 |
| Angiosperm | Violaceae | *Viola tricolor* subsp. *tricolor* | DQ055398 |
| Angiosperm | Violaceae | *Viola tricolor* subsp. *tricolor* | DQ055406 |
| Angiosperm | Violaceae | *Viola tricolor* subsp. *tricolor* | DQ055409 |
| Angiosperm | Violaceae | *Viola tricolor* subsp. *tricolor* | DQ055411 |
| Angiosperm | Violaceae | *Viola tricolor* subsp. *tricolor* | DQ055414 |
| Angiosperm | Violaceae | *Viola variegata* | AY928305 |
| Angiosperm | Violaceae | *Viola verecunda* | AY928284 |
| Angiosperm | Violaceae | *Viola verecunda* var. *semilunaris* | AY928285 |
| Angiosperm | Violaceae | *Viola violacea* | AY928308 |
| Angiosperm | Violaceae | *Viola websteri* | AY928274 |
| Angiosperm | Violaceae | *Viola woosanensis* | AY928291 |
| Angiosperm | Violaceae | *Viola yazawana* | AY928289 |
| Angiosperm | Zingiberaceae | *Alpinia argentea* | AY742337 |
| Angiosperm | Zingiberaceae | *Alpinia blepharocalyx* | AF478709 |
| Angiosperm | Zingiberaceae | *Alpinia caerulea* | AY742342 |
| Angiosperm | Zingiberaceae | *Alpinia calcarata* | AF478710 |
| Angiosperm | Zingiberaceae | *Alpinia carolinensis* | AF478711 |
| Angiosperm | Zingiberaceae | *Alpinia chinensis* | EU909426 |
| Angiosperm | Zingiberaceae | *Alpinia conchigera* | AF478712 |
| Angiosperm | Zingiberaceae | *Alpinia elegans* | AF478713 |
| Angiosperm | Zingiberaceae | *Alpinia foxworthyi* | AF478714 |
| Angiosperm | Zingiberaceae | *Alpinia galanga* | AF478715 |
| Angiosperm | Zingiberaceae | *Alpinia galanga* | EU909429 |
| Angiosperm | Zingiberaceae | *Alpinia glabra* | AB097221 |
| Angiosperm | Zingiberaceae | *Alpinia intermedia* | AF202420 |
| Angiosperm | Zingiberaceae | *Alpinia intermedia* | AF478716 |
| Angiosperm | Zingiberaceae | *Alpinia japonica* | EU909427 |
| Angiosperm | Zingiberaceae | *Alpinia luteocarpa* | AF478717 |
| Angiosperm | Zingiberaceae | *Alpinia monopleura* | AY742363 |
| Angiosperm | Zingiberaceae | *Alpinia nieuwenhuizii* | AF414490 |
| Angiosperm | Zingiberaceae | *Alpinia pinetorum* | AY742373 |
| Angiosperm | Zingiberaceae | *Alpinia pumila* | AF478719 |
| Angiosperm | Zingiberaceae | *Alpinia rosea* | AY742377 |
| Angiosperm | Zingiberaceae | *Alpinia vittata* | AF478720 |
| Angiosperm | Zingiberaceae | *Amomum angustipetalum* | AB097245 |
| Angiosperm | Zingiberaceae | *Amomum austrosinense* | AY351985 |
| Angiosperm | Zingiberaceae | *Amomum calyptratum* | AB097239 |
| Angiosperm | Zingiberaceae | *Amomum compactum* | AY351986 |
| Angiosperm | Zingiberaceae | *Amomum coriaceum* | AB097240 |
| Angiosperm | Zingiberaceae | *Amomum coriandriodorum* | AY351987 |
| Angiosperm | Zingiberaceae | *Amomum dimorphum* | AB097244 |
| Angiosperm | Zingiberaceae | *Amomum durum* | AB097241 |
| Angiosperm | Zingiberaceae | *Amomum glabrum* | AY351989 |
| Angiosperm | Zingiberaceae | *Amomum gyrolophos* | AB097242 |
| Angiosperm | Zingiberaceae | *Amomum koenigii* | AY351991 |
| Angiosperm | Zingiberaceae | *Amomum lappaceum* | AF414488 |
| Angiosperm | Zingiberaceae | *Amomum laxesquamosum* | AY351994 |
| Angiosperm | Zingiberaceae | *Amomum longipetiolatum* | AF478722 |
| Angiosperm | Zingiberaceae | *Amomum maximum* | AY351995 |
| Angiosperm | Zingiberaceae | *Amomum menglaense* | AY351996 |
| Angiosperm | Zingiberaceae | *Amomum oliganthum* | AB097243 |
| Angiosperm | Zingiberaceae | *Amomum paratsaoko* | AY351997 |
| Angiosperm | Zingiberaceae | *Amomum pierreanum* | AY769829 |
| Angiosperm | Zingiberaceae | *Amomum propinquum* | AY351999 |
| Angiosperm | Zingiberaceae | *Amomum purpureorubrum* | AY352000 |
| Angiosperm | Zingiberaceae | *Amomum putrescens* | AY352002 |
| Angiosperm | Zingiberaceae | *Amomum quadratolaminare* | AY352003 |
| Angiosperm | Zingiberaceae | *Amomum queenslandicum* | AY352004 |
| Angiosperm | Zingiberaceae | *Amomum roseisquamosum* | AB097246 |
| Angiosperm | Zingiberaceae | *Amomum sericeum* | AY352005 |
| Angiosperm | Zingiberaceae | *Amomum somniculosum* | AB097247 |
| Angiosperm | Zingiberaceae | *Amomum subcapitatum* | AY352006 |
| Angiosperm | Zingiberaceae | *Amomum tsaoko* | AY352007 |
| Angiosperm | Zingiberaceae | *Amomum uliginosum* | AY352008 |
| Angiosperm | Zingiberaceae | *Amomum uliginosum* | AY769827 |
| Angiosperm | Zingiberaceae | *Amomum villosum* | AY352009 |
| Angiosperm | Zingiberaceae | *Amomum villosum* var. *xanthioides* | AY352011 |
| Angiosperm | Zingiberaceae | *Amomum yunnanense* | AY352012 |
| Angiosperm | Zingiberaceae | *Chamaecostus cuspidatus* | AY994739 |
| Angiosperm | Zingiberaceae | *Cheilocostus globosus* | AF434894 |
| Angiosperm | Zingiberaceae | *Cheilocostus globosus* | AY041037 |
| Angiosperm | Zingiberaceae | *Costus allenii* | AY041043 |
| Angiosperm | Zingiberaceae | *Costus allenii* | AY972877 |
| Angiosperm | Zingiberaceae | *Costus amazonicus* subsp. *krukovii* | AY972879 |
| Angiosperm | Zingiberaceae | *Costus asplundii* | AY972885 |
| Angiosperm | Zingiberaceae | *Costus chartaceus* | AY972911 |
| Angiosperm | Zingiberaceae | *Costus claviger* | AY994740 |
| Angiosperm | Zingiberaceae | *Costus dirzoi* | AY972930 |
| Angiosperm | Zingiberaceae | *Costus dubius* | AY972933 |
| Angiosperm | Zingiberaceae | *Costus dubius* | AY972936 |
| Angiosperm | Zingiberaceae | *Costus erythrophyllus* | AY972912 |
| Angiosperm | Zingiberaceae | *Costus gabonensis* | AY994747 |
| Angiosperm | Zingiberaceae | *Costus guanaiensis* var. *macrostrobilus* | AY972917 |
| Angiosperm | Zingiberaceae | *Costus laevis* | AY972918 |
| Angiosperm | Zingiberaceae | *Costus lasius* | AY972893 |
| Angiosperm | Zingiberaceae | *Costus lateriflorus* | AY994734 |
| Angiosperm | Zingiberaceae | *Costus letestui* | AY972939 |
| Angiosperm | Zingiberaceae | *Costus lima* var. *lima* | AY972926 |
| Angiosperm | Zingiberaceae | *Costus lucanusianus* | AY972935 |
| Angiosperm | Zingiberaceae | *Costus maculatus* | AY994731 |
| Angiosperm | Zingiberaceae | *Costus malortieanus* | AY972890 |
| Angiosperm | Zingiberaceae | *Costus malortieanus* | AY994732 |
| Angiosperm | Zingiberaceae | *Costus montanus* | AY972929 |
| Angiosperm | Zingiberaceae | *Costus montanus* | AY994729 |
| Angiosperm | Zingiberaceae | *Costus mosaicus* | AY994728 |
| Angiosperm | Zingiberaceae | *Costus osae* | AY972927 |
| Angiosperm | Zingiberaceae | *Costus phaeotrichus* | AY994721 |
| Angiosperm | Zingiberaceae | *Costus pictus* | AY972932 |
| Angiosperm | Zingiberaceae | *Costus productus* | AY972895 |
| Angiosperm | Zingiberaceae | *Costus pulverulentus* | AY041029 |
| Angiosperm | Zingiberaceae | *Costus pulverulentus* | AY673070 |
| Angiosperm | Zingiberaceae | *Costus pulverulentus* | AY972896 |
| Angiosperm | Zingiberaceae | *Costus pulverulentus* | AY972897 |
| Angiosperm | Zingiberaceae | *Costus pulverulentus* | AY972908 |
| Angiosperm | Zingiberaceae | *Costus pulverulentus* | AY972909 |
| Angiosperm | Zingiberaceae | *Costus pulverulentus* | AY994723 |
| Angiosperm | Zingiberaceae | *Costus pulverulentus* | AY994724 |
| Angiosperm | Zingiberaceae | *Costus scaber* | AY972900 |
| Angiosperm | Zingiberaceae | *Costus scaber* | AY972901 |
| Angiosperm | Zingiberaceae | *Costus scaber* | AY972902 |
| Angiosperm | Zingiberaceae | *Costus scaber* | AY972905 |
| Angiosperm | Zingiberaceae | *Costus spiralis* | AY972915 |
| Angiosperm | Zingiberaceae | *Costus stenophyllus* | AY972931 |
| Angiosperm | Zingiberaceae | *Costus stenophyllus* | AY994720 |
| Angiosperm | Zingiberaceae | *Costus talbotii* | AY972937 |
| Angiosperm | Zingiberaceae | *Costus varzearum* | AY972888 |
| Angiosperm | Zingiberaceae | *Costus varzearum* | AY972916 |
| Angiosperm | Zingiberaceae | *Costus vinosus* | AY972923 |
| Angiosperm | Zingiberaceae | *Costus zingiberoides* | AY972910 |
| Angiosperm | Zingiberaceae | *Curcuma aeruginosa* | AF478740 |
| Angiosperm | Zingiberaceae | *Curcuma bicolor* | AF478737 |
| Angiosperm | Zingiberaceae | *Curcuma petiolata* | AF202408 |
| Angiosperm | Zingiberaceae | *Curcuma roscoeana* | AF478739 |
| Angiosperm | Zingiberaceae | *Curcuma thorelii* | AF478741 |
| Angiosperm | Zingiberaceae | *Hedychium acuminatum* | AF202379 |
| Angiosperm | Zingiberaceae | *Hedychium bordelonianum* | AF478757 |
| Angiosperm | Zingiberaceae | *Hedychium borneense* | AF202380 |
| Angiosperm | Zingiberaceae | *Hedychium bousigonianum* | AF202381 |
| Angiosperm | Zingiberaceae | *Hedychium coccineum* | AF202375 |
| Angiosperm | Zingiberaceae | *Hedychium coronarium* | AF202383 |
| Angiosperm | Zingiberaceae | *Hedychium glabrum* | AF202387 |
| Angiosperm | Zingiberaceae | *Hedychium greenei* | AF478759 |
| Angiosperm | Zingiberaceae | *Hedychium hasseltii* | AF202389 |
| Angiosperm | Zingiberaceae | *Hedychium horsfieldii* | AF478760 |
| Angiosperm | Zingiberaceae | *Hedychium maximum* | AF202392 |
| Angiosperm | Zingiberaceae | *Hedychium puerense* | AF202394 |
| Angiosperm | Zingiberaceae | *Hedychium spicatum* | AF202395 |
| Angiosperm | Zingiberaceae | *Hedychium tenuiflorum* | AF202376 |
| Angiosperm | Zingiberaceae | *Hedychium thyrsiforme* | AF202403 |
| Angiosperm | Zingiberaceae | *Hedychium villosum* | AF478762 |
| Angiosperm | Zingiberaceae | *Hedychium yunnanense* | AF202398 |
| Angiosperm | Zingiberaceae | *Hornstedtia conica* | AF414481 |
| Angiosperm | Zingiberaceae | *Hornstedtia gracilis* | AF414482 |
| Angiosperm | Zingiberaceae | *Hornstedtia hainanensis* | AF478766 |
| Angiosperm | Zingiberaceae | *Hornstedtia havilandii* | AF414479 |
| Angiosperm | Zingiberaceae | *Hornstedtia leonurus* | AB097237 |
| Angiosperm | Zingiberaceae | *Hornstedtia minor* | AB097238 |
| Angiosperm | Zingiberaceae | *Hornstedtia reticulata* | AB097236 |
| Angiosperm | Zingiberaceae | *Hornstedtia sanhan* | AY769844 |
| Angiosperm | Zingiberaceae | *Hornstedtia scottiana* | AF414480 |
| Angiosperm | Zingiberaceae | *Kaempferia rotunda* | AF478767 |
| Angiosperm | Zingiberaceae | *Plagiostachys crocydocalyx* | AB097250 |
| Angiosperm | Zingiberaceae | *Plagiostachys glandulosa* | AB097251 |
| Angiosperm | Zingiberaceae | *Plagiostachys parva* | AF414491 |
| Angiosperm | Zingiberaceae | *Plagiostachys strobilifera* | AB097252 |
| Angiosperm | Zingiberaceae | *Setaria parviflora* | AF019831 |
| Angiosperm | Zingiberaceae | *Zingiber coloratum* | AF414498 |
| Angiosperm | Zingiberaceae | *Zingiber corallinum* | AF202418 |
| Angiosperm | Zingiberaceae | *Zingiber ellipticum* | AF478799 |
| Angiosperm | Zingiberaceae | *Zingiber gramineum* | AF478800 |
| Angiosperm | Zingiberaceae | *Zingiber longipedunculatum* | AB097254 |
| Angiosperm | Zingiberaceae | *Zingiber spectabile* | AF414499 |
| Angiosperm | Zingiberaceae | *Zingiber sulphureum* | AF478801 |
| Angiosperm | Zingiberaceae | *Zingiber wrayii* | AF478802 |
| Angiosperm | Zygophyllaceae | *Tribulus terrestris* | AY260972 |
| Angiosperm | Zygophyllaceae | *Tribulus terrestris* | DQ233661 |
| Angiosperm | Zygophyllaceae | *Tribulus terrestris* | DQ309043 |
| Gymnosperm | Cupressaceae | *Chamaecyparis pisifera* | DQ269982 |
| Gymnosperm | Cupressaceae | *Juniperus saltuaria* | EU243567 |
| Gymnosperm | Cupressaceae | *Platycladus orientalis* | AY380875 |
| Gymnosperm | Cupressaceae | *Platycladus orientalis* | AY836780 |
| Gymnosperm | Cycadaceae | *Cycas circinalis* | AF531222 |
| Gymnosperm | Cycadaceae | *Cycas furfuracea* | AF531225 |
| Gymnosperm | Cycadaceae | *Cycas micholitzii* | AB076240 |
| Gymnosperm | Cycadaceae | *Cycas revolute* | AF531223 |
| Gymnosperm | Cycadaceae | *Cycas taitungensis* | AB076188 |
| Gymnosperm | Ginkgoaceae | *Ginkgo biloba* | AB076568 |
| Gymnosperm | Ginkgoaceae | *Ginkgo biloba* | DQ191445 |
| Gymnosperm | Ginkgoaceae | *Ginkgo biloba* | EU350117 |
| Gymnosperm | Ginkgoaceae | *Ginkgo biloba* | EU643829 |
| Gymnosperm | Ginkgoaceae | *Ginkgo biloba* | Y16380 |
| Gymnosperm | Ginkgoaceae | *Ginkgo biloba* | Y16892 |
| Gymnosperm | Gnetaceae | *Gnetum africanum* | AY449543 |
| Gymnosperm | Gnetaceae | *Gnetum africanum* | AY449545 |
| Gymnosperm | Gnetaceae | *Gnetum africanum* | AY449546 |
| Gymnosperm | Gnetaceae | *Gnetum cuspidatum* | AY449547 |
| Gymnosperm | Gnetaceae | *Gnetum cuspidatum* | AY449549 |
| Gymnosperm | Gnetaceae | *Gnetum cuspidatum* | AY449551 |
| Gymnosperm | Gnetaceae | *Gnetum diminutum* | AY449552 |
| Gymnosperm | Gnetaceae | *Gnetum diminutum* | AY449554 |
| Gymnosperm | Gnetaceae | *Gnetum gnemon* | AY449558 |
| Gymnosperm | Gnetaceae | *Gnetum gnemon* | AY449559 |
| Gymnosperm | Gnetaceae | *Gnetum gnemon* | AY449560 |
| Gymnosperm | Gnetaceae | *Gnetum gnemon* | AY449561 |
| Gymnosperm | Gnetaceae | *Gnetum gnemon* | AY449562 |
| Gymnosperm | Gnetaceae | *Gnetum hainanense* | AY449563 |
| Gymnosperm | Gnetaceae | *Gnetum hainanense* | AY449564 |
| Gymnosperm | Gnetaceae | *Gnetum hainanense* | AY449565 |
| Gymnosperm | Gnetaceae | *Gnetum hainanense* | AY449566 |
| Gymnosperm | Gnetaceae | *Gnetum hainanense* | AY449567 |
| Gymnosperm | Gnetaceae | *Gnetum hainanense* | AY449568 |
| Gymnosperm | Gnetaceae | *Gnetum hainanense* | AY449569 |
| Gymnosperm | Gnetaceae | *Gnetum hainanense* | AY449570 |
| Gymnosperm | Gnetaceae | *Gnetum hainanense* | AY449571 |
| Gymnosperm | Gnetaceae | *Gnetum hainanense* | AY449572 |
| Gymnosperm | Gnetaceae | *Gnetum hainanense* | AY449573 |
| Gymnosperm | Gnetaceae | *Gnetum hainanense* | AY449574 |
| Gymnosperm | Gnetaceae | *Gnetum hainanense* | AY449575 |
| Gymnosperm | Gnetaceae | *Gnetum hainanense* | AY449576 |
| Gymnosperm | Gnetaceae | *Gnetum hainanense* | AY449577 |
| Gymnosperm | Gnetaceae | *Gnetum klossii* | AY449579 |
| Gymnosperm | Gnetaceae | *Gnetum klossii* | AY449581 |
| Gymnosperm | Gnetaceae | *Gnetum latifolium* | AY449584 |
| Gymnosperm | Gnetaceae | *Gnetum latifolium* | AY449585 |
| Gymnosperm | Gnetaceae | *Gnetum leyboldii* var. *woodsonianum* | AY449612 |
| Gymnosperm | Gnetaceae | *Gnetum leyboldii* var. *woodsonianum* | AY449613 |
| Gymnosperm | Gnetaceae | *Gnetum leyboldii* var. *woodsonianum* | AY449614 |
| Gymnosperm | Gnetaceae | *Gnetum leyboldii* var. *woodsonianum* | AY449615 |
| Gymnosperm | Gnetaceae | *Gnetum neglectum* | AY449597 |
| Gymnosperm | Gnetaceae | *Gnetum neglectum* | AY449598 |
| Gymnosperm | Gnetaceae | *Gnetum nodiflorum* | AY449601 |
| Gymnosperm | Gnetaceae | *Gnetum nodiflorum* | AY449602 |
| Gymnosperm | Gnetaceae | *Gnetum parvifolium* | AY449603 |
| Gymnosperm | Gnetaceae | *Gnetum parvifolium* | AY449604 |
| Gymnosperm | Gnetaceae | *Gnetum ula* | AY449608 |
| Gymnosperm | Gnetaceae | *Gnetum ula* | AY449609 |
| Gymnosperm | Gnetaceae | *Gnetum urens* | AY449610 |
| Gymnosperm | Gnetaceae | *Gnetum urens* | AY449611 |
| Gymnosperm | Pinaceae | *Cathaya argyrophylla* | DQ975347 |
| Gymnosperm | Pinaceae | *Cathaya argyrophylla* | DQ975348 |
| Gymnosperm | Pinaceae | *Picea mexicana* | U24251 |
| Gymnosperm | Pinaceae | *Pinus armandii* | AY430072 |
| Gymnosperm | Pinaceae | *Pinus flexilis* | AY430075 |
| Gymnosperm | Pinaceae | *Pinus sibirica* | AY430077 |
| Gymnosperm | Pinaceae | *Pseudolarix amabilis* | DQ975355 |
| Gymnosperm | Pinaceae | *Pseudolarix amabilis* | DQ975356 |
| Gymnosperm | Podocarpaceae | *Nageia nagi* | AB023989 |
| Gymnosperm | Taxaceae | *Torreya californica* | AF259275 |
| Gymnosperm | Taxaceae | *Torreya fargesii* | AF259283 |
| Gymnosperm | Taxaceae | *Torreya fargesii* var. *yunnanensis* | AF259284 |
| Gymnosperm | Taxaceae | *Torreya grandis* | AF259278 |
| Gymnosperm | Taxaceae | *Torreya taxifolia* | AF259273 |
| Gymnosperm | Taxodiaceae | *Taxodium distichum* | AF387535 |
| Gymnosperm | Taxodiaceae | *Taxodium distichum* var. *imbricarium* | AF387536 |
| Fern | Ephedraceae | *Ephedra alata* | AY755774 |
| Fern | Ephedraceae | *Ephedra ciliata* | AY755776 |
| Fern | Ephedraceae | *Ephedra equisetina* | AY755751 |
| Fern | Ephedraceae | *Ephedra foliata* | AY755775 |
| Fern | Ephedraceae | *Ephedra likiangensis* | AY755766 |
| Fern | Ephedraceae | *Ephedra minuta* | AY755755 |
| Fern | Ephedraceae | *Ephedra monosperma* | AY755746 |
| Fern | Ephedraceae | *Ephedra pachyclada* | AY755779 |
| Fern | Ephedraceae | *Ephedra rhytidosperma* | DQ212958 |
| Fern | Ephedraceae | *Ephedra torreyana* | AY755759 |
| Fern | Ephedraceae | *Ephedra tweediana* | AY755768 |
| Fern | Equisetaceae | *Equisetum ramosissimum* | AF448794 |
| Fern | Lycopodiaceae | *Diphasiastrum alpinum* | AF338758 |
| Fern | Lycopodiaceae | *Lycopodium annotinum* | AF338755 |
| Fern | Lycopodiaceae | *Lycopodium casuarinoides* | AF338751 |
| Fern | Lycopodiaceae | *Lycopodium clavatum* | AF338759 |
| Fern | Lycopodiaceae | *Lycopodium deuterodensum* | AF338756 |
| Fern | Lycopodiaceae | *Lycopodium fastigiatum* | AF338757 |
| Fern | Lycopodiaceae | *Lycopodium obscurum* | AF338752 |
| Fern | Lycopodiaceae | *Lycopodium scariosum* | AF338754 |
| Fern | Lycopodiaceae | *Lycopodium volubile* | AF338753 |
| Fern | Lygodiaceae | *Lygodium japonicum* | AF448793 |
| Fern | Pteridaceae | *Pteris cretica* | AM920396 |
| Fern | Pteridaceae | *Pteris ensiformis* | AM920397 |
| Fern | Pteridaceae | *Pteris vittata* | AM920401 |
| Fern | Pteridaceae | *Pteris vittata* | AM920402 |
| Fern | Pteridaceae | *Pteris vittata* | AM920403 |
| Fern | Salviniacae | *Salvinia oblongifolia* | DQ522291 |
| Fern | Selaginellaceae | *Selaginella arenicola* | AF419008 |
| Fern | Selaginellaceae | *Selaginella arizonica x Selaginella eremophila* | AF419046 |
| Fern | Selaginellaceae | *Selaginella arsenei* | AF419029 |
| Fern | Selaginellaceae | *Selaginella asprella* | AF419022 |
| Fern | Selaginellaceae | *Selaginella balansae* | AF419005 |
| Fern | Selaginellaceae | *Selaginella bigelovii* | AF419004 |
| Fern | Selaginellaceae | *Selaginella caffrorum* | AF419017 |
| Fern | Selaginellaceae | *Selaginella cinerascens* | AF419027 |
| Fern | Selaginellaceae | *Selaginella dregei* | AF419015 |
| Fern | Selaginellaceae | *Selaginella eremophila* | AF419010 |
| Fern | Selaginellaceae | *Selaginella extensa* | AF419026 |
| Fern | Selaginellaceae | *Selaginella hansenii* | AF419033 |
| Fern | Selaginellaceae | *Selaginella landii* | AF419012 |
| Fern | Selaginellaceae | *Selaginella leucobryoides* | AF419023 |
| Fern | Selaginellaceae | *Selaginella mutica* | AF419025 |
| Fern | Selaginellaceae | *Selaginella neomexicana* | AF419047 |
| Fern | Selaginellaceae | *Selaginella nivea* | AF419014 |
| Fern | Selaginellaceae | *Selaginella njamnjamensis* | AF419016 |
| Fern | Selaginellaceae | *Selaginella oregana* | AF419030 |
| Fern | Selaginellaceae | *Selaginella peruviana* | AF419013 |
| Fern | Selaginellaceae | *Selaginella phillipsiana* | AF419019 |
| Fern | Selaginellaceae | *Selaginella rupincola* | AF419003 |
| Fern | Selaginellaceae | *Selaginella sartorii* | AF419039 |
| Fern | Selaginellaceae | *Selaginella sellowii* | AF419028 |
| Fern | Selaginellaceae | *Selaginella sibirica* | AF419032 |
| Fern | Selaginellaceae | *Selaginella steyermarkii* | AF419034 |
| Fern | Selaginellaceae | *Selaginella tortipila* | AF419007 |
| Fern | Selaginellaceae | *Selaginella underwoodii* | AF419031 |
| Fern | Selaginellaceae | *Selaginella utahensis* | AF419024 |
| Fern | Selaginellaceae | *Selaginella wallacei* | AF419035 |
| Fern | Selaginellaceae | *Selaginella watsonii* | AF419037 |
| Fern | Selaginellaceae | *Selaginella weatherbiana* | AF419006 |
| Fern | Selaginellaceae | *Selaginella wrightii* | AF419011 |
| Moss | Marchantiaceae | *Marchantia polymorpha* | AY342318 |
| Moss | Sphagnaceae | *Sphagnum angustifolium* | AF061159 |
| Moss | Sphagnaceae | *Sphagnum angustifolium* | AF061164 |
| Moss | Sphagnaceae | *Sphagnum angustifolium* | AF061165 |
| Moss | Sphagnaceae | *Sphagnum angustifolium* | AF061166 |
| Moss | Sphagnaceae | *Sphagnum angustifolium* | AF061168 |
| Moss | Sphagnaceae | *Sphagnum angustifolium* | AF061170 |
| Moss | Sphagnaceae | *Sphagnum angustifolium* | AF061173 |
| Moss | Sphagnaceae | *Sphagnum angustifolium* | AF061174 |
| Moss | Sphagnaceae | *Sphagnum angustifolium* | AF061176 |
| Liverwort | Usneaceae | *Usnea aciculifera* | AB051049 |
| Liverwort | Usneaceae | *Usnea arizonica* | AF297732 |
| Liverwort | Usneaceae | *Usnea articulata* | AJ457140 |
| Liverwort | Usneaceae | *Usnea aurantiacoatra* | EF179798 |
| Liverwort | Usneaceae | *Usnea baileyi* | AB051051 |
| Liverwort | Usneaceae | *Usnea barbata* | AJ457138 |
| Liverwort | Usneaceae | *Usnea chaetophora* | AJ748106 |
| Liverwort | Usneaceae | *Usnea dasaea* | AB051056 |
| Liverwort | Usneaceae | *Usnea diffracta* | AB051059 |
| Liverwort | Usneaceae | *Usnea diffracta* | AB051060 |
| Liverwort | Usneaceae | *Usnea filipendula* | AJ457149 |
| Liverwort | Usneaceae | *Usnea filipendula* | AJ457150 |
| Liverwort | Usneaceae | *Usnea florida* | AJ457148 |
| Liverwort | Usneaceae | *Usnea fragilescens* | AJ748104 |
| Liverwort | Usneaceae | *Usnea fragilescens* | AJ748105 |
| Liverwort | Usneaceae | *Usnea glabrescens* | AB051639 |
| Liverwort | Usneaceae | *Usnea himalayana* | AB051640 |
| Liverwort | Usneaceae | *Usnea hirta* | AJ457151 |
| Liverwort | Usneaceae | *Usnea longissima* | AB051645 |
| Liverwort | Usneaceae | *Usnea longissima* | AB051647 |
| Liverwort | Usneaceae | *Usnea merrillii* | AB051649 |
| Liverwort | Usneaceae | *Usnea mutabilis* | AB051651 |
| Liverwort | Usneaceae | *Usnea nipparensis* | AB051652 |
| Liverwort | Usneaceae | *Usnea pangiana* | AB051654 |
| Liverwort | Usneaceae | *Usnea pectinata* | AB051655 |
| Liverwort | Usneaceae | *Usnea pectinata* | AB051656 |
| Liverwort | Usneaceae | *Usnea pygmoidea* | AB051657 |
| Liverwort | Usneaceae | *Usnea rigida* | AJ457152 |
| Liverwort | Usneaceae | *Usnea rubicunda* | AB051659 |
| Liverwort | Usneaceae | *Usnea rubrotincta* | AB051661 |
| Liverwort | Usneaceae | *Usnea sphacelata* | DQ767960 |
| Liverwort | Usneaceae | *Usnea strigosa* | AF112990 |
| Liverwort | Usneaceae | *Usnea subantarctica* | EF179806 |
| Liverwort | Usneaceae | *Usnea trichodeoides* | AB051672 |
| Liverwort | Usneaceae | *Usnea wasmuthii* | AB051673 |
| Liverwort | Usneaceae | *Usnea wasmuthii* | AB051674 |
| Liverwort | Usneaceae | *Usnea wasmuthii* | AB051676 |
| Algae | Alariaceae | *Lessoniopsis littoralis* | AF319035 |
| Algae | Alariaceae | *Pterygophora californica* | AF319005 |
| Algae | Alariaceae | *Undaria peterseniana* | AF319006 |
| Algae | Alariaceae | *Undaria pinnatifida* | AF319007 |
| Algae | Alariaceae | *Undaria undarioides* | AF319008 |
| Algae | Costariaceae | *Agarum clathratum* | AF319025 |
| Algae | Costariaceae | *Agarum turneri* | AF319026 |
| Algae | Costariaceae | *Costaria costata* | AF319027 |
| Algae | Costariaceae | *Thalassiophyllum clathrus* | AF319028 |
| Algae | Diplostomatidae | *Alaria crassifolia* | AF319001 |
| Algae | Diplostomatidae | *Alaria esculenta* | AF319002 |
| Algae | Diplostomatidae | *Alaria marginata* | AF319003 |
| Algae | Diplostomatidae | *Alaria praelonga* | AF319004 |
| Algae | Laminariaceae | *Arthrothamnus bifidus* | AB087245 |
| Algae | Laminariaceae | *Arthrothamnus bifidus* | AF319023 |
| Algae | Laminariaceae | *Cymathaere triplicata* | AY857884 |
| Algae | Laminariaceae | *Hedophyllum sessile* | AF319024 |
| Algae | Laminariaceae | *Laminaria digitata* | AB087251 |
| Algae | Laminariaceae | *Laminaria digitata* | FJ042773 |
| Algae | Laminariaceae | *Laminaria ephemera* | AY857887 |
| Algae | Laminariaceae | *Laminaria ephemera* | FJ042733 |
| Algae | Laminariaceae | *Laminaria farlowii* | AY857888 |
| Algae | Laminariaceae | *Laminaria hyperborea* | AY441771 |
| Algae | Laminariaceae | *Laminaria hyperborea* | AY441772 |
| Algae | Laminariaceae | *Laminaria hyperborea* | AY441773 |
| Algae | Laminariaceae | *Laminaria setchellii* | AF319016 |
| Algae | Laminariaceae | *Laminaria sinclairii* | AF319017 |
| Algae | Laminariaceae | *Laminaria solidungula* | FJ042759 |
| Algae | Laminariaceae | *Laminaria yezoensis* | AY857885 |
| Algae | Laminariaceae | *Laminaria yezoensis* | FJ042727 |
| Algae | Laminariaceae | *Laminaria yezoensis* | FJ042749 |
| Algae | Laminariaceae | *Macrocystis integrifolia* | AF319036 |
| Algae | Laminariaceae | *Macrocystis integrifolia* | AY857882 |
| Algae | Laminariaceae | *Macrocystis pyrifera* | AF319037 |
| Algae | Laminariaceae | *Pelagophycus porra* | AF319039 |
| Algae | Laminariaceae | *Postelsia palmaeformis* | AF319034 |
| Algae | Laminariaceae | *Postelsia palmaeformis* | AF362998 |
| Algae | Laminariaceae | *Saccharina dentigera* | AY857895 |
| Algae | Laminariaceae | *Saccharina groenlandica* | AY857894 |
| Algae | Laminariaceae | *Saccharina gyrata* | AF319021 |
| Algae | Laminariaceae | *Saccharina japonica* | AB022790 |
| Algae | Laminariaceae | *Saccharina japonica* | DQ143048 |
| Algae | Laminariaceae | *Saccharina japonica* | DQ143049 |
| Algae | Laminariaceae | *Saccharina japonica* | DQ143050 |
| Algae | Laminariaceae | *Saccharina japonica* | DQ143051 |
| Algae | Laminariaceae | *Saccharina kurilensis* | AF319022 |
| Algae | Laminariaceae | *Saccharina latissima* | AF319019 |
| Algae | Laminariaceae | *Saccharina sculpera* | AB087250 |
| Algae | Laminariaceae | *Saccharina sculpera* | AF319020 |
| Algae | Lessoniaceae | *Ecklonia cava* | AF319009 |
| Algae | Lessoniaceae | *Ecklonia kurome* | EF407574 |
| Algae | Lessoniaceae | *Ecklonia maxima* | EF407575 |
| Algae | Lessoniaceae | *Ecklonia radiata* | AY857898 |
| Algae | Lessoniaceae | *Ecklonia stolonifera* | AF319010 |
| Algae | Lessoniaceae | *Eckloniopsis radicosa* | AF319011 |
| Algae | Lessoniaceae | *Lessonia flavicans* | AF319031 |
| Algae | Lessoniaceae | *Lessonia trabeculata* | AF319030 |
| Algae | Lessoniaceae | *Lessonia vadosa* | AF319032 |
| Algae | Sargassaceae | *Sargassum binderi* | AB043116 |
| Algae | Sargassaceae | *Sargassum boreale* | AB038270 |
| Algae | Sargassaceae | *Sargassum carpophyllum* | AB043067 |
| Algae | Sargassaceae | *Sargassum confusum* | AB038271 |
| Algae | Sargassaceae | *Sargassum crassifolium* | AB043115 |
| Algae | Sargassaceae | *Sargassum cristaefolium* | AY315628 |
| Algae | Sargassaceae | *Sargassum cristaefolium* | AY315630 |
| Algae | Sargassaceae | *Sargassum duplicatum* | AY258152 |
| Algae | Sargassaceae | *Sargassum duplicatum* | AY315629 |
| Algae | Sargassaceae | *Sargassum duplicatum* | AY315637 |
| Algae | Sargassaceae | *Sargassum mcclurei* | AB043111 |
| Algae | Sargassaceae | *Sargassum microceratium* | AB038273 |
| Algae | Sargassaceae | *Sargassum pallidum* | AB038274 |
| Algae | Sargassaceae | *Sargassum pallidum* | AY150002 |
| Algae | Sargassaceae | *Sargassum piluliferum* | AY150019 |
| Algae | Sargassaceae | *Sargassum quinhonense* | AB043112 |
| Fungi | Agaricomycotina | *Rhizoctonia solani* | FJ492159 |
| Fungi | Ascomycota | *Corynespora cassiicola* | FJ852614 |
| Fungi | Ascomycota | *Stachybotrys dichroa* | FJ914701 |
| Fungi | Botryosphaeriaceae | *Botryosphaeria dothidea* | EU979360 |
| Fungi | Clavicipitaceae | *Cordyceps bifusispora* | AY245627 |
| Fungi | Clavicipitaceae | *Cordyceps brasiliensis* | AJ536545 |
| Fungi | Clavicipitaceae | *Cordyceps brongniartii* | DQ376245 |
| Fungi | Clavicipitaceae | *Cordyceps cardinalis* | AB237660 |
| Fungi | Clavicipitaceae | *Cordyceps chlamydosporia* | AB100362 |
| Fungi | Clavicipitaceae | *Cordyceps cuboidea* | AB296169 |
| Fungi | Clavicipitaceae | *Cordyceps cylindrica* | EF029230 |
| Fungi | Clavicipitaceae | *Cordyceps formosana* | EF689044 |
| Fungi | Clavicipitaceae | *Cordyceps gunnii* | AJ536552 |
| Fungi | Clavicipitaceae | *Cordyceps imagamiana* | AJ536547 |
| Fungi | Clavicipitaceae | *Cordyceps kanzashiana* | AB027371 |
| Fungi | Clavicipitaceae | *Cordyceps koreana* | AJ536569 |
| Fungi | Clavicipitaceae | *Cordyceps kyushuensis* | AY781661 |
| Fungi | Clavicipitaceae | *Cordyceps militaris* | EU825999 |
| Fungi | Clavicipitaceae | *Cordyceps ochraceostromata* | AY245646 |
| Fungi | Clavicipitaceae | *Cordyceps pruinosa* | AY491995 |
| Fungi | Clavicipitaceae | *Cordyceps pseudomilitaris* | AY646404 |
| Fungi | Clavicipitaceae | *Cordyceps roseostromata* | AY245637 |
| Fungi | Clavicipitaceae | *Cordyceps scarabaeucika* | AY491993 |
| Fungi | Clavicipitaceae | *Cordyceps spegazzinii* | DQ196435 |
| Fungi | Clavicipitaceae | *Cordyceps sphingum* | AY245641 |
| Fungi | Clavicipitaceae | *Elaphocordyceps capitata* | EF530933 |
| Fungi | Clavicipitaceae | *Elaphocordyceps ophioglossoides* | AY245636 |
| Fungi | Clavicipitaceae | *Metacordyceps brittlebankisoides* | AJ309332 |
| Fungi | Clavicipitaceae | *Ophiocordyceps agriotidis* | AY245626 |
| Fungi | Clavicipitaceae | *Ophiocordyceps crinalis* | EF495104 |
| Fungi | Clavicipitaceae | *Ophiocordyceps dipterigena* | AY245629 |
| Fungi | Clavicipitaceae | *Ophiocordyceps heteropoda* | AY245630 |
| Fungi | Clavicipitaceae | *Ophiocordyceps myrmecophila* | AY245635 |
| Fungi | Clavicipitaceae | *Ophiocordyceps nutans* | AJ536559 |
| Fungi | Clavicipitaceae | *Ophiocordyceps robertsii* | AJ309336 |
| Fungi | Clavicipitaceae | *Ophiocordyceps sinensis* | AB067715 |
| Fungi | Clavicipitaceae | *Ophiocordyceps sinensis* | AB067739 |
| Fungi | Clavicipitaceae | *Ophiocordyceps sinensis* | AB067742 |
| Fungi | Clavicipitaceae | *Ophiocordyceps sinensis* | AB067748 |
| Fungi | Clavicipitaceae | *Ophiocordyceps sinensis* | AJ309354 |
| Fungi | Clavicipitaceae | *Ophiocordyceps sinensis* | AJ413181 |
| Fungi | Clavicipitaceae | *Ophiocordyceps sinensis* | AJ507403 |
| Fungi | Clavicipitaceae | *Ophiocordyceps sinensis* | AY725795 |
| Fungi | Clavicipitaceae | *Ophiocordyceps sobolifera* | AY754003 |
| Fungi | Clavicipitaceae | *Ophiocordyceps sphecocephala* | AY491994 |
| Fungi | Erysiphaceae | *Leveillula taurica* | AB044998 |
| Fungi | Erysiphaceae | *Phyllactinia guttata* | AB080525 |
| Fungi | Lumbricidae | *Eisenia bicyclis* | AF319012 |
| Fungi | Lycoperdaceae | *Calvatia candida* | DQ112624 |
| Fungi | Lycoperdaceae | *Calvatia craniiformis* | DQ112625 |
| Fungi | Lycoperdaceae | *Calvatia cretacea* | DQ112597 |
| Fungi | Lycoperdaceae | *Calvatia gigantea* | AJ617492 |
| Fungi | Lycoperdaceae | *Calvatia turneri* | DQ112594 |
| Fungi | Mycosphaerellaceae | *Mycosphaerella cannabis* | AY152549 |
| Fungi | Peronosporales | *Phytophthora capsici* | AB359950 |
| Fungi | Peronosporales | *Phytophthora drechsleri* | AY251651 |
| Fungi | Pleosporales | *Coniothyrium diplodiella* | AB470851 |
| Fungi | Pleosporales | *Phoma dictamnicola* | EF600960 |
| Fungi | Polyporaceae | *Ganoderma carnosum* | EU486458 |
| Fungi | Polyporaceae | *Ganoderma cupreum* | AY569450 |
| Fungi | Polyporaceae | *Ganoderma fornicatum* | AY593863 |
| Fungi | Polyporaceae | *Ganoderma gibbosum* | EU918695 |
| Fungi | Polyporaceae | *Ganoderma lucidum* | EU520235 |
| Fungi | Polyporaceae | *Ganoderma lucidum* | EU520247 |
| Fungi | Polyporaceae | *Ganoderma mastoporum* | AJ627585 |
| Fungi | Polyporaceae | *Ganoderma neojaponicum* | AY593867 |
| Fungi | Polyporaceae | *Ganoderma pfeifferi* | AY884185 |
| Fungi | Polyporaceae | *Ganoderma philippii* | AJ627584 |
| Fungi | Polyporaceae | *Ganoderma ramosissimum* | EU918700 |
| Fungi | Polyporaceae | *Ganoderma resinaceum* | EF060007 |
| Fungi | Polyporaceae | *Ganoderma sinense* | DQ424982 |
| Fungi | Polyporaceae | *Ganoderma sinense* | DQ424990 |
| Fungi | Polyporaceae | *Ganoderma sinense* | DQ424995 |
| Fungi | Polyporaceae | *Ganoderma sinense* | DQ425014 |
| Fungi | Polyporaceae | *Ganoderma weberianum* | AY569451 |
| Fungi | Polyporaceae | *Polyporus arcularius* | AF516522 |
| Fungi | Polyporaceae | *Polyporus badius* | AF516559 |
| Fungi | Polyporaceae | *Polyporus brumalis* | AF516536 |
| Fungi | Polyporaceae | *Polyporus ciliatus* | AF516537 |
| Fungi | Polyporaceae | *Polyporus corylinus* | AF516538 |
| Fungi | Polyporaceae | *Polyporus dictyopus* | AF516562 |
| Fungi | Polyporaceae | *Polyporus gayanus* | AF518757 |
| Fungi | Polyporaceae | *Polyporus grammocephalus* | AF516563 |
| Fungi | Polyporaceae | *Polyporus guianensis* | AF516566 |
| Fungi | Polyporaceae | *Polyporus leprieurii* | AF516567 |
| Fungi | Polyporaceae | *Polyporus melanopus* | AF516569 |
| Fungi | Polyporaceae | *Polyporus mikawai* | AF516570 |
| Fungi | Polyporaceae | *Polyporus pseudobetulinus* | AF516571 |
| Fungi | Polyporaceae | *Polyporus radicatus* | AF516585 |
| Fungi | Polyporaceae | *Polyporus squamosus* | AY218421 |
| Fungi | Polyporaceae | *Polyporus tricholoma* | AJ132942 |
| Fungi | Polyporaceae | *Polyporus tuberaster* | AY218420 |
| Fungi | Polyporaceae | *Polyporus udus* | AF518756 |
| Fungi | Polyporaceae | *Polyporus umbellatus* | AY322495 |
| Fungi | Polyporaceae | *Polyporus varius* | AF518758 |
| Fungi | Polyporaceae | *Polyporus virgatus* | AF516582 |
| Fungi | Polyporaceae | *Poria cocos* | EF397597 |
| Fungi | Polyporaceae | *Trametes versicolor* | AY673076 |
| Fungi | Teloschistaceae | *Caloplaca dichroa* | DQ173232 |
