# Supplementary material for: Validation of the ITS2 Region as a Novel DNA Barcode for Identifying Medicinal Plant Species
Source: PLoS One. 2010 Jan 7;5(1):e8613. doi: 10.1371/journal.pone.0008613 (PMC2799520; doi:10.1371/journal.pone.0008613)
Supplement: Table S7 — Samples for determining the ability of the psbA-trnH barcode to identify species and accession numbers in GenBank. (1.28 MB DOC) [file pone.0008613.s010.doc]

**Table S7.** Samples for determining the ability of the *psbA-trnH* barcode to identify species and accession numbers in GenBank.

| **Phylum** | **Family** | **Species** | **GenBank accession** |
| --- | --- | --- | --- |
| Angiosperm | Aceraceae | *Acer negundo* | EU750427 |
| Angiosperm | Aceraceae | *Acer negundo* | EU750428 |
| Angiosperm | Aceraceae | *Acer platanoides* | EU750429 |
| Angiosperm | Aceraceae | *Acer platanoides* | EU750430 |
| Angiosperm | Aceraceae | *Acer rubrum* | EU750431 |
| Angiosperm | Aceraceae | *Acer rubrum* | EU750432 |
| Angiosperm | Aceraceae | *Acer rubrum* | EU750433 |
| Angiosperm | Aceraceae | *Acer saccharinum* | EU750434 |
| Angiosperm | Aceraceae | *Acer saccharinum* | EU750435 |
| Angiosperm | Aceraceae | *Acer saccharinum* | EU750436 |
| Angiosperm | Aceraceae | *Acer saccharinum* | EU750437 |
| Angiosperm | Aceraceae | *Acer saccharum* | EU750438 |
| Angiosperm | Aceraceae | *Acer saccharum* | EU750439 |
| Angiosperm | Aceraceae | *Acer saccharum* | EU750440 |
| Angiosperm | Anacardiaceae | *Rhus gueinzii* | EU213842 |
| Angiosperm | Anacardiaceae | *Rhus gueinzii* | EU213843 |
| Angiosperm | Anacardiaceae | *Rhus gueinzii* | EU213844 |
| Angiosperm | Anacardiaceae | *Rhus transvaalensis* | EU213848 |
| Angiosperm | Anacardiaceae | *Rhus transvaalensis* | EU213849 |
| Angiosperm | Anacardiaceae | *Rhus transvaalensis* | EU213850 |
| Angiosperm | Anacardiaceae | *Searsia leptodictya* | EU213845 |
| Angiosperm | Anacardiaceae | *Searsia leptodictya* | EU213846 |
| Angiosperm | Anacardiaceae | *Searsia leptodictya* | EU213847 |
| Angiosperm | Apiaceae | *Geocaryum bornmuelleri* | DQ457175 |
| Angiosperm | Apiaceae | *Heteromorpha arborescens* | EU213836 |
| Angiosperm | Apiaceae | *Heteromorpha arborescens* | EU213837 |
| Angiosperm | Apiaceae | *Heteromorpha arborescens* | EU213838 |
| Angiosperm | Apiaceae | *Pimpinella affinis* | AY587848 |
| Angiosperm | Apiaceae | *Pimpinella anisetum* | AY587849 |
| Angiosperm | Apiaceae | *Pimpinella anisum* | AY587850 |
| Angiosperm | Apiaceae | *Pimpinella anthriscoides* var. *anthriscoides* | AY587851 |
| Angiosperm | Apiaceae | *Pimpinella aromatica* | AY587852 |
| Angiosperm | Apiaceae | *Pimpinella aurea* | AY587853 |
| Angiosperm | Apiaceae | *Pimpinella cappadocica* var. *cappadocica* | AY587854 |
| Angiosperm | Apiaceae | *Pimpinella corymbosa* | AY587855 |
| Angiosperm | Apiaceae | *Pimpinella cretica* var. *arabica* | AY587856 |
| Angiosperm | Apiaceae | *Pimpinella cretica* var. *cretica* | AY587857 |
| Angiosperm | Apiaceae | *Pimpinella eriocarpa* | AY587858 |
| Angiosperm | Apiaceae | *Pimpinella flabellifolia* | AY587859 |
| Angiosperm | Apiaceae | *Pimpinella isaurica* | AY587860 |
| Angiosperm | Apiaceae | *Pimpinella kotschyana* | AY587861 |
| Angiosperm | Apiaceae | *Pimpinella nudicaulis* | AY587862 |
| Angiosperm | Apiaceae | *Pimpinella oliverioides* | AY587863 |
| Angiosperm | Apiaceae | *Pimpinella paucidentata* | AY587864 |
| Angiosperm | Apiaceae | *Pimpinella peregrina* | AY587865 |
| Angiosperm | Apiaceae | *Pimpinella peucedanifolia* | AY587866 |
| Angiosperm | Apiaceae | *Pimpinella puberula* | AY587867 |
| Angiosperm | Apiaceae | *Pimpinella rhodantha* | AY587868 |
| Angiosperm | Apiaceae | *Pimpinella saxifraga* | AY587869 |
| Angiosperm | Apiaceae | *Pimpinella sintenisii* | AY587870 |
| Angiosperm | Apiaceae | *Pimpinella tragium* subsp. *lithophila* | AY587871 |
| Angiosperm | Apiaceae | *Pimpinella tragium* subsp. *pseudotragium* | AY587873 |
| Angiosperm | Apiaceae | *Steganotaenia araliacea* | EU213854 |
| Angiosperm | Apiaceae | *Steganotaenia araliacea* | EU213855 |
| Angiosperm | Apiaceae | *Steganotaenia araliacea* | EU213856 |
| Angiosperm | Apiaceae | *Thapsia villosa* | EU531683 |
| Angiosperm | Apiaceae | *Trigonosciadium brachytaenium* | DQ996582 |
| Angiosperm | Apiaceae | *Trigonosciadium lasiocarpum* | DQ996581 |
| Angiosperm | Apiaceae | *Daucus aureus* | EU531688 |
| Angiosperm | Apiaceae | *Daucus carota* | EU531686 |
| Angiosperm | Apiaceae | *Daucus carota* | FJ493257 |
| Angiosperm | Apiaceae | *Daucus crinitus* | EU531684 |
| Angiosperm | Apiaceae | *Daucus durieua* | EU531687 |
| Angiosperm | Apiaceae | *Daucus muricatus* | EU531689 |
| Angiosperm | Apiaceae | *Daucus setifolius* | EU531685 |
| Angiosperm | Apiaceae | *Foeniculum vulgare* | AY587874 |
| Angiosperm | Apocynaceae | *Nerium oleander* | EU531690 |
| Angiosperm | Apocynaceae | *Nerium oleander* | FJ493258 |
| Angiosperm | Apocynaceae | *Vinca minor* | FJ493259 |
| Angiosperm | Araliaceae | *Hedera colchica* | AY163512 |
| Angiosperm | Araliaceae | *Hedera cypria* | AY163516 |
| Angiosperm | Araliaceae | *Hedera helix* subsp. *hibernica* | AJ537465 |
| Angiosperm | Araliaceae | *Hedera maderensis* subsp. *iberica* | AY163514 |
| Angiosperm | Araliaceae | *Hedera maroccana* | AY163513 |
| Angiosperm | Araliaceae | *Hedera nepalensis* var. *nepalensis* | AY163523 |
| Angiosperm | Araliaceae | *Hedera nepalensis* var. *sinensis* | AY163522 |
| Angiosperm | Araliaceae | *Hedera rhombea* | AY163510 |
| Angiosperm | Araliaceae | *Panax ginseng* | AY582139 |
| Angiosperm | Arecaceae | *Hyphaene coriacea* | EU213775 |
| Angiosperm | Arecaceae | *Hyphaene coriacea* | EU213776 |
| Angiosperm | Arecaceae | *Hyphaene coriacea* | EU213777 |
| Angiosperm | Arecaceae | *Hyphaene coriacea* | EU213778 |
| Angiosperm | Arecaceae | *Hyphaene petersiana* | EU213779 |
| Angiosperm | Arecaceae | *Hyphaene petersiana* | EU213780 |
| Angiosperm | Asclepiadaceae | *Cibirhiza albersiana* | AM231756 |
| Angiosperm | Asclepiadaceae | *Cibirhiza dhofarensis* | AM231757 |
| Angiosperm | Asclepiadaceae | *Fockea angustifolia* | AM231758 |
| Angiosperm | Asclepiadaceae | *Fockea angustifolia* | AM231759 |
| Angiosperm | Asclepiadaceae | *Fockea angustifolia* | AM231760 |
| Angiosperm | Asclepiadaceae | *Fockea angustifolia* | AM231761 |
| Angiosperm | Asclepiadaceae | *Fockea capensis* | AM231762 |
| Angiosperm | Asclepiadaceae | *Fockea comaru* | AM231763 |
| Angiosperm | Asclepiadaceae | *Fockea edulis* | AM231765 |
| Angiosperm | Asclepiadaceae | *Fockea multiflora* | AM231766 |
| Angiosperm | Asclepiadaceae | *Fockea multiflora* | AM231767 |
| Angiosperm | Asclepiadaceae | *Fockea multiflora* | AM231768 |
| Angiosperm | Asclepiadaceae | *Fockea sinuata* | AM231769 |
| Angiosperm | Asclepiadaceae | *Secamone filiformis* | AM231772 |
| Angiosperm | Asclepiadaceae | *Telosma africana* | AM231773 |
| Angiosperm | Asclepiadaceae | *Vincetoxicum acuminatum* | AB109145 |
| Angiosperm | Asclepiadaceae | *Vincetoxicum ambiguum* | AB109146 |
| Angiosperm | Asclepiadaceae | *Vincetoxicum amplexicaule* | AB109147 |
| Angiosperm | Asclepiadaceae | *Vincetoxicum atratum* | AB109148 |
| Angiosperm | Asclepiadaceae | *Vincetoxicum austrokiusianum* | AB109149 |
| Angiosperm | Asclepiadaceae | *Vincetoxicum calcareum* | AB109150 |
| Angiosperm | Asclepiadaceae | *Vincetoxicum hoyoense* | AB109164 |
| Angiosperm | Asclepiadaceae | *Vincetoxicum inamoenum* | AB109165 |
| Angiosperm | Asclepiadaceae | *Vincetoxicum izuense* | AB109163 |
| Angiosperm | Asclepiadaceae | *Vincetoxicum japonicum* | AB109151 |
| Angiosperm | Asclepiadaceae | *Vincetoxicum katoi* | AB109152 |
| Angiosperm | Asclepiadaceae | *Vincetoxicum macrophyllum* | AB109154 |
| Angiosperm | Asclepiadaceae | *Vincetoxicum macrophyllum* | AB109155 |
| Angiosperm | Asclepiadaceae | *Vincetoxicum magnificum* | AB109156 |
| Angiosperm | Asclepiadaceae | *Vincetoxicum nipponicum* | AB109157 |
| Angiosperm | Asclepiadaceae | *Vincetoxicum pycnostelma* | AB109161 |
| Angiosperm | Asclepiadaceae | *Vincetoxicum sublanceolatum* var. *macranthum* | AB109160 |
| Angiosperm | Asclepiadaceae | *Vincetoxicum sublanceolatum* var. *sublanceolatum* | AB109159 |
| Angiosperm | Asclepiadaceae | *Vincetoxicum yamanakae* | AB109153 |
| Angiosperm | Asclepiadaceae | *Vincetoxicum yonakuniense* | AB109162 |
| Angiosperm | Asteraceae | *Ajania shiwogiku* | EF091602 |
| Angiosperm | Asteraceae | *Anacyclus homogamus* | EU531693 |
| Angiosperm | Asteraceae | *Anacyclus pyrethrum* | EU531691 |
| Angiosperm | Asteraceae | *Anacyclus radiatus* | EU531692 |
| Angiosperm | Asteraceae | *Andryala agardhii* | AY898756 |
| Angiosperm | Asteraceae | *Andryala glandulosa* | AY898759 |
| Angiosperm | Asteraceae | *Anthemis arvensis* | EU547792 |
| Angiosperm | Asteraceae | *Anthemis cotula* | EU547794 |
| Angiosperm | Asteraceae | *Arctium lappa* | AF129838 |
| Angiosperm | Asteraceae | *Arctotheca calendula* | DQ444764 |
| Angiosperm | Asteraceae | *Arctotheca forbesiana* | EU846399 |
| Angiosperm | Asteraceae | *Arctotheca marginata* | EU846400 |
| Angiosperm | Asteraceae | *Arctotheca populifolia* | EU846401 |
| Angiosperm | Asteraceae | *Arctotheca prostrata* | EU846402 |
| Angiosperm | Asteraceae | *Arctotis acaulis* | DQ444765 |
| Angiosperm | Asteraceae | *Arctotis adpressa* | EU846415 |
| Angiosperm | Asteraceae | *Arctotis angustifolia* | EU846416 |
| Angiosperm | Asteraceae | *Arctotis arctotoides* | DQ444766 |
| Angiosperm | Asteraceae | *Arctotis arctotoides* | EU846405 |
| Angiosperm | Asteraceae | *Arctotis argentea* | EU846417 |
| Angiosperm | Asteraceae | *Arctotis aspera* | DQ444767 |
| Angiosperm | Asteraceae | *Arctotis aspera* var. *scabra* | EU846419 |
| Angiosperm | Asteraceae | *Arctotis auriculata* | EU846420 |
| Angiosperm | Asteraceae | *Arctotis bellidifolia* | EU846421 |
| Angiosperm | Asteraceae | *Arctotis bellidifolia* | EU846422 |
| Angiosperm | Asteraceae | *Arctotis breviscapa* | DQ444768 |
| Angiosperm | Asteraceae | *Arctotis campanulata* | EU846423 |
| Angiosperm | Asteraceae | *Arctotis canescens* | EU846424 |
| Angiosperm | Asteraceae | *Arctotis debensis* | EU846406 |
| Angiosperm | Asteraceae | *Arctotis decurrens* | EU846425 |
| Angiosperm | Asteraceae | *Arctotis dregei* | DQ444769 |
| Angiosperm | Asteraceae | *Arctotis dregei* | EU846403 |
| Angiosperm | Asteraceae | *Arctotis elongata* | EU846426 |
| Angiosperm | Asteraceae | *Arctotis erosa* | EU846427 |
| Angiosperm | Asteraceae | *Arctotis erosa* | EU846428 |
| Angiosperm | Asteraceae | *Arctotis fastuosa* | EU846431 |
| Angiosperm | Asteraceae | *Arctotis flaccida* | EU846432 |
| Angiosperm | Asteraceae | *Arctotis flaccida* | EU846433 |
| Angiosperm | Asteraceae | *Arctotis graminea* | EU846434 |
| Angiosperm | Asteraceae | *Arctotis hirsuta* | EU846435 |
| Angiosperm | Asteraceae | *Arctotis hispidula* | EU846408 |
| Angiosperm | Asteraceae | *Arctotis incisa* | EU846436 |
| Angiosperm | Asteraceae | *Arctotis laevis* | EU846438 |
| Angiosperm | Asteraceae | *Arctotis lanceolata* | EU846439 |
| Angiosperm | Asteraceae | *Arctotis leiocarpa* | EU846441 |
| Angiosperm | Asteraceae | *Arctotis leucanthemoides* | EU846442 |
| Angiosperm | Asteraceae | *Arctotis microcephala* | EU846409 |
| Angiosperm | Asteraceae | *Arctotis microcephala* | EU846410 |
| Angiosperm | Asteraceae | *Arctotis muricata* | EU846443 |
| Angiosperm | Asteraceae | *Arctotis perfoliata* | DQ444770 |
| Angiosperm | Asteraceae | *Arctotis perfoliata* | EU846411 |
| Angiosperm | Asteraceae | *Arctotis pinnatifida* | EU846444 |
| Angiosperm | Asteraceae | *Arctotis reptans* | EU846445 |
| Angiosperm | Asteraceae | *Arctotis revoluta* | EU846447 |
| Angiosperm | Asteraceae | *Arctotis rotundifolia* | EU846448 |
| Angiosperm | Asteraceae | *Arctotis scapiformis* | EU846412 |
| Angiosperm | Asteraceae | *Arctotis scapiformis* | EU846413 |
| Angiosperm | Asteraceae | *Arctotis semipapposa* | EU846450 |
| Angiosperm | Asteraceae | *Arctotis stoechadifolia* | EU846451 |
| Angiosperm | Asteraceae | *Arctotis stoechadifolia* | EU846452 |
| Angiosperm | Asteraceae | *Arctotis sulcocarpa* | DQ444772 |
| Angiosperm | Asteraceae | *Arctotis venusta* | DQ444773 |
| Angiosperm | Asteraceae | *Arctotis verbascifolia* | EU846454 |
| Angiosperm | Asteraceae | *Artemisia annua* | FJ418749 |
| Angiosperm | Asteraceae | *Artemisia capillaris* | FJ418750 |
| Angiosperm | Asteraceae | *Artemisia caruifolia* | FJ418751 |
| Angiosperm | Asteraceae | *Berkheya carduoides* | DQ444760 |
| Angiosperm | Asteraceae | *Brachylaena huillensis* | EU213791 |
| Angiosperm | Asteraceae | *Brachylaena huillensis* | EU213792 |
| Angiosperm | Asteraceae | *Brachylaena huillensis* | EU213793 |
| Angiosperm | Asteraceae | *Carduus nutans* | AF129839 |
| Angiosperm | Asteraceae | *Carlina brachylepis* | EU531703 |
| Angiosperm | Asteraceae | *Carlina macrophylla* | EU531704 |
| Angiosperm | Asteraceae | *Catananche arenaria* | EU531695 |
| Angiosperm | Asteraceae | *Catananche caerulea* | EU531696 |
| Angiosperm | Asteraceae | *Catananche lutea* | EU531694 |
| Angiosperm | Asteraceae | *Centaurea aspera* | DQ846282 |
| Angiosperm | Asteraceae | *Centaurea aspera* subsp. *pseudoaerocephala* | DQ846283 |
| Angiosperm | Asteraceae | *Centaurea corymbosa* | DQ846180 |
| Angiosperm | Asteraceae | *Centaurea corymbosa* | DQ846181 |
| Angiosperm | Asteraceae | *Centaurea exarata* | DQ846216 |
| Angiosperm | Asteraceae | *Centaurea filiformis* | DQ846186 |
| Angiosperm | Asteraceae | *Centaurea filiformis* | DQ846257 |
| Angiosperm | Asteraceae | *Centaurea maculosa* subsp. *albida* | DQ846168 |
| Angiosperm | Asteraceae | *Centaurea maculosa* subsp. *maculosa* | DQ846184 |
| Angiosperm | Asteraceae | *Centaurea maculosa* subsp. *subalbida* | DQ846213 |
| Angiosperm | Asteraceae | *Centaurea maculosa* subsp. *subalbida* | DQ846215 |
| Angiosperm | Asteraceae | *Centaurea paniculata* | DQ846189 |
| Angiosperm | Asteraceae | *Centaurea paniculata* | DQ846191 |
| Angiosperm | Asteraceae | *Centaurea subtilis* | DQ846219 |
| Angiosperm | Asteraceae | *Centaurea subtilis* | DQ846220 |
| Angiosperm | Asteraceae | *Centaurea subtilis* | DQ846221 |
| Angiosperm | Asteraceae | *Centaurea vallesiaca* | DQ846175 |
| Angiosperm | Asteraceae | *Centaurea vallesiaca* | DQ846208 |
| Angiosperm | Asteraceae | *Chrysanthemum arcticum* | AB234775 |
| Angiosperm | Asteraceae | *Chrysanthemum arcticum* subsp. *maekawanum* | EF091604 |
| Angiosperm | Asteraceae | *Chrysanthemum arisanense* | AB234780 |
| Angiosperm | Asteraceae | *Chrysanthemum arisanense* | AB234781 |
| Angiosperm | Asteraceae | *Chrysanthemum boreale* | AB234778 |
| Angiosperm | Asteraceae | *Chrysanthemum chanetii* | EF091605 |
| Angiosperm | Asteraceae | *Chrysanthemum crassum* | EF091606 |
| Angiosperm | Asteraceae | *Chrysanthemum dichrum* | EF091607 |
| Angiosperm | Asteraceae | *Chrysanthemum indicum* | AB234760 |
| Angiosperm | Asteraceae | *Chrysanthemum indicum* | EF091608 |
| Angiosperm | Asteraceae | *Chrysanthemum japonense* | AB234771 |
| Angiosperm | Asteraceae | *Chrysanthemum japonense* | AB234772 |
| Angiosperm | Asteraceae | *Chrysanthemum japonicum* | EF091612 |
| Angiosperm | Asteraceae | *Chrysanthemum lavandulifolium* | EF091614 |
| Angiosperm | Asteraceae | *Chrysanthemum marginatum* | EF091622 |
| Angiosperm | Asteraceae | *Chrysanthemum nankingense* | EF091615 |
| Angiosperm | Asteraceae | *Chrysanthemum okiense* | EF091616 |
| Angiosperm | Asteraceae | *Chrysanthemum ornatum* | EF091617 |
| Angiosperm | Asteraceae | *Chrysanthemum rupestre* | AB234773 |
| Angiosperm | Asteraceae | *Chrysanthemum vestitum* | EF091618 |
| Angiosperm | Asteraceae | *Chrysanthemum wakasaense* | EF091613 |
| Angiosperm | Asteraceae | *Chrysanthemum weyrichii* | AB234774 |
| Angiosperm | Asteraceae | *Chrysanthemum x morifolium* | EF091621 |
| Angiosperm | Asteraceae | *Chrysanthemum x morifolium* | EF091625 |
| Angiosperm | Asteraceae | *Chrysanthemum x shimotomaii* | EF091624 |
| Angiosperm | Asteraceae | *Chrysanthemum yoshinaganthum* | EF091626 |
| Angiosperm | Asteraceae | *Chrysanthemum zawadskii* | AB234743 |
| Angiosperm | Asteraceae | *Chrysanthemum zawadskii* subsp. *latilobum* | EF091628 |
| Angiosperm | Asteraceae | *Cymbonotus lawsonianus* | DQ444774 |
| Angiosperm | Asteraceae | *Cymbonotus lawsonianus* | EU846404 |
| Angiosperm | Asteraceae | *Cynara baetica* | EU531698 |
| Angiosperm | Asteraceae | *Cynara cardunculus* | AF129842 |
| Angiosperm | Asteraceae | *Cynara humilis* | AF129843 |
| Angiosperm | Asteraceae | *Cynara humilis* | EU531699 |
| Angiosperm | Asteraceae | *Dendranthema indicum* var. *aromaticum* | EF091609 |
| Angiosperm | Asteraceae | *Dendranthema vestitum* var. *lanceifolium* | EF091619 |
| Angiosperm | Asteraceae | *Echinacea angustifolia* | EU785933 |
| Angiosperm | Asteraceae | *Echinacea pallida* | EU785935 |
| Angiosperm | Asteraceae | *Echinacea purpurea* | EU785934 |
| Angiosperm | Asteraceae | *Echinops sphaerocephalus* | AF129844 |
| Angiosperm | Asteraceae | *Encelia actoni* | DQ383887 |
| Angiosperm | Asteraceae | *Encelia asperifolia* | DQ383888 |
| Angiosperm | Asteraceae | *Encelia asperifolia* | DQ383889 |
| Angiosperm | Asteraceae | *Encelia californica* | DQ383890 |
| Angiosperm | Asteraceae | *Encelia canescens* | DQ383891 |
| Angiosperm | Asteraceae | *Encelia conspersa* | DQ383893 |
| Angiosperm | Asteraceae | *Encelia densifolia* | DQ383894 |
| Angiosperm | Asteraceae | *Encelia farinosa* | DQ661036 |
| Angiosperm | Asteraceae | *Erigeron annuus* | EU750456 |
| Angiosperm | Asteraceae | *Erigeron annuus* | EU750457 |
| Angiosperm | Asteraceae | *Erigeron annuus* | EU750458 |
| Angiosperm | Asteraceae | *Erigeron compositus* | DQ131883 |
| Angiosperm | Asteraceae | *Erigeron pallens* | DQ131884 |
| Angiosperm | Asteraceae | *Erigeron strigosus* | EU750459 |
| Angiosperm | Asteraceae | *Erigeron strigosus* | EU750460 |
| Angiosperm | Asteraceae | *Erigeron strigosus* | EU750461 |
| Angiosperm | Asteraceae | *Erigeron strigosus* | EU750462 |
| Angiosperm | Asteraceae | *Erigeron trifidus* | DQ131882 |
| Angiosperm | Asteraceae | *Eupatorium perfoliatum* | EU750465 |
| Angiosperm | Asteraceae | *Eupatorium perfoliatum* | EU750466 |
| Angiosperm | Asteraceae | *Eutrochium maculatum* | EU750463 |
| Angiosperm | Asteraceae | *Eutrochium maculatum* | EU750464 |
| Angiosperm | Asteraceae | *Gazania heterochaeta* | EF556370 |
| Angiosperm | Asteraceae | *Gazania heterochaeta* | EF556371 |
| Angiosperm | Asteraceae | *Gazania krebsiana* | DQ444762 |
| Angiosperm | Asteraceae | *Gazania krebsiana* subsp. *arctotoides* | EF556378 |
| Angiosperm | Asteraceae | *Gazania leiopoda* | EF556382 |
| Angiosperm | Asteraceae | *Gazania leiopoda* | EF556383 |
| Angiosperm | Asteraceae | *Gazania linearis* subsp. *linearis* | EF556386 |
| Angiosperm | Asteraceae | *Gazania linearis* subsp. *ovalis* | EF556388 |
| Angiosperm | Asteraceae | *Gazania maritima* | EF556391 |
| Angiosperm | Asteraceae | *Gazania pectinata* | EF556393 |
| Angiosperm | Asteraceae | *Gazania serrata* | EF556403 |
| Angiosperm | Asteraceae | *Gazania serrata* | EF556404 |
| Angiosperm | Asteraceae | *Gazania tenuifolia* | EF556405 |
| Angiosperm | Asteraceae | *Gazania tenuifolia* | EF556406 |
| Angiosperm | Asteraceae | *Haplocarpha nervosa* | DQ444778 |
| Angiosperm | Asteraceae | *Haplocarpha nervosa* | EU846394 |
| Angiosperm | Asteraceae | *Haplocarpha parvifolia* | EU846398 |
| Angiosperm | Asteraceae | *Haplocarpha rueppellii* | DQ444779 |
| Angiosperm | Asteraceae | *Haplocarpha rueppellii* | EU846395 |
| Angiosperm | Asteraceae | *Haplocarpha scaposa* | DQ444780 |
| Angiosperm | Asteraceae | *Haplocarpha scaposa* | EU846396 |
| Angiosperm | Asteraceae | *Haplocarpha schimperi* | EU846397 |
| Angiosperm | Asteraceae | *Hieracium alpinum* | AY898758 |
| Angiosperm | Asteraceae | *Hirpicium echinus* | DQ444763 |
| Angiosperm | Asteraceae | *Jacobaea abrotanifolia* | AY155643 |
| Angiosperm | Asteraceae | *Jacobaea alpina* | AY155645 |
| Angiosperm | Asteraceae | *Jacobaea aquatica* var. *aquatica* | AY155646 |
| Angiosperm | Asteraceae | *Jacobaea arnautorum* | AY155662 |
| Angiosperm | Asteraceae | *Jacobaea boissieri* | AY155648 |
| Angiosperm | Asteraceae | *Jacobaea cannabifolia* var. *integrifolia* | AY155649 |
| Angiosperm | Asteraceae | *Jacobaea carniolica* subsp. *insubrica* | AY155656 |
| Angiosperm | Asteraceae | *Jacobaea erucifolia* | AY155652 |
| Angiosperm | Asteraceae | *Jacobaea gigantea* | AY155653 |
| Angiosperm | Asteraceae | *Jacobaea gnaphalioides* | AY155654 |
| Angiosperm | Asteraceae | *Jacobaea leucophylla* | AY155658 |
| Angiosperm | Asteraceae | *Jacobaea maritima* | AY155647 |
| Angiosperm | Asteraceae | *Jacobaea minuta* | AY155659 |
| Angiosperm | Asteraceae | *Jacobaea othonnae* | AY155660 |
| Angiosperm | Asteraceae | *Jacobaea paludosa* | AY155661 |
| Angiosperm | Asteraceae | *Jacobaea persoonii* | AY155663 |
| Angiosperm | Asteraceae | *Jacobaea subalpina* | AY155664 |
| Angiosperm | Asteraceae | *Jacobaea uniflora* | AY155665 |
| Angiosperm | Asteraceae | *Jacobaea vulgaris* | AY155657 |
| Angiosperm | Asteraceae | *Jurinea cyanoides* | AJ289234 |
| Angiosperm | Asteraceae | *Jurinea humilis* | AF129846 |
| Angiosperm | Asteraceae | *Lactuca canadensis* | EU750467 |
| Angiosperm | Asteraceae | *Lactuca canadensis* | EU750468 |
| Angiosperm | Asteraceae | *Lactuca sativa* | AP007232 |
| Angiosperm | Asteraceae | *Lactuca sativa* | NC007578 |
| Angiosperm | Asteraceae | *Lactuca serriola* | EU750469 |
| Angiosperm | Asteraceae | *Lactuca serriola* | EU750470 |
| Angiosperm | Asteraceae | *Matricaria matricarioides* | EU547791 |
| Angiosperm | Asteraceae | *Matricaria recutita* | EU547790 |
| Angiosperm | Asteraceae | *Onopordum acanthium* | AF129848 |
| Angiosperm | Asteraceae | *Packera aurea* | AY155641 |
| Angiosperm | Asteraceae | *Packera eurycephala* | EF538040 |
| Angiosperm | Asteraceae | *Pittocaulon praecox* | DQ131854 |
| Angiosperm | Asteraceae | *Pittocaulon praecox* | EF538042 |
| Angiosperm | Asteraceae | *Psacalium cirsiifolium* | EF538038 |
| Angiosperm | Asteraceae | *Psacalium peltatum* | DQ131863 |
| Angiosperm | Asteraceae | *Pulicaria mauritanica* | EU531702 |
| Angiosperm | Asteraceae | *Pulicaria odora* | EU531700 |
| Angiosperm | Asteraceae | *Pulicaria paludosa* | EF211002 |
| Angiosperm | Asteraceae | *Pulicaria paludosa* | EU531701 |
| Angiosperm | Asteraceae | *Robinsonia berteroi* | EF538082 |
| Angiosperm | Asteraceae | *Robinsonia gracilis* | EF538068 |
| Angiosperm | Asteraceae | *Senecio adamantinus* | EF538059 |
| Angiosperm | Asteraceae | *Senecio arnaldii* | EF538067 |
| Angiosperm | Asteraceae | *Senecio ayopayensis* | EF538083 |
| Angiosperm | Asteraceae | *Senecio chilensis* | EF538043 |
| Angiosperm | Asteraceae | *Senecio deltoideus* | EF538076 |
| Angiosperm | Asteraceae | *Senecio flavus* | EF538087 |
| Angiosperm | Asteraceae | *Senecio glaberrimus* | EF538081 |
| Angiosperm | Asteraceae | *Senecio hemmendorffii* | EF538063 |
| Angiosperm | Asteraceae | *Senecio jarae* | EF538044 |
| Angiosperm | Asteraceae | *Senecio latifolius* | EF538084 |
| Angiosperm | Asteraceae | *Senecio lineatus* | EF538075 |
| Angiosperm | Asteraceae | *Senecio mairetianus* | EF538045 |
| Angiosperm | Asteraceae | *Senecio medley-woodii* | EF538080 |
| Angiosperm | Asteraceae | *Senecio nemorensis* | EF538046 |
| Angiosperm | Asteraceae | *Senecio otites* | EF538065 |
| Angiosperm | Asteraceae | *Senecio pinifolius* | EF538079 |
| Angiosperm | Asteraceae | *Senecio retrorsus* | EF538077 |
| Angiosperm | Asteraceae | *Senecio saxatilis* | EF538086 |
| Angiosperm | Asteraceae | *Senecio scandens* | EF538048 |
| Angiosperm | Asteraceae | *Senecio scaposus* | EF538049 |
| Angiosperm | Asteraceae | *Senecio stigophlebius* | EF538072 |
| Angiosperm | Asteraceae | *Senecio thapsoides* | EF538085 |
| Angiosperm | Asteraceae | *Senecio triqueter* | EF538074 |
| Angiosperm | Asteraceae | *Senecio viscosus* | AY155666 |
| Angiosperm | Asteraceae | *Senecio vulgaris* | EF538071 |
| Angiosperm | Asteraceae | *Senecio vulgaris* | FJ493263 |
| Angiosperm | Asteraceae | *Silybum marianum* | AF129851 |
| Angiosperm | Asteraceae | *Solidago caesia* | EU750561 |
| Angiosperm | Asteraceae | *Solidago caesia* | EU750562 |
| Angiosperm | Asteraceae | *Solidago canadensis* | EU337694 |
| Angiosperm | Asteraceae | *Solidago canadensis* | EU750564 |
| Angiosperm | Asteraceae | *Solidago canadensis* var. *scabra* | EU750557 |
| Angiosperm | Asteraceae | *Solidago canadensis* var. *scabra* | EU750558 |
| Angiosperm | Asteraceae | *Solidago canadensis* var. *scabra* | EU750559 |
| Angiosperm | Asteraceae | *Solidago canadensis* var. *scabra* | EU750560 |
| Angiosperm | Asteraceae | *Solidago flexicaulis* | EU750565 |
| Angiosperm | Asteraceae | *Solidago flexicaulis* | EU750566 |
| Angiosperm | Asteraceae | *Solidago gigantea* | EU337193 |
| Angiosperm | Asteraceae | *Solidago gigantea* | EU337195 |
| Angiosperm | Asteraceae | *Solidago lepida* | EU337702 |
| Angiosperm | Asteraceae | *Solidago nemoralis* | EU750567 |
| Angiosperm | Asteraceae | *Solidago nemoralis* | EU750568 |
| Angiosperm | Asteraceae | *Solidago nemoralis* | EU750569 |
| Angiosperm | Asteraceae | *Solidago rugosa* | EU750570 |
| Angiosperm | Asteraceae | *Solidago rugosa* | EU750571 |
| Angiosperm | Asteraceae | *Solidago rugosa* | EU750572 |
| Angiosperm | Asteraceae | *Solidago virgaurea* | EU337703 |
| Angiosperm | Asteraceae | *Solidago virgaurea* | EU337705 |
| Angiosperm | Asteraceae | *Sonchus asper* | EU750573 |
| Angiosperm | Asteraceae | *Sonchus asper* | EU750574 |
| Angiosperm | Asteraceae | *Sonchus asper* | EU750575 |
| Angiosperm | Asteraceae | *Sonchus oleraceus* | EU750576 |
| Angiosperm | Asteraceae | *Sonchus oleraceus* | EU750577 |
| Angiosperm | Asteraceae | *Symphyotrichum ciliolatum* | EU750578 |
| Angiosperm | Asteraceae | *Symphyotrichum ciliolatum* | EU750579 |
| Angiosperm | Asteraceae | *Symphyotrichum ciliolatum* | EU750580 |
| Angiosperm | Asteraceae | *Symphyotrichum ericoides* | EU750581 |
| Angiosperm | Asteraceae | *Symphyotrichum ericoides* | EU750582 |
| Angiosperm | Asteraceae | *Symphyotrichum lanceolatum* | EU750583 |
| Angiosperm | Asteraceae | *Symphyotrichum lanceolatum* | EU750584 |
| Angiosperm | Asteraceae | *Symphyotrichum lateriflorum* | EU750585 |
| Angiosperm | Asteraceae | *Symphyotrichum lateriflorum* | EU750586 |
| Angiosperm | Asteraceae | *Symphyotrichum lateriflorum* | EU750587 |
| Angiosperm | Asteraceae | *Symphyotrichum lateriflorum* | EU750588 |
| Angiosperm | Asteraceae | *Symphyotrichum novae-angliae* | EU750589 |
| Angiosperm | Asteraceae | *Symphyotrichum novae-angliae* | EU750590 |
| Angiosperm | Asteraceae | *Symphyotrichum novae-angliae* | EU750591 |
| Angiosperm | Asteraceae | *Symphyotrichum novae-angliae* | EU750592 |
| Angiosperm | Asteraceae | *Symphyotrichum pilosum* | EU750593 |
| Angiosperm | Asteraceae | *Symphyotrichum pilosum* | EU750594 |
| Angiosperm | Asteraceae | *Symphyotrichum urophyllum* | EU750595 |
| Angiosperm | Asteraceae | *Symphyotrichum urophyllum* | EU750596 |
| Angiosperm | Betulaceae | *Betula alleghaniensis* | EU750441 |
| Angiosperm | Betulaceae | *Betula alleghaniensis* | EU750442 |
| Angiosperm | Betulaceae | *Betula papyrifera* | EU750443 |
| Angiosperm | Betulaceae | *Betula papyrifera* | EU750444 |
| Angiosperm | Betulaceae | *Betula papyrifera* | EU750445 |
| Angiosperm | Caprifoliaceae | *Viburnum acerifolium* | EU750607 |
| Angiosperm | Caprifoliaceae | *Viburnum acerifolium* | EU750608 |
| Angiosperm | Caprifoliaceae | *Viburnum lentago* | EU750609 |
| Angiosperm | Caprifoliaceae | *Viburnum lentago* | EU750610 |
| Angiosperm | Caprifoliaceae | *Viburnum opulus* | EU750611 |
| Angiosperm | Caprifoliaceae | *Viburnum opulus* | EU750612 |
| Angiosperm | Caryophyllaceae | *Silene latifolia* | EU750546 |
| Angiosperm | Caryophyllaceae | *Silene latifolia* | EU750547 |
| Angiosperm | Caryophyllaceae | *Silene vulgaris* | EU750548 |
| Angiosperm | Caryophyllaceae | *Silene vulgaris* | EU750549 |
| Angiosperm | Caryophyllaceae | *Silene vulgaris* | EU750550 |
| Angiosperm | Combretaceae | *Combretum apiculatum* | EU213794 |
| Angiosperm | Combretaceae | *Combretum apiculatum* | EU213795 |
| Angiosperm | Combretaceae | *Combretum apiculatum* | EU213796 |
| Angiosperm | Combretaceae | *Combretum collinum* | EU213797 |
| Angiosperm | Combretaceae | *Combretum collinum* | EU213798 |
| Angiosperm | Combretaceae | *Combretum collinum* | EU213799 |
| Angiosperm | Combretaceae | *Combretum hereroense* | EU213800 |
| Angiosperm | Combretaceae | *Combretum hereroense* | EU213801 |
| Angiosperm | Combretaceae | *Combretum hereroense* | EU213802 |
| Angiosperm | Cornaceae | *Cornus alternifolia* | EU750447 |
| Angiosperm | Cornaceae | *Cornus racemosa* | EU750448 |
| Angiosperm | Cornaceae | *Cornus racemosa* | EU750449 |
| Angiosperm | Cornaceae | *Cornus rugosa* | EU750450 |
| Angiosperm | Cornaceae | *Cornus rugosa* | EU750451 |
| Angiosperm | Cornaceae | *Cornus sericea* | EU750452 |
| Angiosperm | Cornaceae | *Cornus sericea* | EU750453 |
| Angiosperm | Cornaceae | *Cornus sericea* | EU750454 |
| Angiosperm | Cornaceae | *Cornus sericea* | EU750455 |
| Angiosperm | Euphorbiaceae | *Croton gratissimus* | EU213803 |
| Angiosperm | Euphorbiaceae | *Croton gratissimus* | EU213804 |
| Angiosperm | Euphorbiaceae | *Croton gratissimus* | EU213805 |
| Angiosperm | Euphorbiaceae | *Croton megalobotrys* | EU213806 |
| Angiosperm | Euphorbiaceae | *Croton megalobotrys* | EU213807 |
| Angiosperm | Euphorbiaceae | *Croton megalobotrys* | EU213808 |
| Angiosperm | Euphorbiaceae | *Croton pseudopulchellus* | EU213809 |
| Angiosperm | Euphorbiaceae | *Croton pseudopulchellus* | EU213810 |
| Angiosperm | Euphorbiaceae | *Croton pseudopulchellus* | EU213811 |
| Angiosperm | Fabaceae | *Acacia ampliceps* | AF525003 |
| Angiosperm | Fabaceae | *Acacia exuvialis* | EU213781 |
| Angiosperm | Fabaceae | *Acacia exuvialis* | EU213782 |
| Angiosperm | Fabaceae | *Acacia exuvialis* | EU213783 |
| Angiosperm | Fabaceae | *Acacia galpinii* | AF525010 |
| Angiosperm | Fabaceae | *Acacia glomerosa* | AF525000 |
| Angiosperm | Fabaceae | *Acacia karroo* | AF524992 |
| Angiosperm | Fabaceae | *Acacia modesta* | AF524995 |
| Angiosperm | Fabaceae | *Acacia nigrescens* | EU213784 |
| Angiosperm | Fabaceae | *Acacia nigrescens* | EU213785 |
| Angiosperm | Fabaceae | *Acacia roemeriana* | AF524997 |
| Angiosperm | Fabaceae | *Acacia schweinfurthii* | AF524999 |
| Angiosperm | Fabaceae | *Acacia tortilis* | AF524994 |
| Angiosperm | Fabaceae | *Acacia tortilis* | EU213786 |
| Angiosperm | Fabaceae | *Acacia tortilis* | EU213787 |
| Angiosperm | Fabaceae | *Acacia tortilis* | EU213788 |
| Angiosperm | Fabaceae | *Acacia translucens* | AF525004 |
| Angiosperm | Fabaceae | *Albizia julibrissin* | FJ493279 |
| Angiosperm | Fabaceae | *Albizia kalkora* | AF524965 |
| Angiosperm | Fabaceae | *Anadenanthera colubrina* | AF524967 |
| Angiosperm | Fabaceae | *Caesalpinia epunctata* | DQ501965 |
| Angiosperm | Fabaceae | *Caesalpinia mimosifolia* | DQ501961 |
| Angiosperm | Fabaceae | *Caesalpinia pannosa* | DQ501959 |
| Angiosperm | Fabaceae | *Callerya megasperma* | EU424092 |
| Angiosperm | Fabaceae | *Cathormion umbellatum* | AF524968 |
| Angiosperm | Fabaceae | *Chloroleucon mangense* | AF524969 |
| Angiosperm | Fabaceae | *Desmanthus fruticosus* | EF643885 |
| Angiosperm | Fabaceae | *Dichrostachys cinerea* | AF524961 |
| Angiosperm | Fabaceae | *Enterolobium contortisiliquum* | AF52497 |
| Angiosperm | Fabaceae | *Glycyrrhiza foetida* | EU531706 |
| Angiosperm | Fabaceae | *Havardia albicans* | AF524975 |
| Angiosperm | Fabaceae | *Havardia pallens* | AF524974 |
| Angiosperm | Fabaceae | *Hoffmannseggia burchellii* subsp. *burchellii* | DQ507372 |
| Angiosperm | Fabaceae | *Hoffmannseggia burchellii* subsp. *rubroviolacea* | DQ501979 |
| Angiosperm | Fabaceae | *Inga edulis* | AF524976 |
| Angiosperm | Fabaceae | *Leucaena collinsii* subsp. *collinsii* | EF643842 |
| Angiosperm | Fabaceae | *Leucaena collinsii* subsp. *zacapana* | EF643844 |
| Angiosperm | Fabaceae | *Leucaena confertiflora* var. *confertiflora* | EF643846 |
| Angiosperm | Fabaceae | *Leucaena diversifolia* | EF643881 |
| Angiosperm | Fabaceae | *Leucaena diversifolia* | EF643882 |
| Angiosperm | Fabaceae | *Leucaena esculenta* | EF643891 |
| Angiosperm | Fabaceae | *Leucaena hybrid* | EF643850 |
| Angiosperm | Fabaceae | *Leucaena involucrata* | EF643887 |
| Angiosperm | Fabaceae | *Leucaena lanceolata* var. *lanceolata* | EF643853 |
| Angiosperm | Fabaceae | *Leucaena lanceolata* var. *sousae* | EF643857 |
| Angiosperm | Fabaceae | *Leucaena lempirana* | EF643856 |
| Angiosperm | Fabaceae | *Leucaena leucocephala* | AF524962 |
| Angiosperm | Fabaceae | *Leucaena leucocephala* subsp. *glabrata* | EF643880 |
| Angiosperm | Fabaceae | *Leucaena macrophylla* subsp. *istmensis* | EF643847 |
| Angiosperm | Fabaceae | *Leucaena magnifica* | EF643843 |
| Angiosperm | Fabaceae | *Leucaena pallida* | EF643893 |
| Angiosperm | Fabaceae | *Leucaena pueblana* | EF643888 |
| Angiosperm | Fabaceae | *Leucaena pulverulenta* | EF643865 |
| Angiosperm | Fabaceae | *Leucaena pulverulenta* | EF643872 |
| Angiosperm | Fabaceae | *Leucaena retusa* | EF643868 |
| Angiosperm | Fabaceae | *Leucaena salvadorensis* | EF643855 |
| Angiosperm | Fabaceae | *Leucaena shannonii* | EF643851 |
| Angiosperm | Fabaceae | *Leucaena trichandra* | EF643852 |
| Angiosperm | Fabaceae | *Leucaena x mixtec* | EF643862 |
| Angiosperm | Fabaceae | *Leucaena x mixtec* | EF643892 |
| Angiosperm | Fabaceae | *Leucaena x spontanea* | EF643860 |
| Angiosperm | Fabaceae | *Lysiloma acapulcense* | AF524977 |
| Angiosperm | Fabaceae | *Lysiloma tergeminum* | AF524978 |
| Angiosperm | Fabaceae | *Mariosousa coulteri* | AF525008 |
| Angiosperm | Fabaceae | *Mariosousa dolichostachya* | AF525009 |
| Angiosperm | Fabaceae | *Microlobius foetidus* | AF524979 |
| Angiosperm | Fabaceae | *Mimosa tenuiflora* | AF524963 |
| Angiosperm | Fabaceae | *Pararchidendron pruinosum* | AF524980 |
| Angiosperm | Fabaceae | *Piptadenia viridiflora* | AF524982 |
| Angiosperm | Fabaceae | *Poincianella mexicana* | DQ501958 |
| Angiosperm | Fabaceae | *Pomaria brachycarpa* | DQ501976 |
| Angiosperm | Fabaceae | *Pomaria canescens* | DQ501966 |
| Angiosperm | Fabaceae | *Pomaria fruticosa* | DQ501971 |
| Angiosperm | Fabaceae | *Pomaria lactea* | DQ501970 |
| Angiosperm | Fabaceae | *Pomaria melanosticta* | DQ501969 |
| Angiosperm | Fabaceae | *Pomaria rubicunda* var. *hauthalii* | DQ501978 |
| Angiosperm | Fabaceae | *Pomaria stipularis* | DQ501968 |
| Angiosperm | Fabaceae | *Pomaria wootonii* | DQ501963 |
| Angiosperm | Fabaceae | *Pseudosamanea guachapele* | AF524983 |
| Angiosperm | Fabaceae | *Schizolobium parahyba* | AF524985 |
| Angiosperm | Fabaceae | *Senegalia visco* | AF525002 |
| Angiosperm | Fabaceae | *Trifolium pratense* | EU750597 |
| Angiosperm | Fabaceae | *Trifolium pratense* | EU750598 |
| Angiosperm | Fabaceae | *Trifolium pratense* | EU750599 |
| Angiosperm | Fabaceae | *Trifolium repens* | EU750600 |
| Angiosperm | Fabaceae | *Trifolium repens* | EU750601 |
| Angiosperm | Fabaceae | *Trifolium repens* | EU750602 |
| Angiosperm | Fabaceae | *Vachellia constricta* | AF524989 |
| Angiosperm | Fabaceae | *Wisteria brachybotrys* | EU424093 |
| Angiosperm | Fabaceae | *Wisteria brachybotrys* | EU424094 |
| Angiosperm | Fabaceae | *Wisteria floribunda* | EU424096 |
| Angiosperm | Fabaceae | *Wisteria floribunda* | EU424097 |
| Angiosperm | Fabaceae | *Wisteria floribunda* | EU424098 |
| Angiosperm | Fabaceae | *Wisteria frutescens* | EU424099 |
| Angiosperm | Fabaceae | *Wisteria frutescens* | EU424100 |
| Angiosperm | Fabaceae | *Wisteria frutescens* var. *macrostachya* | EU424101 |
| Angiosperm | Fabaceae | *Wisteria sinensis* | EU424102 |
| Angiosperm | Fabaceae | *Wisteria sinensis* | EU424103 |
| Angiosperm | Fabaceae | *Wisteria sinensis* | EU424104 |
| Angiosperm | Fabaceae | *Wisteria villosa* | EU424105 |
| Angiosperm | Fabaceae | *Wisteria villosa* | EU424106 |
| Angiosperm | Fabaceae | *Wisteria villosa* | EU424107 |
| Angiosperm | Fabaceae | *Zapoteca tetragona* | AF524986 |
| Angiosperm | Fagaceae | *Quercus alba* | EU750506 |
| Angiosperm | Fagaceae | *Quercus alba* | EU750507 |
| Angiosperm | Fagaceae | *Quercus alba* | EU750508 |
| Angiosperm | Fagaceae | *Quercus alba* | EU750509 |
| Angiosperm | Fagaceae | *Quercus cf. velutina* | EU750510 |
| Angiosperm | Fagaceae | *Quercus macrocarpa* | EU750511 |
| Angiosperm | Fagaceae | *Quercus macrocarpa* | EU750512 |
| Angiosperm | Fagaceae | *Quercus rubra* | EU750513 |
| Angiosperm | Fagaceae | *Quercus rubra* | EU750514 |
| Angiosperm | Fagaceae | *Quercus rubra* | EU750515 |
| Angiosperm | Gramineae | *Arundinaria gigantea* | FJ644249 |
| Angiosperm | Gramineae | *Arundinaria tecta* | FJ644250 |
| Angiosperm | Hernandiaceae | *Hernandia guianensis* | AF261993 |
| Angiosperm | Hernandiaceae | *Hernandia nymphaeifolia* | AF261994 |
| Angiosperm | Lauraceae | *Actinodaphne sesquipedalis* | AF268787 |
| Angiosperm | Lauraceae | *Aiouea dubia* | EU153942 |
| Angiosperm | Lauraceae | *Aiouea guianensis* | AF268780 |
| Angiosperm | Lauraceae | *Alseodaphne semecarpifolia* | AF268799 |
| Angiosperm | Lauraceae | *Anaueria brasiliensis* | AF268800 |
| Angiosperm | Lauraceae | *Aniba cinnamomiflora* | AF268770 |
| Angiosperm | Lauraceae | *Aniba hypoglauca* | AF268771 |
| Angiosperm | Lauraceae | *Beilschmiedia brenesii* | AF268809 |
| Angiosperm | Lauraceae | *Beilschmiedia madagascariensis* | AF268810 |
| Angiosperm | Lauraceae | *Beilschmiedia ovalis* | AF268811 |
| Angiosperm | Lauraceae | *Beilschmiedia pendula* | EU153943 |
| Angiosperm | Lauraceae | *Beilschmiedia pendula* | EU153945 |
| Angiosperm | Lauraceae | *Beilschmiedia sary* | AF268812 |
| Angiosperm | Lauraceae | *Beilschmiedia tawa* | EU153946 |
| Angiosperm | Lauraceae | *Beilschmiedia velutina* | AF268813 |
| Angiosperm | Lauraceae | *Caryodaphnopsis bilocellata* | AF261995 |
| Angiosperm | Lauraceae | *Caryodaphnopsis cogolloi* | EU153947 |
| Angiosperm | Lauraceae | *Caryodaphnopsis tomentosa* | AF268807 |
| Angiosperm | Lauraceae | *Chlorocardium rodiei* | AF268802 |
| Angiosperm | Lauraceae | *Chlorocardium venenosum* | AF268801 |
| Angiosperm | Lauraceae | *Cinnamomum bejolghota* | EU153949 |
| Angiosperm | Lauraceae | *Cinnamomum camphora* | AB331294 |
| Angiosperm | Lauraceae | *Cinnamomum camphora* | EU153948 |
| Angiosperm | Lauraceae | *Cinnamomum japonicum* | AF268782 |
| Angiosperm | Lauraceae | *Cinnamomum quadrangulum* | AF268781 |
| Angiosperm | Lauraceae | *Cinnamomum triplinerve* | EU153950 |
| Angiosperm | Lauraceae | *Cinnamomum triplinerve* | EU153951 |
| Angiosperm | Lauraceae | *Cinnamomum triplinerve* | EU153952 |
| Angiosperm | Lauraceae | *Cinnamomum verum* | AF268784 |
| Angiosperm | Lauraceae | *Cryptocarya rhodosperma* | AF268817 |
| Angiosperm | Lauraceae | *Cryptocarya sclerophylla* | AF268818 |
| Angiosperm | Lauraceae | *Cryptocarya thouvenotii* | AF261997 |
| Angiosperm | Lauraceae | *Dicypellium manausense* | AF268775 |
| Angiosperm | Lauraceae | *Endiandra microneura* | AF268814 |
| Angiosperm | Lauraceae | *Endlicheria chalisea* | AF268756 |
| Angiosperm | Lauraceae | *Endlicheria citriodora* | AF268757 |
| Angiosperm | Lauraceae | *Endlicheria reflectens* | AF268758 |
| Angiosperm | Lauraceae | *Eusideroxylon zwageri* | AF268820 |
| Angiosperm | Lauraceae | *Kubitzkia mezii* | AF268772 |
| Angiosperm | Lauraceae | *Laurus azorica* | EU153958 |
| Angiosperm | Lauraceae | *Laurus nobilis* | AF268785 |
| Angiosperm | Lauraceae | *Laurus nobilis* | EU153959 |
| Angiosperm | Lauraceae | *Laurus nobilis* | FJ493285 |
| Angiosperm | Lauraceae | *Licaria cannella* | AF268773 |
| Angiosperm | Lauraceae | *Licaria triandra* | AF268774 |
| Angiosperm | Lauraceae | *Lindera benzoin* | AF268788 |
| Angiosperm | Lauraceae | *Lindera benzoin* | EF491227 |
| Angiosperm | Lauraceae | *Lindera benzoin* | EU153960 |
| Angiosperm | Lauraceae | *Lindera umbellata* | AF268789 |
| Angiosperm | Lauraceae | *Litsea coreana* | AF268791 |
| Angiosperm | Lauraceae | *Litsea cubeba* | EU153961 |
| Angiosperm | Lauraceae | *Litsea glaucescens* | AF129063 |
| Angiosperm | Lauraceae | *Litsea krukovii* | AB331293 |
| Angiosperm | Lauraceae | *Mezilaurus triunca* | AF268804 |
| Angiosperm | Lauraceae | *Nectandra cissiflora* | EU153962 |
| Angiosperm | Lauraceae | *Nectandra cissiflora* | EU153963 |
| Angiosperm | Lauraceae | *Nectandra cissiflora* | EU153964 |
| Angiosperm | Lauraceae | *Nectandra cissiflora* | EU153965 |
| Angiosperm | Lauraceae | *Nectandra cuspidata* | EU153966 |
| Angiosperm | Lauraceae | *Nectandra cuspidata* | EU153967 |
| Angiosperm | Lauraceae | *Nectandra lineata* | EU153970 |
| Angiosperm | Lauraceae | *Nectandra lineata* | EU153971 |
| Angiosperm | Lauraceae | *Nectandra membranacea* | AF268767 |
| Angiosperm | Lauraceae | *Nectandra purpurea* | EU153972 |
| Angiosperm | Lauraceae | *Nectandra purpurea* | EU153973 |
| Angiosperm | Lauraceae | *Nectandra purpurea* | EU153974 |
| Angiosperm | Lauraceae | *Nectandra turbacensis* | AF268768 |
| Angiosperm | Lauraceae | *Neocinnamomum mekongense* | AF268806 |
| Angiosperm | Lauraceae | *Neolitsea aciculata* | EU153977 |
| Angiosperm | Lauraceae | *Neolitsea sericea* | AF268792 |
| Angiosperm | Lauraceae | *Ocotea bullata* | AF268778 |
| Angiosperm | Lauraceae | *Ocotea calophylla* | EU153978 |
| Angiosperm | Lauraceae | *Ocotea cernua* | EU153979 |
| Angiosperm | Lauraceae | *Ocotea cernua* | EU153980 |
| Angiosperm | Lauraceae | *Ocotea cernua* | EU153981 |
| Angiosperm | Lauraceae | *Ocotea floribunda* | EU153982 |
| Angiosperm | Lauraceae | *Ocotea guianensis* | AF268761 |
| Angiosperm | Lauraceae | *Ocotea guianensis* | EU153983 |
| Angiosperm | Lauraceae | *Ocotea leucoxylon* | AF268763 |
| Angiosperm | Lauraceae | *Ocotea malcomberi* | AF268779 |
| Angiosperm | Lauraceae | *Ocotea oblonga* | EU153984 |
| Angiosperm | Lauraceae | *Ocotea odorifera* | AF268762 |
| Angiosperm | Lauraceae | *Ocotea pauciflora* | AF268764 |
| Angiosperm | Lauraceae | *Ocotea puberula* | EU153985 |
| Angiosperm | Lauraceae | *Ocotea puberula* | EU153986 |
| Angiosperm | Lauraceae | *Ocotea quixos* | AF261999 |
| Angiosperm | Lauraceae | *Ocotea rhynchophylla* | AF268766 |
| Angiosperm | Lauraceae | *Ocotea tomentella* | AF268765 |
| Angiosperm | Lauraceae | *Ocotea whitei* | EU153988 |
| Angiosperm | Lauraceae | *Parasassafras confertiflora* | AF268790 |
| Angiosperm | Lauraceae | *Persea americana* | AF268794 |
| Angiosperm | Lauraceae | *Persea americana* | EU153989 |
| Angiosperm | Lauraceae | *Persea caerulea* | AF268795 |
| Angiosperm | Lauraceae | *Persea caerulea* | EU153990 |
| Angiosperm | Lauraceae | *Persea lingue* | AF268796 |
| Angiosperm | Lauraceae | *Persea meridensis* | AF268797 |
| Angiosperm | Lauraceae | *Persea thunbergii* | AF268798 |
| Angiosperm | Lauraceae | *Pleurothyrium cinereum* | AF268769 |
| Angiosperm | Lauraceae | *Potameia micrantha* | AF268815 |
| Angiosperm | Lauraceae | *Potameia microphylla* | AF268816 |
| Angiosperm | Lauraceae | *Potoxylon melagangai* | AF268821 |
| Angiosperm | Lauraceae | *Rhodostemonodaphne crenaticupula* | AF268759 |
| Angiosperm | Lauraceae | *Rhodostemonodaphne kunthiana* | EU153991 |
| Angiosperm | Lauraceae | *Rhodostemonodaphne penduliflora* | EU153992 |
| Angiosperm | Lauraceae | *Rhodostemonodaphne praeclara* | AF268760 |
| Angiosperm | Lauraceae | *Sassafras albidum* | AF268793 |
| Angiosperm | Lauraceae | *Sassafras albidum* | EF491223 |
| Angiosperm | Lauraceae | *Sassafras albidum* | EF491224 |
| Angiosperm | Lauraceae | *Sassafras albidum* | EF491225 |
| Angiosperm | Lauraceae | *Sassafras albidum* | EF491226 |
| Angiosperm | Lauraceae | *Sassafras albidum* | EU153993 |
| Angiosperm | Lauraceae | *Sassafras randaiense* | EF491221 |
| Angiosperm | Lauraceae | *Sassafras randaiense* | EF491222 |
| Angiosperm | Lauraceae | *Sassafras tzumu* | EF491217 |
| Angiosperm | Lauraceae | *Sassafras tzumu* | EF491218 |
| Angiosperm | Lauraceae | *Sassafras tzumu* | EF491219 |
| Angiosperm | Lauraceae | *Sassafras tzumu* | EF491220 |
| Angiosperm | Lauraceae | *Sextonia pubescens* | AF262000 |
| Angiosperm | Lauraceae | *Sextonia rubra* | AF268805 |
| Angiosperm | Lauraceae | *Umbellularia californica* | AF268777 |
| Angiosperm | Liliaceae | *Lilium philadelphicum* | DQ122704 |
| Angiosperm | Liliaceae | *Lilium philadelphicum* | DQ122705 |
| Angiosperm | Liliaceae | *Lilium philadelphicum* | DQ122706 |
| Angiosperm | Liliaceae | *Lilium philadelphicum* | DQ122707 |
| Angiosperm | Liliaceae | *Lilium philadelphicum* | DQ122708 |
| Angiosperm | Liliaceae | *Lilium philadelphicum* | DQ122709 |
| Angiosperm | Liliaceae | *Lilium philadelphicum* | DQ122710 |
| Angiosperm | Liliaceae | *Lilium philadelphicum* | DQ122711 |
| Angiosperm | Liliaceae | *Lilium philadelphicum* | DQ122712 |
| Angiosperm | Liliaceae | *Lilium philadelphicum* | DQ122713 |
| Angiosperm | Loganiaceae | *Strychnos decussata* | EU213857 |
| Angiosperm | Loganiaceae | *Strychnos decussata* | EU213858 |
| Angiosperm | Loganiaceae | *Strychnos decussata* | EU213859 |
| Angiosperm | Loganiaceae | *Strychnos madagascariensis* | EU213860 |
| Angiosperm | Loganiaceae | *Strychnos madagascariensis* | EU213861 |
| Angiosperm | Loganiaceae | *Strychnos madagascariensis* | EU213862 |
| Angiosperm | Loganiaceae | *Strychnos spinosa* | EU213863 |
| Angiosperm | Loganiaceae | *Strychnos spinosa* | EU213864 |
| Angiosperm | Loganiaceae | *Strychnos spinosa* | EU213865 |
| Angiosperm | Magnoliaceae | *Elmerrillia ovalis* | AY009014 |
| Angiosperm | Magnoliaceae | *Kmeria duperreana* | AY009036 |
| Angiosperm | Magnoliaceae | *Kmeria septentrionalis* | AY009037 |
| Angiosperm | Magnoliaceae | *Liriodendron chinense* | AB021046 |
| Angiosperm | Magnoliaceae | *Liriodendron tulipifera* | AB021047 |
| Angiosperm | Magnoliaceae | *Liriodendron tulipifera* | AY727182 |
| Angiosperm | Magnoliaceae | *Liriodendron tulipifera* | FJ493286 |
| Angiosperm | Magnoliaceae | *Magnolia acuminata* | AB021041 |
| Angiosperm | Magnoliaceae | *Magnolia acuminata* | AY727183 |
| Angiosperm | Magnoliaceae | *Magnolia biondii* | AY009017 |
| Angiosperm | Magnoliaceae | *Magnolia campbellii* | AY009019 |
| Angiosperm | Magnoliaceae | *Magnolia cathcartii* | AY009015 |
| Angiosperm | Magnoliaceae | *Magnolia coco* | AB021034 |
| Angiosperm | Magnoliaceae | *Magnolia cylindrica* | AY009020 |
| Angiosperm | Magnoliaceae | *Magnolia dawsoniana* | AY009018 |
| Angiosperm | Magnoliaceae | *Magnolia dealbata* | AB055556 |
| Angiosperm | Magnoliaceae | *Magnolia dealbata* | AY009038 |
| Angiosperm | Magnoliaceae | *Magnolia delavayi* | AB021035 |
| Angiosperm | Magnoliaceae | *Magnolia denudata* | AB021037 |
| Angiosperm | Magnoliaceae | *Magnolia dodecapetala* | AB055560 |
| Angiosperm | Magnoliaceae | *Magnolia dodecapetala* | AY009035 |
| Angiosperm | Magnoliaceae | *Magnolia elegans* | AY009016 |
| Angiosperm | Magnoliaceae | *Magnolia fraseri* var. *fraseri* | AB021025 |
| Angiosperm | Magnoliaceae | *Magnolia grandiflora* | AB021020 |
| Angiosperm | Magnoliaceae | *Magnolia grandiflora* | FJ493287 |
| Angiosperm | Magnoliaceae | *Magnolia guatemalensis* | AB021021 |
| Angiosperm | Magnoliaceae | *Magnolia henryi* | AY009027 |
| Angiosperm | Magnoliaceae | *Magnolia iltisiana* | AB055551 |
| Angiosperm | Magnoliaceae | *Magnolia kobus* | AB021038 |
| Angiosperm | Magnoliaceae | *Magnolia liliifera* | AY009028 |
| Angiosperm | Magnoliaceae | *Magnolia liliifera* var. *obovata* | AB021043 |
| Angiosperm | Magnoliaceae | *Magnolia liliiflora* | AB021042 |
| Angiosperm | Magnoliaceae | *Magnolia macrophylla* | AB021027 |
| Angiosperm | Magnoliaceae | *Magnolia macrophylla* | AB021028 |
| Angiosperm | Magnoliaceae | *Magnolia mexicana* | AB055562 |
| Angiosperm | Magnoliaceae | *Magnolia minor* | AB055561 |
| Angiosperm | Magnoliaceae | *Magnolia nitida* | AB021036 |
| Angiosperm | Magnoliaceae | *Magnolia obovata* | AB021029 |
| Angiosperm | Magnoliaceae | *Magnolia officinalis* | AY009031 |
| Angiosperm | Magnoliaceae | *Magnolia officinalis* subsp. *biloba* | AB021030 |
| Angiosperm | Magnoliaceae | *Magnolia ovata* | AB055563 |
| Angiosperm | Magnoliaceae | *Magnolia pacifica* subsp. *pugana* | AB055552 |
| Angiosperm | Magnoliaceae | *Magnolia panamensis* | AY009026 |
| Angiosperm | Magnoliaceae | *Magnolia portoricensis* | AB055554 |
| Angiosperm | Magnoliaceae | *Magnolia pterocarpa* | AY009029 |
| Angiosperm | Magnoliaceae | *Magnolia pyramidata* | AB021026 |
| Angiosperm | Magnoliaceae | *Magnolia salicifolia* | AB021039 |
| Angiosperm | Magnoliaceae | *Magnolia sargentiana* | AB055559 |
| Angiosperm | Magnoliaceae | *Magnolia schiedeana* | AB055568 |
| Angiosperm | Magnoliaceae | *Magnolia sharpii* | AB021023 |
| Angiosperm | Magnoliaceae | *Magnolia sieboldii* subsp. *japonica* | AB021033 |
| Angiosperm | Magnoliaceae | *Magnolia sieboldii* subsp. *sieboldii* | AB021032 |
| Angiosperm | Magnoliaceae | *Magnolia sieboldii* subsp. *sinensis* | AB055557 |
| Angiosperm | Magnoliaceae | *Magnolia sinica* | AY009021 |
| Angiosperm | Magnoliaceae | *Magnolia splendens* | AB055555 |
| Angiosperm | Magnoliaceae | *Magnolia splendens* | AY009033 |
| Angiosperm | Magnoliaceae | *Magnolia stellata* | AB021040 |
| Angiosperm | Magnoliaceae | *Magnolia tamaulipana* | AB021024 |
| Angiosperm | Magnoliaceae | *Magnolia tripetala* | AB021031 |
| Angiosperm | Magnoliaceae | *Magnolia virginiana* | AB021018 |
| Angiosperm | Magnoliaceae | *Magnolia yoroconte* | AB055553 |
| Angiosperm | Magnoliaceae | *Manglietia aromatica* | AY009023 |
| Angiosperm | Magnoliaceae | *Manglietia conifera* | AB055564 |
| Angiosperm | Magnoliaceae | *Manglietia decidua* | AB055565 |
| Angiosperm | Magnoliaceae | *Manglietia glauca* | AY009025 |
| Angiosperm | Magnoliaceae | *Manglietia grandis* | AY009022 |
| Angiosperm | Magnoliaceae | *Manglietia insignis* | AB055566 |
| Angiosperm | Magnoliaceae | *Michelia baillonii* | AY009012 |
| Angiosperm | Magnoliaceae | *Michelia cavaleriei* | AY009009 |
| Angiosperm | Magnoliaceae | *Michelia champaca* | AY009011 |
| Angiosperm | Magnoliaceae | *Michelia compressa* | AB021044 |
| Angiosperm | Magnoliaceae | *Michelia macclurei* | AB055567 |
| Angiosperm | Magnoliaceae | *Michelia odora* | AY009013 |
| Angiosperm | Moraceae | *Ficus abutilifolia* | EU213819 |
| Angiosperm | Moraceae | *Ficus abutilifolia* | EU213820 |
| Angiosperm | Moraceae | *Ficus abutilifolia* | EU213821 |
| Angiosperm | Moraceae | *Ficus glumosa* | EU213822 |
| Angiosperm | Moraceae | *Ficus glumosa* | EU213823 |
| Angiosperm | Moraceae | *Ficus glumosa* | EU213824 |
| Angiosperm | Moraceae | *Ficus sycomorus* | EU213825 |
| Angiosperm | Moraceae | *Ficus sycomorus* | EU213826 |
| Angiosperm | Moraceae | *Ficus sycomorus* | EU213827 |
| Angiosperm | Myristicaceae | *Compsoneura atopa* | EU090622 |
| Angiosperm | Myristicaceae | *Compsoneura capitellata* | EU090623 |
| Angiosperm | Myristicaceae | *Compsoneura capitellata* | EU090624 |
| Angiosperm | Myristicaceae | *Compsoneura capitellata* | EU090625 |
| Angiosperm | Myristicaceae | *Compsoneura capitellata* | EU090626 |
| Angiosperm | Myristicaceae | *Compsoneura capitellata* | EU090627 |
| Angiosperm | Myristicaceae | *Compsoneura debilis* | EU090628 |
| Angiosperm | Myristicaceae | *Compsoneura debilis* | EU090629 |
| Angiosperm | Myristicaceae | *Compsoneura debilis* | EU090630 |
| Angiosperm | Myristicaceae | *Compsoneura debilis* | EU090631 |
| Angiosperm | Myristicaceae | *Compsoneura excelsa* | EU090632 |
| Angiosperm | Myristicaceae | *Compsoneura excelsa* | EU090633 |
| Angiosperm | Myristicaceae | *Compsoneura excelsa* | EU090634 |
| Angiosperm | Myristicaceae | *Compsoneura mexicana* | EU090637 |
| Angiosperm | Myristicaceae | *Compsoneura mexicana* | EU090638 |
| Angiosperm | Myristicaceae | *Compsoneura mexicana* | EU090639 |
| Angiosperm | Myristicaceae | *Compsoneura mutisii* | EU090646 |
| Angiosperm | Myristicaceae | *Compsoneura mutisii* | EU090647 |
| Angiosperm | Myristicaceae | *Compsoneura mutisii* | EU090648 |
| Angiosperm | Myristicaceae | *Compsoneura mutisii* | EU090649 |
| Angiosperm | Myristicaceae | *Compsoneura mutisii* | EU090650 |
| Angiosperm | Myristicaceae | *Compsoneura sprucei* | EU090651 |
| Angiosperm | Myristicaceae | *Compsoneura sprucei* | EU090652 |
| Angiosperm | Myristicaceae | *Compsoneura sprucei* | EU090653 |
| Angiosperm | Myristicaceae | *Compsoneura sprucei* | EU090654 |
| Angiosperm | Myristicaceae | *Compsoneura sprucei* | EU090655 |
| Angiosperm | Myristicaceae | *Compsoneura sprucei* | EU090656 |
| Angiosperm | Myristicaceae | *Compsoneura ulei* | EU090657 |
| Angiosperm | Myristicaceae | *Compsoneura ulei* | EU090658 |
| Angiosperm | Myristicaceae | *Compsoneura ulei* | EU090659 |
| Angiosperm | Myristicaceae | *Iryanthera lancifolia* | EU090660 |
| Angiosperm | Myrothamnaceae | *Myrothamnus flabellifolia* | EU213839 |
| Angiosperm | Myrothamnaceae | *Myrothamnus flabellifolia* | EU213840 |
| Angiosperm | Myrothamnaceae | *Myrothamnus flabellifolia* | EU213841 |
| Angiosperm | Orchidaceae | *Aa paleacea* | EU213704 |
| Angiosperm | Orchidaceae | *Acampe praemorsa* | EU213789 |
| Angiosperm | Orchidaceae | *Acostaea costaricensis* | EU213705 |
| Angiosperm | Orchidaceae | *Ada chlorops* | EU213706 |
| Angiosperm | Orchidaceae | *Arpophyllum giganteum* | EU213708 |
| Angiosperm | Orchidaceae | *Aulosepalum hemichreum* | AM884889 |
| Angiosperm | Orchidaceae | *Aulosepalum oestlundii* | AM884886 |
| Angiosperm | Orchidaceae | *Aulosepalum oestlundii* | AM884887 |
| Angiosperm | Orchidaceae | *Aulosepalum pyramidale* | AM884884 |
| Angiosperm | Orchidaceae | *Aulosepalum ramentaceum* | AM884882 |
| Angiosperm | Orchidaceae | *Aulosepalum tenuiflorum* | AM884891 |
| Angiosperm | Orchidaceae | *Barbosella prorepens* | EU213709 |
| Angiosperm | Orchidaceae | *Bonatea speciosa* | EU213790 |
| Angiosperm | Orchidaceae | *Brassavola nodosa* | EU213710 |
| Angiosperm | Orchidaceae | *Brassia arcuigera* | EU213711 |
| Angiosperm | Orchidaceae | *Cattleya aurantiaca* | EU213712 |
| Angiosperm | Orchidaceae | *Cattleya patinii* | EU213713 |
| Angiosperm | Orchidaceae | *Cattleya skinneri* | EU213714 |
| Angiosperm | Orchidaceae | *Clowesia warczewiczii* | EU213715 |
| Angiosperm | Orchidaceae | *Clowesia warczewiczii* | EU213716 |
| Angiosperm | Orchidaceae | *Clowesia warczewiczii* | EU213717 |
| Angiosperm | Orchidaceae | *Clowesia warczewiczii* | EU213718 |
| Angiosperm | Orchidaceae | *Clowesia warczewiczii* | EU213719 |
| Angiosperm | Orchidaceae | *Clowesia warczewiczii* | EU213720 |
| Angiosperm | Orchidaceae | *Clowesia warczewiczii* | EU213721 |
| Angiosperm | Orchidaceae | *Coeliopsis hyacinthosma* | EU213723 |
| Angiosperm | Orchidaceae | *Coryanthes horichiana* | EU213724 |
| Angiosperm | Orchidaceae | *Coryanthes speciosa* | EU213725 |
| Angiosperm | Orchidaceae | *Cranichis ciliilabia* | EU213726 |
| Angiosperm | Orchidaceae | *Cyclopogon comosus* | EU213727 |
| Angiosperm | Orchidaceae | *Cycnoches egertonianum* | EU213728 |
| Angiosperm | Orchidaceae | *Dendrobium aphyllum* | FJ385772 |
| Angiosperm | Orchidaceae | *Dendrobium cariniferum* | EU672793 |
| Angiosperm | Orchidaceae | *Dendrobium chrysotoxum* | EU672792 |
| Angiosperm | Orchidaceae | *Dendrobium fimbriatum* | EU672798 |
| Angiosperm | Orchidaceae | *Dendrobium hancockii* | EU672800 |
| Angiosperm | Orchidaceae | *Dendrobium loddigesii* | EU881986 |
| Angiosperm | Orchidaceae | *Dendrobium moniliforme* | EU672796 |
| Angiosperm | Orchidaceae | *Dendrobium nobile* | EU672797 |
| Angiosperm | Orchidaceae | *Dendrobium thyrsiflorum* | EU672799 |
| Angiosperm | Orchidaceae | *Dendrobium tosaense* | EU672794 |
| Angiosperm | Orchidaceae | *Dendrobium williamsonii* | EU672795 |
| Angiosperm | Orchidaceae | *Dichaea muricata* | EU213729 |
| Angiosperm | Orchidaceae | *Diodonopsis erinacea* | EU213749 |
| Angiosperm | Orchidaceae | *Diodonopsis erinacea* | EU213750 |
| Angiosperm | Orchidaceae | *Encyclia alata* | EU213730 |
| Angiosperm | Orchidaceae | *Encyclia alata* | EU213731 |
| Angiosperm | Orchidaceae | *Encyclia cordigera* | EU213732 |
| Angiosperm | Orchidaceae | *Encyclia cordigera* | EU213733 |
| Angiosperm | Orchidaceae | *Encyclia cordigera* | EU213734 |
| Angiosperm | Orchidaceae | *Encyclia cordigera* | EU213735 |
| Angiosperm | Orchidaceae | *Epidendrum pseudepidendrum* | EU213736 |
| Angiosperm | Orchidaceae | *Erycina crista-galli* | EU213751 |
| Angiosperm | Orchidaceae | *Erycina pumilio* | EU213737 |
| Angiosperm | Orchidaceae | *Erycina pumilio* | EU213738 |
| Angiosperm | Orchidaceae | *Galeandra dives* | EU213740 |
| Angiosperm | Orchidaceae | *Gongora amparoana* | EU213741 |
| Angiosperm | Orchidaceae | *Gongora cornuta* | EU213742 |
| Angiosperm | Orchidaceae | *Gongora horichiana* | EU213743 |
| Angiosperm | Orchidaceae | *Isochilus amparoanus* | EU213744 |
| Angiosperm | Orchidaceae | *Kegeliella atropilosa* | EU213745 |
| Angiosperm | Orchidaceae | *Lockhartia oerstedii* | EU213746 |
| Angiosperm | Orchidaceae | *Lycaste macrophylla* | EU213747 |
| Angiosperm | Orchidaceae | *Malaxis steyermarkii* | EU213748 |
| Angiosperm | Orchidaceae | *Oncidium obryzatoides* | EU213752 |
| Angiosperm | Orchidaceae | *Oncidium storkii* | EU213753 |
| Angiosperm | Orchidaceae | *Ophrys aegirtica* | AM711641 |
| Angiosperm | Orchidaceae | *Ophrys apifera* | AM711642 |
| Angiosperm | Orchidaceae | *Ophrys atlantica* | AM711644 |
| Angiosperm | Orchidaceae | *Ophrys attica* | AM711645 |
| Angiosperm | Orchidaceae | *Ophrys basilissa* | AM711649 |
| Angiosperm | Orchidaceae | *Ophrys bilunulata* | AM711651 |
| Angiosperm | Orchidaceae | *Ophrys biscutella* | AM711652 |
| Angiosperm | Orchidaceae | *Ophrys bornmuelleri* | AM711654 |
| Angiosperm | Orchidaceae | *Ophrys bremifera* | AM711655 |
| Angiosperm | Orchidaceae | *Ophrys cinereophila* | AM711660 |
| Angiosperm | Orchidaceae | *Ophrys drumana* | AM711663 |
| Angiosperm | Orchidaceae | *Ophrys funerea* | AM711669 |
| Angiosperm | Orchidaceae | *Ophrys garganica* | AM711671 |
| Angiosperm | Orchidaceae | *Ophrys gortynia* | AM711672 |
| Angiosperm | Orchidaceae | *Ophrys gracilis* | AM711673 |
| Angiosperm | Orchidaceae | *Ophrys mammosa* | AM711681 |
| Angiosperm | Orchidaceae | *Ophrys melena* | AM711682 |
| Angiosperm | Orchidaceae | *Ophrys minutula* | AM711683 |
| Angiosperm | Orchidaceae | *Ophrys omegaifera* | AM711685 |
| Angiosperm | Orchidaceae | *Ophrys oxyrrhynchos* | AM711686 |
| Angiosperm | Orchidaceae | *Ophrys pallida* | AM711687 |
| Angiosperm | Orchidaceae | *Ophrys parosica* | AM711689 |
| Angiosperm | Orchidaceae | *Ophrys phryganae* | AM711691 |
| Angiosperm | Orchidaceae | *Ophrys regis-ferdinandii* | AM711692 |
| Angiosperm | Orchidaceae | *Ophrys reinholdii* | AM711693 |
| Angiosperm | Orchidaceae | *Ophrys sicula* | AM711696 |
| Angiosperm | Orchidaceae | *Ophrys sitiaca* | AM711697 |
| Angiosperm | Orchidaceae | *Ophrys sphegifera* | AM711698 |
| Angiosperm | Orchidaceae | *Ophrys vasconica* | AM711705 |
| Angiosperm | Orchidaceae | *Phalaenopsis amabilis* | FJ460367 |
| Angiosperm | Orchidaceae | *Phalaenopsis amabilis* | FJ460368 |
| Angiosperm | Orchidaceae | *Phalaenopsis amabilis* | FJ460373 |
| Angiosperm | Orchidaceae | *Phalaenopsis amabilis* | FJ460374 |
| Angiosperm | Orchidaceae | *Phalaenopsis amabilis* | FJ460388 |
| Angiosperm | Orchidaceae | *Phalaenopsis amabilis* | FJ460389 |
| Angiosperm | Orchidaceae | *Phalaenopsis amabilis* subsp. *rosenstromii* | FJ460372 |
| Angiosperm | Orchidaceae | *Phalaenopsis aphrodite* | FJ460384 |
| Angiosperm | Orchidaceae | *Phalaenopsis aphrodite* | FJ460401 |
| Angiosperm | Orchidaceae | *Phalaenopsis aphrodite* | FJ460402 |
| Angiosperm | Orchidaceae | *Phalaenopsis aphrodite* | FJ460403 |
| Angiosperm | Orchidaceae | *Phalaenopsis aphrodite* subsp. *formosana* | FJ460382 |
| Angiosperm | Orchidaceae | *Phalaenopsis aphrodite* subsp. *formosana* | FJ460383 |
| Angiosperm | Orchidaceae | *Phalaenopsis aphrodite* subsp. *formosana* | FJ460385 |
| Angiosperm | Orchidaceae | *Phalaenopsis aphrodite* subsp. *formosana* | FJ460386 |
| Angiosperm | Orchidaceae | *Phalaenopsis sanderiana* | FJ460366 |
| Angiosperm | Orchidaceae | *Phalaenopsis sanderiana* | FJ460381 |
| Angiosperm | Orchidaceae | *Ponthieva racemosa* | EU213754 |
| Angiosperm | Orchidaceae | *Ponthieva racemosa* | EU213755 |
| Angiosperm | Orchidaceae | *Ponthieva racemosa* | EU213756 |
| Angiosperm | Orchidaceae | *Prosthechea cochleata* | EU213757 |
| Angiosperm | Orchidaceae | *Prosthechea cochleata* | EU213758 |
| Angiosperm | Orchidaceae | *Prosthechea fragrans* | EU213759 |
| Angiosperm | Orchidaceae | *Prosthechea fragrans* | EU213760 |
| Angiosperm | Orchidaceae | *Prosthechea radiata* | EU213761 |
| Angiosperm | Orchidaceae | *Prosthechea radiata* | EU213762 |
| Angiosperm | Orchidaceae | *Prosthechea radiata* | EU213763 |
| Angiosperm | Orchidaceae | *Rhynchostele beloglossa* | EU213707 |
| Angiosperm | Orchidaceae | *Rhynchostele bictoniensis* | EU213764 |
| Angiosperm | Orchidaceae | *Sarcoglottis smithii* | EU213765 |
| Angiosperm | Orchidaceae | *Stanhopea saccata* | EU213766 |
| Angiosperm | Orchidaceae | *Stanhopea saccata* | EU213767 |
| Angiosperm | Orchidaceae | *Stanhopea saccata* | EU213768 |
| Angiosperm | Orchidaceae | *Trichopilia tortilis* | EU213769 |
| Angiosperm | Orchidaceae | *Trichopilia tortilis* | EU213770 |
| Angiosperm | Orchidaceae | *Trichopilia tortilis* | EU213771 |
| Angiosperm | Orchidaceae | *Trichopilia turialbae* | EU213772 |
| Angiosperm | Orchidaceae | *Trichopilia turialbae* | EU213773 |
| Angiosperm | Orchidaceae | *Trichopilia turialbae* | EU213774 |
| Angiosperm | Plantaginaceae | *Plantago lanceolata* | EU750471 |
| Angiosperm | Plantaginaceae | *Plantago lanceolata* | EU750472 |
| Angiosperm | Plantaginaceae | *Plantago lanceolata* | EU750473 |
| Angiosperm | Plantaginaceae | *Plantago major* | EU750474 |
| Angiosperm | Plantaginaceae | *Plantago major* | EU750475 |
| Angiosperm | Plantaginaceae | *Plantago major* | EU750476 |
| Angiosperm | Plantaginaceae | *Plantago rugelii* | EU750477 |
| Angiosperm | Plantaginaceae | *Plantago rugelii* | EU750478 |
| Angiosperm | Poaceae | *Achnatherum altum* | EU204651 |
| Angiosperm | Poaceae | *Achnatherum calamagrostis* | EU204652 |
| Angiosperm | Poaceae | *Achnatherum caragana* | EU204654 |
| Angiosperm | Poaceae | *Achnatherum hymenoides* | EU204657 |
| Angiosperm | Poaceae | *Achnatherum lobatum* | EU204658 |
| Angiosperm | Poaceae | *Achnatherum nelsonii* | EU204660 |
| Angiosperm | Poaceae | *Achnatherum nevadense* | EU204661 |
| Angiosperm | Poaceae | *Achnatherum occidentale* | EU204662 |
| Angiosperm | Poaceae | *Achnatherum occidentale* subsp. *californicum* | EU204664 |
| Angiosperm | Poaceae | *Achnatherum richardsonii* | EU204665 |
| Angiosperm | Poaceae | *Achnatherum robustum* | EU204666 |
| Angiosperm | Poaceae | *Achnatherum stillmanii* | EU204653 |
| Angiosperm | Poaceae | *Acidosasa purpurea* | FJ644248 |
| Angiosperm | Poaceae | *Amelichloa brachychaeta* | EU204674 |
| Angiosperm | Poaceae | *Amelichloa caudata* | EU204679 |
| Angiosperm | Poaceae | *Amelichloa clandestina* | EU204683 |
| Angiosperm | Poaceae | *Ampelodesmos mauritanicus* | EU204684 |
| Angiosperm | Poaceae | *Anatherostipa bomanii* | EU204685 |
| Angiosperm | Poaceae | *Anatherostipa bomanii* | EU204686 |
| Angiosperm | Poaceae | *Anatherostipa obtusa* | EU204687 |
| Angiosperm | Poaceae | *Anatherostipa orurensis* | EU204688 |
| Angiosperm | Poaceae | *Anatherostipa rosea* | EU204689 |
| Angiosperm | Poaceae | *Arthraxon hispidus* | DQ006213 |
| Angiosperm | Poaceae | *Arundo donax* | EU531719 |
| Angiosperm | Poaceae | *Arundo pliniana* | EU531720 |
| Angiosperm | Poaceae | *Austrostipa aristiglumis* | EU204690 |
| Angiosperm | Poaceae | *Austrostipa bigeniculata* | EU204691 |
| Angiosperm | Poaceae | *Austrostipa elegantissima* | EU204692 |
| Angiosperm | Poaceae | *Austrostipa nitida* | EU204693 |
| Angiosperm | Poaceae | *Austrostipa ramosissima* | EU204694 |
| Angiosperm | Poaceae | *Austrostipa scabra* | EU204695 |
| Angiosperm | Poaceae | *Austrostipa scabra* | EU204696 |
| Angiosperm | Poaceae | *Bambusa oldhamii* | AB331267 |
| Angiosperm | Poaceae | *Bambusa vulgaris* | FJ644251 |
| Angiosperm | Poaceae | *Brachyelytrum erectum* | FJ644252 |
| Angiosperm | Poaceae | *Buergersiochloa bambusoides* | FJ644253 |
| Angiosperm | Poaceae | *Celtica gigantea* | EU204698 |
| Angiosperm | Poaceae | *Chasmanthium latifolium* | DQ006214 |
| Angiosperm | Poaceae | *Chimonobambusa marmorea* | FJ644254 |
| Angiosperm | Poaceae | *Chimonocalamus pallens* | FJ644255 |
| Angiosperm | Poaceae | *Cynodon dactylon* | EU531721 |
| Angiosperm | Poaceae | *Digitaria sanguinalis* | AB331271 |
| Angiosperm | Poaceae | *Elymus repens* | EU531722 |
| Angiosperm | Poaceae | *Ferrocalamus strictus* | FJ644256 |
| Angiosperm | Poaceae | *Guadua angustifolia* | FJ644257 |
| Angiosperm | Poaceae | *Hesperostipa comata* subsp. *comata* | EU204699 |
| Angiosperm | Poaceae | *Hesperostipa comata* subsp. *intermedia* | EU204700 |
| Angiosperm | Poaceae | *Hesperostipa curtiseta* | EU204701 |
| Angiosperm | Poaceae | *Indosasa sinica* | FJ644258 |
| Angiosperm | Poaceae | *Jarava chrysophylla* | EU204730 |
| Angiosperm | Poaceae | *Jarava eriostachya* | EU204702 |
| Angiosperm | Poaceae | *Jarava eriostachya* | EU204703 |
| Angiosperm | Poaceae | *Jarava eriostachya* | EU204704 |
| Angiosperm | Poaceae | *Jarava eriostachya* | EU204705 |
| Angiosperm | Poaceae | *Jarava humilis* | EU204733 |
| Angiosperm | Poaceae | *Jarava sorianoi* | EU204735 |
| Angiosperm | Poaceae | *Jarava speciosa* | EU204736 |
| Angiosperm | Poaceae | *Jarava speciosa* | EU204737 |
| Angiosperm | Poaceae | *Jarava speciosa* | EU204738 |
| Angiosperm | Poaceae | *Jarava speciosa* | EU204739 |
| Angiosperm | Poaceae | *Jarava speciosa* | EU204741 |
| Angiosperm | Poaceae | *Jarava speciosa* | EU204742 |
| Angiosperm | Poaceae | *Jarava speciosa* | EU204744 |
| Angiosperm | Poaceae | *Jarava speciosa* | EU204745 |
| Angiosperm | Poaceae | *Jarava speciosa* var. *major* | EU204743 |
| Angiosperm | Poaceae | *Jarava speciosa* var. *major* | EU204746 |
| Angiosperm | Poaceae | *Jarava vaginata f. inmersa* | EU204753 |
| Angiosperm | Poaceae | *Jarava vaginata* var. *argyroidea* | EU204751 |
| Angiosperm | Poaceae | *Leymus akmolinensis* | EF485570 |
| Angiosperm | Poaceae | *Leymus akmolinensis* | EF485571 |
| Angiosperm | Poaceae | *Leymus ambiguus* | EF485572 |
| Angiosperm | Poaceae | *Leymus ambiguus* | EF485573 |
| Angiosperm | Poaceae | *Leymus ambiguus* | EF485574 |
| Angiosperm | Poaceae | *Leymus ambiguus* | EF485577 |
| Angiosperm | Poaceae | *Leymus angustus* | EF485578 |
| Angiosperm | Poaceae | *Leymus arenarius* | EF485580 |
| Angiosperm | Poaceae | *Leymus arenarius* | EF485581 |
| Angiosperm | Poaceae | *Leymus chinensis* | EF485582 |
| Angiosperm | Poaceae | *Leymus chinensis* | EF485583 |
| Angiosperm | Poaceae | *Leymus cinereus* | EF485584 |
| Angiosperm | Poaceae | *Leymus cinereus* | EF485585 |
| Angiosperm | Poaceae | *Leymus cinereus* | EF485586 |
| Angiosperm | Poaceae | *Leymus cinereus* | EF485587 |
| Angiosperm | Poaceae | *Leymus cinereus* | EF485591 |
| Angiosperm | Poaceae | *Leymus cinereus* | EF485592 |
| Angiosperm | Poaceae | *Leymus cinereus* | EF485593 |
| Angiosperm | Poaceae | *Leymus condensatus* | EF485843 |
| Angiosperm | Poaceae | *Leymus flavescens* | EF485845 |
| Angiosperm | Poaceae | *Leymus flavescens* | EF485846 |
| Angiosperm | Poaceae | *Leymus innovatus* | EF485847 |
| Angiosperm | Poaceae | *Leymus mollis* | EF485855 |
| Angiosperm | Poaceae | *Leymus multicaulis* | EF485856 |
| Angiosperm | Poaceae | *Leymus multicaulis* | EF485857 |
| Angiosperm | Poaceae | *Leymus multicaulis* | EF485881 |
| Angiosperm | Poaceae | *Leymus multicaulis* | EF485882 |
| Angiosperm | Poaceae | *Leymus racemosus* | EF485862 |
| Angiosperm | Poaceae | *Leymus ramosus* | EF485865 |
| Angiosperm | Poaceae | *Leymus ramosus* | EF485866 |
| Angiosperm | Poaceae | *Leymus sabulosus* | EF485869 |
| Angiosperm | Poaceae | *Leymus salinus* | EF485870 |
| Angiosperm | Poaceae | *Leymus salinus* | EF485873 |
| Angiosperm | Poaceae | *Leymus salinus* subsp. *mojavensis* | EF485853 |
| Angiosperm | Poaceae | *Leymus secalinus* | EF485876 |
| Angiosperm | Poaceae | *Leymus secalinus* | EF485880 |
| Angiosperm | Poaceae | *Leymus triticoides* | EF485860 |
| Angiosperm | Poaceae | *Leymus triticoides* | EF485889 |
| Angiosperm | Poaceae | *Nassella chilensis* | EU204712 |
| Angiosperm | Poaceae | *Nassella cordobensis* | EU204713 |
| Angiosperm | Poaceae | *Nassella cordobensis* | EU204714 |
| Angiosperm | Poaceae | *Nassella neesiana* | EU204719 |
| Angiosperm | Poaceae | *Nassella punensis* | EU204722 |
| Angiosperm | Poaceae | *Nassella tenuis* | EU204723 |
| Angiosperm | Poaceae | *Piptatherum canadense* | EU204754 |
| Angiosperm | Poaceae | *Piptatherum holciforme* | EU204755 |
| Angiosperm | Poaceae | *Piptatherum holciforme* | EU204756 |
| Angiosperm | Poaceae | *Piptatherum micranthum* | EU204757 |
| Angiosperm | Poaceae | *Piptatherum microcarpum* | EU204758 |
| Angiosperm | Poaceae | *Piptatherum miliaceum* | EU204759 |
| Angiosperm | Poaceae | *Piptatherum miliaceum* | EU204760 |
| Angiosperm | Poaceae | *Piptatherum miliaceum* | EU204761 |
| Angiosperm | Poaceae | *Piptatherum racemosum* | EU204762 |
| Angiosperm | Poaceae | *Piptatherum virescens* | EU204763 |
| Angiosperm | Poaceae | *Poa annua* | EU750479 |
| Angiosperm | Poaceae | *Poa annua* | EU750480 |
| Angiosperm | Poaceae | *Poa annua* | FJ493300 |
| Angiosperm | Poaceae | *Poa compressa* | EU750481 |
| Angiosperm | Poaceae | *Poa compressa* | EU750482 |
| Angiosperm | Poaceae | *Poa compressa* | EU750483 |
| Angiosperm | Poaceae | *Ptilagrostis kingii* | EU204769 |
| Angiosperm | Poaceae | *Ptilagrostis pelliotii* | EU204770 |
| Angiosperm | Poaceae | *Ptilagrostis porteri* | EU204771 |
| Angiosperm | Poaceae | *Stipa barbata* | EU204773 |
| Angiosperm | Poaceae | *Stipa cazorlensis* | EU204774 |
| Angiosperm | Poaceae | *Stipa iberica* | EU204775 |
| Angiosperm | Poaceae | *Stipa juncea* | EU204776 |
| Angiosperm | Polygonaceae | *Bistorta amplexicaulis* | EF653739 |
| Angiosperm | Polygonaceae | *Bistorta paleacea* | EF653740 |
| Angiosperm | Polygonaceae | *Fagopyrum esculentum* | EF653736 |
| Angiosperm | Polygonaceae | *Fallopia convolvulus* | EU750488 |
| Angiosperm | Polygonaceae | *Fallopia convolvulus* | EU750489 |
| Angiosperm | Polygonaceae | *Persicaria acuminata* | EU196966 |
| Angiosperm | Polygonaceae | *Persicaria arifolia* | EF653744 |
| Angiosperm | Polygonaceae | *Persicaria barbata* | EU196967 |
| Angiosperm | Polygonaceae | *Persicaria bicornis* | EU196968 |
| Angiosperm | Polygonaceae | *Persicaria careyi* | EU196969 |
| Angiosperm | Polygonaceae | *Persicaria densiflora* | EU196970 |
| Angiosperm | Polygonaceae | *Persicaria ferruginea* | EU196971 |
| Angiosperm | Polygonaceae | *Persicaria foliosa* | EU196972 |
| Angiosperm | Polygonaceae | *Persicaria glabra* | EU196973 |
| Angiosperm | Polygonaceae | *Persicaria hirsuta* | EU196975 |
| Angiosperm | Polygonaceae | *Persicaria hirsuta* | EU196976 |
| Angiosperm | Polygonaceae | *Persicaria hydropiper* | EF653753 |
| Angiosperm | Polygonaceae | *Persicaria hydropiper* | EF653754 |
| Angiosperm | Polygonaceae | *Persicaria hydropiper* | EU750490 |
| Angiosperm | Polygonaceae | *Persicaria hydropiper* | EU750491 |
| Angiosperm | Polygonaceae | *Persicaria hydropiperoides* | EU196980 |
| Angiosperm | Polygonaceae | *Persicaria japonica* | EU196981 |
| Angiosperm | Polygonaceae | *Persicaria kawagoeana* | EU196982 |
| Angiosperm | Polygonaceae | *Persicaria limbata* | EU196985 |
| Angiosperm | Polygonaceae | *Persicaria maackiana* | EF653745 |
| Angiosperm | Polygonaceae | *Persicaria maculosa* | EU750492 |
| Angiosperm | Polygonaceae | *Persicaria maculosa* | EU750493 |
| Angiosperm | Polygonaceae | *Persicaria maculosa* | EU750494 |
| Angiosperm | Polygonaceae | *Persicaria meisneriana* | EF653746 |
| Angiosperm | Polygonaceae | *Persicaria mexicana* | EU196989 |
| Angiosperm | Polygonaceae | *Persicaria minor* | EU196990 |
| Angiosperm | Polygonaceae | *Persicaria minor* | EU196991 |
| Angiosperm | Polygonaceae | *Persicaria nepalensis* | EF653742 |
| Angiosperm | Polygonaceae | *Persicaria opelousana* | EU196993 |
| Angiosperm | Polygonaceae | *Persicaria pensylvanica* | EF653756 |
| Angiosperm | Polygonaceae | *Persicaria posumbu* | EU196996 |
| Angiosperm | Polygonaceae | *Persicaria punctata* | EF653757 |
| Angiosperm | Polygonaceae | *Persicaria punctata* | EU196998 |
| Angiosperm | Polygonaceae | *Persicaria puritanorum* | EU196999 |
| Angiosperm | Polygonaceae | *Persicaria robustior* | EU197000 |
| Angiosperm | Polygonaceae | *Persicaria sagittata* | EF653747 |
| Angiosperm | Polygonaceae | *Persicaria segetum* | EU197001 |
| Angiosperm | Polygonaceae | *Persicaria senegalensis* | EU197002 |
| Angiosperm | Polygonaceae | *Persicaria taquetii* | EU197004 |
| Angiosperm | Polygonaceae | *Persicaria tinctoria* | EU197005 |
| Angiosperm | Polygonaceae | *Persicaria tomentosa* | EU197006 |
| Angiosperm | Polygonaceae | *Persicaria virginiana* var. *filiformis* | EF653748 |
| Angiosperm | Polygonaceae | *Persicaria viscofera* | EU197007 |
| Angiosperm | Polygonaceae | *Polygonella articulata* | EF653734 |
| Angiosperm | Polygonaceae | *Polygonum aviculare* | EU750484 |
| Angiosperm | Polygonaceae | *Polygonum aviculare* | EU750485 |
| Angiosperm | Polygonaceae | *Polygonum aviculare* | EU750486 |
| Angiosperm | Polygonaceae | *Polygonum aviculare* | EU750487 |
| Angiosperm | Polygonaceae | *Polygonum capitatum* | EF653741 |
| Angiosperm | Polygonaceae | *Polygonum paraguayense* | EU196995 |
| Angiosperm | Polygonaceae | *Polygonum runcinatum* | EF653743 |
| Angiosperm | Proteaceae | *Faurea rochetiana* | EU213813 |
| Angiosperm | Proteaceae | *Faurea rochetiana* | EU213814 |
| Angiosperm | Proteaceae | *Faurea rochetiana* | EU213815 |
| Angiosperm | Proteaceae | *Faurea saligna* | EU213816 |
| Angiosperm | Proteaceae | *Faurea saligna* | EU213817 |
| Angiosperm | Proteaceae | *Faurea saligna* | EU213818 |
| Angiosperm | Ranunculaceae | *Aconitum anthora* | AF216570 |
| Angiosperm | Ranunculaceae | *Aconitum lycoctonum* | AF216560 |
| Angiosperm | Ranunculaceae | *Aconitum lycoctonum* | AF216561 |
| Angiosperm | Ranunculaceae | *Aconitum lycoctonum* | AF216563 |
| Angiosperm | Ranunculaceae | *Aconitum lycoctonum* | AF216564 |
| Angiosperm | Ranunculaceae | *Aconitum lycoctonum* | AF216565 |
| Angiosperm | Ranunculaceae | *Aconitum lycoctonum* | AF216568 |
| Angiosperm | Ranunculaceae | *Aconitum lycoctonum* | AF216569 |
| Angiosperm | Ranunculaceae | *Aconitum lycoctonum* | AF216573 |
| Angiosperm | Ranunculaceae | *Aconitum moldavicum* | AF216558 |
| Angiosperm | Ranunculaceae | *Aconitum septentrionale* | AF216575 |
| Angiosperm | Ranunculaceae | *Anemone amurensis* | EF139299 |
| Angiosperm | Ranunculaceae | *Anemone amurensis* | EF139300 |
| Angiosperm | Ranunculaceae | *Anemone amurensis* | EF139301 |
| Angiosperm | Ranunculaceae | *Anemone amurensis* | EF139302 |
| Angiosperm | Ranunculaceae | *Anemone amurensis* | EF139303 |
| Angiosperm | Ranunculaceae | *Anemone amurensis* | EF139304 |
| Angiosperm | Ranunculaceae | *Anemone amurensis* | EF139305 |
| Angiosperm | Ranunculaceae | *Anemone amurensis* | EF139306 |
| Angiosperm | Ranunculaceae | *Anemone amurensis* | EF139307 |
| Angiosperm | Ranunculaceae | *Anemone pendulisepala* | EF139289 |
| Angiosperm | Ranunculaceae | *Anemone pendulisepala* | EF139298 |
| Angiosperm | Ranunculaceae | *Anemone raddeana* | EF139280 |
| Angiosperm | Ranunculaceae | *Anemone raddeana* | EF139288 |
| Angiosperm | Ranunculaceae | *Anemone reflexa* | EF139308 |
| Angiosperm | Ranunculaceae | *Anemone reflexa* | EF139313 |
| Angiosperm | Ranunculaceae | *Anemone stolonifera* | EF139278 |
| Angiosperm | Ranunculaceae | *Anemone stolonifera* | EF139279 |
| Angiosperm | Ranunculaceae | *Consolida ajacis* | AF216578 |
| Angiosperm | Ranunculaceae | *Coptis aspleniifolia* | AB163747 |
| Angiosperm | Ranunculaceae | *Coptis chinensis* | AB163745 |
| Angiosperm | Ranunculaceae | *Coptis deltoidea* | AB163744 |
| Angiosperm | Ranunculaceae | *Coptis japonica* var. *anemonifolia* | AB163752 |
| Angiosperm | Ranunculaceae | *Coptis japonica* var. *japonica* | AB163751 |
| Angiosperm | Ranunculaceae | *Coptis japonica* var. *major* | AB163753 |
| Angiosperm | Ranunculaceae | *Coptis laciniata* | AB163748 |
| Angiosperm | Ranunculaceae | *Coptis lutescens* | AB159519 |
| Angiosperm | Ranunculaceae | *Coptis occidentalis* | AB163749 |
| Angiosperm | Ranunculaceae | *Coptis quinquefolia* | AB159523 |
| Angiosperm | Ranunculaceae | *Coptis quinquefolia* | AB159526 |
| Angiosperm | Ranunculaceae | *Coptis ramosa* | AB159534 |
| Angiosperm | Ranunculaceae | *Coptis trifolia* | AB159539 |
| Angiosperm | Ranunculaceae | *Coptis trifolia* | AB159541 |
| Angiosperm | Ranunculaceae | *Coptis trifoliolata* | AB159530 |
| Angiosperm | Rhamnaceae | *Frangula alnus* | EU750522 |
| Angiosperm | Rhamnaceae | *Frangula alnus* | EU750523 |
| Angiosperm | Rhamnaceae | *Rhamnus alnifolia* | EU750516 |
| Angiosperm | Rhamnaceae | *Rhamnus alnifolia* | EU750517 |
| Angiosperm | Rhamnaceae | *Rhamnus cathartica* | EU750518 |
| Angiosperm | Rhamnaceae | *Rhamnus cathartica* | EU750519 |
| Angiosperm | Rhamnaceae | *Rhamnus cathartica* | EU750520 |
| Angiosperm | Rhamnaceae | *Rhamnus cathartica* | EU750521 |
| Angiosperm | Rosaceae | *Cliffortia acanthophylla* | EU937617 |
| Angiosperm | Rosaceae | *Cliffortia acutifolia* | EU937605 |
| Angiosperm | Rosaceae | *Cliffortia alata* | EU937722 |
| Angiosperm | Rosaceae | *Cliffortia amplexistipula* | EU937660 |
| Angiosperm | Rosaceae | *Cliffortia amplexistipula* | EU937661 |
| Angiosperm | Rosaceae | *Cliffortia arborea* | EU937569 |
| Angiosperm | Rosaceae | *Cliffortia arborea* | EU937570 |
| Angiosperm | Rosaceae | *Cliffortia arcuata* | EU937718 |
| Angiosperm | Rosaceae | *Cliffortia baccans* | EU937720 |
| Angiosperm | Rosaceae | *Cliffortia burchellii* | EU937640 |
| Angiosperm | Rosaceae | *Cliffortia burgersii* | EU937723 |
| Angiosperm | Rosaceae | *Cliffortia carinata* | EU937664 |
| Angiosperm | Rosaceae | *Cliffortia castanea* | EU937648 |
| Angiosperm | Rosaceae | *Cliffortia ceresana* | EU937618 |
| Angiosperm | Rosaceae | *Cliffortia complanata* | EU937592 |
| Angiosperm | Rosaceae | *Cliffortia conifera* | EU937571 |
| Angiosperm | Rosaceae | *Cliffortia crenata* | EU937665 |
| Angiosperm | Rosaceae | *Cliffortia cristata* | EU937689 |
| Angiosperm | Rosaceae | *Cliffortia curvifolia* | EU937585 |
| Angiosperm | Rosaceae | *Cliffortia cymbifolia* | EU937654 |
| Angiosperm | Rosaceae | *Cliffortia dentata* | EU937586 |
| Angiosperm | Rosaceae | *Cliffortia denticulata* | EU937627 |
| Angiosperm | Rosaceae | *Cliffortia dodecandra* | EU937652 |
| Angiosperm | Rosaceae | *Cliffortia dracomontana* | EU937696 |
| Angiosperm | Rosaceae | *Cliffortia dregeana* | EU937616 |
| Angiosperm | Rosaceae | *Cliffortia dregeana x Cliffortia pungens* | EU937619 |
| Angiosperm | Rosaceae | *Cliffortia erectisepala* | EU937643 |
| Angiosperm | Rosaceae | *Cliffortia erectisepala* | EU937644 |
| Angiosperm | Rosaceae | *Cliffortia eriocephalina* | EU937573 |
| Angiosperm | Rosaceae | *Cliffortia eriocephalina* | EU937574 |
| Angiosperm | Rosaceae | *Cliffortia ferricola* | EU937658 |
| Angiosperm | Rosaceae | *Cliffortia ferruginea* | EU937603 |
| Angiosperm | Rosaceae | *Cliffortia geniculata* | EU937674 |
| Angiosperm | Rosaceae | *Cliffortia grandifolia* | EU937633 |
| Angiosperm | Rosaceae | *Cliffortia heterophylla* | EU937635 |
| Angiosperm | Rosaceae | *Cliffortia hexandra* | EU937688 |
| Angiosperm | Rosaceae | *Cliffortia hirsuta* | EU937599 |
| Angiosperm | Rosaceae | *Cliffortia incana* | EU937711 |
| Angiosperm | Rosaceae | *Cliffortia integerrima* | EU937629 |
| Angiosperm | Rosaceae | *Cliffortia intermedia* | EU937624 |
| Angiosperm | Rosaceae | *Cliffortia intermedia* | EU937625 |
| Angiosperm | Rosaceae | *Cliffortia juniperina* var. *juniperina* | EU937682 |
| Angiosperm | Rosaceae | *Cliffortia juniperina* var. *pilosula* | EU937683 |
| Angiosperm | Rosaceae | *Cliffortia lanata* | EU937687 |
| Angiosperm | Rosaceae | *Cliffortia lanceolata* | EU937634 |
| Angiosperm | Rosaceae | *Cliffortia linearifolia* | EU937691 |
| Angiosperm | Rosaceae | *Cliffortia marginata* | EU937669 |
| Angiosperm | Rosaceae | *Cliffortia mirabilis* | EU937666 |
| Angiosperm | Rosaceae | *Cliffortia montana* | EU937576 |
| Angiosperm | Rosaceae | *Cliffortia montana* | EU937577 |
| Angiosperm | Rosaceae | *Cliffortia multiformis* | EU937649 |
| Angiosperm | Rosaceae | *Cliffortia neglecta* | EU937646 |
| Angiosperm | Rosaceae | *Cliffortia nitidula* subsp. *pilosa* | EU937693 |
| Angiosperm | Rosaceae | *Cliffortia nitidula* subsp. *pilosa* | EU937694 |
| Angiosperm | Rosaceae | *Cliffortia obcordata* | EU937659 |
| Angiosperm | Rosaceae | *Cliffortia odorata* | EU937597 |
| Angiosperm | Rosaceae | *Cliffortia oligodonta* | EU937623 |
| Angiosperm | Rosaceae | *Cliffortia ovalis* | EU937622 |
| Angiosperm | Rosaceae | *Cliffortia phillipsii* | EU937636 |
| Angiosperm | Rosaceae | *Cliffortia phyllanthoides* | EU937667 |
| Angiosperm | Rosaceae | *Cliffortia polita* | EU937678 |
| Angiosperm | Rosaceae | *Cliffortia polygonifolia* var. *polygonifolia* | EU937684 |
| Angiosperm | Rosaceae | *Cliffortia polygonifolia* var. *trifoliata* | EU937685 |
| Angiosperm | Rosaceae | *Cliffortia prionota* | EU937626 |
| Angiosperm | Rosaceae | *Cliffortia propinqua* | EU937591 |
| Angiosperm | Rosaceae | *Cliffortia pterocarpa* | EU937650 |
| Angiosperm | Rosaceae | *Cliffortia pungens* | EU937607 |
| Angiosperm | Rosaceae | *Cliffortia repens* | EU937645 |
| Angiosperm | Rosaceae | *Cliffortia reticulata* | EU937598 |
| Angiosperm | Rosaceae | *Cliffortia robusta* | EU937647 |
| Angiosperm | Rosaceae | *Cliffortia ruscifolia* var. *ruscifolia* | EU937653 |
| Angiosperm | Rosaceae | *Cliffortia ruscifolia x Cliffortia teretifolia* | EU937727 |
| Angiosperm | Rosaceae | *Cliffortia ruscifolia x Cliffortia theodori-friesii* | EU937724 |
| Angiosperm | Rosaceae | *Cliffortia scandens* | EU937637 |
| Angiosperm | Rosaceae | *Cliffortia sericea* | EU937686 |
| Angiosperm | Rosaceae | *Cliffortia sparsa* | EU937690 |
| Angiosperm | Rosaceae | *Cliffortia strigosa* | EU937621 |
| Angiosperm | Rosaceae | *Cliffortia tenuis* | EU937670 |
| Angiosperm | Rosaceae | *Cliffortia theodori-friesii* | EU937631 |
| Angiosperm | Rosaceae | *Cliffortia tricuspidata* | EU937602 |
| Angiosperm | Rosaceae | *Cliffortia varians* | EU937676 |
| Angiosperm | Rosaceae | *Cliffortia verrucosa* | EU937657 |
| Angiosperm | Rosaceae | *Cliffortia virgata* | EU937620 |
| Angiosperm | Rosaceae | *Rubus allegheniensis* | EU750524 |
| Angiosperm | Rosaceae | *Rubus allegheniensis* | EU750525 |
| Angiosperm | Rosaceae | *Rubus allegheniensis* | EU750526 |
| Angiosperm | Rosaceae | *Rubus idaeus* | EU750527 |
| Angiosperm | Rosaceae | *Rubus idaeus* | EU750528 |
| Angiosperm | Rosaceae | *Rubus idaeus* | EU750529 |
| Angiosperm | Rosaceae | *Rubus idaeus* | EU750530 |
| Angiosperm | Rosaceae | *Rubus idaeus* | EU750531 |
| Angiosperm | Rosaceae | *Rubus occidentalis* | EU750532 |
| Angiosperm | Rosaceae | *Rubus occidentalis* | EU750533 |
| Angiosperm | Rosaceae | *Rubus occidentalis* | EU750534 |
| Angiosperm | Rosaceae | *Rubus occidentalis* | EU750535 |
| Angiosperm | Rosaceae | *Rubus odoratus* | EU750536 |
| Angiosperm | Rosaceae | *Rubus odoratus* | EU750537 |
| Angiosperm | Salicaceae | *Populus balsamifera* | EU750495 |
| Angiosperm | Salicaceae | *Populus balsamifera* | EU750496 |
| Angiosperm | Salicaceae | *Populus grandidentata* | EU750500 |
| Angiosperm | Salicaceae | *Populus grandidentata* | EU750501 |
| Angiosperm | Salicaceae | *Populus grandidentata* | EU750502 |
| Angiosperm | Salicaceae | *Populus tremuloides* | EU750503 |
| Angiosperm | Salicaceae | *Populus tremuloides* | EU750504 |
| Angiosperm | Salicaceae | *Populus tremuloides* | EU750505 |
| Angiosperm | Salicaceae | *Salix babylonica* | EU750538 |
| Angiosperm | Salicaceae | *Salix babylonica* | EU750539 |
| Angiosperm | Salicaceae | *Salix eriocephala* | EU750540 |
| Angiosperm | Salicaceae | *Salix eriocephala* | EU750541 |
| Angiosperm | Salicaceae | *Salix eriocephala* | EU750542 |
| Angiosperm | Salicaceae | *Salix eriocephala* | EU750543 |
| Angiosperm | Salicaceae | *Salix exigua* | EU750544 |
| Angiosperm | Salicaceae | *Salix exigua* | EU750545 |
| Angiosperm | Solanaceae | *Solanum dulcamara* | EU750551 |
| Angiosperm | Solanaceae | *Solanum dulcamara* | EU750552 |
| Angiosperm | Solanaceae | *Solanum dulcamara* | EU750553 |
| Angiosperm | Solanaceae | *Solanum nigrum* | EU750554 |
| Angiosperm | Solanaceae | *Solanum nigrum* | EU750555 |
| Angiosperm | Solanaceae | *Solanum nigrum* | EU750556 |
| Angiosperm | Solanaceae | *Solanum panduriforme* | EU213851 |
| Angiosperm | Solanaceae | *Solanum panduriforme* | EU213852 |
| Angiosperm | Solanaceae | *Solanum panduriforme* | EU213853 |
| Angiosperm | Taccaceae | *Tylophora aristolochioides* | AB109137 |
| Angiosperm | Taccaceae | *Tylophora brownii* | AB109138 |
| Angiosperm | Taccaceae | *Tylophora japonica* | AB109140 |
| Angiosperm | Taccaceae | *Tylophora matsumurae* | AB109143 |
| Angiosperm | Taccaceae | *Tylophora ovata* var. *brownii* | AB109141 |
| Angiosperm | Taccaceae | *Tylophora tanakae* | AB109144 |
| Angiosperm | Tiliaceae | *Grewia bicolor* | EU213828 |
| Angiosperm | Tiliaceae | *Grewia bicolor* | EU213829 |
| Angiosperm | Tiliaceae | *Grewia bicolor* | EU213830 |
| Angiosperm | Tiliaceae | *Grewia flavescens* | EU213831 |
| Angiosperm | Tiliaceae | *Grewia flavescens* | EU213832 |
| Angiosperm | Tiliaceae | *Grewia villosa* | EU213833 |
| Angiosperm | Tiliaceae | *Grewia villosa* | EU213834 |
| Angiosperm | Tiliaceae | *Grewia villosa* | EU213835 |
| Angiosperm | Typhaceae | *Typha angustifolia* | EU750603 |
| Angiosperm | Typhaceae | *Typha angustifolia* | EU750604 |
| Angiosperm | Typhaceae | *Typha latifolia* | EU750605 |
| Angiosperm | Typhaceae | *Typha latifolia* | EU750606 |
| Angiosperm | Velloziaceae | *Xerophyta retinervis* | EU213866 |
| Angiosperm | Velloziaceae | *Xerophyta retinervis* | EU213867 |
| Angiosperm | Velloziaceae | *Xerophyta retinervis* | EU213868 |
| Angiosperm | Verbenaceae | *Vitex negundo* | DQ304781 |
| Angiosperm | Verbenaceae | *Vitex negundo* | DQ304782 |
| Angiosperm | Verbenaceae | *Vitex negundo* | DQ304783 |
| Gymnosperm | Cupressaceae | *Calocedrus decurrens* | FJ493277 |
| Gymnosperm | Cupressaceae | *Chamaecyparis lawsoniana* | FJ493278 |
| Gymnosperm | Cupressaceae | *Juniperus communis* | EU750613 |
| Gymnosperm | Cupressaceae | *Juniperus communis* | EU750614 |
| Gymnosperm | Cupressaceae | *Juniperus communis* | EU750615 |
| Gymnosperm | Cupressaceae | *Juniperus communis* | EU750616 |
| Gymnosperm | Cupressaceae | *Juniperus virginiana* | EU750617 |
| Gymnosperm | Cupressaceae | *Juniperus virginiana* | EU750618 |
| Gymnosperm | Cupressaceae | *Juniperus virginiana* | EU750619 |
| Gymnosperm | Cupressaceae | *Juniperus virginiana* | EU750620 |
| Gymnosperm | Pinaceae | *Picea glauca* | EU750621 |
| Gymnosperm | Pinaceae | *Picea glauca* | EU750622 |
| Gymnosperm | Pinaceae | *Picea glauca* | EU750623 |
| Gymnosperm | Pinaceae | *Picea glauca* | EU750624 |
| Gymnosperm | Pinaceae | *Picea mariana* | EU750625 |
| Gymnosperm | Pinaceae | *Picea mariana* | EU750626 |
| Gymnosperm | Pinaceae | *Pinus strobus* | EU750629 |
| Gymnosperm | Pinaceae | *Pinus strobus* | EU750630 |
| Gymnosperm | Pinaceae | *Pinus strobus* | EU750631 |
| Gymnosperm | Pinaceae | *Pinus sylvestris* | EU750632 |
| Gymnosperm | Pinaceae | *Pinus sylvestris* | EU750633 |
| Gymnosperm | Pinaceae | *Pinus sylvestris* | EU750634 |
| Fern | Dryopteridaceae | *Dryopteris carthusiana* | EU750635 |
| Fern | Dryopteridaceae | *Dryopteris carthusiana* | EU750636 |
| Fern | Dryopteridaceae | *Dryopteris carthusiana* | EU750637 |
| Fern | Dryopteridaceae | *Dryopteris erythrosora* | EF590692 |
| Fern | Dryopteridaceae | *Dryopteris goldiana* | EF590693 |
| Fern | Dryopteridaceae | *Dryopteris intermedia* | EU750638 |
| Fern | Dryopteridaceae | *Dryopteris intermedia* | EU750639 |
| Fern | Dryopteridaceae | *Dryopteris intermedia* | EU750640 |
| Fern | Dryopteridaceae | *Dryopteris marginalis* | EU750641 |
| Fern | Dryopteridaceae | *Dryopteris marginalis* | EU750642 |
| Fern | Dryopteridaceae | *Dryopteris marginalis* | EU750643 |
| Fern | Dryopteridaceae | *Dryopteris marginalis* | EU750644 |
| Fern | Equisetaceae | *Equisetum arvense* | EU750645 |
| Fern | Equisetaceae | *Equisetum arvense* | EU750646 |
| Fern | Equisetaceae | *Equisetum hyemale* | EU750647 |
| Fern | Equisetaceae | *Equisetum hyemale* | EU750648 |
| Fern | Equisetaceae | *Equisetum hyemale* | EU750649 |
| Fern | Huperziaceae | *Huperzia carinata* | DQ464213 |
| Fern | Huperziaceae | *Huperzia fargesii* | DQ464214 |
| Fern | Huperziaceae | *Huperzia fordii* | DQ464215 |
| Fern | Huperziaceae | *Huperzia mingcheensis* | DQ464216 |
| Fern | Huperziaceae | *Huperzia petiolata* | DQ464217 |
| Fern | Huperziaceae | *Huperzia phyllantha* | DQ464218 |
| Fern | Huperziaceae | *Huperzia squarrosa* | DQ464219 |
| Fern | Lycopodiaceae | *Diphasiastrum digitatum* | EU750650 |
| Fern | Lycopodiaceae | *Diphasiastrum digitatum* | EU750651 |
| Fern | Lycopodiaceae | *Lycopodium obscurum* | EU750652 |
| Fern | Lycopodiaceae | *Lycopodium obscurum* | EU750653 |
| Fern | Lycopodiaceae | *Lycopodium obscurum* | EU750654 |
| Fern | Lycopodiaceae | *Lycopodium obscurum* | EU750655 |
| Moss | Aytoniaceae | *Asterella lindenbergiana* | FJ173595 |
| Moss | Aytoniaceae | *Asterella wallichiana* | FJ173555 |
| Moss | Aytoniaceae | *Cryptomitrium tenerum* | FJ173556 |
| Moss | Brachytheciaceae | *Brachythecium oxycladon* | EU750656 |
| Moss | Brachytheciaceae | *Brachythecium oxycladon* | EU750657 |
| Moss | Brachytheciaceae | *Brachythecium salebrosum* | EU750660 |
| Moss | Brachytheciaceae | *Brachythecium salebrosum* | EU750661 |
| Moss | Dicranaceae | *Dicranum flagellare* | EU750662 |
| Moss | Dicranaceae | *Dicranum flagellare* | EU750663 |
| Moss | Dicranaceae | *Dicranum flagellare* | EU750664 |
| Moss | Dicranaceae | *Dicranum polysetum* | EU750665 |
| Moss | Dicranaceae | *Dicranum polysetum* | EU750666 |
| Moss | Marchantiaceae | *Marchantia polymorpha* | FJ572500 |
| Moss | Mniaceae | *Plagiomnium cuspidatum* | EU750667 |
| Moss | Mniaceae | *Plagiomnium cuspidatum* | EU750668 |
| Moss | Mniaceae | *Plagiomnium drummondii* | EU750669 |
| Moss | Mniaceae | *Plagiomnium drummondii* | EU750670 |
| Moss | Polytrichaceae | *Polytrichum commune* | EU750671 |
| Moss | Polytrichaceae | *Polytrichum commune* | EU750672 |
| Moss | Polytrichaceae | *Polytrichum juniperinum* | EU750673 |
| Moss | Polytrichaceae | *Polytrichum juniperinum* | EU750674 |
| Moss | Polytrichaceae | *Polytrichum juniperinum* | EU750675 |
| Moss | Sphagnaceae | *Sphagnum palustre* | AY312920 |
| Moss | Sphagnaceae | *Sphagnum quinquefarium* | AY309617 |
| Moss | Sphagnaceae | *Sphagnum teres* | AY309625 |
| Moss | Sphagnaceae | *Sphagnum wulfianum* | AY309626 |
